# Homer1 promotes dendritic spine growth through ankyrin-G and its loss reshapes the synaptic proteome

**DOI:** 10.1038/s41380-020-00991-1

**Published:** 2021-01-04

**Authors:** Sehyoun Yoon, Nicolas H. Piguel, Natalia Khalatyan, Leonardo E. Dionisio, Jeffrey N. Savas, Peter Penzes

**Affiliations:** 1 Department of Physiology, Northwestern University Feinberg School of Medicine, Chicago, IL 60611 USA; 2 Department of Neurology, Northwestern University Feinberg School of Medicine, Chicago, IL 60611 USA; 3 Department of Psychiatry and Behavioral Sciences, Northwestern University Feinberg School of Medicine, Chicago, IL 60611 USA; 4 Northwestern University, Center for Autism and Neurodevelopment, Chicago, IL 60611 USA; 5 Present Address: Neuroscience Interdepartmental Program, David Geffen School of Medicine, University of California, Los Angeles, CA 90095 USA

**Keywords:** Neuroscience, Molecular biology

## Abstract

Homer1 is a synaptic scaffold protein that regulates glutamatergic synapses and spine morphogenesis. *HOMER1* knockout (KO) mice show behavioral abnormalities related to psychiatric disorders, and *HOMER1* has been associated with psychiatric disorders such as addiction, autism disorder (ASD), schizophrenia (SZ), and depression. However, the mechanisms by which it promotes spine stability and its global function in maintaining the synaptic proteome has not yet been fully investigated. Here, we used computational approaches to identify global functions for proteins containing the Homer1-interacting PPXXF motif within the postsynaptic compartment. Ankyrin-G was one of the most topologically important nodes in the postsynaptic peripheral membrane subnetwork, and we show that one of the PPXXF motifs, present in the postsynaptically-enriched 190 kDa isoform of ankyrin-G (ankyrin-G 190), is recognized by the EVH1 domain of Homer1. We use proximity ligation combined with super-resolution microscopy to map the interaction of ankyrin-G and Homer1 to distinct nanodomains within the spine head and correlate them with spine head size. This interaction motif is critical for ankyrin-G 190’s ability to increase spine head size, and for the maintenance of a stable ankyrin-G pool in spines. Intriguingly, lack of Homer1 significantly upregulated the abundance of ankyrin-G, but downregulated Shank3 in cortical crude plasma membrane fractions. In addition, proteomic analysis of the cortex in *HOMER1* KO and wild-type (WT) mice revealed a global reshaping of the postsynaptic proteome, surprisingly characterized by extensive upregulation of synaptic proteins. Taken together, we show that Homer1 and its protein interaction motif have broad global functions within synaptic protein-protein interaction networks. Enrichment of disease risk factors within these networks has important implications for neurodevelopmental disorders including bipolar disorder, ASD, and SZ.

## Introduction

The structural plasticity of dendritic spines is governed by several processes including spine formation, maturation, stabilization, remodeling, and elimination. Changes in spine number and morphology, along with the glutamate receptor content of synapses, contribute to functional connectivity in synaptic circuits [1]. Altered spine density in cortical pyramidal neurons has been observed in postmortem studies of patients with schizophrenia (SZ), bipolar disorder (BD), autism spectrum disorder (ASD), and intellectual disability (ID) [2–5]. Thus, dendritic spine abnormalities on cortical pyramidal neurons have emerged as key cellular substrates in the pathogenesis of several psychiatric disorders [6, 7].

The Homer family consists of three members in mammals, Homer1, Homer2, and Homer3, all of which are postsynaptic density scaffolding proteins highly expressed in the central nervous system. Homer1 protein regulates glutamatergic synapses and spine morphogenesis [8–10]. *HOMER1* is associated with ASD [11], SZ [12], and depression [13] in human cohorts and, *HOMER1* KO mice show relevant behavioral abnormalities related to the above psychiatric disorders [14, 15]. The Homer1 EVH1 domain interacts with the PPXXF motif [16] and the longer isoforms of Homer1b/c include the coiled-coiled C-terminus which mediates its multimerization [17]. Multimerized Homer1b/c forms a mesh-like matrix structure in dendritic spines and serves as an assembly platform for Shank proteins to positively regulate spine morphology [18]. However, the complete list of Homer1-interacting proteins has not yet been identified and the mechanisms by which Homer1 regulates spine structure are not fully understood. To identify novel Homer1 interaction partners we performed an in silico screen for proteins encoded by neuropsychiatric risk genes with a PPXXF motif, and identified the synaptic scaffold protein ankyrin-G.

Genetic studies have shown that synaptic genes are key factors in the pathogenesis of neuropsychiatric disorders [19, 20]. Among these, common variants at the *ANK3* gene locus are one of the most strongly associated risk factors for BD in genome-wide association studies (GWAS) [21, 22]. The isoforms of 190/270/480 kDa of ankyrin-G express in the brain [23], and the largest 270/480 kDa isoforms have well-characterized roles at the axon initial segment (AIS) and nodes of Ranvier [24]; however, the role of the 190 kDa isoform has been mostly studied in the dendritic spines [25–28]. Even though the major isoforms of ankyrin-G share four conserved domains [29], brain-specific expressing exons generate unique properties of ankyrin-G isoforms [30]. Intriguingly, allelic association tests supported that single nucleotide polymorphisms (SNPs) into ankyrin-G 190 specific exons were associated with SZ and BD [31–33]. However, the mechanism through which ankyrin-G 190 regulates these processes is elusive, largely due to the lack of information on its protein interactome and the signaling networks regulating ankyrin-G in spines.

Here, we identified a PPXXF domain present in ankyrin-G 190, which is recognized by Homer1, and confirmed the interaction between ankyrin-G and Homer1 by immunoprecipitation and Proximity Ligation Assay (PLA). Using super-resolution microscopy, we find that the interaction of ankyrin-G and Homer1 occurred in distinct nanodomain structures within the spine head and that the number of both ankyrin-G and Homer1b/c puncta correlates with the head size of mushroom spines. This interaction was reduced by disrupting the PPXXF motif through single amino acid modifications. We have shown that ankyrin-G and Homer1, both neuropsychiatric disorder factors, interact in a complex to regulate spine morphology and to modulate the steady-state levels of ankyrin-G in the spine head. In addition, a comparison of protein abundances in the crude plasma membrane (P2) fraction from the cortex of *HOMER1* KO and wild-type (WT) mice demonstrated global proteomic alterations, characterized by extensive upregulation of synaptic proteins. Taken together, these findings demonstrate that the ankyrin-G 190 and Homer1 interaction regulates spine stability, revealing novel mechanisms underlying spine structure that may be relevant to neuropsychiatric disease.

## Materials and methods

### Plasmids and antibodies

3XHA-ankyrin-G (EX-Mm25668-M06) was purchased from GeneCopoeia. Three domain fragments of ankyrin-G (amino acids 1–807, 808–1475, 1476–1961) were amplified from 3XHA-ankyrin-G. GFP-tagged ankyrin-G was amplified from 3XHA-ankyrin-G, and subcloned into pEGFP-N2 (#6801-1; Clontech) using the Infusion ligation independent cloning kit (Clontech). The detailed information was described previously [26, 27]. 3XHA-ankyrin-G P1606L, F1609R and GFP-ankyrin-G F1609R were generated using QuickChange Site-Directed Mutagenesis Kit (Agilent) as per the manufacturer’s instructions. Homer1c was amplified from pmEmerald-Homer1c (Addgene; #54120) and subcloned into pEZ-M12 to generate 3XFlag-Homer1c using the Infusion ligation independent cloning kit (Clontech). The following primary antibodies were used: mouse ankyrin-G (Neuromab; N106/36), rabbit ankyrin-G (SantaCruz; sc-28561), rabbit Homer1b/c (Antibodies-online; ABIN-1742339), mouse Homer1b/c (SantaCruz; sc-25271), mouse Flag (Sigma; F1804), mouse HA (Abcam; ab130275), rabbit Flag (Sigma; F7425), rabbit HA (Enzo; ADI-MSA-106), mouse PSD-95 (NeuroMab; K28/43), mouse Na K ATPase α1 (Novus; NB-300-146), mouse β-actin (Sigma; A2228), mouse Flag-488 (Abcam; ab117505), rabbit HA-488 (Abcam; ab117505), rabbit HA-568 (Synaptic System, 245 003C3), goat mCherry (SICGEN; AB0040), and rabbit GAPDH (Cell signaling; 5174).

### Co-immunoprecipitation assays

Six cortical hemispheres were dissected from 16-week-old mice and were homogenized in cold sucrose buffer (20 mM HEPES pH 7.4, 320 mM sucrose, 5 mM EDTA) with protease inhibitor cocktail (Roche). Homogenates were centrifuged at 3000 × *g* for 20 min at 4 °C to pellet nuclei. The supernatant (S1; cytosol/membranes) was then centrifuged at 38,000 × *g* for 30 min at 4 °C to obtain crude synaptosomes (P2) from pellets and cytosol (S2) from the supernatant. P2 was resuspended in 0.5 ml of tris buffer (per cortex) (20 mM Tris pH 7.4, 150 mM NaCl, 1 mM EGTA with protease inhibitors). We then added 0.5 ml of 2 x Triton buffer (per cortex) (20 mM Tris pH 7.4, 150 mM NaCl, 1 mM EGTA, 2% Triton X-100 with protease inhibitors) to P2 and rotated it for 1 h at 4 °C to solubilize. The samples were centrifuged at 16,000 × *g* for 10 min at 4 °C. Triton-insoluble pellets were solubilized by adding 100 µl of SDS buffer (20 mM Tris pH 7.4, 150 mM NaCl, 1 mM EGTA, 1% SDS with protease inhibitors) and incubating at 37 °C for 20 min. The samples were centrifuged at 16,000 × *g* for 10 min at 4 °C again. The final supernatant contains PSD-enriched synaptosomal fraction. For the immunoprecipitation, PSD-enriched samples were diluted in tris buffer (the final concentration per each antibody: 150 µg/1.0 ml).

For assays of overexpressed ankyrin-G and Homer1c constructs, HEK293T cells (ATCC; CRL-11268) were transfected with 3XHA-ankyrin-G and 3XFlag-Homer1c using PEI transfection reagent (Sigma) in two 100-mm dishes per each construct. The detailed information for immunoprecipitation was described previously [26, 27]. For the western blotting, VeriBlot for IP Detection Reagent (Abcam; ab131366) was used to avoid overlapping the heavy chain bands with 3XFlag-Homer1c. The membrane was blocked with Protein-Free T20 (TBS) Blocking buffer (Pierce; #37571), and primary and secondary antibodies were diluted in the same buffer.

### Neuronal cell culture and transfection

Dissociated primary cortical cultured neurons were prepared from P0 C57BL/6J (The Jackson Laboratory). Brains were dissected in ice-cold Leibowitz’s L-15 media with penicillin/streptomycin, and cortical tissue isolated, digested with 0.25% trypsin-EDTA solution at 37 °C for 10 min, and mechanically dissociated in high-glucose Dulbecco’s Modified Eagle Medium (DMEM) supplemented with 10% FBS, 1.4 mM L-glutamine, and 6.0 g/L glucose. The detailed information was described previously [26, 27]. All procedures were in compliance with the National Institutes of Health standards and were approved by Northwestern University’s Animal Care and Use Committee.

### Confocal microscopy

Immunostained neuronal images were obtained with a Nikon C2+ confocal microscope. Confocal images were taken using the 63x oil-immersion objective (NA = 1.4) as z-series of 8–10 images, taken at 0.4 μm intervals, with 1024 × 1024 pixel resolution. Detector gain and offset were adjusted in the channel of cell fill (mCherry) to include all spines and enhance edge detection.

### Immunocytochemistry

By using 4% paraformaldehyde in PBS, cells were fixed for 10 min at 4 °C. PBS washing for 5 min (three times), fixed neurons were permeabilized and blocked in PBS containing 0.3% Triton-X-100 and 1% bovine serum albumin (BSA) for 1 h at room temperature. Primary antibodies were added in PBS containing 0.3% Triton-X-100 and 1% BSA overnight at 4 °C, followed by 10 min washes in PBS (three times). Secondary antibodies were incubated for 1 h at room temp in 0.3% Triton-X-100 and 1% BSA in PBS. PBS washing for 5 min (three times) was performed before coverslips were mounted using Fluorescent Mounting Medium (Invitrogen).

### SIM imaging and analysis

With EM gain and no binning, the acquisition was set to 10 MHz, 14 bits. The EM gain multiplier restrained below 300, and auto exposure was set between 100–300 ms and. Within the first quarter of the scale (<4000), the laser power was adjusted to keep LUTs. Imaging and reconstruction parameters were determined with the assistance of the expertise in the Center for Advanced Microscopy at Northwestern University. The single-plane where the spine head was in focus, based on the cell fill, was chosen for analysis. Each spine head was outlined using the Image J software manually in the channel of the cell fill to detect the area. Within the spine head, ankyrin-G and Homer1b/c puncta were outlined manually, and the size was recorded. A 100 μm dendritic region was selected, and puncta counts were made; puncta smaller than 0.006 μm^2^ were excluded from the analysis. To present more informative images, z-series images were reconstituted by Imaris v9.1.2 (OXFORD instruments).

### Proximity ligation assay (PLA)

Cortical neurons were transfected with pmCherry-C1 (Clontech, #632524) and 3XHA-ankyrin-G at DIV21. The transfected cells for three days were fixed with 4% paraformaldehyde in PBS for 10 min at 4 °C. For the negative control, PLA experimented on only mCherry transfected cultured neurons. With 3XFlag-Homer1c and 3XHA-ankyrin-G, COS7 cells were transfected using lipofectamine 2000 reagent. The transfected cells were fixed at 24 h post-transfection. Rabbit 568 conjugated-HA and mouse 488 conjugated-Flag antibodies were treated to confirm the transfection of constructs into the cells. Duolink™ In Situ Detection Reagents FarRed (Sigma, DUO92013) was used to detect the PLA signal.

### Fluorescent recovery after photobleaching (FRAP)

Cortical neurons for FRAP experiments were grown on 18 × 18 mm coverslip and transfected with GFP alone, GFP-ankyrin-G WT, or GFP-ankyrin-G F1609R. Coverslip was placed in a round bath chamber (Warner Instruments; RC-41LP) and neurons were imaged with a C2 confocal microscope (Nikon) in a CO2 incubator (TOKAI HIT; UNIV-D35). The size of spine heads in GFP-ankyrin-GF1609R transfected neurons generally was downsized, and we decided to match the spine head size from each group. Images were captured with an EMCCD camera of 512 × 512 pixel resolution every 10 s for 200 s. 100% laser power pulses of 1 ms (2 iterations) were used to bleach GFP clusters. Fluorescence intensity of bleached GFP clusters was measured and normalized to the fluorescence of an unbleached region to correct for photobleaching. These values were normalized to the mean of the 5 frames prior to bleaching, and the lowest value in the dataset was subtracted from all values. Recovery data points were then fitted to a one-phase association exponential in GraphPad Prism8. The mobile fraction was calculated as an average of the plateaued fluorescence level and expressed as a percentage of the pre-bleached level. We determined the mobile fraction by calculating the ratio of fluorescence intensity between the end of the time-lapse recordings (F_end_) and the initial intensity before the bleaching event (F_initial_), corrected by the experimental bleach value (F_0_) and expressed as a percentage.

### Quantitative proteomics and analysis

Homer1 heterozygous mice (The Jackson Laboratory; #023312) were mated with the same heterozygous mice to maintain the colonies. Littermates that five *HOMER1* KO mice and five WT mice (all females) from 4 cohorts were used for the proteomic analysis. The mice were housed with mixed-genotype home cages together until testing. Within an SPF barrier area mice were maintained on a 12:12 h light/dark schedule in an air-conditioned room, under constant conditions of temperature and humidity. Food and tap water (membrane filter purified and autoclaved water) were provided ad libitum. All experiments were performed in accordance with the Institutional Animal Care and Use Committee at Northwestern University.

3-week-old mouse cortex was dissected and homogenized in sucrose buffer (20 mM HEPES pH 7.4, 320 mM sucrose, 5 mM EDTA with Roche protease inhibitors) and centrifuged at 3000 × *g* for 20 min, 4 °C. The collected supernatant was centrifuged further at 16,000 × *g* for 30 min at 4 °C to obtain crude synaptosomes (P2) and collect supernatant for cytosolic proteins (S2). Pellets were resuspended with binding buffer (50 mM Tris-HCl pH 7.5, 1% triton-X-100, 150 mM NaCl, 1 mM EDTA, 1 mM AEBSF with protease inhibitor cocktail and phosphatase inhibitor) and solubilized for 1 h at 4 °C. The detailed information for Tandem Mass Tag-liquid chromatography/mass spectrometry (TMT-LC/MS) was described previously [34].

The normalized average intensity values for proteins from Census were used to calculate the fold change WT versus KO. The values were standardized to the mean of the five WT samples, and the fold change could then be calculated as the mean of the KO standardized values. *P* values were calculated by one-tailed Student’s *t* test. For GO analysis, the list of significantly upregulated or downregulated proteins was queried against all proteins quantified in the dataset using a statistical overrepresentation test of the DAVID Bioinformatics Resources (v6.8). Biological processes which were a subset of a larger group that was also statistically significant defaulted to the more encompassing annotation and its associated *P*-value. For STRING analysis, 82 regulated proteins in postsynaptic density (PSD) from the cortex of *HOMER1* KO mice were loaded into multiple proteins option of STRING (v11.0) and analyzed setting with all interaction sources and medium confidence (0.400) score. The data were exported and the list of interactors was sorted PSD proteins and ASD risk factors out again. To generate a representative interacting network in PSD, 63 of sorting proteins were analyzed by Cytoscape (v3.7.1).

### Blinding and statistical analysis

Data from spine morphology and dynamics, protein localization, and PLA analysis in pyramidal neurons (in vitro) were analyzed under blinded conditions (coverslip identity hidden and raw data pooled during quantification). Fractionation, immunoprecipitation, western blotting, and MS experiments were not blinded. Cells of visibly poor health (i.e., blebs, broken dendrites, poor expression, etc.) were excluded from quantification. No animals were excluded from the analysis, and no method of animal randomization was employed. Sample sizes were on average between 9 and 13 cells for endogenous analysis and between 16 and 18 cells for overexpression analysis to account for cellular diversity. Five animals per genotype were used for western blotting and MS experiments, which was sufficient for statistical significance.

GraphPad Prism 8 was used for all statistical tests. Two-sample comparisons were performed using two-tailed unpaired Student’s *t* test or two-tailed non-parametric Spearman correlation, and multiple comparisons were made using one-way ANOVA followed by non-parametric statistical analysis or two-way ANOVA followed by a Bonferroni test. Statistical details are given in each figure legend. Bar graphs are displayed as mean ± SEM. *P* values < 0.05 were considered statistically significant.

## Results

### Proteins containing the Homer1-binding PPXXF motif are abundant within postsynaptic and disease networks

**Fig. 1:**
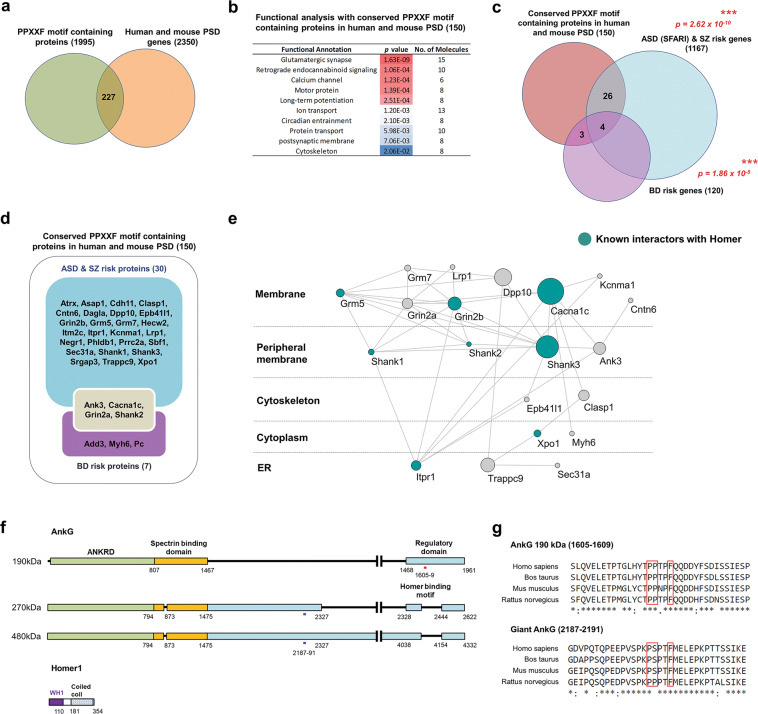
Multiple postsynaptic psychiatric risk factors include a Homer1-binding motif. **a** Diagram of PPXXF motif-containing proteins in PSD. **b** Functional analysis of conserved (in human, bovine, mouse, and rat) PPXXF motif-containing proteins in PSD. **c** Enrichment of BD, ASD, and SZ risk factors (identified through GWAS, SFARI gene archive and de novo studies, respectively) among PPXXF motif-containing proteins in the combined human and mouse PSD. ***p < 0.001; hypergeometry test. **d** Diagram of PPXXF motif-containing proteins in the PSD is encoded by psychiatric risk genes. **e** Protein interaction network generated from the list of PPXXF motif-containing PSD proteins is encoded by psychiatric risk genes in **d**. The representative majority of a subnetwork is visualized and annotated by Cytoscape. The size of node indicates betweenness centrality. Known interactors with Homer1/2/3 from BioGrid and text mining were colored emerald. Edges indicate known and predicted protein-protein interactions, including experimental data from the STRING database. **f** Schematic representation of the Homer1 recognition motifs in ankyrin-G (190, 270, and 480 kDa) isoforms. **g** Amino acid sequence alignment of human, bovine, mouse, and rat ankyrin-G regions containing the Homer-binding motif PPXXF. Sequence alignments were performed by Clustal Omega. SFARI: Simons Foundation Autism Research Initiative.

The PPXXF motif present in some proteins known to interact with Homer’s EVH1 ligand motif is important for protein-protein interactions [16]. To determine whether Homer1-binding proteins are enriched in the PSD, we retrieved all PPXXF-containing proteins identified in UniProt (*n* = 1995) with the aid of the MOTIF search and performed a gene set analysis with genes encoded by PSD proteins [35, 36]. We found a subset of genes encoding PPXXF-containing proteins that have been reported to be localized to PSDs (*n* = 227, 11.4%, Fig. 1a). To evaluate the conservation of the PPXXF motif across species within this PSD subset, we examined the sequence of the motif in human, bovine, mouse, and rat (Supplementary Table 1), and 150 proteins were confirmed to have a conserved PPXXF motif. Gene ontology (GO) analysis revealed this PPXXF-PSD gene set was highly enriched for specific biological processes including proteins regulating glutamatergic synapses, retrograde endocannabinoid signaling and long-term potentiation (Fig. 1b). To determine whether PPXXF motif-containing proteins in the PSD a role in neuropsychiatric disease, we again performed gene set analysis with bipolar disorder, autism and schizophrenia-associated gene sets. We found a significant enrichment of PPXXF motif-containing proteins in genome-wide significant loci from BD GWAS (7/120) and de novo variants associated with ASD and SZ (30/1167). The enrichment of PSD-PPXXF motif-containing proteins within neuropsychiatric disease risk factors suggests proteins containing this motif may play a role in pathophysiology (Fig. 1c, d). To determine the functional relationship between these risk genes we seeded a protein-protein interaction (PPI) network with the 33 proteins associated with neuropsychiatric disease. We found that the majority of PPXXF motif-containing psychiatric risk factors in PSDs are physically connected (20 nodes, 43 edges) (Fig. 1e; Supplementary Fig. 1). By performing network analysis, we found that Cacna1c and Shank3 were the central hubs and ankyrin-G is one of the most topologically important nodes in the peripheral membrane as an unknown interactor of Homer.

We found two PPXXF motifs in ankyrin-G (Fig. 1f, g). One was within the “regulatory domain”, present in the 190 kDa isoform, but not 270 and 480 kDa isoforms (ankyrin-G 190: 1605–1609). A second motif was within a region present in the giant ankyrin-G forms, including 270 and 480 kDa forms, but not the 190 kDa isoform (ankyrin-G 270 and 480: 2187–2191).

### Ankyrin-G 190 and Homer1b/c interact in nanodomains within dendrites and spines

**Fig. 2:**
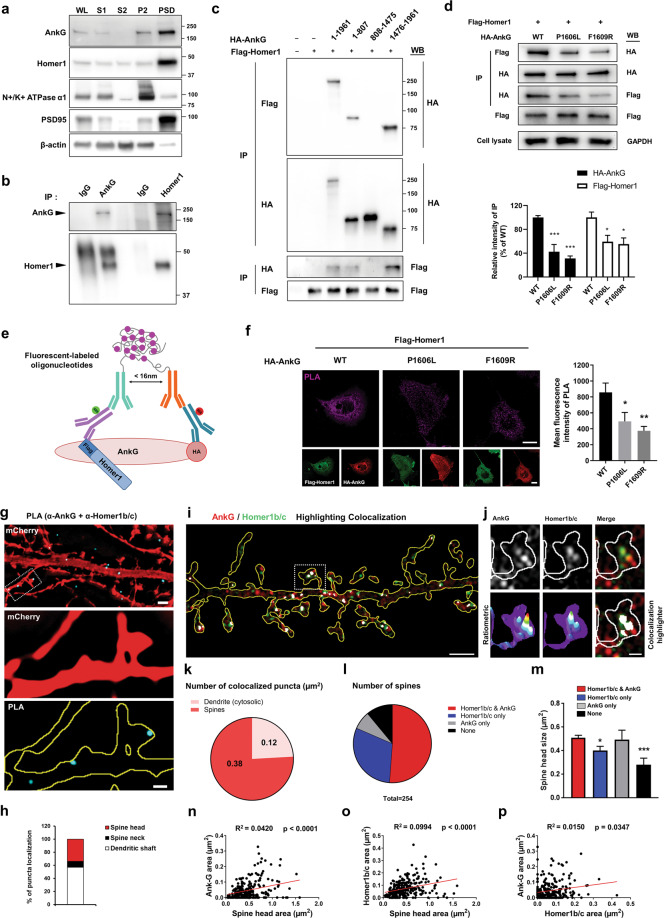
Ankyrin-G 190 and Homer1b/c interaction in nanodomains within dendrites and spines. **a** Western blot of subcellular fractionation from 16-week-old mouse cortex is probed with ankyrin-G, Homer1b/c, N+/K+ ATPase α1 (as a plasma membrane marker), PSD-95 (as a postsynaptic marker), and β-actin antibodies. Each lane was loaded with 10 µg of each sample. WL whole cell lysate, S1 cytosol/membranes, S2 cytosol, P2 crude synaptosomes, PSD PSD-enriched synaptosomal fraction. **b** Co-immunoprecipitation experiments with anti-ankyrin-G or anti-Homer1b/c from the PSD-enriched synaptosomal fraction. IgG control IgG, IP immunoprecipitation. **c** Binding of ankyrin-G and its truncation mutants to Homer1c. The top panel shows the immunoprecipitated full-length ankyrin-G or the truncated versions of ankyrin-G co-expressed with Homer1c. **d** Effects of point mutations in the PPXXF Homer-binding motif of ankyrin-G (P1606L and F1609R) on the interaction with Homer1c. The HEK293T cell lysate was analyzed by immunoblotting with HA or Flag antibody. *n* = 4 per each group. *F*(2, 9) = 24.10; *F*(2, 9) = 6.17; *p < 0.05, ***p < 0.001; followed by one-way ANOVA followed by a Bonferroni test. Data are represented as mean ± SEM. **e** Schematic representation for the in situ proximity ligation assay (PLA). **f** Confocal images for detection of the interaction between HA-ankyrin-G wild-type (WT) or point mutants (red) and Flag-Homer1c (green) with PLA (magenta) in COS7 cells. Scale bar, 20 µm (top and bottom). Bar graph of the PLA signal with the mutants of HA-ankyrin-G and Flag-Homer1c. *n* = 13 per each group. *F*(2, 36) = 6.48; *p < 0.05, **p < 0^.^01; one-way ANOVA followed by a Bonferroni test. Data are represented as mean ± SEM. **g** SIM image of a mCherry-expressing neuron to detect the interaction between ankyrin-G and Homer1b/c by PLA (cyan). Scale bar, 2 µm (top panel). Zoomed images were shown from the boxed area in the top panel. Scale bar, 0.5 µm (middle and botton panels). **h** Bar graph showing the PLA puncta ratio in spines *versus* dendrites, *n* = 9 cells. **i** SIM image of mCherry-expressing neurons immunostained for anti-ankyrin-G (red) and anti-Homer1b/c (green). Scale bar, 2 µm. Colocalization is shown in white by ‘colocalization highlighter’ in ImageJ. **j** High-resolution image of boxed spine in **i**. Bottom panels: ratiometric images and colocalization (white). Scale bar, 0.5 µm. **k** Pie chart showing the highlighted puncta ratio of spines *versus* dendrites per spine area. **l** Pie chart of expression patterns of ankyrin-G and Homer1b/c in spines. **m** Spine head size from the chart (**k**) was analyzed with a bar graph. *F*(3, 250) = 5.77; *p < 0.05, ***p < 0.001; one-way ANOVA followed by a Bonferroni test. Data are represented as mean ± SEM. Correlation plot of the size of anti-ankyrin-G (**n**) or anti-Homer1b/c (**o**) nanodomains versus spine head area, and the size of anti-ankyrin-G versus anti-Homer1b/c nanodomains (**p**). *n* = 13 cells. Head area: 0.452 ± 0.017 µm^2^; anti-ankyrin-G nanodomain area: 0.032 ± 0.002 µm^2^; anti-Homer1b/c area: 0.056 ± 0.003 µm^2^.

Because here we are investigating postsynaptic interactions within spines, and both Homer1 and ankyrin-G 190 are enriched in spines, we decided to focus on the interaction of ankyrin-G 190 with Homer1, in postsynaptic regions. In order to confirm the spatial co-distribution of ankyrin-G and Homer1b/c and investigate the interaction of these proteins in vivo, we performed subcellular fractionation of mouse cortex by ultracentrifugation and detergent solubilization. Both ankyrin-G 190 and Homer1b/c were highly expressed in a PSD-enriched fraction (Fig. 2a). We then used this PSD-enriched fraction for coimmunoprecipitation experiments and showed that ankyrin-G 190, but not 270, robustly coimmunoprecipitated with Homer1b/c from this fraction (Fig. 2b). To determine the interaction sites between ankyrin-G and Homer1c, we conducted immunoprecipitation experiments with three HA-tagged truncated ankyrin-Gs (amino acids 1–807, 808–1475, 1476–1961) and Flag-tagged Homer1c. As predicted, we found that Homer1c robustly co-immunoprecipitated with the regulatory domain (1476–1961) of ankyrin-G 190 (Fig. 2c). However, we detected a weaker interaction with the ankyrin-repeat domain of ankyrin-G as well (1–807) (Supplementary Fig. 2). One previous study showed that a point mutation (proline to leucine or phenylalanine to arginine) of the Homer-binding motif impaired interaction with diacylglycerol lipase type-α [37]. To determine the role of the ankyrin-G PPXXF motif in the Homer1c interaction, PPXXF mutants (Pro1606Leu and Phe1609Arg) of ankyrin-G were produced and co-immunoprecipitated after overexpression in HEK293T cells. Point mutations in the PPXXF Homer1-binding motif of ankyrin-G resulted in the attenuation of Homer1c interaction (Fig. 2d). To test the interaction of ankyrin-G and Homer1b/c, in situ PLA was employed. This technique labels interacting proteins that fall within a defined, 16-nanometer distance (as calculated from the number of nucleotides in the attached DNA arms [38]), a strong indicator of direct protein-protein interaction or complex formation (Fig. 2e). Despite robust interaction with the native ankyrin-G, the PLA signal was significantly decreased in cells expressing Homer1 and PPXXF mutated ankyrin-G (Fig. 2f). Colocalization and the subcellular compartments of Homer1b/c and ankyrin-G complexes were assessed using PLA in primary cortical neurons, confirming an interaction within spine heads (Fig. 2g, h). Taken together, these data indicate that ankyrin-G 190’s binding to Homer1 through its PPXXF motif strongly modulates their interaction, while an additional binding site in the ankyrin-repeat region, present in all ankyrin-G isoforms, may also affect their interaction.

Because both ankyrin-G and Homer1b/c have been detected in the PSD by proteomics, we hypothesized that their interaction might have a role in regulating spine morphology [36]. To understand how the localization of ankyrin-G and Homer1b/c nanodomains correlates with spine morphology, we imaged mouse primary cortical neurons stained for endogenous ankyrin-G and Homer1b/c by SIM microscopy and analyzed their subcellular distribution (Fig. 2i, j). Manders’ colocalization coefficients showed that ankyrin-G colocalizes with Homer1b/c in the dendritic shaft and spine heads. Interestingly, ankyrin-G/Homer1b/c overlapping nanodomains were largely localized in the spine head (Fig. 2k). Ankyrin-G or Homer1b/c puncta were observed in 58.7 and 81.5% of spines, respectively. Both ankyrin-G and Homer1b/c were observed in 51.2% of spines (Fig. 2l). Spine heads expressing ankyrin-G are significantly larger than spine heads without ankyrin-G (Fig. 2m, n), as previously reported [28]. The presence of Homer1b/c in spine heads was also significantly correlated to larger spine head size (Fig. 2o). Homer1b/c clusters had a mean area of 0.056 ± 0.003 µm^2^, and this area correlated to the size of ankyrin-G nanodomains and spine head area (Fig. 2p). Taken together, the size of Homer1b/c clusters in spine head is positively correlated to the number and size of ankyrin-G nanodomains, and spine head size.

### PPXXF-mediated interaction of ankyrin-G and Homer1b/c regulates spine morphogenesis

**Fig. 3:**
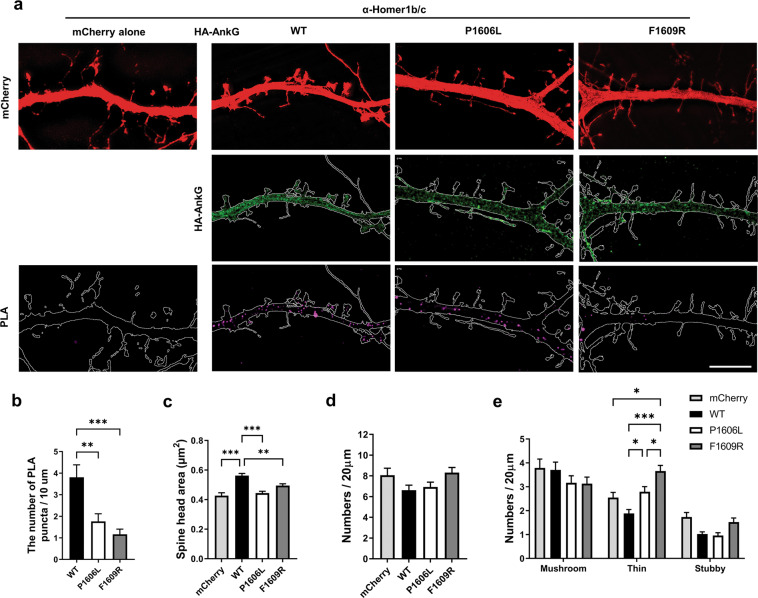
Mutation of the PPXXF binding motif in ankyrin-G 190 attenuates its interaction with Homer1b/c in dendrites. **a** SIM images to detect the interaction between HA-ankyrin-G WT or mutants (P1606L or F1609R) and anti-Homer1b/c by in situ PLA. The PLA was conducted after transfection of HA-ankyrin-G WT or mutant constructs into the cultured mouse cortical neurons. Negative control was transfected with only mCherry construct. Scale bar, 2 µm. **b** The number of PLA puncta per 10 µm from HA-ankryn-G WT, P1606L, and F1609R transfected neurons. PLA was performed with anti-Homer1b/c and anti-HA antibodies. *F*(2, 123) = 11.75. **c** Spine head size from mushroom spines was analyzed from only mCherry or HA-ankryn-G WT, P1606L, and F1609R transfected neurons with mCherry. *F*(3, 1445) = 18.62. **d** Total numbers of all spines were counted. *F*(3, 141) = 3.64. **e** Mushroom, thin, and stubby types of spine morphology were analyzed. F(6, 423) = 5.59. *n* = 13 (only mCherry), 17 (WT), 19 (P1606L), 21 (F1609R) cells. *p < 0.05; **p < 0.01; ***p < 0.001; one-way ANOVA followed by a Bonferroni test for **b**–**d** and two-way ANOVA followed by a Bonferroni test for **e**. Data are represented as mean ± SEM.

To understand interaction patterns of Homer1b/c and ankyrin-G in neurons, we expressed exogenous WT HA-ankyrin-G 190, or its mutants, P1606L, or F1609R, in mouse cultured cortical neurons. Comparing the effect of moderate exogenous expression of WT *versus* mutant forms of ankyrin-G 190 allows us to specifically test the effect of increased abundance of ankyrin-G 190 in spines, as shown to be induced by neuronal activity or by mood stabilizers, without altering the expression of all other isoforms that may cause indirect effects on spines. The PLA method allows interacting proteins to be quantitatively and spatially visualized. SIM imaging of PLA signal revealed decreased Homer1 interaction with ankyrin-G^P1606L^ and ankyrin-G^F1609R^, as compared with WT ankyrin-G 190, in dendrites and spines (Fig. 3a, b). As shown before, exogenous expression of WT ankyrin-G 190 increased the spine head size. However, expression of the Homer1 interaction-deficient mutants of ankyrin-G 190 failed to induce a similar increase in spine head size, coincident with a decrease in PLA positive signal in spines (Fig. 3c; Supplementary Fig. 3). Interestingly, ankyrin-G^F1609R^ overexpression also increased the proportion of thin spines (Fig. 3d, e). Total spine numbers were not altered in any of these conditions (Fig. 3d).

### The PPXXF motif in ankyrin-G 190 affects its mobile fraction in spines and spine head remodeling

**Fig. 4:**
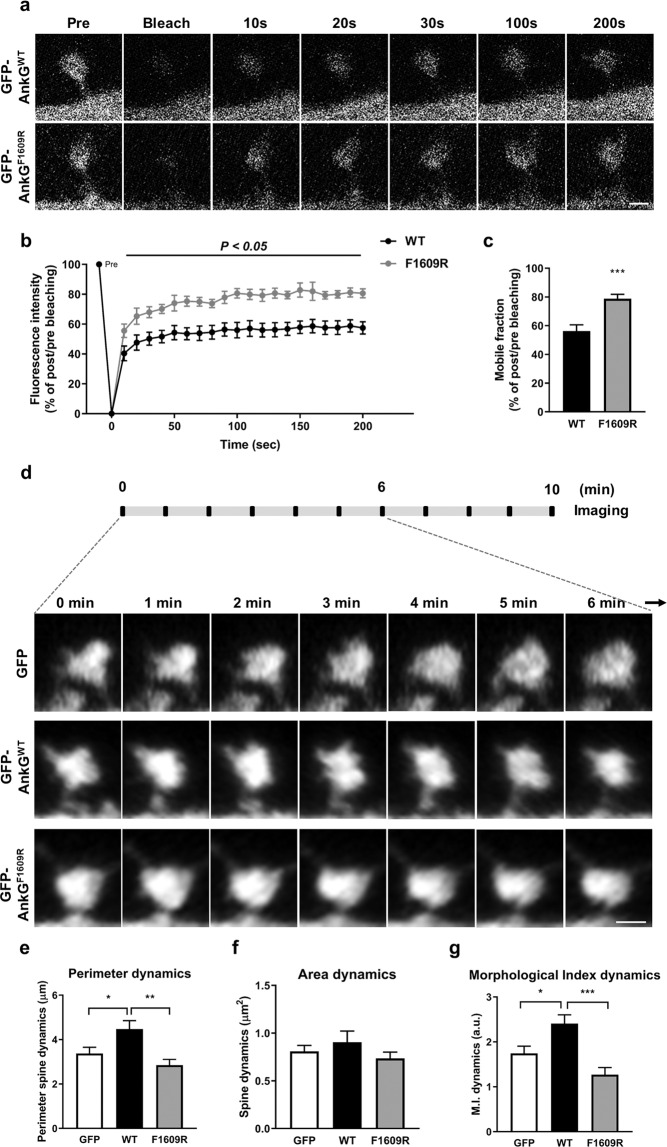
Mutation of the PPXXF binding motif in ankyrin-G 190 increases its mobile fraction in spines and reduces spine head dynamics. **a** Representative time-lapse of fluorescence recovery in a FRAP experiment in neurons overexpressing GFP-ankyrin-G 190 WT or F1609R. Scale bar, 1 µm. **b** Quantification of GFP-ankyrin-G or mutant fluorescence intensity in each spine every 10 s for 200 s. *F*(20, 126) = 19.32; two-way ANOVA followed by a Bonferroni test. Data are represented as mean ± SEM. **c** The graph of the mobile fraction (% of post per pre bleaching at 200 s) in GFP-ankyrin-G^WT^ and GFP-ankyrin-G^F1609R^-overexpressing neurons (*n* = 7 cells per each group, 3–5 spines from a neuron on three independent experiments). t(12) = 4.24; ***p < 0.001; two-tailed Student’s *t*test was performed. **d** Time-lapse imaging of spine head dynamics. Neurons were co-transfected with tdTomato together with GFP, GFP-ankyrin-G^WT^ or GFP-ankyrin-G^F1069R^ and imaged for 10 min. Seven representative images from 0 to 6 min taken at different time points were shown. **e** Analysis of perimeter dynamics from spine head. *F*(2, 48) = 7.28. **f** Quantification of area dynamics from spine head area. *F*(2, 48) = 1.05. **g** Spine morphological index (M.I. = P^2^/4πA) dynamics in 10 min from spines. *F*(2, 48) = 11.22. GFP: *n* = 17 spines, GFP-ankyrin-G^WT^: *n* = 16 spines, GFP-ankyrin-G^F1069R^: *n* = 18 spines; all spines were measured from 9 neurons per each group on four independent experiments. *p < 0.05; **p < 0.01; ***p < 0.001; one-way ANOVA followed by a Bonferroni test. Data are represented as mean ± SEM. See also Movie 1.

Our previous experiments demonstrated that HA-ankyrin-G^F1609R^ significantly reduced the interaction with Homer1 and impaired the ability of ankyrin-G 190 to increase spine head size. We thus wanted to determine the impact of impairing ankyrin-G 190’s interaction with Homer1 on its mobile fraction within spines. To this end, we performed fluorescence recovery after photobleaching (FRAP) time-lapse imaging of GFP-ankyrin-G^WT^ and GFP-ankyrin-G^F1609R^ in dendritic spines of primary cortical neurons. Upon recovery after photobleaching for 200 s, the mobile fraction of GFP-ankyrin-G^F1609R^ was 22.44% higher than that of GFP-ankyrin-G^WT^, indicating a higher mobile fraction for the Homer1 interaction-deficient mutant (Fig. 4a–c). This indicates that Homer1 interaction-deficient ankyrin-G 190 is less strongly associated with PSD or other stable components in spines that wild type ankyrin-G 190, likely due to its weak interactions with Homer1.

To assess the role of its interaction with Homer1 in ankyrin-G 190’s ability to modulate spine head remodeling, we performed live confocal imaging of spines with mature morphologies, defined by head sizes above 0.6 µm^2^. First, to visualize morphological remodeling of spine heads, we acquired stacks of images every minute for 10 min and generated 2D projections of spines for each time point, and used them to measure parameters reflecting spine head remodeling. While overexpression of GFP-ankyrin-G^WT^ caused an increase in perimeter dynamics, defined as the change in spine head perimeter during imaging, GFP-ankyrin-G^F1609R^ failed to do so (Fig. 4e, f; Movie 1). On the other hand, the area dynamics, defined as the change in the spine area, showed no significant difference between conditions (Fig. 4f). Finally, overexpression of GFP-ankyrin-G^WT^, but not GFP-ankyrin-G^F1609R^, caused an increase in morphological index dynamics, defined at the change in morphological index (perimeter^2^/4πArea), which is an indicator of spine shape complexity (Fig. 4g). These data indicate that the PPXXF motif is important for ankyrin-G 190’s ability to form a stable pool in spines and to enhance spine head remodeling. We hypothesize that Homer1b/c acts to stabilize steady-state ankyrin-G pools in the spine head ultimately regulating spine head morphology.

### The PSD proteome is reshaped in *HOMER1* KO mouse cortical synaptosomal membranes

**Fig. 5:**
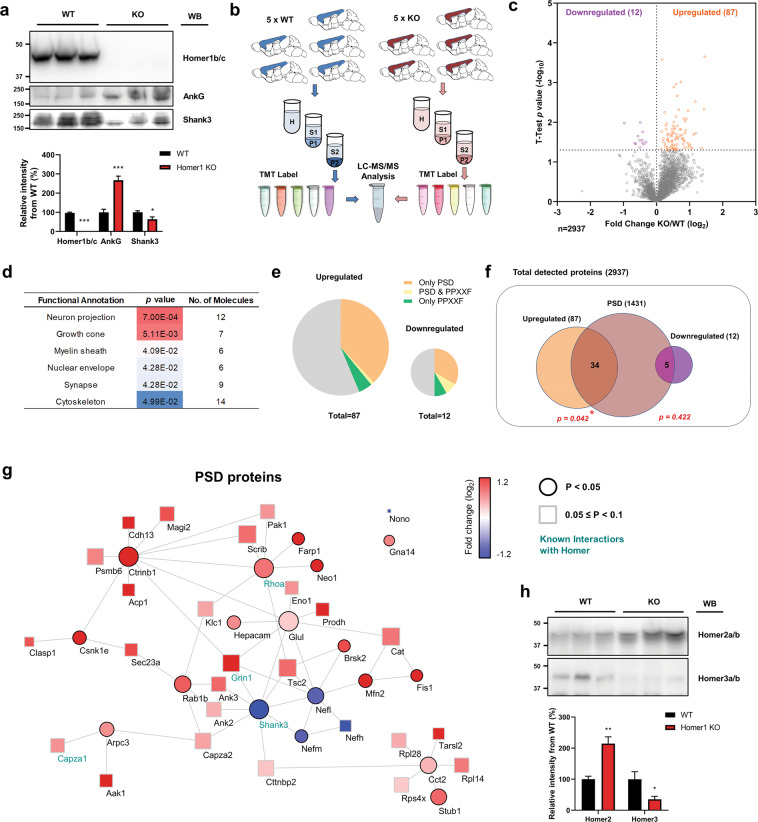
The proteome is remodeled in the cortex of *HOMER1* KO mice. **a** Levels of Homer1b/c (t(8) = 25.45), ankyrin-G (t(8) = 6.32), and Shank3 (t(8) = 2.54) proteins in P2 (crude synaptosomes) samples from the cortex of 3-week-old WT or *HOMER1* KO mice. *n* = 5 per each group. *p < 0.05; ***p < 0.001; two-tailed Student’s *t* test was performed. Data are represented as mean ± SEM. **b** Scheme of experimental work-flow of the proteomic analysis. **c** Volcano plot showing the fold change of individual protein levels *versus* significance between WT and *HOMER1* KO mice. Significantly upregulated proteins are in orange (*p* < 0.05), significantly downregulated proteins are in purple (*p* < 0.05), and all other proteins are in gray. **d** Gene ontology (GO) analysis of statistically overrepresented biological processes among the significantly differentially regulated proteins in *HOMER1* KO mice. The analysis was performed from data in **c** by DAVID. **e** Pie charts showing the presence in the PSD, inclusion of a PPXXF motif, or both, among the proteins significantly upregulated (left) and significantly downregulated (right) in *HOMER1* KO *versus* WT mice. Gray indicates proteins that do belong to any of the categories. **f** Diagram showing the number of either upregulated or downregulated proteins presents in the PSD. Significance was tested by hypergeometric tests. **g** Cytoscape analysis of PSD proteins with altered levels coded by fold and significance of change. Circular nodes indicate *p* < 0.05 and rectangular nodes indicate 0.05 ≤ *p* < 0.1. Edges indicate predicted protein-protein interaction, including experimental data from the STRING database. The size of nodes indicates betweenness centrality. Previously known Homer1/2/3 interactors labeled in emerald. **h** Levels of Homer2a/b (t(8) = 4.80) and Homer3a/b (t(8) = 2.49) proteins in P2 samples from the cortex of 3-week-old WT or *HOMER1* KO mice. *n* = 5 per each group. *p < 0.05; **p < 0.01; two-tailed Student’s *t* test was performed. Data are represented as mean ± SEM.

Ankyrin-G and Homer1b/c are reported to be enriched in the frontal cortex and hippocampus. Western blotting of Homer1b/c protein levels in the mouse cortex at different developmental time points revealed that Homer1b/c expression increases throughout postnatal development (Supplementary Fig. 4). Homer1b/c potentially forms an assembly platform and supports a structural framework for PSD proteins [18]. Our results show that the PPXXF motif in ankyrin-G is critical to regulating the ankyrin-G dynamics in the spine heads (Fig. 4). We, therefore, hypothesized that the absence of Homer1 could affect the abundances of proteins in this specific cellular compartment. To assess the levels of ankyrin-G in P2 crude synaptosomal fractions in the presence *vs*. absence of Homer1, we assessed ankyrin-G by western blotting of P2 from WT and *HOMER1* KO mice. Unexpectedly, we found that ankyrin-G was more abundant in the *HOMER1* KO than in WT P2 fractions (Fig. 5a). In order to compare the abundance of synaptosomal membrane proteins in *HOMER1* KO and WT mice, we performed TMT-LC/MS with P2 fractions (Fig. 5b). Differentially expressed proteins were quantified by comparing the normalized average reporter ion intensities of peptides among the biological replicates from *HOMER1* KO and WT mice. Proteome quantification was robust, and we were able to quantify 2937 proteins in both groups (Supplementary Table S2). 99 proteins showed significantly different abundance between *HOMER1* KO and WT (*p* < 0.05). Surprisingly, most (87) of the 99 proteins increased in abundance in P2 fraction of *HOMER1* KO mice, and only 12 were reduced compared to WT mice (Fig. 5c). Organizing the significantly altered proteins by functional assignment, we found that the significantly altered proteins were involved in neuron projection, growth cone, myelin sheath, nuclear envelope, synapse, and cytoskeleton in the significantly enriched categories (Fig. 5d). In order to determine whether significantly altered proteins are enriched in PPXXF-containing proteins and PSDs, we retrieved all PPXXF-containing proteins and PSD proteins (Fig. 5e). We found a significant enrichment of PSD proteins (*n* = 39) in upregulated proteins (Fig. 5f); however, PPXXF-containing proteins were not significant within either the upregulated or downregulated proteins (Supplementary Fig. 5a).To understand how the absence of Homer1 reshapes the protein network within the PSD, we seeded a network with the regulated proteins in PSD. The cutoff for regulated expression was expanded to *p* < 0.1 allowing the inclusion of many more known Homer interactors and facilitating interrogation of the PSD-PPI network within *HOMER1* KO mice. Interestingly, Shank3 and neurofilament proteins Nefh, Nefl, Nefm were nodes in a downregulated PPI subnetwork, with Shank3 having the highest centrality. Conversely, the downregulated subnetwork was much broader, with multiple nodes including RhoA, Ctnnb1, Grin1, Tsc2, and Ank3 (0.05 ≤ *p* < 0.1) which were upregulated (Fig. 5g; Supplementary Fig. 6a). It is important to note that a significant increase of Homer2a/b abundances was observed in P2 samples, perhaps compensating for the decrease of Shank3 (Fig. 5h; Supplementary Fig. 6c). Taken together, our data suggest that Homer1 is important for the maintenance of synaptic PPI networks, and its loss results primarily in the upregulation of synaptic abundance of postsynaptic and neurodevelopmental disorder-related proteins.

## Discussion

The PPXXF motif is known to mediate the interaction of Homer’s EVH1 ligand motif with other proteins [16]. However, the global functions of these interactions are not known. Using computational approaches, we identified global roles for proteins containing the Homer1-interacting PPXXF motif within the postsynaptic compartment. PPXXF motif-containing proteins as a group are likely to be important in glutamatergic transmission and long-term potentiation, retrograde signaling, calcium channel function, protein transport, as well as in neurodevelopmental and psychiatric disease. Among the 150 PPXXF motif-containing proteins in the PSD, ankyrin-G (*ANK3)*, NR2A (*GRIN2A*), Shank2 (*SHANK2*), and Ca_V_1.2 (*CACNA1C*) are both BD and ASD risk factors. The enrichment of PSD-PPXXF motif-containing proteins in neuropsychiatric disease risk factors suggests that proteins containing this motif may play a role in pathophysiological processes downstream of genetic risk factors. We found that the majority of PPXXF motif-containing psychiatric risk factors in PSDs are physically connected, and central hubs of this network are Cacna1c, Shank3, Grin2b, and ankyrin-G. While the interaction between Homer1 and Shank2 or Ca_V_1.2 has already been reported [39, 40], future investigations of the interactions predicted by the presence of this motif is likely to reveal important biological and pathogenic pathways.

Ankyrin-G and Homer1 have emerged as candidate genes in multiple psychiatric disorders including BD, SZ, and ASDs [11–13, 41, 42]. These disorders share common synaptic pathologies, such as alterations in synaptic number and size. However, little is known about the underlying molecular mechanisms. Our findings demonstrate ankyrin-G and Homer1, both neuropsychiatric disorder genes, interact in a complex to regulate spine morphology. This interaction reveals a potential mechanism through which Homer1 may regulate the localization and function of interacting proteins within the synapse. We identified two Homer recognition sites within ankyrin-G and showed a robust interaction between the 190 kDa isoform of ankyrin-G and Homer1b/c. This interaction was partially mediated by the Homer1 recognition motif (PPXXF) within the regulatory domain of ankyrin-G 190. It is possible that Homer1 also interacts with the giant ankyrin forms in other cellular locations and may be involved in additional physiological processes at those sites. These possibilities remain to be investigated in the future. We validated this interaction by in vivo and in vitro immunoprecipitation, PLA, and confirmed the interaction domain by site-directed mutagenesis. Because of the limited resolution of confocal microscopy, we utilized SIM to analyze the precise postsynaptic localization of ankyrin-G and Homer1b/c. High-resolution SIM allowed us to visualize and quantify the localization of ankyrin-G and Homer1b/c nanodomains in spine heads and revealed correlations between the presence of nanodomains and spine head size. While ankyrin-G colocalized with Homer1b/c in both the dendritic shaft and spine heads, colocalization was most extensive in spine heads. The presence of ankyrin-G alone, or ankyrin-G and Homer1b/c together predicted the largest spine head size, while the presence of Homer1b/c correlated with larger spine head sizes than spines without either protein.

Although colocalization supports a functional connection between proteins, in situ PLA confirms protein-protein interaction and allows spatial and quantitative visualization. To this end, we developed an approach combining PLA with SIM (PLA-SIM) to investigate the spatial organization of ankyrin-G-Homer1b/c interaction within spines and its relationship to spine architecture. The majority of interaction occurred in the dendritic shaft and spine head, but not the spine neck, although ankyrin-G is abundant there [28]. Point mutations in the PPXXF motif reduced the interaction in dendritic regions and led to attenuation of exogenous ankyrin-G 190-induced enlargement of spine head size. The effects of the mutations on protein interaction mirrored those on spine architecture. In our interpretation, exogenous expression of WT ankyrin-G 190 mimics its increased delivery into spines upon neuronal activity [28] or the mood stabilizer lithium [25]. Exogenous ankyrin-G 190 likely participates in multiprotein complexes or mesh-like matrix structures with Homer1 and Shank, promoting spine enlargement. On the other hand, while Homer1 interaction-deficient mutants of ankyrin-G 190 are delivered to dendrites and spines, they are unable to participate in such multiprotein matrices, failing to promote spine head enlargement. They may also disrupt Homer1-mediated arrays by competing with endogenous WT ankyrin-G 190.

Remarkably, while exogenous expression of ankyrin-G 190 enhanced perimeter dynamics and morphological index dynamics in dendritic spines, this effect was abolished by the interaction-impairing point mutation in PPXXF. This suggests that a stable ankyrin-G pool within spines is essential for normal levels of spine head remodeling. We hypothesize that Homer1b/c stabilizes the steady-state levels of ankyrin-G within the spine head. Homer1 may thus provide a structural framework for other scaffolds within the PSD, such as ankyrin-G or Shank3, which in turn assemble downstream effector complexes, such as β-spectrin [28].

The unexpected finding that genetic deletion of all Homer1 isoforms causes an increase in ankyrin-G 190 abundance in synaptosomes, prompted us to perform a quantitative analysis of the cortical synaptosomal fraction proteome from Homer1 WT *vs*. KO mice. We found widespread proteomic dysregulation in *HOMER1* KO mouse synaptosomes, characterized primarily by dysregulation of postsynaptic proteins (39% of total). This indicates that loss of Homer1 causes a global reshaping of the postsynaptic proteome and that Homer1 is important for the maintenance of correct synaptic proteome homeostasis. The proteome reshaping was surprisingly characterized by extensive upregulation of synaptic proteins, with significant enrichment of PSD proteins, suggesting perhaps a limiting role for Homer1 in postsynaptic protein networks. In addition to synaptic function, altered proteins also implicated unexpected processes like myelination and the nuclear envelope. The relationship of these with Homer1 is subject to future investigation.

Computational analysis through network seeding around dysregulated PSD proteins allows for the inclusion of proteins not detected by MS but known to interact with the seed proteins, and thus for higher predictive power. This approach indicated that Shank3 and neurofilaments are central nodes in the subnetwork of proteins downregulated in synaptosomes. As Shank3 had the highest centrality in this subnetwork and is known to interact with Homer1, downregulation of Shank3 consequent to the absence of Homer1 may have driven the downregulation of neurofilament proteins in synapses. This also suggests that Homer1 may function in stabilizing Shank3 at the synapse.

Conversely, we found that the subnetwork of proteins that increased in abundance in synaptic membranes was much broader, with multiple nodes including RhoA, Ctnnb1, Grin1, Tsc2, and Ank3. Of these, RhoA and ankyrin-G directly interact with Homer1, and their abundance in synaptic membranes may be limited by Homer1. Our proteomic data suggests that Homer1 is important for the maintenance of synaptic PPI networks, and its loss results primarily in the upregulation of synaptic abundance of postsynaptic and neurodevelopmental disorder-related proteins. Several mechanisms could potentially contribute to the synaptosomal proteome reshaping in *HOMER1* KO mice. Homer1 has been shown to drive the homeostatic downregulation of synaptic proteins and scaling down during sleep [43, 44]. Hence its absence may have the opposite effect, enabling the upregulation of synaptic proteins. Homer1 may also act as an architecturally limiting factor within the structure of the PSD. Homer and Shank form a postsynaptic polymeric network that stabilizes proteins at the PSD [18]. As for ankyrin-G, this may limit their diffusion and restrict their abundance in the PSD, while providing compartmentalized localization. In the absence of Homer1, this scaffold does not exist, proteins freely diffuse in an out of spines, and may be localized there due to other interacting partners, but not in a highly organized network. In addition, we found a robust upregulation of Homer2, which may partially compensate for the absence of Homer1. In the 3-week-old mouse brain Homer1b/c and Homer2a/b, but not Homer3a/b, are highly expressed in the cerebral cortex [45]. Homer1a has also been shown to act as a dominant-negative form of Homer1 [46, 47]. In the *HOMER1* KO mice, all isoforms of Homer1 are lost, including Homer1a, potentially leading to effects opposite to its normal function in inhibiting synaptic transmission and spine morphogenesis. The absence of Homer 1 may also affect mGluR1/5-regulated protein translation [48]. Unexpectedly, the absence of Homer1 only affected the synaptosomal abundance of a few PPXXF-containing proteins. This suggests that the proteome alterations were unlikely to be globally driven by PPXXF domain proteins.

Our data also suggest that phenotypes in *HOMER1* KO mice [14, 15] may be in part caused by a global reshaping of the synaptosome. These data provide novel insights into a role for Homer1 in maintaining normal protein homeostasis in synapses and a potential role for ankyrin-G and Homer1 in neuropsychiatric disorder pathogenesis.

## Supplementary information

Movie 1

Supplementary information

Supplementary table 1

Supplementary table 2
