# Supplementary material for: Homer1 promotes dendritic spine growth through ankyrin-G and its loss reshapes the synaptic proteome
Source: Mol Psychiatry. 2021 Jan 4;26(6):1775–89. doi: 10.1038/s41380-020-00991-1 (PMC8254828; doi:10.1038/s41380-020-00991-1)
Supplement: Supplementary file 2 — Supplementary information [file 41380_2020_991_MOESM2_ESM.docx]

**Supplementary Information**

**Homer1 promotes dendritic spine growth through ankyrin-G and its loss reshapes the synaptic proteome**

Yoon et al.


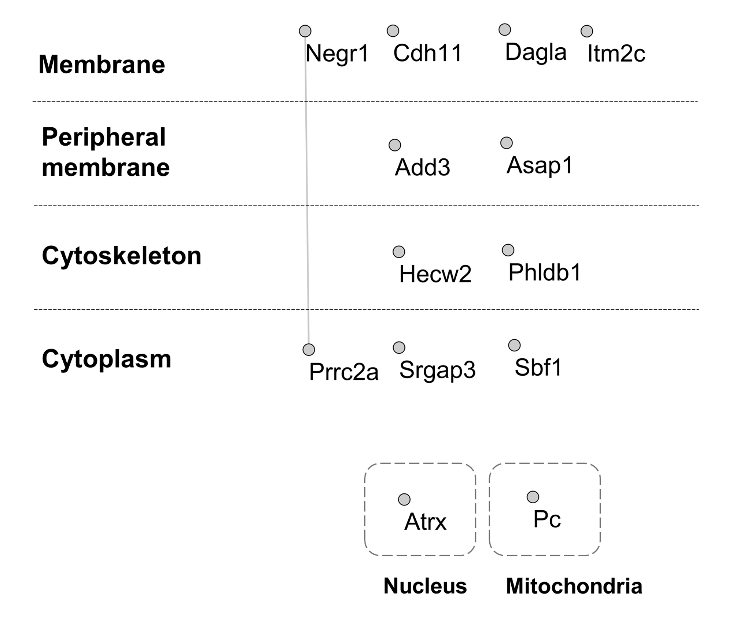


**Supplementary Fig. 1** Psychiatric risk gene-related protein interaction network representing all unconnected nodes with the majority of subnetworks (from Fig. 1e). The size of node means betweenness centrality. No known interactor with Homer1/2/3 was shown in these nodes. Edge refers to predicted protein-protein interaction, including experimental data from the STRING database.


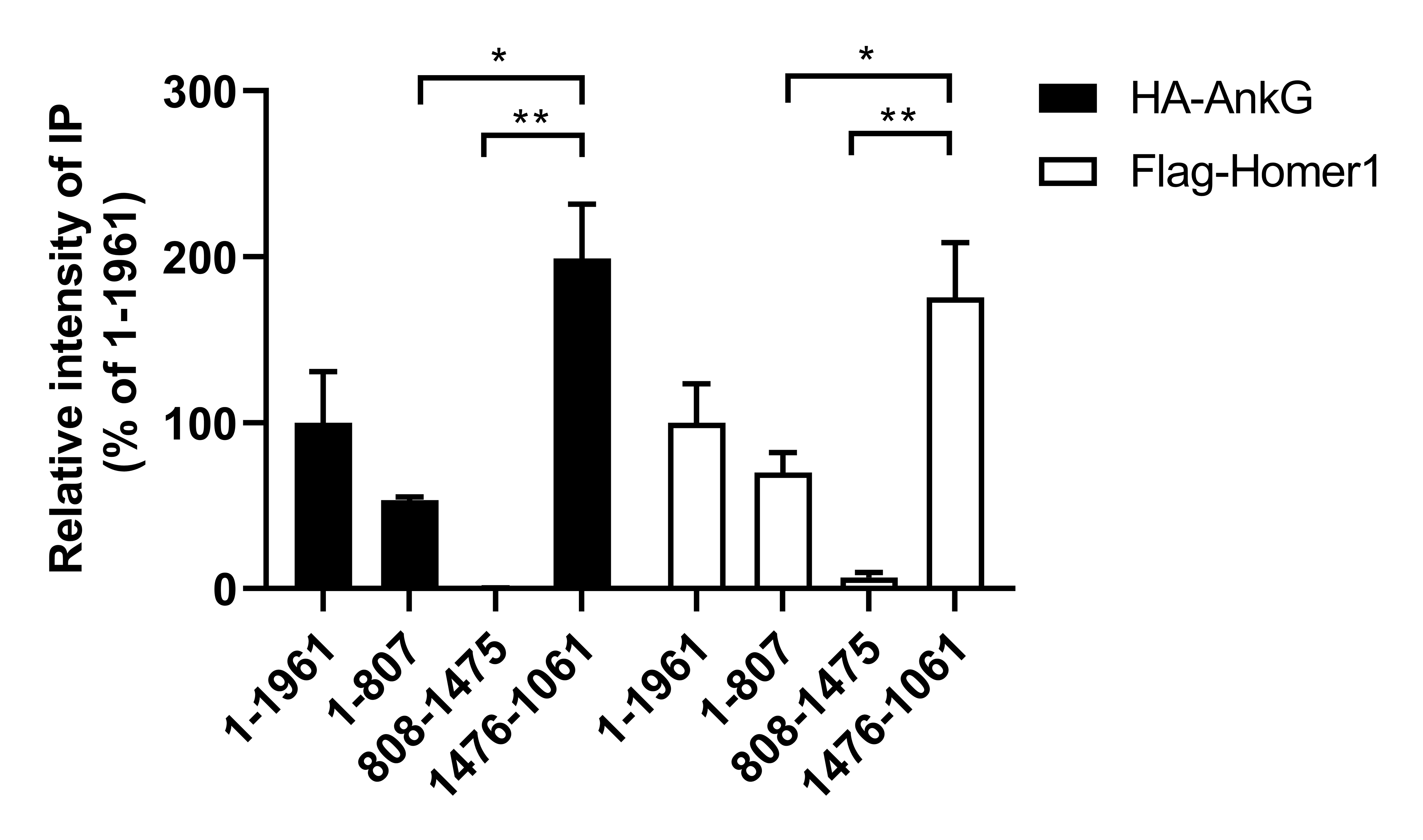


**Supplementary Fig. 2** Binding of ankyrin-G and its truncation mutants to Homer1c. Images related to Fig. 2c were analyzed. The band intensities were analyzed by immunoblotting with HA or Flag antibody. n = 3 per each group. *F*(3, 8) = 14.14; *F*(3, 8) = 10.95; ^*^, *p* < 0.05 ^**^, *p* < 0.01; one-way ANOVA followed by a Bonferroni test. Data are represented as mean ± SEM.


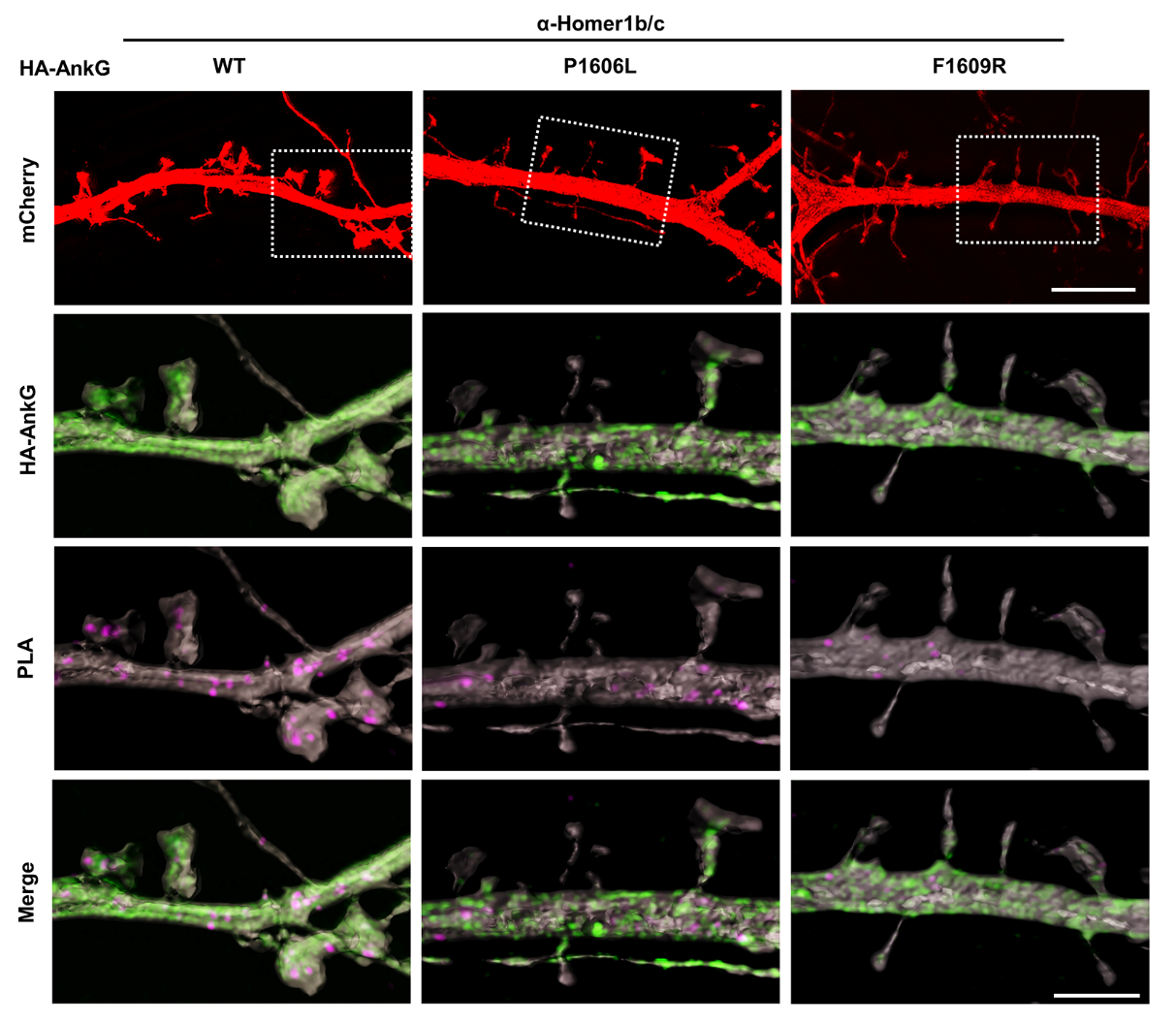


**Supplementary Fig. 3** Representative SIM to detect the interaction between HA-ankyrin-G WT or mutants (P1606L or F1609R) and anti-Homer1b/c by PLA. Images related to Fig. 3a were magnified and reconstituted by Imaris. Scale bar, 5 µm (top); 2 µm (bottom).


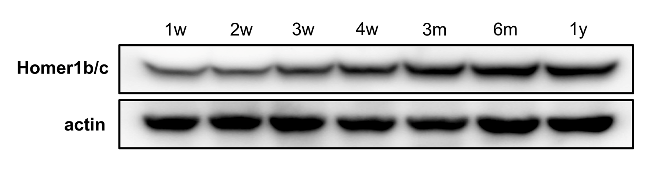


**Supplementary Fig. 4** Protein levels of Homer1b/c in the mouse cortex throughout the lifespan.


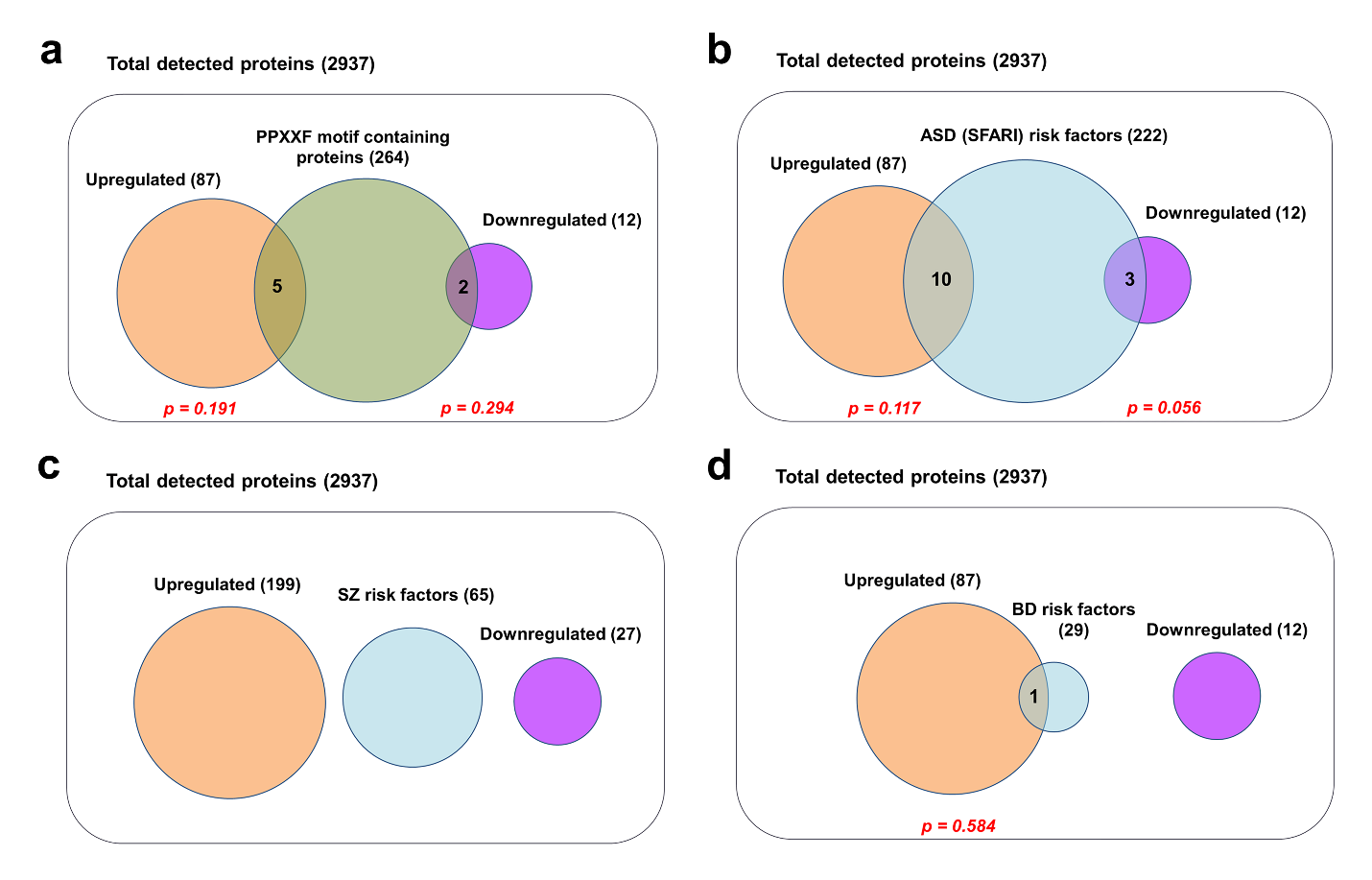


**Supplementary Fig. 5** PPXXF motif-containing proteins or SZ or BD risk factors that were detected through TMT-MS/MS in the cortex of WT and *HOMER1* KO mice. **a** Diagram of PPXXF motif-containing proteins among upregulated or downregulated proteins. **b-c** Diagram of ASD risk factors from SFARI archive (b) or de novo SZ (c) risk factors among upregulated or downregulated proteins. **d** Enrichment of BD risk factors identified through GWAS upregulated or downregulated proteins.


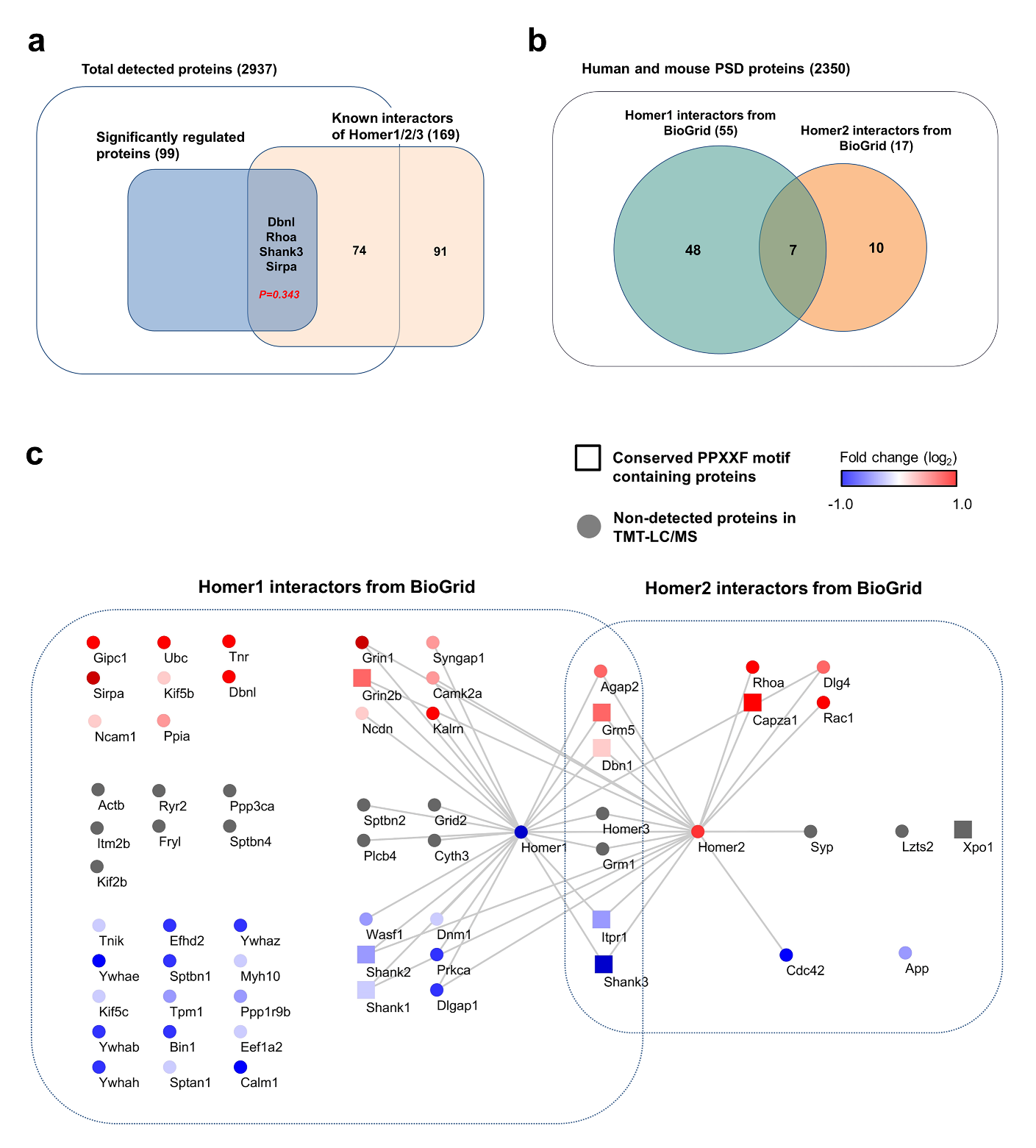


**Supplementary Fig. 6** Analysis of known interactors of Homers. (a) Diagram of known interactors of Homer1/2/3 proteins from BioGrid among significantly upregulated or downregulated proteins in the cortex of WT and *HOMER1* KO mice. (b) Diagram of known interactors of Homer1 (55/102) and 2 (17/27) proteins in human and mouse PSD proteins. (c) Cytoscape analysis of known interactors of Homer1 and 2 proteins from (b). Rectangular node shape refers to conserved PPXXF motif-containing proteins. Edge refers to predicted protein-protein interaction, including experimental data from the STRING database. Non-detected proteins in TMT-LC/MS experiments were color-coded to nodes as gray.
