# Supplementary material for: Homer1 promotes dendritic spine growth through ankyrin-G and its loss reshapes the synaptic proteome
Source: Mol Psychiatry. 2021 Jan 4;26(6):1775–89. doi: 10.1038/s41380-020-00991-1 (PMC8254828; doi:10.1038/s41380-020-00991-1)
Supplement: Supplementary file 3 — Supplementary table 1 [file 41380_2020_991_MOESM3_ESM.pdf]

PPXXF motif containing genes in PSD (227)	Human	Bovine	Mouse	Rat	ASD & SZ risk genes	BD risk genes	Homer1/2/3 interactors in BioGrid and text mining
ABI2	o	o	o	o	X	X	O
ABR	o	o	o	o	X	X	X
ADD3	o	o	o	o	X	O	X
AFG3L2	o	o	o	o	X	X	X
AGAP3	o	o	o	o	X	X	X
AGRN	x	o	o	o	X	X	X
ALDH5A1	x	x	o	x	O	X	X
ANK3 (190KDa)	o	o	o	o	O	O	X
ANXA11	o	o	o	o	X	X	X
ANXA3	o	o	x	x	X	X	X
AP1B1	o	o	o	o	X	X	X
AP2A1	o	o	o	o	X	X	X
AP2B1	o	o	o	o	X	X	X
APOO	o	o	o	x	X	X	X
APOOL	o	o	o	o	X	X	X
ARHGAP21	x	x	o	o	X	X	X
ARHGAP33	x	o	o	o	X	X	X
ARHGAP39	o	x	x	x	X	X	X
ARHGEF7	o	o	o	o	X	X	X
ASAP1	o	o	o	o	O	X	X
ATF7IP	x	x	o	o	X	X	X
ATP1A2	x	x	x	x	X	X	X
ATP8A2	o	o	o	x	X	X	X
ATRX	o	o	o	o	O	X	X
ATXN2	o	o	o	x	X	X	X
BAG6	x	x	o	o	X	X	X
BCAN	o	x	x	x	X	X	X
CACNA1A	o	o	o	o	X	X	X
CACNA1C	o	o	o	o	O	O	O
CACNB1	o	o	o	o	X	X	X
CACNB3	o	o	o	o	X	X	X
CACNB4	o	o	o	o	X	X	X
CAD	o	o	o	o	X	X	X
CALCOCO1	o	o	o	o	X	X	X
CAP2	o	o	o	o	X	X	X
CAPZA1	o	o	o	o	X	X	O
CAPZA2	o	o	o	o	X	X	X
CDH11	o	o	o	o	O	X	X
CKAP4	o	x	o	o	X	X	X
CLASP1	o	o	o	o	O	X	X
CLU	o	x	o	x	X	X	X
CNTN1	o	o	o	o	X	X	X
CNTN6	o	o	o	o	O	X	X
COBL	o	x	x	x	X	X	X
COX7A2L	o	o	o	o	X	X	X
CPEB3	o	o	o	o	X	X	X
CYP1B1	o	o	o	o	X	X	X
DAAM2	o	o	o	x	X	X	X
DACT3	o	o	o	o	X	X	X
DAGLA	o	o	o	o	O	X	X
DBN1	o	o	o	o	X	X	O
DCHS1	x	o	o	o	X	O	X
DCTN1	o	o	o	o	X	X	X
DDX17	o	o	o	o	X	X	X
DECR1	o	x	o	o	X	X	X
DHX30	x	x	x	x	X	X	X
DHX57	o	o	x	x	X	X	X
DNM3	o	x	o	o	X	X	X
DPP10	o	o	o	o	O	X	X

EHMT2	o	o	o	o	X	X	X
EPB41L1	o	o	o	o	O	X	X
EPB41L3	o	o	o	o	X	X	X
EPS15	o	o	o	o	X	X	X
EPS15L1	o	o	o	o	X	X	X
EPS8	o	o	o	o	X	X	X
FAAH	o	o	o	o	X	X	X
FAM120C	x	x	o	x	X	X	X
FAM171A2	o	x	o	o	X	X	X
FASN	o	o	o	o	X	X	X
FBXO6	x	x	o	x	X	X	X
FDPS	o	o	o	o	X	X	X
FGD4	x	x	o	x	X	X	X
FGF2	o	o	o	o	X	X	X
FLNC	o	o	o	o	X	X	X
FMNL2	o	o	o	o	X	X	X
FMNL3	o	o	o	o	X	X	X
FNBP1L	o	o	o	o	X	X	X
FRMPD3	o	o	o	o	X	X	X
FRY	o	o	o	o	X	X	X
G3BP2	o	x	o	x	X	X	X
GAK	o	x	x	x	X	X	X
GDAP1L1	o	o	o	o	X	X	X
GNAS	o	x	x	x	O	X	X
GOT1	o	o	o	o	X	X	X
GPRIN1	o	o	o	o	X	X	X
GRIA3	o	o	o	o	X	X	X
GRIA4	o	o	o	o	X	X	X
GRIN2A	o	o	o	o	O	O	X
GRIN2B	o	o	o	o	O	X	O
GRIN2D	o	o	o	o	X	X	X
GRM1	o	x	o	o	X	X	O
GRM2	o	o	o	o	X	X	X
GRM5	o	o	o	o	O	X	O
GRM7	o	o	o	o	O	X	X
GSN	o	o	o	o	X	X	X
HAX1	o	x	o	x	X	X	X
HDAC11	o	o	o	o	X	X	X
HECW2	o	o	o	o	O	X	X
HTT	o	x	o	x	X	X	X
IGLON5	o	o	o	o	X	X	X
INPP4A	o	o	o	o	X	X	X
IRGQ	o	o	o	o	X	X	X
ITM2B	x	x	x	x	O	X	O
ITM2C	o	o	o	o	O	X	X
ITPR1	o	o	o	o	O	X	O
ITPR2	o	o	o	o	X	X	X
ITPR3	o	o	o	o	X	X	X
KCNJ4	o	o	o	o	X	X	X
KCNMA1	o	o	o	o	O	X	X
KDM3B	o	o	x	o	X	X	X
KIAA1217	o	x	x	x	X	X	X
KIF17	o	o	o	o	X	X	X
KIF1A	o	o	o	o	X	X	X
KIF21A	x	o	o	o	X	X	X
KIF3A	o	o	o	o	X	X	X
KPNA1	o	o	o	o	X	X	X
L1CAM	o	o	x	x	X	X	X
LAP3	o	o	x	x	X	X	X
LIMCH1	o	o	o	o	X	X	X
LIPE	o	o	o	o	X	X	X
LMO7	o	o	o	o	X	X	X
LMTK2	o	x	o	o	X	X	X

LMTK3	o	x	o	x	X	X	X
LRP1	o	o	o	o	O	X	X
LRPPRC	o	o	o	o	X	X	X
LRRC47	o	o	o	o	X	X	X
LRRC8A	o	o	o	o	X	X	X
MAG	o	o	o	o	X	X	X
MAGI2	o	x	o	o	X	X	X
MAGI3	o	x	x	x	X	X	X
MAP2	x	o	o	x	X	X	X
MAP3K12	x	o	o	o	X	X	X
MGST3	o	o	o	o	X	X	X
MKL2	o	o	o	x	O	X	X
MLF1	o	o	o	o	X	X	X
MYCBP2	o	o	o	o	X	X	X
MYH6	o	o	o	o	X	O	X
MYO1C	o	o	o	o	X	X	X
MYO1D	o	o	o	o	X	X	X
MYO1E	x	x	o	x	O	X	X
MYO5C	o	x	x	x	O	X	X
MYO6	o	o	o	o	X	X	X
MYO7B	o	o	o	o	X	X	X
NEGR1	o	o	o	o	O	X	X
NFASC	x	x	x	x	X	X	X
NRCAM	o	x	o	o	O	X	X
NRXN2	o	x	x	o	O	X	X
NTNG2	o	o	o	o	X	X	X
NUMBL	x	x	o	o	X	X	X
OGDHL	o	o	x	x	X	X	X
ORC1	x	o	x	x	X	X	X
PAG1	x	o	x	x	X	X	X
PC	o	o	o	o	X	O	X
PDE4D	x	x	o	x	X	X	X
PDXP	x	x	x	o	X	X	X
PHLDB1	o	o	o	o	O	X	X
PICALM	o	o	o	o	X	X	X
PIGK	o	o	o	o	X	X	X
PLEKHA7	o	o	o	o	X	X	X
PPM1H	o	o	o	o	X	X	X
PREX1	x	x	o	x	O	X	X
PRKAR2A	x	x	x	o	X	X	X
PRRC2A	o	o	o	o	O	X	X
PRRC2C	o	o	o	N/A	X	X	X
PSMD1	o	o	o	o	X	X	X
PTPN23	o	o	x	o	X	X	X
RAP1GDS1	o	o	o	o	X	X	X
RAPGEF6	o	o	o	o	X	X	X
RHOT1	o	o	o	o	X	X	X
RHOT2	x	x	o	x	X	X	X
RIN1	o	o	o	o	X	X	X
ROCK1	o	o	o	o	X	X	X
RPS26	o	o	o	o	X	X	X
RTN1	o	o	o	o	X	X	X
RTN4	x	x	o	o	X	X	X
SACS	o	o	o	o	X	X	X
SBF1	o	o	o	o	O	X	X
SCIN	x	x	x	x	X	X	X
SEC24C	o	o	o	o	X	X	X
SEC31A	o	o	o	o	O	X	X
SGSM1	o	o	o	o	X	X	X
SH3BGRL2	o	o	o	o	X	X	X
SH3PXD2A	o	o	o	o	X	X	X
SHANK1	o	o	o	o	O	X	O
SHANK2	o	o	o	o	O	O	O

SHANK3	o	o	o	o	O	X	O
SIRT2	x	x	x	o	X	X	X
SLC4A4	o	o	o	o	X	X	X
SMARCA1	o	o	o	o	X	X	X
SNX12	o	o	o	o	X	X	X
SNX3	o	o	o	o	X	X	X
SPEG	o	o	o	o	X	X	X
SRGAP3	o	o	o	o	O	X	X
ST14	x	o	x	x	X	X	X
STK38L	o	x	o	x	O	X	X
SYT11	o	o	o	o	X	X	X
TACC1	x	x	o	x	X	X	X
TAOK2	x	x	o	o	O	O	X
TBCD	x	x	x	x	X	X	X
TJP1	o	x	o	o	X	X	X
TJP2	o	o	o	o	X	X	X
TLN1	o	o	o	o	X	X	X
TNC	x	x	x	x	X	X	X
TOMM20	o	o	o	o	X	X	X
TPP1	o	o	o	o	X	X	X
TRAPPC9	o	o	o	o	O	X	X
TRIM46	o	o	o	o	X	X	X
TSG101	o	o	o	o	X	X	X
UBR4	o	o	o	o	X	X	X
UPF1	o	o	o	o	X	X	X
USP14	o	o	o	o	X	X	X
USP9X	o	o	o	o	X	X	X
VAV2	o	x	x	o	X	X	X
VCAN	x	x	o	o	X	X	X
VPS13A	o	o	o	x	X	X	X
VPS16	o	o	o	o	X	X	X
VPS33A	o	o	o	o	X	X	X
VPS35	o	o	o	o	X	X	X
VPS53	x	x	x	x	X	X	X
WASF2	o	o	o	o	X	X	O
WDR1	o	o	o	o	X	X	X
WDR6	o	o	o	o	X	X	X
WIPF2	o	o	o	o	X	X	X
WNK1	x	x	o	x	X	X	X
WNK2	o	x	o	x	X	X	X
XPO1	o	o	o	o	O	X	O
ZDHHC8	x	o	o	o	X	X	X
