# Supplementary material for: Homer1 promotes dendritic spine growth through ankyrin-G and its loss reshapes the synaptic proteome
Source: Mol Psychiatry. 2021 Jan 4;26(6):1775–89. doi: 10.1038/s41380-020-00991-1 (PMC8254828; doi:10.1038/s41380-020-00991-1)
Supplement: Supplementary file 4 — Supplementary table 2 [file 41380_2020_991_MOESM4_ESM.pdf]

Gene Name	Accession	Spec Count	Peptide Num	Norm Int WT	WT	WT	WT	WT	KO	KO	KO	KO	KO	t-test	T-Test *p* value ($-\log_{10}$)	Mean WT	Mean KO		Fold Change KO/WT ($\log_2$)	Description
UROD	P70697	3	3	917.67	2364	1191.33	2839.67	1637.33	4942.67	4444	5502.67	4840	NA	0.000224593	3.648604477	1790	4932.335	Up	1.46231120219963	Uroporphyrinogen decarboxylase OS=Mus musculus GN=Urod PE=1 SV=2
SEPT8	Q8CHH9	15	15	75457.27	80777.8	80498.6	80324.8	82542.33	89369.07	91827	91953.87	85981.47	90588.73	0.000262056	3.581605931	79920.16	89944.028	Up	0.170468022846855	Septin-8 OS=Mus musculus GN=Sept8 PE=1 SV=4
SIRPA	P97797-3	4	4	11546.5	15069.75	19307	17066.75	17060.25	26488.25	31471.75	28490.5	25040	21964.75	0.000959726	3.017852521	16010.05	26691.05	Up	0.737378247365696	Isoform 3 of Tyrosine-protein phosphatase non-receptor type substrate 1 OS=Mus musculus GN=Sirpa
CSNK1E	Q9JMK2	5	5	14625.8	17282.4	14478.2	17797.6	20288.8	25043.2	24109.2	NA	27246.8	NA	0.001098903	2.959040577	16894.56	25466.4	Up	0.592036253101068	Casein kinase I isoform epsilon OS=Mus musculus GN=Csnk1e PE=1 SV=2
SNX17	Q8BVL3	3	3	6511.67	7445.33	5673.33	8408	7029	10043	9676.33	NA	10475.67	NA	0.001301409	2.885586359	7013.466	10065	Up	0.521147678084246	Sorting nexin-17 OS=Mus musculus GN=Snx17 PE=1 SV=2
NTRK3	Q6VNS1-3	1	1	39555	44029	24849	42282	42445	56721	58319	64126	58884	NA	0.002174181	2.662704305	38632	59512.5	Up	0.623396355158407	Isoform 3 of NT-3 growth factor receptor OS=Mus musculus GN=Ntrk3
ICA1	P97411	1	1	10559	9051	21539	19101	20789	37115	27797	37212	44206	NA	0.002991188	2.52415636	16207.8	36582.5	Up	1.17446539345656	Islet cell autoantigen 1 OS=Mus musculus GN=Ica1 PE=2 SV=3
EDC3	Q8K2D3	1	1	810	985	NA	2609	1950	4510	4089	NA	NA	NA	0.004752482	2.323079499	1588.5	4299.5	Up	1.43650380477205	Enhancer of mRNA-decapping protein 3 OS=Mus musculus GN=Edc3 PE=1 SV=1
NEO1	P97798-3	10	10	18809.1	29849.5	28420.4	28459.6	29020	32469.3	40970.1	40986.7	49470.4	50289.1	0.004829091	2.31613461	26911.72	42837.12	Up	0.67062688850246	Isoform 3 of Neogenin OS=Mus musculus GN=Neo1
PI4K2A	Q2TBE6	1	1	3457	5469	2353	6843	5301	9010	9144	NA	9061	NA	0.005181212	2.285568653	4684.6	9071.6667	Up	0.953441763273863	Phosphatidylinositol 4-kinase type 2-alpha OS=Mus musculus GN=Pi4k2a PE=1 SV=1
MPP1	P70290	2	2	35113	38645.5	36243.5	35935.5	39537.5	41326.5	43314	44952.5	49330	41387.5	0.005674816	2.24604821	37095	44062.1	Up	0.248313515084295	55 kDa erythrocyte membrane protein OS=Mus musculus GN=Mpp1 PE=1 SV=1
PLPPR3	Q7TPB0	12	12	19754.08	27167.42	22944.17	36071.75	22537.17	37822.83	41890.75	38569.67	35248.5	40385.17	0.007207917	2.142190233	25694.918	38783.384	Up	0.593955641561002	Lipid phosphate phosphatase-related protein type 3 OS=Mus musculus GN=Lppr3 PE=2 SV=1
KCNIP4	Q6PHZ8	3	3	3759	1698	NA	3009	3010	4585	5229	NA	5434	NA	0.008102188	2.091397668	2869	5082.6667	Up	0.825037650420122	Kv channel-interacting protein 4 OS=Mus musculus GN=Kcnip4 PE=1 SV=1
MRI1	Q9CQT1	1	1	3708	4607	NA	6561	3873	8806	8689	NA	NA	NA	0.008147302	2.088986172	4687.25	8747.5	Up	0.900129014756664	Methylthioribose-1-phosphate isomerase OS=Mus musculus GN=Mri1 PE=2 SV=1
NECAB1	Q8BG18	1	1	3121	6822	3627	7398	6406	10692	8434	NA	10854	NA	0.009354463	2.028981116	5474.8	9993.3333	Up	0.868159714889478	N-terminal EF-hand calcium-binding protein 1 OS=Mus musculus GN=Necab1 PE=1 SV=1
BRSK2	Q69Z98	3	3	35386.67	47967.33	30246.67	50463.67	53025	58898	69298	63834.67	57816.67	NA	0.009750771	2.010961048	43417.868	62461.835	Up	0.524686068092805	Serine/threonine-protein kinase BRSK2 OS=Mus musculus GN=Brsk2 PE=1 SV=2
NEFL	P08551	68	68	88875.59	106017.49	95295.29	77376.84	88685.94	72985.59	69262.99	76791.53	63851.51	42102.6	0.010180514	1.992230306	91250.23	64998.844	Down	-0.489414135120942	Neurofilament light polypeptide OS=Mus musculus GN=Nefl PE=1 SV=5
CKMT2	Q6P8J7	1	1	256532	332158	243949	354207	221003	192188	175625	61041	143884	NA	0.010524467	1.977799902	281569.8	143184.5	Down	-0.975617278315472	Creatine kinase S-type, mitochondrial OS=Mus musculus GN=Ckmt2 PE=1 SV=1
CCT4	P80315	15	15	55461.2	67608.6	70762.93	78567.93	67655.6	81982.13	81894.13	96445.87	76575.73	85984.67	0.010714104	1.970044156	68011.252	84576.506	Up	0.31448351118879	T-complex protein 1 subunit delta OS=Mus musculus GN=Cct4 PE=1 SV=3
PCLO	Q9QYX7	112	112	27288.54	27696.85	26993.79	31662.31	33810.5	35233.64	36155.1	31620.17	40083.21	35418.01	0.012091141	1.917532721	29490.398	35702.026	Up	0.275760652859781	Protein piccolo OS=Mus musculus GN=Pclo PE=1 SV=4
PDLIM5	Q8CI51	2	2	16867.5	18392	15851	17642	20062.5	20121.5	21390	29076	26743	27936	0.012179033	1.9143872	17763	25053.3	Up	0.496125386942747	PDZ and LIM domain protein 5 OS=Mus musculus GN=Pdlim5 PE=1 SV=4
CXADR	P97792	14	14	6818.36	12762	10800.07	16324.29	10172.71	14071.36	20296.57	18382.93	17130.21	18489.07	0.012269576	1.911170455	11375.486	17674.028	Up	0.635702691292211	Coxsackievirus and adenovirus receptor homolog OS=Mus musculus GN=Cxadr PE=1 SV=1
TENM2	Q9WTS5	1	1	16061	18081	18354	29558	22746	30614	32593	25036	36136	29185	0.013443566	1.871485509	20960	30712.8	Up	0.551201327902502	Teneurin-2 OS=Mus musculus GN=Tenm2 PE=2 SV=1
SYT13	Q9EQT6	1	1	21045	26219	28528	27120	26763	34139	30477	35555	30354	28082	0.014899895	1.826816786	25935	31721.4	Up	0.290556075000251	Synaptotagmin-13 OS=Mus musculus GN=Syt13 PE=1 SV=1
CLPB	Q60649	1	1	14057	14254	7001	16433	14031	18989	18256	NA	18144	21831	0.01496045	1.825055342	13155.2	19305	Up	0.553341372152645	Caseinolytic peptidase B protein homolog OS=Mus musculus GN=Clpb PE=1 SV=1
RAB1B	Q9D1G1	2	2	26429.5	37971.5	29408.5	45457.5	37217	44615.5	51493.5	42892	47036.5	58464.5	0.015143774	1.81976587	35296.8	48900.4	Up	0.470308871300677	Ras-related protein Rab-1B OS=Mus musculus GN=Rab1b PE=1 SV=1
CAMSAP3	Q80VC9	23	23	13946.5	16132.45	18437.45	14104.2	17914.7	18787.85	23979.8	28556.25	24998.75	34598.6	0.015202248	1.818092195	16107.06	26184.25	Up	0.701006097126672	Calmodulin-regulated spectrin-associated protein 3 OS=Mus musculus GN=Camsap3 PE=1 SV=1
ECSIT	Q9QZH6	1	1	35678	18189	30317	54132	32443	65851	46843	54921	60480	NA	0.015305392	1.815155531	34151.8	57023.75	Up	0.739601298009352	Evolutionarily conserved signaling intermediate in Toll pathway, mitochondrial OS=Mus musculus GN=Ecsit PE=1 SV=2
ARTN	Q9Z0L2	1	1	22988	23929	28275	18094	33232	38087	35517	64901	39671	54782	0.015990685	1.79613293	25303.6	46591.6	Up	0.88072721983576	Artemin OS=Mus musculus GN=Artn PE=1 SV=1
PDHX	Q8BKZ9	21	21	63450.24	64244.57	58720.9	55594.86	61160.43	64471	63770.38	67284.14	69390	72975.29	0.017066525	1.767854906	60634.2	67578.162	Up	0.156425354152505	Pyruvate dehydrogenase protein X component, mitochondrial OS=Mus musculus GN=Pdhx PE=1 SV=1
HNRNPD	Q60668-4	7	7	152268.71	117043.14	110406.86	122419	124104.29	99408	103667.14	100795.29	102070.86	63256.71	0.017411416	1.759165915	125248.4	93839.6	Down	-0.416523404794797	Isoform 4 of Heterogeneous nuclear ribonucleoprotein D0 OS=Mus musculus GN=Hnrnpd
CAMSAP3	Q80VC9-2	20	20	16150.88	18825.76	21691.12	16016.59	20795.12	21550.59	27714.35	33595.59	28934.47	40704.24	0.01755438	1.755614515	18695.894	30499.848	Up	0.706080593216147	Isoform 2 of Calmodulin-regulated spectrin-associated protein 3 OS=Mus musculus GN=Camsap3
CD200	O54901	8	8	40753.88	50366.25	77121.12	64796.62	53562.75	71858.62	90474.25	90035.88	75115.62	74829.38	0.017733382	1.751208442	57320.124	80462.75	Up	0.489279315665624	OX-2 membrane glycoprotein OS=Mus musculus GN=Cd200 PE=1 SV=1
BRD3	Q8K2F0	1	1	3113	1655	NA	2964	2544	4589	3961	NA	5487	NA	0.018682445	1.72856628	2569	4679	Up	0.864993338459494	Bromodomain-containing protein 3 OS=Mus musculus GN=Brd3 PE=1 SV=2
SYNE1	Q6ZWR6-5	2	2	24107.5	28808.5	36306.5	41641	48255	50355	52851.5	54170	42506.5	57469.5	0.018724061	1.727599958	35823.7	51470.5	Up	0.522831445659712	Isoform 5 of Nesprin-1 OS=Mus musculus GN=Syne1
ZFAND5	O88878	1	1	2629	4029	7446	9226	7160	11007	10204	15570	18427	NA	0.018838183	1.724960978	6098	13802	Up	1.17846928263124	AN1-type zinc finger protein 5 OS=Mus musculus GN=Zfand5 PE=1 SV=1
GNA11	P21278	2	2	44682	66343.5	67961	60077.5	62363.5	66637.5	81352	105112.5	78002.5	84176	0.019316906	1.714062439	60285.5	83056.1	Up	0.462275086166005	Guanine nucleotide-binding protein subunit alpha-11 OS=Mus musculus GN=Gna11 PE=1 SV=1
GNA14	P30677	3	3	35133.33	48134.33	45307.33	52869.33	47057.67	51426	59802.67	70075	54576.67	56117.33	0.019413369	1.711899101	45700.398	58399.534	Up	0.353750127524749	Guanine nucleotide-binding protein subunit alpha-14 OS=Mus musculus GN=Gna14 PE=1 SV=2
KIF1A	P33173	2	2	42747.5	57001	37944	67387.5	79284	87423	95533	NA	76478	NA	0.020131571	1.696122339	56872.8	86478	Up	0.604594324088846	Kinesin-like protein KIF1A OS=Mus musculus GN=Kif1a PE=1 SV=2
FIS1	Q9CQ92	1	1	29414	32924	13671	66570	43230	86118	62021	NA	93801	NA	0.020300438	1.692494588	37161.8	80646.667	Up	1.11779452133568	Mitochondrial fission 1 protein OS=Mus musculus GN=Fis1 PE=1 SV=1
CLVS1	Q9D4C9-2	1	1	6959	8896	4200	14973	9913	13064	14604	16378	17083	NA	0.020826962	1.681374085	8988.2	15282.25	Up	0.765752834500692	Isoform 2 of Clavesin-1 OS=Mus musculus GN=Clvs1
HEPACAM	Q640R3	3	3	16936	17523.67	15846.33	18962.67	15585	19251.67	21596	25988.67	19110.67	20791.67	0.020880262	1.680264056	16970.734	21347.736	Up	0.331034111113346	Hepatocyte cell adhesion molecule OS=Mus musculus GN=Hepacam PE=1 SV=2
STUB1	Q9WUD1	14	14	34854.69	37087.69	43422.62	26077.38	37275.92	40983.15	40202.15	55823.54	55341	49385.15	0.021306042	1.671497213	35743.66	48346.998	Up	0.435738943693402	STIP1 homology and U box-containing protein 1 OS=Mus musculus GN=Stub1 PE=1 SV=1
RGS3	Q9DC04-4	1	1	2966	3628	3641	4043	3427	4633	4229	7605	6797	5692	0.021677555	1.663989707	3541	5791.2	Up	0.709705477658629	Isoform 2 of Regulator of G-protein signaling 3 OS=Mus musculus GN=Rgs3
SHANK3	Q4ACU6-2	6	6	29980.5	34081.67	38454.83	29101.17	35483.33	22829.67	23943	15053.5	28779.33	NA	0.022352147	1.650680755	33420.3	22651.375	Down	-0.561126055486787	Isoform 2 of SH3 and multiple ankyrin repeat domains protein 3 OS=Mus musculus GN=Shank3
PRRT3	Q6PE13	6	6	19985.67	30935	31919	31003.67	40895.17	47866.5	47423.83	41971.17	40212.83	35714.17	0.022569832	1.646471677	30947.702	42637.7	Up	0.462297329978	Proline-rich transmembrane protein 3 OS=Mus musculus GN=Prrt3 PE=1 SV=1
ADGRB1	Q3UHD1	5	5	12022.2	22393.8	21083.6	13264.4	25675.8	27537.4	38251	27594.2	32503.8	21826.6	0.023974312	1.620253856	18887.96	29542.6	Up	0.645329909707753	Brain-specific angiogenesis inhibitor 1 OS=Mus musculus GN=Bai1 PE=1 SV=1
CRBN	Q8C7D2-3	3	3	6246	4995.33	6674.67	4885.67	5674.33	7131.67	6686.33	9128.67	8230.67	11393.33	0.024274951	1.614841642	5695.2	8514.134	Up	0.580113289839728	Isoform 3 of Protein cereblon OS=Mus musculus GN=Crbn
HNRNPD	Q60668	8	8	138176.5	106827.75	100272.75	112774.62	113738	93120.12	96650.62	88195.88	97046.75	55349.62	0.024457253	1.61159233	114357.92	86072.598	Down	-0.409930414663436	Heterogeneous nuclear ribonucleoprotein D0 OS=Mus musculus GN=Hnrnpd PE=1 SV=2
SPRY2	Q9QXV8	1	1	7566	9780	12237	15211	12818	17080	17514	29582	19628	15657	0.025073317	1.600788201	11522.4	19892.2	Up	0.787761594516369	Protein sprouty homolog 2 OS=Mus musculus GN=Spry2 PE=2 SV=1
CHGA	P26339	1	1	37849	36385	25870	27841	31187	37604	43127	NA	39794	NA	0.025695347	1.590145517	31826.4	40175	Up	0.336074046187465	Chromogranin-A OS=Mus musculus GN=Chga PE=1 SV=1
CADPS2	Q8BYR5-7	1	1	25561	36370	41885	39943	48903	46127	68519	55492	49613	81026	0.025708602	1.589921534	38532.4	60155.4	Up	0.642622202933627	Isoform 7 of Calcium-dependent secretion activator 2 OS=Mus musculus GN=Cadps2
HSD17B4	P51660	7	7	45333	50729.57	39845.71	41122.14	42457	48406.86	52420.14	53769	52642.86	46005	0.025767104	1.588934392	43897.484	50648.772	Up	0.206389037176792	Peroxisomal multifunctional enzyme type 2 OS=Mus musculus GN=Hsd17b4 PE=1 SV=3
MAGED1	Q9QYH6	3	3	2348.5	3305.5	2060.5	4249	4599	6033.5	4548	NA	6310	NA	0.026113037	1.583142612	3312.5	5630.5	Up	0.765342587722932	Melanoma-associated antigen D1 OS=Mus musculus GN=Maged1 PE=1 SV=1
BAX	Q07813	2	2	9275	14860	NA	15178	10422	18111	18208	NA	18936	NA	0.026974006	1.56905455	12433.75	18418.333	Up	0.566881041874435	Apoptosis regulator BAX OS=Mus musculus GN=Bax PE=1 SV=1
DBNL	Q62418-3	29	29	60702.19	48950.22	48415.56	40852.48	55824.37	52230.22	59945.07	67671.63	71022.26	72579.96	0.027623438	1.558722269	50948.964	64689.828	Up	0.344486066037914	Isoform 3 of Drebrin-like protein OS=Mus musculus GN=Dbnl
DBNL	Q62418	28	28	62688.04	50482.08	50277.69	42060.08	57614.77	53952.62	61767.35	70274.38	73239.5	75371.5	0.027898402	1.554420673	52624.532	66921.07	Up	0.346725014716458	Drebrin-like protein OS=Mus musculus GN=Dbnl PE=1 SV=2
TIGAR	Q8BZA9	3	3	14378.33	19412	21122	34335.33	21872.67	27579	30080.33	57431.67	41038	41994	0.028186998	1.549951173	22224.066	39624.6	Up	0.83427358280838	Fructose-2,6-bisphosphatase TIGAR OS=Mus musculus GN=Tigar PE=1 SV=1
U2AF2	P26369	2	2	39069.5	32281.5	39621	31062.5	35825	25948	32625	16835	31308	25413	0.028329887	1.547755152	35571.9	26425.8	Down	-0.428790887433287	Splicing factor U2AF 65 kDa subunit OS=Mus musculus GN=U2af2 PE=1 SV=3
RNF7	Q9WTZ1	1	1	34843	45696	37185	44996	53936	51550	56517	NA	55175	NA	0.028382748	1.546945556	43331.2	54414	Up	0.328571694835627	RING-box protein 2 OS=Mus musculus GN=Rnf7 PE=2 SV=1
NEFM	P08553	65	65	74212.81	87116.38	68388.25	86326.39	76543.7	69770.02	65019.33	46970.22	63522.47	30740.66	0.028460749	1.545753679	78517.506	55204.54	Down	-0.508227429754778	Neurofilament medium polypeptide OS=Mus musculus GN=Nefm PE=1 SV=4
DBNL	Q62418-2	29	29	62706.07	50394.96	48415.56	41471.48	57103.41	53544.07	62029.67	67671.63	73600.04	72579.96	0.028604538	1.543565055	52018.296	65885.074	Up	0.340932524781979	Isoform 2 of Drebrin-like protein OS=Mus musculus GN=Dbnl
STX17	Q9D0I4	3	3	31504	43393.33	34137.33	28095	42154.67	41168.67	44882.33	47070	49729	64882.33	0.029083218	1.536357545	35856.866	49546.466	Up	0.466532760247309	Syntaxin-17 OS=Mus musculus GN=Stx17 PE=1 SV=1
MARCH5	Q3KNM2	1	1	5804	9544	4133	14484	9891	17744	16630	NA	12568	NA	0.029612976	1.52851795	8771.2	15647.333	Up	0.83507067119182	E3 ubiquitin-protein ligase MARCH5 OS=Mus musculus GN=March5 PE=2 SV=1
PCDHGA4	Q91XY4	3	3	29238.33	46478	59826	54882.33	50616	55415.33	67832.67	79078.67	57822.67	71656.33	0.029670596	1.527673726	48208.132	66361.134	Up	0.461062011399533	Protocadherin gamma-A4 OS=Mus musculus GN=Pcdhga4 PE=2 SV=1
CCT2	P80314	31	31	47380.83	52117.37	56149.7	46906.07	48417.6	51975.83	56562.67	60126.33	55158.83	65217.03	0.0304731	1.516083357	50194.314	57808.138	Up	0.203748658619603	T-complex protein 1 subunit beta OS=Mus musculus GN=Cct2 PE=1 SV=4
TOM1	O88746	11	11	13979	15942	18221.3	13356.7	18633.9	18081.1	19440.9	26010.3	21211.8	18924.6	0.031036832	1.508122614	16026.58	20733.74	Up	0.371513782879764	Target of Myb protein 1 OS=Mus musculus GN=Tom1 PE=1 SV=1
STMN4	P63042	1	1	201257	189381	183215	173699	184638	179496	157035	113604	164830	133399	0.031359056	1.503637017	186438	149672.8	Down	-0.31688387705463	Stathmin-4 OS=Mus musculus GN=Stmn4 PE=2 SV=1
MFN2	Q80U63	2	2	4319.5	12062	8762.5	22608.5	24860	34643.5	28709.5	NA	27990	19796.5	0.033460056	1.475473338	14522.5	27784.875	Up	0.936009919975143	Mitofusin-2 OS=Mus musculus GN=Mfn2 PE=1 SV=3
HNRNPH2	P70333	14	14	45762.43	36150.79	33636.29	33761.14	34344.79	32723.79	34136.5	18597.93	26678.71	24373.64	0.03366978	1.472759721	36731.088	27302.114	Down	-0.427988967113197	Heterogeneous nuclear ribonucleoprotein H2 OS=Mus musculus GN=Hnrnph2 PE=2 SV=1
CBX3	P23198	5	5	59226.4	42936.2	48185	66463.6	48523.6	40796.8	49853.8	29763	34493.2	13618.6	0.033758434	1.47161771	53066.96	33705.08	Down	-0.654847855199726	Chromobox protein homolog 3 OS=Mus musculus GN=Cbx3 PE=1 SV=2
PPP1R21	Q3TDD9	7	7	27421	39040.57	21212	27045	30514.29	34409.86	36832.14	52933.29	43629	35344.71	0.034794028	1.458495296	29046.572	40629.8	Up	0.484170352323593	Protein phosphatase 1 regulatory subunit 21 OS=Mus musculus GN=Ppp1r21 PE=2 SV=2
HNRNPH1	O35737	10	10	70054.2	55255.5	50899.3	50191.8	52000.3	51123.3	48295.6	21596.2	37988.1	20987.4	0.034954512	1.456496751	55680.22	35998.12	Down	-0.629243347632435	Heterogeneous nuclear ribonucleoprotein H OS=Mus musculus GN=Hnrnph1 PE=1 SV=3
SPG20	Q8R1X6	1	1	18501	33412	16975	26837	30663	34187	34766	NA	51507	56113	0.034970016	1.456304165	25277.6	44143.25	Up	0.804333358127204	Spartin OS=Mus musculus GN=Spg20 PE=1 SV=1
NUP98	Q6PFD9	3	3	12898.67	11934.33	NA	14356	14379	10826	11355.67	NA	8767	NA	0.035286549	1.452390814	13392	10316.223	Down	-0.376456521879702	Nuclear pore complex protein Nup98-Nup96 OS=Mus musculus GN=Nup98 PE=1 SV=2
OXR1	Q4KMM3-4	13	13	36457.3	41740.9	40612.1	51502.5	46224.1	48931.2	49960.6	54966.6	46804.8	57458.5	0.035311488	1.452083983	43307.38	51624.34	Up	0.25343853706431	Isoform 4 of Oxidation resistance protein 1 OS=Mus musculus GN=Oxr1
CTNNB1	Q02248	16	16	35586.8	51530.6	45869	40305.8	56752.67	57132.4	81349.27	58452.67	57997.73	97197.4	0.036113738	1.442327558	46008.974	70425.894	Up	0.614190687863748	Catenin beta-1 OS=Mus musculus GN=Ctnnb1 PE=1 SV=1
RNF157	Q3TEL6	1	1	3151	4696	3624	4142	3552	5818	5720	9588	9641	NA	0.036518068	1.437492202	3833	7691.75	Up	1.00483787431297	RING finger protein 157 OS=Mus musculus GN=Rnf157 PE=2 SV=2
MAPK8IP2	Q9ERE9	2	2	1293	2942.5	1611	2334	2205.5	2736	2908.5	2725.5	3516.5	NA	0.037459592	1.426436953	2077.2	2971.625	Up	0.516611937971094	C-Jun-amino-terminal kinase-interacting protein 2 OS=Mus musculus GN=Mapk8ip2 PE=1 SV=1
SHANK3	Q4ACU6	20	20	57700.1	49016.4	69718.9	32545.2	54951.65	29460.35	37461.1	41469.7	40580.35	9777.35	0.037808781	1.422407325	52786.45	31749.77	Down	-0.733421504161475	SH3 and multiple ankyrin repeat domains protein 3 OS=Mus musculus GN=Shank3 PE=1 SV=3
NUDT8	Q9CR24	1	1	9115	15393	NA	16063	18964	29906	20717	25991	42017	NA	0.038820309	1.410941008	14883.75	29657.75	Up	0.994671088106163	Nucleoside diphosphate-linked moiety X motif 8, mitochondrial OS=Mus musculus GN=Nudt8 PE=1 SV=1
4931406C07RIK	Q91V76	2	2	35660.5	29242	20083	36996	31021	32850	45535.5	NA	42034	48164	0.038916754	1.409863388	30600.5	42145.875	Up	0.461836209068018	Ester hydrolase C11orf54 homolog OS=Mus musculus PE=2 SV=1
TOM1	O88746-2	7	7	18996.14	19975.14	25300.86	15771.43	23922.43	22686.14	26416.43	37157.57	27667.71	27035.14	0.039653169	1.401722096	20793.2	28192.598	Up	0.439204630097151	Isoform 2 of Target of Myb protein 1 OS=Mus musculus GN=Tom1
RPL36A	P83882	5	5	100170.2	123596.2	121571.2	118735.4	112857	114021	154857.4	125957.6	149862	147485	0.040656197	1.390873247	115386	138436.6	Up	0.262757224787035	60S ribosomal protein L36a OS=Mus musculus GN=Rpl36a PE=1 SV=2
MRPS27	Q8BK72	1	1	36289	67245	59384	75665	70322	94941	109623	101134	65523	NA	0.040701213	1.390392652	61781	92805.25	Up	0.587043198114935	28S ribosomal protein S27, mitochondrial OS=Mus musculus GN=Mrps27 PE=2 SV=2
BRSK2	Q69Z98-4	2	2	41110.5	56308.5	33123	64533.5	73203.5	71115	82967	88367	64245	NA	0.041410207	1.382892604	53655.8	76673.5	Up	0.514993908058695	Isoform 4 of Serine/threonine-protein kinase BRSK2 OS=Mus musculus GN=Brsk2
RCAN1	Q9JHG6	2	2	62513.5	56699.5	52225.5	42160.5	65149.5	58733	62090.5	74094	80061.5	72265	0.042167073	1.37502654	55749.7	69448.8	Up	0.316985724584554	Calcipressin-1 OS=Mus musculus GN=Rcan1 PE=1 SV=1
ANKRD63	A2ARS0	4	4	9346.25	10600.5	8344	8084.75	12663.25	13708.5	11827.5	11228.75	15608.5	NA	0.04239118	1.372724496	9807.75	13093.313	Up	0.416836023152212	Ankyrin repeat domain-containing protein 63 OS=Mus musculus GN=Ankrd63 PE=4 SV=1
MTMR7	Q9Z2C9	2	2	1074	916	1323	4227	3327.5	5317	3874.5	3292.5	4219	NA	0.042895948	1.36758373	2173.5	4175.75	Up	0.942015245468839	Myotubularin-related protein 7 OS=Mus musculus GN=Mtmr7 PE=2 SV=2
ERMN	Q5EBJ4	6	6	39584	35532	22732.67	27654.5	36345	32035.33	21448.83	19524.17	22618.5	18368.5	0.042996016	1.366571789	32369.634	22799.066	Down	-0.505666329936641	Ermin OS=Mus musculus GN=Ermn PE=1 SV=1
CDC42BPA	Q3UU96	2	2	4927	6717	NA	15306	11691	18147	14409	NA	19489	NA	0.043113013	1.365391624	9660.25	17348.333	Up	0.844664638208042	Serine/threonine-protein kinase MRCK alpha OS=Mus musculus GN=Cdc42bpa PE=2 SV=2
NENF	Q9CQ45	3	3	19850.67	24803.67	20958	29142	24489.67	24352.67	30789.67	41368.67	29333	39703.67	0.043316998	1.363341649	23848.802	33109.536	Up	0.473329995857521	Neudesin OS=Mus musculus GN=Nenf PE=1 SV=1
SMARCA2	Q6DIC0	3	3	433.5	1137.5	NA	3100	2389	3892.5	3490.5	NA	NA	NA	0.044531698	1.351330748	1765	3691.5	Up	1.06453897502077	Probable global transcription activator SNF2L2 OS=Mus musculus GN=Smarca2 PE=1 SV=1
CHMP7	Q8R1T1	1	1	1614	3151	2603	1933	2222	3869	3052	5818	6345	NA	0.044750712	1.349200047	2304.6	4771	Up	1.04977531661945	Charged multivesicular body protein 7 OS=Mus musculus GN=Chmp7 PE=1 SV=1
TCOF1	O08784	1	1	1075	728	NA	1155	544	1696	2908	NA	2133	NA	0.044945665	1.347312189	875.5	2245.6667	Up	1.35896471483257	Treacle protein OS=Mus musculus GN=Tcof1 PE=1 SV=1
SHANK3	Q4ACU6-3	16	16	63739.75	51628.19	74395.44	32512.25	58723.81	30682.56	39969.31	46192.06	41917.81	12221.69	0.045111004	1.345717509	56199.888	34196.686	Down	-0.716710734977208	Isoform 4 of SH3 and multiple ankyrin repeat domains protein 3 OS=Mus musculus GN=Shank3
CEP170B	Q80U49-2	7	7	19888.17	16484.83	14389.67	16872.5	21081.17	21449	18973.33	26643	23872.83	33854.83	0.04573695	1.339732799	17743.268	24958.598	Up	0.492265142592893	Isoform 2 of Centrosomal protein of 170 kDa protein B OS=Mus musculus GN=Cep170b
SUN1	Q9D666-3	1	1	13908	18663	15491	15353	21201	21679	22284	NA	28008	NA	0.04641133	1.333375986	16923.2	23990.333	Up	0.503450810489996	Isoform 3 of SUN domain-containing protein 1 OS=Mus musculus GN=Sun1
PLCL2	Q8K394	5	5	24041	34562.33	28315.33	49645.67	33681.67	50087.33	41957.33	NA	56384.33	NA	0.046571627	1.331878591	34049.2	49476.33	Up	0.539117587739717	Inactive phospholipase C-like protein 2 OS=Mus musculus GN=Plcl2 PE=1 SV=2
GLUL	P15105	56	56	56008.2	55792.35	55488.2	57709.47	56921.56	59277.22	59893.56	60470.85	61687.53	70971.44	0.047216456	1.325906615	56383.956	62460.12	Up	0.147650637559849	Glutamine synthetase OS=Mus musculus GN=Glul PE=1 SV=6

FCHSD2	Q3USJ8-3	1	1	7366	6960	3816	11163	10216	14202	11436	9066	14210	NA	0.047371197	1.324485642	7904.2	12228.5	Up	0.629556090572498	Isoform 3 of FCH and double SH3 domains protein 2 OS=Mus musculus GN=Fchsd2
FARP1	F8VPU2	1	1	11853	19297	10878	29448	22283	30233	24961	NA	30288	NA	0.048091949	1.317927625	18751.8	28494	Up	0.603629074345114	FERM, RhoGEF and pleckstrin domain-containing protein 1 OS=Mus musculus GN=Farp1 PE=1 SV=1
CHMP6	P0C0A3	2	2	6174	5147	5011	7995	6817	7472	7665	7622	8270	NA	0.048440627	1.31479024	6228.8	7757.25	Up	0.316591047651031	Charged multivesicular body protein 6 OS=Mus musculus GN=Chmp6 PE=2 SV=2
PCDHA4	O88689	1	1	14366	23643	NA	31432	23080	34249	36444	NA	30599	NA	0.048740836	1.31210703	23130.25	33764	Up	0.545704970129329	Protocadherin alpha-4 OS=Mus musculus GN=Pcdha4 PE=1 SV=1
ARPC3	Q9JM76	3	3	92684.33	95938	111727	111626	87656	108442.33	113118.67	155930	113532.67	130864	0.049264966	1.307461808	99926.266	124377.53	Up	0.315790067766672	Actin-related protein 2/3 complex subunit 3 OS=Mus musculus GN=Arpc3 PE=1 SV=3
MAGI1	Q6RHR9	2	2	14183.5	14500.5	9602.5	16511.5	18188	21567.5	20198.5	16753	27918	NA	0.049432366	1.305988606	14597.2	21609.25	Up	0.565957339601352	Membrane-associated guanylate kinase, WW and PDZ domain-containing protein 1 OS=Mus musculus GN=Magi1 PE=1 SV=1
RHOA	Q9QUI0	7	7	42077.29	65709.71	62697.29	65396.71	54438.29	61407.43	74916	93259.57	64060.57	88971.57	0.04957086	1.304773549	58063.858	76523.028	Up	0.398253528247191	Transforming protein RhoA OS=Mus musculus GN=Rhoa PE=1 SV=1
2310035C23RIK	Q148V7-3	1	1	21147	11237	14744	41355	31281	44839	39077	NA	34464	NA	0.050748228	1.294579117	23952.8	39460	Up	0.720196644150089	Isoform 3 of LisH domain and HEAT repeat-containing protein KIAA1468 OS=Mus musculus GN=Kiaa1468
DDRGK1	Q80WW9	1	1	3658	10773	NA	12903	10895	17237	15590	NA	14089	NA	0.050855959	1.293658155	9557.25	15638.667	Up	0.710450053068058	DDRGK domain-containing protein 1 OS=Mus musculus GN=Ddrgk1 PE=1 SV=2
PFDN2	O70591	4	4	48187.75	52115.75	43620.25	46150	45023	57438.5	59107.25	47054	77782.75	60947	0.051118485	1.291422025	47019.35	60465.9	Up	0.362867162578135	Prefoldin subunit 2 OS=Mus musculus GN=Pfdn2 PE=2 SV=2
HBS1L	Q69ZS7	1	1	11099	14222	6573	14204	11337	14749	16347	NA	14801	NA	0.05120068	1.290724272	11487	15299	Up	0.413435290020907	HBS1-like protein OS=Mus musculus GN=Hbs1l PE=1 SV=2
TTC9	Q3V038	3	3	4560	4421.67	2687.33	5543.67	4284.33	5461.67	5700.33	NA	5490.67	NA	0.052016247	1.283860987	4299.4	5550.89	Up	0.368583764527572	Tetratricopeptide repeat protein 9A OS=Mus musculus GN=Ttc9 PE=2 SV=1
PSD	Q5DTT2-2	3	3	76978	81927.33	31989	62994.33	75197.67	132721.67	71719.33	108199	100996.67	NA	0.05283362	1.277089635	65817.266	103409.17	Up	0.651826085759903	Isoform 2 of PH and SEC7 domain-containing protein 1 OS=Mus musculus GN=Psd
PLPPR3	Q7TPB0-2	1	1	NA	6202	NA	13774	8558	19174	14621	NA	24244	NA	0.053616682	1.270700065	9511.3333	19346.333	Up	1.02434065894124	Isoform 2 of Lipid phosphate phosphatase-related protein type 3 OS=Mus musculus GN=Lppr3
SEC23A	Q01405	4	4	38302	63027	72242.25	79715.25	61735.5	83143.25	90731.75	91453.75	64137	125951	0.054257205	1.26554258	63004.4	91083.35	Up	0.5317347693037	Protein transport protein Sec23A OS=Mus musculus GN=Sec23a PE=1 SV=2
LARP4B	Q6A0A2-3	2	2	10284	11359.5	5394	14361.5	11020	13721	13366.5	14223.5	14767	15644.5	0.054258056	1.265535773	10483.8	14344.5	Up	0.452335944916311	Isoform 3 of La-related protein 4B OS=Mus musculus GN=Larp4b
FAM96B	Q9D187	1	1	6284	6235	3132	6641	6363	7282	7030	8372	7224	NA	0.054825711	1.261015724	5731	7477	Up	0.383672636788831	Mitotic spindle-associated MMXD complex subunit MIP18 OS=Mus musculus GN=Fam96b PE=2 SV=1
SDK2	Q6V4S5-2	1	1	3475	5606	NA	7077	3531	8138	7541	NA	6731	NA	0.055092019	1.258911309	4922.25	7470	Up	0.601790309192272	Isoform 2 of Protein sidekick-2 OS=Mus musculus GN=Sdk2
SPON1	Q8VCC9	1	1	4777	6214	4482	3222	5416	5770	9248	6740	6417	NA	0.055175761	1.258251669	4822.2	7043.75	Up	0.546652217549617	Spondin-1 OS=Mus musculus GN=Spon1 PE=2 SV=1
2610301B20RIK	Q3UJP5	7	7	17581.86	21833	18268.43	17694.86	19960.71	20133.14	22234.71	20483	24645.86	20822.86	0.055340073	1.256960276	19067.772	21663.914	Up	0.184157637104165	Protein C8orf37 homolog OS=Mus musculus PE=1 SV=1
WNK3	Q80XP9	1	1	20717	25565	NA	26113	15673	27243	36288	NA	30135	NA	0.055584994	1.25504244	22017	31222	Up	0.503945052508557	Serine/threonine-protein kinase WNK3 OS=Mus musculus GN=Wnk3 PE=2 SV=3
NDUFAF2	Q59J78	2	2	13027	12749.5	11403.5	10437	11384.5	13416	13973	NA	12546.5	NA	0.056172483	1.250476375	11800.3	13311.833	Up	0.173885738082068	Mimitin, mitochondrial OS=Mus musculus GN=Ndufaf2 PE=2 SV=1
NEFH	P19246	23	23	108805.48	131166.67	90077.52	71687.43	113262.1	87275.43	86761.52	57393.76	78798.67	17842.95	0.056496163	1.247981045	102999.84	65614.466	Down	-0.650556270796563	Neurofilament heavy polypeptide OS=Mus musculus GN=Nefh PE=1 SV=3
LACTB	Q9EP89	4	4	54321.25	56899.5	56201.25	37467	50795.5	53879	57063.25	65782.75	61139.5	74981.5	0.056814618	1.245539908	51136.9	62569.2	Up	0.291087953313779	Serine beta-lactamase-like protein LACTB, mitochondrial OS=Mus musculus GN=Lactb PE=1 SV=1
SNX1	Q9WV80	1	1	27342	38842	22091	27432	22519	28934	39344	NA	48257	41242	0.056904998	1.244849585	27645.2	39444.25	Up	0.512785997221288	Sorting nexin-1 OS=Mus musculus GN=Snx1 PE=1 SV=1
CAPZA2	P47754	6	6	91597.33	121201.17	125903.67	90724.83	118672.17	115126	141236.67	141582.67	118098.83	141006.33	0.057337293	1.241562816	109619.83	131410.1	Up	0.261567308390793	F-actin-capping protein subunit alpha-2 OS=Mus musculus GN=Capza2 PE=1 SV=3
RIMS1	Q99NE5-4	31	31	41564.7	44901.52	39503.41	41722.63	55983.37	50202.19	53666.85	47740.59	63448.22	52761.7	0.057558197	1.239892819	44735.126	53563.91	Up	0.25985319693756	Isoform 4 of Regulating synaptic membrane exocytosis protein 1 OS=Mus musculus GN=Rims1
SCRIB	Q80U72-3	3	3	5016	6962.33	5449.67	5770	9634.67	9292	8357	9678.33	7741.67	NA	0.058550748	1.232467555	6566.534	8767.25	Up	0.41699231058882	Isoform 3 of Protein scribble homolog OS=Mus musculus GN=Scrib
SLC9A1	Q61165	8	8	6246.2	9020.6	3797.4	12052.6	12624.8	13944	15625	9520	14046.6	13040.6	0.059075972	1.228589121	8748.32	13235.24	Up	0.597306458032958	Sodium/hydrogen exchanger 1 OS=Mus musculus GN=Slc9a1 PE=1 SV=1
DPYSL4	O35098	59	59	117058.15	122190.07	130061.59	98944.03	119560.75	113717.22	130886.81	137950.34	152019.36	156650.56	0.060660795	1.217091899	117562.92	138244.86	Up	0.233792747276704	Dihydropyrimidinase-related protein 4 OS=Mus musculus GN=Dpysl4 PE=1 SV=1
DPYSL4	O35098-2	58	58	118986.93	124193.34	132195.91	100600.72	121535.78	115599.62	133060.05	140190.07	154496.34	159351.43	0.060677017	1.216975776	119502.54	140539.5	Up	0.233934457106075	Isoform 2 of Dihydropyrimidinase-related protein 4 OS=Mus musculus GN=Dpysl4
ATAD2	Q8CDM1	1	1	NA	26166	NA	47066	52624	73603	56093	NA	82337	NA	0.061737518	1.209450835	41952	70677.667	Up	0.752514821260306	ATPase family AAA domain-containing protein 2 OS=Mus musculus GN=Atad2 PE=1 SV=1
DLST	Q9D2G2-2	7	7	56525.43	61154.29	54867.14	47304	47084.29	47763.14	47129.14	45486	33189.14	NA	0.061922314	1.208152825	53387.03	43391.855	Down	-0.299065029334656	Isoform 2 of Dihydrolipoyllysine-residue succinyltransferase component of 2-oxoglutarate dehydrogenase complex, mitochondrial OS=Mus musculus GN=Dlst
FAM171B	Q14CH0	1	1	4648	18103	NA	21944	15844	24135	31873	NA	22788	NA	0.063158742	1.199566532	15134.75	26265.333	Up	0.795295049325663	Protein FAM171B OS=Mus musculus GN=Fam171b PE=1 SV=2
NCLN	Q8VCM8	1	1	4045	4955	6573	5116	5874	8705	7060	18777	7053	14858	0.063417494	1.197790925	5312.6	11290.6	Up	1.08763215759344	Nicalin OS=Mus musculus GN=Ncln PE=2 SV=2
ATL1	Q8BH66	1	1	9719	12105	20730	20656	10914	16510	22579	27705	17862	23682	0.063481183	1.197354991	14824.8	21667.6	Up	0.547526721306972	Atlastin-1 OS=Mus musculus GN=Atl1 PE=1 SV=1
SDHAF2	Q8C6I2	1	1	83543	78360	85886	42148	87513	77795	84695	158144	120324	163725	0.063505599	1.197187981	75490	120936.6	Up	0.679893473423218	Succinate dehydrogenase assembly factor 2, mitochondrial OS=Mus musculus GN=Sdhaf2 PE=2 SV=1
SLIRP	Q9D8T7	9	9	72456.78	75484.67	67848.56	78077.22	76992.22	79302.22	71601.33	91672.56	97205.22	88289	0.064499467	1.190443876	74171.89	85614.066	Up	0.206975313002993	SRA stem-loop-interacting RNA-binding protein, mitochondrial OS=Mus musculus GN=Slirp PE=1 SV=2
ATG16L1	Q8C0J2-3	1	1	3081	3141	NA	5057	3264	5192	4485	NA	5165	NA	0.064677046	1.189249825	3635.75	4947.3333	Up	0.44439810539994	Isoform 3 of Autophagy-related protein 16-1 OS=Mus musculus GN=Atg16l1
ADAM17	Q9Z0F8	1	1	6451	13358	NA	18920	15967	28138	20021	NA	20025	NA	0.065199877	1.185753224	13674	22728	Up	0.733035406203088	Disintegrin and metalloproteinase domain-containing protein 17 OS=Mus musculus GN=Adam17 PE=1 SV=3
FAM189A1	Q6A044	1	1	13925	11658	24119	12456	17326	17337	19244	28978	26658	41318	0.065474925	1.183924993	15896.8	26707	Up	0.748481544426282	Protein FAM189A1 OS=Mus musculus GN=Fam189a1 PE=2 SV=3
HIST1H2AK	Q8CGP7	6	6	479723.67	260643.5	311687.17	90220.83	325429.67	198972.83	183378.67	117133.5	168528.5	1538.17	0.066351005	1.178152497	293540.97	133910.33	Down	-1.1322945676607	Histone H2A type 1-K OS=Mus musculus GN=Hist1h2ak PE=1 SV=3
OPLAH	Q8K010	1	1	2265	6014	6434	15510	10229	14400	12316	14264	NA	NA	0.066812922	1.175139531	8090.4	13660	Up	0.755674545359449	5-oxoprolinase OS=Mus musculus GN=Oplah PE=2 SV=1
SORBS2	Q3UTJ2-2	20	20	51499.9	55081.35	60464.75	39681.95	64104.9	53516.9	62274.7	83863.75	72340.2	69032.4	0.067067128	1.173490292	54166.57	68205.59	Up	0.332487246978324	Isoform 2 of Sorbin and SH3 domain-containing protein 2 OS=Mus musculus GN=Sorbs2
SRR	Q9QZX7	1	1	5147	4818	9575	12813	7772	10128	14264	NA	16071	NA	0.067171097	1.172817556	8025	13487.667	Up	0.749067489812451	Serine racemase OS=Mus musculus GN=Srr PE=1 SV=1
GRIA3	Q9Z2W9	1	1	5457	4932	4200	9880	5596	7674	9237	NA	8653	NA	0.06720068	1.172626331	6013	8521.3333	Up	0.502994228124603	Glutamate receptor 3 OS=Mus musculus GN=Gria3 PE=1 SV=2
PAK1	O88643	11	11	50765.82	50873.82	60002.73	59716.55	66166.27	57842.55	73889.18	69105.55	64263.36	89001.64	0.067557327	1.170327539	57505.038	70820.456	Up	0.300477777313365	Serine/threonine-protein kinase PAK 1 OS=Mus musculus GN=Pak1 PE=1 SV=1
NT5C	Q9JM14	5	5	62251.4	55437.6	67325.6	42446	59617.4	60585.8	66068.6	81578.2	85546.4	63307	0.067558737	1.170318478	57415.6	71417.2	Up	0.314828797417312	5'(3')-deoxyribonucleotidase, cytosolic type OS=Mus musculus GN=Nt5c PE=1 SV=1
SLC17A6	Q8BLE7	2	2	15119	36287.5	30489	25304.5	28712.5	34822.5	34403	34152	39485	36221.5	0.067759567	1.169029375	27182.5	35816.8	Up	0.397958299365196	Vesicular glutamate transporter 2 OS=Mus musculus GN=Slc17a6 PE=1 SV=1
FRMD8	Q3UFK8	1	1	76328	77114	65545	62939	65138	75213	74884	77215	80242	NA	0.067912515	1.168050188	69412.8	76888.5	Up	0.147566108306264	FERM domain-containing protein 8 OS=Mus musculus GN=Frmd8 PE=1 SV=2
LZTS1	P60853	5	5	10669.5	12915.5	19933	8592.5	12442.75	14824.75	14569	39260.5	23626.75	30988.5	0.068075899	1.167006612	12910.65	24653.9	Up	0.933254248114359	Leucine zipper putative tumor suppressor 1 OS=Mus musculus GN=Lzts1 PE=2 SV=3
GPC6	Q9R087	2	2	7273.5	10457.5	3317	23201	14277.5	25268	21511	13620	22809.5	NA	0.069183399	1.159998107	11705.3	20802.125	Up	0.829569001202484	Glypican-6 OS=Mus musculus GN=Gpc6 PE=1 SV=1
HIST1H2AH	Q8CGP6	7	7	433925.57	231455.43	273275	85893.86	293726.29	180890.29	168119.29	109428	154012.86	1318.43	0.06949906	1.158021067	263655.23	122753.77	Down	-1.10288523491886	Histone H2A type 1-H OS=Mus musculus GN=Hist1h2ah PE=1 SV=3
HMOX2	O70252	2	2	55029	93670	74521.5	58903.5	104507.5	89634.5	111331.5	78370.5	128646.5	118439	0.069937469	1.155290087	77326.3	105284.4	Up	0.445260599893296	Heme oxygenase 2 OS=Mus musculus GN=Hmox2 PE=2 SV=1
SORBS2	Q3UTJ2	18	18	57005.56	61075	67183.06	43406.61	71070.94	59032.33	68875.5	93181.94	80184.61	76702.67	0.069994174	1.154938105	59948.234	75595.41	Up	0.334581385314409	Sorbin and SH3 domain-containing protein 2 OS=Mus musculus GN=Sorbs2 PE=1 SV=2
CASKIN1	Q6P9K8-3	44	44	38825.12	39295.98	43802.24	33396.98	40394.07	38518.98	41804.26	47731.52	56289.83	50367.76	0.07014298	1.154015785	39142.878	46942.47	Up	0.262143920254834	Isoform 3 of Caskin-1 OS=Mus musculus GN=Caskin1
KLC1	O88447	17	17	58044.73	62263.47	62242.53	46388.13	65456.87	60488.67	67647.8	65963.87	71205.73	85386.4	0.070294535	1.153078436	58879.146	70138.494	Up	0.252449707373525	Kinesin light chain 1 OS=Mus musculus GN=Klc1 PE=1 SV=3
ALYREF	O08583	4	4	33566	21040.67	22433.67	13518.67	22201.67	16693.67	16392	7698	20807	7589.67	0.070428587	1.152251024	22552.136	13836.068	Down	-0.704830073936322	THO complex subunit 4 OS=Mus musculus GN=Alyref PE=1 SV=3
CAPZA1	P47753	6	6	48145	61720.33	66954.5	47992	69828.83	71442.67	82125.67	87015.33	62344.67	62727.33	0.070781721	1.150078883	58928.132	73131.134	Up	0.311529199178588	F-actin-capping protein subunit alpha-1 OS=Mus musculus GN=Capza1 PE=1 SV=4
HIST3H2A	Q8BFU2	8	8	385604	204350.25	241445.12	75296.12	259417.38	159435.38	148299.25	96666.5	137552.5	1153.62	0.070967528	1.148940324	233222.57	108621.45	Down	-1.10239840894194	Histone H2A type 3 OS=Mus musculus GN=Hist3h2a PE=1 SV=3
VASP	P70460	2	2	40663	42349.5	51891.5	40036.5	47021.5	42295.5	50937	67369.5	64705	84436	0.071161069	1.147757535	44392.4	61948.6	Up	0.480758971081707	Vasodilator-stimulated phosphoprotein OS=Mus musculus GN=Vasp PE=1 SV=4
DUSP3	Q9D7X3	10	10	64200.5	54900.2	62228	49999	54753.1	59080	59167.4	70375.5	67931.5	80951.2	0.071778494	1.144005656	57216.16	67501.12	Up	0.238488764005589	Dual specificity protein phosphatase 3 OS=Mus musculus GN=Dusp3 PE=1 SV=1
CTTNBP2	B9EJA2-3	11	11	23703.09	24806.45	19903.64	24790.45	22317	24636.73	23385.27	28126.45	28718.45	25035.82	0.072357763	1.14051487	23104.126	25980.544	Up	0.169281124126883	Isoform 3 of Cortactin-binding protein 2 OS=Mus musculus GN=Cttnbp2
2610002M06RIK	Q9CQD4	1	1	53356	42515	55929	38526	39812	35534	35642	32422	38806	43663	0.072974593	1.13682832	46027.6	37213.4	Down	-0.306677010383773	Charged multivesicular body protein 1b-2 OS=Mus musculus GN=Chmp1b2 PE=2 SV=2
ARSB	P50429	4	4	32457.5	32278.5	28801.25	54093	34201	51233.75	40670.5	78766.25	45960	49635	0.073077235	1.136217895	36366.25	53253.1	Up	0.550265344544353	Arylsulfatase B OS=Mus musculus GN=Arsb PE=2 SV=3
PPP1R21	Q3TDD9-2	2	2	39293.5	40200.5	NA	21854.5	31871.5	34789	38588	53419.5	55926.5	77653.5	0.073488143	1.133782727	33305	52075.3	Up	0.644860463582871	Isoform 2 of Protein phosphatase 1 regulatory subunit 21 OS=Mus musculus GN=Ppp1r21
FGFR1OP2	Q9CRA9	2	2	11947	14377	16183.5	7411	13376.5	13830.5	12056.5	31642.5	24831	32310	0.073756399	1.132200294	12659	22934.1	Up	0.857330849719707	FGFR1 oncogene partner 2 homolog OS=Mus musculus GN=Fgfr1op2 PE=2 SV=1
LRRC8A	Q80WG5	1	1	2867	17225	NA	21645	15131	26064	29085	NA	20230	NA	0.074046759	1.130493944	14217	25126.333	Up	0.821583088386723	Leucine-rich repeat-containing protein 8A OS=Mus musculus GN=Lrrc8a PE=2 SV=1
SHB	Q6PD21	1	1	2437	2981	NA	2977	2359	4803	3144	5386	7882	NA	0.074123633	1.130043305	2688.5	5303.75	Up	0.980211301902631	SH2 domain-containing adapter protein B OS=Mus musculus GN=Shb PE=1 SV=2
WIPI2	Q80W47	2	2	3894	4564.5	1928	6190.5	7398.5	7737	8879.5	NA	6009	NA	0.074261667	1.129235307	4795.1	7541.8333	Up	0.653354366355602	WD repeat domain phosphoinositide-interacting protein 2 OS=Mus musculus GN=Wipi2 PE=1 SV=1
FKBP1B	Q9Z2I2	2	2	15200.5	13503	17551	8245	15992	13644.5	15753	23592	23244	20798.5	0.074450529	1.128132212	14098.3	19406.4	Up	0.461011304373961	Peptidyl-prolyl cis-trans isomerase FKBP1B OS=Mus musculus GN=Fkbp1b PE=1 SV=3
SHANK3	Q4ACU6-10	13	13	72426.15	58325.31	87478.77	33842.08	64762.08	33548.08	43783.23	56851.77	49743.77	15042.08	0.074710361	1.126619164	63366.878	39793.786	Down	-0.671185773394065	Isoform 9 of SH3 and multiple ankyrin repeat domains protein 3 OS=Mus musculus GN=Shank3
GRIN1	P35438	6	6	16189.5	19748.17	34507.5	19956.83	32986.5	28706.67	33856.33	50625.5	31329	67014.33	0.074740046	1.126446642	24677.7	42306.366	Up	0.777666828164092	Glutamate receptor ionotropic, NMDA 1 OS=Mus musculus GN=Grin1 PE=1 SV=1
STAM	P70297	5	5	31216.8	31587	38098.8	38085.8	36661.4	32724.4	32056.6	25629	34240.8	22632.8	0.076033223	1.118996598	35129.96	29456.72	Down	-0.25410513835963	Signal transducing adapter molecule 1 OS=Mus musculus GN=Stam PE=2 SV=3
MAGI2	Q9WVQ1-3	3	3	86255.67	98829	94198	67656	91018.33	83572.33	97945.33	161705.67	121341	162022.33	0.078087269	1.107419764	87591.4	125317.33	Up	0.516724826698591	Isoform 3 of Membrane-associated guanylate kinase, WW and PDZ domain-containing protein 2 OS=Mus musculus GN=Magi2
RNMT	Q9D0L8-3	2	2	5790.5	7284	606	10407	4679.5	8799	7518	10404	12148.5	NA	0.07823164	1.106617563	5753.4	9717.375	Up	0.756151868700902	Isoform 3 of mRNA cap guanine-N7 methyltransferase OS=Mus musculus GN=Rnmt
LYSMD2	Q9D7V2	2	2	8190.5	5900	8625.5	4123	5700	6213.5	7673	20152.5	12784.5	15841	0.078450593	1.105403767	6507.8	12532.9	Up	0.945478460513182	LysM and putative peptidoglycan-binding domain-containing protein 2 OS=Mus musculus GN=Lysmd2 PE=2 SV=2
SFXN1	Q99JR1	3	3	12864.67	15582.33	31641	22802.67	23628.67	24030.67	26478	43983	32413.33	61949.67	0.078646597	1.104320063	21303.868	37770.934	Up	0.826161064528464	Sideroflexin-1 OS=Mus musculus GN=Sfxn1 PE=1 SV=3
ENO1	P17182	63	63	108366.05	109946.87	100987.54	81807.75	101701.63	105620.38	115020.4	163334.56	114923.06	123974.71	0.078673888	1.104169387	100561.97	124574.62	Up	0.308925407699105	Alpha-enolase OS=Mus musculus GN=Eno1 PE=1 SV=3
PSMB6	Q60692	3	3	17779.67	16273.33	17350.67	26277	17622.67	26634.67	23820.33	30452.33	20759.67	20213	0.078781918	1.10357345	19060.668	24376	Up	0.354862724325706	Proteasome subunit beta type-6 OS=Mus musculus GN=Psmb6 PE=1 SV=3
RPL28	P41105	9	9	73779.33	81266.33	76616.78	69521.89	71124	69876.44	79328.44	92766.78	100277.11	96725.78	0.079395046	1.100206598	74461.666	87794.91	Up	0.2376394051533	60S ribosomal protein L28 OS=Mus musculus GN=Rpl28 PE=1 SV=2
TARSL2	Q8BLY2	1	1	40667	61824	32749	57043	91574	97301	99558	NA	67567	NA	0.07965709	1.098775561	56771.4	88142	Up	0.634665313336689	Probable threonine--tRNA ligase 2, cytoplasmic OS=Mus musculus GN=Tarsl2 PE=2 SV=1
TSC2	Q61037-7	2	2	3753.5	6669	9741	9470	9148	10357	10358	11110	9762.5	NA	0.080695646	1.093149898	7756.3	10396.875	Up	0.422709450572714	Isoform F of Tuberin OS=Mus musculus GN=Tsc2
AMPD2	Q9DBT5	6	6	3616.4	6955.2	7066.4	7834.6	6068.6	8255.6	8800	6520.2	9715.4	7459.8	0.080881053	1.092153202	6308.24	8150.2	Up	0.369597913275003	AMP deaminase 2 OS=Mus musculus GN=Ampd2 PE=1 SV=1
ATG3	Q9CPX6	3	3	51710	61162	64096.33	70729	71274	86955.67	90064	90289	70232.67	159503.67	0.081903777	1.086696069	63794.266	99409.002	Up	0.639949744699573	Ubiquitin-like-conjugating enzyme ATG3 OS=Mus musculus GN=Atg3 PE=1 SV=1
COX14	Q8BH51	1	1	12983	14396	17234	12680	12910	14974	18568	23021	18287	14009	0.082929663	1.081290097	14040.6	17771.8	Up	0.339985222814069	Cytochrome c oxidase assembly protein COX14 OS=Mus musculus GN=Cox14 PE=2 SV=1
MAN2B1	O09159	1	1	32291	34692	14406	31369	29852	35908	35196	43161	41509	30376	0.083061674	1.080599321	28522	37230	Up	0.38439046719459	Lysosomal alpha-mannosidase OS=Mus musculus GN=Man2b1 PE=2 SV=4
VIPAS39	Q8BGQ1	1	1	19749	20304	38479	38083	22619	40760	33569	42076	35018	NA	0.08315679	1.080102283	27846.8	37855.75	Up	0.443000900672002	Spermatogenesis-defective protein 39 homolog OS=Mus musculus GN=Vipas39 PE=1 SV=1
RCHY1	Q9CR50	1	1	4982	5413	10014	7660	8362	8737	8738	14036	15893	NA	0.083312532	1.079289669	7286.2	11851	Up	0.701770298913721	RING finger and CHY zinc finger domain-containing protein 1 OS=Mus musculus GN=Rchy1 PE=1 SV=1
RPL14	Q9CR57	10	10	174539.3	197015	181347.5	111579.6	184248.1	149769	213832.4	248519.6	219462	268565.2	0.083444265	1.078603505	169745.9	220029.64	Up	0.374321152630086	60S ribosomal protein L14 OS=Mus musculus GN=Rpl14 PE=1 SV=3
LYNX1	Q9WVC2-2	1	1	18221	21126	30919	31301	27396	25271	39794	78407	39776	46503	0.08358937	1.077848949	25792.6	45950.2	Up	0.833113930870958	Isoform 2 of Ly-6/neurotoxin-like protein 1 OS=Mus musculus GN=Lynx1
MRPL39	Q9JKF7	7	7	18509.14	21321	26758.43	20371.57	17836.43	22120.86	24102.86	37296	29140.57	24383.57	0.083667459	1.077443421	20959.314	27408.772	Up	0.387046194168038	39S ribosomal protein L39, mitochondrial OS=Mus musculus GN=Mrpl39 PE=2 SV=4
TMOD1	P49813	2	2	25444.5	35225	27317	28003.5	27672.5	37940	36538.5	75893.5	58933	NA	0.084412991	1.073590713	28732.5	52326.25	Up	0.864851344350849	Tropomodulin-1 OS=Mus musculus GN=Tmod1 PE=2 SV=2
CLASP1	Q80TV8	6	6	10882.8	12189.6	11273.4	13987.6	16043.8	14781.6	19429.4	23055.2	22897.4	11032.4	0.084871765	1.071236768	12875.44	18239.2	Up	0.502420716810439	CLIP-associating protein 1 OS=Mus musculus GN=Clasp1 PE=1 SV=2
ADAM11	Q9R1V4	2	2	11375.5	10843	14197	13050.5	11932	15478.5	13020.5	NA	15983	NA	0.084912735	1.07102717	12279.6	14827.333	Up	0.271995588644344	Disintegrin and metalloproteinase domain-containing protein 11 OS=Mus musculus GN=Adam11 PE=1 SV=2
NONO	Q99K48-2	4	4	181110.33	111395.33	89578	93462.33	109032	97035.33	85152	62183.67	84465.67	67177	0.085756729	1.066731794	116915.6	79202.734	Down	-0.56184527955245	Isoform 2 of Non-POU domain-containing octamer-binding protein OS=Mus musculus GN=Nono
RRBP1	Q99PL5-3	2	2	11506	13417	34512	16653	16050	19260	26802	41880	26361	32468	0.086563471	1.062665339	18427.6	29354.2	Up	0.671698751529923	Isoform RRp47 of Ribosome-binding protein 1 OS=Mus musculus GN=Rrbp1
RRBP1	Q99PL5-4	3	3	11506	13417	34512	16653	16050	19260	26802	41880	26361	32468	0.086563471	1.062665339	18427.6	29354.2	Up	0.671698751529923	Isoform RRp41 of Ribosome-binding protein 1 OS=Mus musculus GN=Rrbp1
RRBP1	Q99PL5-6	1	1	11506	13417	34512	16653	16050	19260	26802	41880	26361	32468	0.086563471	1.062665339	18427.6	29354.2	Up	0.671698751529923	Isoform RRp15a of Ribosome-binding protein 1 OS=Mus musculus GN=Rrbp1
PTRHD1	D3Z4S3	1	1	29580	26043	31393	30259	29850	37430	32876	60037	47432	NA	0.08667557	1.062103292	29425	44443.75	Up	0.594938135895205	Putative peptidyl-tRNA hydrolase PTRHD1 OS=Mus musculus GN=Ptrhd1 PE=3 SV=1
ATP5J	P97450	5	5	37983	39775.2	47421	36502	46648.2	43231.8	36270.2	18635.4	33868.8	14742	0.086714636	1.061907593	41665.88	29349.64	Down	-0.505523643235637	ATP synthase-coupling factor 6, mitochondrial OS=Mus musculus GN=Atp5j PE=1 SV=1
ACOT10	Q32MW3	3	3	20493.33	21553.67	16811.33	23115.33	27428.33	26206	26371.67	23377.33	27540.67	NA	0.087237273	1.059297918	21880.398	25873.918	Up	0.24185952442288	Acyl-coenzyme A thioesterase 10, mitochondrial OS=Mus musculus GN=Acot10 PE=2 SV=1

CHL1	P70232-2	1	1	41009	39877	21294	35265	29654	41327	42239	37016	46980	NA	0.087478294	1.058099698	33419.8	41890.5	Up	0.325920004492038	Isoform 2 of Neural cell adhesion molecule L1-like protein OS=Mus musculus GN=Chl1
GLCCI1	Q8K3I9-3	1	1	3203	4631	3583	3001	3475	4494	5157	NA	6739	NA	0.087819815	1.056407483	3578.6	5463.3333	Up	0.610386153876037	Isoform 3 of Glucocorticoid-induced transcript 1 protein OS=Mus musculus GN=Glcci1
AGFG1	Q8K2K6-2	5	5	18554.2	21759.2	16938.4	23664.4	25230.2	21968.6	26300.6	28454.2	25400.8	22992.8	0.088171511	1.054671717	21229.28	25023.4	Up	0.237222383408835	Isoform 2 of Arf-GAP domain and FG repeat-containing protein 1 OS=Mus musculus GN=Agfg1
RNF114	Q9ET26	1	1	3089	2935	2617	2697	3638	4052	5645	NA	7015	NA	0.089020202	1.050511424	2995.2	5570.6667	Up	0.89519765164991	RING finger protein 114 OS=Mus musculus GN=Rnf114 PE=2 SV=2
CLASP1	Q80TV8-2	8	8	9451.17	10807.67	9394.5	13271.67	14453.17	13702.83	18722.5	19212.67	20201.17	9193.67	0.089052688	1.050352966	11475.636	16206.568	Up	0.498004496888456	Isoform 2 of CLIP-associating protein 1 OS=Mus musculus GN=Clasp1
CDH13	Q9WTR5	10	10	10825	14951.89	25481.67	19242.44	15693.11	19130	20889.22	36467.89	24558.44	49656.11	0.089328301	1.049010924	17238.822	30140.332	Up	0.806034116035546	Cadherin-13 OS=Mus musculus GN=Cdh13 PE=1 SV=2
MRPL35	Q9CQL6	2	2	43531.5	43948	6176.5	63744.5	64905	114670.5	90925.5	143129	39863.5	NA	0.089972797	1.045888781	44461.1	97147.125	Up	1.12762765877714	39S ribosomal protein L35, mitochondrial OS=Mus musculus GN=Mrpl35 PE=2 SV=1
PRODH	Q9WU79	1	1	4423	4030	2696	5155	3488	6381	4868	NA	7912	NA	0.090052713	1.045503199	3958.4	6387	Up	0.690221045084443	Proline dehydrogenase 1, mitochondrial OS=Mus musculus GN=Prodh PE=1 SV=2
RAB11FIP2	G3XA57	6	6	56930.6	64845.4	68028	57927	69044.8	74222.4	76640	102303	91023	57525.6	0.09011243	1.0452153	63355.16	80342.8	Up	0.342706615804073	Rab11 family-interacting protein 2 OS=Mus musculus GN=Rab11fip2 PE=1 SV=1
GART	Q64737	2	2	14500	21869	17130.5	10534.5	15360.5	17462.5	23131	42161	25891	63675.5	0.090135246	1.04510535	15878.9	34464.2	Up	1.1179875529317	Trifunctional purine biosynthetic protein adenosine-3 OS=Mus musculus GN=Gart PE=2 SV=3
UBTD2	Q6PGH0	2	2	41492	49427	38952	74809.5	48308	88407	59334.5	NA	73092	NA	0.090218422	1.044704773	50597.7	73611.167	Up	0.540852830452445	Ubiquitin domain-containing protein 2 OS=Mus musculus GN=Ubtd2 PE=2 SV=1
PTEN	O08586	3	3	2316	3029	1463	4835	2546	4202	3689	NA	4375	NA	0.09047996	1.043447598	2837.8	4088.6667	Up	0.526857534282394	Phosphatidylinositol 3,4,5-trisphosphate 3-phosphatase and dual-specificity protein phosphatase PTEN OS=Mus musculus GN=Pten PE=1 SV=1
PCP4	P63054	7	7	24608.29	27483.86	34198.29	34858	28014	28847.43	25963.71	17187.29	26783.43	10350.57	0.091258113	1.039728514	29832.488	21826.486	Down	-0.450804422661459	Purkinje cell protein 4 OS=Mus musculus GN=Pcp4 PE=2 SV=2
CLASP2	Q8BRT1-5	2	2	9256	11722.5	17942	17173	16739.5	18893	18723.5	21956	13502.5	27581	0.092042865	1.036009871	14566.6	20131.2	Up	0.466768996114628	Isoform 2 of CLIP-associating protein 2 OS=Mus musculus GN=Clasp2
LXN	P70202	1	1	23343	19419	31745	13340	20742	19929	33907	43373	38420	NA	0.092112467	1.035681586	21717.8	33907.25	Up	0.642715814939025	Latexin OS=Mus musculus GN=Lxn PE=1 SV=2
DMWD	Q08274	2	2	13030	19747.5	4384.5	25535.5	23854	27188	25179	NA	25212.5	NA	0.092161157	1.035452084	17310.3	25859.833	Up	0.579082249138169	Dystrophia myotonica WD repeat-containing protein OS=Mus musculus GN=Dmwd PE=2 SV=3
BORCS6	Q9D6W8	2	2	2692.5	2409	1246	3437.5	3339	3969.5	3269.5	NA	6368	3632	0.092203984	1.035250314	2624.8	4309.75	Up	0.715396684911296	Uncharacterized protein C17orf59 homolog OS=Mus musculus PE=1 SV=1
PMM1	O35621	7	7	62519.17	52357.33	68050.67	44793.83	64159.17	69827.17	66537	148274.5	81556.17	88994.17	0.092548103	1.033632479	58376.034	91037.802	Up	0.641089526757599	Phosphomannomutase 1 OS=Mus musculus GN=Pmm1 PE=1 SV=1
MAP7	O88735	1	1	121926	88855	154683	86117	114081	150360	109327	233010	153216	151821	0.092896888	1.031998835	113132.4	159546.8	Up	0.495967510956982	Ensconsin OS=Mus musculus GN=Map7 PE=1 SV=1
PLXND1	Q3UH93	5	5	3269.4	6928	7000.2	10563.6	8352.4	11058.2	10572.6	12785.8	7506.4	8658.8	0.093080872	1.031139557	7222.72	10116.36	Up	0.486076134407257	Plexin-D1 OS=Mus musculus GN=Plxnd1 PE=1 SV=1
ANK3	G5E8K5-2	23	23	22926.83	28839.78	33730	34533.65	28138.35	33425.91	34776.26	53492.39	29611.17	48944.22	0.093123736	1.030939609	29633.722	40049.99	Up	0.434562042013734	Isoform 2 of Ankyrin-3 OS=Mus musculus GN=Ank3
ISCA2	Q9DCB8	10	10	24307.8	31712.4	27691	22962.6	28450.1	29344	27449.4	31942.8	34678.8	30675.7	0.093151498	1.030810155	27024.78	30818.14	Up	0.18949691847741	Iron-sulfur cluster assembly 2 homolog, mitochondrial OS=Mus musculus GN=Isca2 PE=2 SV=2
ANK3	G5E8K5	21	21	25027.43	31474.33	36876.05	37702.33	30671.71	36356.24	37954.52	58348.33	32375.81	53605.57	0.093693249	1.028291702	32350.37	43728.094	Up	0.434778253454689	Ankyrin-3 OS=Mus musculus GN=Ank3 PE=1 SV=1
VWA5A	Q99KC8	1	1	4223	5166	852	4552	4700	5891	5564	NA	8982	NA	0.094386553	1.025089873	3898.6	6812.3333	Up	0.805192888583227	von Willebrand factor A domain-containing protein 5A OS=Mus musculus GN=Vwa5a PE=1 SV=2
PSPC1	Q8R326	4	4	71562.25	60057.25	48629	42520.75	62785.5	61670	52872	26142.25	31844	27426	0.094684228	1.023722357	57110.95	39990.85	Down	-0.514097437240908	Paraspeckle component 1 OS=Mus musculus GN=Pspc1 PE=1 SV=1
AAK1	Q6PIU9	2	2	900	552.5	NA	3133.5	2247.5	3138.5	2884.5	NA	3380	NA	0.094813973	1.023127655	1708.375	3134.3333	Up	0.875533926046921	Uncharacterized protein FLJ45252 homolog OS=Mus musculus PE=1 SV=2
ACP1	Q9D358	7	7	24995.5	18520	31513.17	12939	21963.83	19586.83	25209.5	41787.67	35486.83	50606.83	0.094980836	1.022364014	21986.3	34535.532	Up	0.651476609353016	Low molecular weight phosphotyrosine protein phosphatase OS=Mus musculus GN=Acp1 PE=1 SV=3
PPP4R4	Q8C0Y0	1	1	41933	49844	44782	41846	60875	63899	69204	128921	73248	NA	0.095252084	1.021125511	47856	83818	Up	0.808560281841054	Serine/threonine-protein phosphatase 4 regulatory subunit 4 OS=Mus musculus GN=Ppp4r4 PE=2 SV=1
CAT	P24270	6	6	21800.33	32442.17	29551.67	35657.67	26162.17	33037.83	32728.67	45419	29851.5	50289.83	0.095434372	1.020295178	29122.802	38265.366	Up	0.393890030174019	Catalase OS=Mus musculus GN=Cat PE=1 SV=4
NAV3	Q80TN7	3	3	17816	22263	27964	14417	19739	27711	26335	68900	50447	23834	0.096002246	1.017718606	20439.8	39445.4	Up	0.948475987617759	Neuron navigator 3 OS=Mus musculus GN=Nav3 PE=1 SV=2
DEK	Q7TNV0	1	1	64342	46641	31257	48932	62209	45627	39023	12551	32760	NA	0.09631108	1.016323748	50676.2	32490.25	Down	-0.641301502067199	Protein DEK OS=Mus musculus GN=Dek PE=1 SV=1
OSCP1	Q8BHW2	1	1	29070	45963	27947	37052	59170	54994	53897	NA	48497	NA	0.096917538	1.013597626	39840.4	52462.667	Up	0.397059009138669	Protein OSCP1 OS=Mus musculus GN=Oscp1 PE=2 SV=1
NHP2L1	Q9D0T1	1	1	313152	204248	161926	198085	223649	207465	213508	62494	147535	108689	0.097157044	1.012525706	220212	147938.2	Down	-0.573898460359101	NHP2-like protein 1 OS=Mus musculus GN=Nhp2l1 PE=1 SV=4
PAK3	Q61036	6	6	33037.5	40003.5	43383.83	48723.83	38273.5	44418.67	46609.5	58145.67	39812.83	60893	0.097351708	1.011656423	40684.432	49975.934	Up	0.296756680534164	Serine/threonine-protein kinase PAK 3 OS=Mus musculus GN=Pak3 PE=1 SV=2
FAHD1	Q8R0F8	3	3	10461.67	11242.33	6287.33	15809.33	10440	13009.33	13350.67	NA	14131.33	16228	0.097377407	1.011541795	10848.132	14179.833	Up	0.38639385237116	Acylpyruvase FAHD1, mitochondrial OS=Mus musculus GN=Fahd1 PE=1 SV=2
PTGR2	Q8VDQ1-2	1	1	13723	23256	10107	17030	18507	22063	27263	18949	39009	NA	0.097664309	1.010264119	16524.6	26821	Up	0.698747678204528	Isoform 2 of Prostaglandin reductase 2 OS=Mus musculus GN=Ptgr2
ANK2	Q8C8R3-3	34	34	19189.06	24526.82	23586.36	28951.64	27801.88	27986.12	32462.03	24994.88	27623.18	31923.12	0.09779402	1.009687702	24811.152	28997.866	Up	0.224958011702962	Isoform 3 of Ankyrin-2 OS=Mus musculus GN=Ank2
RPS4X	P62702	18	18	79712.28	92650.83	83519.5	102978.83	83548.61	87831.28	95862.89	104476.17	99252.28	104266.5	0.098669538	1.005816906	88482.01	98337.824	Up	0.152362272939023	40S ribosomal protein S4, X isoform OS=Mus musculus GN=Rps4x PE=2 SV=2
HIST1H1T	Q07133	5	5	493508.8	256429.8	206467.2	162368.2	331856.4	212019.6	176863.4	125646	157283.2	157843.2	0.099112303	1.00387243	290126.08	165931.08	Down	-0.806095850014742	Histone H1t OS=Mus musculus GN=Hist1h1t PE=1 SV=4
PDCD10	Q8VE70	2	2	37913	39569.5	27547	44376.5	42741.5	45340	43786.5	NA	45067.5	NA	0.099165445	1.003639635	38429.5	44731.333	Up	0.219071556397262	Programmed cell death protein 10 OS=Mus musculus GN=Pdcd10 PE=1 SV=1
SRRM2	Q8BTI8-3	2	2	9051	15218.5	6351	14955.5	17247	22855	19786	12285.5	18549	NA	0.099372344	1.002734466	12564.6	18368.875	Up	0.547898528365715	Isoform 3 of Serine/arginine repetitive matrix protein 2 OS=Mus musculus GN=Srrm2
TRIM32	Q8CH72	2	2	52051	61006	51286	74618	82930	82536	85305	80129	69204	139713	0.100401358	0.998260413	64378.2	91377.4	Up	0.505265153564707	E3 ubiquitin-protein ligase TRIM32 OS=Mus musculus GN=Trim32 PE=1 SV=2
MTCH1	Q791T5	1	1	61842	168421	47741	295601	303326	377588	393611	NA	219819	NA	0.100428896	0.99814131	175386.2	330339.33	Up	0.913413524848866	Mitochondrial carrier homolog 1 OS=Mus musculus GN=Mtch1 PE=2 SV=1
FRRS1L	B1AXV0	3	3	48873	43258.67	61860	52123	47664.33	53935.67	66774.33	59306	50391.33	76339.33	0.100632998	0.997259588	50755.8	61349.332	Up	0.273474942930425	DOMON domain-containing protein FRRS1L OS=Mus musculus GN=Frrs1l PE=1 SV=1
GLOD4	Q9CPV4-3	5	5	59663.4	62066.4	72277	75875.2	67418.6	65338	66458.8	23618.4	52922.4	45101	0.10097783	0.995773968	67460.12	50687.72	Down	-0.412398613590403	Isoform 3 of Glyoxalase domain-containing protein 4 OS=Mus musculus GN=Glod4
MRPL46	Q9EQI8	5	5	17861.6	17822.8	19467.2	14011.2	14667	19061	18159.8	27667.6	20566.4	38133	0.101057861	0.9954299	16765.96	24717.56	Up	0.560001241685093	39S ribosomal protein L46, mitochondrial OS=Mus musculus GN=Mrpl46 PE=1 SV=1
SEPT9	Q80UG5-2	25	25	38682.6	40655.4	40534.2	37355.72	40042.84	43167.16	45740.68	57909.84	51942.04	36860.32	0.10134864	0.994182075	39454.152	47124.008	Up	0.256285116969168	Isoform 2 of Septin-9 OS=Mus musculus GN=Sept9
DDX3X	Q62167	7	7	23753.29	24513.71	32637.29	19928.43	26110.86	25973.29	25147.57	47997	33759.43	42694.57	0.101498301	0.993541225	25388.716	35114.372	Up	0.467874199166641	ATP-dependent RNA helicase DDX3X OS=Mus musculus GN=Ddx3x PE=1 SV=3
IK	Q9Z1M8	1	1	5439	5855	6585	4750	7660	6737	8125	7360	6614	NA	0.101699423	0.992681512	6057.8	7209	Up	0.25100520125889	Protein Red OS=Mus musculus GN=Ik PE=1 SV=2
MRPL50	Q8VDT9	2	2	52920	73404.5	36796.5	58146.5	67610	72050.5	76222.5	104374.5	97629.5	49115	0.101976866	0.991498341	57775.5	79878.4	Up	0.467347595145714	39S ribosomal protein L50, mitochondrial OS=Mus musculus GN=Mrpl50 PE=2 SV=2
NDRG3	Q9QYF9	4	4	4846.5	8806.25	11685.5	6002	7975	8393.5	10400.75	13267	9114	11984	0.102295177	0.990144842	7863.05	10631.85	Up	0.43523172211465	Protein NDRG3 OS=Mus musculus GN=Ndrg3 PE=1 SV=1
MRPL45	Q9D0Q7	3	3	83232.33	76477.33	96265.67	65381.67	72791.67	78499.67	76328.67	159145.67	113088.67	139658	0.103475386	0.985162944	78829.734	113344.14	Up	0.523897941901901	39S ribosomal protein L45, mitochondrial OS=Mus musculus GN=Mrpl45 PE=2 SV=1
NONO	Q99K48	9	9	85239.38	54627.62	42549.75	46720.62	52980	48870	42052.38	33865.38	39062.75	39246.5	0.104176734	0.98222926	56423.474	40619.402	Down	-0.474126495170585	Non-POU domain-containing octamer-binding protein OS=Mus musculus GN=Nono PE=1 SV=3
RNF34	Q99KR6	4	4	4791	4610	1173	5577	5880	5952.33	6173.33	NA	6377.67	NA	0.104446381	0.981106605	4406.2	6167.7767	Up	0.485215545347947	E3 ubiquitin-protein ligase RNF34 OS=Mus musculus GN=Rnf34 PE=1 SV=1
PALM3	A2TJV2	2	2	1258	1098	NA	1578	1044	1895	2524	NA	1549	NA	0.104447135	0.981103469	1244.5	1989.3333	Up	0.676718804553046	Paralemmin-3 OS=Mus musculus GN=Palm3 PE=2 SV=1
ACP1	Q9D358-2	5	5	29589	21690.6	36686.2	14539.4	25534.8	22242.6	29022	50145.2	40928	60728.2	0.104868514	0.979354886	25608	40613.2	Up	0.665354122740849	Isoform 2 of Low molecular weight phosphotyrosine protein phosphatase OS=Mus musculus GN=Acp1
GM996	A2AJA9	16	16	30876.88	34392.94	33201	28205.94	37176.19	34857.31	38696.19	51584.06	40128	33078	0.104896756	0.979237943	32770.59	39668.712	Up	0.275599908181309	Uncharacterized protein C9orf172 homolog OS=Mus musculus GN=Gm996 PE=1 SV=1
ALDH1B1	Q9CZS1	7	7	39750.71	49931.29	51775	44970	53738.14	51609.86	46649.86	30805	32154.14	28580	0.105003645	0.978795624	48033.028	37959.772	Down	-0.339555431833464	Aldehyde dehydrogenase X, mitochondrial OS=Mus musculus GN=Aldh1b1 PE=1 SV=1
NFX1	B1AY10	3	3	1375.67	1745	504	2988	2190.67	3922.67	1978.67	2361	4616.33	NA	0.105455464	0.976930914	1760.668	3219.6675	Up	0.870788813382949	Transcriptional repressor NF-X1 OS=Mus musculus GN=Nfx1 PE=2 SV=1
AGFG2	Q80WC7	4	4	34911	37651	27263	24304.5	35689.25	27669.25	30898.5	18444.5	29891.5	17517.75	0.105521451	0.976659246	31963.75	24884.3	Down	-0.361200871452373	Arf-GAP domain and FG repeat-containing protein 2 OS=Mus musculus GN=Agfg2 PE=1 SV=1
TPD52	Q62393	5	5	20946.4	25674.2	27788.8	19046.6	21679.8	23887.8	22945.6	43291.8	31170.4	32577.6	0.105632718	0.976201544	23027.16	30774.64	Up	0.418405489502023	Tumor protein D52 OS=Mus musculus GN=Tpd52 PE=1 SV=2
CORT	P56469	1	1	8745	4718	10276	7681	6143	11539	8585	26244	20175	NA	0.10615776	0.974048254	7512.6	16635.75	Up	1.14690271488062	Cortistatin OS=Mus musculus GN=Cort PE=2 SV=1
RAN	P62827	6	6	101014	115723.5	139880	125695.67	111028.67	133346	129256.33	241789.67	127923.67	218550.17	0.106307357	0.973436679	118668.37	170173.17	Up	0.520068154120587	GTP-binding nuclear protein Ran OS=Mus musculus GN=Ran PE=1 SV=3
TBCE	Q8CIV8	1	1	32432	38637	28176	19036	43089	30849	42755	51452	52852	92867	0.106575147	0.97234406	32274	54155	Up	0.74672214976106	Tubulin-specific chaperone E OS=Mus musculus GN=Tbce PE=1 SV=1
SNTA1	Q61234	1	1	3793	4034	NA	5855	7342	7255	7050	NA	NA	NA	0.106628353	0.972127297	5256	7152.5	Up	0.444482316888216	Alpha-1-syntrophin OS=Mus musculus GN=Snta1 PE=1 SV=1
RNF11	Q9QYK7	3	3	1901.67	2948.67	NA	6264.67	6077.33	7327	6813.33	NA	6161	NA	0.107895795	0.966995481	4298.085	6767.11	Up	0.65484582573518	RING finger protein 11 OS=Mus musculus GN=Rnf11 PE=1 SV=1
PJA1	O55176	2	2	3990.5	5502.5	4035.5	5263	6269	6208	6034.5	NA	8317.5	NA	0.108164168	0.965916586	5012.1	6853.3333	Up	0.451390659508122	E3 ubiquitin-protein ligase Praja-1 OS=Mus musculus GN=Pja1 PE=1 SV=3
MAP6	Q7TSJ2	74	74	56163.49	52521.57	68060.57	43911.27	54571.35	53350.92	55244.34	70606.05	81328.42	84517.16	0.108434081	0.964834195	55045.65	69009.378	Up	0.326163870781116	Microtubule-associated protein 6 OS=Mus musculus GN=Map6 PE=1 SV=2
TRMU	Q9DAT5	1	1	6784	7471	5096	6716	5983	8061	9255	NA	12581	NA	0.108823268	0.963278237	6410	9965.6667	Up	0.636641962472486	Mitochondrial tRNA-specific 2-thiouridylase 1 OS=Mus musculus GN=Trmu PE=2 SV=1
GMPS	Q3THK7	2	2	20670.5	8377	NA	17081.5	18745.5	19995.5	22745	NA	30099	NA	0.109217567	0.961707504	16218.625	24279.833	Up	0.58210700399038	GMP synthase [glutamine-hydrolyzing] OS=Mus musculus GN=Gmps PE=1 SV=2
GRIP1	Q925T6-3	1	1	8616	6800	5003	5309	6618	9069	5997	11073	9665	19169	0.109904114	0.95898605	6469.2	10994.6	Up	0.765135897227806	Isoform 3 of Glutamate receptor-interacting protein 1 OS=Mus musculus GN=Grip1
GPSM1	Q6IR34-4	1	1	8794	12914	NA	12054	9717	9012	8422	NA	NA	NA	0.110224046	0.957723653	10869.75	8717	Down	-0.318415144646285	Isoform 4 of G-protein-signaling modulator 1 OS=Mus musculus GN=Gpsm1
GIGYF2	Q6Y7W8	1	1	8538	3128	9447	15784	13067	12848	15132	NA	21039	NA	0.11029003	0.957463746	9992.8	16339.667	Up	0.709417666995599	PERQ amino acid-rich with GYF domain-containing protein 2 OS=Mus musculus GN=Gigyf2 PE=1 SV=2
HIST2H2AB	Q64522	2	2	1367939.5	734959.5	902591.5	236997	932025	560117	510691.5	314910.5	465601	NA	0.111673486	0.952049925	834902.5	462830	Down	-0.851125348375833	Histone H2A type 2-B OS=Mus musculus GN=Hist2h2ab PE=1 SV=3
NDUFB3	Q9CQZ6	1	1	12815	16039	14959	23986	15166	18648	23341	NA	28023	NA	0.111722206	0.951860498	16593	23337.333	Up	0.492064971402321	NADH dehydrogenase [ubiquinone] 1 beta subcomplex subunit 3 OS=Mus musculus GN=Ndufb3 PE=1 SV=1
MATK	P41242-3	4	4	4039.25	5268	3412.5	6925.75	5272.25	7816.5	8177.25	NA	5415.75	NA	0.112093534	0.950419437	4983.55	7136.5	Up	0.51804289431475	Isoform 3 of Megakaryocyte-associated tyrosine-protein kinase OS=Mus musculus GN=Matk
KIF21B	Q9QXL1	5	5	18805.8	28169.2	20178.6	22094.8	23857	21873.8	36938.4	31002.2	28296.6	NA	0.112337172	0.949476513	22621.08	29527.75	Up	0.384403618334113	Kinesin-like protein KIF21B OS=Mus musculus GN=Kif21b PE=1 SV=2
CCSAP	Q8QZT2	7	7	35911.29	38730	25296	24019.43	37418.71	37333.57	40169.43	34637.86	60116.14	39173.57	0.112915771	0.947245395	32275.086	42286.114	Up	0.389763046252726	Centriole, cilia and spindle-associated protein OS=Mus musculus GN=Ccsap PE=2 SV=1
MAP6	Q7TSJ2-2	67	67	57157.88	53571.15	71784.28	41955.96	55968.84	54225.21	56421.72	73512.9	85014.94	88610.24	0.113005206	0.94690155	56087.622	71557.002	Up	0.351410527530841	Isoform 2 of Microtubule-associated protein 6 OS=Mus musculus GN=Map6
GNG3	P63216	7	7	26990	31481.29	45881.71	27431.57	35711	38710.57	42977	50620.29	36695.86	80131.14	0.11331379	0.945717236	33499.114	49826.972	Up	0.572803964663997	Guanine nucleotide-binding protein G(I)/G(S)/G(O) subunit gamma-3 OS=Mus musculus GN=Gng3 PE=1 SV=1
HIST2H2AC	Q64523	5	5	573386	311414	373418.4	107385.6	389557.8	237636.2	218851.8	138466.2	201714.6	NA	0.113419736	0.945311369	351032.36	199167.2	Down	-0.817623956188375	Histone H2A type 2-C OS=Mus musculus GN=Hist2h2ac PE=1 SV=3
CCDC43	Q9CR29-2	1	1	30068	45685	39744	26274	29555	39792	35354	88363	47799	55999	0.113572251	0.944727767	34265.2	53461.4	Up	0.641753510317932	Isoform 2 of Coiled-coil domain-containing protein 43 OS=Mus musculus GN=Ccdc43
SDHA	Q8K2B3	49	49	70049.65	85744.45	84518.84	90933.8	82415.1	90535.33	90350.24	120476.31	84119.55	144032.29	0.113678031	0.944323456	82732.368	105902.74	Up	0.356216189589878	Succinate dehydrogenase [ubiquinone] flavoprotein subunit, mitochondrial OS=Mus musculus GN=Sdha PE=1 SV=1
HSPA8	P63017	169	169	111804.06	121385.92	101570.57	127210	114518.25	111946.58	119639.11	90925.36	97743.73	79374.06	0.113739988	0.944086823	115297.76	99925.768	Down	-0.206435823713951	Heat shock cognate 71 kDa protein OS=Mus musculus GN=Hspa8 PE=1 SV=1
FASN	P19096	2	2	10679.5	15311.5	17900	22902.5	13075.5	16921.5	15401.5	37020.5	22248.5	36868	0.113920531	0.943397999	15973.8	25692	Up	0.685611645913911	Fatty acid synthase OS=Mus musculus GN=Fasn PE=1 SV=2
SOGA3	Q6NZL0	15	15	22180.33	52064.07	55082.4	88817.2	52849.6	74455.4	92035.6	83499.47	44629.4	118281.4	0.114060417	0.942865046	54198.72	82580.254	Up	0.60753807580626	Protein SOGA3 OS=Mus musculus GN=Soga3 PE=2 SV=2
HBB-B1	P02088	8	8	83544.75	52426.88	39273.75	52763.62	82525.12	47852.88	49710.5	34468	49582.12	38902.5	0.11445862	0.941351494	62106.824	44103.2	Down	-0.493868456228525	Hemoglobin subunit beta-1 OS=Mus musculus GN=Hbb-b1 PE=1 SV=2
ADK	P55264	4	4	68034	94853.25	140246.5	70363.75	129879.75	111521.5	125839.75	157391.75	117067.25	149897	0.114739932	0.940285412	100675.45	132343.45	Up	0.394574873236372	Adenosine kinase OS=Mus musculus GN=Adk PE=1 SV=2
H2AFV	Q3THW5	3	3	935144.67	503063.33	613226	169188	636086.67	385425.33	353042	228437.67	324420.33	NA	0.114965042	0.939434196	571341.73	322831.33	Down	-0.823573309648815	Histone H2A.V OS=Mus musculus GN=H2afv PE=1 SV=3
APBA1	B2RUJ5	7	7	7378.4	11914.6	2514.8	11967.4	12242.2	13903.6	15216.6	NA	11016.2	NA	0.115772933	0.936392963	9203.48	13378.8	Up	0.539697342309372	Amyloid beta A4 precursor protein-binding family A member 1 OS=Mus musculus GN=Apba1 PE=1 SV=2
MAP1S	Q8C052	1	1	99308	95003	84379	123891	102729	125147	134040	106488	186319	NA	0.115981722	0.935610448	101062	137998.5	Up	0.449411949406905	Microtubule-associated protein 1S OS=Mus musculus GN=Map1s PE=1 SV=2
TNRC6B	Q8BKI2	1	1	4933	3642	3528	4303	3639	5487	4255	10869	8296	NA	0.116251874	0.934600036	4009	7226.75	Up	0.850104569516941	Trinucleotide repeat-containing gene 6B protein OS=Mus musculus GN=Tnrc6b PE=2 SV=2
ACYP1	P56376	4	4	24708.25	26971	38227.5	23693	24303.5	25686.75	27282	44436	42372	55571.25	0.116900851	0.932182329	27580.65	39069.6	Up	0.502390027481273	Acylphosphatase-1 OS=Mus musculus GN=Acyp1 PE=2 SV=2
ACOT9	Q9R0X4	4	4	17966.75	19576.25	16138.75	25834.5	23238	25229.5	23773.75	23069.75	24223.5	NA	0.116956056	0.931977286	20550.85	24074.125	Up	0.228285296526278	Acyl-coenzyme A thioesterase 9, mitochondrial OS=Mus musculus GN=Acot9 PE=1 SV=1
MPP3	O88910	2	2	8477.5	6450	NA	6026	8305.5	8575	8138	NA	9906	NA	0.117036286	0.931679467	7314.75	8873	Up	0.278613411020579	MAGUK p55 subfamily member 3 OS=Mus musculus GN=Mpp3 PE=1 SV=2
UFD1L	P70362	1	1	41376	43618	52287	32170	38969	49693	52759	NA	70939	NA	0.117318295	0.930634255	41684	57797	Up	0.471500885025509	Ubiquitin fusion degradation protein 1 homolog OS=Mus musculus GN=Ufd1l PE=1 SV=2
OXCT1	Q9D0K2	86	86	82975.38	81263.15	87459.19	81150.74	75531.88	80407.21	85192.66	98141.32	89381.31	86576.49	0.117754644	0.929021956	81676.068	87939.798	Up	0.106602804113384	Succinyl-CoA:3-ketoacid coenzyme A transferase 1, mitochondrial OS=Mus musculus GN=Oxct1 PE=1 SV=1
CAMSAP2	Q8C1B1	2	2	9761.5	9879	22489.5	8896	12897.5	14789.5	14904	31614	29582	NA	0.117773147	0.92895372	12784.7	22722.375	Up	0.829695329505679	Calmodulin-regulated spectrin-associated protein 2 OS=Mus musculus GN=Camsap2 PE=1 SV=3
PLXNB2	B2RXS4	7	7	8331.14	7445.86	5773.29	9010.86	7538.71	8806.14	11816	NA	14450.29	NA	0.117892799	0.928512721	7619.972	11690.81	Up	0.617517289153632	Plexin-B2 OS=Mus musculus GN=Plxnb2 PE=1 SV=1
VSTM2L	Q6PDS0	1	1	35651	39628	NA	26652	30949	38125	38076	76914	79336	NA	0.117966165	0.928242541	33220	58112.75	Up	0.806802653905287	V-set and transmembrane domain-containing protein 2-like protein OS=Mus musculus GN=Vstm2l PE=2 SV=1
CNPY3	Q9DAU1	5	5	48945	51076.4	40951	30824.2	43735.8	36657.4	40861.8	24124.2	42446.6	15786.8	0.118812278	0.925138679	43106.48	31975.36	Down	-0.430944157339927	Protein canopy homolog 3 OS=Mus musculus GN=Cnpy3 PE=1 SV=1
NRAS	P08556	21	21	44660.32	51097.79	54490.42	56262.26	49946.42	50347.79	54927.47	58187.16	55468.11	60823.05	0.119376561	0.923080938	51291.442	55950.716	Up	0.125438461854405	GTPase NRas OS=Mus musculus GN=Nras PE=2 SV=1

ATXN2	O70305	3	3	15618.67	18422.33	19447.33	16465.33	14985.33	16753.33	17562	24084.67	22982.67	33842	0.119630059	0.922159684	16987.798	23044.934	Up	0.439950776870055	Ataxin-2 OS=Mus musculus GN=Atxn2 PE=1 SV=1
RAB11A	P62492	5	5	83607.8	95021.8	97392.8	143533.2	95571	112857	141155.8	123860.2	121566.8	120546.4	0.119804992	0.921525087	103025.32	123997.24	Up	0.267309063972312	Ras-related protein Rab-11A OS=Mus musculus GN=Rab11a PE=1 SV=3
THSD7A	Q69ZU6	8	8	8033.62	10583.5	9088.5	14383.88	11830.25	14655.88	14931.5	17966.38	15924.62	8096.5	0.120255595	0.919894709	10783.95	14314.976	Up	0.408639539002561	Thrombospondin type-1 domain-containing protein 7A OS=Mus musculus GN=Thsd7a PE=2 SV=2
SFXN5	Q925N0	2	2	38692	38560	23618	39555	46500	43342	50814	39905	65380	NA	0.120544147	0.918853871	37385	49860.25	Up	0.415430583498377	Sideroflexin-5 OS=Mus musculus GN=Sfxn5 PE=1 SV=2
EML1	Q05BC3-3	1	1	21319	NA	64690	40006	47165	70582	39342	101991	76992	NA	0.120884606	0.917629002	43295	72226.75	Up	0.738332832260653	Isoform 3 of Echinoderm microtubule-associated protein-like 1 OS=Mus musculus GN=Eml1
FAM21	Q6PGL7	4	4	37195.67	44305.67	32230.33	20624	29992.33	36602.67	40622	33363	57343.67	74370	0.121095134	0.916873308	32869.6	48460.268	Up	0.560048483415753	WASH complex subunit FAM21 OS=Mus musculus GN=Fam21 PE=1 SV=1
TARS2	Q3UQ84	2	2	2201.5	3242	6122.5	10820	7019.5	10729	10394	NA	6998.5	NA	0.121121582	0.916778467	5881.1	9373.8333	Up	0.672553122191785	Threonine--tRNA ligase, mitochondrial OS=Mus musculus GN=Tars2 PE=2 SV=1
MLF2	Q99KX1	5	5	21732	15359.6	19406.2	14363.6	20141.6	19207.8	14188.2	9324	17109.4	6171.6	0.121180223	0.916568254	18200.6	13200.2	Down	-0.463426222581081	Myeloid leukemia factor 2 OS=Mus musculus GN=Mlf2 PE=1 SV=1
HIST2H2AA1	Q6GSS7	7	7	439060	232573	275504.29	85424.57	295792.86	181403.86	168625.86	108980.29	156831.71	NA	0.121863287	0.914127112	265670.94	153960.43	Down	-0.787080845634127	Histone H2A type 2-A OS=Mus musculus GN=Hist2h2aa1 PE=1 SV=3
GAD1	P48318	7	7	20197	25985.5	46898	18498.17	29193.67	28033	31588.5	65238.5	38953.17	81245.5	0.122171425	0.91303036	28154.468	49011.734	Up	0.799763298834659	Glutamate decarboxylase 1 OS=Mus musculus GN=Gad1 PE=2 SV=2
CPNE1	Q8C166	1	1	19277	21830	11975	24384	19930	35507	22193	NA	28153	NA	0.122289065	0.912612376	19479.2	28617.667	Up	0.554971619181556	Copine-1 OS=Mus musculus GN=Cpne1 PE=1 SV=1
HDGFRP2	Q3UMU9-3	1	1	12097	12736	15032	14167	12918	16267	12370	32764	15985	27613	0.122759528	0.91094479	13390	20999.8	Up	0.649219627210975	Isoform 3 of Hepatoma-derived growth factor-related protein 2 OS=Mus musculus GN=Hdgfrp2
ARPC4	P59999	4	4	32154.5	38024	49787.75	35342	33476	38626.75	43746	67049.5	48613	100673.75	0.124628027	0.904384279	37756.85	59741.8	Up	0.662002298475474	Actin-related protein 2/3 complex subunit 4 OS=Mus musculus GN=Arpc4 PE=1 SV=3
PRKAR2A	P12367	11	11	47279.73	50789.09	52910	70214.36	53210.73	55193.82	48620.36	36424.45	47427	43278.45	0.125320482	0.901977944	54880.782	46188.816	Down	-0.248757473972455	cAMP-dependent protein kinase type II-alpha regulatory subunit OS=Mus musculus GN=Prkar2a PE=1 SV=2
STRAP	Q9Z1Z2	11	11	19699.91	23911.36	19352.36	23547.45	22590.45	24876.91	23903.82	37548.27	27134.64	22205.09	0.125357822	0.901848561	21820.306	27133.746	Up	0.314416901697479	Serine-threonine kinase receptor-associated protein OS=Mus musculus GN=Strap PE=1 SV=2
PIP5K1C	O70161-3	5	5	29522.2	23222.8	33751.6	25100.8	25874.8	30673.2	35110.6	23987.2	36933.6	38901.4	0.125898113	0.899980778	27494.44	33121.2	Up	0.268615041009766	Isoform 3 of Phosphatidylinositol 4-phosphate 5-kinase type-1 gamma OS=Mus musculus GN=Pip5k1c
STK32C	Q8QZV4	2	2	6425.5	7470	14470.5	3370	8782	6628.5	7804.5	23098	15406	23467	0.12612147	0.899210975	8103.6	15280.8	Up	0.915085206708525	Serine/threonine-protein kinase 32C OS=Mus musculus GN=Stk32c PE=2 SV=1
CCT5	P80316	28	28	56342.71	63196.82	59381.46	60285.86	54471	60055.82	59510.43	78596.54	61343.64	81988.61	0.126268945	0.898703448	58735.57	68299.008	Up	0.217630166737589	T-complex protein 1 subunit epsilon OS=Mus musculus GN=Cct5 PE=1 SV=1
CLASP2	Q8BRT1	21	21	18017.81	32063.33	28358	39525.86	42020.9	47085.9	57588.57	34604.76	38653.9	34488.67	0.126451018	0.89807767	31997.18	42484.36	Up	0.408987069753719	CLIP-associating protein 2 OS=Mus musculus GN=Clasp2 PE=1 SV=1
CLNS1A	Q61189	3	3	4736	5508	7702	4248	4123	6715	4951	9082	8440	NA	0.126545535	0.897753175	5263.4	7297	Up	0.471308414983226	Methylosome subunit pICln OS=Mus musculus GN=Clns1a PE=2 SV=1
NUDT9	Q8BVU5	2	2	20570	24907.5	36268.5	6458.5	20108	21555.5	19842	64652.5	45126	41051.5	0.126561549	0.897698219	21662.5	38445.5	Up	0.827614992946856	ADP-ribose pyrophosphatase, mitochondrial OS=Mus musculus GN=Nudt9 PE=2 SV=1
GNAT2	P50149	3	3	159916.33	247675	264717.67	229857.33	275468	301057.33	430996	272485.67	220870	304292.33	0.126954962	0.896350321	235526.87	305940.27	Up	0.377358364071821	Guanine nucleotide-binding protein G(t) subunit alpha-2 OS=Mus musculus GN=Gnat2 PE=2 SV=2
RAB5A	Q9CQD1	4	4	32636	33445.25	40087	35934	31063.5	37076.75	39049.25	64813.5	40873.25	90563	0.127283725	0.895227122	34633.15	54475.15	Up	0.653444652470645	Ras-related protein Rab-5A OS=Mus musculus GN=Rab5a PE=1 SV=1
DPYSL2	O08553	174	174	124831.25	126447.44	134853.85	115568.53	120226.84	127324.06	135787.99	141555.96	137811.91	121292.35	0.127369323	0.894935159	124385.58	132754.45	Up	0.0939409982582639	Dihydropyrimidinase-related protein 2 OS=Mus musculus GN=Dpysl2 PE=1 SV=2
MRPS14	Q9CR88	4	4	44650	46725.5	60616	15926	37502	36298.5	35174.5	113804	84170.5	88880	0.127441128	0.894690395	41083.9	71665.5	Up	0.802705628182452	28S ribosomal protein S14, mitochondrial OS=Mus musculus GN=Mrps14 PE=2 SV=1
DNAJB12	Q9QYI4	1	1	17105	24905	17475	34591	27671	35183	39222	NA	26374	NA	0.127503501	0.894477891	24349.4	33593	Up	0.464274417308493	DnaJ homolog subfamily B member 12 OS=Mus musculus GN=Dnajb12 PE=2 SV=2
DBT	P53395	2	2	41688	36876	43227.5	39071.5	24515.5	39207.5	29265	24428.5	27127.5	29704.5	0.127600233	0.894148533	37075.7	29946.6	Down	-0.308081716123928	Lipoamide acyltransferase component of branched-chain alpha-keto acid dehydrogenase complex, mitochondrial OS=Mus musculus GN=Dbt PE=1 SV=2
PDE4A	O89084-3	1	1	5268	5304	3068	6939	5386	7010	5831	6863	11430	NA	0.128080766	0.892516082	5193	7783.5	Up	0.58385081117211	Isoform 3 of cAMP-specific 3',5'-cyclic phosphodiesterase 4A OS=Mus musculus GN=Pde4a
DOCK3	Q8CIQ7	1	1	54482	76374	69876	63507	61693	71459	81210	91276	63724	70262	0.128238975	0.891979964	65186.4	75586.2	Up	0.213551858500131	Dedicator of cytokinesis protein 3 OS=Mus musculus GN=Dock3 PE=1 SV=1
RALA	P63321	4	4	36398	46102.67	32231	37926.67	34445.33	45292	40665.67	86894	48264.67	46871	0.128362459	0.891561973	37420.734	53597.468	Up	0.518326990859498	Ras-related protein Ral-A OS=Mus musculus GN=Rala PE=1 SV=1
SHANK3	Q4ACU6-6	5	5	24802.6	23607	15956.2	24829.6	30224.4	18697.6	20854.4	NA	11154	NA	0.128402905	0.891425151	23883.96	16902	Down	-0.498848088107895	Isoform 5 of SH3 and multiple ankyrin repeat domains protein 3 OS=Mus musculus GN=Shank3
GABRB2	P63137	2	2	28893.5	53087.5	21770	71126.5	60999	78680	73517.5	64901.5	50121	NA	0.128958912	0.88954864	47175.3	66805	Up	0.501924392486076	Gamma-aminobutyric acid receptor subunit beta-2 OS=Mus musculus GN=Gabrb2 PE=1 SV=2
SPG7	Q3ULF4	2	2	4362	3472	4838.5	3161	5517	2795	6098.5	8805	5811	7835	0.129423131	0.887988098	4270.1	6268.9	Up	0.553942460210189	Paraplegin OS=Mus musculus GN=Spg7 PE=1 SV=1
CORO7	Q9D2V7	3	3	4693.5	5365	5990	4645.5	5063	5057.5	4940.5	7511.5	6741.5	6345	0.131659472	0.88054789	5151.4	6119.2	Up	0.248378485062388	Coronin-7 OS=Mus musculus GN=Coro7 PE=2 SV=2
PRRC2B	Q7TPM1	1	1	NA	1472	NA	3323	1441	7386	3450	NA	3957	NA	0.13284094	0.87666806	2078.6667	4931	Up	1.24622182552449	Protein PRRC2B OS=Mus musculus GN=Prrc2b PE=1 SV=1
PALM	Q9Z0P4	19	19	37284.56	53297.89	48897.11	66329.78	62249.11	71710.78	74416.44	62479.33	67565.78	48970.06	0.133058027	0.875958919	53611.69	65028.478	Up	0.278524043849857	Paralemmin-1 OS=Mus musculus GN=Palm PE=1 SV=1
C1QB	P14106	1	1	3617	7831	NA	5641	7132	9619	7611	NA	14104	NA	0.133530541	0.874419392	6055.25	10444.667	Up	0.786508020588766	Complement C1q subcomponent subunit B OS=Mus musculus GN=C1qb PE=1 SV=2
STX7	O70439	8	8	18597	31231.86	25316.14	60039	35028.14	52215.86	53667.71	40417	47360.43	43638	0.133749605	0.873707493	34042.428	47459.8	Up	0.479372083204975	Syntaxin-7 OS=Mus musculus GN=Stx7 PE=1 SV=3
ICAM5	Q60625	27	27	92158.63	96600.37	80350.07	104586.93	118378.22	118798.3	131067.22	116571.67	142507.3	83368.7	0.133969284	0.872994762	98414.844	118462.64	Up	0.267484278349069	Intercellular adhesion molecule 5 OS=Mus musculus GN=Icam5 PE=1 SV=2
OPTN	Q8K3K8	2	2	11653.5	10758.5	8791.5	9536	9775.5	11648.5	11723	NA	15347	NA	0.133976939	0.872969947	10103	12906.167	Up	0.353276808652722	Optineurin OS=Mus musculus GN=Optn PE=1 SV=1
EIF6	O55135	1	1	77894	58183	38860	53379	67802	57440	73048	101069	121381	NA	0.134091084	0.872600098	59223.6	88234.5	Up	0.575170675255684	Eukaryotic translation initiation factor 6 OS=Mus musculus GN=Eif6 PE=1 SV=2
BSN	O88737	212	212	29099.29	31407.33	31629.96	41477.98	39545.03	42669.68	41737.75	32587.77	44992.64	38433.31	0.134362214	0.871722849	34631.918	40084.23	Up	0.210932471264336	Protein bassoon OS=Mus musculus GN=Bsn PE=1 SV=4
GLIPR2	Q9CYL5	1	1	5105	7381	2350	6612	6468	6480	7468	NA	9602	NA	0.134377192	0.87167444	5583.2	7850	Up	0.491600417097914	Golgi-associated plant pathogenesis-related protein 1 OS=Mus musculus GN=Glipr2 PE=2 SV=3
DLAT	Q8BMF4	17	17	99236.53	101134.88	117401.24	76691.06	99579.18	106169.12	104073.06	161466.94	98895.88	149456	0.135225692	0.868940788	98808.578	124012.2	Up	0.327773857250941	Dihydrolipoyllysine-residue acetyltransferase component of pyruvate dehydrogenase complex, mitochondrial OS=Mus musculus GN=Dlat PE=1 SV=2
ATXN2	O70305-3	2	2	13956.5	16828	7667	22789	14005.5	17990.5	20963.5	NA	20096.5	NA	0.135741178	0.867288386	15049.2	19683.5	Up	0.387299977888083	Isoform 3 of Ataxin-2 OS=Mus musculus GN=Atxn2
TRIM3	Q9R1R2	10	10	34792.67	34174.22	45447.11	30950.89	33096.67	36127.44	36917.44	63217.44	40105.11	70491.67	0.135797831	0.867107167	35692.312	49371.82	Up	0.468074472101924	Tripartite motif-containing protein 3 OS=Mus musculus GN=Trim3 PE=1 SV=1
PDE12	Q3TIU4	1	1	1131	1474	NA	7418	3637	6602	5535	NA	6830	NA	0.135837431	0.866980542	3415	6322.3333	Up	0.888571522160374	2',5'-phosphodiesterase 12 OS=Mus musculus GN=Pde12 PE=2 SV=2
GDI1	P50396	30	30	134252.7	177921.63	172030.1	153577.2	184686.1	180315.53	220548.27	142734.1	214530.87	208883.13	0.135951594	0.866615697	164493.55	193402.38	Up	0.233574568565411	Rab GDP dissociation inhibitor alpha OS=Mus musculus GN=Gdi1 PE=1 SV=3
AURKAIP1	Q9DCJ7	1	1	81365	76810	27411	45508	89752	64995	102012	168068	98040	NA	0.136261585	0.865626564	64169.2	108278.75	Up	0.754797235577207	Aurora kinase A-interacting protein OS=Mus musculus GN=Aurkaip1 PE=2 SV=2
MRPS22	Q9CXW2	1	1	13559	14032	9761	18552	11015	18530	11468	21968	22726	NA	0.136414922	0.86513812	13383.8	18673	Up	0.480465937423011	28S ribosomal protein S22, mitochondrial OS=Mus musculus GN=Mrps22 PE=2 SV=1
GFAP	P03995	29	29	69661.04	67526.14	69242.5	60895.07	69983.75	67500.96	64850.89	80226.68	76554.89	87912.07	0.137539575	0.861572321	67461.7	75409.098	Up	0.160669919107414	Glial fibrillary acidic protein OS=Mus musculus GN=Gfap PE=1 SV=4
VPS36	Q91XD6	1	1	41853	32677	29872	34389	34640	36533	51303	NA	55161	NA	0.137597261	0.861390211	34686.2	47665.667	Up	0.458588677756856	Vacuolar protein-sorting-associated protein 36 OS=Mus musculus GN=Vps36 PE=1 SV=1
PNKD	Q69ZP3-4	2	2	5993	2971.5	NA	9601.5	4864	7610.5	9227	NA	9112	NA	0.138073118	0.859890869	5857.5	8649.8333	Up	0.562387285800839	Isoform 4 of Probable hydrolase PNKD OS=Mus musculus GN=Pnkd
ATP5J2	P56135	2	2	58542	60930	73107.5	54965.5	59247	63178.5	78085.5	116611	62662	160708	0.138442362	0.858731	61358.4	96249	Up	0.64951068839393	ATP synthase subunit f, mitochondrial OS=Mus musculus GN=Atp5j2 PE=1 SV=3
HSPH1	Q61699	30	30	64424.32	77501.93	72625.82	72996.07	78463.57	79177.07	79055.64	86129.89	69370.57	83438.86	0.138625151	0.858157967	73202.342	79434.406	Up	0.117874221568652	Heat shock protein 105 kDa OS=Mus musculus GN=Hsph1 PE=1 SV=2
TPD52	Q62393-3	6	6	33883.5	31168.83	28689.17	24588	29971.5	30711.33	30883.67	48386.67	31377.67	54854	0.138685055	0.857970336	29660.2	39242.668	Up	0.40389480374043	Isoform 3 of Tumor protein D52 OS=Mus musculus GN=Tpd52
DYNC1LI1	Q8R1Q8	14	14	35974.46	37152.77	49052.08	33400.15	36511.23	34573.15	43233.77	85140.85	46754	95451.77	0.138720792	0.857858441	38418.138	61030.708	Up	0.66774772876197	Cytoplasmic dynein 1 light intermediate chain 1 OS=Mus musculus GN=Dync1li1 PE=1 SV=1
LYSMD2	Q9D7V2-2	1	1	13792	8443	17251	6474	9662	7382	10752	40305	23143	31682	0.138785441	0.857656089	11124.4	22652.8	Up	1.02596185994673	Isoform 2 of LysM and putative peptidoglycan-binding domain-containing protein 2 OS=Mus musculus GN=Lysmd2
SPHKAP	Q6NSW3-3	1	1	14639	26748	9587	22757	32097	23182	36188	25118	35626	NA	0.138971359	0.857074697	21165.6	30028.5	Up	0.504611023816393	Isoform 3 of A-kinase anchor protein SPHKAP OS=Mus musculus GN=Sphkap
SDF2	Q9DCT5	4	4	257659	275928.67	275117.33	133837.33	234749.67	227931	245772.67	297017	395947.67	348928.33	0.139096861	0.85668267	235458.4	303119.33	Up	0.364413683305543	Stromal cell-derived factor 2 OS=Mus musculus GN=Sdf2 PE=2 SV=1
CCDC43	Q9CR29	2	2	26834.5	36166	24287	27144	27927	33554	31294.5	49147	35561.5	27999.5	0.139212434	0.856321973	28471.7	35511.3	Up	0.318749538918171	Coiled-coil domain-containing protein 43 OS=Mus musculus GN=Ccdc43 PE=1 SV=1
ARL3	Q9WUL7	2	2	479.5	1468	1018	5137.5	1915.5	5364	4557.5	NA	2412	NA	0.139412525	0.855698207	2003.7	4111.1667	Up	1.03688133987944	ADP-ribosylation factor-like protein 3 OS=Mus musculus GN=Arl3 PE=1 SV=1
CARHSP1	Q9CR86	1	1	137406	81241	104967	53541	85075	85545	103233	284406	176312	142604	0.139424249	0.855661685	92446	158420	Up	0.777071679545058	Calcium-regulated heat stable protein 1 OS=Mus musculus GN=Carhsp1 PE=1 SV=1
ATE1	Q9Z2A5	1	1	28684	45387	77189	34476	52854	48108	60317	84168	53823	109184	0.139662945	0.854918803	47718	71120	Up	0.575721747407561	Arginyl-tRNA--protein transferase 1 OS=Mus musculus GN=Ate1 PE=1 SV=2
GDI2	Q61598-2	15	15	41888.4	45938.07	49730.6	46785.73	47455.73	45593.13	50503.07	76792.8	48008.07	67228.73	0.139862641	0.854298274	46359.706	57625.16	Up	0.313827434070083	Isoform 2 of Rab GDP dissociation inhibitor beta OS=Mus musculus GN=Gdi2
H2AFX	P27661	10	10	369796.8	193241.1	253291.1	70689.1	233792	155226.2	143773.2	158848	146110.6	68771.2	0.14055215	0.852162507	224162.02	134545.84	Down	-0.736444076329631	Histone H2AX OS=Mus musculus GN=H2afx PE=1 SV=2
PRKAR1A	Q9DBC7	8	8	43111.75	47208.88	40627.5	50144.62	44792	38439.25	43899.38	23195.38	44187	40054.62	0.140708262	0.8516804	45176.95	37955.126	Down	-0.251292133497049	cAMP-dependent protein kinase type I-alpha regulatory subunit OS=Mus musculus GN=Prkar1a PE=1 SV=3
VPS50	Q8CI71	1	1	9923	16187	16614	21929	20344	21272	27689	44083	19350	NA	0.140843214	0.851264074	16999.4	28098.5	Up	0.725009289383958	Coiled-coil domain-containing protein 132 OS=Mus musculus GN=Ccdc132 PE=2 SV=2
UTP18	Q5SSI6	1	1	1253	1249	NA	937	2001	4647	3687	NA	1705	NA	0.141215418	0.850117884	1360	3346.3333	Up	1.2989745100975	U3 small nucleolar RNA-associated protein 18 homolog OS=Mus musculus GN=Utp18 PE=1 SV=1
SCG2	Q03517	15	15	43978.77	46193.31	46675.46	39175.23	45135.08	42982.31	46694.15	48505.31	69574.69	79809.92	0.141509389	0.849214743	44231.57	57513.276	Up	0.378818565456271	Secretogranin-2 OS=Mus musculus GN=Scg2 PE=1 SV=1
DGKB	Q6NS52	2	2	11313.5	NA	4082	14651	11366	18256	14264.5	39929.5	16110.5	NA	0.142165111	0.847206973	10353.125	22140.125	Up	1.09659706908739	Diacylglycerol kinase beta OS=Mus musculus GN=Dgkb PE=2 SV=2
NAA11	Q3UX61	2	2	47281	57854.5	51855	49916	56248.5	44213.5	48755	13905.5	54941.5	36044	0.142191194	0.847127299	52631	39571.9	Down	-0.41143647025492	N-alpha-acetyltransferase 11 OS=Mus musculus GN=Naa11 PE=2 SV=1
FXR1	Q61584-3	3	3	21280	24764.5	31126	33462.5	20873	35873.5	27827	65570.5	39995.5	NA	0.142191417	0.847126619	26301.2	42316.625	Up	0.686095944210647	Isoform B of Fragile X mental retardation syndrome-related protein 1 OS=Mus musculus GN=Fxr1
DLGAP3	Q6PFD5	19	19	26617.05	26117.32	42349.68	19700.16	32098.42	27840.95	31157.05	57536.95	41075.42	83059.16	0.142701465	0.845571569	29376.526	48133.906	Up	0.712389704559822	Disks large-associated protein 3 OS=Mus musculus GN=Dlgap3 PE=1 SV=1
ATXN3	Q9CVD2	1	1	2516	2934	4482	8818	3193	8209	7627	6218	4746	NA	0.142822885	0.8452022	4388.6	6700	Up	0.610400314166318	Ataxin-3 OS=Mus musculus GN=Atxn3 PE=1 SV=2
VAT1	Q62465	5	5	61806	84502	117718.2	79861	82173.2	78114.4	97677	169580.8	95654	160475	0.143041976	0.8445365	85212.08	120300.24	Up	0.497509648513771	Synaptic vesicle membrane protein VAT-1 homolog OS=Mus musculus GN=Vat1 PE=1 SV=3
MCCC2	Q3ULD5	9	9	23768.44	25542	17657.22	25527.22	24016.11	31204	26284	22933.11	27798.33	NA	0.14369593	0.842555533	23302.198	27054.86	Up	0.215421731110676	Methylcrotonoyl-CoA carboxylase beta chain, mitochondrial OS=Mus musculus GN=Mccc2 PE=1 SV=1
H1F0	P10922	5	5	377166.4	172840.8	183834	96892.2	237429.2	152497.8	131197.6	140850.6	110957.4	108839.4	0.143762988	0.842352908	213632.52	128868.56	Down	-0.729230943554865	Histone H1.0 OS=Mus musculus GN=H1f0 PE=2 SV=4
EID2	Q6X7S9	1	1	14466	19170	11838	10630	14083	18777	15732	14958	24517	NA	0.14395648	0.841768781	14037.4	18496	Up	0.397937557797692	EP300-interacting inhibitor of differentiation 2 OS=Mus musculus GN=Eid2 PE=1 SV=1
CACNB4	Q8R0S4-2	10	10	52405.1	50598	52691.3	28766.7	50578.9	46248.2	48613.8	61057.5	66010.6	93310.9	0.143986844	0.841677187	47008	63048.2	Up	0.423548881788651	Isoform 2 of Voltage-dependent L-type calcium channel subunit beta-4 OS=Mus musculus GN=Cacnb4
CDK14	O35495	1	1	5902	6675	15616	18399	12726	28110	20992	NA	13745	NA	0.144062479	0.841449115	11863.6	20949	Up	0.820339517571264	Cyclin-dependent kinase 14 OS=Mus musculus GN=Cdk14 PE=2 SV=2
SORBS1	Q62417	9	9	34619.38	39861.38	35591.25	27690.12	41531.88	32139.5	34907.25	51413.75	49489.25	56333.62	0.144582216	0.839885124	35858.802	44856.674	Up	0.322995362205494	Sorbin and SH3 domain-containing protein 1 OS=Mus musculus GN=Sorbs1 PE=1 SV=2
BCL11A	Q9QYE3-15	1	1	7337	3611	5858	3780	9552	6398	11294	NA	12210	NA	0.145630054	0.836748991	6027.6	9967.3333	Up	0.725623895859252	Isoform 7 of B-cell lymphoma/leukemia 11A OS=Mus musculus GN=Bcl11a
MRPS2	Q924T2	2	2	NA	1273	NA	1249	1624	1725	2090	NA	NA	NA	0.145647721	0.836696306	1382	1907.5	Up	0.464925441333634	28S ribosomal protein S2, mitochondrial OS=Mus musculus GN=Mrps2 PE=2 SV=1
SRGAP3	Q812A2	7	7	27962.86	34111.86	35597.57	40830.29	39493.43	36428.57	43249.57	43148.57	40340.86	70637	0.145860694	0.836061725	35599.202	46760.914	Up	0.393458227640992	SLIT-ROBO Rho GTPase-activating protein 3 OS=Mus musculus GN=Srgap3 PE=1 SV=1
CRH	Q8CIT0	1	1	14802	14154	12542	15050	27663	18802	24484	NA	31137	NA	0.145973473	0.835726059	16842.2	24807.667	Up	0.558705444430531	Corticoliberin OS=Mus musculus GN=Crh PE=2 SV=1
PHB	P67778	34	34	61890	60325.73	64597.48	62894.06	59073.52	68324.18	62218.21	84451.91	58898.45	78485.36	0.145995958	0.835659168	61756.158	70475.622	Up	0.190541303864254	Prohibitin OS=Mus musculus GN=Phb PE=1 SV=1
ARHGEF2	Q60875-3	7	7	37529.86	30928.14	30093	38254.29	46919.29	52009.29	48632.71	44340	58955.57	28572.71	0.146264273	0.834861742	36744.916	46502.056	Up	0.339749850134951	Isoform 3 of Rho guanine nucleotide exchange factor 2 OS=Mus musculus GN=Arhgef2
TPP1	O89023	1	1	139056	111729	221174	111168	108728	158450	133958	231370	178303	215059	0.146346078	0.834618912	138371	183428	Up	0.406672268423538	Tripeptidyl-peptidase 1 OS=Mus musculus GN=Tpp1 PE=1 SV=2
GDI2	Q61598	17	17	41752.71	45642.47	49150.71	45916.53	46596.65	45205.12	49653.06	71109.47	47195.41	59319.47	0.146558879	0.833987866	45811.814	54496.506	Up	0.250444045558189	Rab GDP dissociation inhibitor beta OS=Mus musculus GN=Gdi2 PE=1 SV=1
SMS	P97355	1	1	NA	4200	NA	15095	5594	15706	16585	NA	NA	NA	0.146773135	0.833353429	8296.3333	16145.5	Up	0.960586353978552	Spermine synthase OS=Mus musculus GN=Sms PE=1 SV=1
FAM65A	Q68FE6	3	3	1472	1244.5	975.5	2580.5	1464	3015	2548.5	1660.5	1764.5	NA	0.146940748	0.832857753	1547.3	2247.125	Up	0.538327436561914	Protein FAM65A OS=Mus musculus GN=Fam65a PE=1 SV=2
ILF3	Q9Z1X4	1	1	14197	9159	11297	6723	16702	13587	11978	32307	27931	NA	0.147487536	0.831244681	11615.6	21450.75	Up	0.884964412715885	Interleukin enhancer-binding factor 3 OS=Mus musculus GN=Ilf3 PE=1 SV=2
ME1	P06801	2	2	12417	15094.5	14115.5	7132	13147	14202.5	13454.5	11885.5	23311	27359	0.147510977	0.831175659	12381.2	18042.5	Up	0.543248105789011	NADP-dependent malic enzyme OS=Mus musculus GN=Me1 PE=1 SV=2
DGKQ	Q6P5E8	1	1	29372	36928	53189	13214	34752	26622	32790	98564	55283	82864	0.14789813	0.830037317	33491	59224.6	Up	0.822423095823611	Diacylglycerol kinase theta OS=Mus musculus GN=Dgkq PE=2 SV=1
SMC3	Q9CW03	2	2	7185	3401	7347	8683	8358	12284	9478	NA	NA	NA	0.147928416	0.829948394	6994.8	10881	Up	0.637456438551141	Structural maintenance of chromosomes protein 3 OS=Mus musculus GN=Smc3 PE=1 SV=2
PPP6R2	Q8R3Q2	2	2	8489.5	8073	17562.5	5680	7385.5	11745	10202	13881	14498	28525.5	0.148701146	0.827685683	9438.1	15770.3	Up	0.740641742390627	Serine/threonine-protein phosphatase 6 regulatory subunit 2 OS=Mus musculus GN=Ppp6r2 PE=1 SV=1
BRSK1	Q5RJI5-3	1	1	6795	11439	NA	9970	16858	18114	15039	36012	14898	NA	0.149286414	0.825979715	11265.5	21015.75	Up	0.899559597064164	Isoform 3 of Serine/threonine-protein kinase BRSK1 OS=Mus musculus GN=Brsk1
SNX2	Q9CWK8	6	6	34649.5	40931.67	37108.33	28692.83	36314.33	38975.5	42556.33	42841.67	44042.67	32443.17	0.149547121	0.825221944	35539.332	40171.868	Up	0.176768983939634	Sorting nexin-2 OS=Mus musculus GN=Snx2 PE=1 SV=2
LMTK3	Q5XJV6	1	1	9748	9559	9910	3138	9704	8016	10461	22890	19811	NA	0.149607455	0.825046764	8411.8	15294.5	Up	0.862526489922046	Serine/threonine-protein kinase LMTK3 OS=Mus musculus GN=Lmtk3 PE=1 SV=1
AKR1B3	P45376	3	3	51973.33	53334.67	62228.33	49899.33	50022	48538	55559.33	74236.67	61992	81089	0.14997814	0.823972038	53491.532	64283	Up	0.265126736065667	Aldose reductase OS=Mus musculus GN=Akr1b1 PE=1 SV=3
MRPL3	Q99N95	2	2	44095	45838.5	40569.5	47890.5	43842	43041.5	43077.5	22890.5	44802	22009	0.15018516	0.823372979	44447.1	35164.1	Down	-0.337985998868407	39S ribosomal protein L3, mitochondrial OS=Mus musculus GN=Mrpl3 PE=2 SV=1

GNAI1	B2RSH2	7	7	80897.57	125300.43	122320.57	117432.71	138656.14	156014.43	215227.14	128408.43	116982.86	132267.43	0.150392929	0.822772581	116921.48	149780.06	Up	0.357305507978715	Guanine nucleotide-binding protein G(i) subunit alpha-1 OS=Mus musculus GN=Gnai1 PE=2 SV=1
FSD1	Q7TPM6	2	2	18528.5	24711.5	8273.5	22682	24630.5	23709	23994.5	NA	29465	NA	0.150713837	0.821846874	19765.2	25722.833	Up	0.380087008649276	Fibronectin type III and SPRY domain-containing protein 1 OS=Mus musculus GN=Fsd1 PE=2 SV=1
DCTN5	Q9QZB9	2	2	52128.5	62197	42167.5	59332	56621	55491	59425	87792	71260.5	NA	0.150810489	0.821568451	54489.2	68492.125	Up	0.329967811564461	Dynactin subunit 5 OS=Mus musculus GN=Dctn5 PE=1 SV=1
GAPVD1	Q6PAR5-4	2	2	22890.5	33585.5	21551.5	22898.5	21460.5	24910.5	43789.5	NA	33687	69958	0.150954283	0.821154559	24477.3	43086.25	Up	0.815783110865089	Isoform 4 of GTPase-activating protein and VPS9 domain-containing protein 1 OS=Mus musculus GN=Gapvd1
HIST1H1C	P15864	16	16	324752.12	175980	174555.38	94129.38	216972.06	145551.44	128715.62	143929.81	133331.62	100694.62	0.151008999	0.820997171	197277.79	130444.62	Down	-0.596791062930982	Histone H1.2 OS=Mus musculus GN=Hist1h1c PE=1 SV=2
TPM4	Q6IRU2	2	2	48443.5	48770	35346.5	48730	53673.5	67554.5	57234	80129	93494	31150	0.151443707	0.819748769	46992.7	65912.3	Up	0.488111052770934	Tropomyosin alpha-4 chain OS=Mus musculus GN=Tpm4 PE=2 SV=3
VCP	Q01853	84	84	68462.22	77686.18	66756.78	75755.92	68444.26	69011.4	79354.03	78367.95	73411.55	87389.63	0.151492515	0.819608824	71421.072	77506.912	Up	0.117975186104415	Transitional endoplasmic reticulum ATPase OS=Mus musculus GN=Vcp PE=1 SV=4
SFPQ	Q8VIJ6	10	10	117809.5	66378.3	77795.4	49684.9	80401.9	58782	62470.5	74862.2	55904.3	34038.2	0.151682282	0.819065147	78414	57211.44	Down	-0.454807598293725	Splicing factor, proline- and glutamine-rich OS=Mus musculus GN=Sfpq PE=1 SV=1
NTRK2	P15209-3	7	7	14994.86	21596.57	19194.43	28219.14	22038.43	29782.86	25813.86	32371.86	24175.29	70557.29	0.151753299	0.81886186	21208.686	36540.232	Up	0.784830553517618	Isoform L1 of BDNF/NT-3 growth factors receptor OS=Mus musculus GN=Ntrk2
ARHGDIA	Q99PT1	18	18	100326.33	99455.33	82141.56	133812.06	85896.78	91239.28	95548	84308.5	83945.72	51621.22	0.151830767	0.818640215	100326.41	81332.544	Down	-0.302796815076247	Rho GDP-dissociation inhibitor 1 OS=Mus musculus GN=Arhgdia PE=1 SV=3
DTD2	Q8BHA3	1	1	36894	36669	11175	42122	40843	42877	42137	29900	54595	78997	0.151918091	0.818390505	33540.6	49701.2	Up	0.567372187880656	Probable D-tyrosyl-tRNA(Tyr) deacylase 2 OS=Mus musculus GN=Dtd2 PE=2 SV=1
CEP170B	Q80U49	22	22	16735	15599.62	19711.95	14114.67	20076.9	17831.57	20642.14	15977.86	23924.33	22998.62	0.152157945	0.817705364	17247.628	20274.904	Up	0.233297116188856	Centrosomal protein of 170 kDa protein B OS=Mus musculus GN=Cep170b PE=1 SV=2
MBNL2	Q8C181-3	2	2	3184.5	1950	5858	9955	4271	8539.5	7250	4884	8049	9413.5	0.152312491	0.817264479	5043.7	7627.2	Up	0.596671064010272	Isoform 3 of Muscleblind-like protein 2 OS=Mus musculus GN=Mbnl2
HAPLN4	Q80WM4	1	1	40522	57447	23621	28289	50332	42156	45800	66452	44782	69742	0.152833461	0.815781551	40042.2	53786.4	Up	0.425720189579983	Hyaluronan and proteoglycan link protein 4 OS=Mus musculus GN=Hapln4 PE=2 SV=2
OTUD6B	Q8K2H2	4	4	35905.67	37986.67	16262.33	46117.33	42859.33	45410	47134	40030.33	47958	NA	0.152933169	0.815498313	35826.266	45133.083	Up	0.333167628930303	OTU domain-containing protein 6B OS=Mus musculus GN=Otud6b PE=2 SV=1
RBM14	Q8C2Q3-2	2	2	22023.5	15353	16461.5	17027.5	18164	21475.5	16098	8348	9454	11619.5	0.152992341	0.81533031	17805.9	13399	Down	-0.410230025771822	Isoform 2 of RNA-binding protein 14 OS=Mus musculus GN=Rbm14
CRTC2	Q3U182	2	2	1112.5	639.5	NA	1729	1977	2101	2066.5	NA	1669	NA	0.153518433	0.813839471	1364.5	1945.5	Up	0.511768585698715	CREB-regulated transcription coactivator 2 OS=Mus musculus GN=Crtc2 PE=1 SV=2
PJA2	Q80U04	2	2	11213	11869.5	12239.5	11114	12406	11586	9506.5	11677.5	10244.5	NA	0.153580931	0.813662703	11768.4	10753.625	Down	-0.130095120775898	E3 ubiquitin-protein ligase Praja-2 OS=Mus musculus GN=Pja2 PE=1 SV=2
ESD	Q9R0P3	1	1	18161	16172	21364	18176	15155	22785	21134	16443	24923	NA	0.15369731	0.813333733	17805.6	21321.25	Up	0.259960970000762	S-formylglutathione hydrolase OS=Mus musculus GN=Esd PE=1 SV=1
RABEP1	O35551-4	2	2	10789.5	12342	2972.5	17611.5	15533	18188.5	16792.5	11820	27391.5	NA	0.153971262	0.812560332	11849.7	18548.125	Up	0.646422819769169	Isoform 4 of Rab GTPase-binding effector protein 1 OS=Mus musculus GN=Rabep1
SEC22B	O08547	1	1	6049	6978	NA	14514	11693	15713	17508	NA	10700	NA	0.154007478	0.812458191	9808.5	14640.333	Up	0.577843972377423	Vesicle-trafficking protein SEC22b OS=Mus musculus GN=Sec22b PE=1 SV=3
RNF214	Q8BFU3-3	6	6	16892.4	14722.4	13793.6	16396.8	16634.2	15044.2	17009.2	16234.6	26360	28259.4	0.154436748	0.811249353	15687.88	20581.48	Up	0.391696323967924	Isoform 3 of RING finger protein 214 OS=Mus musculus GN=Rnf214
NUMBL	O08919	9	9	91385.78	95232.67	81605.67	68038.44	83766.56	80461.78	89603.78	89138.33	117235.44	129838.44	0.154518863	0.811018497	84005.824	101255.55	Up	0.269439787490904	Numb-like protein OS=Mus musculus GN=Numbl PE=1 SV=3
GNAI3	Q9DC51	4	4	137309.75	213238	209094.75	196819	238057.75	265758.25	367876.5	215287.25	197588.5	228219.25	0.155592298	0.808011906	198903.85	254945.95	Up	0.3581202185279	Guanine nucleotide-binding protein G(k) subunit alpha OS=Mus musculus GN=Gnai3 PE=1 SV=3
PPP2R5E	Q61151	1	1	NA	2002	NA	3984	2501	4419	5925	NA	3286	NA	0.1557943	0.807448437	2829	4543.3333	Up	0.683458979575163	Serine/threonine-protein phosphatase 2A 56 kDa regulatory subunit epsilon isoform OS=Mus musculus GN=Ppp2r5e PE=2 SV=3
DDX1	Q91VR5	7	7	48053.5	53118.83	69107.67	29474.33	56578.67	44595.67	59954.67	94471.33	63834	131337	0.156046524	0.806745902	51266.6	78838.534	Up	0.620881728364656	ATP-dependent RNA helicase DDX1 OS=Mus musculus GN=Ddx1 PE=1 SV=1
GSR	P47791	2	2	139719	152816	250009.5	72375.5	172170	147118	188067	249939.5	226393	433605.5	0.156113053	0.806560782	157418	249024.6	Up	0.66168775097284	Glutathione reductase, mitochondrial OS=Mus musculus GN=Gsr PE=1 SV=3
GLRX2	Q923X4	2	2	52855	72729.5	56767.5	36312	64379	60496	60788.5	76313.5	69849.5	71175	0.156164674	0.806417202	56608.6	67724.5	Up	0.258656592776149	Glutaredoxin-2, mitochondrial OS=Mus musculus GN=Glrx2 PE=1 SV=1
SLC17A8	Q8BFU8	1	1	600	977	NA	3212	756	2671	2484	NA	5791	NA	0.156380511	0.805817371	1386.25	3648.6667	Up	1.39618189543726	Vesicular glutamate transporter 3 OS=Mus musculus GN=Slc17a8 PE=2 SV=1
SART1	Q9Z315	3	3	12124.5	15024	16034	10784	13920.5	15417	15212.5	NA	15081	NA	0.156764223	0.804753047	13577.4	15236.833	Up	0.166355861498913	U4/U6.U5 tri-snRNP-associated protein 1 OS=Mus musculus GN=Sart1 PE=2 SV=1
ACOT7	Q91V12-3	14	14	60363	63211.29	87907	33410.36	56796.29	56762.93	66267.71	103376.07	72530.79	126522.29	0.156835674	0.804555145	60337.588	85091.958	Up	0.495965764154516	Isoform C of Cytosolic acyl coenzyme A thioester hydrolase OS=Mus musculus GN=Acot7
ITGA6	Q61739	5	5	2806.67	4003.67	1052.67	5394.67	4032.67	5688.33	4896.33	NA	4024	NA	0.156851378	0.804511661	3458.07	4869.5533	Up	0.493822372095735	Integrin alpha-6 OS=Mus musculus GN=Itga6 PE=1 SV=3
SUPV3L1	Q80YD1	1	1	34879	38018	30410	20523	35239	35135	32952	45005	61796	112987	0.156926993	0.804302348	31813.8	57575	Up	0.855789801109285	ATP-dependent RNA helicase SUPV3L1, mitochondrial OS=Mus musculus GN=Supv3l1 PE=2 SV=1
MDH1	P14152	56	56	154629.48	196093.5	185174.45	122802.02	196677.52	173224.8	223854.8	205572.5	167881.59	244152.73	0.157240267	0.803436227	171075.39	202937.28	Up	0.246401673938955	Malate dehydrogenase, cytoplasmic OS=Mus musculus GN=Mdh1 PE=1 SV=3
UROS	P51163	1	1	21270	18420	22196	14228	25787	21613	25371	NA	36259	56100	0.157263337	0.803372512	20380.2	34835.75	Up	0.773400414899572	Uroporphyrinogen-III synthase OS=Mus musculus GN=Uros PE=2 SV=1
ARPC5L	Q9D898	13	13	77005.69	77566.85	73977.08	66790.08	75705.46	65807.15	75149.23	57022.54	67464.38	74719.08	0.157309328	0.803245525	74209.032	68032.476	Down	-0.125371192248357	Actin-related protein 2/3 complex subunit 5-like protein OS=Mus musculus GN=Arpc5l PE=1 SV=1
ADGRL2	Q8JZZ7	1	1	26637	33040	21003	45036	37902	50316	38886	NA	37607	NA	0.157359779	0.803106264	32723.6	42269.667	Up	0.369291264223599	Latrophilin-2 (Fragment) OS=Mus musculus GN=Lphn2 PE=1 SV=2
CKMT1	P30275	87	87	68406.94	67597.17	61015.81	60936.59	67495.42	62714.81	63318.81	59483.77	65402.12	56893.87	0.157729341	0.802087512	65090.386	61562.676	Down	-0.0803885257971498	Creatine kinase U-type, mitochondrial OS=Mus musculus GN=Ckmt1 PE=1 SV=1
LSM14B	Q8CGC4	2	2	41705.5	55662.5	69230.5	27744	45575	44910	50546	124029	62005	165145.5	0.158101828	0.801063108	47983.5	89327.1	Up	0.89655953151903	Protein LSM14 homolog B OS=Mus musculus GN=Lsm14b PE=2 SV=3
STRIP1	Q8C079-4	1	1	7669	9691	NA	43710	20731	42363	36680	NA	29207	NA	0.158790299	0.799176034	20450.25	36083.333	Up	0.819214139200296	Isoform 4 of Striatin-interacting protein 1 OS=Mus musculus GN=Strip1
RUFY2	Q8R4C2	1	1	NA	9504	9520	15641	10605	14533	18499	NA	NA	NA	0.159258355	0.797897774	11317.5	16516	Up	0.545309017040975	RUN and FYVE domain-containing protein 2 OS=Mus musculus GN=Rufy2 PE=1 SV=2
PKP4	Q68FH0-3	4	4	21170.25	27423.5	1850.5	34107.25	37291.5	45122.25	43505.5	26079.25	31906.25	NA	0.159400747	0.797509648	24368.6	36653.313	Up	0.588920216922974	Isoform 3 of Plakophilin-4 OS=Mus musculus GN=Pkp4
HRAS	Q61411	31	31	91221.34	104523.9	98531.55	80388.14	105015.41	96784.17	109630.69	95033.93	121324.83	157280.86	0.159431837	0.79742495	95936.068	116010.9	Up	0.274115096725003	GTPase HRas OS=Mus musculus GN=Hras PE=1 SV=2
MTCL1	Q3UHU5-3	2	2	119772.5	194585.5	383219.5	223387.5	202598	234356.5	339737.5	595518	197798.5	887110.5	0.159483918	0.797283105	224712.6	450904.2	Up	1.00473992715295	Isoform 3 of Protein SOGA2 OS=Mus musculus GN=Soga2
PAFAH1B3	Q61205	1	1	13134	17513	7469	15750	14195	14051	19462	14991	22394	NA	0.160062604	0.795710122	13612.2	17724.5	Up	0.380844676614887	Platelet-activating factor acetylhydrolase IB subunit gamma OS=Mus musculus GN=Pafah1b3 PE=1 SV=1
GANAB	Q8BHN3	4	4	73363.25	93047	109212.5	89749.75	76054.75	97090	95048.75	138106.25	93180.75	100639	0.16038993	0.794822903	88285.45	104812.95	Up	0.247569380497566	Neutral alpha-glucosidase AB OS=Mus musculus GN=Ganab PE=1 SV=1
LDHA	P06151	9	9	38963.22	49364.11	63831.78	39586.67	42534.33	50261	59974.33	65203.22	44224.33	96811.78	0.160404733	0.79478282	46856.022	63294.932	Up	0.433855511321269	L-lactate dehydrogenase A chain OS=Mus musculus GN=Ldha PE=1 SV=3
BCAR1	Q61140	2	2	5901	16600	3929	22164	22138	16995	17014	NA	24909	27891	0.16108567	0.792943091	14146.4	21702.25	Up	0.617409662840906	Breast cancer anti-estrogen resistance protein 1 OS=Mus musculus GN=Bcar1 PE=1 SV=2
LMNB2	P21619	9	9	43667.78	23096.11	19580	17662.33	33181.22	26206.44	21853.11	12685.89	23277.56	3990.78	0.161106097	0.792888024	27437.488	17602.756	Down	-0.640347079569715	Lamin-B2 OS=Mus musculus GN=Lmnb2 PE=1 SV=2
CCDC58	Q8R3Q6	2	2	18104.5	14288.5	4665.5	34849	21926	30865	28118.5	NA	23561	NA	0.16189795	0.790758649	18766.7	27514.833	Up	0.552034605705671	Coiled-coil domain-containing protein 58 OS=Mus musculus GN=Ccdc58 PE=2 SV=1
SCN8A	Q9WTU3-4	1	1	4972	5669	14660	5513	4815	8965	10949	NA	17147	NA	0.162568732	0.788962982	7125.8	12353.667	Up	0.793815411753502	Isoform 4 of Sodium channel protein type 8 subunit alpha OS=Mus musculus GN=Scn8a
EIF4A3	Q91VC3	1	1	48599	52897	86662	26066	44614	37652	50867	151502	81636	136569	0.16268988	0.78863946	51767.6	91645.2	Up	0.82400988538963	Eukaryotic initiation factor 4A-III OS=Mus musculus GN=Eif4a3 PE=2 SV=3
GNAI2	P08752	7	7	79603	124770	121363.29	121893.71	138586.57	156753.29	214305.43	125025.29	117322	132267.43	0.162807592	0.788325348	117243.31	149134.69	Up	0.347110207532069	Guanine nucleotide-binding protein G(i) subunit alpha-2 OS=Mus musculus GN=Gnai2 PE=1 SV=5
RAC3	P60764	4	4	28136	43081.25	57244	75181.25	32419.25	50714.5	47680.25	93922.5	54106.25	109856	0.162975688	0.787877177	47212.35	71255.9	Up	0.59384517945618	Ras-related C3 botulinum toxin substrate 3 OS=Mus musculus GN=Rac3 PE=1 SV=1
ANK3	G5E8K5-6	12	12	26831.83	34138.67	52529.42	27391.75	32285.92	31037.33	39185.08	93096.92	38180.58	88222.92	0.163206996	0.787261228	34635.518	57944.566	Up	0.742421122090392	Isoform 6 of Ankyrin-3 OS=Mus musculus GN=Ank3
GAPDH	P16858	81	81	213304.19	222427.5	224378.34	255052.73	240190.61	247576.65	250368.23	303737.35	218755.06	380902.96	0.163374849	0.786814801	231070.67	280268.05	Up	0.278473115508878	Glyceraldehyde-3-phosphate dehydrogenase OS=Mus musculus GN=Gapdh PE=1 SV=2
SUMF2	Q8BPG6	2	2	24496.5	27518	14115.5	21022	28283	28268	32175.5	19568.5	27965.5	37577.5	0.163690142	0.785977474	23087	29111	Up	0.334483681930612	Sulfatase-modifying factor 2 OS=Mus musculus GN=Sumf2 PE=2 SV=2
DMXL2	Q8BPN8	3	3	9052.67	15028	15137	31863.33	18478.33	26114.33	21391.67	NA	26290.67	NA	0.163793695	0.785702819	17911.866	24598.89	Up	0.457677576445796	DmX-like protein 2 OS=Mus musculus GN=Dmxl2 PE=1 SV=3
HRAS	Q61411-2	26	26	101545.92	116534.29	106886.04	92047.04	117003.58	109024.46	121605.42	105164.79	133380.88	177141.92	0.163800768	0.785684065	106803.37	129263.49	Up	0.275357669807599	Isoform 2 of GTPase HRas OS=Mus musculus GN=Hras
1700025G04RIK	Q8K207	2	2	15033.5	9022.5	17285	11285.5	23768	15673.5	18692	23208.5	23270.5	NA	0.164021764	0.785098521	15278.9	20211.125	Up	0.403608947141902	Uncharacterized protein C1orf21 homolog OS=Mus musculus PE=1 SV=1
ANK3	G5E8K5-5	10	10	32024.1	40731	62896	32617.6	38435.5	36713.3	46741.2	111215.3	45700.2	105867.5	0.164370026	0.784177376	41340.84	69247.5	Up	0.744194284344519	Isoform 5 of Ankyrin-3 OS=Mus musculus GN=Ank3
OCIAD1	Q9CRD0-2	1	1	4220	6427	3278	8786	5664	8710	4595	9482	9348	NA	0.16456768	0.783655453	5675	8033.75	Up	0.501453174858341	Isoform 2 of OCIA domain-containing protein 1 OS=Mus musculus GN=Ociad1
CLVS1	Q9D4C9-3	1	1	6573	10721	NA	22470	15397	28278	22152	NA	15613	NA	0.165288355	0.781757742	13790.25	22014.333	Up	0.674794544021951	Isoform 3 of Clavesin-1 OS=Mus musculus GN=Clvs1
PCYT1B	Q811Q9-2	1	1	10263	11209	2199	12931	8471	14049	9809	NA	14433	NA	0.165518336	0.781153889	9014.6	12763.667	Up	0.501707454831418	Isoform 2 of Choline-phosphate cytidylyltransferase B OS=Mus musculus GN=Pcyt1b
PTPRN2	P80560	6	6	24277.5	23879.33	26426.83	18368	24919.67	27784.67	26491.83	22038.83	31151.5	46742.17	0.165527492	0.781129864	23574.266	30841.8	Up	0.387674114428814	Receptor-type tyrosine-protein phosphatase N2 OS=Mus musculus GN=Ptprn2 PE=1 SV=2
RHOC	Q62159	2	2	106603.5	165448.5	204302	156189.5	127802.5	139893	177959.5	304726	153653	311400.5	0.165925998	0.780085562	152069.2	217526.4	Up	0.516462523559106	Rho-related GTP-binding protein RhoC OS=Mus musculus GN=Rhoc PE=1 SV=2
DNAJA2	Q9QYJ0	9	9	52960.89	59278.22	52120.78	101123.44	58931.44	60399.56	53962.33	50690.78	44973.78	33419.33	0.166369744	0.778925653	64882.954	48689.156	Down	-0.414239011776462	DnaJ homolog subfamily A member 2 OS=Mus musculus GN=Dnaja2 PE=1 SV=1
MBP	P04370-7	52	52	891042.77	712721.21	818273.73	395431.29	753258.17	564682.96	355315.19	642554.6	468351.4	721631.19	0.166753124	0.777926021	714145.43	550507.07	Down	-0.375456818775208	Isoform 7 of Myelin basic protein OS=Mus musculus GN=Mbp
CRYM	O54983	7	7	54217.43	62337.29	71442.14	77060.57	48747.14	69240.71	63700.71	82050.29	70102.29	76224.71	0.167022822	0.777224182	62760.914	72263.742	Up	0.203405600394787	Ketimine reductase mu-crystallin OS=Mus musculus GN=Crym PE=1 SV=1
VPS9D1	Q8C190	1	1	25246	38370	65411	17407	36869	25670	42691	105247	60714	71479	0.167269596	0.776582993	36660.6	61160.2	Up	0.738362726749486	VPS9 domain-containing protein 1 OS=Mus musculus GN=Vps9d1 PE=2 SV=1
STK4	Q9JI11	1	1	19436	27184	11050	29535	27986	36133	37166	NA	23503	NA	0.167758681	0.775314996	23038.2	32267.333	Up	0.486046352392395	Serine/threonine-protein kinase 4 OS=Mus musculus GN=Stk4 PE=1 SV=1
SNRK	Q8VDU5	1	1	20044	24571	8025	27545	30994	31571	34230	20997	32991	NA	0.16793674	0.77485428	22235.8	29947.25	Up	0.42953921817649	SNF-related serine/threonine-protein kinase OS=Mus musculus GN=Snrk PE=2 SV=1
KCND2	Q9Z0V2	2	2	30931	30579.5	42743	31674.5	37509	35247.5	37466.5	61127	36699	84797.5	0.168552121	0.773265779	34687.4	51067.5	Up	0.557993726687359	Potassium voltage-gated channel subfamily D member 2 OS=Mus musculus GN=Kcnd2 PE=1 SV=1
MEF2D	Q63943	2	2	24465.5	19410.5	19825.5	26264.5	20936.5	16272.5	19744	4831	21139.5	NA	0.168642016	0.773034215	22180.5	15496.75	Down	-0.517326204355874	Myocyte-specific enhancer factor 2D OS=Mus musculus GN=Mef2d PE=1 SV=2
EPHA7	Q61772-3	1	1	17436	NA	NA	11662	13127	NA	25897	NA	19542	NA	0.169037557	0.772016793	14075	22719.5	Up	0.690796162737006	Isoform 3 of Ephrin type-A receptor 7 OS=Mus musculus GN=Epha7
RUVBL1	P60122	3	3	21073.67	22502.33	43501.67	20129.33	19700	27328.33	23585	42870.33	33974.33	60986	0.169180668	0.771649265	25381.4	37748.798	Up	0.572659058496657	RuvB-like 1 OS=Mus musculus GN=Ruvbl1 PE=1 SV=1
COX7B	P56393	1	1	235117	231504	463451	150157	206063	288848	213899	605396	327637	724455	0.169215216	0.771560588	257258.4	432047	Up	0.747970078968528	Cytochrome c oxidase subunit 7B, mitochondrial OS=Mus musculus GN=Cox7b PE=2 SV=1
RPL32	P62911	8	8	90049.12	111149.88	127552.25	134205.5	98586.5	107261.88	119587	246064.25	135162	164354.5	0.169244323	0.77148589	112308.65	154485.93	Up	0.460006363263347	60S ribosomal protein L32 OS=Mus musculus GN=Rpl32 PE=1 SV=2
8030462N17RIK	Q8BH50	2	2	1511.5	1369.5	1479	2722	2766.5	4055	1582	2913	5865	NA	0.169411995	0.771055844	1969.7	3603.75	Up	0.871523018521056	Uncharacterized protein C18orf25 homolog OS=Mus musculus PE=2 SV=1
MAPK3	Q63844	4	4	48662.75	84265.5	74514	92176	91810.75	101851.5	119431	81589.5	88243.75	82024.25	0.169786958	0.770095673	78285.8	94628	Up	0.273516488106311	Mitogen-activated protein kinase 3 OS=Mus musculus GN=Mapk3 PE=1 SV=5
UBQLN4	Q99NB8	1	1	3257	5533	7897	4131	3516	7599	7777	NA	5064	NA	0.169813809	0.770026998	4866.8	6813.3333	Up	0.485387301820151	Ubiquilin-4 OS=Mus musculus GN=Ubqln4 PE=1 SV=1
ROBO2	Q7TPD3	4	4	46008.33	46375.33	66198.67	44275	55143	45200	55579	2556.67	51209.67	4128.67	0.170690386	0.767790939	51600.066	31734.802	Down	-0.70130706957924	Roundabout homolog 2 OS=Mus musculus GN=Robo2 PE=2 SV=2
ROBO2	Q7TPD3-2	3	3	46008.33	46375.33	66198.67	44275	55143	45200	55579	2556.67	51209.67	4128.67	0.170690386	0.767790939	51600.066	31734.802	Down	-0.70130706957924	Isoform 2 of Roundabout homolog 2 OS=Mus musculus GN=Robo2
TMED10	Q9D1D4	1	1	35266	35750	56252	25115	36675	40852	50688	86976	36537	146863	0.171257182	0.766351206	37811.6	72383.2	Up	0.936825991107516	Transmembrane emp24 domain-containing protein 10 OS=Mus musculus GN=Tmed10 PE=2 SV=1
PRNP	P04925	1	1	6804	6673	NA	8265	6764	11031	7164	NA	10889	NA	0.17150748	0.765716934	7126.5	9694.6667	Up	0.443997587291833	Major prion protein OS=Mus musculus GN=Prnp PE=1 SV=2
RPL22L1	Q9D7S7	4	4	14596.67	20712	7330	21741	15887.67	18122.33	21782.33	20918.33	20714.67	NA	0.171621413	0.765428526	16053.468	20384.415	Up	0.344581560720124	60S ribosomal protein L22-like 1 OS=Mus musculus GN=Rpl22l1 PE=1 SV=1
SHANK3	Q4ACU6-12	9	9	76433	58952.22	96090.78	36531.22	64890.56	34807.11	43490.33	79315.67	50355.56	21727.44	0.171753816	0.765093605	66579.556	45939.222	Down	-0.53535282531314	Isoform 10 of SH3 and multiple ankyrin repeat domains protein 3 OS=Mus musculus GN=Shank3
IGFBP4	P47879	1	1	3275	4086	NA	5247	3369	3825	2692	NA	1824	NA	0.171826678	0.764909405	3994.25	2780.3333	Down	-0.522666776255966	Insulin-like growth factor-binding protein 4 OS=Mus musculus GN=Igfbp4 PE=2 SV=2
ST6GAL2	Q76K27	1	1	1008	2724	3342	1924	2543	3276	2809	NA	4104	8709	0.171887475	0.764755767	2308.2	4724.5	Up	1.03339341964535	Beta-galactoside alpha-2,6-sialyltransferase 2 OS=Mus musculus GN=St6gal2 PE=2 SV=2
KIF3C	O35066	1	1	NA	NA	NA	1394	1401	1701	1934	NA	NA	NA	0.171974904	0.764534923	1397.5	1817.5	Up	0.379107081076404	Kinesin-like protein KIF3C OS=Mus musculus GN=Kif3c PE=1 SV=3
CHMP4B	Q9D8B3	10	10	30292.7	35572.5	38076.1	48501.4	36543.1	41216.4	32801.1	29995	31034.7	17177.6	0.172476599	0.76326982	37797.16	30444.96	Down	-0.312074419949393	Charged multivesicular body protein 4b OS=Mus musculus GN=Chmp4b PE=1 SV=2
PHACTR3	Q8BYK5-3	1	1	22840	20408	7024	15961	26754	25707	20519	NA	28958	NA	0.173019626	0.76190463	18597.4	25061.333	Up	0.430362231942252	Isoform 3 of Phosphatase and actin regulator 3 OS=Mus musculus GN=Phactr3
DDX3Y	Q62095	4	4	28490	32065.75	43941.75	22102.75	33341.75	32555.75	31617.5	44420.25	38505	57866.25	0.17396805	0.759530504	31988.4	40992.95	Up	0.35782698188054	ATP-dependent RNA helicase DDX3Y OS=Mus musculus GN=Ddx3y PE=1 SV=2
RABGEF1	Q9JM13	3	3	7374	10107.33	11639.67	6285	10836	9179.67	11226	9390	12588.67	16371.67	0.174456895	0.75831186	9248.4	11751.202	Up	0.345532631968794	Rab5 GDP/GTP exchange factor OS=Mus musculus GN=Rabgef1 PE=1 SV=1
SAMD14	Q8K070	4	4	14169.25	14999.75	16137.5	7544.25	13780.25	13186.25	14416.75	13585.25	24131.25	22890	0.174464052	0.758294044	13326.2	17641.9	Up	0.404740493959794	Sterile alpha motif domain-containing protein 14 OS=Mus musculus GN=Samd14 PE=1 SV=1
RPLP0	P14869	5	5	17292	22394	24100.2	25171.6	18689.4	20527.8	20695.8	2173.8	22726	2708	0.174626531	0.757889774	21529.44	13766.28	Down	-0.645172035066324	60S acidic ribosomal protein P0 OS=Mus musculus GN=Rplp0 PE=1 SV=3
SORT1	Q6PHU5	1	1	4574	9933	10096	12894	7843	15059	18228	7654	11974	NA	0.17486773	0.757290328	9068	13228.75	Up	0.544820449930194	Sortilin OS=Mus musculus GN=Sort1 PE=1 SV=1
PALMD	Q9JHU2	5	5	33665.2	31149	53691	19327.6	31777.4	30621	35319.2	68501.8	49511.8	117103	0.174905977	0.757195349	33922.04	60211.36	Up	0.827812770937125	Palmdelphin OS=Mus musculus GN=Palmd PE=1 SV=1
PDZRN3	Q69ZS0-3	1	1	1175	NA	NA	2598	2407	3283	3056	NA	2506	NA	0.175006348	0.756946198	2060	2948.3333	Up	0.517255304786084	Isoform 3 of E3 ubiquitin-protein ligase PDZRN3 OS=Mus musculus GN=Pdzrn3
CLVS1	Q9D4C9	2	2	6766	9808.5	2100	18721.5	12655	20671	18378	8189	16348	NA	0.175176859	0.756523264	10010.2	15896.5	Up	0.667238357209586	Clavesin-1 OS=Mus musculus GN=Clvs1 PE=2 SV=1
SCHIP1	Q3TI53-3	1	1	675	NA	NA	1278	774	1647	3014	NA	1251	NA	0.176124898	0.754179245	909	1970.6667	Up	1.11633156923089	Isoform 3 of Schwannomin-interacting protein 1 OS=Mus musculus GN=Schip1
CREG2	Q8BGC9	1	1	17568	22024	34093	19192	25304	31720	28606	46230	35865	17495	0.176503453	0.753246793	23636.2	31983.2	Up	0.436316179744669	Protein CREG2 OS=Mus musculus GN=Creg2 PE=1 SV=1

POLR1C	P52432	1	1	1122	1502	1035	768	740	2052	1092	NA	1588	NA	0.176571899	0.753078411	1033.4	1577.3333	Up	0.610088784081134	DNA-directed RNA polymerases I and III subunit RPAC1 OS=Mus musculus GN=Polr1c PE=1 SV=3
MBP	P04370-9	52	52	921470.19	737288.62	838126.85	400264.9	778637.73	575824.77	367873.04	663242.13	484414.5	751390.52	0.176756115	0.752625552	735157.66	568548.99	Down	-0.370769003987721	Isoform 9 of Myelin basic protein OS=Mus musculus GN=Mbp
MAP7D1	A2AJI0	8	8	22287.38	28013.25	31569.38	17687.38	23877.38	23943.38	25684.75	26002.5	41591.88	42386.12	0.176783777	0.752557591	24686.954	31921.726	Up	0.370789819317657	MAP7 domain-containing protein 1 OS=Mus musculus GN=Map7d1 PE=1 SV=1
PARK7	Q99LX0	23	23	101470.91	94860.74	122800.87	72162.52	110343	92741.3	101963.52	130563.17	134826.3	131308.04	0.177155961	0.751644231	100327.61	118280.47	Up	0.237493173264741	Protein DJ-1 OS=Mus musculus GN=Park7 PE=1 SV=1
CRTAC1	Q8R555	4	4	18512.25	21696.75	5580.75	32337	24357.25	25828.75	27863.25	NA	29416	NA	0.177368788	0.7511228	20496.8	27702.667	Up	0.434626166223699	Cartilage acidic protein 1 OS=Mus musculus GN=Crtac1 PE=2 SV=1
YIPF5	Q9EQQ2	1	1	3084	6674	2623	8396	3456	7755	5771	NA	6918	NA	0.177483899	0.750841038	4846.6	6814.6667	Up	0.491670071828236	Protein YIPF5 OS=Mus musculus GN=Yipf5 PE=2 SV=1
PPFIBP1	Q8C8U0-3	1	1	1053	1517	NA	2712	2502	2502	2734	NA	2680	NA	0.17772506	0.750251331	1946	2638.6667	Up	0.439297401545244	Isoform 3 of Liprin-beta-1 OS=Mus musculus GN=Ppfibp1
GAK	Q99KY4	3	3	11364.33	15376.67	8050	19208.67	14969.67	16029.67	22459.33	13459	19757.67	NA	0.178077102	0.74939192	13793.868	17926.418	Up	0.378060135886232	Cyclin-G-associated kinase OS=Mus musculus GN=Gak PE=1 SV=2
COPG2	Q9QXK3	1	1	1126	NA	NA	2250	1862	2853	4116	NA	1965	NA	0.178153993	0.74920444	1746	2978	Up	0.77029019495266	Coatomer subunit gamma-2 OS=Mus musculus GN=Copg2 PE=2 SV=1
NIT2	Q9JHW2	5	5	69663.6	82252.2	110192.2	45209.2	90009.6	70016.4	84775.8	146528.8	109926.8	246372	0.179635946	0.745606754	79465.36	131523.96	Up	0.726927629571719	Omega-amidase NIT2 OS=Mus musculus GN=Nit2 PE=1 SV=1
HSPA4L	P48722	23	23	27865.17	33790.39	47970.48	38190.17	42321.13	42497.09	40573.91	80059.48	39769.39	51149.13	0.179678535	0.745503802	38027.468	50809.8	Up	0.418064902786813	Heat shock 70 kDa protein 4L OS=Mus musculus GN=Hspa4l PE=1 SV=2
HSPA4L	P48722-2	21	21	30354.43	36872.29	52539.1	41502.33	46058.48	46284.48	44320.81	87684.19	43318.33	56020.48	0.179799686	0.745211072	41465.326	55525.658	Up	0.421249149520579	Isoform 2 of Heat shock 70 kDa protein 4L OS=Mus musculus GN=Hspa4l
CELF1	P28659-3	5	5	53820	42702.8	66854.6	27034.8	49362.4	38668.2	48822.2	87055.4	64819.4	95281.2	0.179841407	0.745110309	47954.92	66929.28	Up	0.480958656326992	Isoform 3 of CUGBP Elav-like family member 1 OS=Mus musculus GN=Celf1
AGK	Q9ESW4	2	2	60621	93186	101342.5	38275	77111	61738.5	71753.5	339147.5	90086.5	302139.5	0.180214133	0.744211152	74107.1	172973.1	Up	1.22286401889041	Acylglycerol kinase, mitochondrial OS=Mus musculus GN=Agk PE=1 SV=1
RPS14	P62264	17	17	113748.88	119058	163374.53	48799	103768.71	83179.06	108922.88	197610.47	179702.82	209939.06	0.180930638	0.742487885	109749.82	155870.86	Up	0.506132597236003	40S ribosomal protein S14 OS=Mus musculus GN=Rps14 PE=2 SV=3
EGFR	Q01279	1	1	12270	13250	NA	14630	11980	16211	13010	NA	15710	NA	0.181182112	0.741884683	13032.5	14977	Up	0.200634810687378	Epidermal growth factor receptor OS=Mus musculus GN=Egfr PE=1 SV=1
AK1	Q9R0Y5	36	36	38576.36	41173.03	49954.22	44154.64	40731.78	41756.81	42044.36	57938.89	47157.69	53782.75	0.181641254	0.740785509	42918.006	48536.1	Up	0.177475139852808	Adenylate kinase isoenzyme 1 OS=Mus musculus GN=Ak1 PE=1 SV=1
NDUFS7	Q9DC70	11	11	95428.55	88691.36	94206.36	101586.18	76457.55	94419.09	72498.09	92043.45	67809.55	80175.36	0.181865798	0.740248967	91274	81389.108	Down	-0.165368220620973	NADH dehydrogenase [ubiquinone] iron-sulfur protein 7, mitochondrial OS=Mus musculus GN=Ndufs7 PE=1 SV=1
WIPF3	P0C7L0	6	6	43406.17	41246.83	49575.83	26055	50830.17	42710	47663.5	39599.33	80829.67	70078.33	0.181955681	0.740034382	42222.8	56176.166	Up	0.411935910477937	WAS/WASL-interacting protein family member 3 OS=Mus musculus GN=Wipf3 PE=1 SV=1
MTFR1L	Q9CWE0-2	3	3	4173	1853	5054	2825.67	3266	3026.67	2779.67	13508.33	4991	10053.67	0.181957616	0.740029762	3434.334	6871.868	Up	1.00067197148849	Isoform 2 of Mitochondrial fission regulator 1-like OS=Mus musculus GN=Mtfr1l
FAU	P62862	2	2	121460	117424.5	197528.5	86384	123676.5	121063.5	147768	176339	179515.5	331451	0.182262982	0.739301528	129294.7	191227.4	Up	0.564626116608293	40S ribosomal protein S30 OS=Mus musculus GN=Fau PE=1 SV=1
STRN4	P58404	26	26	23230.7	26391.35	25077.78	24354.43	28283.78	28131.78	31312.74	21891.87	32837.17	28908.3	0.182342689	0.739111645	25467.608	28616.372	Up	0.168177314558885	Striatin-4 OS=Mus musculus GN=Strn4 PE=1 SV=2
CEP170	Q6A065	17	17	36966	38454.88	43027.06	26425.94	35150.35	30802.41	37618.41	42193.53	56299.35	63261.59	0.182411726	0.738947247	36004.846	46035.058	Up	0.354541867924025	Centrosomal protein of 170 kDa OS=Mus musculus GN=Cep170 PE=1 SV=2
DYNLRB1	P62627	12	12	41529.92	39811.58	55187.5	27185	38032.17	36084.67	37993.08	87115.08	49076.33	102270.33	0.182525416	0.738676654	40349.234	62507.898	Up	0.631497203465766	Dynein light chain roadblock-type 1 OS=Mus musculus GN=Dynlrb1 PE=1 SV=3
WARS	P32921	3	3	43239.33	50676	39006	30838.67	51153.67	43316.33	44205	51346.33	52021.67	59151.67	0.182531475	0.738662236	42982.734	50008.2	Up	0.218407426379772	Tryptophan--tRNA ligase, cytoplasmic OS=Mus musculus GN=Wars PE=1 SV=2
ACTR1B	Q8R5C5	7	7	74225.29	80287.29	84242.86	50362.43	72880.57	69224.43	74811	115183.14	86871.14	177401.71	0.182866539	0.737865754	72399.688	104698.28	Up	0.532182411489075	Beta-centractin OS=Mus musculus GN=Actr1b PE=1 SV=1
DNAJB11	Q99KV1	2	2	12922	12777	11887.5	17404	11281.5	15648	17080	46556	23098	NA	0.183355693	0.736705602	13254.4	25595.5	Up	0.949418825412355	DnaJ homolog subfamily B member 11 OS=Mus musculus GN=Dnajb11 PE=1 SV=1
LMNB1	P14733	14	14	47886.57	25093.21	22400.93	19472.5	37045.57	29359.64	23403.14	16198.71	24209.14	13617.21	0.183382325	0.736642526	30379.756	21357.568	Down	-0.508362906878114	Lamin-B1 OS=Mus musculus GN=Lmnb1 PE=1 SV=3
SEPT9	Q80UG5	33	33	35825.12	36705.61	37271.55	34012.61	37767.58	39912.12	41650.15	48056.45	47984.03	30303.73	0.183535766	0.73627929	36316.494	41581.296	Up	0.195309793266775	Septin-9 OS=Mus musculus GN=Sept9 PE=1 SV=1
PDZD11	Q9CZG9	1	1	26148	26660	26667	18347	25412	20732	31621	51012	30506	29793	0.183849433	0.735537706	24646.8	32732.8	Up	0.409336669931888	PDZ domain-containing protein 11 OS=Mus musculus GN=Pdzd11 PE=1 SV=1
MAPK10	Q61831	1	1	52088	65286	133423	54579	59169	55870	92014	96393	134827	149932	0.184325359	0.734414911	72909	105807.2	Up	0.537268984565941	Mitogen-activated protein kinase 10 OS=Mus musculus GN=Mapk10 PE=1 SV=2
AHCYL2	Q68FL4-2	10	10	56364.25	80882.25	69854.75	112109.5	77557.5	103593.62	92345.12	95573	81757.62	99320.5	0.184349711	0.734357539	79353.65	94517.972	Up	0.252292091149292	Isoform 2 of Putative adenosylhomocysteinase 3 OS=Mus musculus GN=Ahcyl2
UBAP2L	Q80X50-3	6	6	19092.4	21564.6	34062.8	31599	19190.2	23043.8	25513.4	42116.8	32984.8	66327	0.184426632	0.734176365	25101.8	37997.16	Up	0.598100771386758	Isoform 3 of Ubiquitin-associated protein 2-like OS=Mus musculus GN=Ubap2l
BSN	O88737-2	208	208	29022.89	31533.01	31338.04	42026.5	39841.92	43026.17	42033.07	31790.31	45066.1	37356.55	0.184795104	0.73330954	34752.472	39854.44	Up	0.197624854456625	Isoform 2 of Protein bassoon OS=Mus musculus GN=Bsn
RALGPS1	A2AR50	1	1	6136	13864	2594	20321	11495	22534	15812	13456	13796	NA	0.184892615	0.733080436	10882	16399.5	Up	0.591708096057542	Ras-specific guanine nucleotide-releasing factor RalGPS1 OS=Mus musculus GN=Ralgps1 PE=1 SV=2
GCSH	Q91WK5	6	6	25910	22219.83	25834.5	17127.5	16539	17455.33	20427.67	43498.5	27935.5	46387.17	0.185064951	0.732675824	21526.166	31140.834	Up	0.532716196876948	Glycine cleavage system H protein, mitochondrial OS=Mus musculus GN=Gcsh PE=1 SV=2
AK5	Q920P5	9	9	25105	29205.33	32703.44	24691.44	31846.78	29665.56	33095.67	37506	38583.11	25930.89	0.185108682	0.73257321	28710.398	32956.246	Up	0.19897858635119	Adenylate kinase isoenzyme 5 OS=Mus musculus GN=Ak5 PE=2 SV=2
MBP	P04370-10	53	53	876604.28	700396.89	804921.85	371323.13	744231.98	548957.87	347263.81	628754.15	461204.87	718682.62	0.185273628	0.732186395	699495.63	540972.66	Down	-0.370759341358464	Isoform 10 of Myelin basic protein OS=Mus musculus GN=Mbp
RACK1	P68040	2	2	16142	17628	16957	20031	16086	27360	18560	NA	21298	NA	0.18540447	0.731879799	17368.8	22406	Up	0.367387034154744	Guanine nucleotide-binding protein subunit beta-2-like 1 OS=Mus musculus GN=Gnb2l1 PE=1 SV=3
CHCHD4	Q8VEA4	2	2	122799.5	114996.5	120030	138912.5	118151	121370	131133	29141.5	116542.5	48158	0.185583161	0.731461433	122977.9	89269	Down	-0.462167906412675	Mitochondrial intermembrane space import and assembly protein 40 OS=Mus musculus GN=Chchd4 PE=1 SV=1
CLDN12	Q9ET43	1	1	5127	10751	NA	16773	11831	17164	14128	NA	NA	NA	0.185680876	0.731232824	11120.5	15646	Up	0.492572214095589	Claudin-12 OS=Mus musculus GN=Cldn12 PE=2 SV=2
DUS3L	Q91XI1	2	2	30197	33481	12322.5	27259	34563.5	43374.5	40557	21658	61264.5	NA	0.18569966	0.731188892	27564.6	41713.5	Up	0.59769770152358	tRNA-dihydrouridine(47) synthase [NAD(P)(+)]-like OS=Mus musculus GN=Dus3l PE=2 SV=1
STRIP1	Q8C079-2	1	1	34882	37556	46211	74994	49764	63043	63229	46360	80749	NA	0.186649275	0.728973692	48681.4	63345.25	Up	0.379865783375333	Isoform 2 of Striatin-interacting protein 1 OS=Mus musculus GN=Strip1
STMN3	O70166	3	3	79346.67	75028	67847.67	79229.67	76585	82492.67	70070.67	47965.33	73280	44466.33	0.186675244	0.728913272	75607.402	63655	Down	-0.248253641878632	Stathmin-3 OS=Mus musculus GN=Stmn3 PE=1 SV=1
FTH1	P09528	3	3	78574.33	86359	109932.33	124570.67	80737.67	98748.33	93530.67	167333.33	117552	118916.33	0.186687203	0.728885451	96034.8	119216.13	Up	0.31195027741356	Ferritin heavy chain OS=Mus musculus GN=Fth1 PE=1 SV=2
PFKP	Q9WUA3-2	8	8	23578	22803.25	45409.62	26404.5	27094	28139	31232.5	57831.12	29392.88	63164	0.187241137	0.72759873	29057.874	41951.9	Up	0.529806998739766	Isoform 2 of 6-phosphofructokinase type C OS=Mus musculus GN=Pfkp
CEP97	Q9CZ62	1	1	2144	2305	2210	4813	5128	5725	5614	7532	2259	NA	0.187256218	0.727563751	3320	5282.5	Up	0.670037620588016	Centrosomal protein of 97 kDa OS=Mus musculus GN=Cep97 PE=2 SV=1
CLU	Q06890	8	8	42873.12	33054.25	39756.38	27938.38	36926.62	38194.88	36585.25	70927.88	44024.25	42192.88	0.187446666	0.727122279	36109.75	46385.028	Up	0.361270780284682	Clusterin OS=Mus musculus GN=Clu PE=1 SV=1
RPL9	P51410	12	12	88742.42	101579.75	116285.42	80715.92	82234.58	78013.33	96804.25	214271.25	122134.67	149325.17	0.187462382	0.727085869	93911.618	132109.73	Up	0.492361217149813	60S ribosomal protein L9 OS=Mus musculus GN=Rpl9 PE=1 SV=2
MARK4	Q8CIP4	1	1	50200	55162	NA	72379	65840	66219	69369	145724	87751	NA	0.18747422	0.727058446	60895.25	92265.75	Up	0.599465505668313	MAP/microtubule affinity-regulating kinase 4 OS=Mus musculus GN=Mark4 PE=1 SV=1
AK2	Q9WTP6-2	16	16	66687.19	69469.62	69633.56	48786.19	67050.81	61800.69	68672.81	81099.44	74875.06	71869.19	0.187553737	0.726874278	64325.474	71663.438	Up	0.155847074533067	Isoform 2 of Adenylate kinase 2, mitochondrial OS=Mus musculus GN=Ak2
CPSF6	Q6NVF9	5	5	22465.6	15277.2	23665	17715.2	18375	16281.6	19263.4	5811.8	22220	4655.4	0.187798096	0.726308816	19499.6	13646.44	Down	-0.514919891178192	Cleavage and polyadenylation specificity factor subunit 6 OS=Mus musculus GN=Cpsf6 PE=2 SV=1
NLGN2	Q69ZK9	4	4	12097	13105.25	24088.25	19439.75	16011.75	20645.75	23040.75	42838.75	24175.75	13831.75	0.187806972	0.726288289	16948.4	24906.55	Up	0.555376113173601	Neuroligin-2 OS=Mus musculus GN=Nlgn2 PE=1 SV=2
PLIN3	Q9DBG5	1	1	NA	5232	NA	10646	1849	10263	8119	NA	15260	NA	0.188098568	0.725614511	5909	11214	Up	0.924315070892126	Perilipin-3 OS=Mus musculus GN=Plin3 PE=1 SV=1
NTRK2	P15209-4	5	5	17140.4	23953.6	19728	28359.6	23796.4	31034.8	27427.8	36394.6	26312	94099.6	0.18833414	0.725070946	22595.6	43053.76	Up	0.930097370926078	Isoform L10 of BDNF/NT-3 growth factors receptor OS=Mus musculus GN=Ntrk2
MARCKS	P26645	25	25	107122.13	99466.04	138065.57	86753.26	94467.04	96106.65	105170.04	146023.57	127617.09	155015.3	0.188434034	0.724840653	105174.81	125986.53	Up	0.260480310087908	Myristoylated alanine-rich C-kinase substrate OS=Mus musculus GN=Marcks PE=1 SV=2
GABRA4	Q9D6F4	1	1	20352	33742	23511	35634	32131	29794	37141	NA	38266	NA	0.188464196	0.724771144	29074	35067	Up	0.270384441495108	Gamma-aminobutyric acid receptor subunit alpha-4 OS=Mus musculus GN=Gabra4 PE=2 SV=1
REPS1	O54916-4	1	1	53900	62876	71857	48497	60237	61635	64514	93716	63230	151909	0.188770698	0.724065418	59473.4	87000.8	Up	0.548784112401425	Isoform 4 of RalBP1-associated Eps domain-containing protein 1 OS=Mus musculus GN=Reps1
TRAP1	Q9CQN1	2	2	16942.5	15452	5560	12641	9512.5	15505	15639.5	NA	14685.5	NA	0.189134653	0.723228893	12021.6	15276.667	Up	0.345700862448823	Heat shock protein 75 kDa, mitochondrial OS=Mus musculus GN=Trap1 PE=1 SV=1
EPB41L3	Q9WV92-8	37	37	21839.54	24829.97	22554.11	27985.41	24193.05	29908.41	28653.49	20873.3	27711.54	28200.95	0.18930405	0.722840094	24280.416	27069.538	Up	0.15687712534783	Isoform 8 of Band 4.1-like protein 3 OS=Mus musculus GN=Epb41l3
CXCL12	P40224	1	1	26632	25731	17269	22602	22207	34188	23052	NA	40455	NA	0.189384995	0.722654433	22888.2	32565	Up	0.508718217392811	Stromal cell-derived factor 1 OS=Mus musculus GN=Cxcl12 PE=2 SV=2
TMX3	Q8BXZ1	2	2	10330	10033	15126.5	13534.5	11227.5	12147	14011	NA	13721.5	18985.5	0.189418566	0.722577455	12050.3	14716.25	Up	0.288341026441884	Protein disulfide-isomerase TMX3 OS=Mus musculus GN=Tmx3 PE=1 SV=2
MKL2	P59759	4	4	20812.25	19181.25	12616.5	20709.25	24886.5	24416.25	23957.75	20383	40441.75	NA	0.189721994	0.721882319	19641.15	27299.688	Up	0.475005033980495	MKL/myocardin-like protein 2 OS=Mus musculus GN=Mkl2 PE=1 SV=1
PTPRN	Q60673	1	1	45424	35902	25791	54198	57428	58080	47563	NA	80048	NA	0.190353384	0.720439399	43748.6	61897	Up	0.500632637180423	Receptor-type tyrosine-protein phosphatase-like N OS=Mus musculus GN=Ptprn PE=1 SV=2
IGSF21	Q7TNR6	6	6	26683	30409.67	42774	35560	31875.17	36686.83	35905.33	64829.33	29085.5	55120.33	0.190398266	0.720337011	33460.368	44325.464	Up	0.405682421021199	Immunoglobulin superfamily member 21 OS=Mus musculus GN=Igsf21 PE=2 SV=1
PDPK1	Q9Z2A0	3	3	21562	24041	39220	16307.67	26278.67	23115	29922.67	55677	28038.33	88856.33	0.190932185	0.719120858	25481.868	45121.866	Up	0.824355689995612	3-phosphoinositide-dependent protein kinase 1 OS=Mus musculus GN=Pdpk1 PE=1 SV=2
DHRS1	Q99L04	3	3	48414.67	60798.33	60490.33	37936.33	58236.33	58705.67	58525	76864.67	87467.67	46532.67	0.190972901	0.719028255	53175.198	65619.136	Up	0.303363098044015	Dehydrogenase/reductase SDR family member 1 OS=Mus musculus GN=Dhrs1 PE=2 SV=1
RGS7BP	Q8BQP9	1	1	208474	194407	238798	104015	241077	182749	215105	318069	221219	318539	0.191659993	0.717468531	197354.2	251136.2	Up	0.347682778396812	Regulator of G-protein signaling 7-binding protein OS=Mus musculus GN=Rgs7bp PE=1 SV=1
XPNPEP1	Q6P1B1	2	2	5831	7254.5	15067	7055	6634.5	7428.5	9688	12179.5	11945.5	19575	0.191799144	0.717153335	8368.4	12163.3	Up	0.539510978996114	Xaa-Pro aminopeptidase 1 OS=Mus musculus GN=Xpnpep1 PE=2 SV=1
PFN2	Q9JJV2-2	28	28	87586.33	83814.56	129338.19	52923.48	86996.48	80700.11	85865.22	135191.04	116803.56	184396.19	0.191913076	0.716895434	88131.808	120591.22	Up	0.452390212215575	Isoform 2 of Profilin-2 OS=Mus musculus GN=Pfn2
PAK2	Q8CIN4	8	8	20680.25	28082.62	28466.62	45845.38	24969.12	36192.75	31452.25	48752.38	27870.88	45669.75	0.192202558	0.716240836	29608.798	37987.602	Up	0.359502719517731	Serine/threonine-protein kinase PAK 2 OS=Mus musculus GN=Pak2 PE=1 SV=1
MBP	P04370	43	43	1030891	820995	949791.35	434537.12	867750.56	644420.74	397575.4	736936.35	531255.21	859740.93	0.192231663	0.716175077	820793.01	633985.73	Down	-0.372568078872115	Myelin basic protein OS=Mus musculus GN=Mbp PE=1 SV=2
HGS	Q99LI8	11	11	31251	33523.4	29861.8	46262.9	38860.7	40908.9	43864	45196.6	47796	31384.7	0.192427091	0.715733786	35951.96	41830.04	Up	0.218468953065682	Hepatocyte growth factor-regulated tyrosine kinase substrate OS=Mus musculus GN=Hgs PE=1 SV=2
UAP1L1	Q3TW96	1	1	2837	7004	NA	9095	7456	9532	8290	NA	NA	NA	0.192483376	0.715606774	6598	8911	Up	0.433558563394031	UDP-N-acetylhexosamine pyrophosphorylase-like protein 1 OS=Mus musculus GN=Uap1l1 PE=2 SV=1
ZFYVE1	Q810J8	1	1	25662	25643	57409	18525	20330	34575	34471	35270	52936	50218	0.192701669	0.715114523	29513.8	41494	Up	0.491513054446109	Zinc finger FYVE domain-containing protein 1 OS=Mus musculus GN=Zfyve1 PE=2 SV=2
VDAC1	Q60932-2	136	136	86736.41	81524.78	78852.62	67441.04	83606.75	77747.55	84476.84	85708.82	85275.61	98892.82	0.192748818	0.715008277	79632.32	86420.328	Up	0.118016616983241	Isoform Mt-VDAC1 of Voltage-dependent anion-selective channel protein 1 OS=Mus musculus GN=Vdac1
TACC2	Q9JJG0-3	1	1	8621	10797	NA	17338	11615	13711	14708	NA	18973	NA	0.192905319	0.714655797	12092.75	15797.333	Up	0.385538681306661	Isoform 3 of Transforming acidic coiled-coil-containing protein 2 OS=Mus musculus GN=Tacc2
VDAC1	Q60932	137	137	87172.17	81788.95	79229.93	67377.13	83756.95	77747.56	84610.44	86550.38	85636.12	100502.34	0.192919646	0.714623545	79865.026	87009.368	Up	0.123606874023756	Voltage-dependent anion-selective channel protein 1 OS=Mus musculus GN=Vdac1 PE=1 SV=3
SNX5	Q9D8U8	2	2	10041	13085	11486	18233	11010.5	12954	10518	7524	9494	NA	0.193524357	0.713264367	12771.1	10122.5	Down	-0.335317149543794	Sorting nexin-5 OS=Mus musculus GN=Snx5 PE=1 SV=1
PPP1R12B	Q8BG95	6	6	18046.5	18759	12331.33	10629.33	17573.17	15352.33	20274.5	12992.83	25216.67	24796.33	0.19386112	0.712509283	15467.866	19726.532	Up	0.350863175685621	Protein phosphatase 1 regulatory subunit 12B OS=Mus musculus GN=Ppp1r12b PE=1 SV=2
CRMP1	P97427	88	88	157581.2	176934.24	191148.55	192122.4	157717.29	189926.81	197842.89	187219.93	196940.11	169484.31	0.193906332	0.712408009	175100.74	188282.81	Up	0.104716141740608	Dihydropyrimidinase-related protein 1 OS=Mus musculus GN=Crmp1 PE=1 SV=1
NAP1L4	Q78ZA7	4	4	18488	19318.5	18744.75	23742	19029.75	20006.5	18966.5	18842.25	17075.25	11831.75	0.194136441	0.711892936	19864.6	17344.45	Down	-0.195725650278463	Nucleosome assembly protein 1-like 4 OS=Mus musculus GN=Nap1l4 PE=1 SV=1
CST3	P21460	15	15	247733.6	228367.8	357167.87	107472.87	201207	182440.33	196124.6	416292.8	392233.93	467867.27	0.194337452	0.711443496	228389.83	330991.79	Up	0.535297017541448	Cystatin-C OS=Mus musculus GN=Cst3 PE=2 SV=2
EFL1	Q8C0D5	1	1	4561	2819	2478	4195	6082	5507	4532	NA	8215	NA	0.194629209	0.710791982	4027	6084.6667	Up	0.59547275835967	Elongation factor Tu GTP-binding domain-containing protein 1 OS=Mus musculus GN=Eftud1 PE=2 SV=1
SRP54	P14576	1	1	8546	12396	5689	9271	11597	12976	10125	NA	17672	NA	0.194633825	0.710781682	9499.8	13591	Up	0.516682565065893	Signal recognition particle 54 kDa protein OS=Mus musculus GN=Srp54 PE=1 SV=2
USP14	Q9JMA1	1	1	18742	18701	8031	26069	24681	23967	33008	19650	24632	NA	0.194735894	0.710553992	19244.8	25314.25	Up	0.395481063179095	Ubiquitin carboxyl-terminal hydrolase 14 OS=Mus musculus GN=Usp14 PE=1 SV=3
ASRGL1	Q8C0M9	20	20	95612.2	96104.45	109877.7	66028.55	95582.2	83172.4	89800.55	148545.65	107440.25	155409.4	0.195514145	0.708821817	92641.02	116873.65	Up	0.335226657671069	Isoaspartyl peptidase/L-asparaginase OS=Mus musculus GN=Asrgl1 PE=1 SV=1
GFAP	P03995-2	27	27	66655	62682.42	65564.77	54974.85	65067	61809.62	58631.69	77232.04	71243.69	82833.65	0.195590743	0.708651704	62988.808	70350.138	Up	0.159457743553894	Isoform 2 of Glial fibrillary acidic protein OS=Mus musculus GN=Gfap
IRGQ	Q8VIM9	1	1	12093	9831	NA	21664	11112	22504	18670	NA	15196	NA	0.196125841	0.70746518	13675	18790	Up	0.458424233740137	Immunity-related GTPase family Q protein OS=Mus musculus GN=Irgq PE=2 SV=1
TARDBP	Q921F2	6	6	21053.17	19027.5	18107.33	27839.5	17546.5	23276.17	16745.67	3699.33	21700.33	4606.33	0.196284395	0.707114227	20714.8	14005.566	Down	-0.564661603750359	TAR DNA-binding protein 43 OS=Mus musculus GN=Tardbp PE=1 SV=1
RBM3	O89086	3	3	238346	215517	177528.33	166695.67	167129.67	168178	253172.67	324406.67	472659.33	181906	0.196363129	0.706940057	193043.33	280064.53	Up	0.536834561866688	Putative RNA-binding protein 3 OS=Mus musculus GN=Rbm3 PE=2 SV=1
FAM163B	Q8BUM6	1	1	1352	2754	3859	7859	3954	7999	8459	NA	3833	NA	0.196645687	0.706315574	3955.6	6763.6667	Up	0.773909016654056	Protein FAM163B OS=Mus musculus GN=Fam163b PE=2 SV=1
MRPL40	Q9Z2Q5	2	2	9013	10031	NA	13601	11366.5	11597.5	13139	NA	16470	NA	0.196710583	0.706172274	11002.875	13735.5	Up	0.320028885884455	39S ribosomal protein L40, mitochondrial OS=Mus musculus GN=Mrpl40 PE=2 SV=2
CD34	Q64314-2	1	1	4902	5740	5564	3423	5862	6765	6408	NA	4947	11182	0.1974355	0.704574756	5098.2	7325.5	Up	0.5229392629297	Isoform Short of Hematopoietic progenitor cell antigen CD34 OS=Mus musculus GN=Cd34
ABHD10	Q6PE15	2	2	12526	18170	31442.5	32170	21073.5	31302	28720.5	39929.5	22406.5	NA	0.197455322	0.704531156	23076.4	30589.625	Up	0.406624244912515	Mycophenolic acid acyl-glucuronide esterase, mitochondrial OS=Mus musculus GN=Abhd10 PE=2 SV=1
SNRPF	P62307	1	1	9161	8113	8231	10271	9017	7775	7949	6797	9425	NA	0.197462928	0.704514428	8958.6	7986.5	Down	-0.165709897915005	Small nuclear ribonucleoprotein F OS=Mus musculus GN=Snrpf PE=2 SV=1
AHCYL2	Q68FL4	15	15	39823.25	56295.17	47486	77982.92	54927	73324.25	65431.42	64113.08	58021.67	66213.67	0.197527364	0.704372733	55302.868	65420.818	Up	0.242395498119915	Putative adenosylhomocysteinase 3 OS=Mus musculus GN=Ahcyl2 PE=1 SV=1
CTNNA2	Q61301	22	22	29399	35454.12	47648.06	59864.76	38363.41	47182.82	47536.29	64083.59	44135	53267.41	0.197638923	0.704127522	42145.87	51241.022	Up	0.28190798637085	Catenin alpha-2 OS=Mus musculus GN=Ctnna2 PE=1 SV=3
SORBS2	Q3UTJ2-4	7	7	26694.29	24361.57	51543.43	13059.57	25986.29	17616	23539.57	95318.43	43510.57	112005.86	0.197793593	0.703787779	28329.03	58398.086	Up	1.04363988151282	Isoform 4 of Sorbin and SH3 domain-containing protein 2 OS=Mus musculus GN=Sorbs2
DIABLO	Q9JIQ3	1	1	4511	6310	2902	11316	5686	9764	7613	NA	7950	NA	0.197855639	0.703651568	6145	8442.3333	Up	0.458228782551379	Diablo homolog, mitochondrial OS=Mus musculus GN=Diablo PE=1 SV=2
SCRG1	O88745	1	1	193167	189146	204347	84960	158397	134276	172051	574125	263235	289569	0.197872882	0.70361372	166003.4	286651.2	Up	0.788083528118435	Scrapie-responsive protein 1 OS=Mus musculus GN=Scrg1 PE=2 SV=1
SORBS2	Q3UTJ2-3	6	6	31020	28195.17	60134	14198.17	30106.5	20476.33	26784.33	111204.83	50550.5	130673.5	0.198010896	0.703310911	32730.768	67937.898	Up	1.05356912748447	Isoform 3 of Sorbin and SH3 domain-containing protein 2 OS=Mus musculus GN=Sorbs2

CHL1	P70232	2	2	26852	24608	10647	26058	22692.5	30943	30634	18508	36094.5	NA	0.198091	0.703135255	22171.5	29044.875	Up	0.389577242761442	Neural cell adhesion molecule L1-like protein OS=Mus musculus GN=Chl1 PE=1 SV=2
MANF	Q9CXI5	4	4	122321.5	112352.25	79781	136254	114977.75	108890	111107.75	36461.25	120230.5	30069.25	0.198287126	0.702705483	113137.3	81351.75	Down	-0.475829360953187	Mesencephalic astrocyte-derived neurotrophic factor OS=Mus musculus GN=Manf PE=1 SV=1
MDP1	Q9D967	2	2	23382.5	39667.5	38617	58521.5	35253.5	45140	38301.5	98719	42421	60880.5	0.198560477	0.702107192	39088.4	57092.4	Up	0.546558178148791	Magnesium-dependent phosphatase 1 OS=Mus musculus GN=Mdp1 PE=1 SV=1
EPB41L3	Q9WV92-7	32	32	23023.59	26185.25	24339.25	28579.72	25568.19	31498.31	29757.81	21620.06	28831.62	30274.56	0.198573303	0.70207914	25539.2	28396.472	Up	0.152998365099338	Isoform 7 of Band 4.1-like protein 3 OS=Mus musculus GN=Epb41l3
STIM2	P83093	2	2	21558.5	18764.5	39935	10988	18799.5	21875.5	22112	102819.5	28867.5	54763	0.198831811	0.701514132	22009.1	46087.5	Up	1.06627535982357	Stromal interaction molecule 2 OS=Mus musculus GN=Stim2 PE=1 SV=2
FAT3	Q8BNA6	1	1	3673	6441	2210	11296	8695	10052	10651	7638	8040	NA	0.199579301	0.699884502	6463	9095.25	Up	0.492909301984195	Protocadherin Fat 3 OS=Mus musculus GN=Fat3 PE=1 SV=2
GANAB	Q8BHN3-3	2	2	92141	109008.5	144981	110196.5	93259.5	126160.5	113756	167819	130137	109786.5	0.200021241	0.698923883	109917.3	129531.8	Up	0.236887850938159	Isoform 3 of Neutral alpha-glucosidase AB OS=Mus musculus GN=Ganab
RPLP1	P47955	9	9	43828.12	45846.62	73199.38	41095.75	40068.12	41153.12	47348.5	88714.88	62297.38	120369.5	0.200033754	0.698896714	48807.598	71976.676	Up	0.560423724605692	60S acidic ribosomal protein P1 OS=Mus musculus GN=Rplp1 PE=2 SV=1
MAP7D1	A2AJI0-2	7	7	19950.57	26615.71	28908.14	18148	22713	22838.71	24838	22711.86	39392.29	35209	0.200083169	0.698789442	23267.084	28997.972	Up	0.317661594506851	Isoform 2 of MAP7 domain-containing protein 1 OS=Mus musculus GN=Map7d1
AI837181	Q8VD62	1	1	3213	2653	NA	8766	7477	6346	8057	7222	11557	NA	0.200223976	0.698483919	5527.25	8295.5	Up	0.585767072514215	UPF0696 protein C11orf68 homolog OS=Mus musculus GN=Bles03 PE=2 SV=1
ACTR1A	P61164	5	5	87973	88903.4	109257	52781.4	81033	79729.4	84743.2	129813.8	100217.4	200459.4	0.200516637	0.697849587	83989.56	118992.64	Up	0.502590426427154	Alpha-centractin OS=Mus musculus GN=Actr1a PE=2 SV=1
ATP6V1A	P50516	40	40	89604.26	100627.74	99662.97	92983.15	86880.46	106313.41	98103.13	133123.74	83195.85	113459.26	0.200828291	0.697175107	93951.716	106839.08	Up	0.185448013575884	V-type proton ATPase catalytic subunit A OS=Mus musculus GN=Atp6v1a PE=1 SV=2
CCNY	Q8BGU5	1	1	54673	81999	42528	61309	66703	82732	69676	237074	106847	NA	0.201577662	0.695557597	61442.4	124082.25	Up	1.01399027695948	Cyclin-Y OS=Mus musculus GN=Ccny PE=1 SV=1
EXOC6B	A6H5Z3	1	1	2257	2468	NA	6511	5584	6861	5422	7654	4457	NA	0.201864092	0.694940928	4205	6098.5	Up	0.536348637494159	Exocyst complex component 6B OS=Mus musculus GN=Exoc6b PE=2 SV=1
MRPL49	Q9CQ40	4	4	4612.5	5270	4649	2487.5	4353.5	4825.5	3542.5	7083	5838.5	13590.5	0.20215902	0.694306877	4274.5	6976	Up	0.706644366246783	39S ribosomal protein L49, mitochondrial OS=Mus musculus GN=Mrpl49 PE=2 SV=1
GGACT	Q923B0	1	1	1330	1352	NA	2173	1703	2873	1790	NA	4364	NA	0.20218474	0.694251626	1639.5	3009	Up	0.876028204940276	Gamma-glutamylaminecyclotransferase OS=Mus musculus GN=Ggact PE=1 SV=1
MAST4	Q811L6	1	1	2029	NA	NA	1745	764	743	859	NA	NA	NA	0.202232625	0.694148781	1512.6667	801	Down	-0.917219960542471	Microtubule-associated serine/threonine-protein kinase 4 OS=Mus musculus GN=Mast4 PE=2 SV=3
MBP	P04370-13	43	43	1067687	850704.42	973799.77	440382.42	898442.12	657894.56	412761.63	761953.84	550680.35	895728.95	0.202374246	0.693844757	846203.14	655803.87	Down	-0.367739625759887	Isoform 12 of Myelin basic protein OS=Mus musculus GN=Mbp
SRGAP1	Q91Z69	1	1	12508	21374	23508	18488	17081	14082	17025	67031	27538	118819	0.202411666	0.69376446	18591.8	48899	Up	1.39513850766313	SLIT-ROBO Rho GTPase-activating protein 1 OS=Mus musculus GN=Srgap1 PE=1 SV=2
SLC39A10	Q6P5F6	1	1	13015	15788	10276	16250	16711	19277	15338	NA	26623	NA	0.202561529	0.693443033	14408	20412.667	Up	0.50259457888841	Zinc transporter ZIP10 OS=Mus musculus GN=Slc39a10 PE=1 SV=1
WIPF3	P0C7L0-2	5	5	48976	49496.2	59491	28065.6	56625.8	47187.2	51770	47519.2	86514.6	84094	0.202703371	0.693139028	48530.92	63417	Up	0.38596542151716	Isoform 2 of WAS/WASL-interacting protein family member 3 OS=Mus musculus GN=Wipf3
EXOC6	Q8R313	1	1	NA	1505	NA	1866	2530	3451	779	6944	5269	NA	0.203049498	0.692398079	1967	4110.75	Up	1.06340467779127	Exocyst complex component 6 OS=Mus musculus GN=Exoc6 PE=1 SV=2
EIF1	P48024	5	5	96573.25	80072	74530.25	77973.5	74950.5	69485.75	75990	14941.75	86189.25	61913.75	0.203163081	0.69215521	80819.9	61704.1	Down	-0.389344212355005	Eukaryotic translation initiation factor 1 OS=Mus musculus GN=Eif1 PE=2 SV=2
RPL29	P47915	11	11	99297.27	111701.91	178864.64	61947	89337.73	87892.27	105186.09	193176.73	153398.27	242612.55	0.203434052	0.69157635	108229.71	156453.18	Up	0.531634415005737	60S ribosomal protein L29 OS=Mus musculus GN=Rpl29 PE=2 SV=2
FDX1L	Q9CPW2	5	5	36766.8	32950.4	48879	25113.2	34048.6	34526.6	32661.2	56032.4	43170.8	79555.2	0.203602524	0.691216842	35551.6	49189.24	Up	0.468428275019469	Adrenodoxin-like protein, mitochondrial OS=Mus musculus GN=Fdx1l PE=2 SV=1
DENND1A	Q8K382	1	1	10647	12639	12673	14624	14880	20347	13912	NA	28729	NA	0.203667243	0.691078816	13092.6	20996	Up	0.681362878668173	DENN domain-containing protein 1A OS=Mus musculus GN=Dennd1a PE=1 SV=2
ARSB	P50429-2	1	1	39406	41486	80342	36453	35386	26535	39214	286120	69080	198540	0.203944217	0.690488604	46614.6	123897.8	Up	1.41029677802305	Isoform 2 of Arylsulfatase B OS=Mus musculus GN=Arsb
USP54	Q8BL06	2	2	4401	5206	NA	8376.5	5814.5	8259	8136	NA	6211	NA	0.203990847	0.690389319	5949.5	7535.3333	Up	0.340902903906313	Inactive ubiquitin carboxyl-terminal hydrolase 54 OS=Mus musculus GN=Usp54 PE=2 SV=2
ZNRF2	Q71FD5	2	2	5108	4399.5	7574	2742	4475	4606	5473	9209	8547.5	5039.5	0.204009288	0.69035006	4859.7	6575	Up	0.436123638572053	E3 ubiquitin-protein ligase ZNRF2 OS=Mus musculus GN=Znrf2 PE=1 SV=1
HOMER2	Q9QWW1	3	3	39305.67	42305	38535.67	31782.67	45271.33	35788.67	45408	38847	59137.33	52669.33	0.204119816	0.690114832	39440.068	46370.066	Up	0.23353174102903	Homer protein homolog 2 OS=Mus musculus GN=Homer2 PE=1 SV=1
WASF2	Q8BH43	2	2	10026.5	9862.5	5961	11313.5	11389	12233	10344	9988	13439.5	NA	0.204678889	0.688926948	9710.5	11501.125	Up	0.244157499495875	Wiskott-Aldrich syndrome protein family member 2 OS=Mus musculus GN=Wasf2 PE=1 SV=1
ARVCF	P98203-5	5	5	715.5	3308.5	NA	2193.5	4706	2989.5	4706.5	5320.5	3696	NA	0.20475406	0.688767478	2730.875	4178.125	Up	0.613492375891263	Isoform 5 of Armadillo repeat protein deleted in velo-cardio-facial syndrome homolog OS=Mus musculus GN=Arvcf
CADM1	Q8R5M8-3	7	7	39033.29	55127.57	44848	85439.43	60354.29	75333.57	85395.29	57451.86	58073.86	76454.71	0.205661703	0.686846573	56960.516	70541.858	Up	0.308517359765146	Isoform 3 of Cell adhesion molecule 1 OS=Mus musculus GN=Cadm1
GCAT	O88986	5	5	17555.75	20262.75	25825.5	19172.5	21238	23580	23846.75	37210.25	27954.25	17204	0.205816503	0.686519805	20810.9	25959.05	Up	0.318898229259103	2-amino-3-ketobutyrate coenzyme A ligase, mitochondrial OS=Mus musculus GN=Gcat PE=1 SV=2
AAK1	Q3UHJ0	28	28	47220.48	45627.63	90086.19	35726.59	53936.15	48024.04	54385.59	172876.15	57684.15	257277.48	0.206096791	0.68592877	54519.408	118049.48	Up	1.11454990923835	AP2-associated protein kinase 1 OS=Mus musculus GN=Aak1 PE=1 SV=2
SHISA4	Q8CA71	1	1	23414	8501	19348	14335	15563	21556	13860	NA	32898	56493	0.206188079	0.685736448	16232.2	31201.75	Up	0.942768401178504	Protein shisa-4 OS=Mus musculus GN=Shisa4 PE=2 SV=1
AAK1	Q3UHJ0-2	27	27	48727.81	47161.46	93051.88	36833	55811.62	49536.5	56197.58	178876.81	59445	266221.92	0.206399788	0.685290753	56317.154	122055.56	Up	1.11589170536676	Isoform 2 of AP2-associated protein kinase 1 OS=Mus musculus GN=Aak1
MKRN2	Q9ERV1	1	1	20306	24363	14610	20814	19978	29966	22278	NA	46142	NA	0.206486026	0.685109335	20014.2	32795.333	Up	0.712466588730708	Probable E3 ubiquitin-protein ligase makorin-2 OS=Mus musculus GN=Mkrn2 PE=2 SV=2
GPHN	Q8BUV3	13	13	34679.31	38226.46	48006.92	31796	36064	38731	38746.08	48490.85	38035.38	57938.92	0.206927609	0.68418156	37754.538	44388.446	Up	0.233534138921317	Gephyrin OS=Mus musculus GN=Gphn PE=1 SV=2
CTNNA2	Q61301-3	24	24	26567.37	32124.58	42882.16	54008.68	34926.26	42517.16	42847.63	57337.95	39757.89	47660.32	0.207083282	0.68385496	38101.81	46024.19	Up	0.272532797601269	Isoform 3 of Catenin alpha-2 OS=Mus musculus GN=Ctnna2
MINOS1	Q7TNS2	1	1	52684	53779	94274	25748	48857	38796	46318	195447	70778	242102	0.207133113	0.683750467	55068.4	118688.2	Up	1.10787991210579	Mitochondrial inner membrane organizing system protein 1 OS=Mus musculus GN=Minos1 PE=2 SV=1
UBE2M	P61082	1	1	123423	110331	113670	143254	114550	118334	129733	65766	116957	69425	0.207327645	0.683342786	121045.6	100043	Down	-0.274930412886676	NEDD8-conjugating enzyme Ubc12 OS=Mus musculus GN=Ube2m PE=2 SV=1
PLXNA3	P70208-2	1	1	5580	4652	3359	8100	4718	6527	6155	5532	8980	NA	0.207955864	0.682028829	5281.8	6798.5	Up	0.364186796081174	Isoform 2 of Plexin-A3 OS=Mus musculus GN=Plxna3
TAOK1	Q5F2E8	3	3	8736.33	9247	8847.67	6753	9193	9671	10024.33	10129.67	13435.67	7247.33	0.208140884	0.681642606	8555.4	10101.6	Up	0.239676607317563	Serine/threonine-protein kinase TAO1 OS=Mus musculus GN=Taok1 PE=1 SV=1
MSN	P26041	4	4	71345.67	83433	83125.33	70499.67	81153	99253.33	62114.67	119109.33	84788.33	194893	0.208156803	0.68160939	77911.334	112031.73	Up	0.524002298884789	Moesin OS=Mus musculus GN=Msn PE=1 SV=3
FKBP2	P45878	6	6	67182.5	92842.5	97825.5	55522	66673.17	66789.17	71986.5	133643.5	89401.33	153454.17	0.208581772	0.680723648	76009.134	103054.93	Up	0.439168876571224	Peptidyl-prolyl cis-trans isomerase FKBP2 OS=Mus musculus GN=Fkbp2 PE=1 SV=1
CTPS	P70698	1	1	4964	6256	2550	7689	7103	8159	8525	6234	6104	NA	0.209321099	0.679186995	5712.4	7255.5	Up	0.344978033610698	CTP synthase 1 OS=Mus musculus GN=Ctps1 PE=1 SV=2
WWC2	Q6NXJ0	1	1	1064	1384	1186	4213	2755	2804	2812	5532	2526	NA	0.209517691	0.6787793	2120.4	3418.5	Up	0.689026979601043	Protein WWC2 OS=Mus musculus GN=Wwc2 PE=1 SV=1
TPP2	Q64514	5	5	27081.8	32153.4	67592.4	57245.6	42803.2	51110.2	50773.8	85797.6	39914.2	137558	0.209806745	0.678180553	45375.28	73030.76	Up	0.686597698501383	Tripeptidyl-peptidase 2 OS=Mus musculus GN=Tpp2 PE=1 SV=3
NFU1	Q9QZ23	4	4	31236	31063.67	19833.33	35937.33	23626.33	34770.33	25223.33	42671.67	32272	34193	0.209874209	0.678040928	28339.332	33826.066	Up	0.255329648038901	NFU1 iron-sulfur cluster scaffold homolog, mitochondrial OS=Mus musculus GN=Nfu1 PE=1 SV=2
LAP3	Q9CPY7	14	14	53669.29	52579.71	57324.5	42648.14	58865.93	56233.43	57471.43	64019.14	61904.43	50604.86	0.209903946	0.677979397	53017.514	58046.658	Up	0.130743983635889	Cytosol aminopeptidase OS=Mus musculus GN=Lap3 PE=1 SV=3
MRPL53	Q9D1H8	5	5	34874	39113.2	25658.4	26349.2	39298.4	39816	41635	35013.8	57910.4	29740.4	0.210243567	0.677277284	33058.64	40823.12	Up	0.304359073048224	39S ribosomal protein L53, mitochondrial OS=Mus musculus GN=Mrpl53 PE=2 SV=1
SEPT11	Q8C1B7-3	18	18	97669.56	104190.22	101486.61	111046.72	99917.28	103767.83	108153.44	135977.5	95636.5	128889.33	0.210278608	0.677204907	102862.08	114484.92	Up	0.154446374315657	Isoform 3 of Septin-11 OS=Mus musculus GN=Sept11
IDH3G	P70404	28	28	72082.54	71121.43	73558.89	59658.46	70043.43	67353.46	66771.11	54513.32	73052.61	51294.75	0.210453986	0.676842843	69292.95	62597.05	Down	-0.146613908090926	Isocitrate dehydrogenase [NAD] subunit gamma 1, mitochondrial OS=Mus musculus GN=Idh3g PE=1 SV=1
ATP6V1C1	Q9Z1G3	3	3	5565	6697.67	NA	15289.33	9129.67	13114.67	12500.33	NA	12236.67	NA	0.210521378	0.676703796	9170.4175	12617.223	Up	0.46033513024763	V-type proton ATPase subunit C 1 OS=Mus musculus GN=Atp6v1c1 PE=1 SV=4
SLC25A22	Q9D6M3	11	11	17826.73	18656.45	22686	32216.27	22281.27	26885.73	30903.82	31231.18	18656	29529.55	0.210687823	0.676360565	22733.344	27441.256	Up	0.27153660044242	Mitochondrial glutamate carrier 1 OS=Mus musculus GN=Slc25a22 PE=1 SV=1
PACSIN1	Q61644	74	74	61323.23	58254.77	65003.58	58687.79	66776.37	60997.48	59658.36	51475.89	57993.49	62519.32	0.210895147	0.675933413	62009.148	58528.908	Down	-0.0833317052103921	Protein kinase C and casein kinase substrate in neurons protein 1 OS=Mus musculus GN=Pacsin1 PE=1 SV=1
SCO2	Q8VCL2	2	2	12868.5	23277.5	16941.5	28428.5	16009	25902	24569	25864.5	19513	NA	0.210931894	0.675857748	19505	23962.125	Up	0.296911855967106	Protein SCO2 homolog, mitochondrial OS=Mus musculus GN=Sco2 PE=2 SV=1
EPN1	Q80VP1	5	5	32071.6	33091.6	64103.6	28601.2	34010.8	35534.4	39157	68265.2	48273.2	119666.2	0.210932491	0.675856518	38375.76	62179.2	Up	0.696236732887238	Epsin-1 OS=Mus musculus GN=Epn1 PE=1 SV=3
MRPL14	Q9D1I6	4	4	34547	37416.67	48048.67	13135.67	26424	27067.67	30265	80863.33	49122	49875.33	0.211287838	0.675125501	31914.402	47438.666	Up	0.571855825745565	39S ribosomal protein L14, mitochondrial OS=Mus musculus GN=Mrpl14 PE=2 SV=1
SYN2	Q64332-2	26	26	76507.92	81337.54	100999.08	65953.38	81783.19	78736.58	82085.62	114594.31	87871.58	162428.5	0.211415994	0.674862161	81316.222	105143.32	Up	0.370742074379392	Isoform IIb of Synapsin-2 OS=Mus musculus GN=Syn2
MOCS3	A2BDX3	1	1	5792	8333	3138	8794	7238	8157	7279	NA	9948	NA	0.211557324	0.674571936	6659	8461.3333	Up	0.345579480379324	Adenylyltransferase and sulfurtransferase MOCS3 OS=Mus musculus GN=Mocs3 PE=2 SV=1
VSTM2B	Q9JME9	1	1	5547	6260	NA	15111	7682	13716	11889	NA	10914	NA	0.211712234	0.674254045	8650	12173	Up	0.492912721952742	V-set and transmembrane domain-containing protein 2B OS=Mus musculus GN=Vstm2b PE=2 SV=1
ASNA1	O54984	3	3	29582.33	28077.33	35250.67	59247.67	35557.67	46312.67	41545.67	51137	34603.33	80324.33	0.212181377	0.673292736	37543.134	50784.6	Up	0.435841990755533	ATPase Asna1 OS=Mus musculus GN=Asna1 PE=1 SV=2
SHC3	Q61120	2	2	20881.5	22789	16311.5	13700	23116	22366.5	23705	NA	37444.5	NA	0.212483298	0.672675201	19359.6	27838.667	Up	0.52404097038615	SHC-transforming protein 3 OS=Mus musculus GN=Shc3 PE=1 SV=2
CTNND2	O35927	36	36	24515.89	29635.14	21922.78	28332.5	33049.92	35436.56	37495.14	27633.53	36876.83	23436.56	0.212516338	0.672607676	27491.246	32175.724	Up	0.227000315173327	Catenin delta-2 OS=Mus musculus GN=Ctnnd2 PE=1 SV=1
NUDT11	P0C028	1	1	17580	28312	13804	44742	21708	36037	20919	50702	38464	NA	0.212531153	0.672577401	25229.2	36530.5	Up	0.534007040075214	Diphosphoinositol polyphosphate phosphohydrolase 3-beta OS=Mus musculus GN=Nudt11 PE=1 SV=1
TMEM263	Q9DAM7	1	1	82072	61213	155873	12401	60561	31774	60573	296206	145220	219306	0.212803735	0.672020754	74424	150615.8	Up	1.01703328361751	UPF0444 transmembrane protein C12orf23 homolog OS=Mus musculus PE=2 SV=1
RAB3GAP1	Q80UJ7	2	2	30836	43880.5	57181	52062	47671	55048	47632.5	59927.5	51379.5	NA	0.213505293	0.670591355	46326.1	53496.875	Up	0.207629386441864	Rab3 GTPase-activating protein catalytic subunit OS=Mus musculus GN=Rab3gap1 PE=2 SV=4
2310022B05RIK	Q8C3W1	10	10	22127.89	21176.33	29680.44	15453	22317.67	22623.33	20429.22	51246.78	34372.56	25118.22	0.213528446	0.67054426	22151.066	30758.022	Up	0.473586599990816	Uncharacterized protein C1orf198 homolog OS=Mus musculus PE=2 SV=1
FARSB	Q9WUA2	1	1	NA	20703	NA	34568	20326	34739	50320	NA	NA	NA	0.215430496	0.666692819	25199	42529.5	Up	0.755097411167507	Phenylalanine--tRNA ligase beta subunit OS=Mus musculus GN=Farsb PE=2 SV=2
TRIM28	Q62318-2	3	3	39123.33	27971	30987	24085	33132	27739	29788	29859.67	27349.67	11887.67	0.215530635	0.666490992	31059.666	25324.802	Down	-0.294491326321763	Isoform 2 of Transcription intermediary factor 1-beta OS=Mus musculus GN=Trim28
CNNM2	Q3TWN3	1	1	1939	1235	NA	5753	2783	3422	4579	NA	6993	NA	0.215711535	0.666126632	2927.5	4998	Up	0.771681730698127	Metal transporter CNNM2 OS=Mus musculus GN=Cnnm2 PE=1 SV=3
ECHS1	Q8BH95	31	31	69905.74	77563.84	92796.19	63762.65	71091.16	71658.42	72331.55	113739.48	78511.19	124033.9	0.215937536	0.665671858	75023.916	92054.908	Up	0.295144073493866	Enoyl-CoA hydratase, mitochondrial OS=Mus musculus GN=Echs1 PE=1 SV=1
DNAJC7	Q9QYI3	1	1	4747	2896	5293	6017	5878	6649	6807	NA	13688	NA	0.215990439	0.665565474	4966.2	9048	Up	0.865456566081598	DnaJ homolog subfamily C member 7 OS=Mus musculus GN=Dnajc7 PE=1 SV=2
SHANK2	Q80Z38-3	36	36	81538.11	83763.08	78564.92	61430.31	84764.36	54182.39	72458.28	72434.33	81730.89	67569.17	0.216424414	0.664693749	78012.156	69675.012	Down	-0.163057599128059	Isoform 3 of SH3 and multiple ankyrin repeat domains protein 2 OS=Mus musculus GN=Shank2
GALNT1	O08912	1	1	9570	5318	14057	4860	7440	6613	6791	23543	10374	23163	0.216671353	0.664198505	8249	14096.8	Up	0.773076563567508	Polypeptide N-acetylgalactosaminyltransferase 1 OS=Mus musculus GN=Galnt1 PE=1 SV=1
PSMD9	Q9CR00	5	5	18599.2	16084	20909.4	17217.8	15584.2	16549.8	16007.4	23097.8	23783	37027.6	0.21667404	0.66419312	17678.92	23293.12	Up	0.397873750860054	26S proteasome non-ATPase regulatory subunit 9 OS=Mus musculus GN=Psmd9 PE=1 SV=1
PDHA2	P35487	1	1	145703	144808	181624	132070	148017	99659	166766	65431	132825	NA	0.217332249	0.662875825	150444.4	116170.25	Down	-0.372989749398486	Pyruvate dehydrogenase E1 component subunit alpha, testis-specific form, mitochondrial OS=Mus musculus GN=Pdha2 PE=2 SV=1
WARS2	Q9CYK1	3	3	11698.5	14290	2680	16516.5	13050	15021	13015.5	NA	20028	NA	0.218041686	0.661460469	11647	16021.5	Up	0.460050827643279	Tryptophan--tRNA ligase, mitochondrial OS=Mus musculus GN=Wars2 PE=2 SV=2
HSPB8	Q9JK92	1	1	2666	3635	1835	1110	2768	2912	2620	4112	2913	NA	0.218207867	0.661129596	2402.8	3139.25	Up	0.38570335621519	Heat shock protein beta-8 OS=Mus musculus GN=Hspb8 PE=1 SV=1
PAFAH1B2	Q61206	3	3	26517	26537.33	43160.33	26102	25754	37471.67	29944.67	35234.67	29296	55183.67	0.218223373	0.661098737	29614.132	37426.136	Up	0.33776030636013	Platelet-activating factor acetylhydrolase IB subunit beta OS=Mus musculus GN=Pafah1b2 PE=1 SV=2
TBC1D5	Q80XQ2	2	2	30325	26811.5	81151.5	15123.5	34834.5	28659	34980	111339.5	63802	247648.5	0.218308676	0.660929004	37649.2	97285.8	Up	1.36961003246669	TBC1 domain family member 5 OS=Mus musculus GN=Tbc1d5 PE=1 SV=2
CTNNA1	P26231	7	7	10507.6	15442	24460	25945.6	20138.8	20996.6	20038.2	32558.8	17821.4	43876.4	0.218692901	0.660165315	19298.8	27058.28	Up	0.487558991704521	Catenin alpha-1 OS=Mus musculus GN=Ctnna1 PE=1 SV=1
ZC3H4	Q6ZPZ3	3	3	20905	8717	NA	11205	19106	19585	14680	55990	27359	NA	0.218807358	0.659938079	14983.25	29403.5	Up	0.972637303140529	Zinc finger CCCH domain-containing protein 4 OS=Mus musculus GN=Zc3h4 PE=1 SV=2
CPE	Q00493	3	3	34040	34033.33	41435.67	26894.33	30743.67	30472.67	27239.67	50100.67	49913	85138	0.21890233	0.659749616	33429.4	48572.802	Up	0.53903125163675	Carboxypeptidase E OS=Mus musculus GN=Cpe PE=1 SV=2
PRKAR1B	P12849	8	8	74996.5	78568.88	52857.88	84520.88	71576.25	73462.5	75370.5	37318.25	68079.75	43508	0.219035082	0.65948632	72504.078	59547.8	Down	-0.284013933406559	cAMP-dependent protein kinase type I-beta regulatory subunit OS=Mus musculus GN=Prkar1b PE=2 SV=2
PDLIM4	P70271	1	1	6095	15362	10497	12108	12336	17668	11129	NA	23476	NA	0.219323786	0.658914267	11279.6	17424.333	Up	0.627387551239857	PDZ and LIM domain protein 4 OS=Mus musculus GN=Pdlim4 PE=1 SV=3
CELF2	Q9Z0H4-2	16	16	71565.25	67744.94	81209.69	49300.5	70248.5	58352.94	69729.12	95412.12	79728.56	99838.69	0.219486654	0.658591883	68013.776	80612.286	Up	0.245172744737085	Isoform 2 of CUGBP Elav-like family member 2 OS=Mus musculus GN=Celf2
SEPHS2	P97364	1	1	3061	4213	3464	2887	3449	6769	7478	4055	2530	NA	0.219544008	0.658478412	3414.8	5208	Up	0.608928364119273	Selenide, water dikinase 2 OS=Mus musculus GN=Sephs2 PE=1 SV=3
ALDOC	P05063	88	88	106604.3	100436.34	114786.31	68717.47	97566.22	87755.64	94826.94	133678.9	115895.34	164347.33	0.219578711	0.658409769	97622.128	119300.83	Up	0.289323974650178	Fructose-bisphosphate aldolase C OS=Mus musculus GN=Aldoc PE=1 SV=4
P4HTM	Q8BG58	1	1	528	1466	NA	2321	537	2468	1960	NA	1425	NA	0.219874714	0.657824713	1213	1951	Up	0.685634227488516	Transmembrane prolyl 4-hydroxylase OS=Mus musculus GN=P4htm PE=2 SV=1
NT5C3B	Q3UFY7	1	1	2339	2893	1483	3747	2372	5058	2365	NA	4512	NA	0.22053069	0.656530963	2566.8	3978.3333	Up	0.632193272249145	7-methylguanosine phosphate-specific 5'-nucleotidase OS=Mus musculus GN=Nt5c3b PE=2 SV=3
SYN2	Q64332	31	31	82183.97	87445.13	105019.74	71390.35	90484.06	83383.58	87128.71	118659.35	93429.71	158409.26	0.220650983	0.656294133	87304.65	108202.12	Up	0.309598391274995	Synapsin-2 OS=Mus musculus GN=Syn2 PE=1 SV=2
RASA3	Q60790	1	1	32839	60962	30634	84251	72146	78439	78708	67782	63071	NA	0.220919129	0.655766679	56166.4	72000	Up	0.358289570588569	Ras GTPase-activating protein 3 OS=Mus musculus GN=Rasa3 PE=1 SV=2
SEPT11	Q8C1B7	19	19	96703.16	102175.95	101666.26	107385.63	98284	100734.79	105950.21	134760.42	94144.21	129598.47	0.221197955	0.655218892	101243	113037.62	Up	0.158980832580793	Septin-11 OS=Mus musculus GN=Sept11 PE=1 SV=4
YBX1	P62960	7	7	8469.83	10231.67	3355	11811	8980.67	9794.67	10171.67	NA	14853.5	NA	0.221295615	0.655027192	8569.634	11606.613	Up	0.437641578194331	Nuclease-sensitive element-binding protein 1 OS=Mus musculus GN=Ybx1 PE=1 SV=3
HADHA	Q8BMS1	28	28	41082.57	47283.39	45815.93	42862.21	52485.54	46362.79	49390.82	52002.68	44310.46	60565.18	0.221446909	0.654730378	45905.928	50526.386	Up	0.13835652587698	Trifunctional enzyme subunit alpha, mitochondrial OS=Mus musculus GN=Hadha PE=1 SV=1
SLC12A6	Q924N4	3	3	20197	31658.67	20962	43096	38382.33	48021	48499.33	26331	38109	NA	0.221581131	0.654467226	30859.2	40240.083	Up	0.382932601270012	Solute carrier family 12 member 6 OS=Mus musculus GN=Slc12a6 PE=1 SV=2
SMARCA4	Q3TKT4	3	3	1538.33	2874.33	1038.33	2891.67	3073	3169.33	3770.67	NA	2341.67	NA	0.221877002	0.653887712	2283.132	3093.89	Up	0.43840763235145	Transcription activator BRG1 OS=Mus musculus GN=Smarca4 PE=1 SV=1
PFKP	Q9WUA3	17	17	35500.35	44173.12	43015.18	69626.94	49036.59	60947.65	61925.35	64882.53	41899.76	58839.88	0.222054889	0.65353966	48270.436	57699.034	Up	0.257407307500009	6-phosphofructokinase type C OS=Mus musculus GN=Pfkp PE=1 SV=1
MIF	P34884	15	15	22668.86	21949.93	31098.5	24339.5	24890.14	23745.79	21496.29	36171.14	30163	43368.14	0.222132703	0.653387499	24989.386	30988.872	Up	0.310434787324612	Macrophage migration inhibitory factor OS=Mus musculus GN=Mif PE=1 SV=2

MYL6	Q60605	12	12	50648	57910.25	46073.33	73539.5	56231.33	55040.83	57847.25	42218.75	53450.17	22875.42	0.222257651	0.65314328	56880.482	46286.484	Down	-0.297342713050525	Myosin light polypeptide 6 OS=Mus musculus GN=Myl6 PE=1 SV=3
TECPR1	Q80VP0	2	2	20541.5	40961	NA	31400.5	45613	51807	53993.5	NA	33934.5	NA	0.222284302	0.653091207	34629	46578.333	Up	0.427678291268582	Tectonin beta-propeller repeat-containing protein 1 OS=Mus musculus GN=Tecpr1 PE=2 SV=1
SORBS2	Q3UTJ2-5	8	8	32435.38	33174.75	59558.12	23732.88	33414.75	25494.62	30379.25	102709.62	48198.75	98005.12	0.222431418	0.652803869	36463.176	60957.472	Up	0.741362845434414	Isoform 5 of Sorbin and SH3 domain-containing protein 2 OS=Mus musculus GN=Sorbs2
SCG5	P12961	3	3	21514.33	20589.33	30852.67	13046	18887	15392	21348	36396.33	27962.67	52897.33	0.2224392	0.652788675	20977.866	30799.266	Up	0.554028044003141	Neuroendocrine protein 7B2 OS=Mus musculus GN=Scg5 PE=1 SV=1
NDUFV2	Q9D6J6-2	8	8	47325.5	50657.88	53864.5	61628.25	51080.88	54666.62	48297.88	79750.62	59831.88	61747.12	0.222492979	0.652683688	52911.402	60858.824	Up	0.201887810812607	Isoform 2 of NADH dehydrogenase [ubiquinone] flavoprotein 2, mitochondrial OS=Mus musculus GN=Ndufv2
SQSTM1	Q64337	1	1	15174	22016	18537	28219	22447	18408	19919	NA	14137	NA	0.222587987	0.652498278	21278.6	17488	Down	-0.283037927446908	Sequestosome-1 OS=Mus musculus GN=Sqstm1 PE=1 SV=1
OSBPL2	Q8BX94	1	1	22646	35865	104365	8459	30308	20486	27601	176147	61519	221119	0.222639832	0.652397135	40328.6	101374.4	Up	1.32981814682474	Oxysterol-binding protein-related protein 2 OS=Mus musculus GN=Osbpl2 PE=2 SV=1
MYL6	Q60605-2	15	15	41189.33	47280.6	37797.2	59941	46028.67	45128.53	47456	33775	43726.27	18300.33	0.222851076	0.651985264	46447.36	37677.226	Down	-0.30190384836924	Isoform Smooth muscle of Myosin light polypeptide 6 OS=Mus musculus GN=Myl6
HP1BP3	Q3TEA8-3	3	3	37542.67	27838	23271.33	20145	37284.67	30456	31484.67	49725.33	48212.67	26362	0.222929124	0.651833191	29216.334	37248.134	Up	0.350392990094363	Isoform 3 of Heterochromatin protein 1-binding protein 3 OS=Mus musculus GN=Hp1bp3
MOB4	Q6PEB6	3	3	2042	3655.67	2636	5913.67	3671.33	5581.33	3985.67	5318	3769.33	NA	0.222951675	0.651789261	3583.734	4663.5825	Up	0.379975080881021	MOB-like protein phocein OS=Mus musculus GN=Mob4 PE=1 SV=1
ATP5D	Q9D3D9	22	22	68413.09	76420.18	67670.64	57656.5	68622.23	65136.36	66802.14	92312.73	70131.18	115211.45	0.222959511	0.651773997	67756.528	81918.772	Up	0.27383413927985	ATP synthase subunit delta, mitochondrial OS=Mus musculus GN=Atp5d PE=1 SV=1
HYOU1	Q9JKR6	23	23	54730.1	62797.29	71046.52	59201.05	54425.57	60025.43	62614	77081.19	61312.62	70788.1	0.22334641	0.651021024	60440.106	66364.268	Up	0.134900480765177	Hypoxia up-regulated protein 1 OS=Mus musculus GN=Hyou1 PE=1 SV=1
NUMB	Q9QZS3-3	5	5	113949	115770.6	114671.8	61699	87144	90491	103131	111201.6	141814	149293	0.223589852	0.650547912	98646.88	119186.12	Up	0.272870907071947	Isoform 3 of Protein numb homolog OS=Mus musculus GN=Numb
MRPS36	Q9CQX8	10	10	214488.5	184316.8	304529.2	74878.6	126726	142027.5	160008	389644.9	274226.7	363295.3	0.224043595	0.649667468	180987.82	265840.48	Up	0.55466819203909	28S ribosomal protein S36, mitochondrial OS=Mus musculus GN=Mrps36 PE=1 SV=1
CENPV	Q9CXS4	13	13	44976.5	23473	8831.8	16090.7	31425.8	26485.9	20787	5837.9	14931	7306.3	0.224214981	0.649335373	24959.56	15069.62	Down	-0.727949463882802	Centromere protein V OS=Mus musculus GN=Cenpv PE=2 SV=2
FHIT	O89106	1	1	13713	17357	10768	17612	15825	16963	14153	18394	20901	NA	0.224484996	0.64881268	15055	17602.75	Up	0.225558124290932	Bis(5'-adenosyl)-triphosphatase OS=Mus musculus GN=Fhit PE=2 SV=3
IDH3A	Q9D6R2-2	18	18	93251.17	108807.72	97015.89	154660.67	97895.72	118605.5	113104.94	44088	98033.61	45895.56	0.224547768	0.648691258	110326.23	83945.522	Down	-0.394250610932072	Isoform 2 of Isocitrate dehydrogenase [NAD] subunit alpha, mitochondrial OS=Mus musculus GN=Idh3a
GLO1	Q9CPU0	3	3	19120.33	21830	23698	19503.67	32784	20741.33	22582	106539	31262.67	50708.33	0.224599872	0.648590496	23387.2	46366.666	Up	0.987368845931651	Lactoylglutathione lyase OS=Mus musculus GN=Glo1 PE=1 SV=3
COTL1	Q9CQI6	6	6	60007.83	66977.33	82785.17	84438.67	55637.17	71474.17	67010.83	90117.5	76578.5	121769.33	0.224654149	0.648485556	69969.234	85390.066	Up	0.287347543610603	Coactosin-like protein OS=Mus musculus GN=Cotl1 PE=1 SV=3
DYNC1LI2	Q6PDL0	6	6	19107.8	25471	26666.8	28331	24446	26986.8	31525.8	22578.6	28995.2	54399.2	0.225313991	0.647211841	24804.52	32897.12	Up	0.407358248343683	Cytoplasmic dynein 1 light intermediate chain 2 OS=Mus musculus GN=Dync1li2 PE=1 SV=2
TIMM10B	Q9WV96	5	5	78872.4	70502.4	105877.8	59271.6	75258.8	71624	60059	139074.6	100672.6	155028.4	0.226471969	0.644985545	77956.6	105291.72	Up	0.433648913852945	Mitochondrial import inner membrane translocase subunit Tim10 B OS=Mus musculus GN=Timm10b PE=2 SV=1
NOL3	Q9D1X0	6	6	7759.4	10182	3700.8	10451.6	8868.6	9220.2	7292.6	3575.8	7272	1019.2	0.227511215	0.642997191	8192.48	5675.96	Down	-0.529435823395451	Nucleolar protein 3 OS=Mus musculus GN=Nol3 PE=1 SV=1
JPH3	Q9ET77	2	2	10765.5	17156.5	8380	26268	21579	26225.5	24304.5	15488.5	22866.5	NA	0.227603205	0.642821627	16829.8	22221.25	Up	0.400921941916368	Junctophilin-3 OS=Mus musculus GN=Jph3 PE=1 SV=1
PRUNE1	Q8BIW1	3	3	14844	17759.33	14408.33	11913	14539.33	14543	16858	NA	22485.67	15873	0.227853954	0.642343431	14692.798	17439.918	Up	0.247284055993092	Protein prune homolog OS=Mus musculus GN=Prune PE=2 SV=1
HNRNPK	P61979-3	25	25	86121.36	76004.76	55946.48	64096.84	77293.2	68905.16	73945.6	49036.64	64610.48	58042.44	0.227856848	0.642337915	71892.528	62908.064	Down	-0.1925968711554	Isoform 3 of Heterogeneous nuclear ribonucleoprotein K OS=Mus musculus GN=Hnrnpk
HTRA1	Q9R118	1	1	34586	61212	38319	40811	32921	42451	48890	101869	55660	NA	0.227951224	0.642158072	41569.8	62217.5	Up	0.581784619305498	Serine protease HTRA1 OS=Mus musculus GN=Htra1 PE=1 SV=2
MYO18A	Q9JMH9-5	36	36	7699.03	12339.86	10538.46	22465.69	14304	19509.17	21077.6	15571.69	17079.09	13213.89	0.228095204	0.641883846	13469.408	17290.288	Up	0.360275456134588	Isoform 5 of Unconventional myosin-XVIIIa OS=Mus musculus GN=Myo18a
IPCEF1	Q5DU31	3	3	7080.67	7237.67	11822.33	4667.33	8175.67	7748	9138.67	7662.33	12026.67	12665.33	0.228127134	0.641823056	7796.734	9848.2	Up	0.336990145612896	Interactor protein for cytohesin exchange factors 1 OS=Mus musculus GN=Ipcef1 PE=2 SV=2
SPECC1L	Q2KN98	1	1	3040	4789	NA	14823	14799	13734	11316	NA	23366	NA	0.228615845	0.640893673	9362.75	16138.667	Up	0.785517150544359	Cytospin-A OS=Mus musculus GN=Specc1l PE=1 SV=1
FGF1	P61148	1	1	74692	70504	104790	29137	66284	66995	57801	140591	78776	185342	0.228658371	0.640812895	69081.4	105901	Up	0.616346986591398	Fibroblast growth factor 1 OS=Mus musculus GN=Fgf1 PE=2 SV=1
SPAST	Q9QYY8	1	1	43641	50115	44576	40682	46814	54579	45674	116582	42846	62123	0.228659705	0.640810361	45165.6	64360.8	Up	0.510957884449593	Spastin OS=Mus musculus GN=Spast PE=1 SV=3
EN2	P09066	1	1	295415	338490	388909	171348	238355	221383	288328	441196	422825	839459	0.22893829	0.640281566	286503.4	442638.2	Up	0.627575702278732	Homeobox protein engrailed-2 OS=Mus musculus GN=En2 PE=2 SV=2
RUNDC3A	O08576-3	1	1	40530	50975	35096	18912	63198	36321	52465	40892	70915	87339	0.229085312	0.640002756	41742.2	57586.4	Up	0.464221496157333	Isoform 3 of RUN domain-containing protein 3A OS=Mus musculus GN=Rundc3a
RPS2	P25444	10	10	84959.3	85824.9	102677.3	88415.2	86849.5	80162	86304.9	110700.5	106796.3	131092.2	0.229161597	0.63985816	89745.24	103011.18	Up	0.198893597413073	40S ribosomal protein S2 OS=Mus musculus GN=Rps2 PE=2 SV=3
TUBA4A	P68368	82	82	181713.84	216139.47	227302.21	251156.57	192347.86	232925.57	232153.28	235751.25	224174.3	231854.3	0.22926995	0.639652865	213731.99	231371.74	Up	0.114409805565396	Tubulin alpha-4A chain OS=Mus musculus GN=Tuba4a PE=1 SV=1
HIST1H1D	P43277	20	20	294026.6	159469.7	175064.1	84878	198409.85	130497.4	116478.35	151668.35	121034.4	150908.75	0.22931458	0.639568332	182369.65	134117.45	Down	-0.443368697554809	Histone H1.3 OS=Mus musculus GN=Hist1h1d PE=1 SV=2
ATP5A1	Q03265	175	175	109360.01	103381.69	116671.13	76516.81	88703.46	102077.24	94382.75	133537.67	101047.06	168420.56	0.229340946	0.6395184	98926.62	119893.06	Up	0.277317411999116	ATP synthase subunit alpha, mitochondrial OS=Mus musculus GN=Atp5a1 PE=1 SV=1
SLAIN1	Q68FF7	1	1	16909	20219	7644	10098	17148	16755	17003	10821	28898	24494	0.22957676	0.639072078	14403.6	19594.2	Up	0.443997230849109	SLAIN motif-containing protein 1 OS=Mus musculus GN=Slain1 PE=2 SV=1
APMAP	Q9D7N9	2	2	23327	37536	15327	36174	31050	37543	39845.5	29259.5	45966	26376.5	0.231466235	0.635512353	28682.8	35798.1	Up	0.319697151790707	Adipocyte plasma membrane-associated protein OS=Mus musculus GN=Apmap PE=1 SV=1
GSK3A	Q2NL51	4	4	9659	13302.75	4798.25	30163	19840.75	27340.5	28812	21825.25	21089	12947.25	0.231797014	0.634892162	15552.75	22402.8	Up	0.52650936110049	Glycogen synthase kinase-3 alpha OS=Mus musculus GN=Gsk3a PE=1 SV=2
SRGAP2	Q91Z67	8	8	22916.75	33932.88	42116.75	25274.38	32459.75	29750.75	36021.25	46940.88	34758.12	95956.12	0.231975721	0.634557466	31340.102	48685.424	Up	0.635480031146732	SLIT-ROBO Rho GTPase-activating protein 2 OS=Mus musculus GN=Srgap2 PE=1 SV=2
FAHD2A	Q3TC72	4	4	30661.75	28767.5	35679.5	17106.25	28862.25	25581.75	31568	36551.25	33956.5	69368.5	0.232576682	0.63343383	28215.45	39405.2	Up	0.481900664680898	Fumarylacetoacetate hydrolase domain-containing protein 2A OS=Mus musculus GN=Fahd2 PE=1 SV=1
NDUFA6	Q9CQZ5	12	12	69783.33	69648.08	68980.17	67384.58	67201.42	69455.17	68375.58	55475	69892.92	45177.25	0.232605662	0.633379718	68599.516	61675.184	Down	-0.153508282953432	NADH dehydrogenase [ubiquinone] 1 alpha subcomplex subunit 6 OS=Mus musculus GN=Ndufa6 PE=1 SV=1
LYRM4	Q8K215	3	3	128373.67	109951	122837.33	77841.67	104777.33	110431	93079.33	168538.67	133738.67	140160.67	0.232732198	0.63314353	108756.2	129189.67	Up	0.248393045663867	LYR motif-containing protein 4 OS=Mus musculus GN=Lyrm4 PE=1 SV=1
MAP7	O88735-2	3	3	91704.33	70816.33	133067.33	53705.67	85192	85778.67	72065.67	139655	121488.67	270264.33	0.232805968	0.633005891	86897.132	137850.47	Up	0.665723697831905	Isoform 2 of Ensconsin OS=Mus musculus GN=Map7
MBP	P04370-5	56	56	1046832.9	843881.27	990346.98	398252.73	874101.41	637518.55	419284.95	744254.02	550458.38	890697.34	0.232814866	0.632989292	830683.06	648442.65	Down	-0.357319161134409	Isoform 5 of Myelin basic protein OS=Mus musculus GN=Mbp
PCYOX1	Q9CQF9	1	1	46156	47395	74824	32380	35630	49210	43360	195822	92809	56810	0.233329605	0.632030155	47277	87602.2	Up	0.889828610391147	Prenylcysteine oxidase OS=Mus musculus GN=Pcyox1 PE=1 SV=1
ALDH1A1	P24549	2	2	28927	43454	76006	43932.5	45529	56776.5	49848	63546.5	47783.5	88734.5	0.23355188	0.631616633	47569.7	61337.8	Up	0.366733495535793	Retinal dehydrogenase 1 OS=Mus musculus GN=Aldh1a1 PE=1 SV=5
ARPP21	Q9DCB4-2	6	6	52657.67	47195.33	66316.83	20837	46283.5	36837	43346.5	71546.67	70350.5	119258.83	0.233661141	0.631413507	46658.066	68267.9	Up	0.549080865447623	Isoform 2 of cAMP-regulated phosphoprotein 21 OS=Mus musculus GN=Arpp21
LYSMD1	Q9D0E3	6	6	48568.83	47632.17	64278.83	30286.67	37042.17	39638.5	44102.33	85472.67	67049.83	57089.5	0.234022815	0.6307418	45561.734	58670.566	Up	0.364814255335894	LysM and putative peptidoglycan-binding domain-containing protein 1 OS=Mus musculus GN=Lysmd1 PE=2 SV=1
FAM221A	Q8C790	1	1	7938	7281	11125	5888	6905	8311	7356	11433	11527	NA	0.234381095	0.63007742	7827.4	9656.75	Up	0.303004556488814	Protein FAM221A OS=Mus musculus GN=Fam221a PE=2 SV=1
ATXN2L	Q7TQH0-3	10	10	24113.4	31294.7	27498.7	27934	25870.9	28609.1	30080.8	32856	38190.4	24217.7	0.234412527	0.630019184	27342.34	30790.8	Up	0.171362635350706	Isoform 3 of Ataxin-2-like protein OS=Mus musculus GN=Atxn2l
PTPRD	Q64487-11	5	5	47171.2	49718.6	61087.2	28758.6	46351	47474.4	56753.4	73954.8	62099.4	41941.8	0.23459152	0.629687692	46617.32	56444.76	Up	0.275973588647577	Isoform K of Receptor-type tyrosine-protein phosphatase delta OS=Mus musculus GN=Ptprd
ARHGAP35	Q91YM2	1	1	4162	5959	3734	14399	5351	10599	8910	NA	8998	NA	0.234964826	0.628997146	6721	9502.3333	Up	0.49960591229097	Rho GTPase-activating protein 35 OS=Mus musculus GN=Arhgap35 PE=1 SV=3
PPIB	P24369	5	5	196214.4	228449.4	123491.6	339738.2	174193.2	175861	209590.4	144312.4	169321.6	93093	0.23499938	0.628933283	212417.36	158435.68	Down	-0.423004406987561	Peptidyl-prolyl cis-trans isomerase B OS=Mus musculus GN=Ppib PE=1 SV=2
SYN3	Q8JZP2	2	2	48424	45161.5	71749	22967.5	44825	37528	33830.5	110551.5	66368	100873	0.236080885	0.626939176	46625.4	69830.2	Up	0.582735002413797	Synapsin-3 OS=Mus musculus GN=Syn3 PE=1 SV=2
GSTM1	P10649	3	3	34333	31749.67	31572.33	33672.67	29514.67	33047.67	31858.33	57904.67	34581.67	37235.67	0.236351319	0.62644197	32168.468	38925.602	Up	0.275072121012749	Glutathione S-transferase Mu 1 OS=Mus musculus GN=Gstm1 PE=1 SV=2
PPA2	Q91VM9	10	10	47904.67	55520	52849.11	55732.33	47859.56	50019.22	54321.11	69575.67	56332.78	120835.33	0.236353326	0.626438282	51973.134	70216.822	Up	0.434050644897769	Inorganic pyrophosphatase 2, mitochondrial OS=Mus musculus GN=Ppa2 PE=1 SV=1
MRPS34	Q9JIK9	2	2	45376.5	48481	55864.5	20958	37498.5	32543	39047.5	69002	55996	99631	0.236390158	0.626370609	41635.7	59243.9	Up	0.508845536267741	28S ribosomal protein S34, mitochondrial OS=Mus musculus GN=Mrps34 PE=2 SV=1
GM7426	P02301	16	16	79373.2	39440.27	45815.93	28599.67	54754.53	30043.67	31930.87	45460.87	29343.07	48460.73	0.237035744	0.62518616	49596.72	37047.842	Down	-0.420855204005342	Histone H3.3C OS=Mus musculus GN=H3f3c PE=3 SV=3
SNX16	Q8C080	1	1	5432	7255	10584	9337	15653	8551	6281	NA	6590	NA	0.237328466	0.624650169	9652.2	7140.6667	Down	-0.434799036078295	Sorting nexin-16 OS=Mus musculus GN=Snx16 PE=1 SV=2
LDHB	P16125	13	13	61820.62	68435.77	73388.77	84033.69	67553.15	82467.15	78259.31	100530.31	55051.46	98901.15	0.237344697	0.624620467	71046.4	83041.876	Up	0.225077487644665	L-lactate dehydrogenase B chain OS=Mus musculus GN=Ldhb PE=1 SV=2
VHL	P40338	2	2	9488	10745	10709	6979	7735	10430	10649	6659	13394	16113	0.237660466	0.624043055	9131.2	11449	Up	0.326345220013206	Von Hippel-Lindau disease tumor suppressor OS=Mus musculus GN=Vhl PE=1 SV=1
CYCS	P62897	18	18	68898.56	65613.39	58611.5	95785.22	60066.83	84433.33	80914.67	76387.22	75069.94	79463.06	0.238514555	0.622485114	69795.1	79253.644	Up	0.183351515531082	Cytochrome c, somatic OS=Mus musculus GN=Cycs PE=1 SV=2
FRMD4A	Q8BIE6	5	5	29972.4	33536.6	41286.8	56593	41414.4	49022.8	57748.2	34664.6	57935	NA	0.238533571	0.622450491	40560.64	49842.65	Up	0.297300358045985	FERM domain-containing protein 4A OS=Mus musculus GN=Frmd4a PE=1 SV=2
REXO2	Q9D8S4	5	5	78273	74494	94056.2	42626.6	75934.4	65014.4	72887.8	122112.4	98243.4	90588	0.238744528	0.622066574	73076.84	89769.2	Up	0.296806288109338	Oligoribonuclease, mitochondrial OS=Mus musculus GN=Rexo2 PE=1 SV=2
TMEM55B	Q3TWL2	1	1	10650	17069	24738	12023	15681	20813	18362	68280	25688	NA	0.239594061	0.620523951	16032.2	33285.75	Up	1.05393226443841	Type 1 phosphatidylinositol 4,5-bisphosphate 4-phosphatase OS=Mus musculus GN=Tmem55b PE=1 SV=1
CABLES1	Q9ESJ1	3	3	3445	4873.67	4616.67	2869.33	5398.33	4792.67	4598	NA	6636.33	NA	0.239759263	0.620224606	4240.6	5342.3333	Up	0.333201590015039	CDK5 and ABL1 enzyme substrate 1 OS=Mus musculus GN=Cables1 PE=1 SV=2
NAA10	Q9QY36	4	4	40515.75	47416.25	29460.75	38240.25	43939.5	37427.75	39463.5	6952.75	45566.75	18022	0.239981647	0.619821971	39914.5	29486.55	Down	-0.436855905526339	N-alpha-acetyltransferase 10 OS=Mus musculus GN=Naa10 PE=1 SV=1
MBP	P04370-4	56	56	1075087	866693.86	1008782	402741.09	897668.14	647864.52	430945.8	763463.88	565374.11	918331	0.240043533	0.61970999	850194.41	665195.86	Down	-0.354013586078774	Isoform 4 of Myelin basic protein OS=Mus musculus GN=Mbp
LMNA	P48678	14	14	44877.71	21543.36	26747.93	18170.79	27326.14	27625.86	22474.79	20284.57	25272.93	1146.43	0.240098559	0.619610446	27733.186	19360.916	Down	-0.518466152565009	Prelamin-A/C OS=Mus musculus GN=Lmna PE=1 SV=2
FDX1L	Q9CPW2-2	1	1	119131	102278	144648	59945	92093	79774	88732	250490	116554	298342	0.240613312	0.618680348	103619	166778.4	Up	0.686643887173315	Isoform 2 of Adrenodoxin-like protein, mitochondrial OS=Mus musculus GN=Fdx1l
RPS7	P62082	12	12	103621.58	108935.92	124861.25	90830.83	92927.5	91283.83	97536.92	176779.33	124228	142214.83	0.240765219	0.618406252	104235.42	126408.58	Up	0.278248868318877	40S ribosomal protein S7 OS=Mus musculus GN=Rps7 PE=2 SV=1
CBS	Q91WT9	3	3	25287.5	34656	32526.5	66602.5	32542	61735.5	44056	71136	61600	23606.5	0.24098993	0.618001105	38322.9	52426.8	Up	0.452097752130078	Cystathionine beta-synthase OS=Mus musculus GN=Cbs PE=2 SV=3
MMAB	Q9D273	1	1	267709	286598	435900	42340	216361	141004	205800	755038	355311	1314688	0.241216982	0.617592121	249781.6	554368.2	Up	1.15017729745046	Cob(I)yrinic acid a,c-diamide adenosyltransferase, mitochondrial OS=Mus musculus GN=Mmab PE=1 SV=1
VDAC2	Q60930	46	46	56734.83	57064.5	72651.8	55768.2	66706.04	57512.76	62697.2	76120.15	66711.2	101579.74	0.241651935	0.616809722	61785.074	72924.21	Up	0.239139497410069	Voltage-dependent anion-selective channel protein 2 OS=Mus musculus GN=Vdac2 PE=1 SV=2
COQ7	P97478	1	1	21881	23977	NA	17403	29773	21228	38641	NA	48149	NA	0.242138901	0.615935433	23258.5	36006	Up	0.63047927896592	Ubiquinone biosynthesis protein COQ7 homolog OS=Mus musculus GN=Coq7 PE=2 SV=3
LRPPRC	Q6PB66	1	1	48816	53210	75196	29833	55049	54918	54277	68320	100668	NA	0.242302623	0.615641884	52420.8	69545.75	Up	0.40782298216572	Leucine-rich PPR motif-containing protein, mitochondrial OS=Mus musculus GN=Lrpprc PE=1 SV=2
RPS21	Q9CQR2	9	9	71746.67	78471.44	123285.78	42076.22	69328	67283.56	70900.89	114873.22	117439.44	131501.78	0.242431613	0.615410749	76981.622	100399.78	Up	0.383170105148132	40S ribosomal protein S21 OS=Mus musculus GN=Rps21 PE=2 SV=1
ATP5G3	P56384	1	1	28529	66588	65210	76994	58632	66066	88272	78758	57885	67511	0.242542902	0.61521143	59190.6	71698.4	Up	0.276572843448332	ATP synthase F(0) complex subunit C3, mitochondrial OS=Mus musculus GN=Atp5g3 PE=2 SV=1
MAT2A	Q3THS6	1	1	81185	118500	74460	108207	78141	101408	86795	32724	85636	57520	0.242654961	0.615010824	92098.6	72816.6	Down	-0.338911846831334	S-adenosylmethionine synthase isoform type-2 OS=Mus musculus GN=Mat2a PE=2 SV=2
PRICKLE1	Q3U5C7	3	3	4077.67	6823.33	7087.33	10090	9359	11228.33	11487	4907	14732.67	NA	0.242693737	0.614941432	7487.466	10588.75	Up	0.499982837633279	Prickle-like protein 1 OS=Mus musculus GN=Prickle1 PE=2 SV=1
MYO18A	Q9JMH9-4	39	39	9323.47	14456.58	12043.13	23207.55	16772.18	21458.24	23205.71	16115.76	19049.66	14299.92	0.242699423	0.614931256	15160.582	18825.858	Up	0.312390479873263	Isoform 4 of Unconventional myosin-XVIIIa OS=Mus musculus GN=Myo18a
DOCK4	P59764	1	1	NA	NA	NA	7025	2677	9098	10448	NA	NA	NA	0.242787973	0.61477283	4851	9773	Up	1.01051931211461	Dedicator of cytokinesis protein 4 OS=Mus musculus GN=Dock4 PE=1 SV=1
NDUFA2	Q9CQ75	20	20	141912.5	127128.95	151711.35	88032.15	122064.95	115779.95	116246.55	223729.6	153349	167975.95	0.243646192	0.613240372	126169.98	155416.21	Up	0.300768299102124	NADH dehydrogenase [ubiquinone] 1 alpha subcomplex subunit 2 OS=Mus musculus GN=Ndufa2 PE=1 SV=3
HSP90AA1	P07901	40	40	39349.44	44909.97	46775.79	49058.79	45570.03	48951.91	45690.18	37250.62	36930.76	37884.5	0.243890381	0.612805328	45132.804	41341.594	Down	-0.12658239719072	Heat shock protein HSP 90-alpha OS=Mus musculus GN=Hsp90aa1 PE=1 SV=4
THRAP3	Q569Z6	2	2	82471	44733.5	30359.5	33338.5	67677	54580.5	44285	8772.5	51802.5	8101	0.244190587	0.612271082	51715.9	33508.3	Down	-0.626089408695261	Thyroid hormone receptor-associated protein 3 OS=Mus musculus GN=Thrap3 PE=1 SV=1
EIF4A1	P60843	9	9	34808.33	43224.89	50472.44	25963.11	33430.33	32917	39746.56	49019.56	47909.11	53629.78	0.244250476	0.612164581	37579.82	44644.402	Up	0.248521129367074	Eukaryotic initiation factor 4A-I OS=Mus musculus GN=Eif4a1 PE=1 SV=1
IDH3A	Q9D6R2	25	25	120439	133388.64	115199.16	179383.12	122028.36	139990.24	134505.28	80356.52	118960.2	93154.92	0.244414906	0.611872312	134087.66	113393.43	Down	-0.241839351437347	Isocitrate dehydrogenase [NAD] subunit alpha, mitochondrial OS=Mus musculus GN=Idh3a PE=1 SV=1
COA3	Q9D2R6	3	3	13659	14580.67	20627	9072.67	10212.33	9506.67	11592.67	30036.33	18524.33	29252.67	0.245296637	0.610308406	13630.334	19782.534	Up	0.537406322150055	Cytochrome c oxidase assembly factor 3 homolog, mitochondrial OS=Mus musculus GN=Coa3 PE=2 SV=1
VAMP3	P63024	1	1	896	1859	NA	5356	2153	3703	4242	NA	NA	NA	0.245408489	0.610110419	2566	3972.5	Up	0.630526049146199	Vesicle-associated membrane protein 3 OS=Mus musculus GN=Vamp3 PE=1 SV=1
RPS25	P62852	7	7	353954.14	375927.14	330350.71	193367.14	321491.29	245205.71	327565	466441.86	426904.86	436074.43	0.245774817	0.609462619	315018.08	380438.37	Up	0.27222811682710 5	40S ribosomal protein S25 OS=Mus musculus GN=Rps25 PE=1 SV=1
DAB2IP	Q3UHC7-4	6	6	45630	51883.33	74424.67	26577	58057.83	43281.67	59975.67	67383.33	62043.5	114240.67	0.24596982	0.609118177	51314.566	69384.968	Up	0.435254738955249	Isoform 4 of Disabled homolog 2-interacting protein OS=Mus musculus GN=Dab2ip
EXOC8	Q6PGF7	3	3	15081	26838.33	3822.67	31635.33	30648.33	39829	29887.67	23361	20163.67	35710	0.246080858	0.608922168	21605.132	29790.268	Up	0.463467057127221	Exocyst complex component 8 OS=Mus musculus GN=Exoc8 PE=1 SV=1
FECH	P22315	7	7	53028.71	41941.14	79764.29	33233.29	46687.71	40948	48156.14	98377.29	58423.57	140798.14	0.246425374	0.608314575	50931.028	77340.628	Up	0.602681641925617	Ferrochelatase, mitochondrial OS=Mus musculus GN=Fech PE=1 SV=2
SHANK2	Q80Z38	42	42	72667.62	74546.1	72367.64	54495.4	75753.29	48826.26	64302.98	66686.21	74081.48	60211.74	0.246801766	0.607651737	69966.01	62821.734	Down	-0.155390455749865	SH3 and multiple ankyrin repeat domains protein 2 OS=Mus musculus GN=Shank2 PE=1 SV=2
PTPRT	Q99M80-3	1	1	38081	51084	38642	27014	38453	62857	35208	74522	37972	NA	0.247456895	0.606500441	38654.8	52639.75	Up	0.445505063920273	Isoform 3 of Receptor-type tyrosine-protein phosphatase T OS=Mus musculus GN=Ptprt
SUGT1	Q9CX34	13	13	18992.83	18109	39570.67	8496.58	18803.17	14908.33	15076.25	52283.58	28915.67	76624.5	0.247885481	0.60574891	20794.45	37561.666	Up	0.853062526196387	Suppressor of G2 allele of SKP1 homolog OS=Mus musculus GN=Sugt1 PE=2 SV=3
CLTA	O08585	11	11	125172.73	96387.91	177180.09	130204.91	119256.82	115548.27	103739.82	236276.82	153699.18	269498.45	0.248366004	0.604907851	129640.49	175752.51	Up	0.439028875833105	Clathrin light chain A OS=Mus musculus GN=Clta PE=1 SV=2
PPA2	Q91VM9-2	7	7	50847.14	60578.14	64848.71	54874.57	50944.29	54136.14	57569.86	81496.71	61581.14	154571.86	0.248382411	0.604879162	56418.57	81871.142	Up	0.537184919807651	Isoform 2 of Inorganic pyrophosphatase 2, mitochondrial OS=Mus musculus GN=Ppa2
SYT1	P46096	14	14	35304.21	55252.79	66869.71	116729.57	44071.29	72096.5	68840	106937.86	57782.79	153758.79	0.248411352	0.604828561	63645.514	91883.188	Up	0.529742083084219	Synaptotagmin-1 OS=Mus musculus GN=Syt1 PE=1 SV=1
ATXN2L	Q7TQH0	9	9	25683.44	33955.11	28528.44	30593.78	27842.11	30945.22	32775.67	32012.89	40850	26908.56	0.248497641	0.60467773	29320.576	32698.468	Up	0.157309598296159	Ataxin-2-like protein OS=Mus musculus GN=Atxn2l PE=1 SV=1

SARS	P26638	5	5	76590.33	64234	39653.33	138515.67	107643	122489.67	108846.33	86663	103488	125387	0.249516115	0.6029014	85327.266	109374.8	Up	0.358201650886018	Serine--tRNA ligase, cytoplasmic OS=Mus musculus GN=Sars PE=2 SV=3
SH3GLB1	Q9JK48	10	10	39485.6	39227.9	48859.1	27678.5	43337.2	35986.9	40473.4	56610.7	45819.1	103697	0.24958887	0.602774785	39717.66	56517.42	Up	0.508914980908142	Endophilin-B1 OS=Mus musculus GN=Sh3glb1 PE=1 SV=1
FKBP1A	P26883	4	4	42613.67	30303.67	52214.67	28374	33566	31750.67	30766	95911.67	36390	116140.33	0.249589117	0.602774356	37414.402	62191.734	Up	0.73312912576913	Peptidyl-prolyl cis-trans isomerase FKBP1A OS=Mus musculus GN=Fkbp1a PE=1 SV=2
ENDOG	O08600	1	1	5674	3581	NA	3307	3004	3080	3628	NA	1487	NA	0.249653123	0.602662996	3891.5	2731.6667	Down	-0.510544908519638	Endonuclease G, mitochondrial OS=Mus musculus GN=Endog PE=2 SV=1
PPP1R7	Q3UM45	9	9	103072.56	94256.67	123824.33	46686.56	101730.44	75881	79377.89	175727.33	117285.11	210716.67	0.249730733	0.602528008	93914.112	131797.6	Up	0.488910233694587	Protein phosphatase 1 regulatory subunit 7 OS=Mus musculus GN=Ppp1r7 PE=1 SV=2
GDAP1	O88741-2	1	1	4861	7599	NA	22718	18089	18536	20188	NA	NA	NA	0.249995607	0.602067623	13316.75	19362	Up	0.539985953125634	Isoform 2 of Ganglioside-induced differentiation-associated protein 1 OS=Mus musculus GN=Gdap1
PSAP	Q61207	3	3	22845.67	22280.33	60871.67	11728.33	19376	20500	18290.67	90811.33	33177.33	149796.67	0.250033091	0.602002511	27420.4	62515.2	Up	1.18895739370744	Sulfated glycoprotein 1 OS=Mus musculus GN=Psap PE=1 SV=2
GCLM	O09172	1	1	74915	90279	118053	40314	74194	60359	81417	89440	151635	229157	0.250203409	0.601706777	79551	122401.6	Up	0.621670445409707	Glutamate--cysteine ligase regulatory subunit OS=Mus musculus GN=Gclm PE=2 SV=1
NDRG2	Q9QYG0	6	6	20173.83	20881.17	27972.83	31944.33	20060	24201.67	27456.33	35619.5	20536.17	44580.33	0.250369784	0.601418085	24206.432	30478.8	Up	0.332415659263809	Protein NDRG2 OS=Mus musculus GN=Ndrg2 PE=1 SV=1
BPNT1	Q9Z0S1	2	2	41481	47818	52070	58280.5	52094.5	70038	57646.5	68328.5	63019	35986.5	0.25041176	0.60134528	50348.8	59003.7	Up	0.228848031904199	3'(2'),5'-bisphosphate nucleotidase 1 OS=Mus musculus GN=Bpnt1 PE=1 SV=2
ATP6V1A	P50516-2	35	35	88803.47	96407.38	96788.62	89026.76	84770.62	101182.29	98311.97	122630.76	77607.79	105586.53	0.250798971	0.600674249	91159.37	101063.87	Up	0.148804442820844	Isoform 2 of V-type proton ATPase catalytic subunit A OS=Mus musculus GN=Atp6v1a
LRRC47	Q505F5	4	4	36932.5	46355.75	13862.75	38732	42335.75	43995	38897.5	NA	48291.5	NA	0.250807188	0.600660021	35643.75	43728	Up	0.294908237378774	Leucine-rich repeat-containing protein 47 OS=Mus musculus GN=Lrrc47 PE=2 SV=1
MARK3	Q03141-2	7	7	20449.43	22105.14	32000.71	17259.86	24574.43	23372.86	25004.43	54292.57	39052.71	19306.86	0.251025434	0.600282273	23277.914	32205.886	Up	0.468362601686839	Isoform 2 of MAP/microtubule affinity-regulating kinase 3 OS=Mus musculus GN=Mark3
MAPRE1	Q61166	13	13	60909.15	71321.46	66195.54	62949	59800.85	62269.46	70698.15	113136.23	66313.54	71298.77	0.251223416	0.599939883	64235.2	76743.23	Up	0.256675396007289	Microtubule-associated protein RP/EB family member 1 OS=Mus musculus GN=Mapre1 PE=1 SV=3
POLR2M	Q6P6I6	1	1	6783	3364	NA	7439	8356	11858	6749	NA	21787	NA	0.251234466	0.599920781	6485.5	13464.667	Up	1.05388880616866	DNA-directed RNA polymerase II subunit GRINL1A OS=Mus musculus GN=Polr2m PE=2 SV=2
PEX14	Q9R0A0	1	1	3391	4145	1436	9571	4019	7948	5849	2790	8131	8570	0.251629421	0.599238582	4512.4	6657.6	Up	0.561107232930995	Peroxisomal membrane protein PEX14 OS=Mus musculus GN=Pex14 PE=1 SV=1
DAB2IP	Q3UHC7-3	5	5	18477	25801	32422.4	17113	29503.2	27887.8	36884	24481.6	37773.8	22932.2	0.252240559	0.59818508	24663.32	29991.88	Up	0.282204940291366	Isoform 3 of Disabled homolog 2-interacting protein OS=Mus musculus GN=Dab2ip
RPL18A	P62717	9	9	105594.78	136411	105092.56	101633.44	101852.33	92537.11	118199.33	85406.11	115840.78	54649.89	0.252558743	0.597637593	110116.82	93326.644	Down	-0.238673956365503	60S ribosomal protein L18a OS=Mus musculus GN=Rpl18a PE=1 SV=1
FAM134A	Q6NS82-3	2	2	634	1731	3054	2707.5	2306.5	3169	2858	3088.5	1186	8006	0.252761894	0.597288399	2086.6	3661.5	Up	0.811280732633122	Isoform 3 of Protein FAM134A OS=Mus musculus GN=Fam134a
PDDC1	Q8BFQ8	5	5	51564.6	35313.2	61411.6	19261.6	35883.6	28706.2	33271.2	101758	56157	211781	0.253153281	0.59661644	40686.92	86334.68	Up	1.0853751231921	Parkinson disease 7 domain-containing protein 1 OS=Mus musculus GN=Pddc1 PE=1 SV=1
RNF20	Q5DTM8	1	1	23454	18172	NA	20082	18089	18918	26576	NA	31307	NA	0.25335392	0.596272372	19949.25	25600.333	Up	0.359828086422219	E3 ubiquitin-protein ligase BRE1A OS=Mus musculus GN=Rnf20 PE=2 SV=2
SPEG	Q62407-4	5	5	11632.8	11668	1048.2	18851.6	17270.2	21835.6	20293.4	11656.6	16524.6	13177.6	0.253572614	0.595897652	12094.16	16697.56	Up	0.465326727923428	Isoform 4 of Striated muscle-specific serine/threonine-protein kinase OS=Mus musculus GN=Speg
KCNA1	P16388	3	3	662.67	1989.67	448.67	3236.33	2916	3735	3527.33	NA	1668.33	NA	0.253848933	0.595424657	1850.668	2976.8867	Up	0.685758191042051	Potassium voltage-gated channel subfamily A member 1 OS=Mus musculus GN=Kcna1 PE=1 SV=1
RAB7	P51150	4	4	25176.25	33008	40940.75	41409.25	30599.75	39019.25	37448.5	50016.25	37656	139301.25	0.253974192	0.595210413	34226.8	60688.25	Up	0.826290804473437	Ras-related protein Rab-7a OS=Mus musculus GN=Rab7a PE=1 SV=2
AIFM1	Q9Z0X1	15	15	43417.73	46555.27	48696.67	60348	51735.73	60157.67	55090.4	69076.8	53806.13	43853.67	0.254093128	0.59500708	50150.68	56396.934	Up	0.169347470462706	Apoptosis-inducing factor 1, mitochondrial OS=Mus musculus GN=Aifm1 PE=1 SV=1
PPP1R21	Q3TDD9-3	3	3	10543.67	12458.33	NA	15832.33	14722.67	19561.33	17672.67	17482.67	10634	NA	0.254430706	0.594430477	13389.25	16337.668	Up	0.287126876918812	Isoform 3 of Protein phosphatase 1 regulatory subunit 21 OS=Mus musculus GN=Ppp1r21
NOVA1	Q9JKN6	2	2	44236	51249.5	27344.5	30464.5	50178.5	45807.5	44260	NA	54195.5	NA	0.254700655	0.593969938	40694.6	48087.667	Up	0.240829557047862	RNA-binding protein Nova-1 OS=Mus musculus GN=Nova1 PE=1 SV=2
PFN2	Q9JJV2	34	34	170254.7	155969.82	259202.27	116196.88	159182.45	160020.09	168655.15	233317.36	209141.03	308477.64	0.255015557	0.593433325	172161.22	215922.25	Up	0.326751702156859	Profilin-2 OS=Mus musculus GN=Pfn2 PE=1 SV=3
SH3BGRL	Q9JJU8	4	4	42249.5	42708	54634.25	49925.25	37247	42469	44003	53692.5	53660.75	61802.75	0.255079751	0.593324015	45352.8	51125.6	Up	0.172854246448539	SH3 domain-binding glutamic acid-rich-like protein OS=Mus musculus GN=Sh3bgrl PE=3 SV=1
NCS1	Q8BNY6	2	2	9579.5	11657	22370.5	5177	10722.5	10809	10656	29496	11067	39086.5	0.25527096	0.592998589	11901.3	20222.9	Up	0.764870726857516	Neuronal calcium sensor 1 OS=Mus musculus GN=Ncs1 PE=2 SV=3
LSP1	P19973	1	1	2991	4343	NA	4532	2611	4044	4379	NA	7341	NA	0.255590037	0.592456079	3619.25	5254.6667	Up	0.53790848332014	Lymphocyte-specific protein 1 OS=Mus musculus GN=Lsp1 PE=1 SV=2
FAM185A	Q7TPD2	3	3	66532.67	56109.67	97192.33	27327.67	65552.33	49469	63752	98438.67	84247.67	200298	0.256088955	0.591609152	62542.934	99241.068	Up	0.666090360892754	Protein FAM185A OS=Mus musculus GN=Fam185a PE=2 SV=1
EXOG	Q8C163	3	3	18208.33	22150.67	28841.67	25903.67	20656	28019.33	19715.33	38724.33	28714.67	NA	0.256256868	0.591324486	23152.068	28793.415	Up	0.314597843408793	Nuclease EXOG, mitochondrial OS=Mus musculus GN=Exog PE=2 SV=1
HOOK3	Q8BUK6	1	1	35508	67714	27566	61367	64874	56124	60672	NA	89477	NA	0.256353407	0.591160907	51405.8	68757.667	Up	0.419589442293998	Protein Hook homolog 3 OS=Mus musculus GN=Hook3 PE=1 SV=2
LYZ2	P08905	1	1	8714	6176	10494	11192	12674	11319	11195	NA	18887	NA	0.25647004	0.59096336	9850	13800.333	Up	0.486507484607235	Lysozyme C-2 OS=Mus musculus GN=Lyz2 PE=1 SV=2
TAOK2	Q6ZQ29	1	1	8416	18965	NA	12092	15550	18644	NA	NA	28449	NA	0.256758164	0.590475739	13755.75	23546.5	Up	0.775477829266376	Serine/threonine-protein kinase TAO2 OS=Mus musculus GN=Taok2 PE=2 SV=3
SOGA1	E1U8D0	1	1	NA	NA	NA	7189	2780	8143	10617	NA	NA	NA	0.256814497	0.590380464	4984.5	9380	Up	0.912139128989551	Protein SOGA1 OS=Mus musculus GN=Soga1 PE=1 SV=3
SCP2	P32020	14	14	75764.86	86024.5	133295.5	57202	70008.71	65266.93	77983.07	159332.57	104813.5	282453	0.256883923	0.590263074	84459.114	137969.81	Up	0.708027640573403	Non-specific lipid-transfer protein OS=Mus musculus GN=Scp2 PE=1 SV=3
DPYSL3	Q62188	100	100	96174.67	110819.72	119542.16	87836.37	90535.19	97269.2	116055.44	113091.27	124832.15	101832.42	0.257220894	0.589693757	100981.62	110616.1	Up	0.131468574958529	Dihydropyrimidinase-related protein 3 OS=Mus musculus GN=Dpysl3 PE=1 SV=1
TNC	Q80YX1-3	22	22	5509.36	5106.43	2899.21	8477	7061.5	8615.43	10606.71	3807.93	9069.21	NA	0.257297548	0.589564353	5810.7	8024.82	Up	0.465757060517824	Isoform 3 of Tenascin OS=Mus musculus GN=Tnc
SMARCE1	O54941	3	3	26689	14875	6685.67	12564.33	15576.67	15480.67	12343.67	900.33	10080	NA	0.257350235	0.589475431	15278.134	9701.1675	Down	-0.655238064164132	SWI/SNF-related matrix-associated actin-dependent regulator of chromatin subfamily E member 1 OS=Mus musculus GN=Smarce1 PE=1 SV=1
NCALD	Q91X97	3	3	85728.33	86217.33	118228	76203.33	81233	72753.33	87970.67	169349	95463	291982.33	0.257771388	0.58876529	89521.998	143503.67	Up	0.68077345238717	Neurocalcin-delta OS=Mus musculus GN=Ncald PE=1 SV=4
FXYD7	P59648	1	1	49733	111029	187019	186051	151228	193422	225818	104072	144567	236168	0.257899733	0.588549108	137012	180809.4	Up	0.4001674280776	FXYD domain-containing ion transport regulator 7 OS=Mus musculus GN=Fxyd7 PE=2 SV=1
SHANK2	Q80Z38-2	40	40	75380.93	77320.7	73978.52	56295.2	78426.3	50465.8	66760.48	70201.7	75849.6	62151.68	0.258197345	0.588048227	72280.33	65085.852	Down	-0.151259120466461	Isoform 2 of SH3 and multiple ankyrin repeat domains protein 2 OS=Mus musculus GN=Shank2
SCP2	P32020-2	13	13	81117.23	91913.15	143287	60617.08	74968.08	69371.62	83366	171588.92	111733.46	304180.15	0.258373779	0.587751562	90380.508	148048.03	Up	0.711981722359574	Isoform SCP2 of Non-specific lipid-transfer protein OS=Mus musculus GN=Scp2
MBP	P04370-8	45	45	1245472.1	999284.24	1184950.2	449423.93	1030940.3	749817.18	483616.8	885047.62	640128.69	1083505.2	0.258453857	0.587616982	982014.16	768423.09	Down	-0.353842953918303	Isoform 8 of Myelin basic protein OS=Mus musculus GN=Mbp
STRIP1	Q8C079	2	2	21275.5	23623.5	23105.5	59352	35247.5	52703	49954.5	23180	54978	NA	0.259074573	0.586575209	32520.8	45203.875	Up	0.475083702083322	Striatin-interacting protein 1 OS=Mus musculus GN=Strip1 PE=1 SV=2
NDUFA8	Q9DCJ5	16	16	100003.19	97790.31	127965.06	48286.56	90210.25	77167.69	88441.88	154965.5	116427.25	151758.88	0.259162341	0.586428106	92851.074	117752.24	Up	0.342764001583019	NADH dehydrogenase [ubiquinone] 1 alpha subcomplex subunit 8 OS=Mus musculus GN=Ndufa8 PE=1 SV=3
MAP6	Q7TSJ2-3	26	26	93154.08	88340.69	88511.58	74122.81	95909.08	91429.85	94821.81	81663.81	132338.27	98912.96	0.259200741	0.586363762	88007.648	99833.34	Up	0.181892791904262	Isoform 3 of Microtubule-associated protein 6 OS=Mus musculus GN=Map6
WDR1	O88342	22	22	43129.09	51085.86	48937.23	72492.77	48010.95	60905.14	61688.05	58577.14	54285.95	62264.05	0.259976133	0.58506652	52731.18	59544.066	Up	0.175301458974432	WD repeat-containing protein 1 OS=Mus musculus GN=Wdr1 PE=1 SV=3
PPP2R5C	Q60996	1	1	15880	24410	23371	70476	40158	59549	80974	NA	31953	NA	0.260534849	0.584134178	34859	57492	Up	0.721830035834014	Serine/threonine-protein phosphatase 2A 56 kDa regulatory subunit gamma isoform OS=Mus musculus GN=Ppp2r5c PE=1 SV=2
MECP2	Q9Z2D6	4	4	90780.5	64463	37581.25	40865.5	73008	56469.5	52266.5	51613.75	50049.5	25926.75	0.26093261	0.583471641	61339.65	47265.2	Down	-0.376041574934237	Methyl-CpG-binding protein 2 OS=Mus musculus GN=Mecp2 PE=1 SV=1
CDKL5	Q3UTQ8	2	2	53126.5	42472.5	69627.5	16297.5	38449.5	36200.5	43650	55855	72713	139884	0.261116818	0.583165155	43994.7	69660.5	Up	0.663011094041566	Cyclin-dependent kinase-like 5 OS=Mus musculus GN=Cdkl5 PE=2 SV=1
LRRC7	Q80TE7	40	40	35051.59	40737.59	36919.43	47401.62	45468.14	50112.41	52577.78	42684	52640.19	33958.14	0.261218791	0.582995585	41115.674	46394.504	Up	0.17426543163722	Leucine-rich repeat-containing protein 7 OS=Mus musculus GN=Lrrc7 PE=1 SV=2
DTD1	Q9DD18-2	3	3	43514.67	41368.67	32007.33	43176.33	50275.67	57741.67	53228.67	36796	53920	39801	0.26131387	0.582837539	42068.534	48297.468	Up	0.199206013507063	Isoform 2 of D-tyrosyl-tRNA(Tyr) deacylase 1 OS=Mus musculus GN=Dtd1
DUSP23	Q6NT99	1	1	65279	61375	65900	42061	70055	41898	68161	85670	80324	166730	0.262228556	0.581320016	60934	88556.6	Up	0.539352384149737	Dual specificity protein phosphatase 23 OS=Mus musculus GN=Dusp23 PE=2 SV=1
CTSD	P18242	14	14	47169.93	43137.64	55324.86	37578.21	41740	41136.21	45742.86	73438.93	41701.36	75172.14	0.26240342	0.581030508	44990.128	55438.3	Up	0.301274546823412	Cathepsin D OS=Mus musculus GN=Ctsd PE=1 SV=1
RDX	P26043	3	3	64456.33	76732.67	80343.67	66320.67	70900.33	87027	54578.67	111414	71830.33	196807.33	0.262458375	0.580939565	71750.734	104331.47	Up	0.540108839645435	Radixin OS=Mus musculus GN=Rdx PE=1 SV=3
GLRX	Q9QUH0	9	9	39896.25	40535.62	57918.12	40344.75	38387.38	38405	38469	73241.38	48300.88	117228.5	0.26247849	0.580906281	43416.424	63128.952	Up	0.540060898142493	Glutaredoxin-1 OS=Mus musculus GN=Glrx PE=1 SV=3
NDUFB2	Q9CPU2	1	1	26946	36234	48407	41182	30981	40772	42301	59238	38870	36056	0.263110238	0.579862253	36750	43447.4	Up	0.241525595230171	NADH dehydrogenase [ubiquinone] 1 beta subcomplex subunit 2, mitochondrial OS=Mus musculus GN=Ndufb2 PE=3 SV=1
ABAT	P61922	38	38	48858.71	51075.05	55458.47	56689.92	46114.03	58644.68	47821.24	72088.79	45691.03	71400.89	0.263156737	0.579785507	51639.236	59129.326	Up	0.195406176086439	4-aminobutyrate aminotransferase, mitochondrial OS=Mus musculus GN=Abat PE=1 SV=1
SLC6A17	Q8BJI1	3	3	1641	4337	1695	10601	4366.5	7547.5	8041	NA	4999.5	NA	0.263171234	0.579761584	4528.1	6862.6667	Up	0.599863462365724	Sodium-dependent neutral amino acid transporter SLC6A17 OS=Mus musculus GN=Slc6a17 PE=1 SV=1
CMPK2	Q3U5Q7	2	2	20648.5	25938.5	12961	18121.5	19116	27610.5	17785	NA	32178.5	NA	0.263284334	0.579574982	19357.1	25858	Up	0.417747863243986	UMP-CMP kinase 2, mitochondrial OS=Mus musculus GN=Cmpk2 PE=2 SV=2
LAMTOR2	Q9JHS3	1	1	15478	16519	29683	9681	12798	14279	13680	36143	20182	68018	0.263299573	0.579549846	16831.8	30460.4	Up	0.855745419785961	Ragulator complex protein LAMTOR2 OS=Mus musculus GN=Lamtor2 PE=1 SV=1
KCNIP3	Q9QXT8	1	1	77431	149241	173700	24270	156742	86980	189862	93251	222580	256289	0.263447868	0.579305312	116276.8	169792.4	Up	0.54620861113233	Calsenilin OS=Mus musculus GN=Kcnip3 PE=1 SV=2
LARP6	Q8BN59	4	4	6590	5010	9153	4802	5081	6716	6570	7140	12993	NA	0.264146949	0.578154401	6127.2	8354.75	Up	0.447368715448942	La-related protein 6 OS=Mus musculus GN=Larp6 PE=1 SV=1
WDR77	Q99J09	3	3	3080.33	2588.67	4176.67	2171	3073.67	3513	3512.67	1931.33	6262.33	4830	0.264427974	0.577692602	3018.068	4009.866	Up	0.409928714565524	Methylosome protein 50 OS=Mus musculus GN=Wdr77 PE=1 SV=1
ZFP706	Q9D115	3	3	12499.67	11625	16618.67	10582	13279.33	11946.67	14577	11131	19373.33	22930.33	0.264529396	0.577526059	12920.934	15991.666	Up	0.30760988539515	Zinc finger protein 706 OS=Mus musculus GN=Znf706 PE=2 SV=1
PSMA7	Q9Z2U0	9	9	41398.89	41550	40235.44	37318.11	41295	41764.44	41373.56	68904.56	50115.44	36742.56	0.2646496	0.577328758	40359.488	47780.112	Up	0.24350236303089	Proteasome subunit alpha type-7 OS=Mus musculus GN=Psma7 PE=1 SV=1
MRPL11	Q9CQF0	2	2	29326	24329.5	38364	24273.5	30281	29389.5	27393.5	31071.5	38697	56277.5	0.26528075	0.576294263	29314.8	36565.8	Up	0.318865712190968	39S ribosomal protein L11, mitochondrial OS=Mus musculus GN=Mrpl11 PE=2 SV=1
MBP	P04370-6	45	45	1280632.7	1027673.2	1207891.6	455009.44	1060267.8	762692.16	498128.09	908953.22	658690.49	1117893.7	0.265496867	0.575940599	1006295	789271.53	Down	-0.350459605346556	Isoform 6 of Myelin basic protein OS=Mus musculus GN=Mbp
UBTD1	Q91WB7	1	1	4874	9500	NA	13576	11180	6277	5462	NA	9355	NA	0.266342954	0.574558788	9782.5	7031.3333	Down	-0.476404915402465	Ubiquitin domain-containing protein 1 OS=Mus musculus GN=Ubtd1 PE=2 SV=1
SPIRE1	Q52KF3-3	1	1	9954	11364	8583	12425	12787	11082	14465	NA	12325	NA	0.26674004	0.573911789	11022.6	12624	Up	0.19570454493247	Isoform 3 of Protein spire homolog 1 OS=Mus musculus GN=Spire1
EIF1AX	Q8BMJ3	8	8	22073.17	23437	28559.83	29806.67	18868.17	24831.5	23936.83	31199	24996.67	44027	0.266973915	0.573531169	24548.968	29798.2	Up	0.279562808217517	Eukaryotic translation initiation factor 1A, X-chromosomal OS=Mus musculus GN=Eif1ax PE=2 SV=3
AUH	Q9JLZ3-2	1	1	149599	130033	91401	135802	156368	180067	135938	151347	136993	NA	0.267204013	0.573157024	132640.6	151086.25	Up	0.187849932240308	Isoform 2 of Methylglutaconyl-CoA hydratase, mitochondrial OS=Mus musculus GN=Auh
EIF3A	P23116	7	7	18581.71	26696.29	22309.86	40929	26732.86	36248.29	37837.57	29416.29	31727.14	26428.43	0.267251469	0.573079899	27049.944	32331.544	Up	0.257316798004757	Eukaryotic translation initiation factor 3 subunit A OS=Mus musculus GN=Eif3a PE=1 SV=5
PFN2	Q9JJV2-3	29	29	196768.61	180797.29	299618.93	134537.57	183479.71	185346.11	194944.14	262153.89	242117.14	348611.79	0.267998695	0.571867321	199040.42	246634.61	Up	0.309313840020085	Isoform 3 of Profilin-2 OS=Mus musculus GN=Pfn2
CAP2	Q9CYT6	3	3	70979.67	67070.33	80211	61918	60875	81249.33	58502.67	87119.67	66741.33	89871	0.268024426	0.571825625	68210.8	76696.8	Up	0.169166202672655	Adenylyl cyclase-associated protein 2 OS=Mus musculus GN=Cap2 PE=1 SV=1
PRKACA	P05132	1	1	265838	320090	258545	251357	301178	264211	250281	112641	341792	164271	0.268108458	0.571689486	279401.6	226639.2	Down	-0.301942868043275	cAMP-dependent protein kinase catalytic subunit alpha OS=Mus musculus GN=Prkaca PE=1 SV=3
HBA-A1	P01942	17	17	182950.53	123768.35	136450.76	60377.82	185810.65	83952.53	98706.29	59782.71	132032.47	147265.71	0.268143222	0.571633176	137871.62	104347.94	Down	-0.401923391444163	Hemoglobin subunit alpha OS=Mus musculus GN=Hba PE=1 SV=2
SIPA1L1	Q8C0T5-2	21	21	40671.81	46765.62	46750.38	38239.14	60671.76	45325.38	59853.62	54891.19	64105.33	42641.33	0.268351573	0.571295855	46619.742	53363.37	Up	0.194908757962853	Isoform 2 of Signal-induced proliferation-associated 1-like protein 1 OS=Mus musculus GN=Sipa1l1
ARPC1A	Q9R0Q6	4	4	41058	33352.5	44372.5	43342.25	36170.5	45581.25	42505.25	39201.5	44112.5	95913.25	0.268430882	0.571167521	39659.15	53462.75	Up	0.430880291235132	Actin-related protein 2/3 complex subunit 1A OS=Mus musculus GN=Arpc1a PE=1 SV=1
EPB41L3	Q9WV92-5	38	38	22118.37	25429.84	22659.63	29381.24	24921.29	30928.29	29188.05	21418.34	27790.13	27458.82	0.268667523	0.570784829	24902.074	27356.726	Up	0.135629677669277	Isoform 5 of Band 4.1-like protein 3 OS=Mus musculus GN=Epb41l3
PDE4A	O89084	2	2	16549.5	19716	31835.5	8168.5	16023.5	16493.5	12776	101865	22347.5	46104	0.268688035	0.570751672	18458.6	39917.2	Up	1.11271739074848	cAMP-specific 3',5'-cyclic phosphodiesterase 4A OS=Mus musculus GN=Pde4a PE=2 SV=2
RPS18	P62270	12	12	76974.67	86540.92	90819.5	74582.83	68128.67	69196.58	75772	76623.92	81534.33	62462	0.268689027	0.570750069	79409.318	73117.766	Down	-0.119086313388467	40S ribosomal protein S18 OS=Mus musculus GN=Rps18 PE=1 SV=3
ATXN2L	Q7TQH0-2	11	11	23431.55	29308.82	25366.09	26763	24592.36	27641.36	28566.82	30376.45	36280	22016.09	0.269504923	0.569433298	25892.364	28976.144	Up	0.162338930221119	Isoform 2 of Ataxin-2-like protein OS=Mus musculus GN=Atxn2l
ARHGAP33	Q80YF9	3	3	10289.33	11386.33	NA	20015	21592.67	22636.67	25902.67	NA	15145.33	NA	0.269614861	0.569256173	15820.833	21228.223	Up	0.424158114806282	Rho GTPase-activating protein 33 OS=Mus musculus GN=Arhgap33 PE=1 SV=1
PSMF1	Q8BHL8	4	4	12796	21413.25	11488.5	22443.5	16390	23286.5	20335.25	16588.25	19964.25	NA	0.269995177	0.568643993	16906.25	20043.563	Up	0.245582263920277	Proteasome inhibitor PI31 subunit OS=Mus musculus GN=Psmf1 PE=2 SV=1
RPS9	Q6ZWN5	6	6	72309.5	85351.83	98208.33	74400.83	68720.33	79904.33	77286.5	86439.83	93913.5	105161.17	0.270102423	0.56847152	79798.164	88541.066	Up	0.149991189795051	40S ribosomal protein S9 OS=Mus musculus GN=Rps9 PE=1 SV=3
MRPL58	Q8R035	6	6	41265.5	52758.83	38238.83	50122.83	48046.17	52407.33	48956.17	19189.67	43258.67	17176.67	0.270210559	0.568297684	46086.432	36197.702	Down	-0.348443967746147	Peptidyl-tRNA hydrolase ICT1, mitochondrial OS=Mus musculus GN=Ict1 PE=1 SV=1
LAP3	Q9CPY7-2	15	15	51128.33	50316.47	54816.6	40272.07	56074.07	53057.27	54909.53	59751.2	58937.8	47231.2	0.270322598	0.568117647	50521.508	54777.4	Up	0.116683088091478	Isoform 2 of Cytosol aminopeptidase OS=Mus musculus GN=Lap3
SLC30A3	P97441	1	1	498622	462380	439972	622605	322644	376755	447903	409598	491602	233341	0.270837158	0.567291753	469244.6	391839.8	Down	-0.260076200177815	Zinc transporter 3 OS=Mus musculus GN=Slc30a3 PE=1 SV=1
TRIM46	Q7TNM2	3	3	782.67	1757.33	NA	5160	2501.33	3814.67	3812.67	NA	NA	NA	0.270887163	0.567211575	2550.3325	3813.67	Up	0.580494660524857	Tripartite motif-containing protein 46 OS=Mus musculus GN=Trim46 PE=2 SV=1
RPL11	Q9CXW4	10	10	112509.3	127277	154297.1	115264	110918.5	108526.6	126470.1	182932.4	135660.6	157925.4	0.271318174	0.566521114	124053.18	142303.02	Up	0.198007561532056	60S ribosomal protein L11 OS=Mus musculus GN=Rpl11 PE=1 SV=4
ACTBL2	Q8BFZ3	17	17	193343.59	233806	216825.35	279100.88	175927.59	218886.88	250032	117269.94	200133.76	121623.76	0.271595325	0.56607771	219800.68	181589.27	Down	-0.275516921422918	Beta-actin-like protein 2 OS=Mus musculus GN=Actbl2 PE=1 SV=1
MARK3	Q03141	8	8	24779.25	28432	38664	22682.75	30387.88	29885.75	31845.5	61587.12	43358.75	23037.62	0.271650689	0.565989189	28989.176	37942.948	Up	0.388317447918798	MAP/microtubule affinity-regulating kinase 3 OS=Mus musculus GN=Mark3 PE=1 SV=2
MARK3	Q03141-3	8	8	24779.25	28432	38664	22682.75	30387.88	29885.75	31845.5	61587.12	43358.75	23037.62	0.271650689	0.565989189	28989.176	37942.948	Up	0.388317447918798	Isoform 3 of MAP/microtubule affinity-regulating kinase 3 OS=Mus musculus GN=Mark3
LIMCH1	Q3UH68	4	4	1717.33	2712	853	3312.67	2742.67	3390.67	3870.67	1844	2907.33	NA	0.271675936	0.565948827	2267.534	3003.1675	Up	0.405360759589935	LIM and calponin homology domains-containing protein 1 OS=Mus musculus GN=Limch1 PE=1 SV=2
COPS3	O88543	3	3	3784.33	6092.33	627	21050	10947.67	14885	24932	NA	8401.33	NA	0.271788693	0.565768614	8500.266	16072.777	Up	0.919039291069336	COP9 signalosome complex subunit 3 OS=Mus musculus GN=Cops3 PE=1 SV=3
HCFC1	Q61191	3	3	44032.33	34935.33	57769.67	16457	33896	31041	33836	90618	48052.67	59832	0.271923866	0.565552674	37418.066	52675.934	Up	0.493408996787308	Host cell factor 1 OS=Mus musculus GN=Hcfc1 PE=1 SV=2
ZNFX1	Q8R151	1	1	1624	9737	3071	11969	10400	14640	13483	4055	12616	NA	0.271931565	0.565540379	7360.2	11198.5	Up	0.605488626725313	NFX1-type zinc finger-containing protein 1 OS=Mus musculus GN=Znfx1 PE=2 SV=3
ZC2HC1A	Q8BJH1	12	12	97521.58	101012.08	93040.83	58588.67	80762.67	80688.08	100493.33	90033.25	132029.75	96238.67	0.272243884	0.565041868	86185.166	99896.616	Up	0.212996230315629	Zinc finger C2HC domain-containing protein 1A OS=Mus musculus GN=Zc2hc1a PE=2 SV=1

TKT	P40142	36	36	55219.63	74654.63	76114.71	79636.97	66689.49	74047.71	92635.46	79421.57	71023.69	70469.77	0.272358478	0.564859101	70463.086	77519.64	Up	0.137694210453643	Transketolase OS=Mus musculus GN=Tkt PE=1 SV=1
VBP1	P61759	4	4	70890.25	76297.75	137009.5	40921.25	72543.5	62898	76660	154722.75	87937	188739.5	0.272401637	0.564790287	79532.45	114191.45	Up	0.521839115028515	Prefoldin subunit 3 OS=Mus musculus GN=Vbp1 PE=2 SV=2
CKAP5	A2AGT5-3	8	8	10983.75	22392	22336.25	36614.25	24320.75	28894.62	32950.38	28275.38	23180.62	30172.25	0.272608412	0.564460746	23329.4	28694.65	Up	0.298632573718177	Isoform 3 of Cytoskeleton-associated protein 5 OS=Mus musculus GN=Ckap5
SNX27	Q3UHD6	3	3	8240.33	10203.33	3646.33	28123	13041.33	23496	19308	12730.33	17856	NA	0.272776605	0.56419288	12650.864	18347.583	Up	0.536354066124361	Sorting nexin-27 OS=Mus musculus GN=Snx27 PE=1 SV=2
CRK	Q64010	9	9	48178.11	59117.89	53866.78	38973.33	45758.44	48899.78	52965.11	61449	48109.67	60918.22	0.272868386	0.564046778	49178.91	54468.356	Up	0.147378564022745	Adapter molecule crk OS=Mus musculus GN=Crk PE=1 SV=1
PPIF	Q99KR7	9	9	106550.22	102879.11	97986.22	72851.89	97282	99428.56	94348.33	85546.78	137851.22	134137.56	0.273064822	0.563734245	95509.888	110262.49	Up	0.207220080419722	Peptidyl-prolyl cis-trans isomerase F, mitochondrial OS=Mus musculus GN=Ppif PE=1 SV=1
EFNB2	P52800	1	1	5086	11960	8013	29978	17204	23088	16313	NA	21652	NA	0.273114566	0.563655137	14448.2	20351	Up	0.494209918200144	Ephrin-B2 OS=Mus musculus GN=Efnb2 PE=1 SV=1
ATP8A2	P98200	2	2	679	1201	1813.5	6184.5	2424	6840.5	2812.5	NA	3619	NA	0.273154859	0.56359107	2460.4	4424	Up	0.846458504518459	Probable phospholipid-transporting ATPase IB OS=Mus musculus GN=Atp8a2 PE=2 SV=1
CENPV	Q9CXS4-2	10	10	21828.29	11056.86	1745.29	5761.71	16192.29	12697.43	9790.86	1977	5064.86	2142.43	0.2736348	0.56282867	11316.888	6334.516	Down	-0.837170992952853	Isoform 2 of Centromere protein V OS=Mus musculus GN=Cenpv
GDA	Q9R111	9	9	42967.11	44323.44	42949	44188.67	36996.44	40161.56	44336.11	38012.78	41819.89	34087.22	0.273864396	0.562464425	42284.932	39683.512	Down	-0.0916039483587688	Guanine deaminase OS=Mus musculus GN=Gda PE=1 SV=1
NUDCD2	Q9CQ48	6	6	23072	20243.4	11343.2	22242.2	22518.8	22652.2	23423.2	19805.8	25681.8	NA	0.274142736	0.562023257	19883.92	22890.75	Up	0.203162528663381	NudC domain-containing protein 2 OS=Mus musculus GN=Nudcd2 PE=1 SV=1
DNAJB2	Q9QYI5	1	1	41333	48418	44416	20407	24255	19141	28130	21699	39651	NA	0.274163505	0.561990356	35765.8	27155.25	Down	-0.397349566469709	DnaJ homolog subfamily B member 2 OS=Mus musculus GN=Dnajb2 PE=1 SV=2
PODXL2	Q8CAE9	3	3	1079	4318.67	2688.33	6131.33	5308.67	6642	7647.33	4395.33	3342.67	NA	0.274710125	0.561125334	3905.2	5506.8325	Up	0.495826290942086	Podocalyxin-like protein 2 OS=Mus musculus GN=Podxl2 PE=2 SV=2
ADGRL1	Q80TR1-2	13	13	15366.08	26558.85	18958.38	37991.77	29726.23	37201.92	38620.85	20004.69	33989.31	29456.15	0.274878467	0.560859279	25720.262	31854.584	Up	0.308595658130312	Isoform 2 of Latrophilin-1 OS=Mus musculus GN=Lphn1
SEPT6	Q9R1T4-3	14	14	87710.57	93099.57	100492.71	91530.21	90115.07	95096.43	96086.71	104426.07	91935.71	173533.86	0.274888114	0.560844039	92589.626	112215.76	Up	0.277352792148735	Isoform V of Septin-6 OS=Mus musculus GN=Sept6
NME1	P15532	13	13	85817.85	119450.69	76912.08	157994	85930.38	104985.77	103589.62	83228.08	83321.77	36445.77	0.275238405	0.560290968	105221	82314.202	Down	-0.354209395137617	Nucleoside diphosphate kinase A OS=Mus musculus GN=Nme1 PE=1 SV=1
CAMK4	P08414	10	10	19364.5	25084.5	16480	31905.5	27461	28605	34328.75	15454	29325	54757	0.275256094	0.560263057	24059.1	32493.95	Up	0.433588454554301	Calcium/calmodulin-dependent protein kinase type IV OS=Mus musculus GN=Camk4 PE=1 SV=2
HPCA	P84075	4	4	92560.25	106956.75	102003	159490.5	123901.25	122967.75	131235.25	153444	98810.5	218986.75	0.275813969	0.559383742	116982.35	145088.85	Up	0.310645777265119	Neuron-specific calcium-binding protein hippocalcin OS=Mus musculus GN=Hpca PE=1 SV=2
MACROD1	Q922B1	3	3	7204.67	13715	4134	21969.33	15671	18708.33	14463	NA	16823.33	NA	0.276727927	0.55794701	12538.8	16664.887	Up	0.410412221515168	O-acetyl-ADP-ribose deacetylase MACROD1 OS=Mus musculus GN=Macrod1 PE=1 SV=2
SHISA6	Q3UH99	1	1	57567	64064	127981	23784	53268	44798	64109	131108	92317	350754	0.27674256	0.557924046	65332.8	136617.2	Up	1.06425975278565	Protein shisa-6 homolog OS=Mus musculus GN=Shisa6 PE=1 SV=1
HOOK1	Q8BIL5	1	1	12779	15098	32996	11692	21795	13057	20581	50922	37699	NA	0.276944202	0.557607723	18872	30564.75	Up	0.695621443143311	Protein Hook homolog 1 OS=Mus musculus GN=Hook1 PE=1 SV=2
DES	P31001	1	1	72858	73538	47854	72005	65829	67773	55832	NA	44555	NA	0.27713118	0.557314609	66416.8	56053.333	Down	-0.244748046051134	Desmin OS=Mus musculus GN=Des PE=1 SV=3
BORCS5	Q9D920	1	1	72494	71149	76435	56055	67364	67887	79521	217823	122013	47200	0.277175979	0.55724441	68699.4	106888.8	Up	0.637741288661808	Loss of heterozygosity 12 chromosomal region 1 protein homolog OS=Mus musculus GN=Loh12cr1 PE=2 SV=1
BIN2	D3Z6Q9	5	5	336278	450543.75	319360.25	476851.75	489534.5	409675	430959.5	290226.25	413541.25	83926.25	0.277560865	0.556641768	414513.65	325665.65	Down	-0.348028053020926	Bridging integrator 2 OS=Mus musculus GN=Bin2 PE=1 SV=1
PSMD4	O35226-3	9	9	68202.38	73175.12	89657.75	39021.75	69212.5	57589.5	62661.5	105629	76974.62	138634	0.277765643	0.556321474	67853.9	88297.724	Up	0.379944511837783	Isoform Rpn10C of 26S proteasome non-ATPase regulatory subunit 4 OS=Mus musculus GN=Psmd4
CHMP3	Q9CQ10	1	1	67543	78594	109772	28774	66483	39297	57086	153020	99874	293106	0.277803691	0.556261988	70233.2	128476.6	Up	0.871280544474292	Charged multivesicular body protein 3 OS=Mus musculus GN=Chmp3 PE=1 SV=3
ARRB1	Q8BWG8	2	2	12249.5	17251.5	5516	20080	21296.5	19453	19615.5	NA	17765	NA	0.277812892	0.556247604	15278.7	18944.5	Up	0.310257267692679	Beta-arrestin-1 OS=Mus musculus GN=Arrb1 PE=1 SV=1
ACADSB	Q9DBL1	2	2	19287	21693.5	56455	5830.5	22720	19328	23350.5	66039.5	33929.5	55384	0.277888409	0.556129568	25197.2	39606.3	Up	0.652466506345152	Short/branched chain specific acyl-CoA dehydrogenase, mitochondrial OS=Mus musculus GN=Acadsb PE=1 SV=1
IMPACT	O55091	1	1	13493	17504	3900	27066	12124	20719	19002	NA	19013	NA	0.278032736	0.555904066	14817.4	19578	Up	0.4019410722177	Protein IMPACT OS=Mus musculus GN=Impact PE=1 SV=2
AFTPH	Q80WT5	2	2	7863.5	10099	1979	6171.5	9905.5	7044.5	10333.5	NA	11322.5	NA	0.278143632	0.555730879	7203.7	9566.8333	Up	0.40930336383117	Aftiphilin OS=Mus musculus GN=Aftph PE=1 SV=2
PCCB	Q99MN9	3	3	32104	42572.67	15085.33	34036.33	38116.33	45418.33	41812	22447	40157.33	51207	0.278222524	0.555607714	32382.932	40208.332	Up	0.312260873344977	Propionyl-CoA carboxylase beta chain, mitochondrial OS=Mus musculus GN=Pccb PE=1 SV=2
ATP5K	Q06185	7	7	130813.29	113362.71	198874.71	60707.29	130642.57	91634.14	116837.29	280934.14	141805.86	278479.14	0.278233767	0.555590165	126880.11	181938.11	Up	0.5199818307295	ATP synthase subunit e, mitochondrial OS=Mus musculus GN=Atp5i PE=1 SV=2
TBCB	Q9D1E6	6	6	33126.67	35824	49870.17	30282.67	44214.5	34830.67	43279	83714.17	42754.17	45112.83	0.278835324	0.554652209	38663.602	49938.168	Up	0.369166847472104	Tubulin-folding cofactor B OS=Mus musculus GN=Tbcb PE=1 SV=2
MRPL1	Q99N96	1	1	79154	99120	81953	45636	66015	64488	62843	137759	88586	308117	0.278970172	0.55444223	74375.6	132358.6	Up	0.831550630066828	39S ribosomal protein L1, mitochondrial OS=Mus musculus GN=Mrpl1 PE=1 SV=2
PTMS	Q9D0J8	2	2	71421	79826	59122	120266.5	62203	91280	66001	22645.5	82787	23860	0.279201026	0.55408299	78567.7	57314.7	Down	-0.455031120408736	Parathymosin OS=Mus musculus GN=Ptms PE=1 SV=3
NELFE	P19426	1	1	5331	4307	2408	4010	3838	4412	4138	9686	4744	NA	0.279836821	0.553095142	3978.8	5745	Up	0.529973511120453	Negative elongation factor E OS=Mus musculus GN=Nelfe PE=1 SV=2
GNAQ	P21279	6	6	21591.33	30676.83	26094.17	42696.67	29162.67	35454.67	39684	40263	31549.67	28058.67	0.279971852	0.552885629	30044.334	35002.002	Up	0.22034450019669	Guanine nucleotide-binding protein G(q) subunit alpha OS=Mus musculus GN=Gnaq PE=1 SV=4
STIP1	Q60864	26	26	130533.62	120155.31	119276.15	84324.62	120493.5	103333.92	110615.73	142297.38	145455.08	142824.77	0.28010714	0.552675821	114956.64	128905.38	Up	0.165222632725877	Stress-induced-phosphoprotein 1 OS=Mus musculus GN=Stip1 PE=1 SV=1
ALB	P07724	43	43	69916.44	71607.7	65529.07	80393.28	84800.98	74377.05	85373.47	41965.12	70943.65	42398.56	0.280694399	0.551766253	74449.494	63011.57	Down	-0.240645287110609	Serum albumin OS=Mus musculus GN=Alb PE=1 SV=3
KHSRP	Q3U0V1	8	8	45127.12	34114.38	41907.88	20285.88	40990.75	28857.25	30594.88	17211.62	42606.75	28483	0.280745373	0.551687393	36485.202	29550.7	Down	-0.304119135735368	Far upstream element-binding protein 2 OS=Mus musculus GN=Khsrp PE=1 SV=2
RPL7A	P12970	25	25	97789.28	94389	140575.2	77781.44	91070.88	75871.04	96684.72	155079.96	122107.32	177939.28	0.281538635	0.550462	100321.16	125536.46	Up	0.323480543012409	60S ribosomal protein L7a OS=Mus musculus GN=Rpl7a PE=2 SV=2
AIDA	Q8C4Q6	3	3	702	1078	NA	971	912	2124	785	NA	1524	NA	0.281925499	0.549865642	915.75	1477.6667	Up	0.690295160735549	Axin interactor, dorsalization-associated protein OS=Mus musculus GN=Aida PE=1 SV=1
NAPG	Q9CWZ7	54	54	54153.18	51071.92	52936.26	44950.28	53079.28	47911.36	51366.48	55823.02	58119.52	57909.38	0.282265126	0.549342776	51238.184	54225.952	Up	0.0817641321888765	Gamma-soluble NSF attachment protein OS=Mus musculus GN=Napg PE=1 SV=1
VDAC3	Q60931	15	15	91311.33	79805.87	142614.4	89565.87	82807.6	102078.73	90386.87	136302.33	95779.2	181629.73	0.282280903	0.549318502	97221.014	121235.37	Up	0.318470598909032	Voltage-dependent anion-selective channel protein 3 OS=Mus musculus GN=Vdac3 PE=1 SV=1
NUDCD3	Q8R1N4	6	6	17097.8	22365.2	15787.6	17832.8	19543.8	22267	20432.8	9934.4	14612.8	3496.6	0.28258933	0.54884424	18525.44	14148.72	Down	-0.388836266686784	NudC domain-containing protein 3 OS=Mus musculus GN=Nudcd3 PE=2 SV=3
RPL8	P62918	20	20	108546.95	119919.45	159265.15	84631.45	104986.75	97885.9	120651.95	168206.7	145972.05	143703.6	0.283051391	0.548134706	115469.95	135284.04	Up	0.228474196564591	60S ribosomal protein L8 OS=Mus musculus GN=Rpl8 PE=2 SV=2
SIPA1L1	Q8C0T5	22	22	39985.36	45951.73	46187.32	37876.77	59773.86	44879	59154.73	55028.27	62634.91	40703.09	0.283169141	0.547954076	45955.008	52480	Up	0.19154563051844	Signal-induced proliferation-associated 1-like protein 1 OS=Mus musculus GN=Sipa1l1 PE=1 SV=2
TUBA1B	P05213	103	103	159728.85	192175.47	205002.17	214364.88	170443.95	202368.86	208840.53	192570.74	197141.72	205511.05	0.283311658	0.547735555	188343.06	201286.58	Up	0.0958880844077071	Tubulin alpha-1B chain OS=Mus musculus GN=Tuba1b PE=1 SV=2
PTRH2	Q8R2Y8	1	1	57256	72802	166792	16417	72475	56946	66548	256456	109058	151060	0.283352248	0.547673337	77148.4	128013.6	Up	0.730588946625676	Peptidyl-tRNA hydrolase 2, mitochondrial OS=Mus musculus GN=Ptrh2 PE=2 SV=1
PGAM2	O70250	4	4	201193.25	256166.75	87956.25	364929.75	378842	263506	260671.25	92266	213916.75	63092.75	0.283917279	0.546808175	257817.6	178690.55	Down	-0.528887413223417	Phosphoglycerate mutase 2 OS=Mus musculus GN=Pgam2 PE=1 SV=3
CPEB2	Q812E0	3	3	50345.33	48166	110546.67	19013.33	47555.67	38926.33	43983.33	132166.33	68694	152835	0.284531653	0.545869413	55125.4	87320.998	Up	0.663611399954291	Cytoplasmic polyadenylation element-binding protein 2 OS=Mus musculus GN=Cpeb2 PE=1 SV=1
GIPC1	Q9Z0G0	1	1	337517	266443	395846	126438	297179	256557	277640	436643	318921	524191	0.284767448	0.545509656	284684.6	362790.4	Up	0.349771827739074	PDZ domain-containing protein GIPC1 OS=Mus musculus GN=Gipc1 PE=1 SV=1
HDHD2	Q3UGR5-2	4	4	10038.25	7545	5561.75	8093.5	8534.5	10712.25	8021.25	4930.75	11986.5	16487.5	0.28507445	0.545041704	7954.6	10427.65	Up	0.39055277426336	Isoform 2 of Haloacid dehalogenase-like hydrolase domain-containing protein 2 OS=Mus musculus GN=Hdhd2
ZNRF1	Q91V17	1	1	36462	36955	11131	16117	43628	28629	23365	59393	58586	NA	0.285726066	0.544050139	28858.6	42493.25	Up	0.55823237620908	E3 ubiquitin-protein ligase ZNRF1 OS=Mus musculus GN=Znrf1 PE=1 SV=1
TXN2	P97493	3	3	48285.33	45856	80532.67	32530.33	44292.33	41394	44022.33	82239.67	61068.33	114542.67	0.286087782	0.543500689	50299.332	68653.4	Up	0.448791929905831	Thioredoxin, mitochondrial OS=Mus musculus GN=Txn2 PE=1 SV=1
5031439G07RIK	Q3UE31	2	2	1224	2607	1009	4258	3483.5	4110	4402	2252	3009	NA	0.286098329	0.543484678	2516.3	3443.25	Up	0.452466998347974	Uncharacterized protein KIAA0930 homolog OS=Mus musculus PE=1 SV=2
CYB5A	P56395	2	2	10018	11578.5	12574	23434	8228	15120	11441.5	2852	5310	NA	0.286118216	0.543454491	13166.5	8680.875	Down	-0.600959517070033	Cytochrome b5 OS=Mus musculus GN=Cyb5a PE=1 SV=2
MAP1LC3A	Q91VR7	2	2	39116	49209	72876.5	119309.5	41120.5	69904	54273	19544.5	24862.5	NA	0.286260935	0.543237915	64326.3	42146	Down	-0.610012994237012	Microtubule-associated proteins 1A/1B light chain 3A OS=Mus musculus GN=Map1lc3a PE=1 SV=1
BCL11A	Q9QYE3-13	2	2	24177	15499	19684.5	14666.5	29823.5	25696	20410.5	NA	32177.5	NA	0.286805292	0.542412839	20770.1	26094.667	Up	0.32924681086826	Isoform 5 of B-cell lymphoma/leukemia 11A OS=Mus musculus GN=Bcl11a
SYNCRIP	Q7TMK9	19	19	76194.58	86672.11	70397.95	73502.58	73706.74	66741.47	76019.89	72201.84	79729.95	34301.53	0.287072294	0.54200872	76094.792	65798.936	Down	-0.209733462599528	Heterogeneous nuclear ribonucleoprotein Q OS=Mus musculus GN=Syncrip PE=1 SV=2
EPN2	Q8CHU3	6	6	22375.5	23144.33	30150.17	15506.33	22181.17	20029.33	20750.17	26368.33	33434.67	35046.67	0.287366266	0.541564216	22671.5	27125.834	Up	0.25878764841275	Epsin-2 OS=Mus musculus GN=Epn2 PE=1 SV=1
CLYBL	Q8R4N0	7	7	76174.29	74131.71	66712.43	44418.57	69080.14	63016.57	71363	80004.29	82381.43	72135.14	0.287987055	0.540627033	66103.428	73780.086	Up	0.158506381540465	Citrate lyase subunit beta-like protein, mitochondrial OS=Mus musculus GN=Clybl PE=1 SV=2
NDUFV2	Q9D6J6	10	10	39660.2	43157.8	44022.6	54631.3	43217.2	46808.5	41191.2	65193.5	49359.3	49397.7	0.28803769	0.540550681	44937.82	50390.04	Up	0.165208462581752	NADH dehydrogenase [ubiquinone] flavoprotein 2, mitochondrial OS=Mus musculus GN=Ndufv2 PE=1 SV=2
NCAN	P55066	25	25	58443.48	61768.08	45373.92	56504.68	57467.2	55371.72	59275.16	23709.36	56086.44	43333.36	0.289370453	0.538545816	55911.472	47555.208	Down	-0.233540981557103	Neurocan core protein OS=Mus musculus GN=Ncan PE=2 SV=1
LMCD1	Q8VEE1	1	1	5926	6647	4793	8905	8104	9686	6188	NA	12334	NA	0.289745115	0.537983877	6875	9402.6667	Up	0.451710260412279	LIM and cysteine-rich domains protein 1 OS=Mus musculus GN=Lmcd1 PE=1 SV=1
ATP5H	Q9DCX2	38	38	201926.61	186207.5	254378.82	110604.89	179211.24	153941.39	160820.16	327995.76	204152.05	402423.42	0.290735998	0.536501192	186465.81	249866.56	Up	0.422246672791405	ATP synthase subunit d, mitochondrial OS=Mus musculus GN=Atp5h PE=1 SV=3
PRKCZ	Q02956	1	1	726	833	NA	3474	1945	2311	2220	4194	2042	NA	0.290865944	0.536307124	1744.5	2691.75	Up	0.625730826342859	Protein kinase C zeta type OS=Mus musculus GN=Prkcz PE=1 SV=2
MYO18A	Q9JMH9-6	41	41	9006.05	14168.12	11662.9	23305.8	16454.05	21204.25	22943.6	15466.02	18431.12	13584.92	0.291282694	0.535685317	14919.384	18325.982	Up	0.296702537411055	Isoform 6 of Unconventional myosin-XVIIIa OS=Mus musculus GN=Myo18a
SCN3B	Q8BHK2	1	1	10263	32051	34922	10890	31018	33140	50206	20915	22737	34826	0.291519495	0.535332397	23828.8	32364.8	Up	0.441719288343159	Sodium channel subunit beta-3 OS=Mus musculus GN=Scn3b PE=2 SV=1
PHF5A	P83870	1	1	9823	6158	14052	3615	5757	7851	8636	10706	20426	NA	0.291965323	0.534668727	7881	11904.75	Up	0.595086717994864	PHD finger-like domain-containing protein 5A OS=Mus musculus GN=Phf5a PE=1 SV=1
SCO1	Q5SUC9	1	1	29564	30672	69091	26689	30029	31209	32111	88151	31536	155751	0.292071361	0.534511025	37209	67751.6	Up	0.86460339885056	Protein SCO1 homolog, mitochondrial OS=Mus musculus GN=Sco1 PE=2 SV=1
HSPA9	P38647	92	92	63821.47	69483.18	79931.6	44924.61	62171.43	55443.43	59108.07	99318.13	73345.43	92815.39	0.292351328	0.534094929	64066.458	76006.09	Up	0.246545787978273	Stress-70 protein, mitochondrial OS=Mus musculus GN=Hspa9 PE=1 SV=3
GNAO1	P18872	34	34	52028.88	89510.71	84121.91	146709.62	90757.5	123448.35	140456.5	94571.21	82069.38	129108.71	0.293240649	0.532775828	92625.724	113930.83	Up	0.298673377912919	Guanine nucleotide-binding protein G(o) subunit alpha OS=Mus musculus GN=Gnao1 PE=1 SV=3
NDUFS3	Q9DCT2	4	4	12626	12892.33	3473.67	27898.33	14225	26132.33	16919.33	13658	22000	NA	0.293464471	0.53244447	14223.066	19677.415	Up	0.468308214367877	NADH dehydrogenase [ubiquinone] iron-sulfur protein 3, mitochondrial OS=Mus musculus GN=Ndufs3 PE=1 SV=2
MIA3	Q8BI84-3	2	2	2024.5	7702	1771	23187.5	9054.5	13545	12712	NA	14099	NA	0.294716581	0.530595429	8747.9	13452	Up	0.620812050285609	Isoform 3 of Melanoma inhibitory activity protein 3 OS=Mus musculus GN=Mia3
PFDN6	Q03958	5	5	48742.6	52041.4	59901.4	24884	45460.6	32759	44116.8	77848.8	53025	123855.4	0.294876681	0.530359571	46206	66321	Up	0.521385557745422	Prefoldin subunit 6 OS=Mus musculus GN=Pfdn6 PE=2 SV=1
ALKBH7	Q9D6Z0	1	1	30724	26286	61275	19409	26783	34794	34048	NA	43296	79986	0.295529697	0.529398872	32895.4	48031	Up	0.546079989978608	Alpha-ketoglutarate-dependent dioxygenase alkB homolog 7, mitochondrial OS=Mus musculus GN=Alkbh7 PE=2 SV=1
PSMC5	P62196	15	15	32684.64	33250.93	35113.86	38318.07	31787.14	38854.43	39879	43489.43	47049.93	24954.21	0.296371926	0.528162938	34230.928	38845.4	Up	0.182443363163619	26S protease regulatory subunit 8 OS=Mus musculus GN=Psmc5 PE=1 SV=1
HSP90AB1	P11499	44	44	32817.26	42334.71	30174.55	51104.42	37603.24	45244.82	38405.05	23989.89	33275.39	19844.58	0.296478573	0.528006688	38806.836	32151.946	Down	-0.271404752598419	Heat shock protein HSP 90-beta OS=Mus musculus GN=Hsp90ab1 PE=1 SV=3
DENR	Q9CQJ6	2	2	46333.5	48386.5	42490.5	62731	39946	41491	46991.5	11269.5	42149.5	51688.5	0.296797012	0.527540476	47977.5	38718	Down	-0.309353552668694	Density-regulated protein OS=Mus musculus GN=Denr PE=2 SV=1
2410015M20RIK	Q8R404	2	2	28572	32365	38303.5	26763	31024.5	27633	28696.5	49151.5	31297.5	75885	0.297728231	0.526179983	31405.6	42532.7	Up	0.437550608865958	Protein QIL1 OS=Mus musculus GN=Qil1 PE=2 SV=1
MLEC	Q6ZQI3	1	1	812	3750	1195	6359	2033	5468	6152	3517	2259	NA	0.298589498	0.524925472	2829.8	4349	Up	0.619983616594429	Malectin OS=Mus musculus GN=Mlec PE=2 SV=2
UCHL1	Q9R0P9	39	39	66975.58	77291.71	115473.82	64496.34	68489.95	60470.58	71487.39	179458.03	82768.5	140234	0.299345349	0.523827485	78545.48	106883.7	Up	0.444441694365749	Ubiquitin carboxyl-terminal hydrolase isozyme L1 OS=Mus musculus GN=Uchl1 PE=1 SV=1
UQCRC2	Q9DB77	43	43	80809.12	80386.4	118323.69	52753.64	75844.9	75952.17	75862.52	124061.33	82440.95	165701.93	0.299624962	0.523422008	81623.55	104803.78	Up	0.360633388804116	Cytochrome b-c1 complex subunit 2, mitochondrial OS=Mus musculus GN=Uqcrc2 PE=1 SV=1
GPR158	Q8C419	28	28	36139.17	49868.67	32904.62	90514.88	64590.46	84544.62	83839.38	60046.58	70725.21	46455.83	0.29970703	0.52330307	54803.56	69122.324	Up	0.33488211130089	Probable G-protein coupled receptor 158 OS=Mus musculus GN=Gpr158 PE=1 SV=2
SCAMP1	Q8K021	2	2	8115.5	14381	9085	18501.5	9393	14519	12439.5	NA	16638	NA	0.299719064	0.523285632	11895.2	14532.167	Up	0.288870288230365	Secretory carrier-associated membrane protein 1 OS=Mus musculus GN=Scamp1 PE=1 SV=1
ZDHHC5	Q8VDZ4	2	2	12940	9969.5	22583	7457	15053.5	11269	16654.5	20577	21491.5	NA	0.299904993	0.523016304	13600.6	17498	Up	0.363519734819683	Palmitoyltransferase ZDHHC5 OS=Mus musculus GN=Zdhhc5 PE=1 SV=1
PSMA8	Q9CWH6	4	4	29885.75	32892.5	32739.5	34872.5	34969	36679.75	31006	65266.5	37752	NA	0.300069638	0.522777946	33071.85	42676.063	Up	0.367823321845183	Proteasome subunit alpha type-7-like OS=Mus musculus GN=Psma8 PE=2 SV=1
GAS7	Q60780	14	14	64364.54	60831.46	55817.92	39061.38	59127.38	58161	64562.46	58568.62	82155.46	52992.54	0.300252196	0.522513807	55840.536	63288.016	Up	0.180619552067642	Growth arrest-specific protein 7 OS=Mus musculus GN=Gas7 PE=1 SV=1
C77080	A2A7S8-3	2	2	24693	22184	NA	14628	24521	23803	23472	NA	40328	NA	0.30075813	0.521782624	21506.5	29201	Up	0.441245018110385	Isoform 3 of Uncharacterized protein KIAA1522 OS=Mus musculus GN=Kiaa1522
NPEPPS	Q11011	4	4	8587.5	34058.75	8860.5	68348.25	44186.25	51563.25	67163.5	NA	31755.25	NA	0.301250828	0.521071751	32808.25	50160.667	Up	0.612497880381994	Puromycin-sensitive aminopeptidase OS=Mus musculus GN=Npepps PE=1 SV=2
CNPY2	Q9QXT0	10	10	19066.75	18645.62	13707	13383.75	15065.12	17342.75	16403.88	16532.12	20543.5	NA	0.301346086	0.520934445	15973.648	17705.563	Up	0.148508850916861	Protein canopy homolog 2 OS=Mus musculus GN=Cnpy2 PE=1 SV=1
SNX12	O70493	10	10	83344.4	84797.3	97906.9	45842.7	77719.5	73210.5	70226.8	118882.2	89465.6	109532.8	0.301599829	0.520568909	77922.16	92263.58	Up	0.243727603324923	Sorting nexin-12 OS=Mus musculus GN=Snx12 PE=1 SV=1
HIST1H3B	P84228	18	18	348901.11	152236.61	151441.28	29383.89	159839.78	89571.33	98924.61	127797.06	127877.78	92197.44	0.301699292	0.520425709	168360.53	107273.64	Down	-0.650258325974092	Histone H3.2 OS=Mus musculus GN=Hist1h3b PE=1 SV=2
APIP	Q9WVQ5	1	1	13094	8280	12862	6434	7720	9342	9762	8984	15083	17635	0.301770002	0.520323934	9678	12161.2	Up	0.329504748650856	Methylthioribulose-1-phosphate dehydratase OS=Mus musculus GN=Apip PE=1 SV=1
CNRIP1	Q5M8N0	4	4	130543.75	125892	77238.5	147503.5	109298	132115.75	124735.25	152599.5	143441.75	114347.25	0.30211398	0.519829178	118095.15	133447.9	Up	0.176326886202727	CB1 cannabinoid receptor-interacting protein 1 OS=Mus musculus GN=Cnrip1 PE=1 SV=1
SGTB	Q8VD33	2	2	243850.5	313081	395315	206171	318750.5	255851	272345	611867.5	234937.5	739086	0.302474139	0.519311751	295433.6	422817.4	Up	0.517200833688675	Small glutamine-rich tetratricopeptide repeat-containing protein beta OS=Mus musculus GN=Sgtb PE=2 SV=1
COMMD3	Q63829	1	1	36179	34341	15032	31847	34859	36658	27910	NA	34116	56113	0.30336305	0.518037318	30451.6	38699.25	Up	0.345787574116525	COMM domain-containing protein 3 OS=Mus musculus GN=Commd3 PE=2 SV=1
CSTF2T	Q8C7E9	2	2	8811	8987	NA	5879	8183	8734	1976	NA	5172	NA	0.303387186	0.518002767	7965	5294	Down	-0.589316166917274	Cleavage stimulation factor subunit 2 tau variant OS=Mus musculus GN=Cstf2t PE=1 SV=2
DIRAS1	Q91Z61	1	1	25354	41088	47074	25499	28536	37390	27155	77305	47326	NA	0.303771323	0.517453227	33510.2	47294	Up	0.497056869192028	GTP-binding protein Di-Ras1 OS=Mus musculus GN=Diras1 PE=2 SV=1
MRPL42	Q9CPV3	2	2	13297.5	14159	14973.5	9192	14537.5	13235	14482	14175	24677	NA	0.304447708	0.516487291	13231.9	16642.25	Up	0.330830259739069	39S ribosomal protein L42, mitochondrial OS=Mus musculus GN=Mrpl42 PE=2 SV=1
PSMD3	P14685	1	1	1482	1850	NA	4980	3349	4840	4021	NA	3127	NA	0.304763928	0.516036438	2915.25	3996	Up	0.454936974732065	26S proteasome non-ATPase regulatory subunit 3 OS=Mus musculus GN=Psmd3 PE=1 SV=3

HEXB	P20060	3	3	106172	117980.67	215245	44987	118586.67	81570	120249.33	222077.67	139272.67	321513.67	0.304896901	0.515846989	120594.27	176936.67	Up	0.553071724513147	Beta-hexosaminidase subunit beta OS=Mus musculus GN=Hexb PE=2 SV=2
MTO1	Q923Z3	1	1	1414	4081	NA	6748	5062	6012	5119	NA	6018	NA	0.305558262	0.514905969	4326.25	5716.3333	Up	0.401973009561116	Protein MTO1 homolog, mitochondrial OS=Mus musculus GN=Mto1 PE=2 SV=1
GPRC5B	Q923Z0	2	2	778	6168	1112.5	8920	8493.5	12497	10439.5	NA	3681.5	NA	0.305673961	0.514741555	5094.4	8872.6667	Up	0.800455528536048	G-protein coupled receptor family C group 5 member B OS=Mus musculus GN=Gprc5b PE=2 SV=1
UBXN4	Q8VCH8	2	2	1138	NA	3537	1644	2167	3070	3091	9817	1650	NA	0.30583976	0.514506055	2121.5	4407	Up	1.0547122179867	UBX domain-containing protein 4 OS=Mus musculus GN=Ubxn4 PE=1 SV=1
EIF3H	Q91WK2	1	1	49365	52208	87442	41656	34980	40658	44034	106561	59245	119479	0.306028584	0.514238007	53130.2	73995.4	Up	0.477903443298872	Eukaryotic translation initiation factor 3 subunit H OS=Mus musculus GN=Eif3h PE=1 SV=1
BIN1	O08539-2	33	33	101709.55	112340.58	85312	146574.18	124593.88	117794.82	111684.42	79034.61	118563.79	50504.73	0.306231928	0.513949531	114106.04	95516.474	Down	-0.256553649383917	Isoform 2 of Myc box-dependent-interacting protein 1 OS=Mus musculus GN=Bin1
PPIH	Q9D868-2	1	1	60324	73339	109065	22136	47556	37181	53109	118100	86392	181652	0.306264806	0.513902907	62484	95286.8	Up	0.608789560088224	Isoform 2 of Peptidyl-prolyl cis-trans isomerase H OS=Mus musculus GN=Ppih
ANXA6	P14824	6	6	24478	30186	26321	32393.67	32293	34810.83	33841.5	11351.17	22025.67	14731.83	0.306391127	0.513723816	29134.334	23352.2	Down	-0.31916185961953	Annexin A6 OS=Mus musculus GN=Anxa6 PE=1 SV=3
NAMPT	Q99KQ4	1	1	761	1050	3112	2504	2015	2897	2471	NA	NA	9343	0.30648658	0.513588536	1888.4	4903.6667	Up	1.37669652517247	Nicotinamide phosphoribosyltransferase OS=Mus musculus GN=Nampt PE=1 SV=1
HIST1H3A	P68433	10	10	659761.11	287949.33	279286.22	51264.22	302791.44	169771.89	192613.78	243768.78	251426.78	146081.78	0.306537764	0.513516015	316210.46	200732.6	Down	-0.655610158833535	Histone H3.1 OS=Mus musculus GN=Hist1h3a PE=1 SV=2
RBMX	Q9WV02	4	4	62616	39007.25	26592	55841	41704.75	46605.5	30757.5	10702.5	45474.75	NA	0.306638316	0.513373579	45152.2	33385.063	Down	-0.435593542331073	RNA-binding motif protein, X chromosome OS=Mus musculus GN=Rbmx PE=1 SV=1
ADSS	P46664	4	4	64427.75	61802	62098.75	40255	48710.25	52486.75	57909.5	91770	60531.25	60702.75	0.307131637	0.512675446	55458.75	64680.05	Up	0.221905694623021	Adenylosuccinate synthetase isozyme 2 OS=Mus musculus GN=Adss PE=1 SV=2
NFX1	B1AY10-3	2	2	499	NA	NA	2604	1353	2778.5	1298.5	NA	3513	NA	0.307330448	0.512394411	1485.3333	2530	Up	0.768350652962797	Isoform 3 of Transcriptional repressor NF-X1 OS=Mus musculus GN=Nfx1
GNAO1	P18872-2	31	31	54825.39	95437.45	87938.74	158243.03	96891.35	132026	149764.16	99134.94	87017.48	138425.81	0.307485375	0.512175535	98667.192	121273.68	Up	0.297624096362803	Isoform Alpha-2 of Guanine nucleotide-binding protein G(o) subunit alpha OS=Mus musculus GN=Gnao1
ECI2	Q9WUR2	6	6	13719	19643.17	29779.17	13947.33	23853.67	19261.5	21214.17	22074	24504.17	42091.33	0.308345048	0.510963021	20188.468	25829.034	Up	0.355462251953048	Enoyl-CoA delta isomerase 2, mitochondrial OS=Mus musculus GN=Eci2 PE=1 SV=2
INPPL1	Q6P549	1	1	8095	10066	8004	14093	13860	11674	18022	NA	11663	NA	0.308694631	0.510470924	10823.6	13786.333	Up	0.349058374332527	Phosphatidylinositol 3,4,5-trisphosphate 5-phosphatase 2 OS=Mus musculus GN=Inppl1 PE=1 SV=1
ALDH4A1	Q8CHT0	5	5	34713.4	34237	29747.8	28530.4	28394	31552.2	31166	23693.2	33077.4	3187.2	0.309138025	0.509847573	31124.52	24535.2	Down	-0.343198556558017	Delta-1-pyrroline-5-carboxylate dehydrogenase, mitochondrial OS=Mus musculus GN=Aldh4a1 PE=1 SV=3
TAOK3	Q8BYC6	2	2	1219	1408.5	692	3597	3529.5	3632	2944	3084.5	1864	NA	0.309141796	0.509842274	2089.2	2881.125	Up	0.463681645554886	Serine/threonine-protein kinase TAO3 OS=Mus musculus GN=Taok3 PE=1 SV=2
CBX1	P83917	1	1	55378	48157	31222	98843	54781	50380	65565	28464	18843	NA	0.309444436	0.509417322	57676.2	40813	Down	-0.498947354435907	Chromobox protein homolog 1 OS=Mus musculus GN=Cbx1 PE=1 SV=1
2900026A02RIK	Q8BRV5	2	2	22598.5	26520.5	15970	34481.5	28045.5	31613.5	25397	NA	31989.5	NA	0.30959996	0.509199104	25523.2	29666.667	Up	0.217033615396495	Uncharacterized protein KIAA1671 OS=Mus musculus GN=Kiaa1671 PE=2 SV=1
OTUB1	Q7TQI3	4	4	26531	41870.67	20813.33	43037	44128	52265.67	67373.33	25915	41039.67	NA	0.309844094	0.508856777	35276	46648.418	Up	0.403141159193214	Ubiquitin thioesterase OTUB1 OS=Mus musculus GN=Otub1 PE=1 SV=2
MRPS11	Q9DCA2	1	1	88886	105014	93565	114821	98041	104751	107614	177216	121992	82763	0.310235726	0.508308192	100065.4	118867.2	Up	0.248407461180825	28S ribosomal protein S11, mitochondrial OS=Mus musculus GN=Mrps11 PE=2 SV=2
ENSA	P60840-3	1	1	61906	51211	58753	52210	90383	76087	96100	97429	45477	NA	0.310549216	0.507869563	62892.6	78773.25	Up	0.324815520880137	Isoform 3 of Alpha-endosulfine OS=Mus musculus GN=Ensa
SOD2	P09671	5	5	94099	100628	155284	45802.8	86157.4	72473.8	76382.4	213039.8	93576	295497.6	0.310558526	0.507856543	96394.24	150193.92	Up	0.639807565829846	Superoxide dismutase [Mn], mitochondrial OS=Mus musculus GN=Sod2 PE=1 SV=3
AK2	Q9WTP6	15	15	68971.27	72476.8	68946.87	51808.2	70227.67	65377.73	72052	77451.07	76919.47	65498.67	0.310710177	0.507644522	66486.162	71459.788	Up	0.104077535243614	Adenylate kinase 2, mitochondrial OS=Mus musculus GN=Ak2 PE=1 SV=5
EPN2	Q8CHU3-2	5	5	26155	27773.2	36180.2	17834	25993.8	22990	24107.6	31642	39464.8	42056	0.311809659	0.506110436	26787.24	32052.08	Up	0.258872040680961	Isoform 2 of Epsin-2 OS=Mus musculus GN=Epn2
ITPA	Q9D892	9	9	80582.33	96852.22	158997.22	56368.78	101656.44	84281.11	97222.89	144380.22	117025.44	185019.11	0.312068352	0.505750272	98891.398	125585.75	Up	0.344755879609077	Inosine triphosphate pyrophosphatase OS=Mus musculus GN=Itpa PE=1 SV=2
CHMP2A	Q9DB34	8	8	29355	24378.38	31457.25	28874	25669.75	27104.5	25819.5	36753.25	27680.88	62393.75	0.313290995	0.504052088	27946.876	35950.376	Up	0.363319837130594	Charged multivesicular body protein 2a OS=Mus musculus GN=Chmp2a PE=1 SV=1
PPP1R2	Q9DCL8	6	6	54719.17	48450.33	73370.33	31113.67	53552.67	43286.83	44509.83	76077	67091.83	94263.83	0.31347299	0.503799874	52241.234	65045.864	Up	0.316268349335168	Protein phosphatase inhibitor 2 OS=Mus musculus GN=Ppp1r2 PE=1 SV=3
UQCRFS1	Q9CR68	17	17	90934.38	98161.94	108445	71029.56	79348.06	83428.31	76762.56	135462.5	92070.56	149094.06	0.313505601	0.503754695	89583.788	107363.6	Up	0.261195349332087	Cytochrome b-c1 complex subunit Rieske, mitochondrial OS=Mus musculus GN=Uqcrfs1 PE=1 SV=1
TCEAL3	Q8R0A5	1	1	17961	20449	9782	27014	19216	19668	18797	NA	26281	68867	0.313858373	0.503266281	18884.4	33403.25	Up	0.822793531042877	Transcription elongation factor A protein-like 3 OS=Mus musculus GN=Tceal3 PE=2 SV=2
CEP170	Q6A065-2	3	3	36907.67	40939	29709.33	39798	36071.33	38363.33	40978	36926.33	62048	34801.33	0.314063349	0.502982743	36685.066	42623.398	Up	0.216452730765921	Isoform 2 of Centrosomal protein of 170 kDa OS=Mus musculus GN=Cep170
LONP1	Q8CGK3	4	4	16240.5	14913.75	11488.25	16860.5	20436	17112	18090.75	9427.25	14659	5125	0.31452824	0.502340355	15987.8	12882.8	Down	-0.311525241810651	Lon protease homolog, mitochondrial OS=Mus musculus GN=Lonp1 PE=1 SV=2
TUBA1C	P68373	83	83	182336.02	222106.54	231350.37	251751.32	195958.54	234726.54	241482.74	222339.12	229147.77	228315.16	0.314538383	0.50232635	216700.56	231202.27	Up	0.0934526693284173	Tubulin alpha-1C chain OS=Mus musculus GN=Tuba1c PE=1 SV=1
CDKN1B	P46414	2	2	13076	13324.5	18253	12984	14827.5	16880	16948.5	13093.5	17084.5	NA	0.314703715	0.50209813	14493	16001.625	Up	0.142862162783791	Cyclin-dependent kinase inhibitor 1B OS=Mus musculus GN=Cdkn1b PE=1 SV=2
DOK4	Q99KE3	1	1	2795	2989	1788	2892	1780	3599	2192	NA	6102	NA	0.314774145	0.502000947	2448.8	3964.3333	Up	0.695003323881039	Docking protein 4 OS=Mus musculus GN=Dok4 PE=1 SV=1
RCN2	Q8BP92	25	25	17913.68	20390.84	19223.48	25088.4	18480.52	20850.16	19794	16329.44	20776.88	11228.12	0.314809943	0.50195156	20219.384	17795.72	Down	-0.18420874076358	Reticulocalbin-2 OS=Mus musculus GN=Rcn2 PE=2 SV=1
RGS8	Q8BXT1	1	1	11172	10349	3964	15537	12451	14912	12767	NA	11478	NA	0.316777754	0.499245325	10694.6	13052.333	Up	0.287425213118151	Regulator of G-protein signaling 8 OS=Mus musculus GN=Rgs8 PE=2 SV=1
CACNA1C	Q01815-3	2	2	870	1062	NA	3222	2100	3043	2387	NA	2082	NA	0.31680338	0.499210193	1813.5	2504	Up	0.465457816962339	Isoform 3 of Voltage-dependent L-type calcium channel subunit alpha-1C OS=Mus musculus GN=Cacna1c
MTIF2	Q91YJ5	2	2	2197.5	2527.5	NA	4331.5	2310.5	2987	2000.5	NA	513	NA	0.31698185	0.498965604	2841.75	1833.5	Down	-0.632179374518199	Translation initiation factor IF-2, mitochondrial OS=Mus musculus GN=Mtif2 PE=1 SV=2
UNKL	Q5FWH2	1	1	24453	29752	NA	24095	38085	30385	26273	31622	34497	63264	0.317276894	0.498561554	29096.25	37208.2	Up	0.354787372867459	Putative E3 ubiquitin-protein ligase UNKL OS=Mus musculus GN=Unkl PE=2 SV=2
PRDX3	P20108	3	3	37521	30532.33	27709.33	60686.33	44346	40326.67	40921.33	24615	20976	NA	0.317875126	0.497743455	40158.998	31709.75	Down	-0.340796770047143	Thioredoxin-dependent peroxide reductase, mitochondrial OS=Mus musculus GN=Prdx3 PE=1 SV=1
PRRT1	O35449	1	1	17450	23028	37630	8770	12624	10404	10770	73674	28618	47720	0.318041988	0.497515541	19900.4	34237.2	Up	0.782767290143975	Proline-rich transmembrane protein 1 OS=Mus musculus GN=Prrt1 PE=1 SV=1
DSTN	Q9R0P5	18	18	167083.83	154154.89	177227.5	151044.78	140154.67	137921.72	174176.72	129882.83	167338.83	93339.94	0.318078321	0.497465929	157933.13	140532.01	Down	-0.168415116470673	Destrin OS=Mus musculus GN=Dstn PE=1 SV=3
PPP3R1	Q63810	23	23	46783.39	48865.3	38542.13	68013.96	33348.7	49290.52	47379.52	35693.91	41741.04	17765.83	0.318110964	0.497421362	47110.696	38374.164	Down	-0.295919325123255	Calcineurin subunit B type 1 OS=Mus musculus GN=Ppp3r1 PE=1 SV=3
EIF5A	P63242	6	6	217007.67	190258	335677	185568.67	167044.33	176618.17	197412.5	400597	211455	500578.17	0.318120304	0.497408611	219111.13	297332.17	Up	0.44041275921554	Eukaryotic translation initiation factor 5A-1 OS=Mus musculus GN=Eif5a PE=1 SV=2
RPS27L	Q6ZWY3	2	2	55453.5	48192	17618	57953	57730.5	50305	53631.5	NA	89890.5	NA	0.31813889	0.497383239	47389.4	64609	Up	0.447170750545672	40S ribosomal protein S27-like OS=Mus musculus GN=Rps27l PE=2 SV=3
SHD	O88834	1	1	1211	1208	NA	2339	1963	4087	2307	NA	1586	NA	0.318147567	0.497371393	1680.25	2660	Up	0.662750341932525	SH2 domain-containing adapter protein D OS=Mus musculus GN=Shd PE=1 SV=1
ERP29	P57759	20	20	57345.37	61286.16	54771.16	43005.63	51409.05	47642.47	59493.16	64421.05	54648.21	65843.58	0.318149887	0.497368226	53563.474	58409.694	Up	0.124958293248531	Endoplasmic reticulum resident protein 29 OS=Mus musculus GN=Erp29 PE=1 SV=2
H3F3A	P84244	17	17	398563.12	171711	171045.25	34588.12	184265.38	102388.69	114486.69	155366.88	148472.69	104188.06	0.318991408	0.496221014	192034.57	124980.6	Down	-0.619661883535718	Histone H3.3 OS=Mus musculus GN=H3f3a PE=1 SV=2
DAGLA	Q6WQJ1	4	4	7192.5	14219	7362.5	21246.5	16618.25	25852.25	22204.5	15131.75	13851.5	10446.75	0.319159488	0.495992241	13327.75	17497.35	Up	0.392703196060211	Sn1-specific diacylglycerol lipase alpha OS=Mus musculus GN=Dagla PE=1 SV=2
DEF8	Q99J78	1	1	4303	3314	NA	2848	3939	3602	4160	NA	4351	NA	0.319223036	0.495905776	3601	4037.6667	Up	0.165124213024075	Differentially expressed in FDCP 8 OS=Mus musculus GN=Def8 PE=2 SV=1
SKP1A	Q9WTX5	8	8	38753.38	40073.88	34583	56198	34906.62	36385.38	40151.12	19247.75	34937.12	43294.25	0.319482856	0.495552442	40902.976	34803.124	Down	-0.232989002770117	S-phase kinase-associated protein 1 OS=Mus musculus GN=Skp1 PE=1 SV=3
ABAT	P61922-2	34	34	45596.85	49016.65	51465.09	55720	44144.91	55848.18	46159.68	66917.38	42540.53	62164.59	0.3195244	0.495495973	49188.7	54726.072	Up	0.153901383054072	Isoform 2 of 4-aminobutyrate aminotransferase, mitochondrial OS=Mus musculus GN=Abat
ATOX1	O08997	7	7	133346.71	138280.29	207377.71	184790.43	109372.86	145682	137498.14	214665.14	178797.71	235254	0.319680906	0.495283302	154633.6	182379.4	Up	0.238088935121304	Copper transport protein ATOX1 OS=Mus musculus GN=Atox1 PE=1 SV=1
NEDD4L	Q8CFI0-3	5	5	17542.4	24488.2	30432	15906.6	40467.6	26335.6	39053.6	40430.8	31045.8	22351.6	0.319773793	0.495157131	25767.36	31843.48	Up	0.305453275596993	Isoform 3 of E3 ubiquitin-protein ligase NEDD4-like OS=Mus musculus GN=Nedd4l
NDUFS5	Q99LY9	15	15	104379.64	88967.5	119230.86	50332.57	99551.07	76831.71	79561.93	180151.57	118688.07	126042.57	0.319967495	0.494894138	92492.328	116255.17	Up	0.329889268068642	NADH dehydrogenase [ubiquinone] iron-sulfur protein 5 OS=Mus musculus GN=Ndufs5 PE=1 SV=3
GSPT1	Q8R050-2	1	1	27779	31793	44186	71053	26480	37637	35176	22025	26473	NA	0.320220428	0.494550967	40258.2	30327.75	Down	-0.408644199666079	Isoform 2 of Eukaryotic peptide chain release factor GTP-binding subunit ERF3A OS=Mus musculus GN=Gspt1
DDT	O35215	1	1	9093	4468	NA	20293	8475	15199	13615	NA	15066	NA	0.320440344	0.49425281	10582.25	14626.667	Up	0.466954618860243	D-dopachrome decarboxylase OS=Mus musculus GN=Ddt PE=1 SV=3
JPH4	Q80WT0	2	2	22146	30837	21240.5	16100	36266	28976.5	34855.5	17019	51798.5	29825	0.320899437	0.493631045	25317.9	32494.9	Up	0.360055563229257	Junctophilin-4 OS=Mus musculus GN=Jph4 PE=2 SV=1
EIF5A2	Q8BGY2	5	5	259851.4	227023.2	402273.8	221430.2	199384.8	210713.4	235810.4	478803.6	251412	600693.8	0.32105202	0.493424593	261992.68	355486.64	Up	0.440268837246196	Eukaryotic translation initiation factor 5A-2 OS=Mus musculus GN=Eif5a2 PE=2 SV=3
DTNA	Q9D2N4-3	2	2	27069	62960.5	88387	49642	65449	86139.5	86225.5	61506	116482	34059.5	0.321151201	0.49329045	58701.5	76882.5	Up	0.389257880202749	Isoform 3 of Dystrobrevin alpha OS=Mus musculus GN=Dtna
SNX6	Q6P8X1	3	3	52641.67	54089.33	23752.33	70852.33	59999	46873.67	53945.33	18361	56949.33	27697	0.321347764	0.493024718	52266.932	40765.266	Down	-0.358558048618362	Sorting nexin-6 OS=Mus musculus GN=Snx6 PE=1 SV=2
UBE2V1	Q9CZY3	21	21	82049.37	83946.21	48981.42	71344.68	82604.89	73519.11	86242.11	33020.63	76267.16	21818.32	0.321636332	0.4926349	73785.314	58173.466	Down	-0.342972432198612	Ubiquitin-conjugating enzyme E2 variant 1 OS=Mus musculus GN=Ube2v1 PE=1 SV=1
GM2A	Q60648	1	1	3124	7297	5314	8978	5120	9525	5942	NA	7200	NA	0.321812266	0.492397407	5966.6	7555.6667	Up	0.34064999368755	Ganglioside GM2 activator OS=Mus musculus GN=Gm2a PE=1 SV=2
VPS26B	Q8C0E2	2	2	6464.5	10934.5	8704	21112.5	10979.5	15325.5	19005	NA	11419	NA	0.32182324	0.492382597	11639	15249.833	Up	0.38982636538149	Vacuolar protein sorting-associated protein 26B OS=Mus musculus GN=Vps26b PE=1 SV=1
GPX1	P11352	7	7	45405.67	41386.67	85402	13986.67	40039.17	31908.67	36019.33	126508.67	56858.83	87379.67	0.321899588	0.492279579	45244.036	67735.034	Up	0.582174588531554	Glutathione peroxidase 1 OS=Mus musculus GN=Gpx1 PE=1 SV=2
ARHGAP44	Q5SSM3-3	2	2	70295	92157	100096.5	82647.5	73624.5	78728	86247.5	26530	95075	59549	0.322131119	0.491967318	83764.1	69225.9	Down	-0.275020154498135	Isoform 3 of Rho GTPase-activating protein 44 OS=Mus musculus GN=Arhgap44
MAP4K4	P97820	1	1	73635	110221	49527	103429	111780	111081	141391	65978	129160	NA	0.322435811	0.491556729	89718.4	111902.5	Up	0.318766470393902	Mitogen-activated protein kinase kinase kinase kinase 4 OS=Mus musculus GN=Map4k4 PE=1 SV=1
PPP2R2D	Q925E7	2	2	15629.5	15779	14988.5	41689.5	13474	21947.5	13850	8066.5	11589.5	14415	0.322749435	0.49113451	20312.1	13973.7	Down	-0.539625330117897	Serine/threonine-protein phosphatase 2A 55 kDa regulatory subunit B delta isoform OS=Mus musculus GN=Ppp2r2d PE=2 SV=1
GPRIN1	Q3UNH4	66	66	37086.32	39017.55	39814.44	51520.91	37776.94	44657.15	48346.33	38428.74	59174.36	39126.15	0.323042414	0.490740453	41043.232	45946.546	Up	0.162812069761699	G protein-regulated inducer of neurite outgrowth 1 OS=Mus musculus GN=Gprin1 PE=1 SV=2
CDC42BPB	Q7TT50	11	11	11637.91	23747.09	15822.18	29085.09	23860.36	29883.09	34179.91	17518.45	22346.64	22870.18	0.323457238	0.490183127	20830.526	25359.654	Up	0.283835791973526	Serine/threonine-protein kinase MRCK beta OS=Mus musculus GN=Cdc42bpb PE=1 SV=2
ERLIN1	Q91X78	1	1	1059	1310	NA	5129	1154	3713	3008	NA	NA	NA	0.323996659	0.489459468	2163	3360.5	Up	0.635642238494048	Erlin-1 OS=Mus musculus GN=Erlin1 PE=1 SV=1
ENO2	P17183	41	41	61701.95	60567.73	64553.73	54358.39	66146.76	65727.15	67601.05	60718.98	61041.9	65220.9	0.324043606	0.489396544	61465.712	64061.996	Up	0.0596869067127679	Gamma-enolase OS=Mus musculus GN=Eno2 PE=1 SV=2
ARHGEF2	Q60875-5	14	14	38572.46	39598.54	33540.15	51928.46	51639.62	60443.08	58387.08	49386.62	61155	26218.23	0.324421186	0.488890793	43055.846	51118.002	Up	0.247622312217849	Isoform 5 of Rho guanine nucleotide exchange factor 2 OS=Mus musculus GN=Arhgef2
NIPSNAP1	O55125	8	8	70318.62	59999.25	60121.62	69627.25	63420.25	72280.12	50121.88	69379.38	82286.62	199267.12	0.324598362	0.488653675	64697.398	94667.024	Up	0.54915427847736	Protein NipSnap homolog 1 OS=Mus musculus GN=Nipsnap1 PE=1 SV=1
PITRM1	Q8K411-3	3	3	126257.67	125951	141144	99856.33	121128	130774.67	115119.67	157940.67	156538	115181	0.324796819	0.488388233	122867.4	135110.8	Up	0.137040840593509	Isoform 3 of Presequence protease, mitochondrial OS=Mus musculus GN=Pitrm1
VCL	Q64727	7	7	28675.43	28879.71	15758.57	27314.14	27999.29	25846.14	28102.43	36828.86	30438	24738.43	0.325032988	0.48807256	25725.428	29190.772	Up	0.182317286112089	Vinculin OS=Mus musculus GN=Vcl PE=1 SV=4
PGLS	Q9CQ60	12	12	48758.42	54284.75	66919.17	45560.42	50792.83	50317.58	52986.33	79912	67115.5	52041.5	0.325038682	0.488064952	53263.118	60474.582	Up	0.18319200657169	6-phosphogluconolactonase OS=Mus musculus GN=Pgls PE=2 SV=1
PACSIN2	Q9WVE8	8	8	33314.5	38107.12	22442	52355.75	37736.25	45758.88	37235	24225.75	27189.75	5966.5	0.32510517	0.487976124	36791.124	28075.176	Down	-0.390062685798652	Protein kinase C and casein kinase substrate in neurons protein 2 OS=Mus musculus GN=Pacsin2 PE=1 SV=1
SSB	P32067	1	1	20791	28151	20817	14394	27534	21847	26902	NA	43068	NA	0.326161742	0.486566982	22337.4	30605.667	Up	0.454337523302989	Lupus La protein homolog OS=Mus musculus GN=Ssb PE=1 SV=1
NDUFA13	Q9ERS2	8	8	24432.75	33349.5	18713.62	29629.38	25554	29150.88	31187.12	15663.88	22411.62	1548.25	0.326236992	0.486466796	26335.85	19992.35	Down	-0.397579960215448	NADH dehydrogenase [ubiquinone] 1 alpha subcomplex subunit 13 OS=Mus musculus GN=Ndufa13 PE=1 SV=3
NDUFA1	O35683	1	1	26979	66714	NA	38694	86792	69288	72932	94410	52303	NA	0.326323079	0.48635221	54794.75	72233.25	Up	0.398625411037227	NADH dehydrogenase [ubiquinone] 1 alpha subcomplex subunit 1 OS=Mus musculus GN=Ndufa1 PE=3 SV=1
SORCS3	Q8VI51	1	1	78269	68710	164823	11630	60396	43511	57439	181125	94772	262285	0.326604019	0.485978475	76765.6	127826.4	Up	0.735653962879745	VPS10 domain-containing receptor SorCS3 OS=Mus musculus GN=Sorcs3 PE=1 SV=1
TUBA1A	P68369	101	101	161202.88	196082.91	205666.41	219910.24	174227.77	206862.54	213619.55	196179.7	202070.03	198787.02	0.326685679	0.485869904	191418.04	203503.77	Up	0.0883286904998415	Tubulin alpha-1A chain OS=Mus musculus GN=Tuba1a PE=1 SV=1
GRSF1	Q8C5Q4	2	2	3037	3185	NA	3984	4166	4025	2371	NA	1762	NA	0.327176003	0.485218558	3593	2719.3333	Down	-0.401935928930797	G-rich sequence factor 1 OS=Mus musculus GN=Grsf1 PE=1 SV=2
ACAT1	Q8QZT1	74	74	96647.59	99295.64	130905.33	76128.37	88471	84461.75	95858.96	159616.75	101925.77	138277.62	0.327242632	0.485130123	98289.586	116028.17	Up	0.239364640816773	Acetyl-CoA acetyltransferase, mitochondrial OS=Mus musculus GN=Acat1 PE=1 SV=1
HCN2	O88703	4	4	2800	3214	2660.33	6540	5816.67	6133	7192.67	NA	3669.67	NA	0.327953208	0.484188117	4206.2	5665.1133	Up	0.429587367946808	Potassium/sodium hyperpolarization-activated cyclic nucleotide-gated channel 2 OS=Mus musculus GN=Hcn2 PE=1 SV=1
THOP1	Q8C1A5	2	2	6540.5	6319.5	11098	11049	7157	10997.5	8395.5	NA	10152	NA	0.328066886	0.484037603	8432.8	9848.3333	Up	0.223867854508666	Thimet oligopeptidase OS=Mus musculus GN=Thop1 PE=1 SV=1
DGKZ	Q80UP3	6	6	20460.83	17767.67	28946.5	12749	16544.33	15024.83	15388.33	34781.83	20085.5	56181	0.328180133	0.483887714	19293.666	28292.298	Up	0.552282065555733	Diacylglycerol kinase zeta OS=Mus musculus GN=Dgkz PE=1 SV=2
TOMM70A	Q9CZW5	31	31	32056.65	38395.19	47475.71	58319.61	45745.03	46473.77	43591.68	48581.39	45859.32	87257.13	0.328588003	0.483348297	44398.438	54352.658	Up	0.291841667468963	Mitochondrial import receptor subunit TOM70 OS=Mus musculus GN=Tomm70a PE=1 SV=2
MRPS28	Q9CY16	7	7	24091.5	22025.83	31581.17	19620.5	21541	24719.33	21278.17	30645.67	25283.83	31775.67	0.328916899	0.482913812	23772	26740.534	Up	0.169764989821062	28S ribosomal protein S28, mitochondrial OS=Mus musculus GN=Mrps28 PE=1 SV=1
CSE1L	Q9ERK4	1	1	NA	NA	NA	1796	673	2515	1761	NA	NA	NA	0.328982276	0.482827499	1234.5	2138	Up	0.792335016577709	Exportin-2 OS=Mus musculus GN=Cse1l PE=2 SV=1
MTPN	P62774	4	4	39148.5	48014.5	37018.25	94820.5	52567.75	56481.75	53762.25	17959.25	54704.25	15340.5	0.329498625	0.482146394	54313.9	39649.6	Down	-0.454015147888904	Myotrophin OS=Mus musculus GN=Mtpn PE=1 SV=2
ABCF2	Q99LE6	1	1	17076	30635	24477	34263	28788	31651	37387	NA	26243	NA	0.329544153	0.48208639	27047.8	31760.333	Up	0.231714800397729	ATP-binding cassette sub-family F member 2 OS=Mus musculus GN=Abcf2 PE=2 SV=1
POLR2D	Q9D7M8	1	1	4898	2961	NA	3066	4571	3768	3849	3117	1897	NA	0.329695094	0.481887515	3874	3157.75	Down	-0.294926996814318	DNA-directed RNA polymerase II subunit RPB4 OS=Mus musculus GN=Polr2d PE=2 SV=2
KCNJ10	Q9JM63	1	1	9493	11558	NA	6220	7589	10074	11118	31100	NA	NA	0.330007395	0.481476329	8715	17430.667	Up	1.00005517947796	ATP-sensitive inward rectifier potassium channel 10 OS=Mus musculus GN=Kcnj10 PE=1 SV=1
PITPNM1	O35954	3	3	756	8518.67	1835.33	26584.33	12371.33	20617.33	11128.67	NA	15531	NA	0.330062309	0.481404066	10013.132	15759	Up	0.654282686070395	Membrane-associated phosphatidylinositol transfer protein 1 OS=Mus musculus GN=Pitpnm1 PE=1 SV=1
PIP5K1A	P70182-3	2	2	3809.5	3750.5	4726.5	3287.5	2545.5	5913	3588.5	3831	4597	3270.5	0.330344783	0.481032547	3623.9	4240	Up	0.226521119419392	Isoform 3 of Phosphatidylinositol 4-phosphate 5-kinase type-1 alpha OS=Mus musculus GN=Pip5k1a
WDR37	Q8CBE3	5	5	67207.2	65253.6	68014.4	58845.8	70847	55561.8	65982	43639.6	71896.2	64592.4	0.33037917	0.480987342	66033.6	60334.4	Down	-0.130219503050337	WD repeat-containing protein 37 OS=Mus musculus GN=Wdr37 PE=2 SV=1
PHACTR4	Q501J7-3	2	2	24242	28250.5	26403.5	22796.5	26598.5	27820	26943.5	NA	46297	NA	0.330577595	0.480726584	25658.2	33686.833	Up	0.392764852455736	Isoform 3 of Phosphatase and actin regulator 4 OS=Mus musculus GN=Phactr4
UBQLN2	Q9QZM0	1	1	22412	9842	28758	27758	32832	27008	31483	NA	NA	NA	0.330672326	0.480602149	24320.4	29245.5	Up	0.266047697414542	Ubiquilin-2 OS=Mus musculus GN=Ubqln2 PE=1 SV=2

DDX42	Q810A7	2	2	2502	1872	1653.5	5677	2612.5	4368.5	4113	NA	2854	NA	0.330712414	0.480549502	2863.4	3778.5	Up	0.400084402436034	ATP-dependent RNA helicase DDX42 OS=Mus musculus GN=Ddx42 PE=1 SV=3
CCDC85A	Q5SP85-3	2	2	16883	15230.5	NA	20593	20249	21423.5	17721	NA	21360	NA	0.33117933	0.479936777	18238.875	20168.167	Up	0.145063200825859	Isoform 3 of Coiled-coil domain-containing protein 85A OS=Mus musculus GN=Ccdc85a
OXR1	Q4KMM3	18	18	32632.93	37595.2	32328.4	44275.87	41723.53	42970.87	43196.07	37254.8	40015.27	39151.67	0.331312361	0.479762361	37711.186	40517.736	Up	0.103561039950459	Oxidation resistance protein 1 OS=Mus musculus GN=Oxr1 PE=2 SV=3
AGAP3	Q8VHH5	4	4	72147.67	59951.67	115084.67	56127.33	66256	57977	84243.33	160832.33	82324.33	90568	0.331951675	0.478925135	73913.468	95188.998	Up	0.364957569414898	Arf-GAP with GTPase, ANK repeat and PH domain-containing protein 3 OS=Mus musculus GN=Agap3 PE=1 SV=1
PARG	O88622	1	1	7402	8635	10811	11180	9111	10446	11027	8625	11569	NA	0.331978619	0.478889885	9427.8	10416.75	Up	0.143912171668525	Poly(ADP-ribose) glycohydrolase OS=Mus musculus GN=Parg PE=1 SV=2
PPP1R1B	Q60829	17	17	21341.76	22227.06	17365.18	13398.06	23724.76	19551.35	15615.24	15010	23725.47	5639.41	0.332209497	0.478587957	19611.364	15908.294	Down	-0.301910750797713	Protein phosphatase 1 regulatory subunit 1B OS=Mus musculus GN=Ppp1r1b PE=2 SV=2
HOMER1	Q9Z2Y3-3	5	5	36673.5	44412.75	50015.5	177525.75	26604.25	48223	29530.75	25595.75	33720.5	43768	0.332819228	0.477791591	67046.35	36167.6	Down	-0.890460926825054	Isoform 3 of Homer protein homolog 1 OS=Mus musculus GN=Homer1
IDE	Q9JHR7	1	1	73436	102221	94394	78173	141314	98280	224968	32422	108628	270881	0.333038029	0.477506172	97907.6	147035.8	Up	0.586674705156337	Insulin-degrading enzyme OS=Mus musculus GN=Ide PE=1 SV=1
PTPN23	Q6PB44	3	3	22720	29593	43586	43391	36721.33	40016.67	41788.33	35999	34249.67	48907.67	0.333039858	0.477503787	35202.266	40192.268	Up	0.191249689824856	Tyrosine-protein phosphatase non-receptor type 23 OS=Mus musculus GN=Ptpn23 PE=1 SV=2
CMC1	Q9CPZ8	1	1	70460	62374	51938	34817	50516	41221	48571	24326	73589	26472	0.333190231	0.477307741	54021	42835.8	Down	-0.334703313819092	COX assembly mitochondrial protein homolog OS=Mus musculus GN=Cmc1 PE=3 SV=1
PRDX2	Q61171	15	15	55103.6	56183.4	63471.27	65780.87	54257.73	55945.13	53270.8	44621.47	54247.2	65532.47	0.333670607	0.476682048	58959.374	54723.414	Down	-0.107562969062076	Peroxiredoxin-2 OS=Mus musculus GN=Prdx2 PE=1 SV=3
SYBU	Q8BHS8-4	2	2	6197.5	4611	13579	4294	4266	6658.5	5770.5	13440.5	11075.5	NA	0.334246477	0.475933162	6589.5	9236.25	Up	0.487138223293562	Isoform 4 of Syntabulin OS=Mus musculus GN=Sybu
PPP3CC	P48455	2	2	43821.5	46413	79329	38704	38769.5	47157.5	52974	96250	58128	NA	0.334731218	0.475303783	49407.4	63627.375	Up	0.364920465402148	Serine/threonine-protein phosphatase 2B catalytic subunit gamma isoform OS=Mus musculus GN=Ppp3cc PE=2 SV=1
PSMA3	O70435	3	3	31675	35965.67	17840	28654.33	28315.33	35782.67	25953.67	NA	44554	NA	0.334950986	0.475018739	28490.066	35430.113	Up	0.31451711652704	Proteasome subunit alpha type-3 OS=Mus musculus GN=Psma3 PE=1 SV=3
PITPNM2	Q6ZPQ6	3	3	57555.33	62678	104243.67	39608	95352.33	52064.33	69033	123165.67	70781.67	306562	0.334966091	0.474999155	71887.466	124321.33	Up	0.790261734043205	Membrane-associated phosphatidylinositol transfer protein 2 OS=Mus musculus GN=Pitpnm2 PE=1 SV=2
VIP	P32648	2	2	56120	45373.5	109279	28989	47232.5	47707.5	45405	84996.5	96547.5	108600.5	0.335232058	0.474654457	57398.8	76651.4	Up	0.417291566524012	VIP peptides OS=Mus musculus GN=Vip PE=1 SV=1
KIF21A	Q9QXL2-3	15	15	33317.29	37265.57	30165.36	31529.43	43228.79	45423.36	42489.71	31863.64	43864.5	31588.5	0.335381794	0.474460517	35101.288	39045.942	Up	0.153648649036938	Isoform 3 of Kinesin-like protein KIF21A OS=Mus musculus GN=Kif21a
COX6A1	P43024	16	16	67265.6	54328.13	79465.2	26675.93	54978.13	45502.53	47868.27	109796.67	74417.47	80990.4	0.335551441	0.474240892	56542.598	71715.068	Up	0.342938101214762	Cytochrome c oxidase subunit 6A1, mitochondrial OS=Mus musculus GN=Cox6a1 PE=1 SV=2
NDUFA10	Q99LC3	17	17	70862.94	70589.82	110485.06	41148	62198.82	62082.82	63306.47	105833.82	76269.41	158139.71	0.335598904	0.474179466	71056.928	93126.446	Up	0.390215603692414	NADH dehydrogenase [ubiquinone] 1 alpha subcomplex subunit 10, mitochondrial OS=Mus musculus GN=Ndufa10 PE=1 SV=1
SMAP1	Q91VZ6	10	10	82261.1	76333.7	127644.8	43667.4	83301.8	56580.6	79197.3	136459.4	95822.4	173703.3	0.335865513	0.473834588	82641.76	108352.6	Up	0.390790888028508	Stromal membrane-associated protein 1 OS=Mus musculus GN=Smap1 PE=1 SV=1
NPY	P57774	2	2	20206.5	18574	30436.5	18150.5	20241.5	21523	16924.5	31479.5	23156	35847.5	0.335912701	0.473773575	21521.8	25786.1	Up	0.2607948458942	Pro-neuropeptide Y OS=Mus musculus GN=Npy PE=1 SV=2
TRMT112	Q9DCG9	3	3	29328.67	28111.67	42087.67	15319.33	26414.33	22783.33	23994.67	48280.67	36822	43502.67	0.336036245	0.473613876	28252.334	35076.668	Up	0.312141652618605	tRNA methyltransferase 112 homolog OS=Mus musculus GN=Trmt112 PE=2 SV=1
FMNL1	Q9JL26-2	2	2	864	4008.5	1394.5	4314.5	7082.5	7547	6800	3215	3218	NA	0.336554097	0.472945119	3532.8	5195	Up	0.556311671900767	Isoform 2 of Formin-like protein 1 OS=Mus musculus GN=Fmnl1
NAXD	Q9CZ42-3	5	5	54224.6	52296.6	49362.4	62075.4	44513.8	62141	52344.4	37517.6	49332	19785.4	0.336596838	0.472889968	52494.56	44224.08	Down	-0.247335793714934	Isoform 3 of ATP-dependent (S)-NAD(P)H-hydrate dehydratase OS=Mus musculus GN=Carkd
SMCR8	Q3UMB5	1	1	616	777	NA	2965	661	3128	1032	NA	2283	NA	0.336996971	0.472374002	1254.75	2147.6667	Up	0.775370147940766	Smith-Magenis syndrome chromosomal region candidate gene 8 protein homolog OS=Mus musculus GN=Smcr8 PE=1 SV=2
HDGFRP3	Q9JMG7	1	1	24040	14115	15582	6592	11015	10318	12574	28627	15295	31124	0.338000896	0.471082149	14268.8	19587.6	Up	0.457076630188258	Hepatoma-derived growth factor-related protein 3 OS=Mus musculus GN=Hdgfrp3 PE=1 SV=2
XKR7	Q5GH64	1	1	4339	5694	10055	5111	7862	7423	8242	6340	6695	36728	0.338802972	0.470052788	6612.2	13085.6	Up	0.984777808481547	XK-related protein 7 OS=Mus musculus GN=Xkr7 PE=2 SV=1
CELF6	Q7TN33	1	1	32965	35344	25273	39644	37043	36893	34157	NA	40855	NA	0.339688466	0.468919199	34053.8	37301.667	Up	0.131424297757919	CUGBP Elav-like family member 6 OS=Mus musculus GN=Celf6 PE=2 SV=1
VPS35	Q9EQH3	3	3	1566	4796	3505	8783	4372	7406	7920	NA	3925	5331	0.339780964	0.468800955	4604.4	6145.5	Up	0.41651722276487	Vacuolar protein sorting-associated protein 35 OS=Mus musculus GN=Vps35 PE=1 SV=1
SIK3	Q6P4S6	3	3	14090.67	21069.33	31339.67	15574.67	15153.33	18518.33	21038.67	19650.67	25561.67	34404	0.340403675	0.46800576	19445.534	23834.668	Up	0.293622676206157	Serine/threonine-protein kinase SIK3 OS=Mus musculus GN=Sik3 PE=1 SV=3
SFXN3	Q91V61	7	7	43031.57	75123.43	47512	104273	71092.43	93490.57	99572.29	94598.86	58718.43	65185.43	0.340540262	0.467831534	68206.486	82313.116	Up	0.271213395157877	Sideroflexin-3 OS=Mus musculus GN=Sfxn3 PE=1 SV=1
IPCEF1	Q5DU31-3	1	1	13376	15311	30890	7337	17015	13299	18483	20042	22985	37996	0.340570256	0.467793285	16785.8	22561	Up	0.426589719013076	Isoform 3 of Interactor protein for cytohesin exchange factors 1 OS=Mus musculus GN=Ipcef1
6430548M08RIK	Q8R0A7	8	8	45344.5	44704.38	57850.12	52882	68850	50971.75	61592.25	55559.38	53809.88	103724.5	0.341078179	0.467146064	53926.2	65131.552	Up	0.272370229118532	Uncharacterized protein KIAA0513 OS=Mus musculus GN=Kiaa0513 PE=2 SV=1
KALRN	A2CG49-7	1	1	1137	3635	2123	3564	3700	4085	3943	4977	1816	NA	0.341153546	0.467050111	2831.8	3705.25	Up	0.387851510510334	Isoform 7 of Kalirin OS=Mus musculus GN=Kalrn
CAR4	Q64444	1	1	8547	14505	23967	7663	12086	6843	15805	37432	17339	19080	0.341607399	0.466472731	13353.6	19299.8	Up	0.531357166381632	Carbonic anhydrase 4 OS=Mus musculus GN=Ca4 PE=1 SV=1
RPL5	P47962	7	7	87296.71	112839.86	122005.71	90747.14	92744.71	91431.57	112126.43	111898.29	116871.43	115537.86	0.341729097	0.466318041	101126.83	109573.12	Up	0.115728120416447	60S ribosomal protein L5 OS=Mus musculus GN=Rpl5 PE=1 SV=3
LRFN1	Q2WF71	3	3	3131.5	8061	6045.5	9356	10521	13505.5	17213.5	4251.5	6416	9527.5	0.342327651	0.46555802	7423	10182.8	Up	0.456060044893262	Leucine-rich repeat and fibronectin type III domain-containing protein 1 OS=Mus musculus GN=Lrfn1 PE=1 SV=1
CPT1C	Q8BGD5	1	1	509	5613	1407	7634	4321	5449	6139	NA	4512	NA	0.342582912	0.465234304	3896.8	5366.6667	Up	0.4617363942151	Carnitine O-palmitoyltransferase 1, brain isoform OS=Mus musculus GN=Cpt1c PE=1 SV=1
TCERG1	Q8CGF7-3	1	1	55153	52898	NA	34754	46848	57513	44370	NA	63816	NA	0.343136227	0.464533428	47413.25	55233	Up	0.220240202256493	Isoform 3 of Transcription elongation regulator 1 OS=Mus musculus GN=Tcerg1
PHF24	Q80TL4	18	18	20291.65	22335.06	23463.53	23950.18	23353.65	25003.18	23093.35	19635.18	24736.47	42529.82	0.343479185	0.464099576	22678.814	26999.6	Up	0.251592838068847	Protein KIAA1045 OS=Mus musculus GN=Kiaa1045 PE=2 SV=2
HIBADH	Q99L13	8	8	42510.12	47827	54930.62	27787.12	45138.25	37632.88	40172.75	66021.25	40758.38	101998.62	0.343616453	0.463926049	43638.622	57316.776	Up	0.393351916382777	3-hydroxyisobutyrate dehydrogenase, mitochondrial OS=Mus musculus GN=Hibadh PE=1 SV=1
ZFP598	Q80YR4-3	2	2	511.5	721.5	NA	2228	598.5	1872	2272	NA	796.5	NA	0.343811894	0.463679103	1014.875	1646.8333	Up	0.698392510798696	Isoform 3 of Zinc finger protein 598 OS=Mus musculus GN=Znf598
DNAL1	Q05A62-3	1	1	52470	45939	59393	31164	41586	46093	53065	21609	80710	129938	0.343944999	0.463511001	46110.4	66283	Up	0.523546720156464	Isoform 3 of Dynein light chain 1, axonemal OS=Mus musculus GN=Dnal1
SNX3	O70492	15	15	118997.07	110372.29	149158.57	60961.79	105738.79	88464.57	112079.64	140778.79	164795	136082.64	0.344000387	0.463441069	109045.7	128440.13	Up	0.236163100173305	Sorting nexin-3 OS=Mus musculus GN=Snx3 PE=1 SV=3
TUBA8	Q9JJZ2	40	40	284778.69	342659.26	349046.36	402476.28	306201.62	372452.38	378218.1	361163.28	372795.18	318094.33	0.344312145	0.463047658	337032.44	360544.65	Up	0.0972904817559186	Tubulin alpha-8 chain OS=Mus musculus GN=Tuba8 PE=1 SV=1
MYL4	P09541	1	1	9783	12750	NA	10164	14391	11472	13831	NA	23223	NA	0.344758094	0.462485528	11772	16175.333	Up	0.458435995484758	Myosin light chain 4 OS=Mus musculus GN=Myl4 PE=2 SV=3
CFL2	P45591	15	15	88123.67	83855.33	108680.27	99870.33	73614.13	79924.93	93729.07	79539.8	92076.13	71166.67	0.344872488	0.46234145	90828.746	83287.32	Down	-0.125052091591717	Cofilin-2 OS=Mus musculus GN=Cfl2 PE=1 SV=1
MAP2K1	P31938	4	4	13579.5	17685	15245.75	37449	12612	22699	15377.5	8003.25	14258.25	9188.5	0.345198982	0.461930494	19314.25	13905.3	Down	-0.47403078628663	Dual specificity mitogen-activated protein kinase kinase 1 OS=Mus musculus GN=Map2k1 PE=1 SV=2
HSPA4	Q61316	39	39	56501.86	58281	62843.22	67588.51	59259.16	61073.49	68396.68	54120.27	65652.43	77614.78	0.345508986	0.461540654	60894.75	65371.53	Up	0.102344610883245	Heat shock 70 kDa protein 4 OS=Mus musculus GN=Hspa4 PE=1 SV=1
MAST1	Q9R1L5	3	3	11419	14856.33	5822	16600.33	17179	15117	17845	11666.67	17944.67	15488.33	0.345548888	0.4614905	13175.332	15612.334	Up	0.244846916063552	Microtubule-associated serine/threonine-protein kinase 1 OS=Mus musculus GN=Mast1 PE=1 SV=3
GSN	P13020	3	3	5663	8000	NA	11638.67	8591.67	8082.33	7767	NA	2721.67	NA	0.345791668	0.461185475	8473.335	6190.3333	Down	-0.452912811377638	Gelsolin OS=Mus musculus GN=Gsn PE=1 SV=3
COQ9	Q8K1Z0	3	3	2765	2035.33	1253.33	6742	2987	4934.67	4390.67	NA	3389	NA	0.346021431	0.460897002	3156.532	4238.1133	Up	0.425081791199783	Ubiquinone biosynthesis protein COQ9, mitochondrial OS=Mus musculus GN=Coq9 PE=1 SV=1
TIMM13	P62075	6	6	59912.83	54549.67	82913.5	47297	55655	55635	57315.17	91249.17	59849	84935	0.346224032	0.460642791	60065.6	69796.668	Up	0.216619180094035	Mitochondrial import inner membrane translocase subunit Tim13 OS=Mus musculus GN=Timm13 PE=1 SV=1
LLGL1	Q80Y17	4	4	16241.25	23326.75	19162.5	18261.25	21690.25	17666.5	17301.25	14501	21641	NA	0.347173325	0.459453651	19736.4	17777.438	Down	-0.150811476112789	Lethal(2) giant larvae protein homolog 1 OS=Mus musculus GN=Llgl1 PE=1 SV=1
PTPRF	A2A8L5	2	2	5553.5	9239.5	3980	18225.5	11455.5	13949	12234.5	NA	11134	NA	0.347392078	0.45918009	9690.8	12439.167	Up	0.360202164886668	Receptor-type tyrosine-protein phosphatase F OS=Mus musculus GN=Ptprf PE=1 SV=1
MRPL10	Q3TBW2	1	1	59527	60770	120354	28446	48903	50209	50949	118108	71997	155814	0.347635409	0.458875995	63600	89415.4	Up	0.491496562434324	39S ribosomal protein L10, mitochondrial OS=Mus musculus GN=Mrpl10 PE=2 SV=2
DDAH2	Q99LD8	9	9	47415.12	56949.12	64846.88	34700.5	49481.12	40274.5	50988	66575.12	68633.88	63770.88	0.347648284	0.45885991	50678.548	58048.476	Up	0.195883001027691	N(G),N(G)-dimethylarginine dimethylaminohydrolase 2 OS=Mus musculus GN=Ddah2 PE=1 SV=1
PSMD2	Q8VDM4	1	1	2591	2653	NA	7720	192	4179	4136	7695	NA	NA	0.347684334	0.458814877	3289	5336.6667	Up	0.698289893847061	26S proteasome non-ATPase regulatory subunit 2 OS=Mus musculus GN=Psmd2 PE=1 SV=1
MPST	Q99J99	11	11	51881.45	61268.55	81402.73	52186.91	54567.45	53293.36	63731.27	66781.18	65229.45	105643.45	0.347721684	0.458768226	60261.418	70935.742	Up	0.235278112317575	3-mercaptopyruvate sulfurtransferase OS=Mus musculus GN=Mpst PE=1 SV=3
GLOD4	Q9CPV4	7	7	84397.14	86324	96433.29	77874.29	79098.57	77760.71	79670.43	71811.14	73562.71	95004.43	0.348706185	0.457540349	84825.458	79561.884	Down	-0.092419875399114	Glyoxalase domain-containing protein 4 OS=Mus musculus GN=Glod4 PE=1 SV=1
TMX2	Q9D710	5	5	12496.8	10946.6	17705.2	17617.8	16389.2	16296	19056.8	34269.4	17300.6	10157.6	0.348820438	0.457398077	15031.12	19416.08	Up	0.369299446478571	Thioredoxin-related transmembrane protein 2 OS=Mus musculus GN=Tmx2 PE=2 SV=1
NAPB	P28663	23	23	35606.78	39828.7	50636.78	35725.13	60539.7	37945.04	42572.83	54460.91	49338.17	114917.35	0.348966695	0.457216019	44467.418	59846.86	Up	0.428526917862897	Beta-soluble NSF attachment protein OS=Mus musculus GN=Napb PE=1 SV=2
EIF4B	Q8BGD9	9	9	108370.22	109928	138772.78	61902.11	88530.11	89332.56	98392.78	140104.44	136032.22	124915.11	0.349131313	0.457011199	101500.64	117755.42	Up	0.214304608634837	Eukaryotic translation initiation factor 4B OS=Mus musculus GN=Eif4b PE=1 SV=1
PSD2	Q6P1I6-3	1	1	6138	12312	9831	12596	12820	14721	14115	NA	9719	NA	0.349522197	0.456525238	10739.4	12851.667	Up	0.25904207368108	Isoform 3 of PH and SEC7 domain-containing protein 2 OS=Mus musculus GN=Psd2
PALM	Q9Z0P4-2	34	34	28028.23	39578.94	31652.9	49821.97	47773.71	53424.39	56897.87	40949.77	49274.32	29016.16	0.350094303	0.455814956	39371.15	45912.502	Up	0.221748200714427	Isoform 2 of Paralemmin-1 OS=Mus musculus GN=Palm
AARS	Q8BGQ7	12	12	22491.67	31401.83	42038.92	39242.83	32926	35260.75	37236	33300.67	41325.17	39898	0.350595294	0.455193918	33620.25	37404.118	Up	0.153866659635091	Alanine--tRNA ligase, cytoplasmic OS=Mus musculus GN=Aars PE=1 SV=1
UBXN2B	Q0KL01	2	2	6127	7881	NA	12503.5	8297	9077	6855.5	NA	3823	NA	0.350821154	0.454914227	8702.125	6585.1667	Down	-0.402147785642011	UBX domain-containing protein 2B OS=Mus musculus GN=Ubxn2b PE=1 SV=2
ERP44	Q9D1Q6	2	2	31860	45310	27788.5	32916.5	33796.5	34576	30685	NA	26197.5	NA	0.351277851	0.454349233	34334.3	30486.167	Down	-0.171495793424322	Endoplasmic reticulum resident protein 44 OS=Mus musculus GN=Erp44 PE=1 SV=1
ACAT2	Q8CAY6	13	13	70296	73679.08	65893	57271.46	83330	71376.85	86098.69	63931.23	102190.92	68167.23	0.351515287	0.454055783	70093.908	78352.984	Up	0.160699157224661	Acetyl-CoA acetyltransferase, cytosolic OS=Mus musculus GN=Acat2 PE=1 SV=2
TXN1	P10639	6	6	65557.17	70548.17	70150.33	105423	62503.5	75832.5	79603.33	97462.17	80508.5	84461.5	0.352557301	0.452770287	74836.434	83573.6	Up	0.159306467497293	Thioredoxin OS=Mus musculus GN=Txn PE=1 SV=3
AHDC1	Q6PAL7	1	1	8513	7302	7382	7621	9720	7293	9964	NA	12861	NA	0.352557909	0.452769539	8107.6	10039.333	Up	0.308316651367182	AT-hook DNA-binding motif-containing protein 1 OS=Mus musculus GN=Ahdc1 PE=1 SV=1
MRPS21	P58059	4	4	30405.25	28673	42576.25	19550.25	24399.25	26397.5	36925.5	33005.5	39238	32278.5	0.352626295	0.452685306	29120.8	33569	Up	0.205079571200435	28S ribosomal protein S21, mitochondrial OS=Mus musculus GN=Mrps21 PE=2 SV=1
CNPY4	Q8BQ47	3	3	26240.67	22533.33	16006.33	15939	21995.33	21976.67	20816.33	NA	31697.33	NA	0.353132431	0.452062396	20542.932	24830.11	Up	0.273448547113862	Protein canopy homolog 4 OS=Mus musculus GN=Cnpy4 PE=1 SV=1
P4HB	P09103	23	23	34370.79	39891.63	30133.11	53938.95	38671.21	39633.42	40473.79	30093.95	39687	17928.21	0.353446516	0.451676294	39401.138	33563.274	Down	-0.231353845441995	Protein disulfide-isomerase OS=Mus musculus GN=P4hb PE=1 SV=2
PPP2R1A	Q76MZ3	13	13	29249.92	40526	42070.85	39831.38	40566.62	39679.38	37018	29282.77	35442.38	36483.77	0.353560636	0.451536094	38448.954	35581.26	Down	-0.11182675026339	Serine/threonine-protein phosphatase 2A 65 kDa regulatory subunit A alpha isoform OS=Mus musculus GN=Ppp2r1a PE=1 SV=3
GLUD1	P26443	70	70	87618.43	91727.2	88608.24	76905.34	78666.81	81720.89	76385.33	86244.53	75304.29	85620.83	0.354159069	0.450801633	84705.204	81055.174	Down	-0.0635463262378076	Glutamate dehydrogenase 1, mitochondrial OS=Mus musculus GN=Glud1 PE=1 SV=1
CAP1	P40124	14	14	103592.15	103452.85	219659.54	61526.38	98841	85654.46	106919.15	221083.31	113564.54	311952	0.354205351	0.450744882	117414.38	167834.69	Up	0.515431798075271	Adenylyl cyclase-associated protein 1 OS=Mus musculus GN=Cap1 PE=1 SV=4
TUBB2A	Q7TMM9	128	128	67028.1	75728.37	62615.87	88863.89	61362.08	73100.07	72132.86	60953.8	62292.65	57246.33	0.354234395	0.450709272	71119.662	65145.142	Down	-0.126590869470406	Tubulin beta-2A chain OS=Mus musculus GN=Tubb2a PE=1 SV=1
PDIA3	P27773	57	57	92149.56	111925.42	84892.58	120531.33	101322.05	108273.63	108055.53	88710.7	93043.49	54187.25	0.354560917	0.450309138	102164.19	90454.12	Down	-0.175631450267918	Protein disulfide-isomerase A3 OS=Mus musculus GN=Pdia3 PE=1 SV=2
GARNL3	Q3V0G7	1	1	11668	9500	NA	32277	21520	44675	33904	NA	12567	NA	0.354603909	0.450256482	18741.25	30382	Up	0.696999662565479	GTPase-activating Rap/Ran-GAP domain-like protein 3 OS=Mus musculus GN=Garnl3 PE=1 SV=1
CMPK1	Q9DBP5	19	19	139958.58	157414.42	170136.11	100612.58	133129.05	111209.74	142350.95	159764.26	166355.26	264082	0.354807069	0.450007736	140250.15	168752.44	Up	0.266906086163224	UMP-CMP kinase OS=Mus musculus GN=Cmpk1 PE=1 SV=1
TXNDC5	Q91W90	4	4	17511.75	16786.75	6819.5	22567.25	20017.25	19591.75	17821.25	22772	18524.75	NA	0.354834813	0.449973778	16740.5	19677.438	Up	0.233199739034971	Thioredoxin domain-containing protein 5 OS=Mus musculus GN=Txndc5 PE=1 SV=2
EIF3K	Q9DBZ5	1	1	6258	6986	15954	7216	5819	7311	7956	16859	13527	NA	0.354839329	0.44996825	8446.6	11413.25	Up	0.434267030271741	Eukaryotic translation initiation factor 3 subunit K OS=Mus musculus GN=Eif3k PE=1 SV=1
CAPN5	O08688	5	5	17910.2	26628.2	32171.6	30361	35390.4	35534.6	37250.4	21049.4	42125.6	NA	0.354884657	0.449912776	28492.28	33990	Up	0.254539288723252	Calpain-5 OS=Mus musculus GN=Capn5 PE=2 SV=1
CHMP2B	Q8BJF9	11	11	37504.73	38685.64	43198	31477.27	33684.55	35025.45	38813.27	48126.64	52762.55	31998.36	0.355704392	0.448910772	36910.038	41345.254	Up	0.163708509811986	Charged multivesicular body protein 2b OS=Mus musculus GN=Chmp2b PE=1 SV=1
HNRNPDL	Q9Z130	4	4	101098.75	81018.5	53126.25	72313.25	81222.25	71494.75	77556.5	42208.75	86822.5	56420.75	0.355890458	0.448683656	77755.8	66900.65	Down	-0.216930065480917	Heterogeneous nuclear ribonucleoprotein D-like OS=Mus musculus GN=Hnrnpdl PE=1 SV=1
JUP	Q02257	3	3	9967	13409	16616.33	22306.67	14528.33	18638	18396.67	NA	15828	NA	0.356012076	0.44853527	15365.466	17620.89	Up	0.197595272786609	Junction plakoglobin OS=Mus musculus GN=Jup PE=1 SV=3
SNRPG	P62309	1	1	14834	15272	19060	10108	12905	12030	12021	24857	12702	37324	0.356235732	0.448262522	14435.8	19786.8	Up	0.454887252122642	Small nuclear ribonucleoprotein G OS=Mus musculus GN=Snrpg PE=1 SV=1
HABP4	Q9JKS5	3	3	32402	29974.5	16915.5	26697.5	31623.5	31253.5	27292	NA	55218	NA	0.356272599	0.448217578	27522.6	37921.167	Up	0.462386585535798	Intracellular hyaluronan-binding protein 4 OS=Mus musculus GN=Habp4 PE=1 SV=2
MAGI1	Q6RHR9-2	1	1	15843	16005	6038	21109	22301	23082	21020	11922	25229	NA	0.356401483	0.448060497	16259.2	20313.25	Up	0.321164806424126	Isoform 2 of Membrane-associated guanylate kinase, WW and PDZ domain-containing protein 1 OS=Mus musculus GN=Magi1
KRAS	P32883	22	22	62465.1	67473.55	68125	77754.3	63332.05	67890	70813.15	74692.8	72114.1	68623	0.356571443	0.447853441	67830	70826.61	Up	0.0623679988879526	GTPase KRas OS=Mus musculus GN=Kras PE=1 SV=1
RIDA	P52760	9	9	44858.78	46813.56	48538.33	33795.44	48850.44	40571.78	42563.44	60948.78	46030.89	56912	0.35668232	0.447718417	44571.31	49405.378	Up	0.148552730298521	Ribonuclease UK114 OS=Mus musculus GN=Hrsp12 PE=1 SV=3
GNAZ	O70443	2	2	10294	12083.5	12110	13682.5	10693.5	12089	15752	13938	7196	27993	0.357389533	0.446858171	11772.7	15393.6	Up	0.386885432718642	Guanine nucleotide-binding protein G(z) subunit alpha OS=Mus musculus GN=Gnaz PE=2 SV=4
ZYX	Q62523	3	3	68880.33	71536.33	50325.67	75791.33	75691.33	76370.33	84394.33	95645	101961	41753	0.35773961	0.44643297	68444.998	80024.732	Up	0.225500827895838	Zyxin OS=Mus musculus GN=Zyx PE=1 SV=2
MT1	P02802	5	5	32562	35239.2	57420.8	25417.8	21007	38248.8	33896.8	58986.2	76437.4	21367.4	0.357850847	0.446297951	34329.36	45787.32	Up	0.415505162917128	Metallothionein-1 OS=Mus musculus GN=Mt1 PE=1 SV=1
BASP1	Q91XV3	50	50	73930.88	72895.08	102739.21	101557.42	52544.9	71890.48	70628.4	135904.1	87504.02	116012.94	0.358347929	0.445695101	80733.498	96387.988	Up	0.255685965302183	Brain acid soluble protein 1 OS=Mus musculus GN=Basp1 PE=1 SV=3
RARS	Q9D0I9	1	1	5111	12632	24168	16572	15662	18374	16802	NA	19009	NA	0.35879636	0.445151972	14829	18061.667	Up	0.284509713441534	Arginine--tRNA ligase, cytoplasmic OS=Mus musculus GN=Rars PE=2 SV=2
TIMM9	Q9WV98	10	10	138983	131356.2	225455.7	53319.6	128088.4	86681.3	106347.7	275776.3	159682.6	304593.9	0.358858681	0.445076543	135440.58	186616.36	Up	0.462415411292235	Mitochondrial import inner membrane translocase subunit Tim9 OS=Mus musculus GN=Timm9 PE=1 SV=1
PDXK	Q8K183	4	4	83358	80358.5	114253	81397.75	92930	87957.5	89704	105010.75	86238.75	158150.25	0.359850828	0.443877493	90459.45	105412.25	Up	0.22069940347054	Pyridoxal kinase OS=Mus musculus GN=Pdxk PE=1 SV=1
SON	Q9QX47-3	1	1	2318	1076	NA	1465	1726	1992	3455	NA	NA	NA	0.360537809	0.443049185	1646.25	2723.5	Up	0.726278426696567	Isoform 3 of Protein SON OS=Mus musculus GN=Son
CISD1	Q91WS0	13	13	143788	157458.46	245206.31	105376.08	140146.38	109678.31	130687.54	284745	156108.15	462304.31	0.360585374	0.442991893	158395.05	228704.66	Up	0.529958560415785	CDGSH iron-sulfur domain-containing protein 1 OS=Mus musculus GN=Cisd1 PE=1 SV=1
SNCB	Q91ZZ3	28	28	126096.93	115328.36	106815.64	173360.25	112806.71	122369.75	119732.46	115454.54	128640.32	71564.82	0.360732962	0.442814173	126881.58	111552.38	Down	-0.185761350435631	Beta-synuclein OS=Mus musculus GN=Sncb PE=1 SV=1

UQCRC1	Q9CZ13	43	43	40483.24	46060.4	36212.71	59349.07	44014.31	51549.62	47496.88	32999.02	37655.74	27798.9	0.361091795		0.44238238	45223.946	39500.032	Down	-0.195233057391034	Cytochrome b-c1 complex subunit 1, mitochondrial OS=Mus musculus GN=Uqcrc1 PE=1 SV=2
PHF24	Q80TL4-2	13	13	24135	25990.23	30099.38	25254.69	27858.92	28105.15	25687.08	24580.77	29457.46	55615.92	0.361119402		0.442349178	26667.644	32689.276	Up	0.293727051658078	Isoform 2 of Protein KIAA1045 OS=Mus musculus GN=Kiaa1045
ARPIN	Q9D0A3	4	4	12202	15216	13209	15715.33	16150	20177.33	13028	5870	11461	9394.33	0.361348281		0.442074007	14498.466	11986.132	Down	-0.274534097865327	Arpin OS=Mus musculus GN=Arpin PE=1 SV=1
MAPRE3	Q6PER3	6	6	94717.83	94981.5	124312	66859	90862.5	83005.5	90655.83	166863	96257	122198.17	0.361360792		0.442058971	94346.566	111795.9	Up	0.244825366707773	Microtubule-associated protein RP/EB family member 3 OS=Mus musculus GN=Mapre3 PE=1 SV=1
KLC4	Q9DBS5	2	2	103198	109464	102707	140131	125220.5	120651	119703	92912.5	116137	75314	0.361368708		0.442049457	116144.1	104943.5	Down	-0.146303056972836	Kinesin light chain 4 OS=Mus musculus GN=Klc4 PE=1 SV=1
ARHGAP39	P59281	6	6	23435	27432.5	29288.5	25480.5	35641.5	35385	48534.67	31147.5	43080.17	14379	0.361400432		0.442011333	28255.6	34505.268	Up	0.288279813582037	Rho GTPase-activating protein 39 OS=Mus musculus GN=Arhgap39 PE=1 SV=2
APLP2	Q06335	4	4	19477.25	45849.75	40952	86856.5	29595.25	49304.75	41355.75	19885.25	23701.25	24877.5	0.361821713		0.441505375	44546.15	31824.9	Down	-0.485144768103882	Amyloid-like protein 2 OS=Mus musculus GN=Aplp2 PE=1 SV=4
SERPINI1	O35684	1	1	1372	NA	NA	1989	1711	2396	4414	NA	1383	NA	0.363171045		0.439888784	1690.6667	2731	Up	0.691837069347035	Neuroserpin OS=Mus musculus GN=Serpini1 PE=1 SV=1
SPEG	Q62407	6	6	11611	12936	873.5	18677.17	17795.33	21587.67	21440.33	9713.83	17812.17	10981.33	0.363596915		0.43937981	12378.6	16307.066	Up	0.397649075554347	Striated muscle-specific serine/threonine-protein kinase OS=Mus musculus GN=Speg PE=1 SV=2
MEA1	Q64327	1	1	14807	12860	9982	12664	18004	13602	15285	NA	16947	NA	0.363743965		0.439204203	13663.4	15278	Up	0.161139168096835	Male-enhanced antigen 1 OS=Mus musculus GN=Mea1 PE=2 SV=1
PKIG	O70139	1	1	5162	5578	2815	3179	4187	4092	4695	NA	7000	NA	0.364598645		0.43818495	4184.2	5262.3333	Up	0.330750823986425	cAMP-dependent protein kinase inhibitor gamma OS=Mus musculus GN=Pkig PE=1 SV=1
HSBP1	Q9CQZ1	1	1	28752	38818	37010	22809	33856	16413	27083	31973	11310	43232	0.364682476		0.438085105	32249	26002.2	Down	-0.310620732827255	Heat shock factor-binding protein 1 OS=Mus musculus GN=Hsbp1 PE=2 SV=1
EFHD2	Q9D8Y0	14	14	35415.46	23861	35978.15	23114.77	27764	29779.77	26748.31	11556.62	34838.69	19320.38	0.36478812		0.437959313	29226.676	24448.754	Down	-0.257524816379239	EF-hand domain-containing protein D2 OS=Mus musculus GN=Efhd2 PE=1 SV=1
CALU	O35887	5	5	32048.4	33330.6	52533.6	27073.6	26071.6	29407.8	33920.2	63667.2	39391.6	41302.6	0.364993792		0.437714522	34211.56	41537.88	Up	0.279943695638561	Calumenin OS=Mus musculus GN=Calu PE=1 SV=1
SLC2A3	P32037	1	1	25754	42815	42927	60816	45070	54417	52937	84837	35068	40075	0.365168626		0.437506542	43476.4	53466.8	Up	0.298410848649267	Solute carrier family 2, facilitated glucose transporter member 3 OS=Mus musculus GN=Slc2a3 PE=1 SV=1
ERBIN	Q80TH2-2	1	1	45277	42483	68875	12249	38723	27196	45895	49812	61589	87720	0.36545897		0.437161374	41521.4	54442.4	Up	0.390875578322571	Isoform 2 of Protein LAP2 OS=Mus musculus GN=Erbb2ip
BIN1	O08539	39	39	97261.95	107203.44	81022.69	135243.54	115666.23	112369.08	107267.18	80959.77	112232.56	52189.41	0.365804445		0.436751022	107279.57	93003.6	Down	-0.206016893216998	Myc box-dependent-interacting protein 1 OS=Mus musculus GN=Bin1 PE=1 SV=1
BAIAP2	Q8BKX1-4	40	40	85369.75	81317.7	121476.12	40945.45	78262.4	56626.6	70516.02	128234.45	93476.6	193253.38	0.365856699		0.436688988	81474.284	108421.41	Up	0.412233000477894	Isoform 4 of Brain-specific angiogenesis inhibitor 1-associated protein 2 OS=Mus musculus GN=Baiap2
NMT2	O70311	1	1	19666	17740	30515	9012	18104	21180	19415	NA	40874	NA	0.366227304		0.43624928	19007.4	27156.333	Up	0.514727498146312	Glycylpeptide N-tetradecanoyltransferase 2 OS=Mus musculus GN=Nmt2 PE=2 SV=1
ARFGAP2	Q99K28	6	6	28478.83	28659	21429.67	33048.67	35273.67	34651.67	42300.33	19905	46167.17	NA	0.366303733		0.436158656	29377.968	35756.043	Up	0.2834524552487	ADP-ribosylation factor GTPase-activating protein 2 OS=Mus musculus GN=Arfgap2 PE=1 SV=1
SHISA7	Q8C3Q5	14	14	73304	74960.21	75678.07	55896.57	75824.79	73139.86	83906.5	68371.14	103987.5	65216.29	0.36656541		0.435848519	71132.728	78924.258	Up	0.149955299050426	Protein shisa-7 OS=Mus musculus GN=Shisa7 PE=1 SV=3
COX6B1	P56391	37	37	436232.69	374883	801486.03	135271.06	368048.47	248739.36	301241.69	883565.69	512935.19	1173059.7	0.366569357		0.435843842	423184.25	623908.32	Up	0.560048123099106	Cytochrome c oxidase subunit 6B1 OS=Mus musculus GN=Cox6b1 PE=1 SV=2
COPS8	Q8VBV7	1	1	52283	49624	72002	28164	42655	34222	41851	114314	36747	121976	0.366599827		0.435807744	48945.6	69822	Up	0.512502508877101	COP9 signalosome complex subunit 8 OS=Mus musculus GN=Cops8 PE=1 SV=1
NDUFB6	Q3UIU2	4	4	11138.5	18971	5921	17917	17931	21173.75	18800.75	NA	13294.75	NA	0.366723782		0.435660926	14375.7	17756.417	Up	0.304708260692915	NADH dehydrogenase [ubiquinone] 1 beta subcomplex subunit 6 OS=Mus musculus GN=Ndufb6 PE=1 SV=3
MAPRE2	Q8R001	11	11	86123.33	88090.89	138339.78	90338.44	85919.56	99325.89	95623.11	108732	97206	180414.33	0.367009788		0.435322353	97762.4	116260.27	Up	0.25000650664945	Microtubule-associated protein RP/EB family member 2 OS=Mus musculus GN=Mapre2 PE=1 SV=1
PSIP1	Q99JF8	4	4	129849.5	89163.25	75103.5	44627.75	92195.75	67283.5	96259.5	51132	92811.25	37787	0.367441116		0.434812249	86187.95	69054.65	Down	-0.319747613259115	PC4 and SFRS1-interacting protein OS=Mus musculus GN=Psip1 PE=1 SV=1
LHPP	Q9D7I5	3	3	11167	12115.33	13797.67	13410	10559.67	12378.67	12804	20099.33	13881.67	10509.67	0.368315474		0.433780035	12209.934	13934.668	Up	0.190623228012598	Phospholysine phosphohistidine inorganic pyrophosphate phosphatase OS=Mus musculus GN=Lhpp PE=2 SV=1
MTA1	Q8K4B0	1	1	7524	7418	7792	10960	8474	11116	8778	NA	8556	NA	0.368460822		0.433608684	8433.6	9483.3333	Up	0.169245649517981	Metastasis-associated protein MTA1 OS=Mus musculus GN=Mta1 PE=1 SV=1
ZRANB2	Q9R020-2	1	1	7309	10084	5008	6026	7077	6036	9653	6634	15589	NA	0.36928718		0.432635769	7100.8	9478	Up	0.416601088322952	Isoform 2 of Zinc finger Ran-binding domain-containing protein 2 OS=Mus musculus GN=Zranb2
EIF4ENIF1	Q9EST3	1	1	3398	4344	5168	10468	5787	10143	6536	8323	4790	NA	0.369292234		0.432629825	5833	7448	Up	0.352614998745029	Eukaryotic translation initiation factor 4E transporter OS=Mus musculus GN=Eif4enif1 PE=1 SV=2
ASAP2	Q7SIG6	1	1	NA	NA	NA	2785	1835	1762	1475	NA	NA	NA	0.369372259		0.432535724	2310	1618.5	Down	-0.513235486094247	Arf-GAP with SH3 domain, ANK repeat and PH domain-containing protein 2 OS=Mus musculus GN=Asap2 PE=1 SV=3
PRPF6	Q91YR7	1	1	939	NA	NA	1933	2116	1866	2145	NA	2241	NA	0.369752554		0.432088818	1662.6667	2084	Up	0.325856313169994	Pre-mRNA-processing factor 6 OS=Mus musculus GN=Prpf6 PE=2 SV=1
VASH1	Q8C1W1	1	1	NA	NA	NA	5972	1416	7772	6174	NA	NA	NA	0.369862103		0.431960165	3694	6973	Up	0.91659561530436	Vasohibin-1 OS=Mus musculus GN=Vash1 PE=2 SV=4
NAP1L1	P28656	16	16	44958.62	46615.62	49824.75	39141.56	45579	42024	46189.19	46887	47728.81	60645.69	0.369918733		0.431893675	45223.91	48694.938	Up	0.106686076172195	Nucleosome assembly protein 1-like 1 OS=Mus musculus GN=Nap1l1 PE=1 SV=2
CSNK2B	P67871	6	6	30310.67	28223	34936.17	19915.67	30799.83	25874.5	26215.33	34861.67	38272.17	35986.67	0.370303997		0.4314416	28837.068	32242.068	Up	0.161019794552453	Casein kinase II subunit beta OS=Mus musculus GN=Csnk2b PE=1 SV=1
HCLS1	P49710	1	1	54872	47640	89798	25239	40243	48305	54483	35988	88145	120937	0.370356215		0.431380363	51558.4	69571.6	Up	0.432291006458401	Hematopoietic lineage cell-specific protein OS=Mus musculus GN=Hcls1 PE=1 SV=2
NSF	P46460	82	82	59424.49	75760.83	67168.97	87328.63	76577.05	84359.36	91146.65	79031.78	69999.78	71079.01	0.370544744		0.431159343	73251.994	79123.316	Up	0.111234857834219	Vesicle-fusing ATPase OS=Mus musculus GN=Nsf PE=1 SV=2
ETL4	A2AQ25-6	13	13	44226.08	44822.08	44536.23	39135.46	50310.23	49177.15	50270.46	46951	63899.31	36086.77	0.371356291		0.430209214	44606.016	49276.938	Up	0.143674312650699	Isoform 6 of Sickle tail protein OS=Mus musculus GN=Skt
TNR	Q8BYI9-2	20	20	15633.47	17711.07	18583.33	25891.07	18765	20573.53	20970.8	14578.07	22629.93	45138.47	0.371376938		0.430185069	19316.788	24778.16	Up	0.359213835996363	Isoform 2 of Tenascin-R OS=Mus musculus GN=Tnr
ATP5E	P56382	9	9	123129.56	110927.67	121919	90140.22	109737.22	89319.67	90420.44	94595	131771.11	102942.22	0.371695873		0.429812261	111170.73	101809.69	Down	-0.126902192793897	ATP synthase subunit epsilon, mitochondrial OS=Mus musculus GN=Atp5e PE=1 SV=2
SH3GL2	Q62420	28	28	93669.21	80428.61	96693.68	57829.21	88272.61	70489.64	72929.11	112064.39	97342.5	124894.61	0.371906424		0.42956632	83378.664	95544.05	Up	0.196487776633369	Endophilin-A1 OS=Mus musculus GN=Sh3gl2 PE=1 SV=2
BAIAP2	Q8BKX1-3	45	45	78610.62	74902.38	111859.87	38223	72621.27	52737.24	65489.53	116002.53	86094.87	174299.18	0.372340702		0.429059487	75243.428	98924.67	Up	0.394764769752848	Isoform 3 of Brain-specific angiogenesis inhibitor 1-associated protein 2 OS=Mus musculus GN=Baiap2
PDP1	Q3UV70	2	2	27576.5	53084.5	30244.5	53686.5	40332.5	57514.5	52573	NA	37114.5	NA	0.372572306		0.42878943	40984.9	49067.333	Up	0.259670389426543	[Pyruvate dehydrogenase [acetyl-transferring]]-phosphatase 1, mitochondrial OS=Mus musculus GN=Pdp1 PE=2 SV=1
TCP1	P11983-2	7	7	22673.5	20025.33	21980.5	29527.17	23414.83	25210	25314.33	30109.67	22199	NA	0.372821128		0.428499483	23524.266	25708.25	Up	0.128081698110431	Isoform 2 of T-complex protein 1 subunit alpha OS=Mus musculus GN=Tcp1
FAM195B	Q3UGS4	5	5	23647.4	22682.4	16485.8	32371.2	21399.8	32916.2	26585.2	27011.6	26598.2	19329	0.3737071		0.427468651	23317.32	26488.04	Up	0.183939114047142	Protein FAM195B OS=Mus musculus GN=Fam195b PE=1 SV=1
MRPL28	Q9D1B9	4	4	35084	33318.33	38745	24414.33	29545.67	28634.33	31038	40291.33	39323	37768	0.374321888		0.426754777	32221.466	35410.932	Up	0.136172680593357	39S ribosomal protein L28, mitochondrial OS=Mus musculus GN=Mrpl28 PE=2 SV=3
CACNG8	Q8VHW2	5	5	30587.6	59505.4	99773.4	82748	55014.2	85452.4	127869	93946.6	101663	20287.6	0.375555574		0.425325788	65525.72	85843.72	Up	0.389651294958161	Voltage-dependent calcium channel gamma-8 subunit OS=Mus musculus GN=Cacng8 PE=1 SV=1
SIRPA	P97797	6	6	7697.67	10046.5	12871.33	35438.67	12296.83	29849.67	25695.33	18993.67	16693.33	14643.17	0.375854339		0.424980432	15670.2	21175.034	Up	0.434340692884791	Tyrosine-protein phosphatase non-receptor type substrate 1 OS=Mus musculus GN=Sirpa PE=1 SV=1
C1QTNF4	Q8R066	5	5	41751.2	46999	34464.4	31872	35447.2	35208.6	39922.8	14920.8	44225.6	28510.4	0.376074878		0.424725677	38106.76	32557.64	Down	-0.227050822241887	Complement C1q tumor necrosis factor-related protein 4 OS=Mus musculus GN=C1qtnf4 PE=1 SV=1
HOMER1	Q9Z2Y3-5	2	2	48924.5	51147	37388.5	340310.5	31483	69326	32218	NA	23050	NA	0.376269955		0.424500459	101850.7	41531.333	Down	-1.29418380140284	Isoform 5 of Homer protein homolog 1 OS=Mus musculus GN=Homer1
HMGB3	O54879	1	1	7054	5694	2210	1913	5546	4013	3869	NA	6365	13236	0.376967039		0.423696621	4483.4	6870.75	Up	0.615874370402512	High mobility group protein B3 OS=Mus musculus GN=Hmgb3 PE=2 SV=3
PRDX1	P35700	11	11	76526.91	83558.91	92859	67333.91	76468.36	74116.64	85344.55	111751.45	80956.91	81218.55	0.378336698		0.422121531	79349.418	86677.62	Up	0.127439898916755	Peroxiredoxin-1 OS=Mus musculus GN=Prdx1 PE=1 SV=1
MT2	P02798	3	3	94580.33	59511.33	106198.33	20571.33	44072.67	39962.33	40729.33	156762.33	69007	175803.67	0.37837914		0.422072815	64986.798	96452.932	Up	0.569678428421349	Metallothionein-2 OS=Mus musculus GN=Mt2 PE=1 SV=2
COMMD1	Q8K4M5	1	1	3898	3485	2289	6604	5093	5829	4705	NA	4662	NA	0.378489422		0.421946253	4273.8	5065.3333	Up	0.245137815160381	COMM domain-containing protein 1 OS=Mus musculus GN=Commd1 PE=2 SV=2
DTD1	Q9DD18	4	4	44586.25	46918	29553	45198.25	50426.75	61010.25	54044.5	48143.25	53642.25	29850.75	0.378553475		0.421872762	43336.45	49338.2	Up	0.187124106162217	D-tyrosyl-tRNA(Tyr) deacylase 1 OS=Mus musculus GN=Dtd1 PE=1 SV=2
SNX32	Q80ZJ7	1	1	23863	26490	25372	21710	25821	24699	30709	22702	28266	NA	0.37856473		0.421859851	24651.2	26594	Up	0.109442911573064	Sorting nexin-32 OS=Mus musculus GN=Snx32 PE=2 SV=1
ALDH6A1	Q9EQ20	9	9	58990.56	68009.44	108842.11	49993.44	62668	56159	60356.44	113921.22	63349.11	172255.22	0.378803055		0.421586527	69700.71	93208.198	Up	0.419283498684305	Methylmalonate-semialdehyde dehydrogenase [acylating], mitochondrial OS=Mus musculus GN=Aldh6a1 PE=1 SV=1
SVIP	Q3UZP4	1	1	10841	11030	20750	9615	10344	7155	11974	21258	13213	31898	0.37893322		0.421437319	12516	17099.6	Up	0.450189013772571	Small VCP/p97-interacting protein OS=Mus musculus GN=Svip PE=2 SV=1
PTK2B	Q9QVP9	2	2	2738.5	3675	7161	13919.5	6606.5	14448.5	8679.5	NA	6380.5	NA	0.379492782		0.42079648	6820.1	9836.1667	Up	0.528303287575236	Protein-tyrosine kinase 2-beta OS=Mus musculus GN=Ptk2b PE=1 SV=2
BLVRB	Q923D2	4	4	28370.5	29827.75	40939.25	36550.5	29072	35932.5	26467.75	35623	29496.25	17825.25	0.380069935		0.420136483	32952	29068.95	Down	-0.180887070513773	Flavin reductase (NADPH) OS=Mus musculus GN=Blvrb PE=2 SV=3
MLST8	Q9DCJ1	3	3	38972.33	30533	48526.67	25580.67	32621.33	35958	25974	53174.67	59648.67	NA	0.380123972		0.420074742	35246.8	43688.835	Up	0.309772352096904	Target of rapamycin complex subunit LST8 OS=Mus musculus GN=Mlst8 PE=1 SV=1
ERLIN2	Q8BFZ9	4	4	29219	36952.75	59372.75	31262	28463.25	28405.5	35440	108236	40628	46855	0.380257589		0.419922109	37053.95	51912.9	Up	0.486465739041399	Erlin-2 OS=Mus musculus GN=Erlin2 PE=1 SV=1
SARNP	Q9D1J3	1	1	134713	90003	95996	34834	107955	66130	67918	23878	117771	NA	0.380274346		0.419902972	92700.2	68924.25	Down	-0.427560787839511	SAP domain-containing ribonucleoprotein OS=Mus musculus GN=Sarnp PE=1 SV=3
CSTB	Q62426	2	2	65781.5	54682.5	95586	47359.5	56356.5	52558.5	53260.5	128284.5	68910	95282	0.380861475		0.419232955	63953.2	79659.1	Up	0.31682263156538	Cystatin-B OS=Mus musculus GN=Cstb PE=1 SV=1
NAA38	Q9D2U5	1	1	8328	3035	NA	11411	6910	9761	8500	NA	9392	NA	0.380924511		0.419161081	7421	9217.6667	Up	0.312787990203369	N-alpha-acetyltransferase 38, NatC auxiliary subunit OS=Mus musculus GN=Naa38 PE=2 SV=1
G3BP1	P97855	7	7	49055.29	58068.57	66574.71	55980.43	55900.57	53176.29	65459.43	68312.29	72073.57	50460.86	0.381044943		0.419023797	57115.914	61896.488	Up	0.115964778915708	Ras GTPase-activating protein-binding protein 1 OS=Mus musculus GN=G3bp1 PE=1 SV=1
KPNB1	P70168	5	5	32847.2	42813.4	46688.4	46956	44860.4	48532.6	60361.8	27938.4	46627.6	62744	0.381141263		0.418914031	42833.08	49240.88	Up	0.20113112404239	Importin subunit beta-1 OS=Mus musculus GN=Kpnb1 PE=1 SV=2
PGK1	P09411	74	74	115932.83	114729.67	143546.67	98594.9	121699.47	108708.29	119543.58	138092.32	128882.58	143897.32	0.381434347		0.418580203	118900.71	127824.82	Up	0.104410666176645	Phosphoglycerate kinase 1 OS=Mus musculus GN=Pgk1 PE=1 SV=4
ACADS	Q07417	2	2	24147	27416.5	18813.5	18284.5	20452.5	25557	19298.5	20238	20471	6643	0.381616153		0.41837325	21822.8	18441.5	Down	-0.242880213338735	Short-chain specific acyl-CoA dehydrogenase, mitochondrial OS=Mus musculus GN=Acads PE=1 SV=2
UBE2L3	P68037	1	1	33603	46243	25410	57942	50079	44453	41502	NA	46328	75650	0.381776085		0.41819128	42655.4	51983.25	Up	0.285318441699321	Ubiquitin-conjugating enzyme E2 L3 OS=Mus musculus GN=Ube2l3 PE=2 SV=1
HIBCH	Q8QZS1	1	1	113187	100774	211395	53561	101383	95336	106050	337262	91460	204448	0.381902898		0.418047046	116060	166911.2	Up	0.524209931016117	3-hydroxyisobutyryl-CoA hydrolase, mitochondrial OS=Mus musculus GN=Hibch PE=1 SV=1
MRPL30	Q9D7N6	3	3	90039.33	86193.33	149535.67	41059.67	70088.33	51702.67	68919.33	192041.33	106851.33	170335	0.382267381		0.417632759	87383.266	117969.93	Up	0.432990261021442	39S ribosomal protein L30, mitochondrial OS=Mus musculus GN=Mrpl30 PE=2 SV=1
TRIM28	Q62318	4	4	33510.5	24187	30413.75	19714.25	28338.5	24576.25	25200.75	31140.75	25585.75	8915.75	0.382353103		0.417535381	27232.8	23083.85	Down	-0.238461462411052	Transcription intermediary factor 1-beta OS=Mus musculus GN=Trim28 PE=1 SV=3
AHCYL1	Q80SW1	22	22	51822.28	67982	59680	112328.11	63193.44	89899.22	80289.06	82129.22	72336.17	83641.94	0.382429081		0.41744909	71001.166	81659.122	Up	0.201771338636701	Putative adenosylhomocysteinase 2 OS=Mus musculus GN=Ahcyl1 PE=1 SV=1
VAPB	Q9QY76	6	6	36838.17	44017.67	78762	95633.33	48478.17	58558.33	66776.83	80632.5	53086.5	121881.5	0.382675754		0.417169054	60745.868	76187.132	Up	0.326761068856472	Vesicle-associated membrane protein-associated protein B OS=Mus musculus GN=Vapb PE=2 SV=3
PRKACB	P68181-3	4	4	77163.5	98304.75	77750.75	105668.5	97535	98811.5	91876	30447.25	110511.75	43850.5	0.383067021		0.416725236	91284.5	75099.4	Down	-0.281568531593852	Isoform 3 of cAMP-dependent protein kinase catalytic subunit beta OS=Mus musculus GN=Prkacb
NDUFA4	Q62425	12	12	104270	95784.92	115672.17	67888.5	79532	66258.58	84686.75	129596.75	102769.67	190558.92	0.383180832		0.416596224	92629.518	114774.13	Up	0.309253634966703	NADH dehydrogenase [ubiquinone] 1 alpha subcomplex subunit 4 OS=Mus musculus GN=Ndufa4 PE=1 SV=2
RPL4	Q9D8E6	26	26	187672.27	218640.27	326455.04	123990.42	189824.42	147250.65	199752.35	343125.19	232828	375401.23	0.383684261		0.416026016	209316.48	259671.48	Up	0.311001661626092	60S ribosomal protein L4 OS=Mus musculus GN=Rpl4 PE=1 SV=3
MCTS1	Q9DB27	5	5	57331.6	48589	105632.2	43479.4	43764.4	43283.8	41292.6	107351	56667.8	227461.2	0.383820387		0.415871961	59759.32	95211.28	Up	0.671968772758826	Malignant T-cell-amplified sequence 1 OS=Mus musculus GN=Mcts1 PE=2 SV=1
ACOT13	Q9CQR4	3	3	132525	114338	139692.67	83259	109548	103373.33	105915	165840	109048.33	194297	0.384125688		0.415526649	115872.53	135694.73	Up	0.227826077176015	Acyl-coenzyme A thioesterase 13 OS=Mus musculus GN=Acot13 PE=1 SV=1
PDHA1	P35486	47	47	184505.98	196507.64	128429.06	210390.43	185062.96	197106.17	191998.57	148783.85	162828.38	102300.32	0.384341107		0.415283163	180979.21	160603.46	Down	-0.172321052736542	Pyruvate dehydrogenase E1 component subunit alpha, somatic form, mitochondrial OS=Mus musculus GN=Pdha1 PE=1 SV=1
TIMM10	P62073	9	9	171926.89	149505	138647.67	136471.56	162305.78	160650.78	142979.67	158332.33	194005.89	153428	0.384810792		0.414752756	151771.38	161879.33	Up	0.0930190557102108	Mitochondrial import inner membrane translocase subunit Tim10 OS=Mus musculus GN=Timm10 PE=1 SV=1
FSIP2	A2ARZ3	1	1	313690	220251	283022	306793	267093	365035	261299	262397	345252	NA	0.385015941		0.414521289	278169.8	308495.75	Up	0.149284814560487	Fibrous sheath-interacting protein 2 OS=Mus musculus GN=Fsip2 PE=4 SV=3
CHCHD7	Q8K2Q5	2	2	11777	12904	16157.5	6656	10481.5	9560	9527	24261	17178.5	12354.5	0.385627646		0.413831838	11595.2	14576.2	Up	0.330086955055156	Coiled-coil-helix-coiled-coil-helix domain-containing protein 7 OS=Mus musculus GN=Chchd7 PE=3 SV=1
GGA1	Q8R0H9	5	5	14923.33	20989.33	1652.67	24511	21444.67	23589.33	29001	24979.33	16427.67	12741.33	0.385645934		0.413811243	16704.2	21347.732	Up	0.353871914355775	ADP-ribosylation factor-binding protein GGA1 OS=Mus musculus GN=Gga1 PE=1 SV=1
QK	Q9QYS9-5	3	3	113667.33	128602.33	90493.33	110239.67	118246.67	118149.33	92855.67	97709.67	132320.33	40329	0.385668757		0.413785541	112249.87	96272.8	Down	-0.221513567293594	Isoform 5 of Protein quaking OS=Mus musculus GN=Qki
MYRIP	Q8K3I4	2	2	4855.5	5874	5120.5	2882.5	4194	3903.5	5792	NA	8679	NA	0.386219162		0.413166183	4585.3	6124.8333	Up	0.417654459218844	Rab effector MyRIP OS=Mus musculus GN=Myrip PE=1 SV=1
ZRANB2	Q9R020	2	2	15011.5	11594	23743.5	4778	11080.5	8859.5	9637	22412.5	22110.5	24158	0.386263627		0.413116186	13241.5	17435.5	Up	0.396961176842332	Zinc finger Ran-binding domain-containing protein 2 OS=Mus musculus GN=Zranb2 PE=1 SV=2
ELAVL1	P70372	2	2	39194	33629.5	72354	42744.5	43230.5	39247	51364.5	75052.5	48195.5	58731.5	0.386407861		0.412954047	46230.5	54518.2	Up	0.23789296425254	ELAV-like protein 1 OS=Mus musculus GN=Elavl1 PE=1 SV=2
IQSEC3	Q3TES0	3	3	1930	3079	626.33	6169	3287	6848	4404.33	2733.67	2967.67	NA	0.386426675		0.412932902	3018.266	4238.4175	Up	0.489805749289894	IQ motif and SEC7 domain-containing protein 3 OS=Mus musculus GN=Iqsec3 PE=2 SV=1
ANXA7	Q07076	2	2	15229	16048.5	31994.5	10451.5	14804.5	17039.5	11986.5	35792.5	13644.5	46110.5	0.386518377		0.412829853	17705.6	24914.7	Up	0.492791468507586	Annexin A7 OS=Mus musculus GN=Anxa7 PE=2 SV=2
MAG	P20917-2	1	1	83785	165359	117210	179412	127543	163198	139885	82642	67384	107371	0.386907859		0.412392449	134661.8	112096	Down	-0.264605855949558	Isoform S-MAG of Myelin-associated glycoprotein OS=Mus musculus GN=Mag
MRPL36	Q99N90	1	1	32146	27508	31975	12249	33791	26976	25576	42671	44614	24899	0.386939114		0.412357367	27533.8	32947.2	Up	0.258952130609227	39S ribosomal protein L36, mitochondrial OS=Mus musculus GN=Mrpl36 PE=2 SV=1
DLG2	Q91XM9-5	2	2	12311.5	25333	18945.5	36057.5	24461.5	41473	23952	NA	24968	NA	0.387132114		0.412140801	23421.8	30131	Up	0.363396601133167	Isoform 5 of Disks large homolog 2 OS=Mus musculus GN=Dlg2
ANKS1	P59672	1	1	NA	4637	NA	14132	4888	12968	8378	NA	12889	NA	0.387200392		0.412064211	7885.6667	11411.667	Up	0.533204878715932	Ankyrin repeat and SAM domain-containing protein 1A OS=Mus musculus GN=Anks1a PE=1 SV=3
NPTX1	Q62443	5	5	6664.4	10316.6	3923.8	18301	10257.6	13012.8	14424	NA	9967.4	NA	0.38746039		0.411772689	9892.68	12468.067	Up	0.333804458399381	Neuronal pentraxin-1 OS=Mus musculus GN=Nptx1 PE=2 SV=1
CLTB	Q6IRU5-3	6	6	37889.83	50626.33	30069.5	102917	52451	74889.83	48740.67	28609.5	39547.83	2751	0.387618108		0.411595943	54790.732	38907.766	Down	-0.493873731816885	Isoform 3 of Clathrin light chain B OS=Mus musculus GN=Cltb
TRAPPC13	Q3TIR1-3	1	1	69104	52747	76746	40100	72162	66495	62201	66631	84467	NA	0.388546138		0.410557403	62171.8	69948.5	Up	0.170032771632406	Isoform 3 of Trafficking protein particle complex subunit 13 OS=Mus musculus GN=Trappc13
KCNB1	Q03717	3	3	12093.33	18528.67	6029.67	42775	24282.33	41218	33293.67	23839.67	20157.67	19925.67	0.388593799		0.410504134	20741.8	27686.936	Up	0.416664306867804	Potassium voltage-gated channel subfamily B member 1 OS=Mus musculus GN=Kcnb1 PE=1 SV=2
OCRL	Q6NVF0	1	1	52353	82087	32231	73808	67122	59815	70860	70467	54440	115878	0.388640723		0.410451695	61520.2	74292	Up	0.272146671656821	Inositol polyphosphate 5-phosphatase OCRL-1 OS=Mus musculus GN=Ocrl PE=1 SV=1
KIF7	B7ZNG0	1	1	23200	27872	24125	33994	32630	28856	32798	36690	26572	NA	0.389220415		0.409804388	28364.2	31229	Up	0.138815198819583	Kinesin-like protein KIF7 OS=Mus musculus GN=Kif7 PE=1 SV=1
EIF3J2	Q66JS6	5	5	14688.67	15160.33	21967.33	11637	14854	13751	15563.33	17452.67	15725.33	43667.67	0.390126953		0.408794044	15661.466	21232	Up	0.439021012620946	Eukaryotic translation initiation factor 3 subunit J-B OS=Mus musculus GN=Eif3j2 PE=1 SV=1

PIK3R2	O08908	1	1	19682	23748	15163	10193	12386	11642	14927	NA	14634	NA	0.390569859	0.408301275	16234.4	13734.333	Down	-0.241267182209551	Phosphatidylinositol 3-kinase regulatory subunit beta OS=Mus musculus GN=Pik3r2 PE=1 SV=2
CARMIL3	Q3UFQ8	1	1	NA	3624	NA	4622	5196	6931	3008	NA	11791	NA	0.390649112	0.408213158	4480.6667	7243.3333	Up	0.692940365612618	Leucine-rich repeat-containing protein 16B OS=Mus musculus GN=Lrrc16b PE=2 SV=2
RNF181	Q9CY62	1	1	36689	42854	17484	60730	59709	64270	44784	44695	54436	NA	0.390693314	0.408164021	43493.2	52046.25	Up	0.259004360625067	E3 ubiquitin-protein ligase RNF181 OS=Mus musculus GN=Rnf181 PE=2 SV=1
SEPT6	Q9R1T4	13	13	80925.77	86122.77	81559.08	97632.62	84595	95826.38	91372.31	76547.08	83511.15	125628.54	0.391015426	0.407806109	86167.048	94577.092	Up	0.134354523966029	Septin-6 OS=Mus musculus GN=Sept6 PE=1 SV=4
TUBB4A	Q9D6F9	83	83	66908.06	70013	81884.01	64053	58033.47	61415.73	67898.06	90672.58	60443.19	106968.71	0.391349545	0.407435166	68178.308	77479.654	Up	0.184504715170176	Tubulin beta-4A chain OS=Mus musculus GN=Tubb4a PE=1 SV=3
MYDGF	Q9CPT4	5	5	64225	59863	84220.2	46591.4	57967	60295.2	52288.2	101346.6	77528	68976.4	0.391416329	0.40736106	62573.32	72086.88	Up	0.204189056870318	UPF0556 protein C19orf10 homolog OS=Mus musculus GN=D17Wsu104e PE=2 SV=1
RPS12	P63323	10	10	57635.3	63826.4	56377.5	109214.3	61903.3	69781.1	71764.8	30378.5	83452.9	21840.5	0.391501284	0.407266809	69791.36	55443.56	Down	-0.332028550273464	40S ribosomal protein S12 OS=Mus musculus GN=Rps12 PE=1 SV=2
ATP5L	Q9CPQ8	6	6	145227.5	155705.17	192835	120223.67	142198.83	150382.83	151324.5	191616.67	141490.67	196091	0.391890043	0.406835771	151238.03	166181.13	Up	0.135935606535421	ATP synthase subunit g, mitochondrial OS=Mus musculus GN=Atp5l PE=1 SV=1
ARPC5	Q9CPW4	10	10	68142.44	73752.22	88553.33	72661.56	65450.33	60838.33	75293.56	89971.56	74052.33	115430.33	0.392464754	0.406199339	73711.976	83117.222	Up	0.173248402993202	Actin-related protein 2/3 complex subunit 5 OS=Mus musculus GN=Arpc5 PE=2 SV=3
TNR	Q8BYI9	21	21	14998.56	17112.25	17421.88	25740.62	17916.69	19979.88	20044.81	13666.94	21588	42317.31	0.392470065	0.406193463	18638	23519.388	Up	0.335603464090185	Tenascin-R OS=Mus musculus GN=Tnr PE=1 SV=2
SMPD1	Q04519	1	1	2126	4230	2926	2023	2852	3246	1976	NA	1214	NA	0.392619098	0.406028579	2831.4	2145.3333	Down	-0.400313751968347	Sphingomyelin phosphodiesterase OS=Mus musculus GN=Smpd1 PE=2 SV=2
CYFIP2	Q5SQX6	1	1	17558	76338	NA	123290	59435	104324	110594	74180	78236	NA	0.392672845	0.405969131	69155.25	91833.5	Up	0.409181751902081	Cytoplasmic FMR1-interacting protein 2 OS=Mus musculus GN=Cyfip2 PE=1 SV=2
RUNDC3B	Q6PDC0	1	1	17369	26988	19933	24789	25779	28189	21574	65130	19664	NA	0.392842152	0.405781919	22971.6	33639.25	Up	0.5502941948242	RUN domain-containing protein 3B OS=Mus musculus GN=Rundc3b PE=2 SV=1
SUMO1	P63166	1	1	159627	110982	30719	65378	135232	151229	142387	65260	158594	NA	0.393029047	0.405575351	100387.6	129367.5	Up	0.365894148867249	Small ubiquitin-related modifier 1 OS=Mus musculus GN=Sumo1 PE=1 SV=1
CMAS	Q99KK2	2	2	92986	49208	50103.5	88751	76723	88928.5	69364	45381	52484	44836	0.393367426	0.405201606	71554.3	60198.7	Down	-0.249306135088829	N-acylneuraminate cytidylyltransferase OS=Mus musculus GN=Cmas PE=1 SV=2
PSMA6	Q9QUM9	11	11	57139.82	60197.55	62467.27	64510.45	59753.09	64862.91	66742.64	65242.64	69164	52601.64	0.393556272	0.404993162	60813.636	63722.766	Up	0.0674140412475368	Proteasome subunit alpha type-6 OS=Mus musculus GN=Psma6 PE=1 SV=1
CPLX3	Q8R1B5	2	2	39067.5	37637	35770	37311	31112	35988	48178.5	29427	52869	NA	0.393609631	0.404934283	36179.5	41615.625	Up	0.201952834192551	Complexin-3 OS=Mus musculus GN=Cplx3 PE=1 SV=1
TBCC	Q8VCN9	1	1	38038	37611	100714	23112	36157	33743	40172	76032	47128	170141	0.393633767	0.404907653	47126.4	73443.2	Up	0.640093442260829	Tubulin-specific chaperone C OS=Mus musculus GN=Tbcc PE=2 SV=1
KCMF1	Q80UY2	5	5	41114	35071.2	32722	30493.6	38221.6	36575.2	41321.8	20378.4	39948	6470.8	0.393903378	0.404610295	35524.48	28938.84	Down	-0.295806438484585	E3 ubiquitin-protein ligase KCMF1 OS=Mus musculus GN=Kcmf1 PE=2 SV=1
MPP5	Q9JLB2	1	1	12468	9445	9098	11002	13705	9584	9624	6961	12890	NA	0.394129953	0.404360559	11143.6	9764.75	Down	-0.190560364985844	MAGUK p55 subfamily member 5 OS=Mus musculus GN=Mpp5 PE=1 SV=1
SPATA2L	Q8BNN1	2	2	11885.5	23506	24801	20269	24427	20101	23465	7316	17425.5	NA	0.394721584	0.403709125	20977.7	17076.875	Down	-0.296812517485141	Spermatogenesis-associated protein 2-like protein OS=Mus musculus GN=Spata2l PE=2 SV=1
PYCR1	Q922W5	3	3	47870.67	44613.33	50720.33	30290	39827.33	42947.33	48631.33	27221	60542	74048.67	0.395261798	0.403115158	42664.332	50678.066	Up	0.248331008965649	Pyrroline-5-carboxylate reductase 1, mitochondrial OS=Mus musculus GN=Pycr1 PE=2 SV=1
MRPL15	Q9CPR5	2	2	25326	27713	52783	22245.5	24508.5	23780	30306.5	38097.5	42039	51631.5	0.395345565	0.403023128	30515.2	37170.9	Up	0.284645573834736	39S ribosomal protein L15, mitochondrial OS=Mus musculus GN=Mrpl15 PE=2 SV=1
SEZ6L2	Q4V9Z5-3	3	3	9389.67	20936	3571.67	40473.67	17964.67	34648.67	38596.67	15842.33	16283	NA	0.39549883	0.402854797	18467.136	26342.668	Up	0.512441301147389	Isoform 3 of Seizure 6-like protein 2 OS=Mus musculus GN=Sez6l2
FAM185A	Q7TPD2-2	2	2	52353	39499.5	80867	24932.5	50482.5	38941.5	49686	51652.5	67192.5	112030.5	0.395603133	0.402740277	49626.9	63900.6	Up	0.364707139656082	Isoform 2 of Protein FAM185A OS=Mus musculus GN=Fam185a
ATPIF1	O35143	10	10	114951.9	102042.3	175724.4	179893	93770.3	147596.4	120953.6	204703.3	124565.5	178561.7	0.396070523	0.402227478	133276.38	155276.1	Up	0.220414667656834	ATPase inhibitor, mitochondrial OS=Mus musculus GN=Atpif1 PE=1 SV=2
CTSL	P06797	2	2	29255	20067	26765.5	15633	21976.5	20519	21830.5	30112.5	32432	NA	0.396254129	0.402026199	22739.4	26223.5	Up	0.205666064241304	Cathepsin L1 OS=Mus musculus GN=Ctsl PE=1 SV=2
LGALSL	Q8VED9	3	3	56302	47749.33	112373.33	58386.67	62787.33	53947.67	50663	135531.67	74230	115646	0.39644265	0.40181963	67519.732	86003.668	Up	0.349089013426818	Galectin-related protein OS=Mus musculus GN=Lgalsl PE=2 SV=1
PFKM	P47857	13	13	58590.38	62412.77	83211.08	87709.77	75337.54	78639.38	89690.31	64775.62	68813.15	110182.77	0.396739616	0.401494431	73452.308	82420.246	Up	0.166190946146315	6-phosphofructokinase, muscle type OS=Mus musculus GN=Pfkm PE=1 SV=3
OS9	Q8K2C7	8	8	14936	15856.57	13030.43	10103	16668.43	15276.14	18828.86	14359.14	25460.29	9803.71	0.396863238	0.401359128	14118.886	16745.628	Up	0.246158219050926	Protein OS-9 OS=Mus musculus GN=Os9 PE=1 SV=2
TRP53I11	Q4QQM4	1	1	8335	13688	10907	22436	7820	22700	17868	NA	10142	NA	0.397782685	0.400354125	12637.2	16903.333	Up	0.419630929915105	Tumor protein p53-inducible protein 11 OS=Mus musculus GN=Trp53i11 PE=2 SV=1
SLC20A2	Q80UP8	1	1	3470	4393	2690	13315	5178	8580	9146	5141	8131	NA	0.398054771	0.400057166	5809.2	7749.5	Up	0.415763730252284	Sodium-dependent phosphate transporter 2 OS=Mus musculus GN=Slc20a2 PE=1 SV=2
HAGHL	Q9DB32	2	2	14097	12359.5	26995.5	15981.5	18018	19410.5	17729.5	NA	23963.5	NA	0.398059407	0.400052108	17490.3	20367.833	Up	0.219737483958464	Hydroxyacylglutathione hydrolase-like protein OS=Mus musculus GN=Haghl PE=2 SV=1
RPL38	Q9JJI8	2	2	95192.5	83686	47886	133574	64151	95468	102558.5	106120.5	91520	NA	0.398179369	0.399921246	84897.9	98916.75	Up	0.220485971145091	60S ribosomal protein L38 OS=Mus musculus GN=Rpl38 PE=2 SV=3
UCHL3	Q9JKB1	3	3	41368.33	45506.33	50556.33	44001.67	40534	46485.67	37435.33	72963.67	50399	44001.67	0.398211047	0.399886696	44393.332	50257.068	Up	0.178983510665552	Ubiquitin carboxyl-terminal hydrolase isozyme L3 OS=Mus musculus GN=Uchl3 PE=1 SV=2
OPA1	P58281	23	23	45067.78	55913.3	35570.39	58645.7	51445.13	56592.52	52677.39	38792.13	42950.83	23799.48	0.398398721	0.399682064	49328.46	42962.47	Down	-0.199343308918662	Dynamin-like 120 kDa protein, mitochondrial OS=Mus musculus GN=Opa1 PE=1 SV=1
MRRF	Q9D6S7	7	7	26077.67	25231.33	22595.17	14773.83	23275.67	18474.83	20076.67	26588.33	27037.5	37159.33	0.399484987	0.398499537	22390.734	25867.332	Up	0.208229437155961	Ribosome-recycling factor, mitochondrial OS=Mus musculus GN=Mrrf PE=1 SV=1
SGIP1	Q8VD37-2	14	14	52919.38	49853.62	61451.15	44212.69	50420.54	43452.69	43035.23	51523.46	49319.62	55254.15	0.401039122	0.396813259	51771.476	48517.03	Down	-0.0936662121953676	Isoform 2 of SH3-containing GRB2-like protein 3-interacting protein 1 OS=Mus musculus GN=Sgip1
DCC	P70211-3	1	1	3770	15815	9008	20065	12922	19274	24508	NA	8691	NA	0.40186973	0.395914705	12316	17491	Up	0.506079001258139	Isoform B of Netrin receptor DCC OS=Mus musculus GN=Dcc
PAM16	Q9CQV1	4	4	47775.25	43419.5	68365	28746.75	42208.25	39531	41244	61553.25	54052.75	74633	0.402159047	0.395602157	46102.95	54202.8	Up	0.233508312359634	Mitochondrial import inner membrane translocase subunit TIM16 OS=Mus musculus GN=Pam16 PE=2 SV=1
NDUFB9	Q9CQJ8	24	24	47871.38	46788.29	41692.67	42730.33	48296.57	46461.86	42804.62	33776.9	49101.71	42012.81	0.402230331	0.395525183	45475.848	42831.58	Down	-0.0864256431271815	NADH dehydrogenase [ubiquinone] 1 beta subcomplex subunit 9 OS=Mus musculus GN=Ndufb9 PE=1 SV=3
RPL30	P62889	11	11	135978.36	147980.45	156027.36	123785.64	119598.91	130401.64	147374.09	190500.73	153418.18	122885.64	0.402296898	0.395453316	136674.14	148916.06	Up	0.123758972571362	60S ribosomal protein L30 OS=Mus musculus GN=Rpl30 PE=2 SV=2
MYEF2	Q8C854-2	2	2	44121	32127.5	60376.5	18824	36753	28692.5	31822	114926	30342.5	68817	0.402328925	0.395418743	38440.4	54920	Up	0.514708277016069	Isoform 4 of Myelin expression factor 2 OS=Mus musculus GN=Myef2
SEPT4	P28661-6	4	4	347778.5	317807.5	600417.5	216191.25	328230	285226.75	320272.25	553676.5	362567.25	805966.75	0.402544807	0.395185771	362084.95	465541.9	Up	0.362582807306234	Isoform 6 of Septin-4 OS=Mus musculus GN=Sept4
SYBU	Q8BHS8-3	1	1	12395	9222	27158	6417	6534	10400	10320	26881	22151	NA	0.403090241	0.394597716	12345.2	17438	Up	0.498284374711951	Isoform 3 of Syntabulin OS=Mus musculus GN=Sybu
NTMT1	Q8R2U4	1	1	1485	5367	NA	6728	4525	4224	3459	NA	2492	NA	0.403399456	0.394264691	4526.25	3391.6667	Down	-0.416321883043725	N-terminal Xaa-Pro-Lys N-methyltransferase 1 OS=Mus musculus GN=Ntmt1 PE=2 SV=3
SRRM1	Q52KI8	2	2	4453	3816.5	930.5	7191	1931.5	5231	3652.5	NA	5416.5	NA	0.403799469	0.393834257	3664.5	4766.6667	Up	0.379364376843546	Serine/arginine repetitive matrix protein 1 OS=Mus musculus GN=Srrm1 PE=1 SV=2
FHL1	P97447-2	1	1	51730	45200	49910	78103	48133	60141	51537	NA	92189	NA	0.403894731	0.393731812	54615.2	67955.667	Up	0.315291334388717	Isoform 2 of Four and a half LIM domains protein 1 OS=Mus musculus GN=Fhl1
GAS2L2	Q5SSG4	1	1	129540	162689	332698	39099	128824	63394	100948	368085	122236	829583	0.403935545	0.393687929	158570	296849.2	Up	0.90461037299648	GAS2-like protein 2 OS=Mus musculus GN=Gas2l2 PE=2 SV=1
UBA1	Q02053	22	22	16148.76	24340.33	19452.9	33483.05	21648.05	27736.24	28795.19	25852.29	24745.86	22277.86	0.403964426	0.393656879	23014.618	25881.488	Up	0.169370067639213	Ubiquitin-like modifier-activating enzyme 1 OS=Mus musculus GN=Uba1 PE=1 SV=1
CAPZB	P47757-4	19	19	67665.32	66631.11	59284.68	74742.47	76465.58	75358.58	83867.53	27952.84	70621.89	31377.63	0.404325179	0.393269213	68957.832	57835.694	Down	-0.253754273189872	Isoform 3 of F-actin-capping protein subunit beta OS=Mus musculus GN=Capzb
SEPT1	P42209	4	4	356070.75	325236.75	593796.5	289995.75	338006	305768.75	339630	569067.25	367402.5	805966.75	0.404460512	0.393123873	380621.15	477567.05	Up	0.327347570334739	Septin-1 OS=Mus musculus GN=Sept1 PE=2 SV=2
RPLP2	P99027	8	8	49269.88	72869.38	66652.12	73481.88	44739.25	60645.75	50351.25	61693.75	62074.25	38940.88	0.40452009	0.393059905	61402.502	54741.176	Down	-0.165671014715871	60S acidic ribosomal protein P2 OS=Mus musculus GN=Rplp2 PE=1 SV=3
SLC12A2	P55012	1	1	22522	29467	NA	55169	32089	41514	32257	NA	61752	NA	0.404981088	0.392565258	34811.75	45174.333	Up	0.375928969752544	Solute carrier family 12 member 2 OS=Mus musculus GN=Slc12a2 PE=1 SV=2
ATCAY	Q8BHE3	7	7	7907.43	14241.29	2985.86	26994	17632.57	26389.14	23983.71	11815.14	13245.71	NA	0.40532339	0.392198334	13952.23	18858.425	Up	0.43471346342059	Caytaxin OS=Mus musculus GN=Atcay PE=1 SV=1
HDHD2	Q3UGR5	6	6	27066.83	26652.83	27974.17	9767.67	24025.17	19777.67	20111.17	36174.33	23638.67	39399	0.405547995	0.391957741	23097.334	27820.168	Up	0.26840479324437	Haloacid dehalogenase-like hydrolase domain-containing protein 2 OS=Mus musculus GN=Hdhd2 PE=1 SV=2
PRKCE	P16054	6	6	18197.5	28975.83	14027	39143.5	27142	31991.83	36146	NA	23704.17	NA	0.406000435	0.391473501	25497.166	30614	Up	0.26385465751804	Protein kinase C epsilon type OS=Mus musculus GN=Prkce PE=1 SV=1
SORBS2	Q3UTJ2-7	4	4	30942.5	34906.25	39268	36233.25	40479.75	35245.75	36958.75	38612	43066	NA	0.406405819	0.391040083	36365.95	38470.625	Up	0.0811690032799979	Isoform 7 of Sorbin and SH3 domain-containing protein 2 OS=Mus musculus GN=Sorbs2
RGS14	P97492	1	1	25721	31999	21675	67010	38511	44054	29141	17953	21875	NA	0.407252663	0.390136068	36983.2	28255.75	Down	-0.388325576906962	Regulator of G-protein signaling 14 OS=Mus musculus GN=Rgs14 PE=1 SV=2
CCDC6	D3YZP9	11	11	6866.8	6961.4	7725.3	8095.5	7634.9	7259.1	7126.9	5847.8	8906.3	4856.9	0.407333309	0.390050075	7456.78	6799.4	Down	-0.133145333688043	Coiled-coil domain-containing protein 6 OS=Mus musculus GN=Ccdc6 PE=1 SV=1
UBE2D2B	Q6ZWY6	1	1	40536	60597	68649	57710	63622	59487	81694	64983	57801	56291	0.407459534	0.389915515	58222.8	64051.2	Up	0.137641377771253	Ubiquitin-conjugating enzyme E2 D2B OS=Mus musculus GN=Ube2d2b PE=2 SV=1
CCDC47	Q9D024	4	4	2294	3646.5	1428	3826	2681	3136	4750.5	NA	2611.5	NA	0.40755688	0.389811771	2775.1	3499.3333	Up	0.334540337291025	Coiled-coil domain-containing protein 47 OS=Mus musculus GN=Ccdc47 PE=2 SV=2
TWF1	Q91YR1	4	4	30336	30360.5	44893	24456.25	29478.75	28010.25	35626.25	27501.25	36745.75	69929.5	0.407666064	0.38969544	31904.9	39562.6	Up	0.310359228992485	Twinfilin-1 OS=Mus musculus GN=Twf1 PE=1 SV=2
AK4	Q9WUR9	9	9	62581.22	54831.33	60572.78	40246.89	60778.89	53628.78	53353.33	74293.11	54787.22	69433.89	0.408056176	0.389280045	55802.222	61099.266	Up	0.130832478544995	Adenylate kinase 4, mitochondrial OS=Mus musculus GN=Ak4 PE=1 SV=1
EIF2S2	Q99L45	3	3	41678	42778.33	69033.67	46112	38106	36554	48176.33	38770.67	65585.67	136594	0.408204101	0.389122636	47541.6	65136.134	Up	0.454267636930721	Eukaryotic translation initiation factor 2 subunit 2 OS=Mus musculus GN=Eif2s2 PE=1 SV=1
CAPZB	P47757	18	18	70005.61	68740.33	61454.72	75408.78	79060.89	77260.28	86604.67	29505.78	72502	33120.83	0.408863282	0.38842189	70934.066	59798.712	Down	-0.246364235792408	F-actin-capping protein subunit beta OS=Mus musculus GN=Capzb PE=1 SV=3
WNK2	Q3UH66-4	6	6	20807.6	20963.2	19636.4	25428.8	27649.4	30765	31582	26309.6	30620	12581.6	0.409286819	0.387972242	22897.08	26371.64	Up	0.203823665111056	Isoform 4 of Serine/threonine-protein kinase WNK2 OS=Mus musculus GN=Wnk2
ETL4	A2AQ25-2	15	15	41608.57	42148.21	42231.43	36687.29	47205.64	46087.86	47105.5	43597.36	59980.14	33509.14	0.409389951	0.387862821	41976.228	46056	Up	0.133816586044073	Isoform 2 of Sickle tail protein OS=Mus musculus GN=Skt
CCDC85B	Q6PDY0	1	1	713	999	NA	1155	1010	963	1000	NA	1877	NA	0.410134946	0.387073224	969.25	1280	Up	0.401203075136182	Coiled-coil domain-containing protein 85B OS=Mus musculus GN=Ccdc85b PE=1 SV=1
NMT1	O70310	4	4	51338	55801.75	56770	49706.5	50386.5	56166.5	58459.5	31198	62324	7857.25	0.410218147	0.386985132	52800.55	43201.05	Down	-0.289486580111727	Glycylpeptide N-tetradecanoyltransferase 1 OS=Mus musculus GN=Nmt1 PE=1 SV=1
DNAJA3	Q99M87-3	16	16	21233.07	28099.43	8430.5	32570.64	23004	26035.07	25123.71	11937.07	20818.93	3926.57	0.410491811	0.386695502	22667.528	17568.27	Down	-0.367654935159181	Isoform 3 of DnaJ homolog subfamily A member 3, mitochondrial OS=Mus musculus GN=Dnaja3
CPOX	P36552	2	2	36941.5	37610.5	31026	28837	27580.5	34446	31034	NA	47468	NA	0.411048291	0.386107153	32399.1	37649.333	Up	0.216670581251063	Coproporphyrinogen-III oxidase, mitochondrial OS=Mus musculus GN=Cpox PE=1 SV=2
FAM136A	Q9CR98	10	10	64125.56	54377.11	47734.22	66939.22	57895.33	61779.11	56771.11	40794.67	69728.11	18834	0.411239318	0.38590537	58214.288	49581.4	Down	-0.231574280312212	Protein FAM136A OS=Mus musculus GN=Fam136a PE=2 SV=1
CIAPIN1	Q8WTY4	2	2	20727.5	35116.5	9329.5	34734	25348.5	34357	45420	25432.5	20544	NA	0.411295414	0.385846133	25051.2	31438.375	Up	0.327646935988442	Anamorsin OS=Mus musculus GN=Ciapin1 PE=1 SV=1
NADK2	Q8C5H8-3	1	1	13778	7836	NA	15020	13962	13999	14407	NA	NA	NA	0.411413536	0.385721424	12649	14203	Up	0.167172358892059	Isoform 3 of NAD kinase 2, mitochondrial OS=Mus musculus GN=Nadk2
PABPC1	P29341	24	24	50133.83	56063.33	62178.21	61204.17	55418.54	53422.17	58889.58	55368.21	70676.33	63044.08	0.411901707	0.385206408	56999.616	60280.074	Up	0.0807289879576477	Polyadenylate-binding protein 1 OS=Mus musculus GN=Pabpc1 PE=1 SV=2
OAT	P29758	11	11	28800.9	30773.5	34378.6	23579	23519.4	29941.2	30236	32270.9	27266.1	31431.6	0.412174191	0.384919206	28210.28	30229.16	Up	0.0997199048835706	Ornithine aminotransferase, mitochondrial OS=Mus musculus GN=Oat PE=1 SV=1
CBR1	P48758	11	11	54793.18	64215.73	48217.73	85747.64	58941	72267.27	59053.18	33674.18	67744	33835.45	0.412405579	0.384675468	62383.056	53314.816	Down	-0.226617720115177	Carbonyl reductase [NADPH] 1 OS=Mus musculus GN=Cbr1 PE=1 SV=3
CARMIL1	Q6EDY6	1	1	403	785	NA	2943	2678	2220	NA	6332	1366	NA	0.41281485	0.384244688	1702.25	3306	Up	0.957643791440752	Leucine-rich repeat-containing protein 16A OS=Mus musculus GN=Lrrc16a PE=1 SV=2
EZR	P26040	8	8	26803.29	33004.57	13236.71	36290.57	25691.86	35394.86	31944.43	8308.29	24455.71	820.43	0.413241778	0.383795779	27005.4	20184.744	Down	-0.419982627978826	Ezrin OS=Mus musculus GN=Ezr PE=1 SV=3
ADPRHL2	Q8CG72-2	1	1	8349	8995	10919	21469	10365	13304	9443	2978	10976	NA	0.413299314	0.383735316	12019.4	9175.25	Down	-0.389545506499657	Isoform 2 of Poly(ADP-ribose) glycohydrolase ARH3 OS=Mus musculus GN=Adprhl2
DNAJC6	Q80TZ3-3	8	8	9689.38	12859.75	10498.88	17737.25	13142.5	19410.12	15677.38	17975.75	17659.25	5654.25	0.413986581	0.383013736	12785.552	15275.35	Up	0.256690988053742	Isoform 3 of Putative tyrosine-protein phosphatase auxilin OS=Mus musculus GN=Dnajc6
EEF1B2	O70251	14	14	57034.79	58687.64	85701.07	27437.86	52758.79	38497.93	54749.86	78111	66163.14	109530.07	0.414001725	0.38299785	56324.03	69410.4	Up	0.301401280081595	Elongation factor 1-beta OS=Mus musculus GN=Eef1b PE=1 SV=5
RBM8A	Q9CWZ3	2	2	34424	29718	32218	22682.5	25846.5	32485.5	25168.5	48747	41627.5	21267.5	0.414020131	0.382978542	28977.8	33859.2	Up	0.224599817335752	RNA-binding protein 8A OS=Mus musculus GN=Rbm8a PE=1 SV=3
NOP16	Q9CPT5	1	1	9198	12181	6317	14500	10486	14129	9713	10592	13904	NA	0.414108747	0.382885596	10536.4	12084.5	Up	0.197775760687207	Nucleolar protein 16 OS=Mus musculus GN=Nop16 PE=2 SV=1
TOLLIP	Q9QZ06	9	9	23473.11	22581.11	17889.22	28740.22	24471.11	26567.78	26163.11	21927.33	30698.22	22069.78	0.414369269	0.382612461	23430.954	25485.244	Up	0.121246470652035	Toll-interacting protein OS=Mus musculus GN=Tollip PE=1 SV=1
ARAF	P04627	2	2	10885.5	12400.5	11182.5	17479	13678.5	16812.5	12560.5	10665.5	24396.5	NA	0.414480454	0.382495945	13125.2	16108.75	Up	0.295505142209726	Serine/threonine-protein kinase A-Raf OS=Mus musculus GN=Araf PE=2 SV=2
GPT2	Q8BGT5	1	1	2546	1116	3275	6177	3110	4770	3390	3035	5187	NA	0.415019836	0.381931146	3244.8	4095.5	Up	0.335910036899748	Alanine aminotransferase 2 OS=Mus musculus GN=Gpt2 PE=1 SV=1
GBE1	Q9D6Y9	2	2	11915.5	12093	9738	23924.5	14047	20437	19369.5	11865.5	16592.5	NA	0.415124974	0.381821139	14343.6	17066.125	Up	0.250728358675916	1,4-alpha-glucan-branching enzyme OS=Mus musculus GN=Gbe1 PE=2 SV=1
KCNT1	Q6ZPR4	1	1	NA	1185	NA	2870	1597	2000	4704	NA	1899	NA	0.415357646	0.38157779	1884	2867.6667	Up	0.606078372096326	Potassium channel subfamily T member 1 OS=Mus musculus GN=Kcnt1 PE=2 SV=2
PDLIM5	Q8CI51-3	1	1	21369	20954	7010	18178	25960	24316	24138	14640	25805	NA	0.415366964	0.381568048	18694.2	22224.75	Up	0.24957645687888	Isoform 3 of PDZ and LIM domain protein 5 OS=Mus musculus GN=Pdlim5
HIST1H1A	P43275	3	3	277093.67	143502	156125	47914	178426	100943	103160.67	190711	114589	116761.33	0.415952308	0.380956461	160612.13	125233	Down	-0.358966115132049	Histone H1.1 OS=Mus musculus GN=Hist1h1a PE=1 SV=2
STARD10	Q9JMD3	2	2	3739	3806.5	NA	8025.5	3706.5	6568.5	4798.5	NA	5880.5	6174	0.415996278	0.380910555	4819.375	5855.375	Up	0.280915506370083	PCTP-like protein OS=Mus musculus GN=Stard10 PE=1 SV=1
MTFR1L	Q9CWE0	9	9	49653.22	46562.67	47788.78	21948.22	47265.67	38020.33	43306.11	38974.89	45448.44	15325	0.416013458	0.38089262	42643.712	36214.954	Down	-0.235747482794347	Mitochondrial fission regulator 1-like OS=Mus musculus GN=Mtfr1l PE=1 SV=1
HBB-B2	P02089	2	2	29716.5	21895	31774.5	16932	27027.5	17449.5	16639.5	4998	20194	40322	0.416266549	0.380628487	25469.1	19920.6	Down	-0.354486878666576	Hemoglobin subunit beta-2 OS=Mus musculus GN=Hbb-b2 PE=1 SV=2
UBQLN1	Q8R317-2	1	1	18332	16888	21364	3899	23373	11761	17018	25640	32894	NA	0.41641808	0.380470422	16771.2	21828.25	Up	0.380210552972593	Isoform 2 of Ubiquilin-1 OS=Mus musculus GN=Ubqln1
HNRNPU	Q8VEK3	9	9	197306.44	144711.22	150058.44	91565.22	142714.67	119451.11	129923.11	143631.33	114596.78	140287.78	0.416521677	0.380362391	145271.2	129578.02	Down	-0.164927656875836	Heterogeneous nuclear ribonucleoprotein U OS=Mus musculus GN=Hnrnpu PE=1 SV=1
CSDE1	Q91W50	6	6	31612.83	36194.83	34549.17	66712	35886.17	48269	40388.17	31067.33	33747.5	14869.17	0.416685895	0.3801912	40991	33668.234	Down	-0.283919136349812	Cold shock domain-containing protein E1 OS=Mus musculus GN=Csde1 PE=2 SV=1
CDC37	Q61081	8	8	25513.75	30986.12	31149.38	37298.75	35150	32908.75	38214	17515.5	25468	28484.5	0.416695978	0.380180691	32019.6	28518.15	Down	-0.167074889524358	Hsp90 co-chaperone Cdc37 OS=Mus musculus GN=Cdc37 PE=2 SV=1
RABL6	Q5U3K5	7	7	13472	15494.14	12605.86	19068.57	13158.57	20335.43	18702.43	11937.71	15772.29	NA	0.416927465	0.379939495	14759.828	16686.965	Up	0.177045673956881	Rab-like protein 6 OS=Mus musculus GN=Rabl6 PE=1 SV=2
NUDCD3	Q8R1N4-4	3	3	19911	21081.33	20549.67	12205.67	20467	20701.67	21374.67	16557.33	15715	5827.67	0.417483397	0.379360792	18842.934	16035.268	Down	-0.232775155966307	Isoform 4 of NudC domain-containing protein 3 OS=Mus musculus GN=Nudcd3
EIF4G3	Q80XI3-4	5	5	4876.25	8002	5529.5	19369.25	6847.25	21848.75	18353	2931.5	10146.75	NA	0.41895434	0.377833306	8924.85	13320	Up	0.577694255334036	Isoform 4 of Eukaryotic translation initiation factor 4 gamma 3 OS=Mus musculus GN=Eif4g3

MAP9	Q3TRR0	3	3	29260.5	26509	21469	13574	23918	19722.5	27020.5	NA	44927.5	22459	0.419700117	0.377060909	22946.1	28532.375	Up	0.31435087114605	Microtubule-associated protein 9 OS=Mus musculus GN=Map9 PE=2 SV=2
RPL10A	P53026	9	9	53052.67	94106.44	76862.11	118320.22	80989.22	100251	122799.44	86820.67	93832.56	76260.44	0.419726906	0.37703319	84666.132	95992.822	Up	0.181141549717822	60S ribosomal protein L10a OS=Mus musculus GN=Rpl10a PE=1 SV=3
PRKCG	P63318	20	20	36464.2	52151.1	41322	70036.6	48158.85	62471.5	66837.55	47775.45	51138.45	49589.15	0.419764493	0.376994301	49626.55	55562.42	Up	0.162997273660253	Protein kinase C gamma type OS=Mus musculus GN=Prkcg PE=1 SV=1
FHL1	P97447	6	6	44688.5	42720	45306.83	50261.33	48003	47218.33	51191.67	10248.17	63945	6110.83	0.420218011	0.376525337	46195.932	35742.8	Down	-0.370113158074742	Four and a half LIM domains protein 1 OS=Mus musculus GN=Fhl1 PE=1 SV=3
DCTN2	Q99KJ8	29	29	55639.41	63893.31	46513.9	69887.1	52754.76	61767.07	62699.69	41633.83	59253.79	30622.86	0.420299578	0.376441046	57737.696	51195.448	Down	-0.173497998628397	Dynactin subunit 2 OS=Mus musculus GN=Dctn2 PE=1 SV=3
ATP1B2	P14231	6	6	9119.33	13077	8591.17	21831	11196	18296.83	16280.5	11668.17	14408.67	NA	0.420701952	0.376025473	12762.9	15163.543	Up	0.248650657568198	Sodium/potassium-transporting ATPase subunit beta-2 OS=Mus musculus GN=Atp1b2 PE=1 SV=2
PTGR2	Q8VDQ1	2	2	13201	17716	14467.5	20064	16599	19729	21361	18675.5	29096	8526	0.421166862	0.375545807	16409.5	19477.5	Up	0.247277234356912	Prostaglandin reductase 2 OS=Mus musculus GN=Ptgr2 PE=1 SV=2
TUBAL3	Q3UX10	9	9	490228.89	617653.11	664621.89	561730.67	492318.78	533588.22	642698.89	652171.44	502597.78	719768.78	0.421243919	0.375466356	565310.67	610165.02	Up	0.110155556702346	Tubulin alpha chain-like 3 OS=Mus musculus GN=Tubal3 PE=2 SV=2
UQCRB	Q9D855	27	27	284065.85	243439.22	201593.3	236289.19	270187.04	264562.11	233559.81	167574.67	299973.41	106612.33	0.421260548	0.375449212	247114.92	214456.47	Down	-0.204497303748971	Cytochrome b-c1 complex subunit 7 OS=Mus musculus GN=Uqcrb PE=1 SV=3
METAP1	Q8BP48	5	5	9140.25	9760.5	13718	19956	15822.75	16430.5	22973.75	NA	11920.25	NA	0.421266669	0.375442901	13679.5	17108.167	Up	0.322669667860014	Methionine aminopeptidase 1 OS=Mus musculus GN=Metap1 PE=2 SV=1
BTF3L4	Q9CQH7	6	6	39779.67	39590.5	30874.67	49137	38023	47652.33	48174.33	71285.67	58472.17	14875.67	0.42140902	0.375296173	39480.968	48092.034	Up	0.284640582282903	Transcription factor BTF3 homolog 4 OS=Mus musculus GN=Btf3l4 PE=2 SV=1
TALDO1	Q93092	1	1	66621	80607	109976	16874	98043	45702	72197	189979	138874	61197	0.421409029	0.375296164	74424.2	101589.8	Up	0.448911843491344	Transaldolase OS=Mus musculus GN=Taldo1 PE=1 SV=2
NCKAP1	P28660	4	4	55944.25	69744	45240.75	60551.25	80983.25	81630.5	93112.25	43581.75	101262	49289.25	0.422561491	0.374110083	62492.7	73775.15	Up	0.239447275802573	Nck-associated protein 1 OS=Mus musculus GN=Nckap1 PE=1 SV=2
RANBP9	P69566-2	1	1	9503	29918	20340	20801	13493	15607	14092	28872	13141	82864	0.422709728	0.373957758	18811	30915.2	Up	0.716739793746019	Isoform 2 of Ran-binding protein 9 OS=Mus musculus GN=Ranbp9
MRPL37	Q921S7	1	1	31835	51619	23461	34834	24402	41610	43152	24530	48286	NA	0.423270847	0.373381642	33230.2	39394.5	Up	0.245499248092485	39S ribosomal protein L37, mitochondrial OS=Mus musculus GN=Mrpl37 PE=2 SV=1
STX2	Q00262	1	1	17572	34272	4502	82919	36515	70619	52682	32732	46517	37020	0.424410588	0.37221379	35156	47914	Up	0.446676326857861	Syntaxin-2 OS=Mus musculus GN=Stx2 PE=1 SV=1
PDHB	Q9D051	48	48	74463.52	67593.74	88608.8	60988.43	67975.5	69193.28	72619.43	79313.76	77891.43	83323.48	0.424488681	0.372133886	71925.998	76468.276	Up	0.0883480144253759	Pyruvate dehydrogenase E1 component subunit beta, mitochondrial OS=Mus musculus GN=Pdhb PE=1 SV=1
SEPT4	P28661-3	6	6	249142	226751.33	417176	163243.33	234664	208648.33	226246	378024.83	256568.17	537311.17	0.425595481	0.371002992	258195.33	321359.7	Up	0.315726101779308	Isoform 3 of Septin-4 OS=Mus musculus GN=Sept4
ADD2	Q9QYB8-3	5	5	28628	33892	30304.4	44483.4	23624.4	34186.4	35943.4	23187	29117.8	18294.4	0.425713631	0.370882444	32186.44	28145.8	Down	-0.193533360390781	Isoform 3 of Beta-adducin OS=Mus musculus GN=Add2
MPRIP	P97434-3	2	2	3454	4365.5	NA	13548	8986	10769.5	10204	NA	8343.5	NA	0.426810338	0.36976507	7588.375	9772.3333	Up	0.364912099456759	Isoform 3 of Myosin phosphatase Rho-interacting protein OS=Mus musculus GN=Mprip
HTRA2	Q9JIY5	5	5	16705.6	18800.8	12658.4	14372	21139.2	15756.8	19793.6	8772.2	18726.2	8755.4	0.42847084	0.368078729	16735.2	14360.84	Down	-0.220745654246443	Serine protease HTRA2, mitochondrial OS=Mus musculus GN=Htra2 PE=1 SV=2
CLTB	Q6IRU5	22	22	30038.18	31488.36	24299.68	56051.5	32915.32	42617.77	33628.55	25142.32	30246.68	12129.91	0.428491349	0.368057941	34958.608	28753.046	Down	-0.281932941638372	Clathrin light chain B OS=Mus musculus GN=Cltb PE=1 SV=1
HNRNPLL	Q921F4	4	4	25452	28598.33	48244	9701	29006.33	17009	32599	20004.33	38008.67	118080	0.428633624	0.367913764	28200.332	45140.2	Up	0.678700663162614	Heterogeneous nuclear ribonucleoprotein L-like OS=Mus musculus GN=Hnrnpll PE=1 SV=3
SHROOM2	A2ALU4	4	4	9980	13997	14160	7004	11642.75	13122.75	13133.75	8531.75	20003.5	NA	0.42929086	0.367248358	11356.75	13697.938	Up	0.270408649851667	Protein Shroom2 OS=Mus musculus GN=Shroom2 PE=1 SV=1
FNBP1	Q80TY0-4	3	3	43886.33	58599.67	47876.33	49546.67	44807.33	54903	51077	36526.67	51914	20483.67	0.429638906	0.366896398	48943.266	42980.868	Down	-0.187415757804882	Isoform 4 of Formin-binding protein 1 OS=Mus musculus GN=Fnbp1
WDR13	Q91V09	8	8	30672	40562.88	34957	32182.38	31584.88	34891.5	35768.62	16170.12	45427.12	4808.12	0.430065036	0.366465864	33991.828	27413.096	Down	-0.310322675190144	WD repeat-containing protein 13 OS=Mus musculus GN=Wdr13 PE=1 SV=1
U2AF1	Q9D883	2	2	88523.5	92045.5	70512	73339.5	71085	67536	77470.5	20597	92992	NA	0.430530216	0.365996363	79101.1	64648.875	Down	-0.291072492406469	Splicing factor U2AF 35 kDa subunit OS=Mus musculus GN=U2af1 PE=1 SV=4
DLG1	Q811D0-3	18	18	47106.94	47512.39	38559.94	54314.78	54071.83	54802.06	50907.22	29285.67	51996.67	25393.61	0.430654197	0.365871316	48313.176	42477.046	Down	-0.185733255777545	Isoform 3 of Disks large homolog 1 OS=Mus musculus GN=Dlg1
MYEF2	Q8C854	5	5	28902.8	20689	38247	13802.6	22325.6	18782.6	19931.4	62262	18175.8	47152.4	0.430858348	0.365665488	24793.4	33260.84	Up	0.423868477760662	Myelin expression factor 2 OS=Mus musculus GN=Myef2 PE=1 SV=1
TBCA	P48428	8	8	70082.25	68899	51556.12	38989.75	63105.5	42849.88	53860.25	37317.12	79690.75	37844	0.431341744	0.36517851	58526.524	50312.4	Down	-0.218176586205518	Tubulin-specific chaperone A OS=Mus musculus GN=Tbca PE=2 SV=3
CLTB	Q6IRU5-2	12	12	37951.17	42271.08	34078.75	81679.75	42137.92	60043.17	45100.08	34130.92	37377.42	14004.92	0.431657743	0.364860465	47623.734	38131.302	Down	-0.320704948419229	Isoform 2 of Clathrin light chain B OS=Mus musculus GN=Cltb
MRPL2	Q9D773	6	6	65453.67	71521.5	57477.17	42924	61036.67	57169.33	59312.33	105716.67	82488.67	44214.83	0.431827867	0.364689335	59682.602	69780.366	Up	0.22551072996313	39S ribosomal protein L2, mitochondrial OS=Mus musculus GN=Mrpl2 PE=2 SV=1
PFDN1	Q9CWM4	2	2	72401.5	91781	101024.5	154906.5	62604	124177.5	84254	172095.5	92852.5	102414	0.431974821	0.364541567	96543.5	115158.7	Up	0.2543723728609	Prefoldin subunit 1 OS=Mus musculus GN=Pfdn1 PE=2 SV=1
GPM6B	P35803-8	2	2	637	3205.5	1300	5990.5	3947	5706.5	6670	2031.5	2640	NA	0.432142108	0.364373414	3016	4262	Up	0.498894164514039	Isoform 8 of Neuronal membrane glycoprotein M6-b OS=Mus musculus GN=Gpm6b
MRPL13	Q9D1P0	2	2	32619.5	35390	32435	8809	29627.5	12085	22340.5	59458	27189.5	69013	0.432390514	0.364123842	27776.2	38017.2	Up	0.452803039222016	39S ribosomal protein L13, mitochondrial OS=Mus musculus GN=Mrpl13 PE=2 SV=1
ETL4	A2AQ25	16	16	39103.67	39338.33	40005.67	34570.2	44640.8	43508.73	44331	40690.87	55981.47	31275.2	0.432510969	0.364002874	39531.734	43157.454	Up	0.126598519824461	Sickle tail protein OS=Mus musculus GN=Skt PE=1 SV=1
ETL4	A2AQ25-4	15	15	39103.67	39338.33	40005.67	34570.2	44640.8	43508.73	44331	40690.87	55981.47	31275.2	0.432510969	0.364002874	39531.734	43157.454	Up	0.126598519824461	Isoform 4 of Sickle tail protein OS=Mus musculus GN=Skt
ARMC6	Q8BNU0	2	2	27679.5	26736	19086.5	40815.5	29206	42009.5	43932.5	22201	28175	NA	0.432517389	0.363996428	28704.7	34079.5	Up	0.247617191386777	Armadillo repeat-containing protein 6 OS=Mus musculus GN=Armc6 PE=2 SV=1
RPS29	P62274	2	2	165909	174668.5	264672.5	106205	146471.5	141719.5	159217.5	396492.5	224009.5	162096.5	0.43259503	0.363918474	171585.3	216707.1	Up	0.336820461360463	40S ribosomal protein S29 OS=Mus musculus GN=Rps29 PE=2 SV=2
DDX19A	Q61655	3	3	14970	18547.33	31965.67	19983.33	18414	17347.67	22930.67	23148.67	22579.33	34682.67	0.433408473	0.363102603	20776.066	24137.802	Up	0.216371807369113	ATP-dependent RNA helicase DDX19A OS=Mus musculus GN=Ddx19a PE=2 SV=2
DDX5	Q61656	12	12	50559.92	42867.5	36257	35690.17	41412.17	39045.42	40577	23479.92	47981.83	35630.83	0.43342272	0.363088327	41357.352	37343	Down	-0.14730598366582	Probable ATP-dependent RNA helicase DDX5 OS=Mus musculus GN=Ddx5 PE=1 SV=2
ANXA5	P48036	3	3	17064.33	19659	21057.67	30076	18168.67	26714.67	20832.67	38849.33	23424.33	15264.33	0.43343428	0.363076744	21205.134	25017.066	Up	0.238499000560082	Annexin A5 OS=Mus musculus GN=Anxa5 PE=1 SV=1
MAG	P20917	2	2	42514	83212.5	58605	93062	64807	83552.5	71447.5	41321	34345	58801	0.433971508	0.362538782	68440.1	57893.4	Down	-0.241442981239913	Myelin-associated glycoprotein OS=Mus musculus GN=Mag PE=1 SV=2
RASAL1	Q9Z268	10	10	28111.44	45781.67	36204.56	42710.67	57876	57358.22	72835.44	52475.11	52885.56	17981.78	0.434425639	0.362084551	42136.868	50707.222	Up	0.267108154622467	RasGAP-activating-like protein 1 OS=Mus musculus GN=Rasal1 PE=2 SV=2
RAB5C	P35278	2	2	32082	17495.5	56823.5	18134	22595.5	25468.5	22882	57091.5	31781.5	51479.5	0.4347377	0.361772696	29426.1	37740.6	Up	0.359021012303638	Ras-related protein Rab-5C OS=Mus musculus GN=Rab5c PE=1 SV=2
CACNA1D	Q99246-4	1	1	NA	1914	NA	5910	4018	7991	5957	NA	2841	NA	0.435308752	0.361202602	3947.3333	5596.3333	Up	0.503603542991672	Isoform 3 of Voltage-dependent L-type calcium channel subunit alpha-1D OS=Mus musculus GN=Cacna1d
PURG	Q8R4E6	4	4	39938	40271.25	16313.5	79777.5	43412.75	50206.25	43318.5	20419.5	43667.25	9074.25	0.435968474	0.360544915	43942.6	33337.15	Down	-0.398489460514054	Purine-rich element-binding protein gamma OS=Mus musculus GN=Purg PE=2 SV=1
PPP1R12A	Q9DBR7-2	4	4	47944.25	50987.5	56443.75	30473.75	50833.75	41124.5	46407.25	29114.75	63152.25	22120	0.436003008	0.360510515	47336.6	40383.75	Down	-0.229181202437992	Isoform 2 of Protein phosphatase 1 regulatory subunit 12A OS=Mus musculus GN=Ppp1r12a
ATP6V1G2	Q9WTT4	43	43	100201.66	80557.07	60345.17	95157.07	101694.76	98609.41	88328.54	44231.41	106098.41	28189.95	0.436231619	0.36028286	87591.146	73091.544	Down	-0.26108053518812	V-type proton ATPase subunit G 2 OS=Mus musculus GN=Atp6v1g2 PE=2 SV=1
RBM14	Q8C2Q3	4	4	80731.5	37876	45879.75	21353.5	46302	44184.5	39901.75	35325	40754.75	29204	0.436232375	0.360282107	46428.55	37874	Down	-0.293804427986894	RNA-binding protein 14 OS=Mus musculus GN=Rbm14 PE=1 SV=1
VCAN	Q62059-3	6	6	39442.33	42607.83	64304.33	27457.67	48744.33	37001.33	44140.83	47050	48516.83	97359.67	0.436248227	0.360266325	44511.298	54813.732	Up	0.300365792078739	Isoform V2 of Versican core protein OS=Mus musculus GN=Vcan
NUDC	O35685	4	4	42762	38784.25	56088.75	81196.75	43414.5	59010.75	47881.75	71113.25	40062	94949.75	0.436402882	0.36011239	52449.25	62603.5	Up	0.255321173687686	Nuclear migration protein nudC OS=Mus musculus GN=Nudc PE=1 SV=1
SMAP2	Q7TN29	3	3	142602.33	142851	123072.33	147325.67	163655.33	132497	148960	128121.67	165045.33	76774.67	0.43650503	0.360010747	143901.33	130279.73	Down	-0.143467267007408	Stromal membrane-associated protein 2 OS=Mus musculus GN=Smap2 PE=1 SV=1
TMPO	Q61029-3	2	2	4181.5	3108	3545.5	6220	4824.5	6435.5	3576.5	NA	6042.5	4348.5	0.436672008	0.359844647	4375.9	5100.75	Up	0.221129623118269	Isoform Epsilon of Lamina-associated polypeptide 2, isoforms beta/delta/epsilon/gamma OS=Mus musculus GN=Tmpo
ABCB10	Q9JI39	1	1	1655	6513	3711	762	1971	3161	2186	NA	3015	12094	0.436810226	0.359707203	2922.4	5114	Up	0.807298501766993	ATP-binding cassette sub-family B member 10, mitochondrial OS=Mus musculus GN=Abcb10 PE=2 SV=1
HMGB2	P30681	2	2	49046	31815.5	107334.5	22807	47679.5	33791	38566.5	93565.5	67184.5	112284	0.437205794	0.359314091	51736.5	69078.3	Up	0.417050120647468	High mobility group protein B2 OS=Mus musculus GN=Hmgb2 PE=1 SV=3
WDR54	Q9R0D8	2	2	3398.5	3189	1271	3196	4719.5	4484	6989.5	NA	2122.5	NA	0.437233399	0.359286671	3154.8	4532	Up	0.522599313368676	WD repeat-containing protein 54 OS=Mus musculus GN=Wdr54 PE=2 SV=1
NPLOC4	P60670	1	1	2511	6411	3083	12591	2878	9362	4617	NA	8499	NA	0.437493171	0.359028721	5494.8	7492.6667	Up	0.447412299518446	Nuclear protein localization protein 4 homolog OS=Mus musculus GN=Nploc4 PE=1 SV=3
PPP1R8	Q8R3G1	1	1	1223	1265	NA	1773	805	908	839	3778	2042	NA	0.437563718	0.358958696	1266.5	1891.75	Up	0.578874368079753	Nuclear inhibitor of protein phosphatase 1 OS=Mus musculus GN=Ppp1r8 PE=1 SV=1
RAP1A	P62835	2	2	49935.5	75604.5	87373.5	64246.5	50558	74395.5	69812.5	69658.5	53281.5	107536	0.437704682	0.358818808	65543.6	74936.8	Up	0.19321945660672	Ras-related protein Rap-1A OS=Mus musculus GN=Rap1a PE=2 SV=1
CPLX2	P84086	14	14	211892.17	209978.58	230542.83	177461.42	225305.67	183527.33	187764.17	275438.08	194005.5	382783.08	0.437870621	0.358654193	211036.13	244703.63	Up	0.213545473681791	Complexin-2 OS=Mus musculus GN=Cplx2 PE=1 SV=1
1810026J23RIK	Q8BGX2	2	2	726.5	1320.5	NA	5219.5	3523.5	3735.5	3451	NA	3671.5	NA	0.438835097	0.357698646	2697.5	3619.3333	Up	0.424101023432749	Uncharacterized protein C19orf52 homolog OS=Mus musculus PE=2 SV=1
HSPA12A	Q8K0U4	6	6	39605.17	48837.67	53750.67	70282.17	46733.17	59423.67	59949	58560.33	47278.17	57541.83	0.438967341	0.35756779	51841.77	56550.6	Up	0.125427355176609	Heat shock 70 kDa protein 12A OS=Mus musculus GN=Hspa12a PE=1 SV=1
KRAS	P32883-2	27	27	56817.83	62019	59239.13	70743.74	58381.57	62511.96	65347.09	64950.26	66113.35	59672.17	0.4389751	0.357560074	61440.254	63718.966	Up	0.0525386753951993	Isoform 2B of GTPase KRas OS=Mus musculus GN=Kras
PPP1CA	P62137	2	2	6664.5	4263.5	5930	8742.5	6121	7171.5	8173.5	4476	10263	NA	0.439107143	0.357429498	6344.3	7521	Up	0.245463504180389	Serine/threonine-protein phosphatase PP1-alpha catalytic subunit OS=Mus musculus GN=Ppp1ca PE=1 SV=1
COA7	Q921H9	3	3	53401	55546.33	64743	49240.33	46589.67	49950.33	50901	26179	74959	27596	0.439201878	0.357335812	53904.066	45917.066	Down	-0.231363640101704	Cytochrome c oxidase assembly factor 7 OS=Mus musculus GN=Coa7 PE=2 SV=1
EIF4G1	Q6NZJ6	3	3	33536.33	35932.33	21598.67	43058.67	48489.67	48282.67	49288.67	51672.33	55957.33	13903.67	0.439266287	0.357272127	36523.134	43820.934	Up	0.262809667962967	Eukaryotic translation initiation factor 4 gamma 1 OS=Mus musculus GN=Eif4g1 PE=1 SV=1
JAM2	Q9JI59	1	1	738	1227	NA	4152	1868	3097	3423	NA	1765	NA	0.439581978	0.356960122	1996.25	2761.6667	Up	0.468246789089063	Junctional adhesion molecule B OS=Mus musculus GN=Jam2 PE=1 SV=1
SCCPDH	Q8R127	1	1	38269	45986	104926	67286	41398	56448	45649	81002	64246	128074	0.439636365	0.356906391	59573	75083.8	Up	0.333843054281705	Saccharopine dehydrogenase-like oxidoreductase OS=Mus musculus GN=Sccpdh PE=2 SV=1
CALB1	P12658	4	4	67913.25	62771	99009.25	69026.5	64462	63244.5	83750.5	111890	65675.5	83308	0.440672186	0.355884361	72636.4	81573.7	Up	0.16741138932441	Calbindin OS=Mus musculus GN=Calb1 PE=1 SV=2
OCIAD2	Q9D8W7	9	9	55523.33	46574.67	47874.89	54572	48300.56	51194	51491.56	65089	55030.56	45405.89	0.441430943	0.355137227	50569.09	53642.202	Up	0.085112643617664	OCIA domain-containing protein 2 OS=Mus musculus GN=Ociad2 PE=1 SV=1
NWD2	Q6P5U7	1	1	62478	72364	118263	19433	63886	51278	58800	61000	86655	240213	0.441644613	0.354927063	67284.8	99589.2	Up	0.565708667964432	Leucine-rich repeat and WD repeat-containing protein KIAA1239 OS=Mus musculus GN=Kiaa1239 PE=1 SV=2
SIRT5	Q8K2C6	4	4	37046.25	37312	6498	43382.75	45686	55178.5	45023	24712.25	42003	NA	0.441667553	0.354904505	33985	41729.188	Up	0.29615870765642	NAD-dependent protein deacylase sirtuin-5, mitochondrial OS=Mus musculus GN=Sirt5 PE=1 SV=1
GNG12	Q9DAS9	7	7	74677	95052.14	52152.43	107136.57	92271	103967.29	93376	38758.14	92089.57	4681.86	0.441852034	0.354723141	84257.828	66574.572	Down	-0.339839478006471	Guanine nucleotide-binding protein G(I)/G(S)/G(O) subunit gamma-12 OS=Mus musculus GN=Gng12 PE=1 SV=3
IVD	Q9JHI5	5	5	47829.8	49635.8	70474.6	45680.8	41710	52286.6	57138.2	62893.6	51741	53839	0.441887982	0.35468781	51066.2	55579.68	Up	0.122188820774291	Isovaleryl-CoA dehydrogenase, mitochondrial OS=Mus musculus GN=Ivd PE=1 SV=1
SERBP1	Q9CY58-4	3	3	75113.33	82751	67930.67	147360.67	65421.33	95994.67	87356.67	46450	105256.33	6909.33	0.441976749	0.354600577	87715.4	68393.4	Down	-0.35897304472672	Isoform 4 of Plasminogen activator inhibitor 1 RNA-binding protein OS=Mus musculus GN=Serbp1
ZFP326	O88291	3	3	13180	9631.67	6306	5978	10052	11223	6950.33	NA	20028.33	NA	0.442450981	0.354134838	9029.534	12733.887	Up	0.495949389682645	DBIRD complex subunit ZNF326 OS=Mus musculus GN=Znf326 PE=2 SV=1
TPI1	P17751	69	69	98132.52	96563.41	97859.99	99758.62	99451.14	97950.46	102506.28	97985.38	91621.3	93274.49	0.443009591	0.353586871	98353.136	96667.582	Down	-0.0249388985477106	Triosephosphate isomerase OS=Mus musculus GN=Tpi1 PE=1 SV=4
LGI1	Q9JIA1	1	1	7673	7461	NA	15844	6139	12232	8619	NA	13694	NA	0.443087591	0.353510412	9279.25	11515	Up	0.311434302497918	Leucine-rich glioma-inactivated protein 1 OS=Mus musculus GN=Lgi1 PE=1 SV=1
HNRNPF	Q9Z2X1-2	5	5	15258.6	14556.6	9486	29709.4	15248.8	20745.8	15854.6	9971.8	15160	5033	0.443478988	0.353126952	16851.88	13353.04	Down	-0.335741319481445	Isoform 2 of Heterogeneous nuclear ribonucleoprotein F OS=Mus musculus GN=Hnrnpf
SIRPA	P97797-2	8	8	7459.62	9649.62	10387.12	29427	10962.62	25695.5	21840.5	14245.25	14745.62	10982.38	0.443683062	0.352927151	13577.196	17501.85	Up	0.366321866490066	Isoform 2 of Tyrosine-protein phosphatase non-receptor type substrate 1 OS=Mus musculus GN=Sirpa
RABEP1	O35551-3	6	6	9669.4	13211.4	6406	10813.8	11122.8	15161.6	11662.8	4728	23214.2	NA	0.444013141	0.352604176	10244.68	13691.65	Up	0.418421396853039	Isoform 3 of Rab GTPase-binding effector protein 1 OS=Mus musculus GN=Rabep1
HSDL2	Q2TPA8	1	1	20100	21103	27281	8083	20176	13278	16774	21160	20742	72747	0.444247822	0.352374693	19348.6	28940.2	Up	0.580845710527364	Hydroxysteroid dehydrogenase-like protein 2 OS=Mus musculus GN=Hsdl2 PE=1 SV=1
CPLX1	P63040	19	19	157505.63	160405.63	170411.79	144521.42	166716.42	141069.63	144064.47	208620.05	148143.21	254308.53	0.444335451	0.352289035	159912.18	179241.18	Up	0.164622302810111	Complexin-1 OS=Mus musculus GN=Cplx1 PE=1 SV=1
BRK1	Q91VR8	11	11	152203.45	144148.73	161249.18	90207.55	132099.27	110772	119756.18	226749.55	159061.91	160979	0.44444678	0.352180235	135981.64	155463.73	Up	0.193166185346841	Protein BRICK1 OS=Mus musculus GN=Brk1 PE=2 SV=1
CAMSAP1	A2AHC3	1	1	18037	23936	73236	25375	32004	21891	31392	62069	34358	83764	0.444914865	0.351723084	34517.6	46694.8	Up	0.435929740505362	Calmodulin-regulated spectrin-associated protein 1 OS=Mus musculus GN=Camsap1 PE=1 SV=1
GNB5	P62881-2	5	5	30084.4	29808.4	33667.2	23285.2	22520.6	30374.4	26248.4	31712	38779.6	25214.2	0.444925725	0.351712483	27873.16	30465.72	Up	0.128310265064263	Isoform 2 of Guanine nucleotide-binding protein subunit beta-5 OS=Mus musculus GN=Gnb5
PPP1R12A	Q9DBR7	5	5	51075.2	51009.8	51034	29771.2	52463.6	39795.2	46191	30122.2	64817.6	17696	0.445151857	0.35149181	47070.76	39724.4	Down	-0.244805717655935	Protein phosphatase 1 regulatory subunit 12A OS=Mus musculus GN=Ppp1r12a PE=1 SV=2
PSMA4	Q9R1P0	1	1	13358	17732	14075	15339	11716	15991	12945	11637	12757	NA	0.445181482	0.351462909	14444	13332.5	Down	-0.115522997378283	Proteasome subunit alpha type-4 OS=Mus musculus GN=Psma4 PE=1 SV=1
ARHGAP21	Q6DFV3	3	3	12960	15738.67	14608	13698.33	18853	18414	19761.33	9030.67	26036	NA	0.445445889	0.351205044	15171.6	18310.5	Up	0.271297946447967	Rho GTPase-activating protein 21 OS=Mus musculus GN=Arhgap21 PE=1 SV=1
XK	Q9QXY7	1	1	981	1244	NA	1767	1560	3097	845	NA	2073	NA	0.445813241	0.350847036	1388	2005	Up	0.530594668764022	Membrane transport protein XK OS=Mus musculus GN=Xk PE=1 SV=1
ADAM10	O35598	3	3	13522	15007.67	28987	6723	14346	14426.33	12599.33	23921	24026.33	21222.67	0.445996272	0.350668772	15717.134	19239.132	Up	0.291705543479939	Disintegrin and metalloproteinase domain-containing protein 10 OS=Mus musculus GN=Adam10 PE=1 SV=2
LMNA	P48678-2	13	13	47127.92	22289	28284.38	18984.69	28430	28475.15	23420.92	21844.92	25465.23	NA	0.446169829	0.350499801	29023.198	24801.555	Down	-0.226775918357612	Isoform C of Prelamin-A/C OS=Mus musculus GN=Lmna
PHB2	O35129	19	19	53950.05	53108.68	30725.84	58392.63	55227.37	58867.47	52999.95	30389.53	54783.74	14003.16	0.446508929	0.350169852	50280.914	42208.77	Down	-0.252468086592967	Prohibitin-2 OS=Mus musculus GN=Phb2 PE=1 SV=1
DLGAP4	B1AZP2	21	21	37901.32	35556.68	23604.95	33831.95	40407.37	35925.11	35804.26	28918.47	32926.16	22278.74	0.446670498	0.350012731	34260.454	31170.548	Down	-0.136360750548113	Disks large-associated protein 4 OS=Mus musculus GN=Dlgap4 PE=1 SV=1
TMPRSS13	Q5U405	2	2	376103	742150	401495	433840.5	272970.5	272477	421140.5	224531	393677	528749	0.446674232	0.350009101	445311.8	368114.9	Down	-0.27465969509609	Transmembrane protease serine 13 OS=Mus musculus GN=Tmprss13 PE=2 SV=2
SLC25A18	Q9DB41	3	3	29183.67	33962.67	49011	32102	37849	36745.33	38098.33	42073.33	30037	59164.67	0.447683199	0.349029204	36421.668	41223.732	Up	0.178676123706931	Mitochondrial glutamate carrier 2 OS=Mus musculus GN=Slc25a18 PE=2 SV=4
KIF3B	Q61771	8	8	15175.75	21154	9052.12	22102.5	19391.88	24890	20115	13834.12	21354.5	NA	0.448116985	0.348608595	17375.25	20048.405	Up	0.20645372821667	Kinesin-like protein KIF3B OS=Mus musculus GN=Kif3b PE=1 SV=1
LMBRD2	Q8C561-3	1	1	877	3256	788	7152	4216	5190	7033	NA	2148	NA	0.448157777	0.348569063	3257.8	4790.3333	Up	0.55622801086946	Isoform 3 of LMBR1 domain-containing protein 2 OS=Mus musculus GN=Lmbrd2
SLC32A1	O35633	7	7	25066.14	27125.43	40321.43	17809.86	26453.14	22899	24681.57	41869.71	29197.43	39173.57	0.448391106	0.34834301	27355.2	31564.256	Up	0.206476642160204	Vesicular inhibitory amino acid transporter OS=Mus musculus GN=Slc32a1 PE=1 SV=3
SYT5	Q9R0N5	2	2	86248.5	123425	159290.5	406344	83119	190245.5	183659	283614.5	123870	376731.5	0.448407247	0.348327377	171685.4	231624.1	Up	0.432018011686563	Synaptotagmin-5 OS=Mus musculus GN=Syt5 PE=1 SV=1
DCTN3	Q9Z0Y1	11	11	60872.73	64262.45	69030.91	59557.91	53555	62368.09	63696.36	58836.36	68815.73	65861.73	0.448790812	0.347956043	61455.8	63915.654	Up	0.0566201403839614	Dynactin subunit 3 OS=Mus musculus GN=Dctn3 PE=2 SV=2

CNST	Q8CBC4	1	1	2752	6510	NA	17969	6244	13861	9521	NA	NA	NA	0.449313126	0.347450894	8368.75	11691	Up	0.482314281791158	Consortin OS=Mus musculus GN=Cnst PE=1 SV=1
HADHB	Q99JY0	13	13	76322.15	70166.69	80925.31	31649.54	62397.23	58561.38	64132.23	121021.23	65550.69	69653.62	0.449393927	0.347372801	64292.184	75783.83	Up	0.23724669351626	Trifunctional enzyme subunit beta, mitochondrial OS=Mus musculus GN=Hadhb PE=1 SV=1
PLXNA1	P70206	8	8	19675.29	32221.86	24660.71	35176.29	39595.14	37187.29	46552.43	33138.71	29030.14	25682.29	0.449871592	0.34691143	30265.858	34318.172	Up	0.181281457678173	Plexin-A1 OS=Mus musculus GN=Plxna1 PE=1 SV=1
SBDS	P70122	2	2	43808	47977	33790.5	25525	43429.5	37845.5	31288	11718.5	53313.5	28754	0.450253456	0.346542945	38906	32583.9	Down	-0.25583336987952	Ribosome maturation protein SBDS OS=Mus musculus GN=Sbds PE=1 SV=4
DMTN	Q9WV69-4	16	16	66834.93	58922.07	46497.73	67986.8	71264.33	65620.67	64607.47	52860.4	66786.93	25528.6	0.450260835	0.346535827	62301.172	55080.814	Down	-0.177709423070498	Isoform 4 of Dematin OS=Mus musculus GN=Dmtn
UCHL4	P58321	2	2	20511.5	26007.5	9163.5	21083	23048	30458.5	20075	21307	20166.5	NA	0.450568266	0.346239399	19962.7	23001.75	Up	0.204436765652811	Ubiquitin carboxyl-terminal hydrolase isozyme L4 OS=Mus musculus GN=Uchl4 PE=2 SV=1
TUBB2B	Q9CWF2	126	126	67399.79	74668.68	61136.81	86219.74	60378.49	71882.86	71937.1	63731.42	63270.12	56391.69	0.450778609	0.346036701	69960.702	65442.638	Down	-0.0963138609804458	Tubulin beta-2B chain OS=Mus musculus GN=Tubb2b PE=1 SV=1
PITPNB	P53811	1	1	49367	64196	53340	69641	72314	104734	83744	NA	45483	NA	0.450962922	0.345859164	61771.6	77987	Up	0.336289955085959	Phosphatidylinositol transfer protein beta isoform OS=Mus musculus GN=Pitpnb PE=1 SV=2
SEPT5	Q9Z2Q6	28	28	160991.21	154699.71	183907.25	154458.14	160106.86	150754.18	158301.93	173284.96	170264.25	204357.82	0.451056373	0.345769177	162832.63	171392.63	Up	0.0739151916768899	Septin-5 OS=Mus musculus GN=Sept5 PE=1 SV=2
ACIN1	Q9JIX8-3	1	1	22507	22967	19282	23041	23557	30883	26100	19610	21421	NA	0.451114877	0.34571285	22270.8	24503.5	Up	0.13783445100812	Isoform 3 of Apoptotic chromatin condensation inducer in the nucleus OS=Mus musculus GN=Acin1
NUTF2	P61971	1	1	26885	26125	NA	7820	19782	13865	21190	NA	25492	48468	0.45111523	0.34571251	20153	27253.75	Up	0.435460136173294	Nuclear transport factor 2 OS=Mus musculus GN=Nutf2 PE=2 SV=1
NETO1	Q8R4I7	1	1	NA	2800	NA	6770	1059	5678	NA	NA	4590	NA	0.451292269	0.345542106	3543	5134	Up	0.535111830457105	Neuropilin and tolloid-like protein 1 OS=Mus musculus GN=Neto1 PE=1 SV=2
RUVBL2	Q9WTM5	2	2	16223	18464.5	1543	13630.5	13433	18782	18517	6238.5	21491	NA	0.451671084	0.345177712	12658.8	16257.125	Up	0.360931495851008	RuvB-like 2 OS=Mus musculus GN=Ruvbl2 PE=2 SV=3
MRPS35	Q8BJZ4	3	3	113512.67	141853	118822.33	77163	100535.33	103827	131573	80782	137357.67	174523.33	0.452064108	0.344799973	110377.27	125612.6	Up	0.186538129816401	28S ribosomal protein S35, mitochondrial OS=Mus musculus GN=Mrps35 PE=2 SV=2
VPS4B	P46467	1	1	39960	35124	42086	27764	39881	41026	35297	31614	27730	NA	0.452827171	0.344067522	36963	33916.75	Down	-0.124083920513285	Vacuolar protein sorting-associated protein 4B OS=Mus musculus GN=Vps4b PE=1 SV=2
SYNPO	Q8CC35-3	49	49	54999.54	49460.88	58169.88	25239.06	51889.15	36461.85	45540.27	60512.4	60877.35	69210.4	0.452913284	0.343984941	47951.702	54520.454	Up	0.185215552492334	Isoform 3 of Synaptopodin OS=Mus musculus GN=Synpo
DAZAP1	Q9JII5	5	5	82040	67198.6	69184.2	45475.2	67645.8	50195.4	54411.4	66763.4	64588.4	68134.6	0.453194061	0.34371579	66308.76	60818.64	Down	-0.124685920466562	DAZ-associated protein 1 OS=Mus musculus GN=Dazap1 PE=2 SV=2
DLGAP4	B1AZP2-2	23	23	36964.86	35174.1	22616.29	34440.52	38976.24	35912.14	35564	27648.71	32667.1	20156.95	0.453202768	0.343707446	33634.402	30389.78	Down	-0.146351377292407	Isoform 2 of Disks large-associated protein 4 OS=Mus musculus GN=Dlgap4
TPRGL	Q9DBS2	1	1	2399	NA	NA	3323	1843	3509	2621	NA	NA	NA	0.453354723	0.343561855	2521.6667	3065	Up	0.281509492195201	Tumor protein p63-regulated gene 1-like protein OS=Mus musculus GN=Tprg1l PE=1 SV=1
UBXN1	Q922Y1	2	2	34749	45567	27022	46494.5	60040	54954	74521.5	18789.5	67822.5	NA	0.453621744	0.343306136	42774.5	54021.875	Up	0.336792723157596	UBX domain-containing protein 1 OS=Mus musculus GN=Ubxn1 PE=1 SV=1
NLGN1	Q99K10	1	1	NA	NA	NA	19251	14703	20884	21141	NA	16543	NA	0.453777437	0.343157102	16977	19522.667	Up	0.201568585671615	Neuroligin-1 OS=Mus musculus GN=Nlgn1 PE=1 SV=2
CSNK2A1	Q60737	4	4	30183.25	31183	30766.75	38427.25	34956.25	45481	41663.25	5024.75	32388.5	3391.25	0.454042636	0.342903364	33103.3	25589.75	Down	-0.371408990523417	Casein kinase II subunit alpha OS=Mus musculus GN=Csnk2a1 PE=1 SV=2
RAPGEF2	Q8CHG7	21	21	23802.81	29180.95	16920.33	36867.43	37207.81	40071.14	44666.29	20371.81	43621.95	21540.67	0.454550741	0.34241763	28795.866	34054.372	Up	0.241978317589078	Rap guanine nucleotide exchange factor 2 OS=Mus musculus GN=Rapgef2 PE=1 SV=2
SUCLG1	Q9WUM5	20	20	171047.25	167260.75	172719.2	170708.45	149820.1	149659.7	143454.05	196173.95	168068.55	271562.7	0.454748139	0.34222907	166311.15	185783.79	Up	0.159739734755485	Succinyl-CoA ligase [ADP/GDP-forming] subunit alpha, mitochondrial OS=Mus musculus GN=Suclg1 PE=1 SV=4
RBFOX1	Q9JJ43-3	1	1	3229	3283	2047	2661	2718	2776	2635	NA	5906	NA	0.455192861	0.341804557	2787.6	3772.3333	Up	0.436433602008003	Isoform 3 of RNA binding protein fox-1 homolog 1 OS=Mus musculus GN=Rbfox1
XPR1	Q9Z0U0	1	1	649	1033	NA	4521	1840	3719	3592	NA	1478	NA	0.455238902	0.341760633	2010.75	2929.6667	Up	0.5430028061404	Xenotropic and polytropic retrovirus receptor 1 OS=Mus musculus GN=Xpr1 PE=1 SV=1
PTPRZ1	B9EKR1	13	13	48392	60660.46	90329.77	34988.54	49987.08	47281.08	53922.38	74596	68929.15	85233.08	0.455385888	0.341620432	56871.57	65992.338	Up	0.214590899003412	Receptor-type tyrosine-protein phosphatase zeta OS=Mus musculus GN=Ptprz1 PE=1 SV=1
ARF5	P84084	2	2	20243.5	30590.5	14137.5	25464	25098	30524	30716.5	14550	32918.5	NA	0.456175637	0.340867912	23106.7	27177.25	Up	0.234088245693367	ADP-ribosylation factor 5 OS=Mus musculus GN=Arf5 PE=1 SV=2
TJP1	P39447	17	17	22563.62	24834.08	19286.46	19752.85	26583.08	25082.15	26580.23	11459.31	26292.62	3928.23	0.456214495	0.340830921	22604.018	18668.508	Down	-0.275972612155315	Tight junction protein ZO-1 OS=Mus musculus GN=Tjp1 PE=1 SV=2
PPP2CB	P62715	4	4	10582.75	15219.5	19573.25	24148.75	20686.25	17882.5	26715	9401	14091	4541.75	0.456677036	0.340390826	18042.1	14526.25	Down	-0.312704955550438	Serine/threonine-protein phosphatase 2A catalytic subunit beta isoform OS=Mus musculus GN=Ppp2cb PE=1 SV=1
AFG3L2	Q8JZQ2	10	10	16832.1	23436.7	22743.5	38779.4	26891.5	37089.5	32047.6	32914.7	27577.1	18100.4	0.456950487	0.340130856	25736.64	29545.86	Up	0.199132275032503	AFG3-like protein 2 OS=Mus musculus GN=Afg3l2 PE=1 SV=1
LIMA1	Q9ERG0	2	2	30589	34353.5	47116.5	13909.5	33985	25204.5	34679.5	20144.5	51996	69774	0.457062681	0.340024237	31990.7	40359.7	Up	0.335262889777428	LIM domain and actin-binding protein 1 OS=Mus musculus GN=Lima1 PE=1 SV=3
KLC2	O88448	16	16	40166.85	45562.15	41099	41149.54	49401	46213.54	48440.69	20064.15	51790.08	22123.23	0.457148277	0.339942913	43475.708	37726.338	Down	-0.204637453755152	Kinesin light chain 2 OS=Mus musculus GN=Klc2 PE=1 SV=1
SLC9A3R1	P70441-2	1	1	32932	25479	56848	20916	22915	21844	19226	69030	29273	73305	0.457290411	0.339807905	31818	42535.6	Up	0.418827650342771	Isoform 2 of Na(+)/H(+) exchange regulatory cofactor NHE-RF1 OS=Mus musculus GN=Slc9a3r1
TIMM8A2	Q4FZG7	1	1	117913	79289	142385	42959	72973	60741	106396	159850	115658	104949	0.457454026	0.339652546	91103.8	109518.8	Up	0.265595407989213	Putative mitochondrial import inner membrane translocase subunit Tim8 A-B OS=Mus musculus GN=Timm8a2 PE=3 SV=1
OGDH	Q60597-3	21	21	82347.86	108448.33	69687.05	110236.05	123269	126613.81	143682	82089.43	115898.9	85883.52	0.457706338	0.339413074	98797.658	110833.53	Up	0.165845677674265	Isoform 3 of 2-oxoglutarate dehydrogenase, mitochondrial OS=Mus musculus GN=Ogdh
STX4A	P70452	2	2	1412	4849.5	1474.5	12113	4623.5	8103	6865	NA	4887	NA	0.458567692	0.338596546	4894.5	6618.3333	Up	0.435306466877654	Syntaxin-4 OS=Mus musculus GN=Stx4 PE=1 SV=1
PRKCSH	O08795	17	17	40984.5	50310.07	32150.64	65448.5	46224.43	50849.07	50965.21	41975.21	42130.29	16615.5	0.458921245	0.338261837	47023.628	40507.056	Down	-0.215212615466379	Glucosidase 2 subunit beta OS=Mus musculus GN=Prkcsh PE=1 SV=1
GPRASP2	Q8BUY8	1	1	NA	NA	NA	5027	3355	5355	4884	NA	NA	NA	0.458929193	0.338254316	4191	5119.5	Up	0.288708393689402	G-protein coupled receptor-associated sorting protein 2 OS=Mus musculus GN=Gprasp2 PE=2 SV=2
ETFB	Q9DCW4	22	22	78381.45	81092.68	59502.55	71505.32	80544.23	77555.23	78298.18	63821	78823	36822.95	0.459283217	0.337919425	74205.246	67064.072	Down	-0.145981099518746	Electron transfer flavoprotein subunit beta OS=Mus musculus GN=Etfb PE=1 SV=3
CETN2	Q9R1K9	4	4	18825.5	20992	2585.5	15538.5	20279.5	18301.5	16490	NA	20850.5	NA	0.459538546	0.337678054	15644.2	18547.333	Up	0.245583891289866	Centrin-2 OS=Mus musculus GN=Cetn2 PE=1 SV=1
AMPH	Q7TQF7	46	46	102461	122671.51	109063.04	168825.02	120720.89	128246.93	127624.04	109330.16	119302.98	83589.96	0.460042982	0.33720159	124748.29	113618.81	Down	-0.134818314211793	Amphiphysin OS=Mus musculus GN=Amph PE=1 SV=1
DACT3	Q0PHV7	7	7	6210.8	9367.8	4126.6	6977.4	7236	8789	8471.4	9495.4	14494.8	1549.2	0.460214187	0.337039997	6783.72	8559.96	Up	0.335527431569066	Dapper homolog 3 OS=Mus musculus GN=Dact3 PE=1 SV=1
PTPRJ	Q64455	1	1	NA	2739	NA	9625	959	6005	7742	NA	NA	NA	0.460495867	0.336774263	4441	6873.5	Up	0.630160338049009	Receptor-type tyrosine-protein phosphatase eta OS=Mus musculus GN=Ptprj PE=1 SV=2
VPS37B	Q8R0J7	4	4	24528.5	27491.75	19340	17538.5	27741	24395.25	29338.25	30520.5	27651	17096.5	0.461142894	0.33616448	23327.95	25800.3	Up	0.145328308496716	Vacuolar protein sorting-associated protein 37B OS=Mus musculus GN=Vps37b PE=2 SV=1
GRK2	Q99MK8	2	2	1171	3284	NA	7606	1647	5332	4118	NA	NA	NA	0.461375272	0.335945686	3427	4725	Up	0.463368137476623	Beta-adrenergic receptor kinase 1 OS=Mus musculus GN=Adrbk1 PE=1 SV=2
DMTN	Q9WV69-3	18	18	57099.44	50618.61	39943.72	57786.67	60901.28	56264.56	55372.22	45103.89	57761.11	21273.83	0.461721848	0.335619575	53269.944	47155.122	Down	-0.175907278994648	Isoform 3 of Dematin OS=Mus musculus GN=Dmtn
FAM177A	Q8BR63	4	4	9368.5	11603.5	1721.5	16724.5	11318	13456.5	11816.5	NA	11240.5	NA	0.461870413	0.335479858	10147.2	12171.167	Up	0.262385776485101	Protein FAM177A1 OS=Mus musculus GN=Fam177a1 PE=2 SV=1
2700060E02RIK	Q9CQE8	1	1	23330	22107	21175	37352	23898	24909	25450	NA	16482	NA	0.462160307	0.335207357	25572.4	22280.333	Down	-0.198816748747459	UPF0568 protein C14orf166 homolog OS=Mus musculus PE=2 SV=1
TMEM169	Q8BG50	1	1	39742	35232	35486	13960	34209	24294	34148	42206	45077	35321	0.463241311	0.334192718	31725.8	36209.2	Up	0.190699758879122	Transmembrane protein 169 OS=Mus musculus GN=Tmem169 PE=2 SV=1
PPFIA3	P60469	20	20	15434.7	17988.2	10852.75	26574.9	20117.45	24730.75	21672.05	24642.1	27315.95	8783.4	0.463492473	0.333957314	18193.6	21428.85	Up	0.2361233885939	Liprin-alpha-3 OS=Mus musculus GN=Ppfia3 PE=1 SV=1
MECR	Q9DCS3	8	8	55878	56510.67	51052.17	81434.83	53974.83	70453.5	57932.17	39964.83	57201	43386.5	0.464009591	0.333473042	59770.1	53787.6	Down	-0.152150339122343	Trans-2-enoyl-CoA reductase, mitochondrial OS=Mus musculus GN=Mecr PE=1 SV=2
IGBP1	Q61249	3	3	7166	5098.33	8693	6539.33	5687	6786	6528.67	NA	8992.33	NA	0.464900092	0.332640367	6636.732	7435.6667	Up	0.163989079686976	Immunoglobulin-binding protein 1 OS=Mus musculus GN=Igbp1 PE=1 SV=1
CDK5	P49615	8	8	29713.62	42462.38	43373.12	35071.62	39163.12	40695.75	37402.38	25656	52045	58715.75	0.46492854	0.332613794	37956.772	42902.976	Up	0.176720420060568	Cyclin-dependent kinase 5 OS=Mus musculus GN=Cdk5 PE=1 SV=1
ARMCX2	Q6A058	1	1	3180	9744	NA	18940	9973	16572	25398	NA	5659	NA	0.465061287	0.332489811	10459.25	15876.333	Up	0.602098353874513	Armadillo repeat-containing X-linked protein 2 OS=Mus musculus GN=Armcx2 PE=2 SV=2
CAMKK2	Q8C078-3	6	6	5799	6849.25	4078.25	8822.5	7850.75	9631	9065.5	4161.5	8566.75	NA	0.465068479	0.332483095	6679.95	7856.1875	Up	0.233992058156637	Isoform 3 of Calcium/calmodulin-dependent protein kinase kinase 2 OS=Mus musculus GN=Camkk2
DPY30	Q99LT0	2	2	28237	35931	40266.5	34086.5	33069	28993.5	41861.5	33576.5	32991	51803	0.465570858	0.332014212	34318	37845.1	Up	0.141141041895549	Protein dpy-30 homolog OS=Mus musculus GN=Dpy30 PE=1 SV=1
IGF2R	Q07113	1	1	NA	5260	3365	7953	6384	8204	8535	NA	4431	NA	0.465647112	0.331943086	5740.5	7056.6667	Up	0.297810461784144	Cation-independent mannose-6-phosphate receptor OS=Mus musculus GN=Igf2r PE=1 SV=1
SUCLA2	Q9Z2I9	12	12	46614.92	56991.83	53559.58	77229.75	56723.33	69833.33	57670.75	42211.08	51016.25	42591.08	0.465949524	0.331661128	58223.882	52664.498	Down	-0.144780287261854	Succinyl-CoA ligase [ADP-forming] subunit beta, mitochondrial OS=Mus musculus GN=Sucla2 PE=1 SV=2
CALB2	Q08331	2	2	36738	50172.5	48037	80760.5	48431	61575	51423	47918.5	84770.5	NA	0.466128233	0.331494591	52827.8	61421.75	Up	0.217452287015757	Calretinin OS=Mus musculus GN=Calb2 PE=1 SV=3
PRKCA	P20444	5	5	64588.8	79076.6	71943.2	98071	90316	98008	98131	115373.2	91762.8	49403.8	0.466788587	0.330879771	80799.12	90535.76	Up	0.164148163035695	Protein kinase C alpha type OS=Mus musculus GN=Prkca PE=1 SV=3
TCEB2	P62869	2	2	38724	31710.5	76940	52880.5	35610	40015	39388	68508.5	38822.5	113970	0.467125123	0.330566774	47173	60140.8	Up	0.350382705951377	Transcription elongation factor B polypeptide 2 OS=Mus musculus GN=Tceb2 PE=1 SV=1
CDHR3	Q8BL00	1	1	3180	4808	1881	3343	4037	3761	2588	NA	2532	NA	0.467551273	0.330170756	3449.8	2960.3333	Down	-0.220753092606572	Cadherin-related family member 3 OS=Mus musculus GN=Cdhr3 PE=2 SV=2
TMEM163	Q8C996	2	2	1353	2424.5	2290	7847	3324	6436	4834	1942	5136.5	NA	0.468052346	0.329705574	3447.7	4587.125	Up	0.411955977205345	Transmembrane protein 163 OS=Mus musculus GN=Tmem163 PE=1 SV=1
ASPSCR1	Q8VBT9-3	1	1	7674	9046	6591	4092	7849	7120	7027	NA	10135	NA	0.46810554	0.329656219	7050.4	8094	Up	0.199147738780165	Isoform 3 of Tether containing UBX domain for GLUT4 OS=Mus musculus GN=Aspscr1
ITCH	Q8C863	2	2	1250	3511	NA	5254	3827	5656	5467	NA	2440	NA	0.468212401	0.329557088	3460.5	4521	Up	0.385661412914841	E3 ubiquitin-protein ligase Itchy OS=Mus musculus GN=Itch PE=1 SV=2
HIST1H4A	P62806	19	19	214061.47	105742.05	164043.16	85676.21	129662.47	95071.42	83821	145357.74	72323	184575.11	0.468520231	0.329271651	139837.07	116229.65	Down	-0.266768687609155	Histone H4 OS=Mus musculus GN=Hist1h4a PE=1 SV=2
CACNA1E	Q61290	13	13	7473.85	18720.08	7234.54	29595.23	16920.69	24979.77	28927.85	11171.77	16555.77	NA	0.468851758	0.328964452	15988.878	20408.79	Up	0.352121947137432	Voltage-dependent R-type calcium channel subunit alpha-1E OS=Mus musculus GN=Cacna1e PE=1 SV=1
WNK2	Q3UH66-2	5	5	21069	23586.5	19573.75	24136.75	27211.5	30345	29369.25	20046.25	34823.75	15727	0.468854247	0.328962146	23115.5	26062.25	Up	0.173101070552069	Isoform 2 of Serine/threonine-protein kinase WNK2 OS=Mus musculus GN=Wnk2
RPL23	P62830	9	9	216797.22	161276	149274	158725.44	199833.56	174272.22	177055.44	166729.67	201207.78	235771.33	0.469350423	0.328502787	177181.24	191007.29	Up	0.108401794635182	60S ribosomal protein L23 OS=Mus musculus GN=Rpl23 PE=1 SV=1
BBC3	Q99ML1	1	1	NA	3442	NA	2669	1840	3238	1964	NA	5913	NA	0.469632831	0.32824155	2650.3333	3705	Up	0.483299723193265	Bcl-2-binding component 3 OS=Mus musculus GN=Bbc3 PE=1 SV=1
NLN	Q91YP2	1	1	28601	31424	57162	22434	27178	35284	32550	71544	34478	30427	0.469673556	0.328203891	33359.8	40856.6	Up	0.292458511524904	Neurolysin, mitochondrial OS=Mus musculus GN=Nln PE=2 SV=1
CTSZ	Q9WUU7	2	2	3001.5	6630	4948.5	5151.5	4567	6472	4218	2737.5	15647.5	NA	0.470437124	0.327498414	4859.7	7268.75	Up	0.58084003075297	Cathepsin Z OS=Mus musculus GN=Ctsz PE=2 SV=1
TCEA1	P10711	6	6	7610.2	5919	3521.8	8477.4	6814.8	8160	6348.8	4881.4	11968.4	NA	0.470520921	0.327421061	6468.64	7839.65	Up	0.277326822368696	Transcription elongation factor A protein 1 OS=Mus musculus GN=Tcea1 PE=1 SV=2
ALPL	P09242	1	1	2812	9340	4054	9034	7959	7466	6494	NA	9802	NA	0.47066758	0.327285715	6639.8	7920.6667	Up	0.254482077922767	Alkaline phosphatase, tissue-nonspecific isozyme OS=Mus musculus GN=Alpl PE=1 SV=2
CPPED1	Q8BFS6-3	1	1	831	NA	NA	1916	737	707	3964	NA	1472	NA	0.470989985	0.326988328	1161.3333	2047.6667	Up	0.818198759365057	Isoform 3 of Calcineurin-like phosphoesterase domain-containing protein 1 OS=Mus musculus GN=Cpped1
LIN7B	O88951	12	12	43993.75	45693.75	28480.33	42934.67	44874.33	49520.58	44477.17	18695	46054.17	17124.92	0.471200141	0.326794589	41195.366	35174.368	Down	-0.227957558221761	Protein lin-7 homolog B OS=Mus musculus GN=Lin7b PE=1 SV=2
DCTN1	O08788	31	31	29251.45	40690.83	29541.48	61803.59	47806.93	57743.21	64055.24	35665.62	51363.59	32831.41	0.472258409	0.3258203	41818.856	48331.814	Up	0.208819547335904	Dynactin subunit 1 OS=Mus musculus GN=Dctn1 PE=1 SV=3
CFL1	P18760	52	52	132304.47	124617.02	148073.84	144690.06	108215.04	112529	127938.31	160432.49	142604.78	158226.49	0.472884244	0.325245156	131580.09	140346.21	Up	0.0930489852399605	Cofilin-1 OS=Mus musculus GN=Cfl1 PE=1 SV=3
ACTN3	O88990	1	1	64008	111777	55251	166852	137132	149333	178639	85678	87729	138914	0.472933465	0.325199954	107004	128058.6	Up	0.25913941476268	Alpha-actinin-3 OS=Mus musculus GN=Actn3 PE=2 SV=1
ACTN2	Q9JI91	2	2	64008	111777	55251	166852	137132	149333	178639	85678	87729	138914	0.472933465	0.325199954	107004	128058.6	Up	0.25913941476268	Alpha-actinin-2 OS=Mus musculus GN=Actn2 PE=1 SV=2
SEPT7	O55131	53	53	55198.65	58915.76	61004.18	63093.43	60578.49	59580.12	62350.86	51838.65	65506.59	42847.29	0.473307855	0.324856288	59758.102	56424.702	Down	-0.0828074328402487	Septin-7 OS=Mus musculus GN=Sept7 PE=1 SV=1
TFAM	P40630	5	5	17781.4	17804.6	9053.6	25989	19688	21961.4	20078.6	9342	18454.2	3907.4	0.4743563	0.323895327	18063.32	14748.72	Down	-0.29247332903102	Transcription factor A, mitochondrial OS=Mus musculus GN=Tfam PE=1 SV=2
EIF3B	Q8JZQ9	1	1	16926	23739	NA	35619	20918	28732	35807	NA	21684	NA	0.474825763	0.323465725	24300.5	28741	Up	0.242124260470453	Eukaryotic translation initiation factor 3 subunit B OS=Mus musculus GN=Eif3b PE=1 SV=1
ATAD3A	Q925I1	9	9	42823.33	47896.56	37808.78	48959.89	49726.89	56417.44	47600.56	30711.78	43267.89	26383.11	0.475393884	0.32294641	45443.09	40876.156	Down	-0.152801405919029	ATPase family AAA domain-containing protein 3 OS=Mus musculus GN=Atad3 PE=1 SV=1
PRRC2A	Q7TSC1	3	3	15121.67	16634	16512.33	16711	20790	18176.33	14243.67	14359.33	22623.33	3752.67	0.476484645	0.32195109	17153.8	14631.066	Down	-0.229493319049195	Protein PRRC2A OS=Mus musculus GN=Prrc2a PE=1 SV=1
POLDIP2	Q91VA6	1	1	12532	15082	NA	21762	13848	16527	14067	9392	15368	NA	0.476717936	0.321738508	15806	13838.5	Down	-0.191784740237219	Polymerase delta-interacting protein 2 OS=Mus musculus GN=Poldip2 PE=2 SV=1
STMN2	P55821	4	4	101443.5	91065.25	106355	104620.75	85163.25	90994.5	91292.75	73441.25	103801.75	103307.75	0.476923512	0.321551266	97729.55	92567.6	Down	-0.07828753102139	Stathmin-2 OS=Mus musculus GN=Stmn2 PE=1 SV=1
SYT2	P46097	3	3	62454.33	92075	110979.33	290619.33	61909.33	141178.33	131199.33	189076.33	95111.67	251154.33	0.478423977	0.320187063	123607.46	161544	Up	0.386161287174194	Synaptotagmin-2 OS=Mus musculus GN=Syt2 PE=1 SV=1
SRA1	Q80VJ2	2	2	27814.5	29701.5	18915	24871	25799	26586	28134	NA	25991	NA	0.478917399	0.319739385	25420.2	26903.667	Up	0.0818274280983658	Steroid receptor RNA activator 1 OS=Mus musculus GN=Sra1 PE=1 SV=3
ERO1L	Q8R180	1	1	NA	NA	NA	3369	1857	2380	1148	NA	NA	NA	0.478969088	0.319692514	2613	1764	Down	-0.5668565637625	ERO1-like protein alpha OS=Mus musculus GN=Ero1l PE=1 SV=2
LIN7C	O88952	14	14	59132.5	62982.29	64702.57	41545.43	52912.71	56152	55838.21	42692	71017.14	18873.21	0.479117407	0.319558051	56255.1	48914.512	Down	-0.201721346047274	Protein lin-7 homolog C OS=Mus musculus GN=Lin7c PE=1 SV=2
GJA1	P23242	3	3	15305	16487	36124.33	16143	23933.67	19766.33	31426.33	22572	35745.67	17952	0.479545448	0.319170228	21598.6	25492.466	Up	0.23913313709379	Gap junction alpha-1 protein OS=Mus musculus GN=Gja1 PE=1 SV=2
DNAJC3	Q91YW3	3	3	2289	2137.5	NA	8339.5	3807	5468.5	4785	NA	5703	NA	0.480011903	0.318747993	4143.25	5318.8333	Up	0.36034695666694	DnaJ homolog subfamily C member 3 OS=Mus musculus GN=Dnajc3 PE=1 SV=1
HNRNPAB	Q99020	2	2	24566	18385.5	36680	16658	16688	20032.5	16815	30084	31882	32811	0.480570307	0.318243066	22595.5	26324.9	Up	0.220392569166899	Heterogeneous nuclear ribonucleoprotein A/B OS=Mus musculus GN=Hnrnpab PE=1 SV=1
RPS28	P62858	12	12	27138.3	24655.3	14461.4	39226.1	25757.8	28542.5	28170.4	11724.9	30436.6	10432.7	0.480584978	0.318229808	26247.78	21861.42	Down	-0.263808293092108	40S ribosomal protein S28 OS=Mus musculus GN=Rps28 PE=1 SV=1
IBA57	Q8CAK1	1	1	53608	37226	16896	44287	37191	47681	44819	24792	64551	NA	0.481589168	0.31732329	37841.6	45460.75	Up	0.264648396279263	Putative transferase CAF17 homolog, mitochondrial OS=Mus musculus GN=Iba57 PE=1 SV=1
CAMKK1	Q8VBY2	1	1	49595	55362	62244	64557	64305	69742	72920	43488	75353	NA	0.481748294	0.317179814	59212.6	65375.75	Up	0.142851388984354	Calcium/calmodulin-dependent protein kinase kinase 1 OS=Mus musculus GN=Camkk1 PE=1 SV=1
FUBP1	Q91WJ8	2	2	89961	71297.5	68651	23884.5	68910	56279.5	62557.5	34694.5	79921.5	38668	0.481988763	0.316963087	64540.8	54424.2	Down	-0.245963164616245	Far upstream element-binding protein 1 OS=Mus musculus GN=Fubp1 PE=1 SV=1
MPP6	Q9JLB0	3	3	32616.33	28226.33	9290.33	31868.33	25557	38371.33	28972.33	NA	23136.67	NA	0.482382189	0.316608736	25511.664	30160.11	Up	0.241484688525351	MAGUK p55 subfamily member 6 OS=Mus musculus GN=Mpp6 PE=1 SV=1
RPRD1A	Q8VDS4	1	1	38591	36549	34989	30192	34797	35378	29493	18655	37635	38947	0.482388815	0.31660277	35023.6	32021.6	Down	-0.129281987143257	Regulation of nuclear pre-mRNA domain-containing protein 1A OS=Mus musculus GN=Rprd1a PE=2 SV=1
HK2	O08528	1	1	29447	51090	40440	21281	44114	30087	42092	NA	28771	114648	0.482851586	0.316186338	37274.4	53899.5	Up	0.532086759978398	Hexokinase-2 OS=Mus musculus GN=Hk2 PE=2 SV=1

9430020K01RIK	Q5DTX6	4	4	7722.75	12254.75	6537.25	7907	10675.75	11222.75	12372	4563.75	11454.75	12152	0.482934429	0.316111832	9019.5	10353.05	Up	0.198936482744312	Junctional protein associated with coronary artery disease OS=Mus musculus GN=Jcad PE=1 SV=2
VARS	Q9Z1Q9	3	3	3286	25508.5	NA	45699	18309	33033.5	41091.5	NA	19949	NA	0.482978048	0.316072608	23200.625	31358	Up	0.434669877370484	Valine--tRNA ligase OS=Mus musculus GN=Vars PE=2 SV=1
PRKAR2B	P31324	26	26	31626.92	32942.58	27862.42	35136.67	31399.29	35097.79	33672.54	26602	32949.33	17585.5	0.483271226	0.315809062	31793.576	29181.432	Down	-0.123684612165267	cAMP-dependent protein kinase type II-beta regulatory subunit OS=Mus musculus GN=Prkar2b PE=1 SV=3
ATAD3A	Q925I1-2	8	8	46917	52449.5	41258.38	50856.25	54588.62	60777.88	51563.25	33435.88	47185.88	29681	0.483675606	0.315445815	49213.95	44528.778	Down	-0.1443292938603	Isoform 2 of ATPase family AAA domain-containing protein 3 OS=Mus musculus GN=Atad3
NDUFS1	Q91VD9	41	41	93183.92	98177.82	80398.85	79417.1	87098.72	102102.08	86472.95	68500.95	85597.13	68497.13	0.483739169	0.315388746	87655.282	82234.048	Down	-0.0921051808367128	NADH-ubiquinone oxidoreductase 75 kDa subunit, mitochondrial OS=Mus musculus GN=Ndufs1 PE=1 SV=2
RPL31	P62900	9	9	102020.44	116825.44	95491.89	117184.67	98533.22	105407.22	113872.44	79162.22	128295.89	44948	0.484464964	0.314737625	106011.13	94337.154	Down	-0.168317784182444	60S ribosomal protein L31 OS=Mus musculus GN=Rpl31 PE=1 SV=1
RPS17	P63276	6	6	292105	350990.67	362326.5	258195.83	318531.67	247215.5	300216.33	401409	359580	407433.67	0.484563632	0.314649184	316429.93	343170.9	Up	0.117041137257642	40S ribosomal protein S17 OS=Mus musculus GN=Rps17 PE=1 SV=2
NDUFS8	Q8K3J1	36	36	48957.39	49101.75	42008.11	49472.39	51020.25	51227.22	46745.14	40090.31	48284.22	45335.5	0.484655919	0.314566479	48111.978	46336.478	Down	-0.0542477230971838	NADH dehydrogenase [ubiquinone] iron-sulfur protein 8, mitochondrial OS=Mus musculus GN=Ndufs8 PE=1 SV=1
PEX5L	Q8C437	6	6	12840.83	15128.67	10845	17362.67	17183.67	17074.17	14645.5	18701.17	19275.5	10928.5	0.484798587	0.314438655	14672.168	16124.968	Up	0.13621423415044	PEX5-related protein OS=Mus musculus GN=Pex5l PE=1 SV=2
BCKDHA	P50136	3	3	11415	14540.67	6510.33	12404	12255	14134	11297	3128	13011.67	6423.33	0.485372844	0.313924526	11425	9598.8	Down	-0.251268202489019	2-oxoisovalerate dehydrogenase subunit alpha, mitochondrial OS=Mus musculus GN=Bckdha PE=1 SV=1
NCL	P09405	12	12	20620.75	20775.92	16767.58	22360.25	20776.75	21738.75	21683.33	16518.25	21509.58	11562.42	0.486092484	0.313281094	20260.25	18602.466	Down	-0.123158094193723	Nucleolin OS=Mus musculus GN=Ncl PE=1 SV=2
SLIRP	Q9D8T7-2	5	5	82908.6	87369.8	74513	93965.6	85507.8	93521.6	79554	91483.6	105504.2	76644.2	0.486162513	0.313218531	84852.96	89341.52	Up	0.074365812510694	Isoform 2 of SRA stem-loop-interacting RNA-binding protein, mitochondrial OS=Mus musculus GN=Slirp
MUT	P16332	5	5	7608.2	10064.4	9977.2	13014	11647	14732.8	14317.4	13213.2	12543.4	4957	0.486295794	0.313099487	10462.16	11952.76	Up	0.192163049225573	Methylmalonyl-CoA mutase, mitochondrial OS=Mus musculus GN=Mut PE=1 SV=2
GRIN1	P35438-2	3	3	17989.67	18758.33	48586.33	17750.67	35232.67	26305	32209.67	60358.67	33847.33	19545.33	0.486627003	0.312803796	27663.534	34453.2	Up	0.316652514420383	Isoform 2 of Glutamate receptor ionotropic, NMDA 1 OS=Mus musculus GN=Grin1
EIF5	P59325	10	10	24354.6	33382.2	32891.3	55420.8	36159	39666.2	37666.4	25375.2	35301.5	21145.8	0.486860071	0.312595842	36441.58	31831.02	Down	-0.195152123778129	Eukaryotic translation initiation factor 5 OS=Mus musculus GN=Eif5 PE=1 SV=1
PVALB	P32848	4	4	88804.5	76745.75	124131.25	66363	82330.25	77388.5	64525.5	103856.25	63680.5	83986.25	0.487092012	0.312388993	87674.95	78687.4	Down	-0.156032063344522	Parvalbumin alpha OS=Mus musculus GN=Pvalb PE=1 SV=3
MCTS2	Q9CQ21	1	1	33931	28182	20201	106064	43681	49628	31759	NA	18072	NA	0.487541703	0.31198823	46411.8	33153	Down	-0.485352225914152	Malignant T-cell-amplified sequence 2 OS=Mus musculus GN=Mcts2 PE=2 SV=1
PNN	O35691	1	1	92511	44879	48075	30441	63976	53092	47024	9074	66817	NA	0.487580577	0.311953603	55976.4	44001.75	Down	-0.34725780358607	Pinin OS=Mus musculus GN=Pnn PE=1 SV=4
MYL6B	Q8CI43	4	4	27971	34640.5	45815	19023.75	30288.5	25289.75	31621	43224.5	37876.5	39492.25	0.487641747	0.311899121	31547.75	35500.8	Up	0.170314420483716	Myosin light chain 6B OS=Mus musculus GN=Myl6b PE=2 SV=1
FMR1	P35922-11	4	4	22320.25	22891.75	20963.5	24199.75	22435	23339	22253	19976.75	27696.25	3885.75	0.487900332	0.311668886	22562.05	19430.15	Down	-0.215601119224065	Isoform ISO11 of Fragile X mental retardation protein 1 homolog OS=Mus musculus GN=Fmr1
FMR1	P35922-3	5	5	22320.25	22891.75	20963.5	24199.75	22435	23339	22253	19976.75	27696.25	3885.75	0.487900332	0.311668886	22562.05	19430.15	Down	-0.215601119224065	Isoform ISO3 of Fragile X mental retardation protein 1 homolog OS=Mus musculus GN=Fmr1
SEMA4A	Q62178	2	2	NA	6193	NA	9943.5	6214.5	7568.5	10391.5	NA	NA	NA	0.488556804	0.311084934	7450.3333	8980	Up	0.269410470721968	Semaphorin-4A OS=Mus musculus GN=Sema4a PE=1 SV=2
HNRNPA3	Q8BG05	26	26	129054.77	71885.12	80328.15	61751.81	84704.88	73789.85	76763.31	84177.88	70809.19	77704.77	0.489301925	0.310423076	85544.946	76649	Down	-0.158415652823825	Heterogeneous nuclear ribonucleoprotein A3 OS=Mus musculus GN=Hnrnpa3 PE=1 SV=1
MAPT	P10637-3	47	47	125329.89	116889.87	115222.72	113980.67	115867.04	107409.15	117104.67	131319.22	158282.09	109363.52	0.489722871	0.310049613	117458.04	124695.73	Up	0.0862666186912359	Isoform Tau-B of Microtubule-associated protein tau OS=Mus musculus GN=Mapt
FCHO2	Q3UQN2-3	1	1	8420	8366	6279	6589	9702	8890	9913	NA	7136	NA	0.490199397	0.309627227	7871.2	8646.3333	Up	0.135504858189927	Isoform 3 of FCH domain only protein 2 OS=Mus musculus GN=Fcho2
PSMD1	Q3TXS7	2	2	604	1526	884	7353	3510	5478	5048	NA	1659	NA	0.49021358	0.309614662	2775.4	4061.6667	Up	0.549376132709169	26S proteasome non-ATPase regulatory subunit 1 OS=Mus musculus GN=Psmd1 PE=1 SV=1
LDHD	Q7TNG8	2	2	32106	36202.5	44769.5	22149.5	31837	23563.5	33734.5	39260.5	68466	NA	0.490403018	0.309446866	33412.9	41256.125	Up	0.304203118124419	Probable D-lactate dehydrogenase, mitochondrial OS=Mus musculus GN=Ldhd PE=1 SV=1
KRT31	Q61765	1	1	25219	19812	18883	45959	22558	30421	21920	31157	40810	NA	0.490942601	0.308969281	26486.2	31077	Up	0.230606367005031	Keratin, type I cuticular Ha1 OS=Mus musculus GN=Krt31 PE=2 SV=2
PPM1H	Q3UYC0	4	4	36019	31843.5	52420	45761.25	38853.5	33632.5	39854.75	10472	42625	50186.25	0.491194391	0.308746601	40979.45	35354.1	Down	-0.213023088078349	Protein phosphatase 1H OS=Mus musculus GN=Ppm1h PE=1 SV=1
MRPS33	Q9D2R8	1	1	37201	64007	27664	49123	52511	47014	38254	76269	27831	89736	0.491295443	0.308657264	46101.2	55820.8	Up	0.275998496595016	28S ribosomal protein S33, mitochondrial OS=Mus musculus GN=Mrps33 PE=2 SV=1
ACYP2	P56375	3	3	42601	45081.67	35520	75141	45201	50594.67	45361.67	48460	47805.33	18632.67	0.491478013	0.308495906	48708.934	42170.868	Down	-0.207939693182529	Acylphosphatase-2 OS=Mus musculus GN=Acyp2 PE=2 SV=2
COX5A	P12787	35	35	435637.36	419585.64	486172.88	200925.94	347049.24	284301.42	303524.76	508986.15	433518.94	674983.36	0.491649886	0.308344057	377874.21	441062.93	Up	0.223078432972082	Cytochrome c oxidase subunit 5A, mitochondrial OS=Mus musculus GN=Cox5a PE=1 SV=2
RPL21	O09167	5	5	283129.8	320137	312330	221912.8	282532.4	239093	301118.2	429887.4	281938.2	300583.8	0.491685588	0.308312522	284008.4	310524.12	Up	0.128771733955887	60S ribosomal protein L21 OS=Mus musculus GN=Rpl21 PE=2 SV=3
DIP2B	Q3UH60-2	1	1	407	1258	NA	3843	1487	2635	3209	2945	874	NA	0.491705736	0.308294726	1748.75	2415.75	Up	0.466147104051855	Isoform 2 of Disco-interacting protein 2 homolog B OS=Mus musculus GN=Dip2b
UBC	P0CG50	9	9	78331.89	128544	64160.22	63749.44	107032.89	83988.11	140445.33	105119.44	180339.22	34066	0.492046678	0.307993696	88363.688	108791.62	Up	0.300041894716535	Polyubiquitin-C OS=Mus musculus GN=Ubc PE=1 SV=2
TAB3	Q571K4	1	1	6192	7872	6550	8079	7539	8203	6319	NA	9756	NA	0.492067327	0.307975471	7246.4	8092.6667	Up	0.15935072822263	TGF-beta-activated kinase 1 and MAP3K7-binding protein 3 OS=Mus musculus GN=Tab3 PE=1 SV=2
CACNA1B	O55017	7	7	23487.71	42687.86	38383.86	47189.86	41878.57	52741.43	59245.57	28152.71	49233.43	30960	0.49250418	0.307590079	38725.572	44066.628	Up	0.186399956460318	Voltage-dependent N-type calcium channel subunit alpha-1B OS=Mus musculus GN=Cacna1b PE=1 SV=1
RBM4B	Q8VE92	1	1	4719	NA	NA	4754	5356	4979	2431	NA	5194	NA	0.493810135	0.306440001	4943	4201.3333	Down	-0.23453965284339	RNA-binding protein 4B OS=Mus musculus GN=Rbm4b PE=1 SV=1
CDH11	P55288	3	3	4029.33	5098	1303	12585.67	5638.33	9400.33	6520.67	NA	5978.67	NA	0.493965221	0.306303628	5730.866	7299.89	Up	0.349121561260226	Cadherin-11 OS=Mus musculus GN=Cdh11 PE=1 SV=1
IMPA1	O55023	4	4	68449.25	81267	89099.25	90091.25	81347.25	94461.25	79792.25	67488.25	81863.75	61609.25	0.494103926	0.306181695	82050.8	77042.95	Down	-0.0908544546582629	Inositol monophosphatase 1 OS=Mus musculus GN=Impa1 PE=1 SV=1
FXYD6	Q9D164	1	1	3216	4214	3426	9472	4199	8444	6023	3411	6217	NA	0.494107435	0.306178611	4905.4	6023.75	Up	0.296291113345941	FXYD domain-containing ion transport regulator 6 OS=Mus musculus GN=Fxyd6 PE=1 SV=2
RANBP9	P69566	2	2	11700.5	19244.5	10170	18511.5	12077	15303.5	10371.5	14436	12415.5	41432	0.4942456	0.306057189	14340.7	18791.7	Up	0.389980139983756	Ran-binding protein 9 OS=Mus musculus GN=Ranbp9 PE=1 SV=1
HNRNPM	Q9D0E1	19	19	126117.16	82616.63	111116.21	39655.58	86933.11	58988.53	70002	74441.32	85349	98557.26	0.49535639	0.30508223	89287.738	77467.622	Down	-0.204868607400264	Heterogeneous nuclear ribonucleoprotein M OS=Mus musculus GN=Hnrnpm PE=1 SV=3
PCSK2	P21661	4	4	32085	36814.25	28457	30063.75	30467.5	30305.75	35964	24712	37488.75	10941.25	0.495669855	0.304807493	31577.5	27882.35	Down	-0.179544796549321	Neuroendocrine convertase 2 OS=Mus musculus GN=Pcsk2 PE=1 SV=1
PPRC1	Q6NZN1-3	1	1	23353	35915	11521	45178	40289	51351	54308	27452	16832	39746	0.496138933	0.304396691	31251.2	37937.8	Up	0.279724431584169	Isoform 3 of Peroxisome proliferator-activated receptor gamma coactivator-related protein 1 OS=Mus musculus GN=Pprc1
PDAP1	Q3UHX2	6	6	14288.5	16104.17	12595.67	31192.5	16072	20569.83	17660.5	8317	22268.5	3972.33	0.496443788	0.304129919	18050.568	14557.632	Down	-0.310268534995809	28 kDa heat- and acid-stable phosphoprotein OS=Mus musculus GN=Pdap1 PE=1 SV=1
NMRAL1	Q8K2T1	1	1	65334	66931	204620	36490	94901	98127	73795	255370	108789	89254	0.497277333	0.303401336	93655.2	125067	Up	0.41727016797368	NmrA-like family domain-containing protein 1 OS=Mus musculus GN=Nmral1 PE=1 SV=1
ERH	P84089	4	4	62434.25	43108.25	16740.75	40666.75	50498.25	42835.75	43392.5	27089	32901.5	NA	0.497713487	0.30302059	42689.65	36554.688	Down	-0.223829915924872	Enhancer of rudimentary homolog OS=Mus musculus GN=Erh PE=1 SV=1
PPP1R14B	Q62084	2	2	5957	7604	NA	5923	9702	7192	7282	NA	10607	NA	0.497773267	0.302968431	7296.5	8360.3333	Up	0.196355869866496	Protein phosphatase 1 regulatory subunit 14B OS=Mus musculus GN=Ppp1r14b PE=1 SV=2
EEF1G	Q9D8N0	4	4	22123.5	33677.5	41164	57071.25	30111.5	43613.5	38855.25	48249.5	35000.75	41466.75	0.498051082	0.302726112	36829.55	41437.15	Up	0.170061009665578	Elongation factor 1-gamma OS=Mus musculus GN=Eef1g PE=1 SV=3
ADH5	P28474	4	4	95444.5	109542.75	216891.25	116044.75	131058	128984	151684.25	186088.5	160663.75	128410.5	0.498526316	0.302311912	133796.25	151166.2	Up	0.176097915146742	Alcohol dehydrogenase class-3 OS=Mus musculus GN=Adh5 PE=1 SV=3
CCDC25	Q78PG9	1	1	2529	2615	1858	2802	2176	2599	2482	4798	1756	NA	0.498544092	0.302296426	2396	2908.75	Up	0.279771397503925	Coiled-coil domain-containing protein 25 OS=Mus musculus GN=Ccdc25 PE=1 SV=1
ADD2	Q9QYB8	20	20	35576.9	39241.5	28946.7	58035.75	37972.85	46117.95	42873.1	23579.4	38109.4	25486.8	0.498706337	0.302155113	39954.74	35233.33	Down	-0.18142583193115	Beta-adducin OS=Mus musculus GN=Add2 PE=1 SV=4
HNRNPA2B1	O88569	35	35	116897.21	70111.09	78805.79	43509.42	82638.48	68684.73	66873.88	83876.64	69207.94	57658.27	0.498827778	0.30204937	78392.398	69260.292	Down	-0.17868528774246	Heterogeneous nuclear ribonucleoproteins A2/B1 OS=Mus musculus GN=Hnrnpa2b1 PE=1 SV=2
MAP2K4	P47809	3	3	21905.33	29240.67	26771.67	58861.67	46712	62850.33	64828.67	16016.33	41879.33	NA	0.499940322	0.301951397	36698.268	46393.665	Up	0.338215844812256	Dual specificity mitogen-activated protein kinase kinase 4 OS=Mus musculus GN=Map2k4 PE=1 SV=2
RAPGEF4	Q9EQZ6	1	1	5671	9931	NA	15408	8423	13843	13507	NA	8212	NA	0.499759762	0.301238714	9858.25	11854	Up	0.265970490050151	Rap guanine nucleotide exchange factor 4 OS=Mus musculus GN=Rapgef4 PE=1 SV=1
MTDH	Q80WJ7	8	8	8106.38	25595.5	4244.12	45564.12	25805.62	42375.62	39170.25	11299.38	23911.12	NA	0.500707925	0.300415535	21863.148	29189.093	Up	0.416928212901997	Protein LYRIC OS=Mus musculus GN=Mtdh PE=1 SV=1
OXR1	Q4KMM3-2	15	15	26997.75	31264	20104.25	38379.67	35236.08	35560.08	32354.92	12724.33	30175.33	22861.75	0.501381659	0.299831557	30396.35	26735.282	Down	-0.185153200788702	Isoform 2 of Oxidation resistance protein 1 OS=Mus musculus GN=Oxr1
GIGYF1	Q99MR1	1	1	46306	43312	NA	47346	42101	38702	43743	61865	50244	NA	0.501394846	0.299820134	44766.25	48638.5	Up	0.119687266133417	PERQ amino acid-rich with GYF domain-containing protein 1 OS=Mus musculus GN=Gigyf1 PE=1 SV=2
ACAP2	Q6ZQK5	1	1	9621	11986	3278	21828	22305	21257	21659	NA	13835	11245	0.502099846	0.299209912	13803.6	16999	Up	0.300405306302471	Arf-GAP with coiled-coil, ANK repeat and PH domain-containing protein 2 OS=Mus musculus GN=Acap2 PE=1 SV=2
H2-EA-PS	P04224	1	1	7953	10974	NA	3877	9828	8235	3018	NA	NA	NA	0.502214465	0.299110783	8158	5626.5	Down	-0.535977743409897	H-2 class II histocompatibility antigen, E-K alpha chain OS=Mus musculus PE=1 SV=1
GLRX5	Q80Y14	5	5	25237.6	37223.4	25916.4	52381.4	34704.4	45693.4	40379.8	8092	33958.6	16664	0.502242467	0.299086569	35092.64	28957.56	Down	-0.277228440711972	Glutaredoxin-related protein 5, mitochondrial OS=Mus musculus GN=Glrx5 PE=1 SV=2
DNAJA3	Q99M87-2	8	8	35245.62	48990.75	16157.88	57509.12	40456.25	45184	45735.12	18492.88	40260.75	14560.75	0.502749294	0.298648531	39671.924	32846.7	Down	-0.272369931679103	Isoform 2 of DnaJ homolog subfamily A member 3, mitochondrial OS=Mus musculus GN=Dnaja3
KAZN	Q69ZS8	6	6	11470	13297.83	4069.67	15218.5	12253.5	12234.67	11330.17	7352.5	12714.5	3636.5	0.502756271	0.298642504	11261.9	9453.668	Down	-0.252504140448659	Kazrin OS=Mus musculus GN=Kazn PE=2 SV=2
PPA1	Q9D819	8	8	59725.12	70790.5	71381.75	90770.62	52143.25	64323.88	70203.25	68094	60678.62	56357.25	0.503171818	0.298283691	68962.248	63931.4	Down	-0.109282116505149	Inorganic pyrophosphatase OS=Mus musculus GN=Ppa1 PE=1 SV=1
HIST1H1E	P43274	18	18	245626.11	137847.28	160800.83	77241.44	170718.61	110946.72	101468.61	170210.56	113733.17	182787.28	0.503299078	0.298173865	158446.85	135829.27	Down	-0.222204635155585	Histone H1.4 OS=Mus musculus GN=Hist1h1e PE=1 SV=2
SORCS2	Q9EPR5	3	3	18730.5	29281	29506	12095	22129	18457.5	24116	27199	31834.5	NA	0.503517987	0.297985011	22348.3	25401.75	Up	0.184762799812081	VPS10 domain-containing receptor SorCS2 OS=Mus musculus GN=Sorcs2 PE=1 SV=2
SFN	O70456	4	4	740799.25	1005789	606064.5	2555605	1079251.5	1615794	1283879	513034.25	922005.75	105821.25	0.504355038	0.297263637	1197501.9	888106.85	Down	-0.431222718957767	14-3-3 protein sigma OS=Mus musculus GN=Sfn PE=1 SV=2
SYNJ1	Q8CHC4	10	10	26843.5	23347.6	27650.4	28062.8	29027.3	27294.6	30675.1	28284.4	24902.1	10011.8	0.504392564	0.297231325	26986.32	24233.6	Down	-0.155219517613061	Synaptojanin-1 OS=Mus musculus GN=Synj1 PE=1 SV=3
VIM	P20152	19	19	17719.53	23633.32	10909.95	32056.26	21361.11	22641	21210.21	7926.84	30131.84	1919.05	0.504800994	0.296879799	21136.034	16765.788	Down	-0.334184400380324	Vimentin OS=Mus musculus GN=Vim PE=1 SV=3
HNRNPA3	Q8BG05-2	24	24	134544.33	74950.21	86131.25	65362.62	88251.71	76438.25	80594.58	90930.92	72563.71	84180.17	0.505104136	0.296619076	89848.024	80941.526	Down	-0.150606726774051	Isoform 2 of Heterogeneous nuclear ribonucleoprotein A3 OS=Mus musculus GN=Hnrnpa3
DCX	O88809	12	12	23963.5	28765.83	26149.17	34561.17	21957	26384.33	30010.83	34302.92	38521.83	19838.08	0.505182278	0.296551893	27079.334	29811.598	Up	0.138681453486559	Neuronal migration protein doublecortin OS=Mus musculus GN=Dcx PE=1 SV=1
TSFM	Q9CZR8	13	13	22384.75	24783.83	55348.92	13086.33	24019.75	22010.17	21772.75	49367.67	28595.25	51917.92	0.505338165	0.296417901	27924.716	34732.752	Up	0.314754116846626	Elongation factor Ts, mitochondrial OS=Mus musculus GN=Tsfm PE=1 SV=1
CACNA2D1	O08532-3	24	24	25183.65	36634.35	16878.3	76583.3	31975.61	54347.7	55712.43	47306.91	35340.57	34707.74	0.505381066	0.296381033	37451.042	45483.07	Up	0.280323776697458	Isoform 2C of Voltage-dependent calcium channel subunit alpha-2/delta-1 OS=Mus musculus GN=Cacna2d1
SF1	Q64213-3	7	7	27569.43	26280.29	12082.43	22625.43	28038	28838.43	26732	7265.14	28096.71	3301.71	0.505420874	0.296346826	23319.116	18846.798	Down	-0.307193662859908	Isoform 3 of Splicing factor 1 OS=Mus musculus GN=Sf1
MRPL55	Q9CZ83	4	4	47768.75	40979.5	62283.5	18560.5	37185.75	31541.75	33082.25	78040	66527.75	39124.75	0.505509915	0.296270322	41355.6	49663.3	Up	0.264097428824088	39S ribosomal protein L55, mitochondrial OS=Mus musculus GN=Mrpl55 PE=2 SV=1
TOM1L2	Q5SRX1-5	2	2	33307.5	50222	30854	62441.5	42747	54764	50665.5	9996.5	44680.5	23099.5	0.505713943	0.296095072	43914.4	36641.2	Down	-0.261227339928793	Isoform 5 of TOM1-like protein 2 OS=Mus musculus GN=Tom1l2
PCP4L1	Q6W8Q3	2	2	63396.5	63801	69437	58128	50885	52662.5	45965.5	121568.5	73547.5	61805.5	0.505761833	0.296053948	61129.5	71109.9	Up	0.218181660615209	Purkinje cell protein 4-like protein 1 OS=Mus musculus GN=Pcp4l1 PE=1 SV=1
SLC25A3	Q8VEM8	6	6	14684.83	17913.83	27187.67	24646.5	15428.83	16864.17	19361.5	28655.83	23755.17	22463.33	0.505953933	0.295889023	19972.332	22220	Up	0.153856022832775	Phosphate carrier protein, mitochondrial OS=Mus musculus GN=Slc25a3 PE=1 SV=1
DNAJA3	Q99M87	17	17	20573.4	28151.73	9062.73	33128.8	23629.67	26597.4	26207.2	11141.27	23080.07	7765.73	0.505964782	0.295879711	22909.266	18958.334	Down	-0.273099046035547	DnaJ homolog subfamily A member 3, mitochondrial OS=Mus musculus GN=Dnaja3 PE=1 SV=1
HCN1	O88704	3	3	21187.67	25533	4070	61964	34987.33	44480	49684.67	NA	21748.33	NA	0.506426341	0.295483714	29548.4	38637.667	Up	0.38692795945701	Potassium/sodium hyperpolarization-activated cyclic nucleotide-gated channel 1 OS=Mus musculus GN=Hcn1 PE=1 SV=1
PTP4A1	Q63739	1	1	30978	38582	NA	100926	62918	73532	68150	NA	69000	NA	0.506491151	0.295428138	58351	70227.333	Up	0.267275273739301	Protein tyrosine phosphatase type IVA 1 OS=Mus musculus GN=Ptp4a1 PE=1 SV=1
RBP1	Q00915	2	2	15050.5	21497.5	NA	23086.5	11364	21631.5	11197	NA	10564	NA	0.506638055	0.295302192	17749.625	14464.167	Down	-0.295305338077757	Retinol-binding protein 1 OS=Mus musculus GN=Rbp1 PE=2 SV=2
GCC2	Q8CHG3	4	4	8290.75	2682.25	NA	12444	9146	12052.5	8045.5	NA	9378.25	NA	0.50748013	0.294580958	8140.75	9825.4167	Up	0.271356874254335	GRIP and coiled-coil domain-containing protein 2 OS=Mus musculus GN=Gcc2 PE=1 SV=2
SERBP1	Q9CY58-3	7	7	46121.4	50951.8	41391.8	90393.8	40568.6	59598.6	54021.2	34191.2	66707.2	4145.6	0.507937125	0.294190044	53885.48	43732.76	Down	-0.30118217562388	Isoform 3 of Plasminogen activator inhibitor 1 RNA-binding protein OS=Mus musculus GN=Serbp1
SNRPN	P63163	1	1	129351	86355	149727	59369	89910	69701	79096	190044	103777	175795	0.508009972	0.294127763	102942.4	123682.6	Up	0.264805229326793	Small nuclear ribonucleoprotein-associated protein N OS=Mus musculus GN=Snrpn PE=2 SV=1
EIF4G3	Q80XI3	2	2	7679	10639.5	11059	28553	8438	30721	25884	5863	10709.5	NA	0.508743233	0.293501355	13273.7	18294.375	Up	0.462829555756455	Eukaryotic translation initiation factor 4 gamma 3 OS=Mus musculus GN=Eif4g3 PE=1 SV=2
MAPT	P10637-4	51	51	119426.18	111005.29	109884.63	107417.08	110180.73	101768.02	111177.39	124163.24	150242.47	103481.76	0.508970158	0.29330768	111582.78	118166.58	Up	0.0827075933417081	Isoform Tau-C of Microtubule-associated protein tau OS=Mus musculus GN=Mapt
OPALIN	Q7M750	2	2	7241	11367	2738	13782.5	10039.5	16847.5	8995	NA	8448	NA	0.509502023	0.292854087	9033.6	11430.167	Up	0.339473501059234	Opalin OS=Mus musculus GN=Opalin PE=1 SV=1
RANBP1	P34022	9	9	111218.75	95887.5	62007.12	126537.75	104991.38	101577.12	101722.38	21037.88	142439.62	49915.38	0.51005664	0.292381594	100128.5	83338.476	Down	-0.264798050024359	Ran-specific GTPase-activating protein OS=Mus musculus GN=Ranbp1 PE=1 SV=2
H2-KE6	P50171	4	4	13239.33	17211	15035	12057.67	17624	16294.67	15163.33	12561.67	15507.67	22444.33	0.510807683	0.291742579	15033.4	16394.334	Up	0.125025964673354	Estradiol 17-beta-dehydrogenase 8 OS=Mus musculus GN=Hsd17b8 PE=1 SV=2
CADPS2	Q8BYR5-3	2	2	30447.5	34923.5	37184.5	48486	47191.5	38605	61055.5	27746	54260.5	40513	0.510839719	0.291715343	39646.6	44436	Up	0.164531806273117	Isoform 3 of Calcium-dependent secretion activator 2 OS=Mus musculus GN=Cadps2
ALYREF2	Q9JJW6	3	3	12835	11264	9092.5	11403.5	11080.5	9024.5	12460.5	11547	7430.5	11384.5	0.510943578	0.291627055	11135.1	10369.4	Down	-0.102782095924701	Aly/REF export factor 2 OS=Mus musculus GN=Alyref2 PE=1 SV=1
RPL37	Q9D823	3	3	281021.67	244028	366275	173962.67	250235.67	267117.33	226940	308235	415332.33	253702.67	0.511869665	0.290840607	263104.6	294265.47	Up	0.161481759292405	60S ribosomal protein L37 OS=Mus musculus GN=Rpl37 PE=2 SV=3
RPL37A	P61514	11	11	167837.82	186460.45	224697.36	115347.18	167710.64	122306.73	187528.27	197801	219120.82	221822.73	0.512316825	0.290461382	172410.69	189715.91	Up	0.1379914426635	60S ribosomal protein L37a OS=Mus musculus GN=Rpl37a PE=2 SV=2
SEC31A	Q3UPL0	2	2	50696	45274.5	104717	47238.5	62426	58413.5	50070.5	54023	57379.5	198951.5	0.512806584	0.290046407	62070.4	83767.6	Up	0.43248689678957	Protein transport protein Sec31A OS=Mus musculus GN=Sec31a PE=1 SV=2
UBE2N	P61089	7	7	30954.57	35457.43	28064	47919	28333.57	35890.71	33620.43	30759.57	39313.43	8345.71	0.512914019	0.289955431	34145.714	29585.97	Down	-0.206791305742172	Ubiquitin-conjugating enzyme E2 N OS=Mus musculus GN=Ube2n PE=1 SV=1
LAMTOR1	Q9CQ22	5	5	28918.4	28552.6	28197.4	24443.6	33832.4	32826.2	25971.8	22722.2	33139.4	17746.4	0.512961931	0.289914864	28788.88	26481.2	Down	-0.120543163921761	Ragulator complex protein LAMTOR1 OS=Mus musculus GN=Lamtor1 PE=1 SV=1
TRIM2	Q9ESN6	9	9	25981.75	32674.75	27655.62	35037.38	30407.62	27202.88	31639.88	33837.75	32662.5	9625.75	0.513016931	0.289868302	30351.424	26993.752	Down	-0.169138686775226	Tripartite motif-containing protein 2 OS=Mus musculus GN=Trim2 PE=1 SV=1
ADAM22	Q9R1V6-11	5	5	41218.25	74697.5	15686.5	132766.75	79637.25	128526.5	129025	53233.5	47591.75	NA	0.513261706	0.289661137	68801.25	89594.188	Up	0.380970362791663	Isoform 9 of Disintegrin and metalloproteinase domain-containing protein 22 OS=Mus musculus GN=Adam22
NEDD8	P29595	1	1	14036	16453	NA	54054	13142	23173	18251	12526	14007	NA	0.513376767	0.28956379	24421.25	16989.25	Down	-0.523514881117999	NEDD8 OS=Mus musculus GN=Nedd8 PE=1 SV=2
LSM1	Q8VC85	1	1	27979	34554	41775	29833	26526	30205	33468	NA	42645	NA	0.513750788	0.289247499	32133.4	35439.333	Up	0.14127782603056	U6 snRNA-associated Sm-like protein LSm1 OS=Mus musculus GN=Lsm1 PE=2 SV=1

MAT2B	Q99LB6	1	1	104696	101518	136943	61817	89761	73544	97846	106814	138968	136619	0.513800258	0.289205682	98947	110758.2	Up	0.162685641278905	Methionine adenosyltransferase 2 subunit beta OS=Mus musculus GN=Mat2b PE=2 SV=1
PRPH	P15331-3	1	1	49205	66288	38901	67926	50544	61480	59511	45960	72709	NA	0.514374213	0.288720812	54572.8	59915	Up	0.134735166827768	Isoform 5b of Peripherin OS=Mus musculus GN=Prph
PRPF19	Q99KP6	1	1	8733	10304	NA	24453	19318	27201	19332	NA	12548	NA	0.514408665	0.288691724	15702	19693.667	Up	0.326783414302484	Pre-mRNA-processing factor 19 OS=Mus musculus GN=Prpf19 PE=1 SV=1
SIRT2	Q8VDQ8	4	4	14557.75	14719	4665.25	17862	18453.5	20592	16911.25	10953.25	16468.75	NA	0.514650477	0.28848762	14051.5	16231.313	Up	0.208055517523522	NAD-dependent protein deacetylase sirtuin-2 OS=Mus musculus GN=Sirt2 PE=1 SV=2
ANKFY1	Q810B6	1	1	2879	2927	NA	3716	1968	3361	3449	NA	2708	NA	0.514719767	0.288429153	2872.5	3172.6667	Up	0.14338906090586	Ankyrin repeat and FYVE domain-containing protein 1 OS=Mus musculus GN=Ankfy1 PE=2 SV=2
MPP2	Q9WV34	7	7	57695.57	66997.57	26864.71	67061.43	60389.29	65550.29	61144.43	16367.71	55764.71	40450.43	0.514913644	0.2882656	55801.714	47855.514	Down	-0.221624272160593	MAGUK p55 subfamily member 2 OS=Mus musculus GN=Mpp2 PE=1 SV=1
DNAJC19	Q9CQV7	1	1	335906	307190	319271	257471	254571	294936	329509	278204	312843	107244	0.515022603	0.28817371	294881.8	264547.2	Down	-0.15661163435681	Mitochondrial import inner membrane translocase subunit TIM14 OS=Mus musculus GN=Dnajc19 PE=2 SV=3
FAM81A	Q3UXZ6	1	1	24220	29053	14171	42498	25431	28013	24670	19153	22691	NA	0.515503069	0.287768745	27074.6	23631.75	Down	-0.196213554579474	Protein FAM81A OS=Mus musculus GN=Fam81a PE=2 SV=2
GRN	P28798	1	1	9714	7252	24974	4608	5857	5219	8234	12673	24272	19410	0.515693739	0.287608141	10481	13961.6	Up	0.413687911891196	Granulins OS=Mus musculus GN=Grn PE=1 SV=2
FBXO2	Q80UW2	2	2	66115.5	70745	89520	54591.5	64083	48115	71019.5	121678.5	63331	89000.5	0.515782463	0.287533428	69011	78628.9	Up	0.188233333139592	F-box only protein 2 OS=Mus musculus GN=Fbxo2 PE=1 SV=1
EPRS	Q8CGC7	6	6	4912.33	11594.67	9371.67	26790.17	12146.5	21089.5	20024.83	18311	12454.5	8105.5	0.516123402	0.287246449	12963.068	15997.066	Up	0.303400121964281	Bifunctional glutamate/proline--tRNA ligase OS=Mus musculus GN=Eprs PE=1 SV=4
HSPE1	Q64433	28	28	152935.71	135012.5	146340.61	156416.71	130349.07	132847.07	127853.14	172555.11	140455.36	106523.54	0.517938383	0.285721903	144210.92	136046.84	Down	-0.084076923584396	10 kDa heat shock protein, mitochondrial OS=Mus musculus GN=Hspe1 PE=1 SV=2
EPS15L1	Q60902-3	7	7	17715.57	22029.14	23295	20872.43	20092.57	19791.57	18898.29	20512.14	20569.29	20752.29	0.518163719	0.285532998	20800.942	20104.716	Down	-0.0491149076098011	Isoform 3 of Epidermal growth factor receptor substrate 15-like 1 OS=Mus musculus GN=Eps15l1
UBB	P0CG49	7	7	76027.86	121571.14	58365.43	67378.57	103083.71	86209.14	136091.71	93737.29	174830.71	29199.43	0.518376358	0.285354814	85285.342	104013.66	Up	0.286403241061411	Polyubiquitin-B OS=Mus musculus GN=Ubb PE=2 SV=1
VPS51	Q3UVL4	2	2	8495	13190	3715.5	21905	14570.5	20021	16494	NA	9576.5	NA	0.518804191	0.284996524	12375.2	15363.833	Up	0.312086377441905	Vacuolar protein sorting-associated protein 51 homolog OS=Mus musculus GN=Vps51 PE=2 SV=2
NGEF	Q8CHT1	3	3	NA	3928	NA	16284	4772.5	6793	3451	NA	NA	NA	0.519331272	0.284555525	8328.1667	5122	Down	-0.701291687792184	Ephexin-1 OS=Mus musculus GN=Ngef PE=1 SV=1
RAC1	P63001	7	7	36394.57	52266.86	53484.57	152707.43	43815	81994.86	77508.86	117233.14	51928.43	91943.57	0.51980997	0.284155395	67733.686	84121.772	Up	0.312605732403102	Ras-related C3 botulinum toxin substrate 1 OS=Mus musculus GN=Rac1 PE=1 SV=1
MCEE	Q9D1I5	1	1	50009	52340	77698	6806	35499	21848	44201	26979	50423	202394	0.519831373	0.284137513	44470.4	69169	Up	0.637280217610384	Methylmalonyl-CoA epimerase, mitochondrial OS=Mus musculus GN=Mcee PE=1 SV=1
AK3	Q9WTP7	18	18	58501.61	59084.06	75691.28	36717.67	56439.61	51764.67	52394.61	71793.72	56920.17	82841.83	0.51984566	0.284125577	57286.846	63143	Up	0.140418895908296	GTP:AMP phosphotransferase AK3, mitochondrial OS=Mus musculus GN=Ak3 PE=1 SV=3
ATP8A1	P70704	1	1	1419	2209	NA	5792	3437	4935	4724	NA	2526	NA	0.520327914	0.283722875	3214.25	4061.6667	Up	0.337589700989344	Probable phospholipid-transporting ATPase IA OS=Mus musculus GN=Atp8a1 PE=1 SV=1
TMEM87A	Q8BXN9	1	1	3462	20445	7210	54258	15574	31131	33460	NA	17209	NA	0.520861424	0.283277806	20189.8	27266.667	Up	0.433511723031425	Transmembrane protein 87A OS=Mus musculus GN=Tmem87a PE=1 SV=1
QDPR	Q8BVI4	10	10	122488.3	116361.2	169223.9	68176.5	108104.8	100359.2	110242.3	154991.3	127224.6	158730.2	0.521019502	0.28314602	116870.94	130309.52	Up	0.1570262386419	Dihydropteridine reductase OS=Mus musculus GN=Qdpr PE=1 SV=2
CYCT	P00015	1	1	42391	36729	41923	28225	43850	29962	38398	40656	49214	50890	0.522427089	0.281974312	38623.6	41824	Up	0.114848406035766	Cytochrome c, testis-specific OS=Mus musculus GN=Cyct PE=1 SV=3
KCNB2	A6H8H5	4	4	4376.5	7439	4882	7820	8918.5	10292.5	12823	301.5	10233	7733.5	0.522519528	0.281897474	6687.2	8276.7	Up	0.307653400039806	Potassium voltage-gated channel subfamily B member 2 OS=Mus musculus GN=Kcnb2 PE=2 SV=2
CDC42	P60766	4	4	17427	24956.25	15309	58232	18964.5	30706	30157.5	12857	19083.75	11638.5	0.522521922	0.281895484	26977.75	20888.55	Down	-0.369057679827871	Cell division control protein 42 homolog OS=Mus musculus GN=Cdc42 PE=1 SV=2
HPCAL1	P62748	1	1	61294	69876	41292	86467	100967	97389	79748	60258	85155	NA	0.522642445	0.281795323	71979.2	80637.5	Up	0.163870843961117	Hippocalcin-like protein 1 OS=Mus musculus GN=Hpcal1 PE=2 SV=2
PRKCQ	Q02111	1	1	1854	5482	4619	9183	5691	9403	5501	9425	2634	NA	0.523848595	0.280794216	5365.8	6740.75	Up	0.329115837873792	Protein kinase C theta type OS=Mus musculus GN=Prkcq PE=1 SV=1
ADGRL1	Q80TR1	16	16	16323.81	27091.69	17755.12	36224.38	29180.94	34973.06	37271.69	16253.81	32027.12	23933.12	0.524067103	0.280613101	25315.188	28891.76	Up	0.19065489298867	Latrophilin-1 OS=Mus musculus GN=Lphn1 PE=1 SV=2
PCBP2	Q61990-3	15	15	58891.93	56260.64	49383.79	78229.86	62570.57	67515.5	74149.14	37907.64	63325.21	28149.86	0.524252703	0.280459322	61067.358	54209.47	Down	-0.1718565282799	Isoform 3 of Poly(rC)-binding protein 2 OS=Mus musculus GN=Pcbp2
BLOC1S1	O55102	1	1	7917	10951	17440	2193	7081	5063	5058	27705	7846	16900	0.524261338	0.280452169	9116.4	12514.4	Up	0.457052990815688	Biogenesis of lysosome-related organelles complex 1 subunit 1 OS=Mus musculus GN=Bloc1s1 PE=1 SV=2
GMFB	Q9CQI3	16	16	103164.67	96164.4	127847.13	78234.33	94617.93	83147	100128.6	124076.27	112943.67	115579	0.524592463	0.280177953	100005.69	107174.91	Up	0.0998850628670071	Glia maturation factor beta OS=Mus musculus GN=Gmfb PE=1 SV=3
GOPC	Q8BH60	4	4	12810.75	18095.25	10622	19679.75	14744.5	20585.75	15331.25	740.5	11515.25	NA	0.524996495	0.279843596	15190.45	12043.188	Down	-0.334947324415254	Golgi-associated PDZ and coiled-coil motif-containing protein OS=Mus musculus GN=Gopc PE=1 SV=1
FDXR	Q61578	1	1	21660	19849	7545	23698	23891	19799	14215	NA	16989	NA	0.52506139	0.279789916	19328.6	17001	Down	-0.185117536448304	NADPH:adrenodoxin oxidoreductase, mitochondrial OS=Mus musculus GN=Fdxr PE=2 SV=1
OTUD7A	Q8R554	2	2	5362.5	6347.5	6358	8524	6973	8102.5	6932.5	5124.5	9700	NA	0.525119115	0.279742173	6713	7464.875	Up	0.153160460090846	OTU domain-containing protein 7A OS=Mus musculus GN=Otud7a PE=2 SV=1
HNRNPA2B1	O88569-2	34	34	115025.09	69241.03	78853.78	43073.47	81595.12	67920.66	66828.28	85219.12	69274.84	56096.44	0.525122323	0.279739519	77557.698	69067.868	Down	-0.167255292309547	Isoform 2 of Heterogeneous nuclear ribonucleoproteins A2/B1 OS=Mus musculus GN=Hnrnpa2b1
GATB	Q99JT1	3	3	7423.67	8782	2426.67	12583	8864	13739.33	10364	5084	10150.67	7970.33	0.525496428	0.279430232	8015.868	9461.666	Up	0.239235482743812	Glutamyl-tRNA(Gln) amidotransferase subunit B, mitochondrial OS=Mus musculus GN=Pet112 PE=1 SV=1
YWHAE	P62259	22	22	202437.83	270062.28	180771.5	634099	287507.5	417252	345301.94	152253.61	254496.89	58163.78	0.526036577	0.278984057	314975.62	245493.64	Down	-0.359554500783308	14-3-3 protein epsilon OS=Mus musculus GN=Ywhae PE=1 SV=1
BCAN	Q61361	14	14	42303.92	39575.31	50073.38	26758.77	38034.77	36175	35652.08	48739.31	41106.85	51533.62	0.52646013	0.278634513	39349.23	42641.372	Up	0.115918453196291	Brevican core protein OS=Mus musculus GN=Bcan PE=1 SV=2
EPB41	P48193	2	2	63355	82770	73667.5	331183	76441	145845.5	97389.5	36473.5	69483	NA	0.526479524	0.278618515	125483.3	87297.875	Down	-0.52347693385653	Protein 4.1 OS=Mus musculus GN=Epb41 PE=1 SV=2
GLYR1	Q922P9	2	2	9896	8502	10929	3600.5	8440	7446.5	6521.5	11326.5	3529	6675	0.526491673	0.278608493	8273.5	7099.7	Down	-0.220739708113249	Putative oxidoreductase GLYR1 OS=Mus musculus GN=Glyr1 PE=1 SV=1
S100A13	P97352	3	3	73640	74213.33	57273.67	31308.33	66745	62193	69897.33	45658.33	61591.33	27545.33	0.526822154	0.27833597	60636.066	53377.064	Down	-0.18395620374033	Protein S100-A13 OS=Mus musculus GN=S100a13 PE=1 SV=1
SRP19	Q9D7A6	1	1	3779	6980	NA	9411	5138	5753	7367	NA	8997	NA	0.526872973	0.278294079	6327	7372.3333	Up	0.220599706732131	Signal recognition particle 19 kDa protein OS=Mus musculus GN=Srp19 PE=2 SV=1
USP8	Q80U87	1	1	21372	23034	24244	31646	24137	31417	24251	19642	34779	NA	0.527026441	0.278167595	24886.6	27522.25	Up	0.1452292749118	Ubiquitin carboxyl-terminal hydrolase 8 OS=Mus musculus GN=Usp8 PE=1 SV=2
SH3GL3	Q62421	2	2	68226	76634	75692	103266.5	68220.5	98865.5	79306	69213.5	91636	80417	0.527212272	0.278014489	78407.8	83887.6	Up	0.0974603911658303	Endophilin-A3 OS=Mus musculus GN=Sh3gl3 PE=2 SV=1
STXBP6	Q8R3T5	1	1	40730	50370	73265	50648	69166	53018	45165	32879	81933	35410	0.527236814	0.277994273	56835.8	49681	Down	-0.194105734538741	Syntaxin-binding protein 6 OS=Mus musculus GN=Stxbp6 PE=2 SV=1
EPHB6	O08644	3	3	322.33	890.33	NA	4228.33	1608	3845.67	3495.67	NA	662.67	NA	0.528915255	0.276613907	1762.2475	2668.0033	Up	0.598343910254341	Ephrin type-B receptor 6 OS=Mus musculus GN=Ephb6 PE=2 SV=4
SMC1A	Q9CU62	1	1	2236	1222	NA	4027	3868	4167	2846	NA	NA	NA	0.528956294	0.276580211	2838.25	3506.5	Up	0.305030056908725	Structural maintenance of chromosomes protein 1A OS=Mus musculus GN=Smc1a PE=1 SV=4
TPM2	P58774	14	14	26907.25	27827.58	16592.92	27402.75	26851.67	35369.17	33052.25	22726.42	42409.17	11547.58	0.529249967	0.27633916	25116.434	29020.918	Up	0.208461509244112	Tropomyosin beta chain OS=Mus musculus GN=Tpm2 PE=1 SV=1
SCRN1	Q9CZC8	13	13	114438.77	115429.77	135866.31	81792.08	106386.23	112319.69	129696.23	93762.23	134703.54	120653	0.530142214	0.275607612	110782.63	118226.94	Up	0.0938270710285195	Secernin-1 OS=Mus musculus GN=Scrn1 PE=1 SV=1
HNRNPLL	Q921F4-4	1	1	3623	3070	NA	2135	703	2097	2529	NA	896	NA	0.530192625	0.275566317	2382.75	1840.6667	Down	-0.372399203567736	Isoform 2 of Heterogeneous nuclear ribonucleoprotein L-like OS=Mus musculus GN=Hnrnpll
GUK1	Q64520	6	6	82776	90420.33	79892.83	49013.83	84787.5	70217.67	86744.83	89993.83	98563.67	71411.67	0.530345591	0.275441037	77378.098	83386.334	Up	0.107885696066614	Guanylate kinase OS=Mus musculus GN=Guk1 PE=1 SV=2
PREB	Q9WUQ2	1	1	NA	13609	14491	33165	134	23192	23551	29982	7063	NA	0.530529147	0.275290751	15349.75	20947	Up	0.448528479213969	Prolactin regulatory element-binding protein OS=Mus musculus GN=Preb PE=1 SV=1
PGRMC1	O55022	11	11	47431.45	61173.91	62656.45	59967.55	74474.45	75044.91	101098.55	35623.09	80081.09	54162.18	0.531595077	0.27441905	61140.762	69201.964	Up	0.178678450425894	Membrane-associated progesterone receptor component 1 OS=Mus musculus GN=Pgrmc1 PE=1 SV=4
CDH2	P15116	9	9	19786	30569.89	17697.11	48086.56	31682.56	40798.56	42928.44	39954.11	33744.89	14795.33	0.531648619	0.27437531	29564.424	34444.266	Up	0.220401665306013	Cadherin-2 OS=Mus musculus GN=Cdh2 PE=1 SV=2
COPS7A	Q9CZ04	2	2	NA	614	NA	3459	591	1040	633	NA	NA	NA	0.532061543	0.27403813	1554.6667	836.5	Down	-0.894167842440988	COP9 signalosome complex subunit 7a OS=Mus musculus GN=Cops7a PE=1 SV=2
UQCRH	P99028	4	4	37205.33	32299.33	17093	25135	30109	34771	32619.67	21119.67	25197	9677.67	0.532675323	0.273537422	28368.332	24677.002	Down	-0.201114192945828	Cytochrome b-c1 complex subunit 6, mitochondrial OS=Mus musculus GN=Uqcrh PE=1 SV=2
ALDH5A1	Q8BWF0	44	44	84352.84	96826.21	101226.98	77830.05	88046.86	87507.09	89950.12	114627.72	85196.44	93131.81	0.532821832	0.273417989	89656.588	94082.636	Up	0.069518885292351	Succinate-semialdehyde dehydrogenase, mitochondrial OS=Mus musculus GN=Aldh5a1 PE=1 SV=1
AKAP1	O08715-3	1	1	1363	914	NA	4497	2399	4759	2155	NA	NA	NA	0.532854081	0.273391704	2293.25	3457	Up	0.59212696385824	Isoform 2 of A-kinase anchor protein 1, mitochondrial OS=Mus musculus GN=Akap1
HNRNPA2B1	O88569-3	32	32	122433.9	73274.5	83861.43	45427.63	86561.23	71676.2	70966.13	90900.4	73625.6	59836.2	0.532992018	0.273279295	82311.738	73400.906	Down	-0.165300309086389	Isoform 3 of Heterogeneous nuclear ribonucleoproteins A2/B1 OS=Mus musculus GN=Hnrnpa2b1
DMTN	Q9WV69-2	18	18	60663.76	53987.35	43727.71	61576.47	65276.24	59624.94	58966.94	52706.71	62201.94	27163.88	0.533356048	0.272982776	57046.306	52132.882	Down	-0.129939851740015	Isoform 2 of Dematin OS=Mus musculus GN=Dmtn
PTPRS	B0V2N1-5	1	1	4633	10053	2347	26719	13891	18535	16983	NA	9297	NA	0.533720763	0.272685902	11528.6	14938.333	Up	0.373801869033994	Isoform 5 of Receptor-type tyrosine-protein phosphatase S OS=Mus musculus GN=Ptprs
CDC42BPG	Q80UW5	1	1	13553	21795	18647	35050	22920	39293	35187	8870	26709	NA	0.533798616	0.272622556	22393	27514.75	Up	0.297157400724392	Serine/threonine-protein kinase MRCK gamma OS=Mus musculus GN=Cdc42bpg PE=2 SV=2
LAMTOR5	Q9D1L9	1	1	119308	79030	22848	128270	76563	105674	101892	64403	129185	NA	0.534096896	0.272379946	85203.8	100288.5	Up	0.235166503095569	Ragulator complex protein LAMTOR5 OS=Mus musculus GN=Lamtor5 PE=2 SV=1
NUP62	Q63850	1	1	32579	38379	34835	26161	42502	41095	35497	38828	32799	NA	0.534754151	0.271845836	34891.2	37054.75	Up	0.0867952751214854	Nuclear pore glycoprotein p62 OS=Mus musculus GN=Nup62 PE=1 SV=2
MRPS9	Q9D7N3	3	3	12056.67	14651	6687.33	12938	12207	14035.67	12962.67	14580.33	13386.33	8988.67	0.534880434	0.271743289	11708	12790.734	Up	0.127604405211359	28S ribosomal protein S9, mitochondrial OS=Mus musculus GN=Mrps9 PE=2 SV=3
MICAL3	Q8CJ19	11	11	21302.09	24767.91	15581.45	25720.18	33120	29597.27	39450	18821.09	34389.36	15726.55	0.535033303	0.271619185	24098.326	27596.854	Up	0.195570878765328	Protein-methionine sulfoxide oxidase MICAL3 OS=Mus musculus GN=Mical3 PE=2 SV=2
CHID1	Q922Q9	2	2	43854	69665	16109.5	117025	85569.5	107580	119403	44785	59378.5	NA	0.535855634	0.270952199	66444.6	82786.625	Up	0.31724574945304	Chitinase domain-containing protein 1 OS=Mus musculus GN=Chid1 PE=2 SV=1
KRT1	P04104	1	1	31304	15458	49390	52821	37921	33040	35067	NA	32083	94059	0.535913401	0.270905383	37378.8	48562.25	Up	0.377615012607629	Keratin, type II cytoskeletal 1 OS=Mus musculus GN=Krt1 PE=1 SV=4
ARHGDIB	Q61599	2	2	26024	34413	6909.5	31427.5	30793.5	28672	39813	14142	42262.5	NA	0.536214279	0.270661625	25913.5	31222.375	Up	0.268876397423638	Rho GDP-dissociation inhibitor 2 OS=Mus musculus GN=Arhgdib PE=1 SV=3
KCND3	Q9Z0V1	1	1	4511	18918	9729	9390	14382	8583	12023	NA	25547	NA	0.536547356	0.270391941	11386	15384.333	Up	0.434200922253034	Potassium voltage-gated channel subfamily D member 3 OS=Mus musculus GN=Kcnd3 PE=1 SV=1
RAB3A	P63011	8	8	39317.38	68320.62	47684.5	130772.25	57120	84614.25	92281	37022.62	44343.25	13920.5	0.536704767	0.270264548	68642.95	54436.324	Down	-0.334541908257367	Ras-related protein Rab-3A OS=Mus musculus GN=Rab3a PE=1 SV=1
PLCH2	A2AP18-3	3	3	16524.5	18294.5	17059.5	37229.5	22121	29609.5	28143.5	24567	18583	NA	0.53803192	0.269191958	22245.8	25225.75	Up	0.181364182070702	Isoform 3 of 1-phosphatidylinositol 4,5-bisphosphate phosphodiesterase eta-2 OS=Mus musculus GN=Plch2
KHDRBS3	Q9R226	2	2	160083	92877.5	93197	57597.5	119526	79798	97060.5	NA	101053.5	NA	0.538074897	0.269157269	104656.2	92637.333	Down	-0.175992152190288	KH domain-containing, RNA-binding, signal transduction-associated protein 3 OS=Mus musculus GN=Khdrbs3 PE=1 SV=1
MTX1	P47802	1	1	1168	5195	NA	6907	3885	6321	7899	NA	2623	NA	0.538238264	0.269025431	4288.75	5614.3333	Up	0.388557501077976	Metaxin-1 OS=Mus musculus GN=Mtx1 PE=1 SV=1
FBXO41	Q6NS60	10	10	6831.9	14683.6	3907.9	29211.8	21193.6	27242	29669.7	8261.6	13583.6	NA	0.538257247	0.269010114	15165.76	19689.225	Up	0.376588528142279	F-box only protein 41 OS=Mus musculus GN=Fbxo41 PE=1 SV=3
RPS26	P62855	5	5	52801	62477.4	62034.8	34096.2	68876.8	49734.8	57568.4	68219.4	69621	58152	0.53846917	0.268839158	56057.24	60659.12	Up	0.113823854037465	40S ribosomal protein S26 OS=Mus musculus GN=Rps26 PE=2 SV=3
CNBP	P53996	2	2	32098	20346.5	16704	22668	20654.5	19651.5	32625	11677.5	49546.5	NA	0.538649428	0.268693797	22494.2	28375.125	Up	0.335073689094991	Cellular nucleic acid-binding protein OS=Mus musculus GN=Cnbp PE=1 SV=2
ATP5C1	Q91VR2	16	16	130283.31	140659.44	158641.12	118479.88	113177.5	110188.31	116463.88	157470.88	122428.75	236736	0.538761885	0.268603136	132248.25	148657.56	Up	0.168744240648402	ATP synthase subunit gamma, mitochondrial OS=Mus musculus GN=Atp5c1 PE=1 SV=1
CPNE4	Q8BLR2	2	2	12003	25019.5	14828.5	45599	21033.5	30515	35511.5	NA	19783	NA	0.539119039	0.268315331	23696.7	28603.167	Up	0.271488713573527	Copine-4 OS=Mus musculus GN=Cpne4 PE=1 SV=1
KRT87	Q6IMF0	1	1	NA	4118	NA	77246	NA	14412	2691	NA	NA	NA	0.539241848	0.268216412	40682	8551.5	Down	-2.25014119910685	Keratin, type II cuticular Hb3 OS=Mus musculus GN=Krt83 PE=2 SV=2
ZC2HC1A	Q8BJH1-2	7	7	78183.71	80572.29	98167.71	41198.57	63236.14	54551.86	75051.43	75716.43	98319	98154	0.539408357	0.26808233	72271.684	80358.544	Up	0.153020913535799	Isoform 2 of Zinc finger C2HC domain-containing protein 1A OS=Mus musculus GN=Zc2hc1a
STAU2	Q8CJ67-4	1	1	23249	10551	NA	34363	23272	35811	29519	NA	17599	NA	0.539632	0.267902305	22858.75	27643	Up	0.27416768109711	Isoform 4 of Double-stranded RNA-binding protein Staufen homolog 2 OS=Mus musculus GN=Stau2
ARHGEF10L	A2AWP8-3	1	1	14636	27672	10561	55834	33455	51241	45006	10078	38773	NA	0.539866235	0.267713834	28431.6	36274.5	Up	0.351460439069585	Isoform 3 of Rho guanine nucleotide exchange factor 10-like protein OS=Mus musculus GN=Arhgef10l
RPL35A	O55142	7	7	120066.71	128994.14	145980.57	88242.71	109511.43	105929.14	112496	117813.43	118906.57	204770.14	0.540962612	0.26683275	118559.11	131983.06	Up	0.154746181010008	60S ribosomal protein L35a OS=Mus musculus GN=Rpl35a PE=1 SV=2
CHMP5	Q9D7S9	3	3	28878.5	34048.5	31757.5	34197.5	38870.5	33914.5	31022	33849.5	44115	NA	0.541192407	0.266648305	33550.5	35725.25	Up	0.0906098401858055	Charged multivesicular body protein 5 OS=Mus musculus GN=Chmp5 PE=2 SV=1
OXSM	Q9D404	2	2	10178.5	11305.5	5242.5	7334	10041.5	10456	10747	5206	14801.5	NA	0.541371224	0.266504833	8820.4	10302.625	Up	0.22409597994023	3-oxoacyl-[acyl-carrier-protein] synthase, mitochondrial OS=Mus musculus GN=Oxsm PE=1 SV=1
NRCAM	Q810U4-5	8	8	11400.88	13564.75	9147.38	20819	13143.62	18367	16654.5	3136.62	14014.75	3733.62	0.542117241	0.265906781	13615.126	11181.298	Down	-0.284122658128367	Isoform 5 of Neuronal cell adhesion molecule OS=Mus musculus GN=Nrcam
PTMA	P26350	2	2	16477	16353.5	28034	9524.5	14994	10777	12425	22613	20372.5	37058	0.542200679	0.265839943	17076.6	20649.1	Up	0.274058143691541	Prothymosin alpha OS=Mus musculus GN=Ptma PE=1 SV=2
ENO3	P21550	16	16	74587.69	71670.19	87069.69	70125.12	76998	81517	74072.31	76955.44	81787.06	76906.25	0.542544342	0.265564762	76090.138	78247.612	Up	0.040337244871419	Beta-enolase OS=Mus musculus GN=Eno3 PE=1 SV=3
DMTN	Q9WV69	20	20	52827.5	47254.45	38244.6	53357.9	56847.7	52103.8	51501.3	45748.9	54766.45	23089.3	0.542791403	0.265367039	49706.43	45441.95	Down	-0.129407745256582	Dematin OS=Mus musculus GN=Dmtn PE=1 SV=1
UBXN6	Q99PL6-2	7	7	50819.33	57265.17	35278.17	52907.17	66536.33	60309.5	66168.5	1821.17	66424	21819	0.543342408	0.264926397	52561.234	43308.434	Down	-0.279351140161442	Isoform 2 of UBX domain-containing protein 6 OS=Mus musculus GN=Ubxn6
ERLEC1	Q8VEH8	2	2	46505.5	43250.5	67341.5	36262.5	41423	46597	47083	47947.5	63371	NA	0.543457491	0.264834421	46956.6	51249.625	Up	0.126213497678099	Endoplasmic reticulum lectin 1 OS=Mus musculus GN=Erlec1 PE=2 SV=1
YWHAQ	P68254	24	24	154350.88	208592.21	125478.71	463858.17	218249.25	314933.79	266918.71	114071.79	196861.79	25840.62	0.543569266	0.264745107	234105.84	183725.34	Down	-0.349610327600417	14-3-3 protein theta OS=Mus musculus GN=Ywhaq PE=1 SV=1
CACNA1A	P97445	4	4	5448.25	16470.25	8049.75	25169	21066.25	23951	25910.75	17008.5	8143.75	NA	0.544151823	0.264279911	15240.7	18753.5	Up	0.299230706721305	Voltage-dependent P/Q-type calcium channel subunit alpha-1A OS=Mus musculus GN=Cacna1a PE=1 SV=2
EIF4A2	P10630	5	5	45245.8	54441.8	64730.2	26078.6	43109	36265	48818.6	55012.2	69393	50560.4	0.545038142	0.263573104	46721.08	52009.84	Up	0.154710975915675	Eukaryotic initiation factor 4A-II OS=Mus musculus GN=Eif4a2 PE=2 SV=2
PLCD1	Q8R3B1	1	1	10761	12762	6512	14547	15218	15685	17889	NA	8717	NA	0.545069465	0.263548146	11960	14097	Up	0.237170784036805	1-phosphatidylinositol 4,5-bisphosphate phosphodiesterase delta-1 OS=Mus musculus GN=Plcd1 PE=2 SV=2
GPC1	Q9QZF2	1	1	76960	205472	13778	225424	145820	191660	258733	106945	112746	NA	0.545071153	0.263546802	133490.8	167521	Up	0.327601642625883	Glypican-1 OS=Mus musculus GN=Gpc1 PE=1 SV=1
EML4	Q3UMY5-3	3	3	16013.67	17083.33	13303	10568	20895.33	18395.33	21940.33	9934	20342.67	15889.67	0.545295327	0.263368224	15572.666	17300.4	Up	0.151789443379679	Isoform 3 of Echinoderm microtubule-associated protein-like 4 OS=Mus musculus GN=Eml4
PPM1E	Q80TL0	5	5	30200.6	41331.2	44639.6	30004.6	38753	34802.8	48587.2	33177.6	36232.8	9696.2	0.545340543	0.263332214	36985.8	32499.32	Down	-0.186561949299503	Protein phosphatase 1E OS=Mus musculus GN=Ppm1e PE=1 SV=2
SRSF2	Q62093	5	5	16681.25	17895	14060	14808	11542.5	12699.75	11519.75	8387	15198.25	20031.5	0.545607659	0.263119542	14997.35	13567.25	Down	-0.144579277301718	Serine/arginine-rich splicing factor 2 OS=Mus musculus GN=Srsf2 PE=1 SV=4
GOT2	P05202	32	32	105904.44	107662.03	139751.78	83210.22	90955.28	104433.06	92557.25	125721.47	103514.59	143214.72	0.545794253	0.262971042	105496.75	113888.22	Up	0.110419949585584	Aspartate aminotransferase, mitochondrial OS=Mus musculus GN=Got2 PE=1 SV=1
SMARCC2	Q6PDG5	6	6	21517.6	12167.8	9453.8	20941	22105.2	16629	20543.4	7009.8	14857.2	NA	0.546048395	0.262768865	17237.08	14759.85	Down	-0.223837339561773	SWI/SNF complex subunit SMARCC2 OS=Mus musculus GN=Smarcc2 PE=1 SV=2

PLXNA3	P70208	2	2	12483	19743.5	9604	19630.5	15359	20442.5	23645.5	12717.5	13234.5	NA	0.546276973	0.262587106	15364	17510	Up	0.188625214789855	Plexin-A3 OS=Mus musculus GN=Plxna3 PE=1 SV=2
STRN3	Q9ERG2	6	6	12458.17	13911	8431.17	20794.33	14151.83	18364.33	12039.33	4336	12087.5	NA	0.546485898	0.26242104	13949.3	11706.79	Down	-0.25284718363058	Striatin-3 OS=Mus musculus GN=Strn3 PE=1 SV=1
COX7C	P17665	1	1	544533	611257	960275	396369	449832	493670	374470	893059	384342	1665822	0.547196325	0.261856828	592453.2	762272.6	Up	0.363605824296447	Cytochrome c oxidase subunit 7C, mitochondrial OS=Mus musculus GN=Cox7c PE=1 SV=1
PSMD12	Q9D8W5	2	2	16715.5	19961	36018.5	13560	17162	17712.5	23951.5	19104	9761.5	69235	0.547430935	0.261670664	20683.4	27952.9	Up	0.43452460486018	26S proteasome non-ATPase regulatory subunit 12 OS=Mus musculus GN=Psmd12 PE=1 SV=4
RPS27A	P62983	8	8	74122.88	91507.75	58899.25	67975.25	86911.5	77661.12	110888.62	64799.12	146265.75	41948.75	0.547587697	0.261546318	75883.326	88312.672	Up	0.218837551176897	Ubiquitin-40S ribosomal protein S27a OS=Mus musculus GN=Rps27a PE=1 SV=2
GRCC10	O35127	5	5	34578.6	35917.2	34370.2	37779	39457.8	38205.8	35190.8	44943.4	43657.8	29666.6	0.547802842	0.261375719	36420.56	38332.88	Up	0.0738292885339643	Protein C10 OS=Mus musculus GN=Grcc10 PE=2 SV=1
AP1B1	O35643	6	6	30942.33	42277.67	40710.83	78284.67	47790.33	55165.17	57431.83	65178.67	31327	62469.33	0.548504964	0.260819437	48001.166	54314.4	Up	0.178265289592855	AP-1 complex subunit beta-1 OS=Mus musculus GN=Ap1b1 PE=2 SV=2
ALDH2	P47738	5	5	20345.6	24130.8	10057.6	50294.2	25133.6	28786.4	27657	3974.2	21481.4	NA	0.549088889	0.260357344	25992.36	20474.75	Down	-0.344241794132753	Aldehyde dehydrogenase, mitochondrial OS=Mus musculus GN=Aldh2 PE=1 SV=1
CACNA2D1	O08532-5	27	27	24886.77	35324.92	19456.73	72819.69	31511.04	52223.73	54232.46	44186.73	35871.15	30703	0.549289521	0.260198686	36799.83	43443.414	Up	0.239438380121099	Isoform 2E of Voltage-dependent calcium channel subunit alpha-2/delta-1 OS=Mus musculus GN=Cacna2d1
PTDSS2	Q9Z1X2	2	2	NA	NA	NA	3028	2662	4085	3123	NA	2402	NA	0.54961787	0.259939156	2845	3203.3333	Up	0.171145277746012	Phosphatidylserine synthase 2 OS=Mus musculus GN=Ptdss2 PE=2 SV=2
SH3PXD2B	A2AAY5	1	1	1319	NA	NA	2666	1753	2380	2036	NA	NA	NA	0.549947333	0.2596789	1912.6667	2208	Up	0.207154704730661	SH3 and PX domain-containing protein 2B OS=Mus musculus GN=Sh3pxd2b PE=1 SV=1
RIMS1	Q99NE5-2	4	4	67819.25	71024.75	54266	89053.75	93252.75	80719.75	90463.75	57314	101949.25	NA	0.55009845	0.259559579	75083.3	82611.688	Up	0.137853841847207	Isoform 2 of Regulating synaptic membrane exocytosis protein 1 OS=Mus musculus GN=Rims1
DHRS4	Q99LB2	3	3	43409.67	62595.33	55668.67	53556.67	37766.33	75065.33	56353.67	30180.33	58739.33	59380	0.550381142	0.259336455	50599.334	55943.732	Up	0.144858103083857	Dehydrogenase/reductase SDR family member 4 OS=Mus musculus GN=Dhrs4 PE=1 SV=3
PTGES2	Q8BWM0	1	1	3008	8663	NA	4897	3988	7338	2276	NA	12060	NA	0.550929118	0.258904273	5139	7224.6667	Up	0.491443373609945	Prostaglandin E synthase 2 OS=Mus musculus GN=Ptges2 PE=1 SV=3
PABPN1	Q8CCS6	1	1	34523	31455	18650	23554	28421	36619	17330	NA	11383	NA	0.55150935	0.258447121	27320.6	21777.333	Down	-0.327161862922249	Polyadenylate-binding protein 2 OS=Mus musculus GN=Pabpn1 PE=2 SV=3
RPL22	P67984	15	15	30500.92	37322	42787.08	25800.62	33952.23	29806.38	36303.54	33619.62	43450.46	38681	0.552418022	0.257732161	34072.57	36372.2	Up	0.0942254208852513	60S ribosomal protein L22 OS=Mus musculus GN=Rpl22 PE=1 SV=2
HSPA1L	P16627	23	23	59620.48	59967.65	66311.43	82872.48	54991.3	64788.09	60723.13	61295.35	58112.91	62792.7	0.552639564	0.257558027	64752.668	61542.436	Down	-0.0733580870977767	Heat shock 70 kDa protein 1-like OS=Mus musculus GN=Hspa1l PE=2 SV=4
FAM175B	Q3TCJ1	1	1	17523	22921	31804	25834	21085	21922	20061	NA	24383	NA	0.552698939	0.257511369	23833.4	22122	Down	-0.107502954409306	BRISC complex subunit Abro1 OS=Mus musculus GN=Fam175b PE=2 SV=1
NKIRAS2	Q9CR56	1	1	33801	33446	38758	17049	31255	24693	27362	46964	33170	38947	0.553630215	0.256780215	30861.8	34227.2	Up	0.149321066782823	NF-kappa-B inhibitor-interacting Ras-like protein 2 OS=Mus musculus GN=Nkiras2 PE=2 SV=1
TACC1	Q6Y685-2	6	6	14414.33	17653.5	6696.5	19548.17	17792.83	17120	17228.5	10561	16583.67	3856.17	0.553639397	0.256773013	15221.066	13069.868	Down	-0.219824830217025	Isoform 2 of Transforming acidic coiled-coil-containing protein 1 OS=Mus musculus GN=Tacc1
DIRAS2	Q5PR73	3	3	14113.67	18959	15691.33	27068.67	14274.67	21805	13865.33	25768.33	19307	NA	0.553705498	0.256721164	18021.468	20186.415	Up	0.163668182875508	GTP-binding protein Di-Ras2 OS=Mus musculus GN=Diras2 PE=2 SV=1
PNKP	Q9JLV6	1	1	5065	3592	NA	3486	3636	4662	5276	5826	2250	NA	0.554501016	0.256097654	3944.75	4503.5	Up	0.191112789640155	Bifunctional polynucleotide phosphatase/kinase OS=Mus musculus GN=Pnkp PE=1 SV=2
NEBL	Q9DC07	12	12	51257	37222.9	33527.6	69932.5	57216.8	52777.9	53632.5	37397.3	57513.3	16904.9	0.555059221	0.255660678	49831.36	43645.18	Down	-0.191231611512483	LIM zinc-binding domain-containing Nebulette OS=Mus musculus GN=Nebl PE=2 SV=1
PACS1	Q8K212	6	6	46412.83	49955.17	40419.33	92970.83	60244	77668.33	70290.17	25662.17	48178.83	23484	0.555132674	0.25560321	58000.432	49056.7	Down	-0.241613457426551	Phosphofurin acidic cluster sorting protein 1 OS=Mus musculus GN=Pacs1 PE=2 SV=2
ARMC6	Q8BNU0-2	1	1	26816	21351	12757	56586	34629	47739	43287	NA	21242	NA	0.555427731	0.255372441	30427.8	37422.667	Up	0.298522338442993	Isoform 2 of Armadillo repeat-containing protein 6 OS=Mus musculus GN=Armc6
CYTH1	Q9QX11	1	1	74461	136958	73733	24913	88407	94152	102728	59556	115923	NA	0.555613756	0.25522701	79694.4	93089.75	Up	0.224143971251082	Cytohesin-1 OS=Mus musculus GN=Cyth1 PE=1 SV=2
MINK1	Q9JM52	11	11	37832.27	48310	45985.18	38881.36	52372.91	51582.73	59965.55	14941.18	54048.36	6635.27	0.555663807	0.25518789	44676.344	37434.618	Down	-0.255138097962556	Misshapen-like kinase 1 OS=Mus musculus GN=Mink1 PE=1 SV=3
RPL3	P27659	20	20	168384.55	181912.2	176341.75	148910.7	174185.85	166030.1	194801.5	167513.45	251006.8	134814.3	0.555757058	0.255115013	169947.01	182833.23	Up	0.105443324796738	60S ribosomal protein L3 OS=Mus musculus GN=Rpl3 PE=1 SV=3
RIMS2	Q9EQZ7	4	4	55616.75	50213.25	48498.75	49579	74347.75	52240	71388.5	43069.75	81063.75	NA	0.55578339	0.255094437	55651.1	61940.5	Up	0.154472824704722	Regulating synaptic membrane exocytosis protein 2 OS=Mus musculus GN=Rims2 PE=1 SV=1
PKM	P52480	79	79	133625.19	138601.78	131879.19	162049.68	152974.9	162769.21	165338.94	141671.03	131762.37	144353.53	0.556233801	0.254742623	143826.15	149179.02	Up	0.0527186300856244	Pyruvate kinase PKM OS=Mus musculus GN=Pkm PE=1 SV=4
SPECC1	Q5SXY1	10	10	27986.22	26723.67	27656.33	27597.67	30523.89	32782.67	29661.67	18083.78	36210.67	34163.33	0.55632443	0.254671869	28097.556	30180.424	Up	0.103168427741661	Cytospin-B OS=Mus musculus GN=Specc1 PE=1 SV=2
CHCHD6	Q91VN4	11	11	91827.27	104779.09	94181.55	62645.82	92790.91	97886.55	98539	37754.55	131190.82	5100	0.556601704	0.254455468	89244.928	74094.184	Down	-0.268409876630132	Coiled-coil-helix-coiled-coil-helix domain-containing protein 6, mitochondrial OS=Mus musculus GN=Chchd6 PE=2 SV=2
SLC6A7	Q6PGE7	3	3	847	6599.67	1742.67	9467.67	7866.67	10012.67	12103.33	1847	5787.33	4509	0.556980491	0.254160016	5304.736	6851.866	Up	0.369215982780976	Sodium-dependent proline transporter OS=Mus musculus GN=Slc6a7 PE=2 SV=1
NDUFB11	O09111	3	3	41820.33	90609.33	32054.67	189389.67	102052.33	143954	143402	77961	82222.33	NA	0.557507498	0.253749287	91185.266	111884.83	Up	0.295141839608961	NADH dehydrogenase [ubiquinone] 1 beta subcomplex subunit 11, mitochondrial OS=Mus musculus GN=Ndufb11 PE=1 SV=2
TUBA3A	P05214	66	66	212403.24	257995.03	259553.61	298720.45	227547.71	275769.12	278584.03	249207.7	263580.94	240673.82	0.558055323	0.253322745	251244.01	261563.12	Up	0.0580699590128852	Tubulin alpha-3 chain OS=Mus musculus GN=Tuba3a PE=1 SV=1
TRIO	Q0KL02-2	6	6	334.6	1919.6	509.6	6367.8	3285.4	5456.8	5045	729.4	2509.4	2984.4	0.558205095	0.253206204	2483.4	3345	Up	0.429689556464739	Isoform 2 of Triple functional domain protein OS=Mus musculus GN=Trio
PSMD11	Q8BG32	1	1	34428	44001	51897	57593	54436	60813	51192	40215	53483	52931	0.558901461	0.252664755	48471	51726.8	Up	0.0937900972644948	26S proteasome non-ATPase regulatory subunit 11 OS=Mus musculus GN=Psmd11 PE=1 SV=3
RPL13A	P19253	3	3	76968.33	91794	90784	95127.33	75799.33	83084	86140	81271.33	101706	48548.67	0.558983438	0.252601059	86094.598	80150	Down	-0.103220198274756	60S ribosomal protein L13a OS=Mus musculus GN=Rpl13a PE=1 SV=4
TPD52L1	O54818	1	1	5791	22536	NA	10267	13812	13078	19155	NA	NA	NA	0.559003386	0.252585561	13101.5	16116.5	Up	0.29880647322252	Tumor protein D53 OS=Mus musculus GN=Tpd52l1 PE=2 SV=1
AQP4	P55088-3	2	2	6165	7436	1164.5	30322.5	6511.5	24000.5	12373.5	NA	7706	NA	0.559636185	0.252094213	10319.9	14693.333	Up	0.509732732097588	Isoform 3 of Aquaporin-4 OS=Mus musculus GN=Aqp4
PSMC1	P62192	17	17	30293.47	34336.65	38204.41	28460.29	29038.82	28663.53	34029.06	31632.59	34982.06	21901.71	0.559650369	0.252083206	32066.728	30241.79	Down	-0.0845336180335018	26S protease regulatory subunit 4 OS=Mus musculus GN=Psmc1 PE=1 SV=1
UBXN6	Q99PL6	8	8	45064.86	49771.29	30238.43	46393.14	58502.71	53247.71	57978.86	1561	59123.43	18702	0.559983946	0.251824423	45994.086	38122.6	Down	-0.270801852975058	UBX domain-containing protein 6 OS=Mus musculus GN=Ubxn6 PE=1 SV=1
CCT7	P80313	16	16	46782.13	53183.93	56647.8	54328.73	55388.93	51908.47	55124.27	49774.27	52433.8	51126.73	0.560033924	0.251785665	53266.304	52073.508	Down	-0.0326735807531121	T-complex protein 1 subunit eta OS=Mus musculus GN=Cct7 PE=1 SV=1
STK24	Q99KH8	2	2	61794	61602	106590	63364	66054.5	74211	82679	96524	62994	74636	0.560125606	0.251714574	71880.9	78208.8	Up	0.12172247518353	Serine/threonine-protein kinase 24 OS=Mus musculus GN=Stk24 PE=1 SV=1
CMC2	Q8K199	1	1	29697	27767	33968	30974	35260	29061	26795	41145	38788	NA	0.560138207	0.251704803	31533.2	33947.25	Up	0.106423126673602	COX assembly mitochondrial protein 2 homolog OS=Mus musculus GN=Cmc2 PE=2 SV=1
ETFA	Q99LC5	28	28	80500.61	84553.82	71880.61	63527.39	85450.39	72101.64	84084.39	73775	78200.36	60346.04	0.560584613	0.251358827	77182.564	73701.486	Down	-0.0665812630844556	Electron transfer flavoprotein subunit alpha, mitochondrial OS=Mus musculus GN=Etfa PE=1 SV=2
OLA1	Q9CZ30	3	3	42570.33	46145.33	53452.67	87983.33	50011.67	62756	63543	60432	58310	NA	0.560747262	0.251232838	56032.666	61260.25	Up	0.128683117765423	Obg-like ATPase 1 OS=Mus musculus GN=Ola1 PE=1 SV=1
PUM1	Q80U78-4	5	5	14477.2	17835.8	6400.2	14700.4	16845.2	18024.4	22321.8	1020	18618.8	24479	0.56088435	0.251126677	14051.76	16892.8	Up	0.265657635164844	Isoform 4 of Pumilio homolog 1 OS=Mus musculus GN=Pum1
FAM98A	Q3TJZ6	2	2	27416.5	36128.5	51619.5	54327	41223	76522	54850	30451	71431	16487.5	0.561239089	0.250852089	42142.9	49948.3	Up	0.245145981999014	Protein FAM98A OS=Mus musculus GN=Fam98a PE=2 SV=1
DMXL1	Q6PNC0	1	1	NA	NA	NA	6153	1810	6623	NA	NA	4878	NA	0.561677764	0.250512769	3981.5	5750.5	Up	0.530375344725712	DmX-like protein 1 OS=Mus musculus GN=Dmxl1 PE=1 SV=1
PIN4	Q9CWW6	1	1	91878	130414	56630	118421	61003	99609	68609	146777	104992	NA	0.56214244	0.250153626	91669.2	104996.75	Up	0.195835684143818	Peptidyl-prolyl cis-trans isomerase NIMA-interacting 4 OS=Mus musculus GN=Pin4 PE=2 SV=1
BPHL	Q8R164	2	2	19998.5	23952	16197	24515.5	22632.5	26964.5	17542.5	24355	25701	19866.5	0.562193751	0.250113986	21459.1	22885.9	Up	0.0928694574181966	Valacyclovir hydrolase OS=Mus musculus GN=Bphl PE=1 SV=1
EIF2S1	Q6ZWX6	6	6	14471.67	19706.67	12260	27637.83	15012.33	21061.5	18219.67	7625.83	14640.83	NA	0.562345809	0.249996537	17817.7	15386.958	Down	-0.211603126095351	Eukaryotic translation initiation factor 2 subunit 1 OS=Mus musculus GN=Eif2s1 PE=1 SV=3
PGK2	P09041	10	10	71550.7	71250.8	69547.3	74868	71519.1	75453.6	73864	62614.6	79845.5	49544.9	0.562346618	0.249995912	71747.18	68264.52	Down	-0.0717861854403396	Phosphoglycerate kinase 2 OS=Mus musculus GN=Pgk2 PE=1 SV=4
GOT1	P05201	21	21	69481.86	76982.19	75553.62	80803.76	76496.95	74133	71149.48	108231.86	71962.86	76474.38	0.562708179	0.249716772	75863.676	80390.316	Up	0.0836124419500159	Aspartate aminotransferase, cytoplasmic OS=Mus musculus GN=Got1 PE=1 SV=3
SPOCK2	Q9ER58	1	1	108583	85126	177240	31901	83102	63722	58602	217619	96840	167136	0.562783995	0.249658262	97190.4	120783.8	Up	0.31354124417733	Testican-2 OS=Mus musculus GN=Spock2 PE=2 SV=1
RAB5B	P61021	4	4	24827.25	23589.75	40722.5	26322.5	20467.75	21788	25583	34912.75	26246.5	43137.25	0.563303571	0.249257496	27185.95	30333.5	Up	0.158050728160226	Ras-related protein Rab-5B OS=Mus musculus GN=Rab5b PE=1 SV=1
TIPRL	Q8BH58	4	4	23008	31593.75	14176	32694.75	24195.25	31607.5	34771.75	15584.75	32014.5	NA	0.563377978	0.249200133	25133.55	28494.625	Up	0.181075346648791	TIP41-like protein OS=Mus musculus GN=Tiprl PE=2 SV=1
DLST	Q9D2G2	23	23	98240.91	91393.52	72642.87	103942	91945.35	113637.52	88880.17	71689.43	102894.52	36320.13	0.56442199	0.248396074	91632.93	82684.354	Down	-0.148251790509519	Dihydrolipoyllysine-residue succinyltransferase component of 2-oxoglutarate dehydrogenase complex, mitochondrial OS=Mus musculus GN=Dlst PE=1 SV=1
MFF	Q6PCP5-4	8	8	16864.29	20777.71	7931.43	48174	32969.71	43129.86	30777.86	2025	17274.57	1260.57	0.564736069	0.248154474	25343.428	18893.572	Down	-0.423716197329447	Isoform 4 of Mitochondrial fission factor OS=Mus musculus GN=Mff
CORO1C	Q9WUM4	8	8	16892.5	24783.38	17042.75	45969.88	27353.88	26882.5	34443.88	12850.75	30863.75	3169.5	0.564864272	0.248055894	26408.478	21642.076	Down	-0.287162261798921	Coronin-1C OS=Mus musculus GN=Coro1c PE=1 SV=2
FABP7	P51880	18	18	82512.94	93975.76	92155.06	77016.35	83667.71	94478.35	108606.59	99753.47	120304.12	46520.59	0.565664575	0.247441019	85865.564	93932.624	Up	0.129546650360402	Fatty acid-binding protein, brain OS=Mus musculus GN=Fabp7 PE=1 SV=2
TACC1	Q6Y685	5	5	16814	20912.8	7393.6	23312.2	20703.2	19974.6	20167.6	12673.2	19373.2	4627.4	0.567104817	0.246336663	17827.16	15363.2	Down	-0.21459814292955	Transforming acidic coiled-coil-containing protein 1 OS=Mus musculus GN=Tacc1 PE=1 SV=1
HNRNPF	Q9Z2X1	6	6	22762.83	20384.67	11038.67	33240.33	20853.5	26643.17	20974	16800.83	19629.17	11186.17	0.567150356	0.246301791	21656	19046.668	Down	-0.185228156118687	Heterogeneous nuclear ribonucleoprotein F OS=Mus musculus GN=Hnrnpf PE=1 SV=3
SUB1	P11031	17	17	53856.82	41013.41	24461.06	45248.06	46980.35	44013.35	44510.47	39653.65	46656.47	12900.29	0.567184736	0.246275466	42311.94	37546.846	Down	-0.172373110744088	Activated RNA polymerase II transcriptional coactivator p15 OS=Mus musculus GN=Sub1 PE=1 SV=3
YWHAZ	P63101	67	67	89791.51	115267.63	75110.35	214258.31	122535.23	156691.97	147855.74	67234.15	112164.02	31793.8	0.56795166	0.245688627	123392.61	103147.94	Down	-0.258540994058633	14-3-3 protein zeta/delta OS=Mus musculus GN=Ywhaz PE=1 SV=1
YWHAH	P68510	23	23	175833.13	234617.22	174071.57	441356.35	248026.7	325860.35	306046.13	116819.61	226907.65	95926.17	0.568360899	0.245375808	254780.99	214311.98	Down	-0.249545148189767	14-3-3 protein eta OS=Mus musculus GN=Ywhah PE=1 SV=2
RGS17	Q9QZB0	4	4	11366	24513	7688.75	49640.5	29189.25	37660	46713.75	12346.75	26376.5	NA	0.56850105	0.245268729	24479.5	30774.25	Up	0.330149606262856	Regulator of G-protein signaling 17 OS=Mus musculus GN=Rgs17 PE=1 SV=1
NCOA5	Q91W39	1	1	21061	17981	8420	23260	18499	16784	14905	29876	9763	33660	0.568521852	0.245252838	17844.2	20997.6	Up	0.234769215816606	Nuclear receptor coactivator 5 OS=Mus musculus GN=Ncoa5 PE=1 SV=1
MAP1A	Q9QYR6	111	111	42676.14	47462.2	39780.21	65870.5	50394.76	54600.49	53724.52	34970.08	53636.04	27547.09	0.568538793	0.245239897	49236.762	44895.644	Down	-0.133160413467541	Microtubule-associated protein 1A OS=Mus musculus GN=Map1a PE=1 SV=2
ROBO1	O89026	2	2	131528	129818	187005	103169	149740	111821	140187	NA	138695	NA	0.569164563	0.244762148	140252	130234.33	Down	-0.106911511349813	Roundabout homolog 1 OS=Mus musculus GN=Robo1 PE=1 SV=1
TTYH3	Q6P5F7	3	3	12254.33	23501.33	4976.67	29790.67	26555	38773	38664	23499.67	18729.33	2142.33	0.5692649	0.244685593	19415.6	24361.666	Up	0.327396505426249	Protein tweety homolog 3 OS=Mus musculus GN=Ttyh3 PE=1 SV=1
MFF	Q6PCP5	12	12	24620	27566.9	23699.8	46178.6	33704.8	42970.8	32949	18465	28661.1	12617.3	0.56983326	0.244252206	31154.02	27132.64	Down	-0.199388906950969	Mitochondrial fission factor OS=Mus musculus GN=Mff PE=1 SV=1
TDRD6	P61407	1	1	322382	363576	168700	261015	263161	353815	325245	155966	430183	NA	0.569885732	0.244212216	275766.8	316302.25	Up	0.197855039454826	Tudor domain-containing protein 6 OS=Mus musculus GN=Tdrd6 PE=1 SV=1
SLC7A5	Q9Z127	1	1	NA	1872	NA	5111	909	5666	2597	NA	2656	NA	0.569952951	0.244160994	2630.6667	3639.6667	Up	0.468377874103584	Large neutral amino acids transporter small subunit 1 OS=Mus musculus GN=Slc7a5 PE=1 SV=2
PCBP2	Q61990	16	16	57332.67	54688.07	46091.53	76447.53	60842.33	65692.33	71496.93	38096.27	60631.13	31772.93	0.570435917	0.243793137	59080.426	53537.918	Down	-0.142119192632446	Poly(rC)-binding protein 2 OS=Mus musculus GN=Pcbp2 PE=1 SV=1
RIMS1	Q99NE5-6	29	29	38435.64	43033.84	37603.6	41468.2	52500.68	49087.56	51767.92	44199.64	58957.72	27001.32	0.570623213	0.243650565	42608.392	46202.832	Up	0.116843677652148	Isoform 6 of Regulating synaptic membrane exocytosis protein 1 OS=Mus musculus GN=Rims1
SLC8A1	P70414	2	2	22902	47473.5	27586	75980.5	33178	56478	55852	70201.5	28396.5	32874	0.570703329	0.243589594	41424	48760.4	Up	0.235243090628984	Sodium/calcium exchanger 1 OS=Mus musculus GN=Slc8a1 PE=1 SV=1
CTTN	Q60598	31	31	116585.97	104839.23	83899.29	96139.58	108550.1	101614.81	118920.58	98997.65	152645.39	78738.13	0.570820855	0.243500168	102002.83	110183.31	Up	0.11129649870381	Src substrate cortactin OS=Mus musculus GN=Cttn PE=1 SV=2
TUBB1	A2AQ07	1	1	72973	73036	152086	48139	61706	53133	80512	114575	47803	221221	0.570825019	0.243497	81588	103448.8	Up	0.342488029444925	Tubulin beta-1 chain OS=Mus musculus GN=Tubb1 PE=1 SV=1
AMOT	Q8VHG2	1	1	23752	24158	36821	18364	24277	21780	33110	40909	34987	13743	0.571314394	0.243124834	25474.4	28905.8	Up	0.182310834176517	Angiomotin OS=Mus musculus GN=Amot PE=1 SV=3
LSM12	Q9D0R8	4	4	49113.75	62769	48451	60224.25	53580.75	52743.75	59269.75	40217.25	62094	44801.25	0.57153219	0.242959304	54827.75	51825.2	Down	-0.081252491200339	Protein LSM12 homolog OS=Mus musculus GN=Lsm12 PE=1 SV=1
PLPPR4	Q7TME0	23	23	21626.09	36582.36	29230.09	74784	43581.82	61404.64	67217.59	37425.09	49635.36	25382.68	0.571886821	0.242689911	41160.872	48213.072	Up	0.228150810800084	Lipid phosphate phosphatase-related protein type 4 OS=Mus musculus GN=Lppr4 PE=2 SV=2
CCT6A	P80317	19	19	49836.37	56127.47	59004	43494.84	50327.79	54021	53103.79	71863.21	55404.74	41097.11	0.572199771	0.24245232	51758.094	55097.97	Up	0.090214675059249	T-complex protein 1 subunit zeta OS=Mus musculus GN=Cct6a PE=1 SV=3
EPB41L1	Q9Z2H5-3	25	25	62562.48	70018.52	44939.8	64276.32	71162.4	75679.6	90669.36	40275.72	88328.48	51272.36	0.572499509	0.242224882	62591.904	69245.104	Up	0.14573600581712	Isoform 3 of Band 4.1-like protein 1 OS=Mus musculus GN=Epb41l1
RPL26	P61255	15	15	91625.07	100528	96768.27	75993.07	91032.93	86341	101291.53	77866.87	113408.73	99088	0.572664434	0.242099788	91189.468	95599.226	Up	0.0681317289591917	60S ribosomal protein L26 OS=Mus musculus GN=Rpl26 PE=1 SV=1
ABI1	Q8CBW3	21	21	101713.19	95845.29	76100.86	94535.52	102191.81	103287	103874.43	66728.9	107488.81	51836.05	0.572873114	0.24194156	94077.334	86643.038	Down	-0.11876334746685	Abl interactor 1 OS=Mus musculus GN=Abi1 PE=1 SV=3
CDC42	P60766-1	6	6	18087.83	25792.17	13695.33	54356	23050.33	31943	33630	15186.83	20838.5	7759	0.573171268	0.241715588	26996.332	21871.466	Down	-0.303713476619859	Isoform 1 of Cell division control protein 42 homolog OS=Mus musculus GN=Cdc42
GAP43	P06837	37	37	161134.62	181800.46	164117.46	289349.57	139454.62	181109.78	182059.22	186051.05	239527.49	235323.27	0.573214723	0.241682664	187171.35	204814.16	Up	0.129955884699825	Neuromodulin OS=Mus musculus GN=Gap43 PE=1 SV=1
TSC2	Q61037-3	3	3	4750	11173.67	11851.67	14341.33	14526.67	16121.33	18550.33	10047.67	13334	6694	0.573283072	0.241630882	11328.668	12949.466	Up	0.192914364200059	Isoform B of Tuberin OS=Mus musculus GN=Tsc2
ALDOA	P05064	187	187	144296.62	122985.96	135905.49	115887.25	140822.58	128067.27	129588.77	129123.48	137467.56	158950.91	0.573905945	0.241159277	131979.58	136639.6	Up	0.0500609038336796	Fructose-bisphosphate aldolase A OS=Mus musculus GN=Aldoa PE=1 SV=2
CKB	Q04447	75	75	157376.57	155525.91	105240.04	152180.16	157226.59	169182.29	160666.84	98888.38	163416.24	67098.29	0.574301885	0.240859759	145509.85	131850.41	Down	-0.142214820962835	Creatine kinase B-type OS=Mus musculus GN=Ckb PE=1 SV=1
TANC2	A2A690	8	8	11055.12	16440.25	6792.88	27146.88	19316.75	26312.75	26495.5	13156.88	22215.62	7619.5	0.574303904	0.240858231	16150.376	19160.05	Up	0.246533572960952	Protein TANC2 OS=Mus musculus GN=Tanc2 PE=1 SV=1
FKBP3	Q62446	7	7	49252.43	54512.43	71213.14	57375.14	45628.71	56883.29	57400.29	51057.29	69026.57	11403	0.574935492	0.240380881	55596.37	49154.088	Down	-0.177679283300605	Peptidyl-prolyl cis-trans isomerase FKBP3 OS=Mus musculus GN=Fkbp3 PE=1 SV=2
EEA1	Q8BL66	7	7	14940.86	21791.29	9959.57	17552.43	19879.43	21714.14	22720.71	9741.29	22008.14	NA	0.575577389	0.239896275	16824.716	19046.07	Up	0.178911187711072	Early endosome antigen 1 OS=Mus musculus GN=Eea1 PE=1 SV=2
CAMKK2	Q8C078	7	7	6380.4	7500.8	4755.2	9769.8	8343.2	10248.2	9437	3329.2	11132.4	NA	0.575710389	0.239795933	7349.88	8536.7	Up	0.215957784865603	Calcium/calmodulin-dependent protein kinase kinase 2 OS=Mus musculus GN=Camkk2 PE=1 SV=2
SPOCK1	Q62288	1	1	32389	39197	NA	26584	27155	26792	30857	NA	52855	NA	0.575789077	0.239736578	31331.25	36834.667	Up	0.233461858084955	Testican-1 OS=Mus musculus GN=Spock1 PE=2 SV=2
GPM6A	P35802	3	3	13881.33	32306	15001	83472.67	48764.67	68019	77205.67	19310.33	34403.67	NA	0.576052122	0.239538219	38685.134	49734.668	Up	0.362472560410569	Neuronal membrane glycoprotein M6-a OS=Mus musculus GN=Gpm6a PE=1 SV=1
AKAP8L	Q9R0L7	2	2	1089	3167	NA	3144	2439	1999	2067	NA	2384	NA	0.576212766	0.239417124	2459.75	2150	Down	-0.194175032949624	A-kinase anchor protein 8-like OS=Mus musculus GN=Akap8l PE=1 SV=1
SSBP1	Q9CYR0	5	5	130003.2	136850.6	194393.2	59673	119175.8	96348.6	124144.2	196893	164676.8	138303.6	0.576623573	0.239107607	128019.16	144073.24	Up	0.170442648378546	Single-stranded DNA-binding protein, mitochondrial OS=Mus musculus GN=Ssbp1 PE=1 SV=1
ATP6V1D	P57746	22	22	67435.95	63827.36	75387.14	40168.36	57835.95	53117.91	60982.36	85351.09	72247.68	57138.05	0.576880305	0.238914288	60930.952	65767.418	Up	0.110197750260062	V-type proton ATPase subunit D OS=Mus musculus GN=Atp6v1d PE=1 SV=1
RABIF	Q91X96	1	1	93665	96777	187758	32667	95756	69707	93685	110552	101921	247439	0.576923675	0.238881639	101324.6	124660.8	Up	0.29902339620662	Guanine nucleotide exchange factor MSS4 OS=Mus musculus GN=Rabif PE=2 SV=1

MFF	Q6PCP5-3	9	9	14756.25	18180.5	6940	42499.12	28972.25	38109	27144	1771.88	15635.12	1103	0.576933296	0.238874397	22269.624	16752.6	Down	-0.410692180975248	Isoform 3 of Mitochondrial fission factor OS=Mus musculus GN=Mff
HSPA2	P17156	44	44	66663.14	69230.66	66359.45	90189.8	65665.55	72857.98	71110.86	77835.11	63246.59	55170.93	0.576938842	0.238870222	71621.72	68044.294	Down	-0.0739229789652578	Heat shock-related 70 kDa protein 2 OS=Mus musculus GN=Hspa2 PE=1 SV=2
SNRPD3	P62320	2	2	15875.5	9349.5	10129.5	13676	13637	14202	15333.5	2831.5	10325	NA	0.577105038	0.238745134	12533.5	10673	Down	-0.231823595459232	Small nuclear ribonucleoprotein Sm D3 OS=Mus musculus GN=Snrpd3 PE=1 SV=1
SET	Q9EQU5	4	4	8525.25	6963	2545.75	21174.25	9466.25	14472.75	6276.5	5341	3681.75	NA	0.577325259	0.238579441	9734.9	7443	Down	-0.387281923246245	Protein SET OS=Mus musculus GN=Set PE=1 SV=1
LIMCH1	Q3UH68-3	2	2	4212	5262	2559	5446	6132	5297	7673	5532	3061	NA	0.577798604	0.238223512	4722.2	5390.75	Up	0.191026859549436	Isoform 3 of LIM and calponin homology domains-containing protein 1 OS=Mus musculus GN=Limch1
NDUFA12	Q7TMF3	8	8	115513.88	119111	123557.75	84183.62	111120.62	100103.88	101092.75	122642.88	123216.75	134523	0.577839786	0.238192559	110697.37	116315.85	Up	0.0714267281576549	NADH dehydrogenase [ubiquinone] 1 alpha subcomplex subunit 12 OS=Mus musculus GN=Ndufa12 PE=2 SV=2
YWHAB	Q9CQV8	25	25	198563.54	291816.67	183774.46	618591.12	290207.46	414422.04	365228.79	184628.33	253671.42	75449.67	0.57827131	0.237868354	316590.65	258680.05	Down	-0.291449854051312	14-3-3 protein beta/alpha OS=Mus musculus GN=Ywhab PE=1 SV=3
ARHGAP44	Q5SSM3	3	3	125436.33	134996.67	132883.33	131251	142310.33	140944.67	169503.67	49613.67	151238	88400.33	0.578834793	0.237445372	133375.53	119940.07	Down	-0.153180329740878	Rho GTPase-activating protein 44 OS=Mus musculus GN=Arhgap44 PE=1 SV=1
RPL12	P35979	10	10	114766.2	135383.4	137905.2	162215.9	106796	130083.6	149203.1	111770.2	150534.9	58342.3	0.579046525	0.237286541	131413.34	119986.82	Down	-0.131235792487006	60S ribosomal protein L12 OS=Mus musculus GN=Rpl12 PE=1 SV=2
VPS37C	Q8R105	7	7	8266.17	10140	7126.33	9709.67	10556.33	15841.67	7865.33	7888.5	9872.17	NA	0.579349403	0.237059436	9159.7	10366.918	Up	0.178614734042219	Vacuolar protein sorting-associated protein 37C OS=Mus musculus GN=Vps37c PE=2 SV=1
PRKCD	P28867	2	2	3165.5	7164.5	8628	29122.5	17995.5	22818	19512	20658	4440	NA	0.579664598	0.236823223	13215.2	16857	Up	0.351149547117471	Protein kinase C delta type OS=Mus musculus GN=Prkcd PE=1 SV=3
CLIP1	Q922J3	23	23	49360.86	53887.57	60320.24	36751.95	53822.52	48510.29	54341.24	44373.19	67852.57	55189.62	0.579883572	0.236659195	50828.628	54053.382	Up	0.0887435978752032	CAP-Gly domain-containing linker protein 1 OS=Mus musculus GN=Clip1 PE=1 SV=1
TMOD2	Q9JKK7-2	3	3	33642.67	40229.67	32436	53447	30538	40044.33	40880.67	31502.67	26838.67	36639.67	0.580028414	0.236550731	38058.668	35181.202	Down	-0.113420295460703	Isoform 2 of Tropomodulin-2 OS=Mus musculus GN=Tmod2
PDCD6IP	Q9WU78	8	8	8374.75	11260.88	7931.25	22610.5	13120.38	16718.25	13896.12	5679.62	10553	6652.75	0.58006555	0.236522927	12659.552	10699.948	Down	-0.242622565766274	Programmed cell death 6-interacting protein OS=Mus musculus GN=Pdcd6ip PE=1 SV=3
MATR3	Q8K310	28	28	95637.25	53854.54	58606.46	38560.11	79521.57	48251.43	55462.57	51584.54	53063.14	83325.36	0.580174409	0.236441432	65235.986	58337.408	Down	-0.161246728082264	Matrin-3 OS=Mus musculus GN=Matr3 PE=1 SV=1
ACLY	Q91V92	7	7	17701.5	21507.17	15382	21451.33	20971	19776	24019.67	9055.5	16603.83	NA	0.580573225	0.236142997	19402.6	17363.75	Down	-0.160171434137161	ATP-citrate synthase OS=Mus musculus GN=Acly PE=1 SV=1
COX6C	Q9CPQ1	7	7	254690.29	246805.71	430663.29	183178.43	232182.29	224822.43	242710.71	405745.14	260543.14	375193.71	0.580645135	0.236089208	269504	301803.03	Up	0.163300574546321	Cytochrome c oxidase subunit 6C OS=Mus musculus GN=Cox6c PE=1 SV=3
EPB41L1	Q9Z2H5	28	28	63775.07	71283	45736.36	73232.11	72020.57	80255.46	93204.89	41967.25	91515.25	52962.43	0.581238762	0.235645431	65209.422	71981.056	Up	0.14253683541022	Band 4.1-like protein 1 OS=Mus musculus GN=Epb41l1 PE=1 SV=2
SH2B1	Q91ZM2-3	2	2	15351	18761	NA	32811.5	21378	26649	26909	13660.5	6224	NA	0.581282624	0.235612659	22075.375	18360.625	Down	-0.265822776242233	Isoform 3 of SH2B adapter protein 1 OS=Mus musculus GN=Sh2b1
SPAG7	Q7TNE3	1	1	20893	27936	20634	20459	25956	24139	23030	18002	30852	27575	0.58170196	0.235299473	23175.6	24719.6	Up	0.0930487087782422	Sperm-associated antigen 7 OS=Mus musculus GN=Spag7 PE=1 SV=1
MRPL38	Q8K2M0	1	1	16334	6324	16178	22046	18894	19069	29613	NA	10512	NA	0.581728752	0.235279471	15955.2	19731.333	Up	0.306461755576146	39S ribosomal protein L38, mitochondrial OS=Mus musculus GN=Mrpl38 PE=2 SV=2
NDUFB10	Q9DCS9	29	29	103203.54	107504.96	88216.07	81444.21	93598.96	96951.64	88199.32	90063.14	100877.14	80840.32	0.581738234	0.235272392	94793.548	91386.312	Down	-0.0528107751924661	NADH dehydrogenase [ubiquinone] 1 beta subcomplex subunit 10 OS=Mus musculus GN=Ndufb10 PE=1 SV=3
TSTD3	Q9D0B5	1	1	200413	180308	79039	156356	201217	126139	182260	179933	120245	132309	0.581868537	0.235175125	163466.6	148177.2	Down	-0.141672412447138	Thiosulfate sulfurtransferase/rhodanese-like domain-containing protein 3 OS=Mus musculus GN=Tstd3 PE=1 SV=1
KALRN	A2CG49-8	2	2	28568	43788.5	56781	33312.5	40443	31310	60225	58796.5	34525	40398.5	0.581922744	0.235134668	40578.6	45051	Up	0.150840038378034	Isoform 8 of Kalirin OS=Mus musculus GN=Kalrn
HNRNPL	Q8R081	14	14	114402.64	69205.64	84442.14	60806.07	101392.29	71260.86	69941.43	93793.43	71556.07	91068.86	0.582037457	0.235049066	86049.756	79524.13	Down	-0.113778417752087	Heterogeneous nuclear ribonucleoprotein L OS=Mus musculus GN=Hnrnpl PE=1 SV=2
HARS	Q61035	2	2	4520.5	10554	4224.5	28117	11617.5	14908.5	16753	NA	11847.5	NA	0.582491563	0.234710361	11806.7	14503	Up	0.296745573524033	Histidine--tRNA ligase, cytoplasmic OS=Mus musculus GN=Hars PE=2 SV=2
TAMM41	Q3TUH1	1	1	NA	NA	NA	5343	1823	2532	1977	NA	2199	NA	0.583697072	0.233812485	3583	2236	Down	-0.680247855367546	Mitochondrial translocator assembly and maintenance protein 41 homolog OS=Mus musculus GN=Tamm41 PE=2 SV=2
ACAA1A	Q921H8	24	24	86791.5	89128.62	99193.92	47195.88	77132.25	70568.88	78031.88	102766.5	97342.08	81594.17	0.583845315	0.2337022	79888.434	86060.702	Up	0.107367959092927	3-ketoacyl-CoA thiolase A, peroxisomal OS=Mus musculus GN=Acaa1a PE=1 SV=1
NEGR1	Q80Z24	31	31	41159.31	96399.97	44713.72	150860.03	96645.86	116105.62	149010.03	86496.79	96458.86	54758.21	0.583935435	0.233635169	85955.778	100565.9	Up	0.226474699155855	Neuronal growth regulator 1 OS=Mus musculus GN=Negr1 PE=1 SV=1
PDXP	P60487	7	7	28106	28516	37414.71	24472.43	33115.86	28015.14	28980	25914.43	30872.86	54514.14	0.584160056	0.233468143	30325	33659.314	Up	0.150498128503204	Pyridoxal phosphate phosphatase OS=Mus musculus GN=Pdxp PE=1 SV=1
HK1	P17710-2	37	37	61774.59	92009.62	75346.16	140573.51	97215.95	127694.57	140381.32	68874.81	87278.59	96133.95	0.584208583	0.233432067	93383.966	104072.65	Up	0.156344188529636	Isoform HK1-SB of Hexokinase-1 OS=Mus musculus GN=Hk1
RNH1	Q91VI7	4	4	25545.5	21793.5	30454.5	24480	22718	25835.75	26107	21062.25	32157	6323	0.584339113	0.233335043	24998.3	22297	Down	-0.164980375734173	Ribonuclease inhibitor OS=Mus musculus GN=Rnh1 PE=1 SV=1
USF1	Q61069	1	1	15175	4852	NA	8602	10905	10453	8667	NA	15924	NA	0.584840153	0.232962818	9883.5	11681.333	Up	0.241111023435386	Upstream stimulatory factor 1 OS=Mus musculus GN=Usf1 PE=1 SV=1
PDLIM1	O70400	1	1	10752	5162	10468	1699	11291	3115	4806	27640	10087	NA	0.584982789	0.232856911	7874.4	11412	Up	0.535309747229506	PDZ and LIM domain protein 1 OS=Mus musculus GN=Pdlim1 PE=2 SV=4
HMGN2	P09602	2	2	174643	76681.5	112461	41637.5	68438.5	50036.5	62575.5	62836.5	72545.5	144581.5	0.585422195	0.232530816	94772.3	78515.1	Down	-0.271495311199318	Non-histone chromosomal protein HMG-17 OS=Mus musculus GN=Hmgn2 PE=1 SV=2
TUFM	Q8BFR5-2	30	30	133731.07	133325.32	108665.75	107420.68	114879.71	125613.29	122059.14	99004.14	127106.93	99859.82	0.585493662	0.232477802	119604.51	114728.66	Down	-0.0600458611782028	Isoform 2 of Elongation factor Tu, mitochondrial OS=Mus musculus GN=Tufm
ECH1	O35459	5	5	24603.8	33758.2	34363.6	27293.2	26700.2	27995.2	27587.4	45895.2	26555.4	30511	0.585515882	0.23246132	29343.8	31708.84	Up	0.111829389468259	Delta(3,5)-Delta(2,4)-dienoyl-CoA isomerase, mitochondrial OS=Mus musculus GN=Ech1 PE=1 SV=1
SH3KBP1	Q8R550-8	1	1	97562	102181	28557	46266	106191	66718	82311	NA	117720	NA	0.58583326	0.232225975	76151.4	88916.333	Up	0.223577896708695	Isoform 8 of SH3 domain-containing kinase-binding protein 1 OS=Mus musculus GN=Sh3kbp1
AGAP2	Q3UHD9	18	18	36794.06	50671.33	35786.5	62990.78	54063.56	62762.17	67604.72	47603.83	58917.89	28295.39	0.585887143	0.232186032	48061.246	53036.8	Up	0.142119681212257	Arf-GAP with GTPase, ANK repeat and PH domain-containing protein 2 OS=Mus musculus GN=Agap2 PE=2 SV=1
NOMO1	Q6GQT9	1	1	1431	4113	NA	12975	3216	9642	7966	3941	6872	NA	0.586097311	0.232030271	5433.75	7105.25	Up	0.386937221443928	Nodal modulator 1 OS=Mus musculus GN=Nomo1 PE=1 SV=1
DLG4	Q62108-3	14	14	41381.71	61638.86	54554.57	86961.43	65397.93	69614.36	79593.93	37827.21	68888.21	85545.64	0.586137513	0.232000483	61986.9	68293.87	Up	0.139792733449388	Isoform 3 of Disks large homolog 4 OS=Mus musculus GN=Dlg4
FADD	Q61160	1	1	NA	NA	NA	1370	704	585	4293	NA	NA	NA	0.586454933	0.231765357	1037	2439	Up	1.23387386398296	FAS-associated death domain protein OS=Mus musculus GN=Fadd PE=1 SV=1
UBA52	P62984	6	6	59900.5	93587	39945.67	65232	81593.33	78450.17	110484	61041.17	139174.67	17033	0.586881587	0.231449516	68051.7	81236.602	Up	0.255498693064587	Ubiquitin-60S ribosomal protein L40 OS=Mus musculus GN=Uba52 PE=1 SV=2
ATP6V1F	Q9D1K2	6	6	178378.17	175273.67	241208.33	154176.33	158785	151436.67	157437.17	189235	212235.33	274989.17	0.587351109	0.231102207	181564.3	197066.67	Up	0.118203217283561	V-type proton ATPase subunit F OS=Mus musculus GN=Atp6v1f PE=1 SV=2
EIF3D	O70194	4	4	18690.5	23855.75	16972.75	38465.75	28885.25	34204.75	32483	16937	30455.5	NA	0.587498599	0.230993165	25374	28520.063	Up	0.168626177164086	Eukaryotic translation initiation factor 3 subunit D OS=Mus musculus GN=Eif3d PE=1 SV=2
VSNL1	P62761	1	1	4328	5888	NA	19374	6181	7933	6941	NA	5526	NA	0.587794398	0.230774557	8942.75	6800	Down	-0.395183798836137	Visinin-like protein 1 OS=Mus musculus GN=Vsnl1 PE=1 SV=2
RAP1GAP	A2ALS5-4	3	3	13454.5	15340.5	7577	22797	18472	16392.5	13915	18455	19893	NA	0.587873943	0.230715789	15528.2	17163.875	Up	0.144484694570379	Isoform 4 of Rap1 GTPase-activating protein 1 OS=Mus musculus GN=Rap1gap
ADAM22	Q9R1V6-18	4	4	12254	23481	941.33	57798.67	26711	44438	49062	18709.33	14272	NA	0.587882926	0.230709153	24237.2	31620.333	Up	0.38362949702798	Isoform 16 of Disintegrin and metalloproteinase domain-containing protein 22 OS=Mus musculus GN=Adam22
SNCG	Q9Z0F7	5	5	62223.4	61002.4	36125.6	192897.6	63793.6	88188.4	61872.8	45431.6	69396.4	NA	0.587916794	0.230684134	83208.52	66222.3	Down	-0.329414139751476	Gamma-synuclein OS=Mus musculus GN=Sncg PE=1 SV=1
NAPA	Q9DB05	12	12	25005.75	24505.08	42572.42	29393	43191.17	33964.5	31142.42	25645.67	38706.42	52621.25	0.588272327	0.230421581	32933.484	36416.052	Up	0.145019381585555	Alpha-soluble NSF attachment protein OS=Mus musculus GN=Napa PE=1 SV=1
STRN	O55106	12	12	36944.33	38719.83	29766.58	41789.33	43738.17	43606.58	41384.5	19427.58	46533.92	22143.33	0.588374899	0.230345863	38191.648	34619.182	Down	-0.141685538382485	Striatin OS=Mus musculus GN=Strn PE=1 SV=2
SGTA	Q8BJU0	13	13	49633	55422.92	62651.69	55108.08	57725.85	57491.69	61374.23	46628.31	63941.62	34094.23	0.588545491	0.230219963	56108.308	52706.016	Down	-0.0902467634295418	Small glutamine-rich tetratricopeptide repeat-containing protein alpha OS=Mus musculus GN=Sgta PE=1 SV=2
FNBP1L	Q8K012	5	5	22875.6	28198.4	24366.2	56659.2	29725.2	38464	38035.2	16353.8	28819.2	18180.4	0.588900252	0.22995826	32364.92	27970.52	Down	-0.210523862596757	Formin-binding protein 1-like OS=Mus musculus GN=Fnbp1l PE=1 SV=2
TAGLN3	Q9R1Q8	41	41	69556.44	69727.46	57524.59	66450.95	74203.98	67526.1	70964.88	76889.37	68291.24	63759.8	0.589005935	0.229880329	67492.684	69486.278	Up	0.0419969787448229	Transgelin-3 OS=Mus musculus GN=Tagln3 PE=1 SV=1
FNBP1	Q80TY0-3	4	4	36824.75	51388.25	43432	44920	39590.5	49658.75	45638.5	36304.25	49340.5	15362.75	0.589522795	0.229499397	43231.1	39260.95	Down	-0.138974463343287	Isoform 3 of Formin-binding protein 1 OS=Mus musculus GN=Fnbp1
LRRC57	Q9D1G5	2	2	150434	140119.5	161368.5	99371	125008.5	117286	116514	95344	130268.5	170553	0.589797586	0.229297009	135260.3	125993.1	Down	-0.102393731862813	Leucine-rich repeat-containing protein 57 OS=Mus musculus GN=Lrrc57 PE=2 SV=1
PTPN11	P35235	1	1	5254	7086	NA	18509	7328	15203	14377	NA	6245	NA	0.589980091	0.229162643	9544.25	11941.667	Up	0.32330046548674	Tyrosine-protein phosphatase non-receptor type 11 OS=Mus musculus GN=Ptpn11 PE=1 SV=2
ADO	Q6PDY2	2	2	25327	27889.5	12732.5	48327.5	34388.5	35532	25416	20005.5	22665	NA	0.590146807	0.229039938	29733	25904.625	Down	-0.198855336595684	2-aminoethanethiol dioxygenase OS=Mus musculus GN=Ado PE=1 SV=2
PPP1R11	Q8K1L5	1	1	17225	9066	10008	20755	13399	20822	20470	NA	8955	NA	0.59071207	0.228624155	14090.6	16749	Up	0.249341916763329	Protein phosphatase 1 regulatory subunit 11 OS=Mus musculus GN=Ppp1r11 PE=2 SV=1
GOLGA3	P55937	6	6	24463.8	26216.8	27414.6	17705	22152.8	25846	29562	24481.6	26706.4	18406	0.591495113	0.228048839	23590.6	25000.4	Up	0.0837390654767349	Golgin subfamily A member 3 OS=Mus musculus GN=Golga3 PE=1 SV=3
PLEKHA6	Q7TQG1	4	4	9769.25	12190.75	5743.25	11971.5	18245.25	17660.25	15824	3209	19240	NA	0.592159884	0.227561018	11584	13983.313	Up	0.271572652982124	Pleckstrin homology domain-containing family A member 6 OS=Mus musculus GN=Plekha6 PE=1 SV=1
DBI	P31786	10	10	57981.9	69597.7	19940.6	122797.9	56120.3	81641.7	70189.8	30038.3	63238.3	25088.7	0.592256278	0.227490327	65287.68	54039.36	Down	-0.272800187543693	Acyl-CoA-binding protein OS=Mus musculus GN=Dbi PE=1 SV=2
CNTFR	O88507	1	1	11748	22079	NA	28916	16164	30619	20302	NA	17619	NA	0.592639751	0.227209222	19726.75	22846.667	Up	0.211830400817	Ciliary neurotrophic factor receptor subunit alpha OS=Mus musculus GN=Cntfr PE=1 SV=2
PRPF31	Q8CCF0-3	1	1	4673	1698	NA	3733	4303	5388	4455	NA	2730	NA	0.592701913	0.227163671	3601.75	4191	Up	0.218596475226354	Isoform 3 of U4/U6 small nuclear ribonucleoprotein Prp31 OS=Mus musculus GN=Prpf31
DNM2	P39054	14	14	68211.46	89883.15	58883.23	133361.08	83081	102290.77	107036.15	67458.54	67942.23	38459.46	0.59302198	0.22692921	86683.984	76637.43	Down	-0.177716279498766	Dynamin-2 OS=Mus musculus GN=Dnm2 PE=1 SV=2
GTPBP8	Q9CY28	1	1	5290	9658	6736	5449	9733	8102	13084	NA	5374	NA	0.593080949	0.226886026	7373.2	8853.3333	Up	0.263929849866021	GTP-binding protein 8 OS=Mus musculus GN=Gtpbp8 PE=2 SV=1
NAV1	Q8CH77	12	12	15978.09	17551.45	13431.91	19316.09	19682.91	19105.91	22045.45	4860.45	23903.82	2866.36	0.593085637	0.226882594	17192.09	14556.398	Down	-0.240091537460226	Neuron navigator 1 OS=Mus musculus GN=Nav1 PE=1 SV=2
BTF3	Q64152	2	2	55732	77518.5	93201.5	63194	70291.5	55323	60632	98584.5	106884	NA	0.593603495	0.226503551	71987.5	80355.875	Up	0.158657089536718	Transcription factor BTF3 OS=Mus musculus GN=Btf3 PE=1 SV=3
BTF3	Q64152-2	2	2	55732	77518.5	93201.5	63194	70291.5	55323	60632	98584.5	106884	NA	0.593603495	0.226503551	71987.5	80355.875	Up	0.158657089536718	Isoform 2 of Transcription factor BTF3 OS=Mus musculus GN=Btf3
TXNDC17	Q9CQM5	3	3	4744.67	4241	5049.33	4210.67	4550.67	4652.67	5704	3041	6707	4513.33	0.593659553	0.226462539	4559.268	4923.6	Up	0.110911344784123	Thioredoxin domain-containing protein 17 OS=Mus musculus GN=Txndc17 PE=1 SV=1
HDAC2	P70288	1	1	1220	4691	2998	5377	3582	5592	4575	NA	2536	NA	0.593726114	0.226413849	3573.6	4234.3333	Up	0.244756685269053	Histone deacetylase 2 OS=Mus musculus GN=Hdac2 PE=1 SV=1
SND1	Q78PY7	16	16	32081.75	33922.38	34509.56	34974.81	36116.44	36963.94	39765.56	25438.44	38793.62	18191.44	0.593963533	0.226240218	34320.988	31830.6	Down	-0.108676732646618	Staphylococcal nuclease domain-containing protein 1 OS=Mus musculus GN=Snd1 PE=1 SV=1
SWI5	Q8K3D3	1	1	2672	3055	NA	3082	2138	2714	2237	5288	2446	NA	0.594884573	0.225567293	2736.75	3171.25	Up	0.212587962645664	DNA repair protein SWI5 homolog OS=Mus musculus GN=Swi5 PE=1 SV=1
PPP1CC	P63087	1	1	7609	5785	5994	8642	5657	7181	5452	8952	7447	NA	0.595026081	0.225463998	6737.4	7258	Up	0.107380102024422	Serine/threonine-protein phosphatase PP1-gamma catalytic subunit OS=Mus musculus GN=Ppp1cc PE=1 SV=1
PPID	Q9CR16	16	16	65766.62	60256.12	51228.25	38923.88	63281.5	52897.69	54797.5	51991.5	53759.25	51817.69	0.595222025	0.225321007	55891.274	53052.726	Down	-0.0751961777150436	Peptidyl-prolyl cis-trans isomerase D OS=Mus musculus GN=Ppid PE=1 SV=3
PPIA	P17742	67	67	338325.22	309442.66	319057.58	221484.12	300639.32	286783	318992.95	293010.37	414199.83	266813.97	0.595655271	0.225005011	297789.78	315960.02	Up	0.0854477946239107	Peptidyl-prolyl cis-trans isomerase A OS=Mus musculus GN=Ppia PE=1 SV=2
ASAP1	Q9QWY8-3	8	8	39224.86	41664.57	34248.71	50898.71	47314.86	48347	48934.86	33497.86	43985.29	22072.57	0.595750751	0.224935402	42670.342	39367.516	Down	-0.116227989072349	Isoform 3 of Arf-GAP with SH3 domain, ANK repeat and PH domain-containing protein 1 OS=Mus musculus GN=Asap1
STX18	Q8VDS8-3	1	1	NA	2856	NA	7924	5881	4105	5114	NA	NA	NA	0.595930779	0.224804183	5553.6667	4609.5	Down	-0.268830320959799	Isoform 3 of Syntaxin-18 OS=Mus musculus GN=Stx18
RPS20	P60867	5	5	38310.6	37308.4	38137	49130.4	42226	47861.2	39796.2	27742.4	55889.8	10279.2	0.596899994	0.224098425	41022.48	36313.76	Down	-0.175898394661156	40S ribosomal protein S20 OS=Mus musculus GN=Rps20 PE=1 SV=1
GRB2	Q60631	1	1	7357	11072	NA	28471	5903	15197	9683	NA	4411	NA	0.597243904	0.223848274	13200.75	9763.6667	Down	-0.435124951359546	Growth factor receptor-bound protein 2 OS=Mus musculus GN=Grb2 PE=1 SV=1
PSMC6	P62334	6	6	36342.5	49403	32760.83	86022.17	41624.67	59426.17	51281	39218.17	40957.83	23923.67	0.598141868	0.223195797	49230.634	42961.368	Down	-0.196516385790299	26S protease regulatory subunit 10B OS=Mus musculus GN=Psmc6 PE=1 SV=1
GPR151	Q7TSN6	1	1	58453	61915	66988	34730	82806	65144	65586	55941	76999	NA	0.598341309	0.223051013	60978.4	65917.5	Up	0.112363231365708	Probable G-protein coupled receptor 151 protein OS=Mus musculus GN=Gpr151 PE=2 SV=1
ECI1	P42125	5	5	87975.2	138844.6	74640.8	237275	157301.6	189772.4	148376.2	71940.4	136264.8	41561.4	0.598537695	0.222908493	139207.44	117583.04	Down	-0.243556335660795	Enoyl-CoA delta isomerase 1, mitochondrial OS=Mus musculus GN=Eci1 PE=1 SV=2
DYNC1I2	O88487	7	7	15008.83	16326.83	18898.33	12967.67	13420.17	14106.67	16344.67	22676.83	19228.17	10710.83	0.598815253	0.222707146	15324.366	16613.434	Up	0.116522921525819	Cytoplasmic dynein 1 intermediate chain 2 OS=Mus musculus GN=Dync1i2 PE=1 SV=1
TSC1	Q9EP53-3	3	3	1371	2456.67	1569.67	4252.33	3549	4258	3694.67	NA	1633.67	NA	0.598819051	0.222704392	2639.734	3195.4467	Up	0.275625048595493	Isoform 3 of Hamartin OS=Mus musculus GN=Tsc1
RANBP2	Q9ERU9	2	2	109089	88279	28502	222022	111984	166705	123565	NA	110626	122344	0.599339256	0.222327276	111975.2	130810	Up	0.224293591398299	E3 SUMO-protein ligase RanBP2 OS=Mus musculus GN=Ranbp2 PE=1 SV=2
LUZP1	Q8R4U7	2	2	3363.5	3408	2611.5	6027.5	3629	6731	3420	NA	3441.5	NA	0.600158813	0.221733812	3807.9	4530.8333	Up	0.250780830053007	Leucine zipper protein 1 OS=Mus musculus GN=Luzp1 PE=1 SV=2
GALNT2	Q6PB93	1	1	12358	18253	NA	48477	20467	43374	42192	NA	11492	NA	0.600591088	0.221421116	24888.75	32352.667	Up	0.378390856221667	Polypeptide N-acetylgalactosaminyltransferase 2 OS=Mus musculus GN=Galnt2 PE=2 SV=1
H2AFY2	Q8CCK0	4	4	146149.75	111400.25	121064.75	62007	120861	89395.5	106782	213353.5	112885.75	110604.25	0.600618811	0.22140107	112296.55	126604.2	Up	0.173011660277042	Core histone macro-H2A.2 OS=Mus musculus GN=H2afy2 PE=1 SV=3
AKT1S1	Q9D1F4	4	4	18993.5	21479.5	6019.25	23932	24425	27893	21592.25	10337.25	27810.25	NA	0.600956453	0.221156997	18969.85	21908.188	Up	0.207761861762492	Proline-rich AKT1 substrate 1 OS=Mus musculus GN=Akt1s1 PE=1 SV=1
SLMAP	Q3URD3	5	5	17514.25	26047.5	18179.75	41923.75	34378.25	38863.75	40169	18185.75	28504.5	NA	0.601063412	0.221079707	27608.7	31430.75	Up	0.187053740199719	Sarcolemmal membrane-associated protein OS=Mus musculus GN=Slmap PE=1 SV=2
SV2B	Q8BG39	16	16	6196.67	11556.2	4164.47	25180.53	12979.93	20254.8	21550.87	11045.47	14476.27	5591.07	0.601273662	0.22092782	12015.56	14583.696	Up	0.279452504925135	Synaptic vesicle glycoprotein 2B OS=Mus musculus GN=Sv2b PE=1 SV=1
SLC16A1	P53986	1	1	NA	1334	NA	4448	1260	4447	3220	NA	1639	NA	0.602098092	0.220332749	2347.3333	3102	Up	0.402175959894116	Monocarboxylate transporter 1 OS=Mus musculus GN=Slc16a1 PE=1 SV=1
SNX15	Q91WE1	2	2	13838.5	13162.5	17234	12075.5	12317.5	15272	11276.5	25342.5	14341.5	10275.5	0.602225919	0.220240557	13725.6	15301.6	Up	0.156813298611849	Sorting nexin-15 OS=Mus musculus GN=Snx15 PE=2 SV=1
TSPAN7	Q62283	2	2	NA	NA	NA	2722.5	1061.5	1778.5	1047	NA	1066.5	NA	0.602313736	0.220177232	1892	1297.3333	Down	-0.54436287927944	Tetraspanin-7 OS=Mus musculus GN=Tspan7 PE=2 SV=2
MIA2	Q8R311	1	1	NA	9114	NA	32847	1729	22919	17875	NA	NA	NA	0.602767484	0.219850184	14563.333	20397	Up	0.486016370498766	cTAGE family member 5 OS=Mus musculus GN=Ctage5 PE=1 SV=1
TIMM50	Q9D880	1	1	76047	82147	167432	21278	99508	67025	69024	156716	136078	NA	0.603045919	0.219649617	89282.4	107210.75	Up	0.2640018579024	Mitochondrial import inner membrane translocase subunit TIM50 OS=Mus musculus GN=Timm50 PE=1 SV=1
MAPT	P10637-5	60	60	122583.76	112495.53	113027.12	106041	111423.57	100350.57	113903.69	128014.41	149308.95	100764.24	0.603153674	0.219572022	113114.2	118468.37	Up	0.0667219470942409	Isoform Tau-D of Microtubule-associated protein tau OS=Mus musculus GN=Mapt
SORBS2	Q3UTJ2-6	2	2	53690.5	62197.5	73507.5	60543	67467.5	58519.5	61257	77224	68628.5	NA	0.604063345	0.218917517	63481.2	66407.25	Up	0.0650113561182119	Isoform 6 of Sorbin and SH3 domain-containing protein 2 OS=Mus musculus GN=Sorbs2
MAPT	P10637-2	63	63	118590.4	108783.78	109315.38	102579.77	107791.75	97112.5	110192.48	123747.27	144402.37	97405.43	0.604231326	0.218796763	109412.22	114572.01	Up	0.0664808103242362	Isoform Tau-A of Microtubule-associated protein tau OS=Mus musculus GN=Mapt
FAM126B	Q8C729	3	3	13882.67	19988.67	27222.67	34119.67	21624	30520	23058	30066	19335	NA	0.604637891	0.21850464	23367.536	25744.75	Up	0.139772644064702	Protein FAM126B OS=Mus musculus GN=Fam126b PE=2 SV=1
SPEG	Q62407-2	4	4	12679	13486.25	1310.25	21701.75	18171.5	21644.75	22934.5	3494.75	18133	NA	0.60479188	0.218394048	13469.75	16551.75	Up	0.297260685328315	Isoform 2 of Striated muscle-specific serine/threonine-protein kinase OS=Mus musculus GN=Speg

MAPK1	P63085	6	6	29163.17	45605.5	15508.5	87121.67	45558.33	62338	64598.83	12106.17	32023.17	5582.5	0.604886852	0.218325855	44591.434	35329.734	Down	-0.335883708393238	Mitogen-activated protein kinase 1 OS=Mus musculus GN=Mapk1 PE=1 SV=3
PYM1	Q8CHP5	3	3	33796	41491.67	17492	38254	40073.67	44405	39735.33	16253	58200.67	NA	0.604925783	0.218297905	34221.468	39648.5	Up	0.212364637754771	Partner of Y14 and mago OS=Mus musculus GN=Wibg PE=2 SV=2
EVL	P70429	1	1	115731	112418	72572	70947	98751	91891	104244	67039	169302	91536	0.605054955	0.218205178	94083.8	104802.4	Up	0.15565351908356	Ena/VASP-like protein OS=Mus musculus GN=Evl PE=1 SV=2
ACAA1B	Q8VCH0	11	11	111278.18	112364.27	140821.09	54937.91	99503.82	86312.73	93862.82	147852.55	117212.36	121389.64	0.605810855	0.21766295	103781.05	113326.02	Up	0.126936053306429	3-ketoacyl-CoA thiolase B, peroxisomal OS=Mus musculus GN=Acaa1b PE=1 SV=1
SLMAP	Q3URD3-4	1	1	2639	4797	NA	18688	12344	16085	17388	NA	4508	NA	0.605988369	0.217535711	9617	12660.333	Up	0.396656565874138	Isoform 4 of Sarcolemmal membrane-associated protein OS=Mus musculus GN=Slmap
TUFM	Q8BFR5	35	35	127828.3	131133.21	108642.39	108735.73	114172.03	122704.33	123784.33	101644.3	123292.09	99324	0.60611493	0.217445018	118102.33	114149.81	Down	-0.0491089936498052	Elongation factor Tu, mitochondrial OS=Mus musculus GN=Tufm PE=1 SV=1
CYTH2	P63034-3	1	1	27885	29871	NA	24364	40313	24544	36980	NA	19968	NA	0.606390279	0.21724777	30608.25	27164	Down	-0.17222462495747	Isoform 3 of Cytohesin-2 OS=Mus musculus GN=Cyth2
HNRNPA1	P49312-2	10	10	92788.3	67525.9	57531.8	74285.7	76961.3	94746.8	76481.5	87171.7	85056.5	51175.3	0.606525596	0.217150866	73818.6	78926.36	Up	0.0965228386539688	Isoform Short of Heterogeneous nuclear ribonucleoprotein A1 OS=Mus musculus GN=Hnrnpa1
SDK2	Q6V4S5	2	2	6263	8431.5	13112.5	18997.5	15761.5	26867.5	20310	NA	3365.5	NA	0.606570169	0.217118952	12513.2	16847.667	Up	0.429098020828921	Protein sidekick-2 OS=Mus musculus GN=Sdk2 PE=2 SV=1
TMOD2	Q9JKK7-3	14	14	147096.55	157949.27	140778.82	120412	136250.27	139377.91	137884.55	164686.64	137491.82	144624.18	0.606934905	0.216857885	140497.38	144813.02	Up	0.0436480717756166	Isoform 3 of Tropomodulin-2 OS=Mus musculus GN=Tmod2
SLC25A46	Q9CQS4	1	1	15226	53506	5032	135585	34691	93006	75520	NA	27437	NA	0.607134329	0.21671521	48808	65321	Up	0.420429241516781	Solute carrier family 25 member 46 OS=Mus musculus GN=Slc25a46 PE=1 SV=1
TACO1	Q8K0Z7	4	4	15772.5	17385.5	11131.5	16069.5	14861.25	14919.25	14904.5	20374.75	17334	12304	0.607412927	0.21651597	15044.05	15967.3	Up	0.0859273736034299	Translational activator of cytochrome c oxidase 1 OS=Mus musculus GN=Taco1 PE=1 SV=1
OCIAD1	Q9CRD0-3	3	3	10979.33	13428	10224	17900	12916.67	14848.33	12963	3160.67	14581	NA	0.607746158	0.216277778	13089.6	11388.25	Down	-0.200874941966982	Isoform 3 of OCIA domain-containing protein 1 OS=Mus musculus GN=Ociad1
ILK	O55222	1	1	4822	7353	NA	10490	3464	10800	7238	NA	5251	NA	0.608037449	0.216069671	6532.25	7763	Up	0.249034281278204	Integrin-linked protein kinase OS=Mus musculus GN=Ilk PE=1 SV=2
HSCB	Q8K3A0	1	1	8301	10114	7158	13393	9059	13001	9762	7058	6768	7175	0.608123209	0.216008421	9605	8752.8	Down	-0.134041008382983	Iron-sulfur cluster co-chaperone protein HscB, mitochondrial OS=Mus musculus GN=Hscb PE=2 SV=2
RTCB	Q99LF4	30	30	54465.8	59693.53	66705.03	37022.93	53271.5	45846.23	55392.27	53473.4	80103.63	56573.93	0.608142723	0.215994486	54231.758	58277.892	Up	0.103810755737791	tRNA-splicing ligase RtcB homolog OS=Mus musculus GN=Rtcb PE=2 SV=1
APPL2	Q8K3G9	1	1	1903	4861	3513	1505	2810	942	4027	7197	2711	NA	0.608579618	0.215682596	2918.4	3719.25	Up	0.349834091457247	DCC-interacting protein 13-beta OS=Mus musculus GN=Appl2 PE=2 SV=1
COPS4	O88544	2	2	3071	3327	1258	2253.5	2536	3303.5	3058.5	NA	1990	NA	0.609853325	0.214774604	2489.1	2784	Up	0.161535019193918	COP9 signalosome complex subunit 4 OS=Mus musculus GN=Cops4 PE=1 SV=1
IPO5	Q8BKC5	1	1	2097	1326	3228	1533	3397	2126	3083	3158	2018	NA	0.610027426	0.214650639	2316.2	2596.25	Up	0.164669478057804	Importin-5 OS=Mus musculus GN=Ipo5 PE=1 SV=3
U2AF1L4	Q8BGJ9	1	1	51899	34583	16219	44375	38274	31140	36258	23584	42003	NA	0.610297505	0.214458405	37070	33246.25	Down	-0.157060493686323	Splicing factor U2AF 26 kDa subunit OS=Mus musculus GN=U2af1l4 PE=1 SV=1
PPM1B	P36993-3	4	4	20683.75	26406	26168	31990.75	25104	35288.5	37984.75	19562.75	35247.75	15679.5	0.610544506	0.214282672	26070.5	28752.65	Up	0.141276676527697	Isoform Beta-3 of Protein phosphatase 1B OS=Mus musculus GN=Ppm1b
LIN7A	Q8JZS0	15	15	69083.47	73218.8	65308.6	61326.87	76813.93	75460.93	71119.27	71894.87	79217	14041.47	0.611752196	0.213424463	69150.334	62346.708	Down	-0.149422834093832	Protein lin-7 homolog A OS=Mus musculus GN=Lin7a PE=1 SV=2
SGIP1	Q8VD37-8	18	18	57126.18	57477.18	60529.88	46461.12	57100.41	48842.18	53013.71	52508.12	61675.71	54210.88	0.612043051	0.213218029	55738.954	54050.12	Down	-0.0443881080288335	Isoform 7 of SH3-containing GRB2-like protein 3-interacting protein 1 OS=Mus musculus GN=Sgip1
CACYBP	Q9CXW3	4	4	71693.5	77314.25	55433.5	83368	86030.25	84711	88534.5	71448	63711.5	37086.5	0.612399597	0.212965103	74767.9	69098.3	Down	-0.113768795795946	Calcyclin-binding protein OS=Mus musculus GN=Cacybp PE=1 SV=1
DLGAP1	Q9D415-3	14	14	17144.67	20757.42	12101.08	22997.42	25311.17	20668.5	21084.83	13573.75	27706.75	2153.17	0.612697729	0.212753729	19662.352	17037.4	Down	-0.206730717164817	Isoform 3 of Disks large-associated protein 1 OS=Mus musculus GN=Dlgap1
ATP12A	Q9Z1W8	11	11	111125.27	276452.64	143047.36	278380.82	270789.73	279025	418857.91	177623.27	171042.36	190700.09	0.612761704	0.212708385	215959.16	247449.73	Up	0.196376907625251	Potassium-transporting ATPase alpha chain 2 OS=Mus musculus GN=Atp12a PE=1 SV=3
PSMB7	P70195	6	6	54993.67	60799.67	53574.17	65132.67	52681	55900.17	61662.17	54346.83	61979.17	60895.17	0.612822007	0.212665647	57436.236	58956.702	Up	0.0376946159149214	Proteasome subunit beta type-7 OS=Mus musculus GN=Psmb7 PE=1 SV=1
RPS3A1	P97351	37	37	84737.84	101553.95	81491.38	121419.86	85363.41	92556.76	99322.51	89282.54	99725.76	69006.73	0.613337229	0.212300673	94913.288	89978.86	Down	-0.0770239922131288	40S ribosomal protein S3a OS=Mus musculus GN=Rps3a PE=1 SV=3
FTL2-PS	P49945	1	1	5723	8027	4421	26857	5760	12197	6870	2848	8777	NA	0.613363554	0.212282033	10157.6	7673	Down	-0.404696907735105	Ferritin light chain 2 OS=Mus musculus GN=Ftl2 PE=2 SV=2
CACNB2	Q8CC27-3	3	3	55522.67	61184.67	46242.67	26521.33	59306.33	50562.67	53856.67	29282.67	58679.33	33820.67	0.613494239	0.212189511	49755.534	45240.402	Down	-0.137245247643202	Isoform 3 of Voltage-dependent L-type calcium channel subunit beta-2 OS=Mus musculus GN=Cacnb2
MRPL12	Q9DB15	2	2	25303.5	39140	26739.5	31982	29877.5	30778	30357.5	32291	37922.5	29273.5	0.614605751	0.21140338	30608.5	32124.5	Up	0.0697416538430776	39S ribosomal protein L12, mitochondrial OS=Mus musculus GN=Mrpl12 PE=1 SV=2
TUBB5	P99024	101	101	61100.95	74462.03	60895.01	77975.35	55379.7	63742.75	68735.42	81006.63	56154.99	76375.04	0.615415126	0.210831834	65962.608	69202.966	Up	0.0691854317486787	Tubulin beta-5 chain OS=Mus musculus GN=Tubb5 PE=1 SV=1
ANKS1B	Q8BIZ1-3	3	3	5512.67	8797	3584.33	18081.67	8683.33	14313.33	10921.67	NA	14483.67	3528.67	0.615552624	0.210734813	8931.8	10811.835	Up	0.275588548158864	Isoform 3 of Ankyrin repeat and sterile alpha motif domain-containing protein 1B OS=Mus musculus GN=Anks1b
GM13889	Q3UPL5	1	1	31434	35826	45219	12166	29619	26560	33041	23527	33293	22237	0.616266749	0.210231264	30852.8	27731.6	Down	-0.153870568572749	Uncharacterized protein C11orf96 homolog OS=Mus musculus GN=Ag2 PE=1 SV=2
RPH3A	P47708	45	45	59641.14	57117.79	39438.3	54167.07	69482.51	63939.12	65253.23	25102.02	73222.7	20184.74	0.617189432	0.209581519	55969.362	49540.362	Down	-0.17603289176622	Rabphilin-3A OS=Mus musculus GN=Rph3a PE=1 SV=2
RGS20	Q9QZB1	1	1	2120	5054	NA	7917	4034	5577	5922	NA	4898	NA	0.617230321	0.209552748	4781.25	5465.6667	Up	0.193009634546646	Regulator of G-protein signaling 20 OS=Mus musculus GN=Rgs20 PE=1 SV=1
CLSTN1	Q9EPL2	2	2	56629.5	61565	54950	78173.5	46042	63631.5	84874.5	45413.5	65335.5	59599.5	0.617353892	0.20946581	59472	63770.9	Up	0.100687648813194	Calsyntenin-1 OS=Mus musculus GN=Clstn1 PE=1 SV=1
UHRF1BP1L	A2RSJ4	3	3	3092.67	6035.33	1247.67	13119.67	7239.33	9500.33	8027.67	NA	4803.67	NA	0.617822824	0.209136052	6146.934	7443.89	Up	0.276189741682539	UHRF1-binding protein 1-like OS=Mus musculus GN=Uhrf1bp1l PE=1 SV=2
CPSF7	Q8BTV2	2	2	6902	2777	2988.5	6253	5674.5	6065.5	8587	2790.5	5326.5	NA	0.617866405	0.209105418	4919	5692.375	Up	0.210665651219827	Cleavage and polyadenylation specificity factor subunit 7 OS=Mus musculus GN=Cpsf7 PE=1 SV=2
PURB	O35295	28	28	55064.7	64080.59	42391.67	68381.67	58941.07	60391.56	62597.7	52499.7	69878.56	15768.26	0.618055407	0.20897259	57771.94	52227.156	Down	-0.145568796933544	Transcriptional activator protein Pur-beta OS=Mus musculus GN=Purb PE=1 SV=3
TOMM40	Q9QYA2	3	3	20547.67	28590	12992	29779.67	32119.33	41991.33	21250	15287.33	35920.33	NA	0.618067953	0.208963774	24805.734	28612.248	Up	0.205959179097538	Mitochondrial import receptor subunit TOM40 homolog OS=Mus musculus GN=Tomm40 PE=1 SV=3
OSTF1	Q62422	3	3	12554	14569	10375	14806.5	18777	18391	15644.5	2591	12213.5	NA	0.61814823	0.20890737	14216.3	12210	Down	-0.219482831000955	Osteoclast-stimulating factor 1 OS=Mus musculus GN=Ostf1 PE=1 SV=2
ILDR2	B5TVM2	10	10	21731.38	43848	14941.88	66867	54628.5	62918.38	71510.88	20929.88	53016.12	29323	0.61832122	0.208785849	40403.352	47539.652	Up	0.234656353647893	Immunoglobulin-like domain-containing receptor 2 OS=Mus musculus GN=Ildr2 PE=2 SV=2
SRPRB	P47758	1	1	11562	16782	NA	32772	23988	26560	30638	16451	23092	NA	0.618476807	0.208676582	21276	24185.25	Up	0.18490050875436	Signal recognition particle receptor subunit beta OS=Mus musculus GN=Srprb PE=1 SV=1
DHODH	O35435	2	2	18492.5	20646.5	36450.5	28248	16344	23637.5	24718.5	NA	30474	NA	0.618570083	0.208611088	24036.3	26276.667	Up	0.12856744026549	Dihydroorotate dehydrogenase (quinone), mitochondrial OS=Mus musculus GN=Dhodh PE=2 SV=2
SLC9A3R1	P70441	25	25	61432.96	55985.08	47409.56	48447.68	60235	59360.04	54130.24	31485.68	75911.36	27427.28	0.618604241	0.208587107	54702.056	49662.92	Down	-0.139425969085684	Na(+)/H(+) exchange regulatory cofactor NHE-RF1 OS=Mus musculus GN=Slc9a3r1 PE=1 SV=3
MRPL15	Q9CPR5-2	1	1	4065	4136	1306	8740	3865	6256	4582	NA	4530	NA	0.618916615	0.208367858	4422.4	5122.6667	Up	0.212065497443475	Isoform 2 of 39S ribosomal protein L15, mitochondrial OS=Mus musculus GN=Mrpl15
SUGP2	Q8CH09	2	2	19313.5	15490	9717.5	21318	21465	23063.5	17677	8021.5	12844	NA	0.619045519	0.208277416	17460.8	15401.5	Down	-0.181048793997329	SURP and G-patch domain-containing protein 2 OS=Mus musculus GN=Sugp2 PE=2 SV=2
SEC31A	Q3UPL0-3	1	1	81934	74733	158281	80401	102592	91088	72594	69447	77574	325042	0.619529493	0.207938014	99588.2	127149	Up	0.352473400002509	Isoform 3 of Protein transport protein Sec31A OS=Mus musculus GN=Sec31a
APPL1	Q8K3H0	3	3	762	219	NA	2488.33	1277.33	2098.33	1658.67	NA	785.33	NA	0.619754785	0.207780111	1186.665	1514.11	Up	0.351557306626633	DCC-interacting protein 13-alpha OS=Mus musculus GN=Appl1 PE=1 SV=1
FMN2	Q9JL04	1	1	2558	7926	NA	12509	7626	7078	8923	NA	2558	NA	0.619898254	0.207679587	7654.75	6186.3333	Down	-0.307270689730038	Formin-2 OS=Mus musculus GN=Fmn2 PE=1 SV=2
DLGAP1	Q9D415-4	12	12	18649.64	22508	13201.18	24902.91	27428.09	22469.91	22885.82	14807.73	30225.55	2348.91	0.620185589	0.20747833	21337.964	18547.584	Down	-0.202191251128151	Isoform 4 of Disks large-associated protein 1 OS=Mus musculus GN=Dlgap1
DLGAP1	Q9D415-5	11	11	18649.64	22508	13201.18	24902.91	27428.09	22469.91	22885.82	14807.73	30225.55	2348.91	0.620185589	0.20747833	21337.964	18547.584	Down	-0.202191251128151	Isoform 5 of Disks large-associated protein 1 OS=Mus musculus GN=Dlgap1
VAMP4	O70480	2	2	NA	1058	NA	3291	2237	2470	3160	NA	2139	NA	0.620241088	0.207439467	2195.3333	2589.6667	Up	0.238326399713061	Vesicle-associated membrane protein 4 OS=Mus musculus GN=Vamp4 PE=1 SV=1
CNTN2	Q61330	6	6	8957.6	5518.2	3885.6	13564.8	10273	13965.4	13953.6	5989.6	5882.2	NA	0.620326585	0.207379606	8439.84	9947.7	Up	0.237147350806507	Contactin-2 OS=Mus musculus GN=Cntn2 PE=1 SV=2
L1CAM	P11627	27	27	101899.56	121812.33	92570.11	105655	120103.41	120469.22	122149.37	72513.78	126984.67	59986.78	0.620475584	0.207275304	108408.08	100420.76	Down	-0.110414709811296	Neural cell adhesion molecule L1 OS=Mus musculus GN=L1cam PE=1 SV=1
BCAS1	Q80YN3	27	27	112788.56	111227.56	113484.04	80093.81	100000.7	92726.81	80988.37	106784.04	95837.26	118179.59	0.620625794	0.207170179	103518.93	98903.214	Down	-0.0658053568981498	Breast carcinoma-amplified sequence 1 homolog OS=Mus musculus GN=Bcas1 PE=2 SV=3
ACAD9	Q8JZN5	4	4	19883.75	45493.75	54811.75	51126.75	42990.25	54354.25	63015	27519.5	46790	NA	0.620899031	0.206979018	42861.25	47919.688	Up	0.160944574668592	Acyl-CoA dehydrogenase family member 9, mitochondrial OS=Mus musculus GN=Acad9 PE=1 SV=2
AIP	O08915	3	3	27034	29467.33	20361.67	43332	31449.33	38739.67	34156	7406.67	41374	8025	0.620968918	0.206930137	30328.866	25940.268	Down	-0.225498171660994	AH receptor-interacting protein OS=Mus musculus GN=Aip PE=1 SV=1
HIP1	Q8VD75	3	3	12402.67	24031.67	5279	61260.67	22570	47671.33	47609	17961.33	13104.67	30228.67	0.622089021	0.206147464	25108.802	31315	Up	0.318660683064856	Huntingtin-interacting protein 1 OS=Mus musculus GN=Hip1 PE=1 SV=2
ALG2	Q9DBE8	3	3	8951.33	13624.67	16697.67	38092	12244.67	22643.67	22778.33	19337.67	12232.33	27651	0.622405089	0.205926865	17922.068	20928.6	Up	0.223738689651863	Alpha-1,3/1,6-mannosyltransferase ALG2 OS=Mus musculus GN=Alg2 PE=2 SV=2
PEBP1	P70296	28	28	51753.39	55672.61	66526.43	77166.18	45551.5	55796.39	51246.75	74961.89	54787.43	80925.43	0.622414763	0.205920115	59334.022	63543.578	Up	0.0988867470940448	Phosphatidylethanolamine-binding protein 1 OS=Mus musculus GN=Pebp1 PE=1 SV=3
ATP6V1E2	Q9D593	14	14	224526	191419.43	76031.57	208397.14	214769.93	203996.29	209159.64	86621.93	251113.64	39111	0.623401369	0.205232249	183028.81	158000.5	Down	-0.212141664200545	V-type proton ATPase subunit E 2 OS=Mus musculus GN=Atp6v1e2 PE=2 SV=1
LANCL2	Q9JJK2	3	3	14657	35009.67	7811.33	47100.33	33378.67	41711	45399	20118.67	22895.33	NA	0.623880857	0.20489834	27591.4	32531	Up	0.237596509834576	LanC-like protein 2 OS=Mus musculus GN=Lancl2 PE=1 SV=1
MYH11	O08638	3	3	16595.33	30215.33	22171.33	147347.33	35250.33	59350.33	54322.33	13317.67	31443.67	25322.33	0.623910889	0.204877434	50315.93	36751.266	Down	-0.453221280463445	Myosin-11 OS=Mus musculus GN=Myh11 PE=1 SV=1
TPT1	P63028	10	10	65228.8	79067.8	42518.4	134701.4	63594.6	82181.4	83873.6	62221.4	60740.9	52590.1	0.624776583	0.204275256	77022.2	68321.48	Down	-0.172935104319673	Translationally-controlled tumor protein OS=Mus musculus GN=Tpt1 PE=1 SV=1
TUBB4B	P68372	106	106	56956.08	64534.64	60855.05	68167.37	50546.37	57845.06	61927.71	69027.93	51231.44	73858.95	0.624831557	0.204237045	60211.902	62778.218	Up	0.0602153870508522	Tubulin beta-4B chain OS=Mus musculus GN=Tubb4b PE=1 SV=1
STAM2	O88811	1	1	39833	41384	25553	123772	46932	68471	49528	31059	32656	NA	0.62484908	0.204224865	55494.8	45428.5	Down	-0.288754923753562	Signal transducing adapter molecule 2 OS=Mus musculus GN=Stam2 PE=1 SV=1
GPX4	Q91XR9	2	2	82109.5	97808.5	128151	88137.5	75711	96262	71285	178995	85745	NA	0.625715155	0.203623326	94383.5	108071.75	Up	0.195382874592424	Phospholipid hydroperoxide glutathione peroxidase, nuclear OS=Mus musculus GN=Gpx4 PE=2 SV=3
DLGAP4	B1AZP2-3	10	10	21224.22	21552.78	14521.56	26488.67	24502.56	23601.22	24964.89	19998.89	17714.44	15088.33	0.626014642	0.203415509	21657.958	20273.554	Down	-0.0952982075385465	Isoform 3 of Disks large-associated protein 4 OS=Mus musculus GN=Dlgap4
PDCD5	P56812	2	2	23658.5	36858	34750	18216.5	33493	23895.5	32715.5	11934.5	36122	68436.5	0.626202075	0.203285497	29395.2	34620.8	Up	0.236058468375584	Programmed cell death protein 5 OS=Mus musculus GN=Pdcd5 PE=1 SV=3
RPL36	P47964	2	2	32952.5	38763.5	14233.5	33538.5	33122.5	26836	30877.5	22727	38220.5	20614.5	0.626296294	0.203220158	30522.1	27855.1	Down	-0.131912731475941	60S ribosomal protein L36 OS=Mus musculus GN=Rpl36 PE=2 SV=2
AP2B1	Q9DBG3	10	10	20803.4	30352.1	27463.6	60608.5	36356.2	44609.3	43653	47482.3	27964.9	31967.7	0.626321954	0.203202365	35116.76	39135.44	Up	0.15631592247544	AP-2 complex subunit beta OS=Mus musculus GN=Ap2b1 PE=1 SV=1
DNAJC28	Q8VCE1	1	1	NA	1860	NA	2824	1981	1091	3535	NA	3449	NA	0.627207872	0.2025885	2221.6667	2691.6667	Up	0.276857384492314	DnaJ homolog subfamily C member 28 OS=Mus musculus GN=Dnajc28 PE=2 SV=2
PPP3CA	P63328	17	17	20651.4	28209.53	25686	69807	30781.6	44583.07	40475.4	25482.2	31551.87	4407.73	0.627405946	0.20245137	35027.106	29300.054	Down	-0.257568471570674	Serine/threonine-protein phosphatase 2B catalytic subunit alpha isoform OS=Mus musculus GN=Ppp3ca PE=1 SV=1
CNN3	Q9DAW9	6	6	50209.5	45539.17	76281.17	44551.67	59047.83	54992.67	49719	83001.5	61421.33	48227.5	0.628186831	0.201911172	55125.868	59472.4	Up	0.109490829422928	Calponin-3 OS=Mus musculus GN=Cnn3 PE=1 SV=1
EPS15L1	Q60902-4	6	6	19514.17	24671.33	26849.83	21891.5	22506.67	21063.67	20576.83	22810.17	23159.17	24211	0.628592025	0.201631133	23086.7	22364.168	Down	-0.0458728821562907	Isoform 4 of Epidermal growth factor receptor substrate 15-like 1 OS=Mus musculus GN=Eps15l1
ZFP512	Q69Z99	1	1	93598	84107	203396	43661	73897	81145	63712	160650	97175	186914	0.628751729	0.201520808	99731.8	117919.2	Up	0.241673148031416	Zinc finger protein 512 OS=Mus musculus GN=Znf512 PE=2 SV=2
THEM4	Q3UUI3	1	1	126185	127231	125814	226357	171215	240759	180600	139236	126794	NA	0.629043604	0.201319249	155360.4	171847.25	Up	0.14550794472499	Acyl-coenzyme A thioesterase THEM4 OS=Mus musculus GN=Them4 PE=1 SV=1
ATAT1	Q8K341-3	2	2	33471.5	34362.5	55821.5	20689	28742	28087.5	31019	34164.5	68270.5	35099.5	0.629466847	0.201027139	34617.3	39328.2	Up	0.18407095243121	Isoform 3 of Alpha-tubulin N-acetyltransferase 1 OS=Mus musculus GN=Atat1
MRPS31	Q61733	1	1	141196	156436	118859	94631	159237	128333	135932	68239	157438	NA	0.629794046	0.20080145	134071.8	122485.5	Down	-0.130394848668051	28S ribosomal protein S31, mitochondrial OS=Mus musculus GN=Mrps31 PE=2 SV=1
SLC25A31	Q3V132	3	3	121677	272569	47285	589993	223009.33	413782.33	346942.33	41722.33	112494	31310.67	0.629820502	0.200783206	250906.67	189250.33	Down	-0.406854969151388	ADP/ATP translocase 4 OS=Mus musculus GN=Slc25a31 PE=2 SV=1
PRDX6	O08709	7	7	72283.57	62753.71	94982.86	65997.71	58173.43	59373.57	53845.57	80805.14	56295.43	81632.57	0.630599096	0.200246656	70838.256	66390.456	Down	-0.093552833163117	Peroxiredoxin-6 OS=Mus musculus GN=Prdx6 PE=1 SV=3
CDC42SE2	Q8BGH7	3	3	18655.67	17171.33	18959.67	12488.33	16559.67	17495.67	21533.67	9980	25027.33	16853	0.630995523	0.199973722	16766.934	18177.934	Up	0.116569338445241	CDC42 small effector protein 2 OS=Mus musculus GN=Cdc42se2 PE=1 SV=1
ATP6V1H	Q8BVE3	11	11	14927.09	20233	27279.82	39718.82	20327	32600.18	27410.91	13145.27	26453.09	6724	0.631197138	0.199834979	24497.146	21266.69	Down	-0.204018174025241	V-type proton ATPase subunit H OS=Mus musculus GN=Atp6v1h PE=1 SV=1
EIF4E	P63073	2	2	47202.5	30458.5	60674	59533	38124	50729.5	34248	34319.5	34438	134052.5	0.632131748	0.199192397	47198.4	57557.5	Up	0.286265976389359	Eukaryotic translation initiation factor 4E OS=Mus musculus GN=Eif4e PE=1 SV=1
NRGN	P60761	5	5	277099.2	252272.6	355385.2	46421.8	235058.4	143797	163326	381422.8	387127.2	271320	0.632133843	0.199190958	233247.44	269398.6	Up	0.207881106569591	Neurogranin OS=Mus musculus GN=Nrgn PE=1 SV=1
CCDC115	Q8VE99	1	1	11964	11248	6960	12916	12043	14638	10836	4039	13117	6896	0.632189623	0.199152637	11026.2	9905.2	Down	-0.154677664694115	Coiled-coil domain-containing protein 115 OS=Mus musculus GN=Ccdc115 PE=2 SV=1
CARMIL2	Q3V3V9	6	6	8689.33	9320.33	2510	10169.83	10513.83	14253.17	10603.83	839.17	14502.33	NA	0.632638921	0.198844093	8240.664	10049.625	Up	0.286309174649911	Leucine-rich repeat-containing protein 16C OS=Mus musculus GN=Rltpr PE=2 SV=2
RPL17	Q9CPR4	6	6	210014.5	226638.67	209562.67	249725.67	204214.83	207812.33	238446.5	270051.33	231438.5	51705.5	0.632690282	0.198808836	220031.27	199890.83	Down	-0.138496251150098	60S ribosomal protein L17 OS=Mus musculus GN=Rpl17 PE=2 SV=3
CACNB1	Q8R3Z5-2	10	10	37410.25	36936.12	36083.88	22232.12	39266.75	38638.25	40763.12	28759.75	46470	28831.38	0.632713586	0.19879284	34385.824	36692.5	Up	0.0936712864190452	Isoform 2 of Voltage-dependent L-type calcium channel subunit beta-1 OS=Mus musculus GN=Cacnb1
ACO2	Q99KI0	131	131	132727.5	123975.3	107560.36	126214.58	137431.15	138430.12	135122.93	113158.29	127599.04	86627.04	0.632885487	0.198674863	125581.78	120187.48	Down	-0.0633404778939829	Aconitate hydratase, mitochondrial OS=Mus musculus GN=Aco2 PE=1 SV=1
METAP2	O08663	4	4	16185.75	20138.5	17446.25	28548.75	20329.25	25414.25	19735.25	31122.25	22014	13432.25	0.633290063	0.198397327	20529.7	22343.6	Up	0.122149105986948	Methionine aminopeptidase 2 OS=Mus musculus GN=Metap2 PE=1 SV=1
CALM1	P62204	21	21	75759.95	113223	33710.11	240742.21	89728.95	153671.11	111580.89	44056	124336.16	10194.53	0.633343668	0.198360567	110632.84	88767.738	Down	-0.317672407867583	Calmodulin OS=Mus musculus GN=Calm1 PE=1 SV=2
PNPO	Q91XF0	5	5	18492.6	19060.2	10605.6	17593	18735	25324.2	22119	3065	29804.4	NA	0.633640627	0.198156985	16897.28	20078.15	Up	0.248835314796459	Pyridoxine-5'-phosphate oxidase OS=Mus musculus GN=Pnpo PE=1 SV=1
ANK2	Q8C8R3-2	133	133	29027.79	39618.01	32156.82	63341.13	46936.77	53193.01	62683.94	30971.35	48533.92	36426.35	0.634088142	0.197850369	42216.104	46361.714	Up	0.135140461248585	Isoform 2 of Ankyrin-2 OS=Mus musculus GN=Ank2
AP3B2	Q9JME5	5	5	23461.4	33364.2	19453.8	44559.6	32560.6	44924.6	39685.2	38806.8	31865.8	14922.2	0.634801425	0.197362107	30679.92	34040.92	Up	0.149975307335554	AP-3 complex subunit beta-2 OS=Mus musculus GN=Ap3b2 PE=1 SV=2
KIF2C	Q922S8	2	2	23456.5	34469.5	24526	53724	31337.5	29550.5	36396.5	NA	24982	NA	0.635281048	0.197034101	33502.7	30309.667	Down	-0.144499381759891	Kinesin-like protein KIF2C OS=Mus musculus GN=Kif2c PE=1 SV=1
MAP4	P27546	25	25	60633.64	58449.64	61952.12	64352	54753.08	57764.4	60247.84	65150.44	80208.76	50079.96	0.635564825	0.196840147	60028.096	62690.28	Up	0.0626038648484137	Microtubule-associated protein 4 OS=Mus musculus GN=Map4 PE=1 SV=3
YWHAG	P61982	42	42	152317.74	191151.79	165883.74	294795.38	174724.18	223198.9	208693.28	159662.33	173727.28	139453.15	0.635647903	0.196783382	195774.57	180946.99	Down	-0.113626256621773	14-3-3 protein gamma OS=Mus musculus GN=Ywhag PE=1 SV=2
MAPT	P10637-6	55	55	132748.81	121648.49	121717.64	114651.32	120776.13	108159.91	123362.62	137133.91	161116.75	108059.32	0.63601196	0.196534718	122308.48	127566.5	Up	0.0607251280585281	Isoform Tau-E of Microtubule-associated protein tau OS=Mus musculus GN=Mapt
AIMP1	P31230	6	6	79627	83542	87953.83	51737.17	74891.33	67553.83	70327.5	60280.83	95886.67	61448.83	0.636236221	0.19638161	75550.266	71099.532	Down	-0.0875967712969289	Aminoacyl tRNA synthase complex-interacting multifunctional protein 1 OS=Mus musculus GN=Aimp1 PE=1 SV=2

TTC9B	Q9D6E4	4	4	31175.75	36731.75	35347.25	25814.5	34439.25	35586.25	34859	55708.25	42704	12900	0.636557737	0.196162199	32701.7	36351.5	Up	0.152649260143762	Tetratricopeptide repeat protein 9B OS=Mus musculus GN=Ttc9b PE=2 SV=1
APBA1	B2RUJ5-2	1	1	4249	5937	NA	11427	4128	7440	5871	NA	2658	NA	0.63734738	0.195623795	6435.25	5323	Down	-0.273756629852245	Isoform 2 of Amyloid beta A4 precursor protein-binding family A member 1 OS=Mus musculus GN=Apba1
GNG4	P50153	4	4	25578.25	38559.5	34259.25	59791.75	39190.25	47492.25	52131.25	41433	46652	27058	0.638174041	0.195060865	39475.8	42953.3	Up	0.121800471020294	Guanine nucleotide-binding protein G(I)/G(S)/G(O) subunit gamma-4 OS=Mus musculus GN=Gng4 PE=2 SV=1
ACOX1	Q9R0H0	5	5	19777.2	24266	10104.6	18604.4	32407	26326.8	35306.2	10205.4	24895.4	NA	0.638479418	0.194853098	21031.84	24183.45	Up	0.201445001699995	Peroxisomal acyl-coenzyme A oxidase 1 OS=Mus musculus GN=Acox1 PE=1 SV=5
SBF1	Q6ZPE2	6	6	10264.17	18089.83	19441.83	32360.33	21234	27645.83	28715.17	9893.17	16774	3513.83	0.638678766	0.194717522	20278.032	17308.4	Down	-0.228445277192471	Myotubularin-related protein 5 OS=Mus musculus GN=Sbf1 PE=1 SV=2
RPS3	P62908	12	12	54201.58	72371	63198.08	59791.33	69729.08	67876.17	74726.75	54173.42	76083.75	18433.92	0.638895083	0.194570455	63858.214	58258.802	Down	-0.132396168828481	40S ribosomal protein S3 OS=Mus musculus GN=Rps3 PE=1 SV=1
CRYZ	P47199	2	2	57718.5	71160	65762	56235	61861.5	55319	52787	94202	81220	51866	0.640735459	0.193321241	62547.4	67078.8	Up	0.100906965167833	Quinone oxidoreductase OS=Mus musculus GN=Cryz PE=1 SV=1
DYNLL2	Q9D0M5	2	2	801.5	1197	NA	2900.5	905	1713	1132	NA	2426	NA	0.64095641	0.193171505	1451	1757	Up	0.276066671941632	Dynein light chain 2, cytoplasmic OS=Mus musculus GN=Dynll2 PE=1 SV=1
UBE2O	Q6ZPJ3	11	11	16281.1	25904.4	19824.6	34644.4	26523.1	34237.2	37329.4	25102	23693.9	15104.7	0.641233509	0.19298379	24635.52	27093.44	Up	0.137203657285006	Ubiquitin-conjugating enzyme E2 O OS=Mus musculus GN=Ube2o PE=1 SV=3
ABI1	Q8CBW3-5	16	16	97348.06	93933.69	68092	107909.44	96453.44	103362.81	98336.69	77635.44	100655.12	56863.81	0.641331965	0.192917114	92747.326	87370.774	Down	-0.0861549168940099	Isoform 5 of Abl interactor 1 OS=Mus musculus GN=Abi1
TTBK1	Q6PCN3	3	3	10982.33	11808	NA	11006.67	10441.33	11217	12099.33	NA	7608.67	NA	0.642020065	0.192451398	11059.583	10308.333	Down	-0.101485830283059	Tau-tubulin kinase 1 OS=Mus musculus GN=Ttbk1 PE=2 SV=3
GNL1	P36916	1	1	16735	28085	13246	119461	45581	89268	51346	NA	31317	NA	0.642262075	0.192287722	44621.6	57310.333	Up	0.361053042074248	Guanine nucleotide-binding protein-like 1 OS=Mus musculus GN=Gnl1 PE=2 SV=4
PCX	Q05920	11	11	41371.3	46130.9	40241.7	53321.4	44953.2	56734.2	46108.1	46305.7	50086.6	36500.2	0.642527139	0.192108524	45203.7	47146.96	Up	0.0607238854990008	Pyruvate carboxylase, mitochondrial OS=Mus musculus GN=Pc PE=1 SV=1
HCCS	P53702	3	3	18081	15570	19897.33	23144.67	12818.33	17559	19219	5872.67	17935.33	21117.33	0.64276591	0.191947164	17902.266	16340.666	Down	-0.131675424928469	Cytochrome c-type heme lyase OS=Mus musculus GN=Hccs PE=1 SV=2
PFKL	P12382	7	7	14218	25803.57	6392.71	69240.71	29001.29	41978.29	42478.71	5011.71	18109.57	3734.57	0.642833743	0.191901335	28931.256	22262.57	Down	-0.378008809127914	6-phosphofructokinase, liver type OS=Mus musculus GN=Pfkl PE=1 SV=4
GNG2	P63213	4	4	48197.5	85676.25	45304.5	302848.5	59710.75	134009.75	101246	27176.5	64609.75	NA	0.642917932	0.191844461	108347.5	81760.5	Down	-0.406189941101434	Guanine nucleotide-binding protein G(I)/G(S)/G(O) subunit gamma-2 OS=Mus musculus GN=Gng2 PE=2 SV=2
TBCD	Q8BYA0	3	3	1871	2516.33	971.33	11807.33	5779.67	8847.33	8094	1876.67	4496	NA	0.643053606	0.191752822	4589.132	5828.5	Up	0.344903340368374	Tubulin-specific chaperone D OS=Mus musculus GN=Tbcd PE=2 SV=1
SGIP1	Q8VD37-4	16	16	60884.2	60763.93	66944.73	46920.67	59939.67	50746.6	55258.93	55645.47	62780.87	61439	0.643142067	0.191693083	59090.64	57174.174	Down	-0.04756600651149	Isoform 4 of SH3-containing GRB2-like protein 3-interacting protein 1 OS=Mus musculus GN=Sgip1
SHTN1	Q8K2Q9	3	3	3764.67	5419	4483.67	9537.67	4666.67	9535.33	6325	1365.33	9351	NA	0.643521963	0.191436626	5574.336	6644.165	Up	0.253287936083229	Shootin-1 OS=Mus musculus GN=Kiaa1598 PE=1 SV=1
PPP2R2B	Q6ZWR4-3	1	1	18385	21927	7519	75236	18506	34422	18982	NA	11445	NA	0.644200856	0.190978703	28314.6	21616.333	Down	-0.389424322097103	Isoform 3 of Serine/threonine-protein phosphatase 2A 55 kDa regulatory subunit B beta isoform OS=Mus musculus GN=Ppp2r2b
GRHPR	Q91Z53	4	4	30553	48601	20350.5	58924	45907	46547.5	45724	NA	40821.5	NA	0.644373726	0.190862176	40867.1	44364.333	Up	0.118460418292204	Glyoxylate reductase/hydroxypyruvate reductase OS=Mus musculus GN=Grhpr PE=1 SV=1
MICU1	Q8VCX5-3	3	3	8570.67	10822	5388.67	16150.67	13766	15769.33	13357.67	NA	8069	NA	0.644380067	0.190857902	10939.602	12398.667	Up	0.180624732211397	Isoform 3 of Calcium uptake protein 1, mitochondrial OS=Mus musculus GN=Micu1
STAU2	Q8CJ67-3	2	2	13972.5	9365.5	4575	20705	15040	22369.5	18750.5	3819	15453.5	NA	0.644521857	0.19076235	12731.6	15098.125	Up	0.2459556594657	Isoform 3 of Double-stranded RNA-binding protein Staufen homolog 2 OS=Mus musculus GN=Stau2
SH3GLB2	Q8R3V5	17	17	57792.47	59550.24	57020.71	76207.35	68230.82	75786.94	68420	63262.47	59023.29	32147.18	0.645064257	0.190397022	63760.318	59727.976	Down	-0.0942519906365899	Endophilin-B2 OS=Mus musculus GN=Sh3glb2 PE=2 SV=2
RBFOX3	Q8BIF2-3	2	2	11974	10742	13861.5	20025	16585.5	18911	10207	5381.5	17089	NA	0.645735089	0.189945613	14637.6	12897.125	Down	-0.18262952790805	Isoform 3 of RNA binding protein fox-1 homolog 3 OS=Mus musculus GN=Rbfox3
PSMC2	P46471	13	13	18507.92	20337.69	16236	25486.85	21294.77	22847.31	22064.08	15457.31	24643.46	8857.15	0.645793903	0.189906059	20372.646	18773.862	Down	-0.117907910045158	26S protease regulatory subunit 7 OS=Mus musculus GN=Psmc2 PE=1 SV=5
WASF1	Q8R5H6	25	25	36924.91	41472	30127.39	40976.52	42013.22	41324.13	43441.91	30967.17	46806.83	13124.57	0.646222542	0.189617897	38302.808	35132.922	Down	-0.124626590510688	Wiskott-Aldrich syndrome protein family member 1 OS=Mus musculus GN=Wasf1 PE=1 SV=2
SPRED2	Q924S7	1	1	752	1658	NA	7045	4288	6232	6025	NA	1235	NA	0.646582998	0.189375719	3435.75	4497.3333	Up	0.388444754057806	Sprouty-related, EVH1 domain-containing protein 2 OS=Mus musculus GN=Spred2 PE=1 SV=1
CCDC50	Q810U5	1	1	17292	18726	3443	17071	18019	17922	17334	13065	17337	NA	0.646733015	0.189274968	14910.2	16414.5	Up	0.138671195292043	Coiled-coil domain-containing protein 50 OS=Mus musculus GN=Ccdc50 PE=1 SV=1
ABI1	Q8CBW3-3	17	17	91788.12	88408.18	64086.59	101822.35	91024.76	97617.94	92850.06	73068.65	95048.94	53518.88	0.647515706	0.188749693	87426	82420.894	Down	-0.0850522806404244	Isoform 3 of Abl interactor 1 OS=Mus musculus GN=Abi1
NDUFS6	P52503	18	18	179476.67	156173.06	170818.89	88085.83	167199.44	116799.22	135960.72	130954.11	165569.89	166459.17	0.647586702	0.188702078	152350.78	143148.62	Down	-0.089883084857971	NADH dehydrogenase [ubiquinone] iron-sulfur protein 6, mitochondrial OS=Mus musculus GN=Ndufs6 PE=1 SV=2
PCBP1	P60335	29	29	51977.5	55349.07	41653.07	67477.46	53229.71	59577.71	59985.89	48262.25	58965.32	54167.75	0.647735363	0.188602392	53937.362	56191.784	Up	0.0590742411807253	Poly(rC)-binding protein 1 OS=Mus musculus GN=Pcbp1 PE=1 SV=1
APP	P12023-3	14	14	60651.36	76203.71	34932.36	59677.64	62827.57	60723.14	76363.21	37194.21	71993.71	16694.36	0.647853209	0.188523386	58858.528	52593.726	Down	-0.162360754095506	Isoform APP751 of Amyloid beta A4 protein OS=Mus musculus GN=App
FNBP1	Q80TY0-5	3	3	47648.67	64565.33	57909.33	58191.33	50534.67	65031.33	59430.67	48405.67	64101	20483.67	0.648086995	0.188366694	55769.866	51490.468	Down	-0.115180421636188	Isoform 5 of Formin-binding protein 1 OS=Mus musculus GN=Fnbp1
NAV1	Q8CH77-2	11	11	16335.1	17720.4	12565.7	19451.9	19977.7	19376	22508.4	5346.5	24370.9	3153	0.648735435	0.18793238	17210.16	14950.96	Down	-0.20302238720074	Isoform 2 of Neuron navigator 1 OS=Mus musculus GN=Nav1
CRIP2	Q9DCT8	25	25	79732.68	64876.2	67403.8	58974.72	76485.84	65753.6	75751.8	63585.12	88734.6	67752.48	0.64932127	0.187540371	69494.648	72315.52	Up	0.0574034288346077	Cysteine-rich protein 2 OS=Mus musculus GN=Crip2 PE=1 SV=1
BCL7A	Q9CXE2	1	1	17314	13614	27097	6320	9827	10767	9324	32805	18863	NA	0.649473449	0.187438598	14834.4	17939.75	Up	0.27421320968073	B-cell CLL/lymphoma 7 protein family member A OS=Mus musculus GN=Bcl7a PE=2 SV=1
MRPS17	Q9CQE3	1	1	75033	59452	79443	28590	60705	47984	54099	89122	76262	NA	0.649732955	0.187265105	60644.6	66866.75	Up	0.140909809464123	28S ribosomal protein S17, mitochondrial OS=Mus musculus GN=Mrps17 PE=2 SV=1
KCNAB2	P62482	3	3	17923.67	13009	30949.67	23369.67	28874.33	29773	27538.67	17199.67	24106	24878.67	0.650947862	0.186453795	22825.268	24699.202	Up	0.113832631379063	Voltage-gated potassium channel subunit beta-2 OS=Mus musculus GN=Kcnab2 PE=2 SV=1
AUH	Q9JLZ3-3	2	2	162394.5	131175.5	70981.5	169848	153625.5	171495	151237.5	95181.5	151176	52177	0.651184261	0.186296105	137605	124253.4	Down	-0.147247563177648	Isoform 3 of Methylglutaconyl-CoA hydratase, mitochondrial OS=Mus musculus GN=Auh
NARS	Q8BP47	1	1	74880	93427	NA	292844	67501	147324	160114	60543	41862	NA	0.651356042	0.186181554	132163	102460.75	Down	-0.367246983033631	Asparagine--tRNA ligase, cytoplasmic OS=Mus musculus GN=Nars PE=1 SV=2
SNCA	O55042-2	39	39	120569.82	106685.23	100131.23	165937.95	104825	133340.49	114674.13	113512.62	132251.41	63177.72	0.651559237	0.186046094	119629.85	111391.27	Down	-0.102941145541034	Isoform 2 of Alpha-synuclein OS=Mus musculus GN=Snca
VAMP1	Q62442	1	1	11886	15905	NA	50372	29426	40253	36763	NA	19276	NA	0.651694659	0.185955838	26897.25	32097.333	Up	0.254994764482502	Vesicle-associated membrane protein 1 OS=Mus musculus GN=Vamp1 PE=1 SV=1
OMG	Q63912	4	4	62959.75	79210	39192.5	136644.25	78608.75	108477.25	111652.5	76862.75	83765.25	60224.25	0.651897681	0.185820564	79323.05	88196.4	Up	0.152979618289089	Oligodendrocyte-myelin glycoprotein OS=Mus musculus GN=Omg PE=1 SV=1
RALY	Q64012	1	1	200024	133336	108420	60401	156665	87432	114469	58120	131101	191769	0.652158897	0.185646576	131769.2	116578.2	Down	-0.176715159972387	RNA-binding protein Raly OS=Mus musculus GN=Raly PE=1 SV=3
PRKRA	Q9WTX2	1	1	16781	22214	11248	44000	13871	25946	32390	NA	17024	NA	0.652397078	0.185487993	21622.8	25120	Up	0.216283110233904	Interferon-inducible double-stranded RNA-dependent protein kinase activator A OS=Mus musculus GN=Prkra PE=1 SV=1
PSAT1	Q99K85	3	3	126839.33	143779	140983	138614.67	156623.33	163976.33	187187.67	143278.67	136599.33	109159	0.653151181	0.184986283	141367.87	148040.2	Up	0.06653476804365	Phosphoserine aminotransferase OS=Mus musculus GN=Psat1 PE=1 SV=1
ARHGEF6	Q8K4I3	1	1	66607	91160	42121	226707	94487	134623	94350	41790	75570	NA	0.653446364	0.184790054	104216.4	86583.25	Down	-0.26742246513004	Rho guanine nucleotide exchange factor 6 OS=Mus musculus GN=Arhgef6 PE=1 SV=1
RPL10	Q6ZWV3	24	24	62957	67014	60378.41	47261.86	63043.23	52342.91	66811.59	51752.09	70380.41	45712.5	0.65346689	0.184776412	60130.9	57399.9	Down	-0.0670583285189956	60S ribosomal protein L10 OS=Mus musculus GN=Rpl10 PE=1 SV=3
SEPT3	Q9Z1S5-2	40	40	52591.08	53411.21	50767.49	64915.72	57014.05	59256.15	57482.95	38263.62	58714.95	54056.49	0.653880818	0.184501403	55739.91	53554.832	Down	-0.0576939245877405	Isoform 2 of Neuronal-specific septin-3 OS=Mus musculus GN=Sept3
PSMA1	Q9R1P4	29	29	89051.21	83823.45	73518.34	62756.34	76947.21	76245.45	87963	74289.59	107384.45	61274.76	0.653934886	0.184465494	77219.31	81431.45	Up	0.0766244287331728	Proteasome subunit alpha type-1 OS=Mus musculus GN=Psma1 PE=1 SV=1
PLCB1	Q9Z1B3-2	14	14	26653.42	32100.75	21158.33	43847.33	31184.92	38621.25	36976	20878.67	27662.5	17726.08	0.653982382	0.184433951	30988.95	28372.9	Down	-0.127240256542357	Isoform B of 1-phosphatidylinositol 4,5-bisphosphate phosphodiesterase beta-1 OS=Mus musculus GN=Plcb1
SH3GLB2	Q8R3V5-3	16	16	60806.31	62791	60584.5	80165.12	71945.44	79845.94	72162.56	67216.38	62386.69	34156.38	0.654047987	0.184390386	67258.474	63153.59	Down	-0.0908512993940448	Isoform 3 of Endophilin-B2 OS=Mus musculus GN=Sh3glb2
RPS6	P62754	25	25	115956.28	128131.28	144244.04	115858.08	116732.8	106305.92	122777.64	113017.2	143400.72	115807.68	0.654123203	0.184340445	124184.5	120261.83	Down	-0.0463062283651702	40S ribosomal protein S6 OS=Mus musculus GN=Rps6 PE=1 SV=1
MAGI3	Q9EQJ9	1	1	9461	10360	6300	18208	12324	14156	10985	NA	11954	NA	0.65451797	0.184078425	11330.6	12365	Up	0.126037980148839	Membrane-associated guanylate kinase, WW and PDZ domain-containing protein 3 OS=Mus musculus GN=Magi3 PE=1 SV=2
NDUFC2	Q9CQ54	1	1	26736	29213	47031	29076	32225	30499	29904	49698	32524	NA	0.655042127	0.183730769	32856.2	35656.25	Up	0.117989347070995	NADH dehydrogenase [ubiquinone] 1 subunit C2 OS=Mus musculus GN=Ndufc2 PE=1 SV=1
EEF1A2	P62631	41	41	102186.34	99937.88	111564.27	137654.51	98285.46	111110.85	106838.54	94627.93	101260.66	116487.95	0.655605403	0.183357476	109925.69	106065.19	Down	-0.0515774194667304	Elongation factor 1-alpha 2 OS=Mus musculus GN=Eef1a2 PE=1 SV=1
SETD7	Q8VHL1	3	3	42592.67	53955.33	74613.33	45009.33	60680.33	57591	66453.33	34981.67	45648.33	NA	0.655843118	0.183200034	55370.198	51168.583	Down	-0.113851413279168	Histone-lysine N-methyltransferase SETD7 OS=Mus musculus GN=Setd7 PE=2 SV=2
GGT7	Q99JP7	4	4	12068.5	13432.75	5092.25	25415.75	12588.5	15225	16986	8525.5	16020.25	1698.75	0.656108902	0.18302407	13719.55	11691.1	Down	-0.230822484512522	Gamma-glutamyltransferase 7 OS=Mus musculus GN=Ggt7 PE=2 SV=2
PCMT1	P23506	16	16	120697.06	101313.81	102848.69	114513.88	111000.12	110746.44	114106.12	131289.88	123888.69	89124.06	0.656506418	0.182761024	110074.71	113831.04	Up	0.0484109174178805	Protein-L-isoaspartate(D-aspartate) O-methyltransferase OS=Mus musculus GN=Pcmt1 PE=1 SV=3
EDC4	Q3UJB9	1	1	4104	12247	NA	42952	18634	28071	38145	NA	9180	NA	0.656543065	0.182736781	19484.25	25132	Up	0.367217085745405	Enhancer of mRNA-decapping protein 4 OS=Mus musculus GN=Edc4 PE=1 SV=2
ELAVL3	Q60900-2	1	1	113621	141996	87317	113943	103439	105812	140819	170084	130647	60753	0.656843537	0.182538069	112063.2	121623	Up	0.118103486271274	Isoform HuC-S of ELAV-like protein 3 OS=Mus musculus GN=Elavl3
PPP1R9B	Q6R891-2	22	22	30852.18	33397.09	15332.55	46040.27	37302.95	38520.55	42773.59	20578.23	35156	6564.91	0.656857918	0.182528561	32585.008	28718.656	Down	-0.182220114877153	Isoform 2 of Neurabin-2 OS=Mus musculus GN=Ppp1r9b
EIF4G2	Q62448	1	1	2092	2732	NA	30756	1963	7960	7175	NA	2332	NA	0.657249358	0.18226983	9385.75	5822.3333	Down	-0.688874595780902	Eukaryotic translation initiation factor 4 gamma 2 OS=Mus musculus GN=Eif4g2 PE=1 SV=2
NPM1	Q61937	15	15	40311.6	42341.13	51451.27	26252.53	33185.13	26548.6	32592.67	59490.07	44958	46504.87	0.657257245	0.182264618	38708.332	42018.842	Up	0.118392262152756	Nucleophosmin OS=Mus musculus GN=Npm1 PE=1 SV=1
NOL4L	Q6DIB4	2	2	29085	13387	17519	6325	18079	12298	14233	19781	13604	NA	0.657527075	0.182086359	16879	14979	Down	-0.17228812129588	Uncharacterized protein C20orf112 homolog OS=Mus musculus PE=2 SV=1
PURA	P42669	32	32	49268.58	55342.85	53063.08	56592.65	54729.19	53896.46	53265.08	76249.81	58983	40620.19	0.657697321	0.181973927	53799.27	56602.908	Up	0.0732895770241973	Transcriptional activator protein Pur-alpha OS=Mus musculus GN=Pura PE=1 SV=1
PIP4K2B	Q80XI4	5	5	33903.4	39841	41877.4	31445.8	33181.2	33778.2	40607.2	68952.4	43562.8	14643.2	0.657952391	0.181805531	36049.76	40308.76	Up	0.161103748095104	Phosphatidylinositol 5-phosphate 4-kinase type-2 beta OS=Mus musculus GN=Pip4k2b PE=1 SV=1
ACTR3	Q99JY9	9	9	41482.78	47702.89	55291.11	71189.67	57078.44	62659.78	65362.67	29705.22	45393.78	50852	0.658026493	0.181756621	54548.978	50794.69	Down	-0.102874479527665	Actin-related protein 3 OS=Mus musculus GN=Actr3 PE=1 SV=3
PPP1R18	Q8BQ30	1	1	NA	2085	NA	3890	2394	3381	2711	NA	3163	NA	0.658092483	0.18171307	2789.6667	3085	Up	0.145177742616557	Phostensin OS=Mus musculus GN=Ppp1r18 PE=1 SV=1
SRSF1	Q6PDM2-3	2	2	5812	4536	1788.5	11263	4186	7319	4752.5	1881	4547	NA	0.658258304	0.181603654	5517.1	4624.875	Down	-0.254495756881531	Isoform 3 of Serine/arginine-rich splicing factor 1 OS=Mus musculus GN=Srsf1
HK1	P17710-4	42	42	59084.76	90549.33	69542.21	140381.52	94768.52	124092.14	137236.93	65234.26	85967.12	86206.88	0.658582757	0.181389644	90865.268	99747.466	Up	0.13455124192215	Isoform HK1-SC of Hexokinase-1 OS=Mus musculus GN=Hk1
STX12	Q9ER00	8	8	44975.62	56765.5	66530.5	66854.25	60553.38	72062.62	82383.62	43144	84460	38341.75	0.658829784	0.181226775	59135.85	64078.398	Up	0.115805077491228	Syntaxin-12 OS=Mus musculus GN=Stx12 PE=1 SV=1
RGS6	Q9Z2H2	2	2	5639	5569	NA	4258	4722	5726	5540	NA	4569	NA	0.658898987	0.18118116	5047	5278.3333	Up	0.0646563748959693	Regulator of G-protein signaling 6 OS=Mus musculus GN=Rgs6 PE=1 SV=2
ICA1L	Q3TY65	1	1	4934	5542	9683	9643	6976	9779	5788	NA	8695	NA	0.659121671	0.181034409	7355.6	8087.3333	Up	0.136821048192744	Islet cell autoantigen 1-like protein OS=Mus musculus GN=Ica1l PE=2 SV=1
NDUFV3	Q8BK30	1	1	28368	11418	30998	29149	19845	19753	18937	25550	24098	NA	0.659333078	0.180895135	23955.6	22084.5	Down	-0.117328778467133	NADH dehydrogenase [ubiquinone] flavoprotein 3, mitochondrial OS=Mus musculus GN=Ndufv3 PE=2 SV=1
ARHGEF7	Q9ES28-7	9	9	31612.86	40963.43	31283.43	59542.71	45201.14	50473.43	47386.86	19908.29	40995.29	32503	0.659865027	0.180544889	41720.714	38253.374	Down	-0.125176846968232	Isoform G of Rho guanine nucleotide exchange factor 7 OS=Mus musculus GN=Arhgef7
CLVS2	Q8BG92	1	1	5830	6288	NA	6930	4777	6627	5570	NA	4633	NA	0.660249023	0.180292233	5956.25	5610	Down	-0.0864035381885114	Clavesin-2 OS=Mus musculus GN=Clvs2 PE=2 SV=1
FBXL17	Q9QZN1	1	1	1819	8723	4694	12859	9375	9975	11793	NA	4719	NA	0.660678962	0.180009522	7494	8829	Up	0.236514065332438	F-box/LRR-repeat protein 17 OS=Mus musculus GN=Fbxl17 PE=2 SV=3
ZADH2	Q8BGC4	3	3	22344	23795.33	26030	28010	26854	27863	28238.33	20094	29045	26188.67	0.660839067	0.179904291	25406.666	26285.8	Up	0.0490765739718779	Zinc-binding alcohol dehydrogenase domain-containing protein 2 OS=Mus musculus GN=Zadh2 PE=1 SV=1
ATP1A4	Q9WV27	18	18	76801.41	191768	97311.88	204980.82	189278	197673.59	294266.47	121426.59	118497.35	126947.71	0.661239958	0.179640911	152028.02	171762.34	Up	0.176076500182772	Sodium/potassium-transporting ATPase subunit alpha-4 OS=Mus musculus GN=Atp1a4 PE=1 SV=3
PPP3CA	P63328-2	15	15	20912.69	27800.31	28173.38	64075.46	28874.92	41812.92	39481.38	27144.62	33311.62	5085.85	0.66127598	0.179617252	33967.352	29367.278	Down	-0.209939203060406	Isoform 2 of Serine/threonine-protein phosphatase 2B catalytic subunit alpha isoform OS=Mus musculus GN=Ppp3ca
BAG2	Q91YN9	2	2	10070	12942.5	11193.5	10392.5	10940.5	10578.5	12570	8719.5	11221.5	15201	0.66132542	0.179584783	11107.8	11658.1	Up	0.0697595756028475	BAG family molecular chaperone regulator 2 OS=Mus musculus GN=Bag2 PE=1 SV=1
KAZN	Q69ZS8-3	8	8	11142.29	12761.57	6629.71	13742.14	12025.71	11498.86	10954.14	10952.43	12727.14	6462.14	0.661553511	0.179435021	11260.284	10518.942	Down	-0.0982536096496881	Isoform 3 of Kazrin OS=Mus musculus GN=Kazn
TOMM20	Q9DCC8	4	4	8515.25	8691.25	9304.5	13149.5	9805	12078	10628.5	4032.75	11325.5	7473.5	0.66214621	0.179046102	9893.1	9107.65	Down	-0.119343809020685	Mitochondrial import receptor subunit TOM20 homolog OS=Mus musculus GN=Tomm20 PE=1 SV=1
ARHGEF7	Q9ES28-5	7	7	35063.67	45576.17	34423.5	67634.17	51268.5	57109.33	52815.67	22300.17	45937.67	35993.17	0.662387537	0.178887847	46793.202	42831.202	Down	-0.127636788794045	Isoform E of Rho guanine nucleotide exchange factor 7 OS=Mus musculus GN=Arhgef7
ARVCF	P98203-3	6	6	16515.33	21416.67	15920.33	15520	23949.67	25112.67	27565.33	4912.33	28050.67	NA	0.662928721	0.178533165	18664.4	21410.25	Up	0.19801251025291	Isoform 3 of Armadillo repeat protein deleted in velo-cardio-facial syndrome homolog OS=Mus musculus GN=Arvcf
DBN1	Q9QXS6-2	37	37	42634.26	46599.2	49450.57	43484.74	43492.43	40034.77	49978.43	43710.49	50771.86	46549.71	0.663189485	0.178362368	45132.24	46209.052	Up	0.0340171081933142	Isoform A2 of Drebrin OS=Mus musculus GN=Dbn1
DOCK7	Q8R1A4	1	1	7070	30523	13726	45675	31522	38523	57354	5051	25802	NA	0.66331452	0.178280496	25703.2	31682.5	Up	0.301738196549326	Dedicator of cytokinesis protein 7 OS=Mus musculus GN=Dock7 PE=1 SV=3
DPH2	Q9CR25	1	1	5021	5562	11003	6028	4958	7793	5384	4790	8634	9229	0.664013038	0.177823393	6514.4	7166	Up	0.137535736111579	Diphthamide biosynthesis protein 2 OS=Mus musculus GN=Dph2 PE=1 SV=1
CHORDC1	Q9D1P4	1	1	59763	53220	22821	76856	82040	75793	78237	NA	43843	NA	0.664146766	0.177735937	58940	65957.667	Up	0.162293302110142	Cysteine and histidine-rich domain-containing protein 1 OS=Mus musculus GN=Chordc1 PE=1 SV=1
PPTC7	Q6NVE9-2	4	4	20490.25	23753	10698	23803.75	22720	23129.5	26825	19391.5	17347.5	4500.75	0.664328222	0.177617298	20293	18238.85	Down	-0.153967393325717	Isoform 2 of Protein phosphatase PTC7 homolog OS=Mus musculus GN=Pptc7
NDUFA7	Q9Z1P6	22	22	120704.23	102926.41	112845.86	60428.95	106222.73	83167.95	91922.18	116445.32	130318.95	111724.86	0.664803611	0.177306631	100625.64	106715.85	Up	0.0847765940313656	NADH dehydrogenase [ubiquinone] 1 alpha subcomplex subunit 7 OS=Mus musculus GN=Ndufa7 PE=1 SV=3
LMNB2	P21619-2	4	4	42606	25966.25	21907.75	11870.25	33841.25	28465	24943	9817	32696.5	NA	0.66550322	0.176849839	27238.3	23980.375	Down	-0.183782445019743	Isoform B3 of Lamin-B2 OS=Mus musculus GN=Lmnb2
CNP	P16330	33	33	51318.03	57887.39	56740.12	84162.73	62820.79	74458.73	54471.3	71224.24	42517.76	51588.09	0.665524064	0.176836236	62585.812	58852.024	Down	-0.0887436076170222	2';,3';-cyclic-nucleotide 3';-phosphodiesterase OS=Mus musculus GN=Cnp PE=1 SV=3
CELSR2	Q9R0M0	5	5	8309.75	9399.5	3207.5	16231.5	10380	13829	12080.75	5951	10993.25	NA	0.66663503	0.176111869	9505.65	10713.5	Up	0.172572684634275	Cadherin EGF LAG seven-pass G-type receptor 2 OS=Mus musculus GN=Celsr2 PE=1 SV=2
EPHA5	Q60629	2	2	8718	1556	NA	10149.5	8397	2432	15437	NA	9771	NA	0.666733944	0.176047434	7205.125	9213.3333	Up	0.354699750302099	Ephrin type-A receptor 5 OS=Mus musculus GN=Epha5 PE=1 SV=2
ADCYAP1	O70176	1	1	5584	6460	NA	4249	4446	5361	5328	NA	3893	NA	0.666738344	0.176044568	5184.75	4860.6667	Down	-0.0931202255530721	Pituitary adenylate cyclase-activating polypeptide OS=Mus musculus GN=Adcyap1 PE=2 SV=1
SNPH	Q80U23-3	11	11	30772.4	30109.7	46679.3	24658.4	38179	29597.8	31498.6	29934.7	44425.7	46949.7	0.667128435	0.175790548	34079.76	36481.3	Up	0.0982419647127322	Isoform 3 of Syntaphilin OS=Mus musculus GN=Snph
SNAP91	Q61548-3	13	13	104860.77	129992.46	83954.08	243373.38	163767.85	169591.92	184425.15	121647.62	110879.38	60081.62	0.668846154	0.174673766	145189.71	129325.14	Down	-0.166936458248638	Isoform 3 of Clathrin coat assembly protein AP180 OS=Mus musculus GN=Snap91
YJEFN3	F6W8I0	1	1	7299	8413	11981	7034	8548	8485	7747	6120	7540	10789	0.669168128	0.174464752	8655	8136.2	Down	-0.0891786763525251	YjeF N-terminal domain-containing protein 3 OS=Mus musculus GN=Yjefn3 PE=2 SV=2
GRM2	Q14BI2	1	1	14480	27938	NA	53686	23729	33912	29188	NA	12305	NA	0.669698358	0.174120766	29958.25	25135	Down	-0.253255662731999	Metabotropic glutamate receptor 2 OS=Mus musculus GN=Grm2 PE=1 SV=2

TMSB4X	P20065	1	1	95211	96291	155594	57064	74947	58987	61275	120655	77916	113583	0.670019189	0.173912759	95821.4	86483.2	Down	-0.147927986336586	Thymosin beta-4 OS=Mus musculus GN=Tmsb4x PE=1 SV=1
KYAT3	Q71RI9	2	2	992	NA	NA	5160	1271	3693	2639	NA	NA	NA	0.670240012	0.173769649	2474.3333	3166	Up	0.355621387354402	Kynurenine--oxoglutarate transaminase 3 OS=Mus musculus GN=Ccbl2 PE=1 SV=1
MTHFD1L	Q3V3R1	1	1	9456	13933	9537	33985	11147	22380	15149	NA	16283	NA	0.670450563	0.17363324	15611.6	17937.333	Up	0.200347023224998	Monofunctional C1-tetrahydrofolate synthase, mitochondrial OS=Mus musculus GN=Mthfd1l PE=1 SV=2
RPN1	Q91YQ5	7	7	17096	29259.14	6513.86	43688.57	32732.43	39358.43	48394.43	4043	32360.71	NA	0.670608502	0.173530945	25858	31039.143	Up	0.263478008103846	Dolichyl-diphosphooligosaccharide--protein glycosyltransferase subunit 1 OS=Mus musculus GN=Rpn1 PE=1 SV=1
CPNE6	Q9Z140	20	20	14159.45	19738.1	14666.75	33314.8	18985.95	22103.8	22849.25	26915.6	21224.9	16395.3	0.671047971	0.173246433	20173.01	21897.77	Up	0.118357594303873	Copine-6 OS=Mus musculus GN=Cpne6 PE=2 SV=1
GFER	P56213	4	4	33489.75	36313.25	39398.25	45764.5	53212.5	45827.75	40112.5	NA	44245.75	NA	0.67144257	0.172991128	41635.65	43395.333	Up	0.0597205588845254	FAD-linked sulfhydryl oxidase ALR OS=Mus musculus GN=Gfer PE=2 SV=2
SERPINE2	Q07235	1	1	45793	61162	40239	65647	41741	52171	62411	45568	54656	NA	0.671531675	0.172933498	50916.4	53701.5	Up	0.0768319680512421	Glia-derived nexin OS=Mus musculus GN=Serpine2 PE=2 SV=2
ARHGAP17	Q3UIA2-3	1	1	955	827	NA	1617	804	2489	451	NA	1118	NA	0.671667608	0.172845596	1050.75	1352.6667	Up	0.364386907883529	Isoform 3 of Rho GTPase-activating protein 17 OS=Mus musculus GN=Arhgap17
CRTC1	Q68ED7	6	6	67171.67	65201.5	56109.5	50864.83	64875.83	66373.67	65327.33	58571.33	92391.83	41326.33	0.671785209	0.172769563	60844.666	64798.098	Up	0.0908206733370206	CREB-regulated transcription coactivator 1 OS=Mus musculus GN=Crtc1 PE=2 SV=1
POLR2F	P61219	1	1	4425	1093	3595	884	2377	2232	1018	NA	2230	7201	0.671946554	0.172665269	2474.8	3170.25	Up	0.357284674025674	DNA-directed RNA polymerases I, II, and III subunit RPABC2 OS=Mus musculus GN=Polr2f PE=2 SV=1
PTPRD	Q64487-3	2	2	5105.5	7205	1173.5	15934	10095	11968.5	11363	5818.5	7588.5	NA	0.671992417	0.172635628	7902.6	9184.625	Up	0.216893432395155	Isoform B of Receptor-type tyrosine-protein phosphatase delta OS=Mus musculus GN=Ptprd
PPTC7	Q6NVE9	6	6	19200.5	21135.67	9800.17	19976.17	21597.17	22233.83	22358	14399.17	18213	6389.67	0.672110546	0.17255929	18341.936	16718.734	Down	-0.133680318384491	Protein phosphatase PTC7 homolog OS=Mus musculus GN=Pptc7 PE=2 SV=1
GATC	Q8CBY0	2	2	58772	62794	57707.5	31309	58299	47198.5	53303.5	37963	51719.5	63232	0.67238329	0.172383088	53776.3	50683.3	Down	-0.085460034389089	Glutamyl-tRNA(Gln) amidotransferase subunit C, mitochondrial OS=Mus musculus GN=Gatc PE=2 SV=1
RAB21	P35282	1	1	9998	31676	NA	51815	29652	28106	34991	NA	15368	NA	0.672430049	0.172352887	30785.25	26155	Down	-0.235152514382332	Ras-related protein Rab-21 OS=Mus musculus GN=Rab21 PE=1 SV=4
PSMA2	P49722	5	5	66008.8	62494.8	70521.8	99014.4	46047.2	73685.8	59676.6	89564.4	64594.8	27136	0.6728684	0.172069867	68817.4	62931.52	Down	-0.128990597349594	Proteasome subunit alpha type-2 OS=Mus musculus GN=Psma2 PE=1 SV=3
ACTN1	Q7TPR4	34	34	11692.83	19423.2	11634.77	40915	19951.97	26469.07	26035.5	9661.27	18352.53	8646.47	0.673522485	0.171647901	20723.554	17832.968	Down	-0.216724604744124	Alpha-actinin-1 OS=Mus musculus GN=Actn1 PE=1 SV=1
OLFM1	O88998	6	6	25999	23659.83	19027.67	23650	21858.5	24497.67	21796.5	26207.5	25155.17	9007.83	0.673574313	0.171614483	22839	21332.934	Down	-0.0984170853450097	Noelin OS=Mus musculus GN=Olfm1 PE=1 SV=1
RIMBP2	Q80U40	11	11	12890.6	13438.2	11747.4	13994.9	13732.8	15464.1	15013.3	4542.9	19038.1	5007.8	0.674211611	0.171203773	13160.78	11813.24	Down	-0.155840290960774	RIMS-binding protein 2 OS=Mus musculus GN=Rimbp2 PE=1 SV=3
FYN	P39688	2	2	21787.5	39429	38976.5	25225	31742	29723	33857	34099	26355.5	43004	0.674218192	0.171199534	31432	33407.7	Up	0.0879465888173889	Tyrosine-protein kinase Fyn OS=Mus musculus GN=Fyn PE=1 SV=4
HNRNPA0	Q9CX86	4	4	50555.25	32429.5	50186	16064.25	32197	22082.25	32012.75	35471.75	40833	75447.25	0.674633408	0.170932156	36286.4	41169.4	Up	0.18214348997631	Heterogeneous nuclear ribonucleoprotein A0 OS=Mus musculus GN=Hnrnpa0 PE=1 SV=1
BRSK1	Q5RJI5	9	9	39675.89	51888.89	48406.22	41454.33	50071.89	53989.11	63076	23218.56	70701.67	40120.56	0.675675306	0.170261953	46299.444	50221.18	Up	0.117301058083847	Serine/threonine-protein kinase BRSK1 OS=Mus musculus GN=Brsk1 PE=1 SV=1
SH3GL1	Q62419	17	17	109599.5	115604.94	128723.81	77694.25	122718.75	98431.06	101321.69	102192.25	128103.25	154805.75	0.675882324	0.170128911	110868.25	116970.8	Up	0.0773021561576592	Endophilin-A2 OS=Mus musculus GN=Sh3gl1 PE=1 SV=1
RTN3	Q9ES97-4	1	1	11600	15827	17234	32320	12079	23217	18770	NA	17018	NA	0.675961247	0.170078201	17812	19668.333	Up	0.143025193971157	Isoform 4 of Reticulon-3 OS=Mus musculus GN=Rtn3
RPL6	P47911	14	14	133609.21	156749.86	187644.64	139852.64	131113.93	145841.64	153296.14	184846.14	193688.79	110741.79	0.676549428	0.169700469	149794.06	157682.9	Up	0.0740458376965894	60S ribosomal protein L6 OS=Mus musculus GN=Rpl6 PE=1 SV=3
DNM1L	Q8K1M6-4	14	14	18551.77	24580.77	23215.54	40408.23	26319.92	31342.92	32036.46	20203	22475.77	37615	0.676775866	0.169555137	26615.246	28734.63	Up	0.11053757160419	Isoform 4 of Dynamin-1-like protein OS=Mus musculus GN=Dnm1l
NRCAM	Q810U4-3	27	27	51125.19	58959.11	53515.59	57358.78	57451.33	67988.33	65163.89	36919.04	62816.78	26344.19	0.676893456	0.169479685	55682	51846.446	Down	-0.102965933460073	Isoform 3 of Neuronal cell adhesion molecule OS=Mus musculus GN=Nrcam
MRPS16	Q9CPX7	1	1	49366	47589	55609	26162	41787	31393	40083	51297	37353	83320	0.677307868	0.169213879	44102.6	48689.2	Up	0.142738086116364	28S ribosomal protein S16, mitochondrial OS=Mus musculus GN=Mrps16 PE=2 SV=1
STX8	O88983	3	3	2379	2050	1503.5	5441	3440.5	4880.5	4615	1966.5	2196.5	NA	0.677786408	0.168907145	2962.8	3414.625	Up	0.204765905645502	Syntaxin-8 OS=Mus musculus GN=Stx8 PE=1 SV=1
SUMO3	Q9Z172-2	5	5	8012.25	8956.25	7420.5	11195.25	7325.5	10183.5	8890.25	19675.25	9695.75	1175.75	0.677917771	0.168822981	8581.95	9924.1	Up	0.20963077688467	Isoform 2 of Small ubiquitin-related modifier 3 OS=Mus musculus GN=Sumo3
APOA1BP	Q8K4Z3	4	4	20595.75	25065	46891.75	13391	18953.75	24518.5	24223	42728.75	28985	19628.75	0.677941669	0.168807672	24979.45	28016.8	Up	0.165550472719983	NAD(P)H-hydrate epimerase OS=Mus musculus GN=Apoa1bp PE=1 SV=1
ANK2	Q8C8R3	129	129	27813.39	38022.64	30864.71	61120.19	44767.01	51330.42	60105.98	27367.89	46946.4	34773.8	0.677991186	0.168775952	40517.588	44104.898	Up	0.122390587418607	Ankyrin-2 OS=Mus musculus GN=Ank2 PE=1 SV=2
FAM110B	Q8C739	1	1	7805	10100	4220	7186	7555	9510	7687	8829	5652	NA	0.678300606	0.168577795	7373.2	7919.5	Up	0.103118457212386	Protein FAM110B OS=Mus musculus GN=Fam110b PE=2 SV=1
CACNB1	Q8R3Z5-4	4	4	51862.5	55338.25	47309.5	26403.25	53275.75	50126.75	54597.25	37646.5	57592	47837.5	0.678552896	0.168416291	46837.85	49560	Up	0.0815013342168357	Isoform 4 of Voltage-dependent L-type calcium channel subunit beta-1 OS=Mus musculus GN=Cacnb1
CLIC1	Q9Z1Q5	1	1	NA	731	NA	3399	744	1228	997	NA	1370	NA	0.679196907	0.168004301	1624.6667	1198.3333	Down	-0.439114480396681	Chloride intracellular channel protein 1 OS=Mus musculus GN=Clic1 PE=1 SV=3
TOMM22	Q9CPQ3	2	2	NA	NA	NA	2429	407	744	978	NA	NA	NA	0.679283957	0.167948642	1418	861	Down	-0.719772389842672	Mitochondrial import receptor subunit TOM22 homolog OS=Mus musculus GN=Tomm22 PE=2 SV=3
ETFDH	Q921G7	4	4	11310.25	15191.5	2878	25008	13869.5	23160	14073	8238	17272	NA	0.679920829	0.167541654	13651.45	15685.75	Up	0.200400315668667	Electron transfer flavoprotein-ubiquinone oxidoreductase, mitochondrial OS=Mus musculus GN=Etfdh PE=1 SV=1
RABGAP1L	A6H6A9-3	3	3	26602.33	32551.33	26473.67	77687.67	30081.67	49652	45021	24187.67	40388.67	7353.33	0.68070219	0.167042852	38679.334	33320.534	Down	-0.215151432247016	Isoform 3 of Rab GTPase-activating protein 1-like OS=Mus musculus GN=Rabgap1l
HDDC2	Q3SXD3	1	1	53592	82929	47793	72578	69652	76894	61186	NA	68032	NA	0.681361875	0.16662217	65308.8	68704	Up	0.0731166962530453	HD domain-containing protein 2 OS=Mus musculus GN=Hddc2 PE=2 SV=1
ITPKA	Q8R071	16	16	8312.38	7119.46	4999.38	7978.46	7962.92	9070.69	9050.15	2760.69	10086.31	8518.62	0.68164765	0.166440058	7274.52	7897.292	Up	0.118505977811195	Inositol-trisphosphate 3-kinase A OS=Mus musculus GN=Itpka PE=2 SV=1
MAP4K3	Q99JP0	1	1	NA	1526	NA	2373	2375	2670	2015	NA	2058	NA	0.682517712	0.165886074	2091.3333	2247.6667	Up	0.104005068484476	Mitogen-activated protein kinase kinase kinase kinase 3 OS=Mus musculus GN=Map4k3 PE=1 SV=4
MAP4	P27546-3	24	24	61055	58248.04	61636.38	63892.25	54873.83	57996	60396.71	65766.42	80451.5	47346.29	0.683200929	0.165451552	59941.1	62391.384	Up	0.0578012529478921	Isoform 3 of Microtubule-associated protein 4 OS=Mus musculus GN=Map4
DLD	O08749	42	42	150014.73	150664.66	181062.39	147211.02	148081.9	147182.27	153173.41	154495.39	147485	199770.46	0.685181961	0.164194079	155406.94	160421.31	Up	0.0458148313154731	Dihydrolipoyl dehydrogenase, mitochondrial OS=Mus musculus GN=Dld PE=1 SV=2
TXNRD2	Q9JLT4-3	5	5	42532.4	47980.8	53157.6	58330.2	38161.6	61641.6	50966.4	65700.8	59088.2	21425.8	0.68524501	0.164154118	48032.52	51764.56	Up	0.107953210833847	Isoform 3 of Thioredoxin reductase 2, mitochondrial OS=Mus musculus GN=Txnrd2
TMEM65	Q4VAE3	1	1	8638	25071	NA	59628	20345	49094	44209	NA	12475	NA	0.685581995	0.163940596	28420.5	35259.333	Up	0.311073261082289	Transmembrane protein 65 OS=Mus musculus GN=Tmem65 PE=2 SV=1
EIF3C	Q8R1B4	2	2	1514	6531	NA	9427	4716	7995	7148	NA	4118	NA	0.685688513	0.163873126	5547	6420.3333	Up	0.210940476224347	Eukaryotic translation initiation factor 3 subunit C OS=Mus musculus GN=Eif3c PE=1 SV=1
NHSL2	B1AXH1	8	8	12400	10805.14	7784.71	13394.57	13513.29	13526	11503.86	6867.43	16110.71	4951.57	0.686085071	0.163622031	11579.542	10591.914	Down	-0.128614878754056	NHS-like protein 2 OS=Mus musculus GN=Nhsl2 PE=2 SV=1
FARSA	Q8C0C7	5	5	9137.8	11297.6	4045.2	24263.8	8670.8	15650	10740	3430.6	8968.2	NA	0.686433097	0.163401785	11483.04	9697.2	Down	-0.243864484908426	Phenylalanine--tRNA ligase alpha subunit OS=Mus musculus GN=Farsa PE=2 SV=1
VAT1L	Q80TB8	3	3	25307.33	35375.67	13846	108839	45366.67	68547	62775.67	7132	39978.67	5954.33	0.688169669	0.162304473	45746.934	36877.534	Down	-0.310932869935718	Synaptic vesicle membrane protein VAT-1 homolog-like OS=Mus musculus GN=Vat1l PE=2 SV=2
MRPL43	Q99N89	4	4	45845.5	42949.25	47505.5	22030.75	42150.25	42248.75	37681.75	69442.5	62053.25	12389.5	0.688274025	0.16223862	40096.25	44763.15	Up	0.158844247889084	39S ribosomal protein L43, mitochondrial OS=Mus musculus GN=Mrpl43 PE=2 SV=1
HIST1H1B	P43276	8	8	112248.62	59583.88	101444.5	29044.62	78567.38	50391.88	47279.62	82744.38	59765.5	102621.62	0.688619564	0.162020643	76177.8	68560.6	Down	-0.151990888670542	Histone H1.5 OS=Mus musculus GN=Hist1h1b PE=1 SV=2
COPRS	Q9CQ13	3	3	6055	6271	6724	2691	3814	4288	5287	2333	12686	NA	0.688950033	0.161812275	5111	6148.5	Up	0.266628899355725	Coordinator of PRMT5 and differentiation stimulator OS=Mus musculus GN=Coprs PE=1 SV=1
GLDC	Q91W43	1	1	9312	12452	2207	31547	15062	20533	12643	8201	5240	NA	0.689104137	0.161715143	14116	11654.25	Down	-0.276475171269393	Glycine dehydrogenase (decarboxylating), mitochondrial OS=Mus musculus GN=Gldc PE=1 SV=1
FH1	P97807	24	24	51567	52306.62	48477.75	63200.58	49369.46	60504.71	50968.79	57592.12	52332.38	31516.25	0.689193644	0.161658737	52984.282	50582.85	Down	-0.0669161165109588	Fumarate hydratase, mitochondrial OS=Mus musculus GN=Fh PE=1 SV=3
BCAS3	Q8CCN5	7	7	11066	12617	8460.43	16569.29	15407.14	15682.14	15703	3919.29	15755.14	6871.43	0.689402103	0.161527396	12823.972	11586.2	Down	-0.146435706595965	Breast carcinoma-amplified sequence 3 homolog OS=Mus musculus GN=Bcas3 PE=1 SV=2
RPS23	P62267	16	16	76714.88	89133.88	65845.44	128274.94	74744.81	104495.75	96589	97920	100095.38	63222.5	0.689520955	0.16145253	86942.79	92464.526	Up	0.088833589522676	40S ribosomal protein S23 OS=Mus musculus GN=Rps23 PE=1 SV=3
ATP5B	P56480	128	128	126953.44	137898.01	124896.12	122124.71	119094.68	135081.92	122360.61	171866.93	113589.07	112352.79	0.690026515	0.161134221	126193.39	131050.26	Up	0.0544838930603042	ATP synthase subunit beta, mitochondrial OS=Mus musculus GN=Atp5b PE=1 SV=2
NDUFS4	Q9CXZ1	16	16	70546.44	66003.75	80906.5	43816	64915.44	55313.81	58515.12	67139.75	69555.62	61787.5	0.690378593	0.160912684	65237.626	62462.36	Down	-0.0627172038902722	NADH dehydrogenase [ubiquinone] iron-sulfur protein 4, mitochondrial OS=Mus musculus GN=Ndufs4 PE=1 SV=3
PEPD	Q11136	1	1	1156	1402	1326	5662	2437	3539	2604	NA	2223	NA	0.690419924	0.160886684	2396.6	2788.6667	Up	0.21858635847472	Xaa-Pro dipeptidase OS=Mus musculus GN=Pepd PE=2 SV=3
ATP1B1	P14094	23	23	16723.96	32538.91	17060.35	159190.96	27094.43	76100.83	53996.13	18025.57	28605.26	13440.91	0.691029583	0.16050336	50521.722	38033.74	Down	-0.409624001737766	Sodium/potassium-transporting ATPase subunit beta-1 OS=Mus musculus GN=Atp1b1 PE=1 SV=1
HSP90B1	P08113	42	42	48459.56	61454.93	55826.78	59613.17	52064.41	55565.61	59352.98	61301.02	49848.93	42849.22	0.691879536	0.159969515	55483.77	53783.552	Down	-0.0449007814052189	Endoplasmin OS=Mus musculus GN=Hsp90b1 PE=1 SV=2
ADCY5	P84309	5	5	14768.2	17712.2	6241	23061	15634.2	22188	19666.8	10567.8	15658.8	NA	0.692025567	0.15987786	15483.32	17020.35	Up	0.136545850838318	Adenylate cyclase type 5 OS=Mus musculus GN=Adcy5 PE=1 SV=2
UBE2V2	Q9D2M8-2	18	18	31236.94	32987.75	25631.75	17751.19	29126.06	28968.12	28505.5	23457	31256.56	16920.44	0.692501548	0.159579251	27346.738	25821.524	Down	-0.0827946025560298	Isoform 2 of Ubiquitin-conjugating enzyme E2 variant 2 OS=Mus musculus GN=Ube2v2
MAP3K7	Q62073	3	3	5076	6571.33	3470	6124	7077.33	7391.33	8123.33	2304	7343.33	NA	0.693126823	0.159187294	5663.732	6290.4975	Up	0.151421120131674	Mitogen-activated protein kinase kinase kinase 7 OS=Mus musculus GN=Map3k7 PE=1 SV=1
PKM	P52480-2	80	80	136535.94	141978.8	132102.34	170360.3	155667.18	167597.53	169568.35	144260.59	133152.29	142642.62	0.693649239	0.158860086	147328.91	151444.28	Up	0.0397464766076384	Isoform M1 of Pyruvate kinase PKM OS=Mus musculus GN=Pkm
CHCHD3	Q9CRB9	26	26	100847.35	95548.08	56206.04	89666.85	96926.58	105232.58	95526.23	66788.85	113931.19	18175.5	0.694935046	0.158055786	87838.98	79930.87	Down	-0.136108509441067	Coiled-coil-helix-coiled-coil-helix domain-containing protein 3, mitochondrial OS=Mus musculus GN=Chchd3 PE=1 SV=1
TMOD2	Q9JKK7	19	19	108815.81	117833.19	103895.56	96023.44	100909.06	105437	104441.5	120327.38	101110.56	106299.06	0.694975204	0.158030691	105495.41	107523.1	Up	0.0274663807623697	Tropomodulin-2 OS=Mus musculus GN=Tmod2 PE=1 SV=2
LARS	Q8BMJ2	3	3	16739	25922	19730.33	59397.67	24599	36866.33	35403	11533.33	28595.67	15103.67	0.696652198	0.156983989	29277.6	25500.4	Down	-0.199277417666265	Leucine--tRNA ligase, cytoplasmic OS=Mus musculus GN=Lars PE=1 SV=2
GTPBP1	O08582	2	2	1418.5	2018	NA	7056.5	1635	3508	3046.5	NA	508.5	NA	0.696703549	0.156951977	3032	2354.3333	Down	-0.364951157728286	GTP-binding protein 1 OS=Mus musculus GN=Gtpbp1 PE=1 SV=2
CANX	P35564	10	10	24965.89	47327.11	48213	113531.44	43830.89	81019.22	72163.33	55144.89	44845.89	58351.67	0.697172558	0.156659716	55573.666	62305	Up	0.164946531758293	Calnexin OS=Mus musculus GN=Canx PE=1 SV=1
RCSD1	Q3UZA1	2	2	5772	4853	7213	4766	4829	6092	4389	6569	5979	NA	0.697371413	0.156535859	5486.6	5757.25	Up	0.0694674603860946	CapZ-interacting protein OS=Mus musculus GN=Rcsd1 PE=1 SV=1
ADCYAP1R1	P70205	1	1	NA	26821	NA	83445	22142	48586	36967	NA	18997	NA	0.69753481	0.156434114	44136	34850	Down	-0.340797229489946	Pituitary adenylate cyclase-activating polypeptide type I receptor OS=Mus musculus GN=Adcyap1r1 PE=2 SV=1
PGP	Q8CHP8	24	24	39532.88	37865.83	40334.83	32995.96	40532.88	38015.08	38972.62	26448.33	45770.25	35029.08	0.69777396	0.156285242	38252.476	36847.072	Down	-0.0540031511837513	Phosphoglycolate phosphatase OS=Mus musculus GN=Pgp PE=1 SV=1
SEPT3	Q9Z1S5	41	41	52469.1	53247.22	52365	63564.88	56842.22	58309.78	56673.03	39702.38	59335.65	55938.32	0.697843685	0.156241847	55697.684	53991.832	Down	-0.0448761694265033	Neuronal-specific septin-3 OS=Mus musculus GN=Sept3 PE=1 SV=2
ENDOD1	Q8C522	5	5	12383	11274.5	8990	9819.5	13875	11266	8590	8686.75	8180	27502	0.69797087	0.156162702	11268.4	12844.95	Up	0.188918592645335	Endonuclease domain-containing 1 protein OS=Mus musculus GN=Endod1 PE=1 SV=2
ANK2	Q8C8R3-4	134	134	26655.99	36334.51	29710.35	58693.93	42887.8	49281.58	57572.02	25558.91	44862.37	33195.01	0.698537683	0.155810161	38856.516	42093.978	Up	0.115457304832103	Isoform 4 of Ankyrin-2 OS=Mus musculus GN=Ank2
BRINP1	Q920P3	1	1	3262	16887	NA	17482	9313	18272	12150	NA	9957	NA	0.698560131	0.155796205	11736	13459.667	Up	0.197701905421487	BMP/retinoic acid-inducible neural-specific protein 1 OS=Mus musculus GN=Brinp1 PE=2 SV=1
SLC6A1	P31648	2	2	7566.5	16480.5	10827	34298	10007.5	16798.5	24947.5	2929.5	13728	8507	0.699275705	0.15535156	15835.9	13382.1	Down	-0.242894332059084	Sodium- and chloride-dependent GABA transporter 1 OS=Mus musculus GN=Slc6a1 PE=1 SV=2
ALCAM	Q61490	17	17	23718	37288.5	24216.56	80596.69	40286.38	59979.62	53426.44	20012.56	32246.38	12716.12	0.700105728	0.154836369	41221.226	35676.224	Down	-0.208424486746852	CD166 antigen OS=Mus musculus GN=Alcam PE=1 SV=3
LUC7L2	Q7TNC4-3	3	3	16671.67	16251.67	10386.33	22548.67	16284.33	18551	18068.67	6525.67	16980	NA	0.700156902	0.154804626	16428.534	15031.335	Down	-0.128230600024903	Isoform 3 of Putative RNA-binding protein Luc7-like 2 OS=Mus musculus GN=Luc7l2
TAGLN	P37804	1	1	129231	107647	113013	182386	109067	146509	101361	191366	122587	121190	0.700491468	0.15459715	128268.8	136602.6	Up	0.090814650039982	Transgelin OS=Mus musculus GN=Tagln PE=1 SV=3
CACNB1	Q8R3Z5	11	11	35063.44	34466.56	34906.67	21364.44	37231.33	36268.11	37971.44	27954.33	44010.89	25627.89	0.700793997	0.154409627	32606.488	34366.532	Up	0.0758452148689698	Voltage-dependent L-type calcium channel subunit beta-1 OS=Mus musculus GN=Cacnb1 PE=1 SV=1
MAPT	P10637	55	55	133676.15	122578.5	121384.81	116826.71	122446.5	109998.1	125117.44	136649.19	162468.44	104577.04	0.701279057	0.154109131	123382.53	127762.04	Up	0.0503210950072967	Microtubule-associated protein tau OS=Mus musculus GN=Mapt PE=1 SV=3
TOMM6	Q9CQN3	1	1	99835	92785	144994	49083	59379	41948	44199	255689	72324	119276	0.701585054	0.153919671	89215.2	106687.2	Up	0.258025661734741	Mitochondrial import receptor subunit TOM6 homolog OS=Mus musculus GN=Tomm6 PE=3 SV=1
PTN	P63089	21	21	67641.24	53244.38	47481.05	23700.57	56157.43	37689.05	53982.81	58012.38	74185.81	43453.9	0.70231087	0.15347061	49644.934	53464.79	Up	0.106942590882548	Pleiotrophin OS=Mus musculus GN=Ptn PE=2 SV=1
PLXNB1	Q8CJH3	2	2	1040	1295	NA	10707	3202.5	7644.5	5049	NA	2746.5	NA	0.70235841	0.153441213	4061.125	5146.6667	Up	0.341758913180306	Plexin-B1 OS=Mus musculus GN=Plxnb1 PE=1 SV=2
KRT76	Q3UV17	1	1	8429	9509	5107	127012	7122	38926	13941	NA	10455	NA	0.702520089	0.153341253	31435.8	21107.333	Down	-0.574664155867254	Keratin, type II cytoskeletal 2 oral OS=Mus musculus GN=Krt76 PE=2 SV=1
VPS37C	Q8R105-2	1	1	27609	35221	39288	32791	29625	41302	14700	47331	13161	NA	0.70297107	0.153062548	32906.8	29123.5	Down	-0.176201993395245	Isoform 2 of Vacuolar protein sorting-associated protein 37C OS=Mus musculus GN=Vps37c
RTN1	Q8K0T0	6	6	30013.33	39768.67	13452	175425.33	67454	106219.67	68028.17	18784.33	57854.83	8048.5	0.704301746	0.152241235	65222.666	51787.1	Down	-0.332780641001636	Reticulon-1 OS=Mus musculus GN=Rtn1 PE=1 SV=1
TMX4	Q8C0L0	3	3	1269.67	5754.67	NA	19935	4286.33	11955	7506.33	NA	NA	NA	0.7043827	0.152191319	7811.4175	9730.665	Up	0.316954032109813	Thioredoxin-related transmembrane protein 4 OS=Mus musculus GN=Tmx4 PE=2 SV=2
PLCB1	Q9Z1B3	19	19	25541	31054.76	19048.47	51194.88	29749.24	41353.18	35920.76	21240.82	27879.47	16252.53	0.704571969	0.152074639	31317.67	28529.352	Down	-0.134529902996015	1-phosphatidylinositol 4,5-bisphosphate phosphodiesterase beta-1 OS=Mus musculus GN=Plcb1 PE=2 SV=2
RAB23	P35288	3	3	14982.67	18384.67	14696.67	19404.67	18829	24904	16429	10184	11868	NA	0.705017414	0.151800156	17259.536	15846.25	Down	-0.123252211727556	Ras-related protein Rab-23 OS=Mus musculus GN=Rab23 PE=1 SV=2
LETM1	Q9Z2I0	7	7	13241.86	27671.14	4452.43	96792.71	24879.57	46349.86	39390.71	12290.86	25301.57	7905.71	0.705499384	0.151503361	33407.542	26247.742	Down	-0.347980520370868	LETM1 and EF-hand domain-containing protein 1, mitochondrial OS=Mus musculus GN=Letm1 PE=2 SV=1
PCBP3	P57722	12	12	69152.91	71566.64	91565	86054.55	70518.45	73249	80375.91	67049	73086.64	114027.36	0.705582794	0.151452018	77771.51	81557.582	Up	0.0685772530978955	Poly(rC)-binding protein 3 OS=Mus musculus GN=Pcbp3 PE=2 SV=3
CIITA	P79621-3	1	1	2035339	1627783	411254	1581526	2059894	1922160	1928530	617278	2493145	NA	0.706049089	0.151165103	1543159.2	1740278.3	Up	0.173431089262906	Isoform 3 of MHC class II transactivator OS=Mus musculus GN=Ciita
SNX30	Q8CE50	1	1	1289	1403	NA	6800	2571	3000	1887	NA	NA	NA	0.706320384	0.15099826	3015.75	2443.5	Down	-0.303567732274843	Sorting nexin-30 OS=Mus musculus GN=Snx30 PE=2 SV=1
MARK2	Q05512-3	12	12	39716.75	46474	40326.75	52152.58	54721.08	50978.83	59501	16849.5	55391	33796.67	0.706906896	0.150637781	46678.232	43303.4	Down	-0.108269614372317	Isoform 3 of Serine/threonine-protein kinase MARK2 OS=Mus musculus GN=Mark2
BOLA2	Q8BGS2-2	1	1	8611	9182	18153	10676	11140	14227	9135	NA	14153	NA	0.70709501	0.150522227	11552.4	12505	Up	0.114312456231575	Isoform 2 of BolA-like protein 2 OS=Mus musculus GN=Bola2
MYO6	Q64331	7	7	6050	9477.5	2627	15700.25	12201.25	11396	15870.25	4324.75	10416	NA	0.707523599	0.15025907	9211.2	10501.75	Up	0.18916873454995	Unconventional myosin-VI OS=Mus musculus GN=Myo6 PE=1 SV=1
EMD	O08579	8	8	7100.12	16791.5	9345.12	29824.12	16849	25286.88	32529.88	4011.75	16998	13348.38	0.708240177	0.14981944	15981.972	18434.978	Up	0.206000262755342	Emerin OS=Mus musculus GN=Emd PE=1 SV=1
RBM20	Q3UQS8	1	1	353198	160156	197334	106685	246327	174193	164334	172915	145363	307623	0.708602424	0.149597366	212740	192885.6	Down	-0.141345876494665	RNA-binding protein 20 OS=Mus musculus GN=Rbm20 PE=1 SV=3
SNCA	O55042	47	47	111576.63	98631.53	93982.16	152406	97118.67	122897.26	106275.58	108891	122110.58	63974.72	0.708648984	0.149568831	110743	104829.83	Down	-0.0791662078095562	Alpha-synuclein OS=Mus musculus GN=Snca PE=1 SV=2
EEF1D	P57776-3	20	20	49238.05	44369.68	65384.11	34189.26	42904.95	42403.16	43948.68	55871.21	54978.74	50316.05	0.708869631	0.149433629	47217.21	49503.568	Up	0.0682197148497029	Isoform 3 of Elongation factor 1-delta OS=Mus musculus GN=Eef1d

CACNB3	P54285	8	8	25028	27654.12	13748.88	20203.88	25180.5	22731.5	26210.62	27373.5	21123.5	5250.25	0.709188925	0.149238055	22363.076	20537.874	Down	-0.122831794616484	Voltage-dependent L-type calcium channel subunit beta-3 OS=Mus musculus GN=Cacnb3 PE=2 SV=2
KBTBD11	Q8BNW9	3	3	15407	26522	22705	17907	14497	16964	16180.5	27387	22511.5	NA	0.709253978	0.14919822	19407.6	20760.75	Up	0.0972368420033164	Kelch repeat and BTB domain-containing protein 11 OS=Mus musculus GN=Kbtbd11 PE=1 SV=3
GLG1	Q61543	16	16	14590.06	23953.56	16995.19	59082.25	23399.62	41530.31	39313.75	26935.56	26651.12	21116.19	0.7105218	0.148422593	27604.136	31109.386	Up	0.172465474981277	Golgi apparatus protein 1 OS=Mus musculus GN=Glg1 PE=1 SV=1
GDAP1	O88741	2	2	2916	4701	618	14072	10636.5	11689	11877.5	NA	1325.5	NA	0.710555186	0.148402186	6588.7	8297.3333	Up	0.332653906760403	Ganglioside-induced differentiation-associated protein 1 OS=Mus musculus GN=Gdap1 PE=1 SV=1
RGS12	Q8CGE9	9	9	19235.75	25718.38	22067.12	21355.12	28188.12	29688.5	25830.62	18169.5	26595.5	21283.38	0.710611789	0.148367592	23312.898	24313.5	Up	0.0606292331159625	Regulator of G-protein signaling 12 OS=Mus musculus GN=Rgs12 PE=1 SV=2
FAM98B	Q80VD1	4	4	13607	18331.75	22117	31107.5	20537.25	34455.5	23634.75	3382.5	35887.25	NA	0.711340547	0.147922436	21140.1	24340	Up	0.203346966867334	Protein FAM98B OS=Mus musculus GN=Fam98b PE=2 SV=1
WDR44	Q6NVE8	1	1	4568	5898	NA	12870	5099	8707	7246	NA	7774	NA	0.711819433	0.14763016	7108.75	7909	Up	0.153899395237001	WD repeat-containing protein 44 OS=Mus musculus GN=Wdr44 PE=1 SV=1
FAM131B	Q3TY60	12	12	11508.64	12030.09	9011.82	16013.36	13772.55	14573.27	15795.27	10632.36	16399.55	8617.55	0.712092998	0.147463285	12467.292	13203.6	Up	0.0827832044461094	Protein FAM131B OS=Mus musculus GN=Fam131b PE=1 SV=1
C1QC	Q02105	1	1	NA	2899	NA	4289	1531	4949	2234	NA	NA	NA	0.712097055	0.14746081	2906.3333	3591.5	Up	0.305386337406943	Complement C1q subcomponent subunit C OS=Mus musculus GN=C1qc PE=2 SV=2
VCPIP1	Q8CDG3	2	2	5165	5805.5	4143	7823	7083.5	6970	7102	4051.5	7480.5	NA	0.712312191	0.147329623	6004	6401	Up	0.092373331644986	Deubiquitinating protein VCIP135 OS=Mus musculus GN=Vcpip1 PE=2 SV=1
ADPRHL2	Q8CG72	2	2	6045.5	6239	5459.5	12964	7210	9021.5	6811	1489	9472	NA	0.712535051	0.147193767	7583.6	6698.375	Down	-0.179071724967444	Poly(ADP-ribose) glycohydrolase ARH3 OS=Mus musculus GN=Adprhl2 PE=1 SV=1
MRPL57	Q9CQF8	1	1	112188	96792	96930	134681	99881	124173	104800	37702	130075	NA	0.712658881	0.147118298	108094.4	99187.5	Down	-0.124061560867791	Ribosomal protein 63, mitochondrial OS=Mus musculus GN=Mrp63 PE=2 SV=1
NDUFB5	Q9CQH3	2	2	43997	34884.5	65226.5	78851.5	49363.5	75012	60398.5	20642	39693.5	NA	0.712770893	0.147050044	54464.6	48936.5	Down	-0.154407913684497	NADH dehydrogenase [ubiquinone] 1 beta subcomplex subunit 5, mitochondrial OS=Mus musculus GN=Ndufb5 PE=1 SV=1
NBEA	Q9EPN1-4	3	3	2572.67	4501.67	5999.67	10517.67	5028.67	6904.33	6627.33	3141.67	6670.67	8291.33	0.713171511	0.146806014	5724.07	6327.066	Up	0.144495330246854	Isoform 4 of Neurobeachin OS=Mus musculus GN=Nbea
SPTB	P15508	5	5	NA	2619.5	237.75	13104.5	5865.5	8876.5	5330.75	1079	1336.75	NA	0.713477251	0.14661987	5456.8125	4155.75	Down	-0.392949604760886	Spectrin beta chain, erythrocytic OS=Mus musculus GN=Sptb PE=1 SV=4
MCAT	Q8R3F5	3	3	8782	8609.67	12385	8924	9264	8870.33	10392.67	11465.67	13822.67	6208	0.713896017	0.146365041	9592.934	10151.868	Up	0.0817011794266787	Malonyl-CoA-acyl carrier protein transacylase, mitochondrial OS=Mus musculus GN=Mcat PE=1 SV=3
ISCA1	Q9D924	8	8	22266.12	21595	23846.62	23011.75	23478.5	26987.5	23297.5	18570.25	29447.12	19985.25	0.714206228	0.146176367	22839.598	23657.524	Up	0.0507618308659364	Iron-sulfur cluster assembly 1 homolog, mitochondrial OS=Mus musculus GN=Isca1 PE=2 SV=1
DLGAP1	Q9D415-6	7	7	12304	13655.29	9762.71	12450.86	16877.57	12896.43	14607.14	5661.14	21561.57	3691.14	0.714332207	0.146099769	13010.086	11683.484	Down	-0.155159950478429	Isoform 6 of Disks large-associated protein 1 OS=Mus musculus GN=Dlgap1
MAP4	P27546-4	23	23	56031.26	52752.22	61254.87	67046.04	47744.17	57979.52	55018.04	66294.83	70987.57	45199	0.714522157	0.1459843	56965.712	59095.792	Up	0.0529615910996259	Isoform 4 of Microtubule-associated protein 4 OS=Mus musculus GN=Map4
ARFGAP3	Q9D8S3	1	1	25658	27772	10942	40068	34700	37559	35379	20793	27126	NA	0.714704679	0.145873375	27828	30214.25	Up	0.118691905042978	ADP-ribosylation factor GTPase-activating protein 3 OS=Mus musculus GN=Arfgap3 PE=1 SV=2
GRIN2A	P35436	6	6	7064	10627.2	4211.4	23182.4	17223.8	21999.4	22479.8	6397.6	20342	1668.4	0.714919574	0.145742812	12461.76	14577.44	Up	0.226229547174004	Glutamate receptor ionotropic, NMDA 2A OS=Mus musculus GN=Grin2a PE=1 SV=2
ETHE1	Q9DCM0	8	8	30722.38	35775.12	19969.75	36025.75	31805.62	36804.5	31388.12	24511.25	34185.5	19151.88	0.715020691	0.145681391	30859.724	29208.25	Down	-0.0793492366494658	Persulfide dioxygenase ETHE1, mitochondrial OS=Mus musculus GN=Ethe1 PE=1 SV=2
SERPINH1	P19324	1	1	10779	15057	22024	13353	15109	18988	15471	11939	18413	NA	0.71503716	0.145671388	15264.4	16202.75	Up	0.0860678118272551	Serpin H1 OS=Mus musculus GN=Serpinh1 PE=1 SV=3
RANBP3	Q9CT10	1	1	2817	3057	NA	3525	2674	3915	3323	NA	2398	NA	0.715641126	0.14530471	3018.25	3212	Up	0.0897595843986482	Ran-binding protein 3 OS=Mus musculus GN=Ranbp3 PE=1 SV=2
SYNGAP1	F6SEU4	71	71	26180.17	33018.13	28855	44114.91	41720.39	43177.09	49642.51	28846.51	43310.06	20969.01	0.715827799	0.14519144	34777.72	37189.036	Up	0.0967139976529646	Ras/Rap GTPase-activating protein SynGAP OS=Mus musculus GN=Syngap1 PE=1 SV=2
CSRP1	P97315	13	13	168826.38	128526.62	123513.08	87365	157318.38	118220.15	134470.38	96084.85	172294.54	107847.92	0.71628162	0.144916193	133109.89	125783.57	Down	-0.0816743234111327	Cysteine and glycine-rich protein 1 OS=Mus musculus GN=Csrp1 PE=1 SV=3
APBB1	Q9QXJ1	2	2	5270.5	4500.5	3327.5	10850.5	8569	10725	6510.5	NA	4863	NA	0.717026839	0.144464588	6503.6	7366.1667	Up	0.179675511929605	Amyloid beta A4 precursor protein-binding family B member 1 OS=Mus musculus GN=Apbb1 PE=1 SV=3
PSD	Q5DTT2	2	2	11267	18438	NA	34397.5	14424	24422.5	25917.5	NA	15544.5	NA	0.717776281	0.144010897	19631.625	21961.5	Up	0.161796999462343	PH and SEC7 domain-containing protein 1 OS=Mus musculus GN=Psd PE=1 SV=2
PGM3	Q9CYR6	1	1	4533	8945	NA	18751	6879	12317	11969	NA	8911	NA	0.718180922	0.143766136	9777	11065.667	Up	0.178626613040759	Phosphoacetylglucosamine mutase OS=Mus musculus GN=Pgm3 PE=1 SV=1
OSBPL6	Q8BXR9	4	4	17100.25	23791.75	19773.75	22625	26598.5	28169	28613.75	13790.75	23182.75	NA	0.719623156	0.142894871	21977.85	23439.063	Up	0.0928646065162625	Oxysterol-binding protein-related protein 6 OS=Mus musculus GN=Osbpl6 PE=2 SV=1
GRIA2	P23819-3	7	7	23271.14	35461.86	30046.14	56186.43	28270.71	44464.29	45272.71	32263.29	29459.43	34127.57	0.719837462	0.142765555	34647.256	37117.458	Up	0.0993568124061889	Isoform 3 of Glutamate receptor 2 OS=Mus musculus GN=Gria2
NDUFS2	Q91WD5	8	8	25129.38	26365.12	29099.12	53930	26998.38	45917	36803.88	30335.88	29147.5	31051.88	0.720352955	0.142454658	32304.4	34651.228	Up	0.101175801083666	NADH dehydrogenase [ubiquinone] iron-sulfur protein 2, mitochondrial OS=Mus musculus GN=Ndufs2 PE=1 SV=1
PIN1	Q9QUR7	20	20	92935.05	74462.65	74220.1	56611.95	77174.45	76036.55	82356.4	53013.6	109220.05	26202.45	0.720798406	0.142186183	75080.84	69365.81	Down	-0.114220048957597	Peptidyl-prolyl cis-trans isomerase NIMA-interacting 1 OS=Mus musculus GN=Pin1 PE=1 SV=1
ISCU	Q9D7P6	3	3	84313.67	83773	104720.67	59220	73804.67	70612	83525.67	129506	88085	60081.33	0.72191086	0.141516425	81166.402	86362	Up	0.0895139920056193	Iron-sulfur cluster assembly enzyme ISCU, mitochondrial OS=Mus musculus GN=Iscu PE=1 SV=1
BABAM1	Q3UI43	2	2	23065	31107.5	37897.5	19892	33870.5	33261	30672	24648.5	31177.5	18034.5	0.722082012	0.141413474	29166.5	27558.7	Down	-0.0818044374443039	BRISC and BRCA1-A complex member 1 OS=Mus musculus GN=Babam1 PE=2 SV=1
CCT6B	Q61390	2	2	22668	28583.5	4274	39253.5	20293	32219.5	22652.5	7960.5	17932	NA	0.722102952	0.14140088	23014.4	20191.125	Down	-0.188815534736903	T-complex protein 1 subunit zeta-2 OS=Mus musculus GN=Cct6b PE=2 SV=4
FXR2	Q9WVR4	4	4	33461.75	33735.75	33161.5	35222.75	32722.5	34186	35586.5	19334.25	47142	22519.5	0.722148382	0.141373558	33660.85	31753.65	Down	-0.0841491724178965	Fragile X mental retardation syndrome-related protein 2 OS=Mus musculus GN=Fxr2 PE=1 SV=1
VCAM1	P29533	12	12	27707	34868.08	32211.17	35013.75	41591.5	47287.83	46742.25	22234.08	46130.25	21240	0.722735712	0.141020485	34278.3	36726.882	Up	0.0995408587470771	Vascular cell adhesion protein 1 OS=Mus musculus GN=Vcam1 PE=1 SV=1
HINT2	Q9D0S9	13	13	56426.92	52014.23	70870.62	41432.15	49180.15	43378.69	44510.62	74612.23	61388	59990.85	0.723243446	0.140715493	53984.814	56776.078	Up	0.0727295611435214	Histidine triad nucleotide-binding protein 2, mitochondrial OS=Mus musculus GN=Hint2 PE=1 SV=1
CRKL	P47941	6	6	28282.67	28919.67	18894.83	29100.5	30989.67	38233.83	34258.67	25504.5	35358.67	12424.5	0.723413776	0.140613225	27237.468	29156.034	Up	0.0982018915316941	Crk-like protein OS=Mus musculus GN=Crkl PE=1 SV=2
RPL18	P35980	9	9	88655.67	105106.44	99001.22	96000.67	104131.11	103128.44	112971.78	73187.89	141741.89	84916.78	0.723430986	0.140602893	98579.022	103189.36	Up	0.0659415909400852	60S ribosomal protein L18 OS=Mus musculus GN=Rpl18 PE=2 SV=3
C2CD2L	Q80X80	28	28	11448.44	21514.74	13350.93	37477.26	24639.11	37707.44	34329.3	16520.63	24489.37	8467.04	0.724298706	0.14008229	21686.096	24302.756	Up	0.164349570897672	C2 domain-containing protein 2-like OS=Mus musculus GN=C2cd2l PE=1 SV=3
FKBP4	P30416	4	4	56831.5	79674.75	88087	81415.75	64560.75	66327.75	66373.25	67114.25	71828	86220.75	0.724545265	0.139934477	74113.95	71572.8	Down	-0.0503336970108294	Peptidyl-prolyl cis-trans isomerase FKBP4 OS=Mus musculus GN=Fkbp4 PE=1 SV=5
SNAP47	Q8R570-2	1	1	5865	10171	6786	17166	8423	14761	10032	3713	5526	NA	0.724627644	0.139885102	9682.2	8508	Down	-0.186514862183809	Isoform 2 of Synaptosomal-associated protein 47 OS=Mus musculus GN=Snap47
PCSK1N	Q9QXV0	13	13	28741.92	31270.42	23288.33	35856.42	34536.25	36920.75	33335.5	20391.17	53756.5	21328.83	0.7255506	0.139332294	30738.668	33146.55	Up	0.108804067773641	ProSAAS OS=Mus musculus GN=Pcsk1n PE=1 SV=2
RAB10	P61027	1	1	2212	13774	NA	37783	14777	23989	16558	NA	NA	NA	0.725678931	0.139255486	17136.5	20273.5	Up	0.242522695935577	Ras-related protein Rab-10 OS=Mus musculus GN=Rab10 PE=1 SV=1
GAB1	Q9QYY0	1	1	8368	9768	10759	5267	8004	4829	7947	NA	10395	NA	0.725791579	0.139188075	8433.2	7723.6667	Down	-0.126794266825412	GRB2-associated-binding protein 1 OS=Mus musculus GN=Gab1 PE=1 SV=2
TPM1	P58771-2	27	27	40112	40039	26730.2	41168.64	38459.4	40280.48	42235.16	24780.32	53399.48	11311.8	0.7261023	0.139002187	37301.848	34401.448	Down	-0.116777814888186	Isoform 2 of Tropomyosin alpha-1 chain OS=Mus musculus GN=Tpm1
ZFYVE19	Q9DAZ9	3	3	5515	6872	4405	8071.33	7112	9233.67	7154	1545	7636.67	3541.33	0.726447535	0.138795746	6395.066	5822.134	Down	-0.1354112040973	Zinc finger FYVE domain-containing protein 19 OS=Mus musculus GN=Zfyve19 PE=1 SV=2
PHYHIPL	Q8BGT8	1	1	18388	25796	4165	34461	24438	34769	35493	18834	15112	15416	0.726457521	0.138789775	21449.6	23924.8	Up	0.157556120444316	Phytanoyl-CoA hydroxylase-interacting protein-like OS=Mus musculus GN=Phyhipl PE=2 SV=1
ARHGAP32	Q811P8	20	20	17280.95	18657.75	13941.15	19295.2	23233.5	24892.05	24963.1	8653.7	25840.95	14967.1	0.726695134	0.138647748	18481.71	19863.38	Up	0.104012889466061	Rho GTPase-activating protein 32 OS=Mus musculus GN=Arhgap32 PE=1 SV=2
SEPT2	P42208	16	16	57928.8	57731.47	69321.93	35123.07	55855.4	51885.67	55747.73	53197.93	48502.93	81306.67	0.726761014	0.138608378	55192.134	58128.186	Up	0.0747752189336524	Septin-2 OS=Mus musculus GN=Sept2 PE=1 SV=2
DLG2	Q91XM9-3	27	27	84484.15	94948.42	94168.35	114942.73	102883.08	96257.62	115540.54	93608.5	105949.19	92243.58	0.727184765	0.138355228	98285.346	100719.89	Up	0.0353003183061685	Isoform 3 of Disks large homolog 2 OS=Mus musculus GN=Dlg2
ARHGAP32	Q811P8-2	21	21	16494.71	17842.43	13277.29	18533.05	22158.33	23816.67	23918.05	8241.62	24691	14254.38	0.727215925	0.138336619	17661.162	18984.344	Up	0.104229880885876	Isoform 2 of Rho GTPase-activating protein 32 OS=Mus musculus GN=Arhgap32
CCDC93	Q7TQK5	1	1	5996	7832	NA	22126	12454	15142	17590	NA	8552	NA	0.727799933	0.137987989	12102	13761.333	Up	0.18537476951381	Coiled-coil domain-containing protein 93 OS=Mus musculus GN=Ccdc93 PE=2 SV=1
EPHA4	Q03137	5	5	4674.8	8214.6	5255.4	18626	7020.4	12046.8	12120.2	8485.4	6597	NA	0.727826509	0.13797213	8758.24	9812.35	Up	0.163957710619485	Ephrin type-A receptor 4 OS=Mus musculus GN=Epha4 PE=1 SV=2
RPL19	P84099	10	10	53913.7	66674.7	52922.8	68505.2	51931.3	64223	73184.8	63625.5	82281.9	28614.5	0.727935562	0.137907063	58789.54	62385.94	Up	0.0856614341307785	60S ribosomal protein L19 OS=Mus musculus GN=Rpl19 PE=1 SV=1
ADGRL3	Q80TS3-4	13	13	23351	30851.25	19890.42	33018.75	27564.42	34385.25	33084.67	28118.5	33502.83	13808.58	0.728172324	0.137765831	26935.168	28579.966	Up	0.0855131365595163	Isoform 4 of Latrophilin-3 OS=Mus musculus GN=Lphn3
NME2	Q01768	11	11	58914.09	66502.91	53405.27	59835.36	55970.18	58817.27	63814.09	70729.36	56657.82	51700.27	0.728388165	0.137637119	58925.562	60343.762	Up	0.034311027775958	Nucleoside diphosphate kinase B OS=Mus musculus GN=Nme2 PE=1 SV=1
NECTIN1	Q9JKF6	3	3	22259	23918.33	43704.33	38230.67	27928.67	28054	35819.67	15170.33	40455.33	49423.33	0.728783616	0.137401399	31208.2	33784.532	Up	0.114437721214997	Poliovirus receptor-related protein 1 OS=Mus musculus GN=Pvrl1 PE=1 SV=3
GPD2	Q64521	20	20	40962.3	50877.45	50417	62969	43313.95	58083.55	54105.3	42639.75	41907.85	42588.3	0.729494708	0.136977854	49707.94	47864.95	Down	-0.0545067139567359	Glycerol-3-phosphate dehydrogenase, mitochondrial OS=Mus musculus GN=Gpd2 PE=1 SV=2
PUM1	Q80U78-3	6	6	14525.17	19006	6816.33	21284	18478.33	20652.33	24603.83	3469.5	18993.5	20399.17	0.729760426	0.136819692	16021.966	17623.666	Up	0.137462872034025	Isoform 3 of Pumilio homolog 1 OS=Mus musculus GN=Pum1
UBA5	Q8VE47	4	4	11438	15195	18507.67	18450	13498.67	19226.67	19547.67	14207	15516.33	2391.67	0.730093919	0.136621269	15417.868	14177.868	Down	-0.120962678258877	Ubiquitin-like modifier-activating enzyme 5 OS=Mus musculus GN=Uba5 PE=2 SV=2
LUC7L3	Q5SUF2-3	1	1	NA	4365	NA	6554	4869	4934	6331	NA	NA	NA	0.730306875	0.136494611	5262.6667	5632.5	Up	0.097981389950843	Isoform 3 of Luc7-like protein 3 OS=Mus musculus GN=Luc7l3
PPP2CA	P63330	8	8	18601.62	24301.62	28319.38	45639.75	25411.88	27442.38	32043.5	34531.5	21270.25	16925.25	0.730981399	0.136093674	28454.85	26442.576	Down	-0.105811845218503	Serine/threonine-protein phosphatase 2A catalytic subunit alpha isoform OS=Mus musculus GN=Ppp2ca PE=1 SV=1
MARC2	Q922Q1	1	1	3414	3774	2897	7923	4060	7184	5051	NA	2739	NA	0.731169974	0.135981652	4413.6	4991.3333	Up	0.177469368143324	MOSC domain-containing protein 2, mitochondrial OS=Mus musculus GN=Marc2 PE=1 SV=1
GDAP1L1	Q8VE33	2	2	3655	11222	2271.5	30855	6615	20256.5	17367.5	6450.5	8510	NA	0.731233454	0.135943948	10923.7	13146.125	Up	0.267176009395215	Ganglioside-induced differentiation-associated protein 1-like 1 OS=Mus musculus GN=Gdap1l1 PE=2 SV=1
TUBB6	Q922F4	34	34	29085.06	32480.72	29769.59	44814.28	26644.47	37118.47	34027.28	23577.16	27860.03	33070.84	0.731327386	0.135888163	32558.824	31130.756	Down	-0.0647079788966391	Tubulin beta-6 chain OS=Mus musculus GN=Tubb6 PE=1 SV=1
DOC2A	Q7TNF0	2	2	17785	20761	8563	12637	19223	19480	18639	9180	20888.5	NA	0.731571727	0.135743087	15793.8	17046.875	Up	0.110148964976104	Double C2-like domain-containing protein alpha OS=Mus musculus GN=Doc2a PE=2 SV=1
MAP6D1	Q14BB9	3	3	18402	22892.33	24145.67	17355	26532.67	18259.33	19773.33	19185.33	21106.67	38566.67	0.732020175	0.135476949	21865.534	23378.266	Up	0.0965093447048327	MAP6 domain-containing protein 1 OS=Mus musculus GN=Map6d1 PE=1 SV=1
MACROD2	Q3UYG8	1	1	NA	6693	17830	13556	14716	15845	15563	NA	11220	NA	0.73229436	0.135314311	13198.75	14209.333	Up	0.106437564230462	O-acetyl-ADP-ribose deacetylase MACROD2 OS=Mus musculus GN=Macrod2 PE=2 SV=1
BANF1	O54962	3	3	50752	39706.33	11032.67	85319.33	58532	51374.67	69508.67	30112.67	66834.33	NA	0.732377794	0.135264832	49068.466	54457.585	Up	0.150336835468672	Barrier-to-autointegration factor OS=Mus musculus GN=Banf1 PE=1 SV=1
SYN1	O88935-3	87	87	121961.66	109546.71	101232.83	125836.17	105139.82	125692.44	111592.34	91474.18	110227.84	111882.74	0.732476696	0.135206188	112743.44	110173.91	Down	-0.0332608695594146	Isoform 3 of Synapsin-1 OS=Mus musculus GN=Syn1
RAB11FIP5	Q8R361	11	11	59451.45	67375	65040	49387.82	70978.36	60515.91	65514.82	62127.45	80186.18	54066.55	0.732553642	0.135160568	62446.526	64482.182	Up	0.046279249132646	Rab11 family-interacting protein 5 OS=Mus musculus GN=Rab11fip5 PE=1 SV=2
ACAN	Q61282	1	1	11419	9648	14680	7736	11066	9487	11285	NA	13791	NA	0.732805135	0.135011496	10909.8	11521	Up	0.078641291075605	Aggrecan core protein OS=Mus musculus GN=Acan PE=1 SV=2
UFC1	Q9CR09	1	1	103559	129999	166362	103011	91231	140854	130268	109654	103363	79656	0.733633471	0.134520863	118832.4	112759	Down	-0.0756856562007936	Ubiquitin-fold modifier-conjugating enzyme 1 OS=Mus musculus GN=Ufc1 PE=2 SV=1
EDF1	Q9JMG1	1	1	17689	36297	NA	36504	16584	30398	24222	NA	32332	NA	0.733794415	0.134425598	26768.5	28984	Up	0.114720412537488	Endothelial differentiation-related factor 1 OS=Mus musculus GN=Edf1 PE=1 SV=1
PTCD3	Q14C51	1	1	7601	NA	NA	11920	6234	12176	9195	NA	6898	NA	0.733810565	0.13441604	8585	9423	Up	0.134368309483624	Pentatricopeptide repeat domain-containing protein 3, mitochondrial OS=Mus musculus GN=Ptcd3 PE=2 SV=2
RHOB	P62746	8	8	57023.75	99419	64677.88	119409.5	103842.62	122880.62	145028.88	67425.88	102449.38	44899.5	0.735105631	0.133650251	88874.55	96536.852	Up	0.119309432333181	Rho-related GTP-binding protein RhoB OS=Mus musculus GN=Rhob PE=1 SV=1
CSPG5	Q71M36-4	4	4	4741.75	7204.75	5660.75	7890	7497	9253.5	8721.5	3427.25	7000.5	6684.25	0.735138416	0.133630882	6598.85	7017.4	Up	0.0887219761717299	Isoform 4 of Chondroitin sulfate proteoglycan 5 OS=Mus musculus GN=Cspg5
MRPL44	Q9CY73	2	2	986.5	1164.5	NA	3030.5	1805	2595	1455.5	NA	NA	NA	0.735180352	0.133606108	1746.625	2025.25	Up	0.213530112306629	39S ribosomal protein L44, mitochondrial OS=Mus musculus GN=Mrpl44 PE=2 SV=3
VPS28	Q9D1C8	5	5	21426.6	28509.6	22776.8	22836.4	34507.2	25869.4	31282.2	20536.6	31364.2	NA	0.735920046	0.133169367	26011.32	27263.1	Up	0.0678100019338	Vacuolar protein sorting-associated protein 28 homolog OS=Mus musculus GN=Vps28 PE=2 SV=1
SLC25A4	P48962	16	16	122254.75	207896.88	109797.5	381635.56	174921.81	283661.25	264781.5	111063.31	144132.69	81301	0.736017558	0.133111825	199301.3	176987.95	Down	-0.171299981010042	ADP/ATP translocase 1 OS=Mus musculus GN=Slc25a4 PE=1 SV=4
AARSD1	Q3THG9	4	4	7459.25	10401.5	1998.75	14041.5	10714	12910.5	11593	2435.75	9458.75	3036.25	0.736200803	0.133003713	8923	7886.85	Down	-0.178079635585573	Alanyl-tRNA editing protein Aarsd1 OS=Mus musculus GN=Aarsd1 PE=1 SV=2
PSMC3	O88685	24	24	43784.96	46193	37178.67	44466.46	44458.71	47563.21	47426.46	38092.79	51511.54	21429.12	0.736638532	0.132745568	43216.36	41204.624	Down	-0.0687713165875899	26S protease regulatory subunit 6A OS=Mus musculus GN=Psmc3 PE=1 SV=2
DNPH1	Q80VJ3	1	1	1241	1620	NA	2134	983	2414	1140	NA	NA	NA	0.7367338	0.132689405	1494.5	1777	Up	0.249780784350584	2'-deoxynucleoside 5'-phosphate N-hydrolase 1 OS=Mus musculus GN=Dnph1 PE=2 SV=2
TENM4	Q3UHK6-3	2	2	NA	NA	NA	40726	13145	25621	16051	NA	NA	NA	0.736971833	0.13254911	26935.5	20836	Down	-0.370430503774912	Isoform 3 of Teneurin-4 OS=Mus musculus GN=Tenm4
DNAJC5	P60904	3	3	910.67	5587	1508.33	11815.33	2704	9138	9074.33	680	3215.33	NA	0.737105465	0.132470369	4505.066	5526.915	Up	0.294926183177971	DnaJ homolog subfamily C member 5 OS=Mus musculus GN=Dnajc5 PE=1 SV=1
PAFAH1B1	P63005	4	4	16940.5	21793.25	18824.75	24108.75	23123.25	21647	21253.5	17945	25966.25	NA	0.737288069	0.132362794	20958.1	21702.938	Up	0.0503823928581263	Platelet-activating factor acetylhydrolase IB subunit alpha OS=Mus musculus GN=Pafah1b1 PE=1 SV=2
ARHGAP5	P97393	1	1	NA	NA	NA	6240	1301	2638	2744	NA	NA	NA	0.737648374	0.132150611	3770.5	2691	Down	-0.486613458693317	Rho GTPase-activating protein 5 OS=Mus musculus GN=Arhgap5 PE=1 SV=2
ABCF1	Q6P542	6	6	14162	15615	12531.2	15904.4	16184.2	18680.8	16971.2	9526.4	17873	6889.2	0.737744656	0.132093928	14879.36	13988.12	Down	-0.0891103960507015	ATP-binding cassette sub-family F member 1 OS=Mus musculus GN=Abcf1 PE=1 SV=1
SH3KBP1	Q8R550	3	3	44068.67	47079.67	35347.33	26737.67	49794	33618.33	38912.67	NA	58880.33	NA	0.738088049	0.131891827	40605.468	43803.777	Up	0.109381245016677	SH3 domain-containing kinase-binding protein 1 OS=Mus musculus GN=Sh3kbp1 PE=1 SV=1
MYH14	Q6URW6-3	19	19	3466.28	6853.06	3162.72	31204.28	9303	16156.56	13560.44	2498.39	7299.78	4220.39	0.738332822	0.131747825	10797.868	8747.112	Down	-0.303867813931511	Isoform 3 of Myosin-14 OS=Mus musculus GN=Myh14
BAG6	Q9Z1R2	4	4	11525.25	28367.75	9048.75	32612.75	28750	38184.25	32070.25	1424	29992.5	NA	0.738892441	0.131418776	22060.9	25417.75	Up	0.204344678903856	Large proline-rich protein BAG6 OS=Mus musculus GN=Bag6 PE=1 SV=1
LSM2	O35900	1	1	4092	2740	1919	5508	3200	4237	3375	3288	2001	NA	0.738938286	0.131391831	3491.8	3225.25	Down	-0.114559934992855	U6 snRNA-associated Sm-like protein LSm2 OS=Mus musculus GN=Lsm2 PE=3 SV=1
SLC9A7	Q8BLV3	2	2	7667	24513.5	6278	55692.5	35101.5	40573	50572.5	4598	26491.5	NA	0.73912812	0.131280275	25850.5	30558.75	Up	0.241393346010713	Sodium/hydrogen exchanger 7 OS=Mus musculus GN=Slc9a7 PE=2 SV=1
MRPL27	Q99N92	3	3	25820.33	22457.33	24745	17344.67	23523.33	22745.33	24381.67	26500	37843	10527	0.739280105	0.131190981	22778.132	24399.4	Up	0.0991962325758179	39S ribosomal protein L27, mitochondrial OS=Mus musculus GN=Mrpl27 PE=2 SV=1
DNAJB4	Q9D832	1	1	79730	70057	40888	74960	72218	75355	93359	73511	97590	23758	0.740272366	0.130608463	67570.6	72714.6	Up	0.105849399000659	DnaJ homolog subfamily B member 4 OS=Mus musculus GN=Dnajb4 PE=2 SV=1
ARPP21	Q9DCB4-3	3	3	93325.67	83893	63692.33	40397	85027	66025.67	79441.33	44831.67	118614.67	83891	0.740635587	0.130395424	73267	78560.868	Up	0.10064732561857	Isoform 3 of cAMP-regulated phosphoprotein 21 OS=Mus musculus GN=Arpp21
METTL26	Q9DCS2	1	1	2554	3246	1986	4471	2304	3353	2759	NA	2014	NA	0.741282922	0.130016005	2912.2	2708.6667	Down	-0.104526574484098	UPF0585 protein C16orf13 homolog OS=Mus musculus PE=1 SV=1
THTPA	Q8JZL3	1	1	5645	6173	6466	3142	4043	4472	4533	4161	6018	7911	0.741329252	0.129988863	5093.8	5419	Up	0.0892843315386536	Thiamine-triphosphatase OS=Mus musculus GN=Thtpa PE=1 SV=3
PYCR2	Q922Q4	6	6	58059.67	56098	85689	39821.5	49684	49382.33	58068	43048.33	76499.17	80497.67	0.741355348	0.129973575	57870.434	61499.1	Up	0.087738834029879	Pyrroline-5-carboxylate reductase 2 OS=Mus musculus GN=Pycr2 PE=2 SV=1

RPL15	Q9CZM2	5	5	103890.8	139961	135253.4	165051.4	120024	147812.4	134303.8	167640.2	120398.4	57107.2	0.741393462	0.129951248	132836.12	125452.4	Down	-0.0825074181338383	60S ribosomal protein L15 OS=Mus musculus GN=Rpl15 PE=2 SV=4
EMC8	O70378	4	4	38823.75	45729.25	43228.75	72699.75	46894.5	52950.5	55613.5	38299.5	34488	53355.75	0.741608643	0.129825217	49475.2	46941.45	Down	-0.0758431325355545	ER membrane protein complex subunit 8 OS=Mus musculus GN=Emc8 PE=1 SV=1
GLRX3	Q9CQM9	2	2	189832	209689	157077	204287	251294	235857	252847	100408	183434	186939	0.741912579	0.129647265	202435.8	191897	Down	-0.0771322902162094	Glutaredoxin-3 OS=Mus musculus GN=Glrx3 PE=1 SV=1
GNB5	P62881	4	4	13761	16919	10475	25230.75	14190.75	23961.25	17625.75	10500.5	17476	NA	0.74196003	0.12961949	16115.3	17390.875	Up	0.10989947621563	Guanine nucleotide-binding protein subunit beta-5 OS=Mus musculus GN=Gnb5 PE=1 SV=1
PRRT2	E9PUL5	5	5	2783.2	9063.6	2734	54023.6	9231	24691	21695.8	3260.8	3655	5900.2	0.742032816	0.129576888	15567.08	11840.56	Down	-0.394761040881734	Proline-rich transmembrane protein 2 OS=Mus musculus GN=Prrt2 PE=1 SV=1
GNAL	Q8CGK7	1	1	246457	333187	230828	564481	351506	486117	595600	361891	309273	140600	0.74216768	0.129497962	345291.8	378696.2	Up	0.133224871241405	Guanine nucleotide-binding protein G(olf) subunit alpha OS=Mus musculus GN=Gnal PE=1 SV=1
RAE1	Q8C570	1	1	13653	14067	NA	13428	13763	14355	12159	NA	NA	NA	0.742213837	0.129470954	13727.75	13257	Down	-0.0503408479266602	mRNA export factor OS=Mus musculus GN=Rae1 PE=1 SV=1
CPNE5	Q8JZW4	2	2	23101	24478.5	25696.5	25250	28182	26604.5	30449.5	29741	27285.5	3695.5	0.742251142	0.129449126	25341.6	23555.2	Down	-0.105462033672235	Copine-5 OS=Mus musculus GN=Cpne5 PE=2 SV=1
LIMD2	Q8BGB5	1	1	22469	21761	6186	32663	19032	18890	17706	NA	30861	NA	0.742862315	0.129091673	20422.2	22485.667	Up	0.138867368191238	LIM domain-containing protein 2 OS=Mus musculus GN=Limd2 PE=2 SV=1
SLC25A5	P51881	17	17	110053.47	184773.76	102013.94	311737.41	159929.82	233578.82	221442.88	95800.18	136459.41	99778.41	0.743389912	0.128783337	173701.68	157411.94	Down	-0.142066731303844	ADP/ATP translocase 2 OS=Mus musculus GN=Slc25a5 PE=1 SV=3
PRRC2C	Q3TLH4	6	6	19173.33	21597.17	19874.5	21182.5	23226.33	23116.5	24552.5	11699.33	29021.5	10043	0.744162155	0.12833242	21010.766	19686.566	Down	-0.0939172814325013	Protein PRRC2C OS=Mus musculus GN=Prrc2c PE=1 SV=3
SDF4	Q61112	2	2	91882	123674.5	47445.5	103598	98890.5	105104.5	118311	48208.5	131229.5	NA	0.744173466	0.128325819	93098.1	100713.38	Up	0.113431659907868	45 kDa calcium-binding protein OS=Mus musculus GN=Sdf4 PE=2 SV=1
ANK1	Q02357-3	5	5	7191.4	11284.8	2027.4	33732	13508.2	24146.6	21726	6156.2	11509.4	NA	0.744179815	0.128322114	13548.76	15884.55	Up	0.229463399615668	Isoform Er3 of Ankyrin-1 OS=Mus musculus GN=Ank1
MAOB	Q8BW75	1	1	31222	34118	73081	64781	35915	61221	42855	NA	25306	NA	0.744252236	0.128279852	47823.4	43127.333	Down	-0.1491141884316	Amine oxidase [flavin-containing] B OS=Mus musculus GN=Maob PE=1 SV=4
MRPL9	Q99N94	1	1	32352	25780	55911	31662	26345	25996	25078	42345	35091	57254	0.744302911	0.128250282	34410	37152.8	Up	0.110643050691443	39S ribosomal protein L9, mitochondrial OS=Mus musculus GN=Mrpl9 PE=2 SV=2
MRPS15	Q9DC71	3	3	18968.33	22770.67	19809	21394.67	23393.33	21430	25925	15575.67	19244.67	20952.67	0.744318347	0.128241276	21267.2	20625.602	Down	-0.0441938753033125	28S ribosomal protein S15, mitochondrial OS=Mus musculus GN=Mrps15 PE=2 SV=2
PAFAH1B1	P63005-2	2	2	12003.5	16115.5	9746.5	11786	13121	13845	13884	2570.5	27221.5	NA	0.744557864	0.128101545	12554.5	14380.25	Up	0.195884184718003	Isoform 2 of Platelet-activating factor acetylhydrolase IB subunit alpha OS=Mus musculus GN=Pafah1b1
ARHGAP12	Q8C0D4	1	1	NA	NA	NA	3672	1295	2909	1032	NA	1967	NA	0.744588464	0.128083697	2483.5	1969.3333	Down	-0.334667421393605	Rho GTPase-activating protein 12 OS=Mus musculus GN=Arhgap12 PE=1 SV=2
PHYHIP	Q8K0S0	3	3	13362.33	17259.33	5903.33	25328.67	18021.67	22302.33	21699	6278	16260.33	5138.67	0.744761149	0.127982987	15975.066	14335.666	Down	-0.156212961369751	Phytanoyl-CoA hydroxylase-interacting protein OS=Mus musculus GN=Phyhip PE=1 SV=1
DIP2B	Q3UH60	10	10	12502.22	11480.67	12663.67	15604.22	13946.89	17106.11	18636.22	5277.89	17085.67	1922.78	0.744767506	0.12797928	13239.534	12005.734	Down	-0.141128734651229	Disco-interacting protein 2 homolog B OS=Mus musculus GN=Dip2b PE=1 SV=1
KIF1B	Q60575-3	2	2	1973.5	10518.5	NA	20136	12809.5	16372.5	17072.5	NA	5976	NA	0.745480489	0.127563718	11359.375	13140.333	Up	0.210118414211526	Isoform 3 of Kinesin-like protein KIF1B OS=Mus musculus GN=Kif1b
PLPPR2	Q8VCY8-2	1	1	28992	48206	40963	85401	44830	60789	70855	51640	32347	NA	0.745862883	0.127341005	49678.4	53907.75	Up	0.117873986462899	Isoform 2 of Lipid phosphate phosphatase-related protein type 2 OS=Mus musculus GN=Lppr2
DNM3	Q8BZ98	18	18	50563.67	67742.28	45000.83	94190.5	61457.28	74897.78	79370	57538.39	49441.39	38431.5	0.746549982	0.126941111	63790.912	59935.812	Down	-0.0899326250384719	Dynamin-3 OS=Mus musculus GN=Dnm3 PE=1 SV=1
MARK1	Q8VHJ5	6	6	10748.33	11216.33	10954.17	15092.83	14445.83	16225.17	13460.33	15309	14431	6391.83	0.746805203	0.126792665	12491.498	13163.466	Up	0.0755929096382018	Serine/threonine-protein kinase MARK1 OS=Mus musculus GN=Mark1 PE=1 SV=2
ARMC1	Q9D7A8	2	2	65998	73403.5	98832.5	44941	69374.5	71499.5	70274	81169	77265	67682	0.747092119	0.126625845	70509.9	73577.9	Up	0.0614466661745811	Armadillo repeat-containing protein 1 OS=Mus musculus GN=Armc1 PE=1 SV=1
PDIA6	Q922R8	6	6	40881.17	51238.33	49175.5	85669.17	38671.83	59530	47404.5	71633.67	45689.17	57674.83	0.747528084	0.126372487	53127.2	56386.434	Up	0.0858974272343396	Protein disulfide-isomerase A6 OS=Mus musculus GN=Pdia6 PE=1 SV=3
KCNQ2	Q9Z351-13	2	2	2923	4403	3940.5	4141.5	6555.5	4440	5464.5	2166.5	7130	NA	0.747632071	0.126312077	4392.7	4800.25	Up	0.12800157048835	Isoform 13 of Potassium voltage-gated channel subfamily KQT member 2 OS=Mus musculus GN=Kcnq2
AUH	Q9JLZ3	15	15	166261.53	139387.27	153460.4	136451.2	142109.73	159052.53	145077.53	162452.93	135514.53	147810.33	0.747800077	0.126214494	147534.03	149981.57	Up	0.0237375068676868	Methylglutaconyl-CoA hydratase, mitochondrial OS=Mus musculus GN=Auh PE=1 SV=1
SGIP1	Q8VD37-5	2	2	485	2023.5	NA	15812	1505.5	6125	3539.5	NA	1165.5	NA	0.748157598	0.126006909	4956.5	3610	Down	-0.457322893371578	Isoform 5 of SH3-containing GRB2-like protein 3-interacting protein 1 OS=Mus musculus GN=Sgip1
ATIC	Q9CWJ9	8	8	9811.12	10141.62	3839.62	19924.5	10666.5	14387.25	13341.5	4082.25	10589.88	6583.12	0.748300175	0.125924153	10876.672	9796.8	Down	-0.15085470081157	Bifunctional purine biosynthesis protein PURH OS=Mus musculus GN=Atic PE=1 SV=2
LRP1B	Q9JI18	4	4	1332.5	1603.25	1793	6077	2679.5	4299.25	3020.25	NA	1897	NA	0.74846771	0.125826931	2697.05	3072.1667	Up	0.187874217532701	Low-density lipoprotein receptor-related protein 1B OS=Mus musculus GN=Lrp1b PE=1 SV=1
HINT1	P70349	6	6	172274.5	164168.33	176967.83	114695.17	167810.5	150129	177911	171321.33	187881.5	67515.17	0.748573333	0.125765648	159183.27	150951.6	Down	-0.0766026331440228	Histidine triad nucleotide-binding protein 1 OS=Mus musculus GN=Hint1 PE=1 SV=3
LNPEP	Q8C129	2	2	NA	8247	3720	22679.5	8406.5	11907.5	15452	NA	9530	NA	0.748656368	0.125717477	10763.25	12296.5	Up	0.192133963807517	Leucyl-cystinyl aminopeptidase OS=Mus musculus GN=Lnpep PE=1 SV=1
CTNND1	P30999-3	20	20	16507.59	17405.18	11347.94	25285.76	19911.24	24358.41	19868.41	9488.82	19166.59	11640.71	0.748746141	0.125665403	18091.542	16904.588	Down	-0.0979005235350357	Isoform 3 of Catenin delta-1 OS=Mus musculus GN=Ctnnd1
GRASP	Q9JJA9	1	1	33215	28882	23543	9493	36149	21845	23472	23339	46473	NA	0.749049022	0.125489759	26256.4	28782.25	Up	0.132510253847712	General receptor for phosphoinositides 1-associated scaffold protein OS=Mus musculus GN=Grasp PE=1 SV=2
PCBD1	P61458	1	1	4981	7069	11387	9528	4693	7926	6812	6487	7183	11968	0.749171486	0.12541876	7531.6	8075.2	Up	0.100541610668353	Pterin-4-alpha-carbinolamine dehydratase OS=Mus musculus GN=Pcbd1 PE=1 SV=2
NUCB1	Q02819	12	12	20880	28411.5	11445.17	39104.75	21600.17	31292.83	24889	2394.33	27864	NA	0.749401482	0.125285452	24288.318	21610.04	Down	-0.168560842167038	Nucleobindin-1 OS=Mus musculus GN=Nucb1 PE=1 SV=2
ATP2A3	Q64518-3	8	8	14886.25	29927.88	5228.25	95098.62	21082.62	54028.25	44778.12	7433.12	19438.5	9602.12	0.749416406	0.125276804	33244.724	27056.022	Down	-0.297175662417083	Isoform SERCA3C of Sarcoplasmic/endoplasmic reticulum calcium ATPase 3 OS=Mus musculus GN=Atp2a3
NSMF	Q99NF2-3	3	3	15064.67	15017	12753.33	15124.33	22067	19559	20055	8244.67	20729.33	NA	0.749560875	0.125193091	16005.266	17147	Up	0.0994095338467956	Isoform 3 of NMDA receptor synaptonuclear signaling and neuronal migration factor OS=Mus musculus GN=Nsmf
CDV3	Q4VAA2	7	7	79884.43	65597.57	65725.86	49945.29	63628.71	61565.29	61695.71	63955.71	98839	14844.29	0.749864733	0.125017071	64956.372	60180	Down	-0.110186949439042	Protein CDV3 OS=Mus musculus GN=Cdv3 PE=1 SV=2
ABLIM3	Q69ZX8	4	4	28825.75	28422.5	12910	26965	36368	32079.25	34762.25	5020.75	40169.5	7714.5	0.750070731	0.124897781	26698.25	23949.25	Down	-0.15676470323765	Actin-binding LIM protein 3 OS=Mus musculus GN=Ablim3 PE=1 SV=2
THY1	P01831	16	16	28614.44	47787.38	39694.31	71656.12	51375.75	64483.19	74112.12	34279.88	48324.5	35418.56	0.750671443	0.124550105	47825.6	51323.65	Up	0.101840706534341	Thy-1 membrane glycoprotein OS=Mus musculus GN=Thy1 PE=1 SV=1
CNDP2	Q9D1A2	7	7	15367.29	19957.57	19034	23709.14	19305	23330.71	21874.57	17947.14	23656.29	3990	0.750674892	0.12454811	19474.6	18159.742	Down	-0.100849990203931	Cytosolic non-specific dipeptidase OS=Mus musculus GN=Cndp2 PE=1 SV=1
A230050P20RIK	Q8CAK3	3	3	67421.67	52069	110086.67	37704.33	57157.67	57613.67	56107	153656.33	100323.33	3317.33	0.750698031	0.124534723	64887.868	74203.532	Up	0.19353909442249	UPF0515 protein C19orf66 homolog OS=Mus musculus PE=2 SV=1
DPYSL5	Q9EQF6	53	53	68720.5	76602.12	70819.18	73225.82	70218.1	71326.66	86350.12	65427.86	88563.84	58110.16	0.752095017	0.123727289	71917.144	73955.728	Up	0.0403261617412129	Dihydropyrimidinase-related protein 5 OS=Mus musculus GN=Dpysl5 PE=1 SV=1
NECAB2	Q91ZP9-2	2	2	14573	13036	1934	20196	11635.5	22666	12775.5	6083.5	22341.5	5356.5	0.752128346	0.123708044	12274.9	13844.6	Up	0.173612100396866	Isoform 2 of N-terminal EF-hand calcium-binding protein 2 OS=Mus musculus GN=Necab2
PPP1R1A	Q9ERT9	5	5	117750	104250.33	125806	81710.33	97686	98139	101500.33	158710.33	121712.33	2024	0.753182578	0.123099734	105440.53	96417.198	Down	-0.12906714559298	Protein phosphatase 1 regulatory subunit 1A OS=Mus musculus GN=Ppp1r1a PE=2 SV=1
IRS2	P81122	1	1	18298	19566	33124	22318	26920	21758	19860	27093	23375	NA	0.753266782	0.123051184	24045.2	23021.5	Down	-0.0627670886532273	Insulin receptor substrate 2 OS=Mus musculus GN=Irs2 PE=1 SV=2
HIST1H2BA	P70696	16	16	595914.88	249428.12	371765	93618.88	333763.88	175980.38	194156.69	409147.75	216392.94	480656	0.753363568	0.122995386	328898.15	295266.75	Down	-0.155621988590869	Histone H2B type 1-A OS=Mus musculus GN=Hist1h2ba PE=1 SV=3
RPSA	P14206	6	6	24332	33805.5	33024.33	40146.5	33433.33	35827.5	38482.33	14856.17	34622.17	NA	0.753655545	0.122827101	32948.332	30947.043	Down	-0.0904038883165125	40S ribosomal protein SA OS=Mus musculus GN=Rpsa PE=1 SV=4
DNM1L	Q8K1M6-3	13	13	21118.83	28621.25	24274.42	48976.58	28989.5	34891.08	37648.58	21886.58	26544.92	40749.58	0.754905199	0.122107583	30396.116	32344.148	Up	0.0896177215869044	Isoform 3 of Dynamin-1-like protein OS=Mus musculus GN=Dnm1l
ENSA	P60840-2	10	10	265359.6	224592.2	210451.4	78648.2	230563.4	164512.2	202960	195297.4	306768.3	57093.2	0.755040701	0.122029637	201922.96	185326.22	Down	-0.123737955474603	Isoform 2 of Alpha-endosulfine OS=Mus musculus GN=Ensa
TPM1	P58771	30	30	39306.04	39011.46	26700.96	39651.96	37773.61	39698.79	41813.86	24338.86	52480.86	11593.11	0.755054599	0.122021643	36488.806	33985.096	Down	-0.102551745203416	Tropomyosin alpha-1 chain OS=Mus musculus GN=Tpm1 PE=1 SV=1
TMEM63C	Q8CBX0	3	3	1201	5190	NA	8252.5	5018	5691.5	8200.5	NA	2935	NA	0.755069109	0.122013297	4915.375	5609	Up	0.190442097277316	Transmembrane protein 63C OS=Mus musculus GN=Tmem63c PE=2 SV=1
LZTS3	A2AHG0	10	10	24615.57	32394.57	33903	15041.29	30357.14	24955.14	28253.86	27272.57	36339.57	10798.14	0.755595659	0.121710546	27262.314	25523.856	Down	-0.095061722174995	Leucine zipper putative tumor suppressor 3 OS=Mus musculus GN=Lzts3 PE=2 SV=1
HEBP1	Q9R257	12	12	62094.33	61263.25	57355.92	66024	59930.58	87462.42	61910.67	66859.83	62452.92	40580.58	0.755705992	0.121647134	61333.616	63853.284	Up	0.0580828108450003	Heme-binding protein 1 OS=Mus musculus GN=Hebp1 PE=1 SV=2
DNAJB5	O89114	4	4	22925.33	21534.33	18802.67	23661.67	20828.33	19601	26557.33	28251.67	21349	5054.33	0.75572384	0.121636877	21550.466	20162.666	Down	-0.0960326548088487	DnaJ homolog subfamily B member 5 OS=Mus musculus GN=Dnajb5 PE=2 SV=1
NEDD4	P46935	3	3	8413.67	11636.33	2379.33	18998	13105.67	12769	18806.33	NA	5785.67	NA	0.755771339	0.121609582	10906.6	12453.667	Up	0.191369140741694	E3 ubiquitin-protein ligase NEDD4 OS=Mus musculus GN=Nedd4 PE=1 SV=3
PSMB1	O09061	3	3	43851.33	34801.33	42055.67	68516	46194.33	59900	49254.33	34987	62881.33	10303	0.756325756	0.12129111	47083.732	43465.132	Down	-0.11537015134643	Proteasome subunit beta type-1 OS=Mus musculus GN=Psmb1 PE=1 SV=1
CHMP1A	Q921W0	2	2	112892	104566	58349	143064	123961.5	112974.5	115574.5	70613.5	112204	NA	0.75662599	0.121118745	108566.5	102841.63	Down	-0.0781546927689794	Charged multivesicular body protein 1a OS=Mus musculus GN=Chmp1a PE=1 SV=1
DNAJB1	Q9QYJ3	1	1	11420	14782	4281	9640	15291	13510	12142	5018	18019	NA	0.756640784	0.121110254	11082.8	12172.25	Up	0.13527345478735	DnaJ homolog subfamily B member 1 OS=Mus musculus GN=Dnajb1 PE=2 SV=3
CXADR	P97792-2	9	9	3674.44	8505.22	2281.56	13381.56	7432.11	11125.67	12695	2136.22	10731.89	3292	0.756792254	0.121023322	7054.978	7996.156	Up	0.180665034455477	Isoform 2 of Coxsackievirus and adenovirus receptor homolog OS=Mus musculus GN=Cxadr
2900011O08RIK	Q8R1Y2	1	1	537	1659	NA	9548	160	3525	1170	NA	1906	NA	0.756868125	0.120979784	2976	2200.3333	Down	-0.435652429096487	Uncharacterized protein C16orf45 homolog OS=Mus musculus PE=2 SV=1
CPEB3	Q7TN99-3	2	2	7164.5	16692.5	2789	20728.5	13976.5	20202	15564	6075.5	12874	NA	0.757327658	0.120716182	12270.2	13678.875	Up	0.156790818040105	Isoform 3 of Cytoplasmic polyadenylation element-binding protein 3 OS=Mus musculus GN=Cpeb3
TTYH3	Q6P5F7-2	1	1	NA	971	NA	4080	NA	2686	784	NA	NA	6427	0.757697573	0.120504104	2525.5	3299	Up	0.385459736133728	Isoform 2 of Protein tweety homolog 3 OS=Mus musculus GN=Ttyh3
SNRNP70	Q62376	6	6	60854.67	52903.17	37494	37408.17	46301.5	41632	43577.17	61736.33	39656.33	58682.67	0.757739051	0.12048033	46992.302	49056.9	Up	0.0620316276906761	U1 small nuclear ribonucleoprotein 70 kDa OS=Mus musculus GN=Snrnp70 PE=1 SV=2
EEF1D	P57776-2	12	12	65506.17	58338.17	91311.08	38480.58	54925.08	52040.42	56208	71613.33	70065.67	74305.42	0.757972763	0.1203464	61712.216	64846.568	Up	0.0714741212748456	Isoform 2 of Elongation factor 1-delta OS=Mus musculus GN=Eef1d
GOLGA5	Q9QYE6	2	2	1017	7445	1517.5	31573	6335.5	17794	14104.5	8074.5	6396.5	NA	0.758301771	0.12015793	9577.6	11592.375	Up	0.275440081226531	Golgin subfamily A member 5 OS=Mus musculus GN=Golga5 PE=1 SV=2
RBMX	Q9WV02-2	2	2	48557	47507.5	25089	37555	38158.5	39208.5	36647.5	21405	50719	NA	0.758535083	0.120024328	39373.4	36995	Down	-0.0898909997321106	Isoform 2 of RNA-binding motif protein, X chromosome OS=Mus musculus GN=Rbmx
TCERG1	Q8CGF7	2	2	30874.5	28707.5	1319	21226.5	26067	33184.5	25276	NA	36067	3676.5	0.759061321	0.119723138	21638.9	24551	Up	0.182154626400109	Transcription elongation regulator 1 OS=Mus musculus GN=Tcerg1 PE=1 SV=2
BCL2L13	P59017	4	4	4374	4995.5	730	12758.75	9105.25	7879.25	4559.5	NA	4469.25	NA	0.759652092	0.119385262	6392.7	5636	Down	-0.181753780228379	Bcl-2-like protein 13 OS=Mus musculus GN=Bcl2l13 PE=1 SV=2
SUOX	Q8R086	4	4	25962.75	22105.5	9455.25	26395.5	17667.75	24743.25	25252.25	11461.5	25782.25	6123.5	0.759652178	0.119385213	20317.35	18672.55	Down	-0.121793281726337	Sulfite oxidase, mitochondrial OS=Mus musculus GN=Suox PE=2 SV=2
CAML	P49070	2	2	1132.5	NA	NA	4029.5	1579	3175	3274	NA	1374	NA	0.759769952	0.119317886	2247	2607.6667	Up	0.21475933972838	Calcium signal-modulating cyclophilin ligand OS=Mus musculus GN=Camlg PE=1 SV=2
CAMK1D	Q8BW96-3	3	3	23754	51998.5	NA	57415	40920	51391.5	58057.5	NA	31665	NA	0.760177317	0.119085094	43521.875	47038	Up	0.112086007992723	Isoform 3 of Calcium/calmodulin-dependent protein kinase type 1D OS=Mus musculus GN=Camk1d
COA6	Q8BGD8	3	3	31538.33	30532.33	17081	19168	27806.33	25110	24024.67	5415.67	37024.67	NA	0.760660907	0.118808903	25225.198	22893.753	Down	-0.139911640037591	Cytochrome c oxidase assembly factor 6 homolog OS=Mus musculus GN=Coa6 PE=3 SV=1
GRIA2	P23819	9	9	18927.67	28743.11	24328.78	46460.33	24033.33	37584.89	38239.22	25093.67	24048.33	26543.67	0.760725994	0.118771743	28498.644	30301.956	Up	0.0885176473520851	Glutamate receptor 2 OS=Mus musculus GN=Gria2 PE=1 SV=3
PDE2A	Q922S4	1	1	NA	2627	NA	24526	1890	9103	4987	NA	NA	NA	0.761148133	0.118530814	9681	7045	Down	-0.4585563720111	cGMP-dependent 3',5'-cyclic phosphodiesterase OS=Mus musculus GN=Pde2a PE=1 SV=3
DLGAP2	Q8BJ42-2	19	19	30972.42	36472.53	31059.79	28751.84	41492.32	34051.11	37538.89	28313.26	44464.68	30187.26	0.761274175	0.118458903	33749.78	34911.04	Up	0.0488052375225191	Isoform 2 of Disks large-associated protein 2 OS=Mus musculus GN=Dlgap2
PSMB4	P99026	1	1	2930	2789	NA	7016	4861	6957	4030	NA	3608	NA	0.761296323	0.118446268	4399	4865	Up	0.14526420375835	Proteasome subunit beta type-4 OS=Mus musculus GN=Psmb4 PE=1 SV=1
EHBP1	Q69ZW3	1	1	2174	4516	NA	8529	2340	6779	3555	NA	4534	NA	0.761310973	0.118437911	4389.75	4956	Up	0.175037408089263	EH domain-binding protein 1 OS=Mus musculus GN=Ehbp1 PE=1 SV=3
PTGDS	O09114	2	2	42046	35204	52136	34716	32146	36806	34952	22939	68246	46681	0.761478104	0.11834258	39249.6	41924.8	Up	0.0951259502023342	Prostaglandin-H2 D-isomerase OS=Mus musculus GN=Ptgds PE=1 SV=1
CPSF7	Q8BTV2-3	1	1	7222	NA	NA	7820	4715	4916	10450	NA	NA	NA	0.7615961	0.118275289	6585.6667	7683	Up	0.222340261844304	Isoform 3 of Cleavage and polyadenylation specificity factor subunit 7 OS=Mus musculus GN=Cpsf7
CBR3	Q8K354	4	4	55607.25	60611	85117	24405.5	49910.75	48197.75	49023.25	50975.25	71738.25	73590	0.761814361	0.118150844	55130.3	58704.9	Up	0.090635475610644	Carbonyl reductase [NADPH] 3 OS=Mus musculus GN=Cbr3 PE=2 SV=1
D10JHU81E	Q9D172	27	27	133338.73	119712.19	91490.35	200290.65	142226.92	156793.5	137446.5	137319.42	152487.08	64542.42	0.761820539	0.118147323	137411.77	129717.78	Down	-0.0831292792284633	ES1 protein homolog, mitochondrial OS=Mus musculus GN=D10Jhu81e PE=1 SV=1
CACNB1	Q8R3Z5-3	5	5	44747.8	47212.6	42945.4	24007.2	46810.2	43562.8	46805.4	34419.4	50941.2	38270	0.76197409	0.118059796	41144.64	42799.76	Up	0.0568982067970464	Isoform 3 of Voltage-dependent L-type calcium channel subunit beta-1 OS=Mus musculus GN=Cacnb1
SPAG9	Q58A65-3	3	3	14608	27735.5	NA	44632.5	31733.5	46567.5	37063	NA	16058	NA	0.762429632	0.117800233	29677.375	33229.5	Up	0.163101095680386	Isoform 3 of C-Jun-amino-terminal kinase-interacting protein 4 OS=Mus musculus GN=Spag9
ACADM	P45952	3	3	104627	122159.33	113674	152145.67	96950.33	149223	118186.33	116661.33	112816	67236.33	0.762772785	0.11760481	117911.27	112824.6	Down	-0.0636199310967476	Medium-chain specific acyl-CoA dehydrogenase, mitochondrial OS=Mus musculus GN=Acadm PE=1 SV=1
MYL12B	Q3THE2	7	7	13598.5	15543.5	373	31813.75	15359.25	20481	18481.25	12585.5	16522.75	NA	0.763025777	0.11746079	15337.6	17017.625	Up	0.149956956653283	Myosin regulatory light chain 12B OS=Mus musculus GN=Myl12b PE=1 SV=2
RTN3	Q9ES97-5	19	19	5503.94	15029.94	4736.29	50381.65	15309.29	27819.18	26383.76	8051.59	12790.71	388.53	0.763028244	0.117459386	18192.222	15086.754	Down	-0.270039329384928	Isoform 5 of Reticulon-3 OS=Mus musculus GN=Rtn3
CDR2L	A2A6T1	1	1	1784	NA	4589	9536	6059	5296	3877	NA	5655	NA	0.764568119	0.116583815	5492	4942.6667	Down	-0.152042011049101	Cerebellar degeneration-related protein 2-like OS=Mus musculus GN=Cdr2l PE=2 SV=1
SLC1A3	P56564	5	5	4651.8	12593	6062	34401	12918.2	20258.6	20567	8625.4	10093.8	968.6	0.764944324	0.116370173	14125.2	12102.68	Down	-0.222944743738578	Excitatory amino acid transporter 1 OS=Mus musculus GN=Slc1a3 PE=1 SV=2
TRF	Q921I1	15	15	80027.2	95527.73	76911.33	67174.2	83663.73	88843.13	103877.93	84573.73	85341.87	54988.33	0.765064678	0.116301848	80660.838	83524.998	Up	0.0503396487742777	Serotransferrin OS=Mus musculus GN=Tf PE=1 SV=1
CCS	Q9WU84	1	1	874	1685	NA	2497	1759	1932	2011	NA	1518	NA	0.765129691	0.116264945	1703.75	1820.3333	Up	0.0954889990605359	Copper chaperone for superoxide dismutase OS=Mus musculus GN=Ccs PE=2 SV=1
NECAB2	Q91ZP9	3	3	12807	13112.33	3949.67	19838.67	11524.33	20723.33	12842	6939	19326.67	7340.33	0.765364188	0.116131862	12246.4	13434.266	Up	0.133559788601883	N-terminal EF-hand calcium-binding protein 2 OS=Mus musculus GN=Necab2 PE=1 SV=1
AGPAT1	O35083	3	3	6335.67	9801.33	1876.67	26534.67	12803.33	19976	16685.33	5666	10107	NA	0.765418604	0.116100986	11470.334	13108.583	Up	0.192604286571364	1-acyl-sn-glycerol-3-phosphate acyltransferase alpha OS=Mus musculus GN=Agpat1 PE=2 SV=1
ANP32-PS	Q64G17	1	1	38876	67918	14607	222588	49169	104343	65380	NA	25523	NA	0.765819713	0.115873458	78631.6	65082	Down	-0.272850624343572	Putative acidic leucine-rich nuclear phosphoprotein 32 family member C OS=Mus musculus GN=Anp32c PE=2 SV=3
GLS	D3Z7P3	5	5	23598.8	31747.2	25982.2	46749.6	34144.4	38674.6	33548.2	37346	29912.2	29677	0.76666228	0.115395903	32444.44	33831.6	Up	0.0604001432923481	Glutaminase kidney isoform, mitochondrial OS=Mus musculus GN=Gls PE=1 SV=1
PRICKLE2	Q80Y24	22	22	35216.05	41403.8	25776.85	34758.2	50198.25	40729.05	47514.6	18107.9	51704.1	16166.5	0.766740807	0.115351423	37470.63	34844.43	Down	-0.104832179303075	Prickle-like protein 2 OS=Mus musculus GN=Prickle2 PE=1 SV=3
CLIP2	Q9Z0H8	22	22	27094.05	28138.82	16939.86	38731.82	31318.27	34280.68	34852.91	12154.77	40087.73	9619.23	0.767083667	0.115157264	28444.564	26199.064	Down	-0.118637697198779	CAP-Gly domain-containing linker protein 2 OS=Mus musculus GN=Clip2 PE=1 SV=2
PAICS	Q9DCL9	3	3	19051	28917.33	8284	93353	24958.67	57473.33	45970	21008	36154	NA	0.767181116	0.115102096	34912.8	40151.333	Up	0.201691801773947	Multifunctional protein ADE2 OS=Mus musculus GN=Paics PE=1 SV=4
AMER2	Q8CCJ4	7	7	50946.5	58984.25	38715.5	76093	58023.25	69522	68291	65095	76400	21330.75	0.767256971	0.115059157	56552.5	60127.75	Up	0.0884401709985107	APC membrane recruitment protein 2 OS=Mus musculus GN=Amer2 PE=1 SV=2
SLC4A8	Q8JZR6	2	2	NA	3429	NA	6663	2692	5145	4302	NA	4587	NA	0.76744498	0.114952751	4261.3333	4678	Up	0.134586954145607	Electroneutral sodium bicarbonate exchanger 1 OS=Mus musculus GN=Slc4a8 PE=2 SV=1

PRDX5	P99029	49	49	199162.69	175320.61	154277.12	189368.65	191433.78	215467.02	181311.96	162882.02	198803.94	123718.12	0.767625383	0.114850673	181912.57	176436.61	Down	-0.0440952727495749	Peroxiredoxin-5, mitochondrial OS=Mus musculus GN=Prdx5 PE=1 SV=2
BCLAF1	Q8K019-3	3	3	49524.33	35090.33	38311.67	43624	45863	45246.33	45474.67	17539.67	51466	NA	0.767870701	0.114711903	42482.666	39931.668	Down	-0.0893409868241626	Isoform 3 of Bcl-2-associated transcription factor 1 OS=Mus musculus GN=Bclaf1
LZTFL1	Q9JHQ5	2	2	48596.5	40611.5	39718.5	24127	43945	36586	38148.5	49477.5	48660.5	32310	0.767972705	0.114654215	39399.7	41036.5	Up	0.0587230441882292	Leucine zipper transcription factor-like protein 1 OS=Mus musculus GN=Lztfl1 PE=1 SV=1
GM996	A2AJA9-2	14	14	22808	26062.14	18754.93	25922.5	29138.43	30081.36	31454	23519.79	29817.21	13601.71	0.76823894	0.114503683	24537.2	25694.814	Up	0.0665065798506461	Isoform 2 of Uncharacterized protein C9orf172 homolog OS=Mus musculus GN=Gm996
MARCKSL1	P28667	25	25	17020	21888.23	18821.59	36466.05	15995.41	23811.32	22282.86	24716.09	29728.27	16283.23	0.768844625	0.114161418	22038.256	23364.354	Up	0.0842990881590143	MARCKS-related protein OS=Mus musculus GN=Marcksl1 PE=1 SV=2
TIMM8A1	Q9WVA2	5	5	57975.8	44108	54851.2	30493.8	42823.6	36935	50308	63301.8	50359.8	39611.4	0.769113416	0.114009613	46050.48	48103.2	Up	0.06291667641259	Mitochondrial import inner membrane translocase subunit Tim8 A OS=Mus musculus GN=Timm8a1 PE=1 SV=1
GNS	Q8BFR4	1	1	14789	14024	5235	19934	16579	19407	15722	5908	20473	NA	0.769320593	0.113892642	14112.2	15377.5	Up	0.123878063720048	N-acetylglucosamine-6-sulfatase OS=Mus musculus GN=Gns PE=2 SV=1
NUDT21	Q9CQF3	2	2	65938.5	49521	64813.5	17985.5	41193	28071.5	42582	27619.5	37693.5	83016	0.76944252	0.113823818	47890.3	43796.5	Down	-0.128917891730862	Cleavage and polyadenylation specificity factor subunit 5 OS=Mus musculus GN=Nudt21 PE=1 SV=1
CCDC177	Q3UHB8	10	10	36925	35666.8	27244.9	37315.7	40714.9	41586.3	40014.1	19177.2	53412.8	11108.4	0.769561685	0.113756563	35573.46	33059.76	Down	-0.105725051517786	Coiled-coil domain-containing protein 177 OS=Mus musculus GN=Ccdc177 PE=1 SV=1
DDX17	Q501J6	11	11	52251.82	38697.64	40479.64	29963.73	42821	35488.55	35896.55	48783.91	42681	48308.27	0.770659037	0.113137725	40842.766	42231.656	Up	0.0482442462768982	Probable ATP-dependent RNA helicase DDX17 OS=Mus musculus GN=Ddx17 PE=2 SV=1
DBN1	Q9QXS6	44	44	40037.45	43897.45	46830.19	39928.19	41289.38	37026.93	46398.52	41065.05	47474.07	43502.83	0.770694602	0.113117683	42396.532	43093.48	Up	0.023523349126461	Drebrin OS=Mus musculus GN=Dbn1 PE=1 SV=4
HUWE1	Q7TMY8-3	2	2	1857.5	4596.5	1813.5	13795	5985.5	12252.5	10042	3060	1279	NA	0.770763338	0.113078951	5609.6	6658.375	Up	0.247272224210288	Isoform 3 of E3 ubiquitin-protein ligase HUWE1 OS=Mus musculus GN=Huwe1
PRPF40A	Q9R1C7	2	2	14897	11889	7457	5740	12041	11310	7597	NA	10470	NA	0.770872992	0.11301717	10404.8	9792.3333	Down	-0.0875246603453404	Pre-mRNA-processing factor 40 homolog A OS=Mus musculus GN=Prpf40a PE=1 SV=1
ABI3	Q8BYZ1	1	1	26438	21018	34264	13952	27823	21233	27250	NA	29457	NA	0.771081281	0.11289984	24699	25980	Up	0.0729487988643445	ABI gene family member 3 OS=Mus musculus GN=Abi3 PE=2 SV=3
GPRIN3	Q8BWS5	5	5	19541.8	23315.6	11108	17054.8	20399.2	19699	21815.4	6066.4	20388.2	NA	0.771510537	0.112658138	18283.88	16992.25	Down	-0.105695358258779	G protein-regulated inducer of neurite outgrowth 3 OS=Mus musculus GN=Gprin3 PE=1 SV=1
NSFL1C	Q9CZ44-3	10	10	9209.3	7859.5	2561.3	16404.5	9860.8	13504.1	11466.5	2110.2	13935.4	NA	0.771687103	0.112558758	9179.08	10254.05	Up	0.159772369932633	Isoform 3 of NSFL1 cofactor p47 OS=Mus musculus GN=Nsfl1c
RAB1A	P62821	6	6	17006.33	23576	16521.83	27119.67	21974	26103	32004	13641.67	18043.67	9462	0.771695193	0.112554205	21239.566	19850.868	Down	-0.0975521949443514	Ras-related protein Rab-1A OS=Mus musculus GN=Rab1A PE=1 SV=3
SPTAN1	P16546	267	267	47768.42	61510.32	35172.62	96241.76	64435.39	73197.13	74873.73	41325.97	62165.2	33715.42	0.771800149	0.112495142	61025.702	57055.49	Down	-0.0970512735643918	Spectrin alpha chain, non-erythrocytic 1 OS=Mus musculus GN=Sptan1 PE=1 SV=4
1700021F05RIK	Q9CQF4	4	4	107397.75	98065.5	90095	47370.25	79910.5	72603.25	73099.75	101028.25	96567.75	60065.75	0.771814198	0.112487237	84567.8	80672.95	Down	-0.0680234354378277	Uncharacterized protein C6orf203 homolog OS=Mus musculus PE=1 SV=1
GRM5	Q3UVX5	5	5	14049.75	15490.75	4156.5	31139.25	16920.75	23245.25	21471.75	12636.5	14166.5	NA	0.772227318	0.112254839	16351.4	17880	Up	0.128932572566147	Metabotropic glutamate receptor 5 OS=Mus musculus GN=Grm5 PE=1 SV=2
NAV1	Q8CH77-4	4	4	18132.25	18688.25	11657.75	19050.5	22113.25	18920	26628.5	3551.5	27584	4586.25	0.772374001	0.112172353	17928.4	16254.05	Down	-0.1414475049101	Isoform 4 of Neuron navigator 1 OS=Mus musculus GN=Nav1
LSM7	Q9CQQ8	1	1	6964	4790	6181	5696	8338	8691	6340	3092	7355	8240	0.773136785	0.111743663	6393.8	6743.6	Up	0.0768453477548664	U6 snRNA-associated Sm-like protein LSm7 OS=Mus musculus GN=Lsm7 PE=3 SV=1
ARL15	Q8BGR6	2	2	23293	28459.5	22238.5	34851.5	32147.5	26994.5	30364	27538	39394.5	22281.5	0.773250454	0.111679817	28198	29314.5	Up	0.0560216095542159	ADP-ribosylation factor-like protein 15 OS=Mus musculus GN=Arl15 PE=2 SV=1
CDC42EP4	Q9JM96	12	12	38538.92	34889.92	41759.08	23845.75	34348	35933	35369.58	32060.08	47323	29118.42	0.774017412	0.111249269	34676.334	35960.816	Up	0.0524743737892615	Cdc42 effector protein 4 OS=Mus musculus GN=Cdc42ep4 PE=1 SV=1
CPEB4	Q7TN98-3	1	1	20576	26558	10168	31322	13707	20241	28829	NA	17077	NA	0.774171212	0.111162982	20466.2	22049	Up	0.10746996677999	Isoform 3 of Cytoplasmic polyadenylation element-binding protein 4 OS=Mus musculus GN=Cpeb4
DBN1	Q9QXS6-3	41	41	42636.87	46440.9	50432.51	42101.92	43659.51	38918.31	48886.1	44223.9	50129.31	46849.21	0.774530757	0.110961332	45054.342	45801.366	Up	0.0237244776523181	Isoform E2 of Drebrin OS=Mus musculus GN=Dbn1
EEF1D	P57776	16	16	57556.4	50095.4	78812.33	33411.33	47964.67	45472	48594.6	61943.8	62539	61727.2	0.774856473	0.110778735	53568.026	56055.32	Up	0.0654791670656221	Elongation factor 1-delta OS=Mus musculus GN=Eef1d PE=1 SV=3
CCT3	P80318	23	23	52836.52	73614.17	65966.17	95005.22	73332.43	82159.61	75605.26	72855.09	69665.96	71304	0.77561875	0.110351701	72150.902	74317.984	Up	0.042693935936742	T-complex protein 1 subunit gamma OS=Mus musculus GN=Cct3 PE=1 SV=1
CIRBP	P60824	6	6	61885.5	77676	27793	75124	47033	62280.67	72594.5	23634.33	166768.5	8196.33	0.775840538	0.110227532	57902.3	66694.866	Up	0.203955054508339	Cold-inducible RNA-binding protein OS=Mus musculus GN=Cirbp PE=1 SV=1
ANP32B	Q9EST5	1	1	5164	17614	NA	29613	18642	17561	18012	NA	22519	NA	0.776432537	0.109896273	17758.25	19364	Up	0.124887582078201	Acidic leucine-rich nuclear phosphoprotein 32 family member B OS=Mus musculus GN=Anp32b PE=1 SV=1
ADAM23	Q9R1V7	9	9	32795.22	40960.89	25596.89	42421.22	39408.67	44405	42432.56	34736.11	38147	11518.78	0.776512312	0.109851654	36236.578	34247.89	Down	-0.0814316140636909	Disintegrin and metalloproteinase domain-containing protein 23 OS=Mus musculus GN=Adam23 PE=1 SV=1
LZTS3	A2AHG0-2	9	9	17951.83	23178.33	18511.83	13628.67	22415.33	20291.33	19023.33	4611.83	26593.5	NA	0.776721223	0.109734828	19137.198	17629.998	Down	-0.118347340966456	Isoform 2 of Leucine zipper putative tumor suppressor 3 OS=Mus musculus GN=Lzts3
TLN1	P26039	15	15	20994.2	32682.6	26933.2	51021.93	41027.27	43735.4	45814.8	27141.47	38762.93	26964.53	0.77672186	0.109734472	34531.84	36483.826	Up	0.0793298215094855	Talin-1 OS=Mus musculus GN=Tln1 PE=1 SV=2
ACADL	P51174	7	7	61256.71	58014.71	71326.43	59587.71	60532.86	70386.71	59796.43	71982.43	61858.43	36589	0.776819708	0.109679765	62143.684	60122.6	Down	-0.0477003698925194	Long-chain specific acyl-CoA dehydrogenase, mitochondrial OS=Mus musculus GN=Acadl PE=1 SV=2
HCN4	O70507	1	1	2878	4416	NA	14184	8286	9508	12303	NA	3716	NA	0.77731557	0.109402633	7441	8509	Up	0.19349307364123	Potassium/sodium hyperpolarization-activated cyclic nucleotide-gated channel 4 OS=Mus musculus GN=Hcn4 PE=2 SV=2
M6PR	P24668	1	1	1277	5059	NA	25771	3656	11294	10098	NA	NA	NA	0.777702303	0.109186615	8940.75	10696	Up	0.25860360763605	Cation-dependent mannose-6-phosphate receptor OS=Mus musculus GN=M6pr PE=1 SV=1
S1PR1	O08530	1	1	4597	12519	9150	21128	13203	16596	24186	5312	8380	NA	0.777770149	0.109148729	12119.4	13618.5	Up	0.168249530984619	Sphingosine 1-phosphate receptor 1 OS=Mus musculus GN=S1pr1 PE=1 SV=3
CCT8	P42932	15	15	16991.2	30384.87	22381.53	47872.53	29719.53	40523.67	37773.33	20242.47	33364	2685.13	0.777867918	0.10909414	29469.932	26917.72	Down	-0.130687516389473	T-complex protein 1 subunit theta OS=Mus musculus GN=Cct8 PE=1 SV=3
LYPLA2	Q9WTL7	1	1	12546	27341	NA	30949	23788	21971	28454	NA	NA	NA	0.778553945	0.108711291	23656	25212.5	Up	0.091933030267362	Acyl-protein thioesterase 2 OS=Mus musculus GN=Lypla2 PE=1 SV=1
ADGRB2	Q8CGM1-3	8	8	22756.88	51521.12	21078.5	67824.5	60233.62	67710.25	90555	19648.62	69366.5	3977.75	0.778834868	0.108554613	44682.924	50251.624	Up	0.169446623903234	Isoform 3 of Brain-specific angiogenesis inhibitor 2 OS=Mus musculus GN=Bai2
PDIA4	P08003	11	11	52221	55347.09	76210.45	71062	51991.91	58070	55060.27	84156.55	51991.18	45363.45	0.779111386	0.108400449	61366.49	58928.29	Down	-0.0584906653063277	Protein disulfide-isomerase A4 OS=Mus musculus GN=Pdia4 PE=1 SV=3
SPTAN1	P16546-2	254	254	43448.19	57595.32	35164.06	91068.26	59034.9	67974.4	69338.24	42046.87	57004.21	32548.69	0.779288788	0.108301572	57262.146	53782.482	Down	-0.0904454034213868	Isoform 2 of Spectrin alpha chain, non-erythrocytic 1 OS=Mus musculus GN=Sptan1
SPTBN1	Q62261-2	143	143	17325.91	40774.52	16300.7	133141.85	41360.74	72301.18	70719.39	19475.89	32905.21	17373.98	0.779370157	0.108256228	49780.744	42555.13	Down	-0.226254733760862	Isoform 2 of Spectrin beta chain, non-erythrocytic 1 OS=Mus musculus GN=Sptbn1
MTIF3	Q9CZD5	3	3	18165	22042.67	10775.67	22298.67	18032.67	24754	17581	6539	30928.67	NA	0.779666975	0.108090861	18262.936	19950.668	Up	0.127518300659691	Translation initiation factor IF-3, mitochondrial OS=Mus musculus GN=Mtif3 PE=1 SV=1
SPEN	Q62504-3	1	1	4096	5836	NA	12040	8572	12179	3853	NA	9582	NA	0.779836366	0.107996517	7636	8538	Up	0.161081059843701	Isoform 3 of Msx2-interacting protein OS=Mus musculus GN=Spen
CCDC136	Q3TVA9	11	11	2628.18	6628.73	1206.73	20118	7752.82	13206.64	12064.09	5383.64	4676.36	NA	0.780050483	0.10787729	7666.892	8832.6825	Up	0.204209795842373	Coiled-coil domain-containing protein 136 OS=Mus musculus GN=Ccdc136 PE=2 SV=2
A830010M20RIK	Q80TK0	14	14	24075.69	21422.77	19270	25080.77	25210.38	28594.92	26412.85	16579.77	33499.92	15419.54	0.780099073	0.107850238	23011.922	24101.4	Up	0.0667354667718717	Uncharacterized protein KIAA1107 OS=Mus musculus GN=Kiaa1107 PE=2 SV=5
ACAA2	Q8BWT1	25	25	62553.6	62768.16	58077.08	56002.6	67880.44	67104.84	61623.84	75837.72	66865.8	44156.32	0.780163069	0.107814612	61456.376	63117.704	Up	0.0384820304871351	3-ketoacyl-CoA thiolase, mitochondrial OS=Mus musculus GN=Acaa2 PE=1 SV=3
GLS	D3Z7P3-2	6	6	19885.17	26727.33	21651.83	39424.33	29033.83	32784.33	28312.33	31121.67	25304.67	24730.83	0.780719043	0.107505228	27344.498	28450.766	Up	0.0572169189152035	Isoform 2 of Glutaminase kidney isoform, mitochondrial OS=Mus musculus GN=Gls
LSAMP	Q8BLK3	23	23	9973.57	22129.61	14326.7	64626.61	21781.57	38669.78	37620.96	12631.83	23385.91	3210.83	0.780793951	0.10746356	26567.612	23103.862	Down	-0.201534528524145	Limbic system-associated membrane protein OS=Mus musculus GN=Lsamp PE=1 SV=1
GOLGA2	Q921M4	5	5	7037.8	9159.6	391.4	14832.4	10112.6	10958.6	11435	NA	5223.8	NA	0.780932328	0.107386598	8306.76	9205.8	Up	0.14825722723273	Golgin subfamily A member 2 OS=Mus musculus GN=Golga2 PE=2 SV=3
SEC62	Q8BU14	1	1	12171	52382	NA	102704	40215	79850	70803	NA	26918	NA	0.781441194	0.107103699	51868	59190.333	Up	0.190516840125312	Translocation protein SEC62 OS=Mus musculus GN=Sec62 PE=1 SV=1
GNAS	Q6R0H7-4	5	5	59260.8	84024	63952.8	136124.6	87841.4	119843.4	142322.2	83419.4	78627	39139.8	0.782256698	0.10665071	86240.72	92670.36	Up	0.103738753384836	Isoform XLas-4 of Guanine nucleotide-binding protein G(s) subunit alpha isoforms XLas OS=Mus musculus GN=Gnas
SRSF1	Q6PDM2	3	3	33234.33	27729	1192.33	21431	30053	27803.33	25829.67	1254	26180.67	NA	0.782274411	0.106640876	22727.932	20266.918	Down	-0.165339741667004	Serine/arginine-rich splicing factor 1 OS=Mus musculus GN=Srsf1 PE=1 SV=3
KIRREL3	Q8BR86-3	2	2	NA	2398.5	NA	6289.5	2504.5	5142.5	5381.5	1746	597	NA	0.78246146	0.106537045	3730.8333	3216.75	Down	-0.213894095713038	Isoform 3 of Kin of IRRE-like protein 3 OS=Mus musculus GN=Kirrel3
SPRED1	Q924S8	1	1	4879	24919	13953	27627	25347	22505	25843	NA	14440	NA	0.782762039	0.106370244	19345	20929.333	Up	0.113565629061326	Sprouty-related, EVH1 domain-containing protein 1 OS=Mus musculus GN=Spred1 PE=1 SV=1
CADM2	Q8BLQ9-3	17	17	31790.38	42395.06	33109.62	78525.81	45295.25	62478.19	59227.81	38729.06	35637.12	18279.44	0.782794627	0.106352164	46223.224	42870.324	Down	-0.108638568496046	Isoform 3 of Cell adhesion molecule 2 OS=Mus musculus GN=Cadm2
AKAP12	Q9WTQ5	6	6	15302.6	17309.8	7722.4	26645.2	19173	21558.4	24170.6	8356.4	17978.4	7584	0.783099098	0.106183276	17230.6	15929.56	Down	-0.11326652177098	A-kinase anchor protein 12 OS=Mus musculus GN=Akap12 PE=1 SV=1
DRG1	P32233	2	2	3471	5963.5	NA	18476	9677.5	14268	12401.5	10319	5061	NA	0.783201416	0.106126536	9397	10512.375	Up	0.161816492033359	Developmentally-regulated GTP-binding protein 1 OS=Mus musculus GN=Drg1 PE=1 SV=1
PREP	Q9QUR6	2	2	1794	3616.5	3347.5	6585.5	5030	6655	5287	2905	3322	3727	0.783255939	0.106096304	4074.7	4379.2	Up	0.103973495059436	Prolyl endopeptidase OS=Mus musculus GN=Prep PE=2 SV=1
CHGB	P16014	3	3	5464.67	6750	12920.33	6178.67	7308.67	8162	7742.67	6117.67	13596.33	5760	0.783435516	0.105996744	7724.468	8275.734	Up	0.0994516995389924	Secretogranin-1 OS=Mus musculus GN=Chgb PE=1 SV=2
PPP1CB	P62141	2	2	18448	21073	8027.5	32533	16535.5	25156	17825.5	21511	17798	NA	0.783780644	0.105805466	19323.4	20572.625	Up	0.0903769265325957	Serine/threonine-protein phosphatase PP1-beta catalytic subunit OS=Mus musculus GN=Ppp1cb PE=1 SV=3
GMFG	Q9ERL7	1	1	97268	86291	62683	134023	115738	87090	96207	NA	102963	NA	0.78386289	0.105759896	99200.6	95420	Down	-0.0560571602416136	Glia maturation factor gamma OS=Mus musculus GN=Gmfg PE=1 SV=1
RIN1	Q921Q7	1	1	3742	4580	1413	12602	5912	8936	6407	2799	1577	NA	0.784032182	0.105666111	5649.8	4929.75	Down	-0.196685312002478	Ras and Rab interactor 1 OS=Mus musculus GN=Rin1 PE=1 SV=1
ABLIM2	Q8BL65	17	17	28020	26484.88	13390.44	30451.94	30984.25	30418.94	32696.81	16254.25	38203.62	1046.69	0.784052318	0.105654957	25866.302	23724.062	Down	-0.124722765305562	Actin-binding LIM protein 2 OS=Mus musculus GN=Ablim2 PE=1 SV=1
CCDC124	Q9D8X2	4	4	13989.5	17424.25	10453	15944.75	15277.5	19401.75	16672.75	1003.75	16264.25	NA	0.784075847	0.105641924	14617.8	13335.625	Down	-0.132440758759521	Coiled-coil domain-containing protein 124 OS=Mus musculus GN=Ccdc124 PE=2 SV=1
PMM2	Q9Z2M7	2	2	8543.5	8051.5	5052	11137.5	7521.5	10120.5	8342.5	3647.5	8196	NA	0.784582839	0.105361195	8061.2	7576.625	Down	-0.0894392716420983	Phosphomannomutase 2 OS=Mus musculus GN=Pmm2 PE=1 SV=1
NELL2	Q61220	8	8	1917.33	2402	1110.17	5894	4141.67	4439.67	5128.17	743.83	2427.33	988.83	0.785497878	0.104854984	3093.034	2745.566	Down	-0.171919099959631	Protein kinase C-binding protein NELL2 OS=Mus musculus GN=Nell2 PE=2 SV=3
VAMP2	P63044	6	6	25407.33	63381.33	25873.33	53308.83	66735	71022.17	108368.33	37221.67	45239.17	1284.67	0.785680073	0.104754262	46941.164	52627.202	Up	0.164955077124865	Vesicle-associated membrane protein 2 OS=Mus musculus GN=Vamp2 PE=1 SV=2
BAALC	Q8VHV1	2	2	30289	34196.5	35827.5	19607	32108.5	30411	35098	21103	40752	NA	0.786273517	0.10426352	30405.7	31841	Up	0.0665438423116121	Brain and acute leukemia cytoplasmic protein OS=Mus musculus GN=Baalc PE=2 SV=3
WBP11	Q923D5	1	1	39191	26017	10305	17371	22089	22881	25282	6789	29247	NA	0.786325108	0.104397857	22994.6	21049.75	Down	-0.12749200265088	WW domain-binding protein 11 OS=Mus musculus GN=Wbp11 PE=1 SV=2
STX1B	P61264	29	29	20884.37	48065.89	27943.22	97321.81	47158.19	75983.07	72510.07	41360.3	53327.33	21688.48	0.786413802	0.104348873	48274.696	52973.85	Up	0.134013189595973	Syntaxin-1B OS=Mus musculus GN=Stx1b PE=1 SV=1
TCP1	P11983	10	10	21352.89	21655.78	23931.33	25955.56	23829.89	23074.67	23668.22	32822.56	23608.44	7581.44	0.787136839	0.103949762	23345.09	22151.066	Down	-0.0757430216254581	T-complex protein 1 subunit alpha OS=Mus musculus GN=Tcp1 PE=1 SV=3
USO1	Q9Z1Z0-4	4	4	18112.5	52725	4103.25	140891.5	46879	93006.25	94761.25	15439.75	41576.5	NA	0.787379936	0.103815656	52542.25	61195.938	Up	0.219957900741598	Isoform 4 of General vesicular transport factor p115 OS=Mus musculus GN=Uso1
SEL1L	Q9Z2G6	1	1	41022	66139	23453	146203	41112	93043	64940	NA	53712	NA	0.787429067	0.103788558	63585.8	70565	Up	0.150248170676493	Protein sel-1 homolog 1 OS=Mus musculus GN=Sel1l PE=2 SV=2
RILPL1	Q9JJC6	1	1	758	1027	NA	1849	468	942	934	NA	NA	NA	0.787705918	0.103635892	1025.5	938	Down	-0.128667663964476	RILP-like protein 1 OS=Mus musculus GN=Rilpl1 PE=2 SV=1
NDUFAF8	A2AMZ4	1	1	43120	41112	68020	29930	47677	54111	37659	25053	87058	43080	0.788326064	0.103294115	45971.8	49392.2	Up	0.103534074696124	Uncharacterized protein C17orf89 homolog OS=Mus musculus PE=4 SV=1
SH3KBP1	Q8R550-3	2	2	62766.5	66574	51684.5	36391	70812	46474.5	54844	NA	82549.5	NA	0.788520047	0.103187261	57645.6	61289.333	Up	0.0884255187664342	Isoform 3 of SH3 domain-containing kinase-binding protein 1 OS=Mus musculus GN=Sh3kbp1
CLIP2	Q9Z0H8-2	21	21	27677.67	28826	17405.38	40190.9	32074.14	35487.57	35808.05	12733.57	41589	10077.29	0.788661245	0.1031095	29234.818	27139.096	Down	-0.107314944353944	Isoform 2 of CAP-Gly domain-containing linker protein 2 OS=Mus musculus GN=Clip2
ENSA	P60840	11	11	246226.73	207672.36	199889.36	71999.18	211682.45	151136.55	187448.36	187748.27	284967.27	61289.36	0.788922867	0.102965456	187494.02	174517.96	Down	-0.103469020438092	Alpha-endosulfine OS=Mus musculus GN=Ensa PE=1 SV=1
CNOT3	Q8K0V4	1	1	NA	18766	NA	17490	12211	15562	18137	NA	12557	NA	0.789473259	0.102662576	16155.667	15418.667	Down	-0.0673622717112426	CCR4-NOT transcription complex subunit 3 OS=Mus musculus GN=Cnot3 PE=1 SV=1
CKAP4	Q8BMK4	20	20	14561.53	21411.79	7913.26	50868.95	19745	36877.79	33480.37	11206.89	18643.26	433.05	0.789581731	0.102602909	22900.106	20128.272	Down	-0.186130953206766	Cytoskeleton-associated protein 4 OS=Mus musculus GN=Ckap4 PE=1 SV=2
RPL34	Q9D1R9	9	9	142645.89	153004.89	181055.89	86172.56	131635.67	119678.11	148102.56	103249.89	203186.78	84557.67	0.789802778	0.102481343	138902.98	131755	Down	-0.0762198170177177	60S ribosomal protein L34 OS=Mus musculus GN=Rpl34 PE=1 SV=2
SLC3A2	P10852	9	9	40963.11	80485	59656.78	130809.22	84672.44	123764	129229.78	57898.44	47705.67	69333.89	0.7901291	0.102301943	79317.31	85586.356	Up	0.109745073253776	4F2 cell-surface antigen heavy chain OS=Mus musculus GN=Slc3a2 PE=1 SV=1
FHL2	O70433	2	2	29515.5	24029.5	27338.5	33505	25481.5	34727	31670	31817.5	40572.5	8969.5	0.791567681	0.101511946	27974	29551.3	Up	0.0791350384125063	Four and a half LIM domains protein 2 OS=Mus musculus GN=Fhl2 PE=1 SV=1
ETL4	A2AQ25-8	11	11	27199.27	28041	24045.18	23144.73	34173.82	29708.36	33754.27	18606.82	42357.82	19004.45	0.791723323	0.101426561	27320.8	28686.344	Up	0.070364383250351	Isoform 8 of Sickle tail protein OS=Mus musculus GN=Skt
PSD3	Q2PFD7-4	10	10	88562.1	83576.2	73944	84438.2	76527.3	73511.1	87204.9	73945.7	107135.5	52058.9	0.791891746	0.101334184	81409.56	78771.22	Down	-0.0475296012571856	Isoform 4 of PH and SEC7 domain-containing protein 3 OS=Mus musculus GN=Psd3
CYS1	Q8R4T1	1	1	1572	2440	NA	3462	3449	3516	2329	NA	2823	NA	0.79207194	0.101235372	2730.75	2889.3333	Up	0.0814394112068463	Cystin-1 OS=Mus musculus GN=Cys1 PE=1 SV=1
HNRNPA1	P49312	13	13	77563.54	57213	48284.69	59723.46	63937.62	76033.54	60627.85	69199.46	68394.69	42634.62	0.792646383	0.100920518	61344.462	63378.032	Up	0.0470497547373623	Heterogeneous nuclear ribonucleoprotein A1 OS=Mus musculus GN=Hnrnpa1 PE=1 SV=2
MICU3	Q9CTY5	1	1	1296	7165	NA	8758	4735	8376	4168	NA	NA	NA	0.792897822	0.100782775	5488.5	6272	Up	0.192513643054965	Calcium uptake protein 3, mitochondrial OS=Mus musculus GN=Micu3 PE=2 SV=2
CC2D1A	Q8K1A6	7	7	28223.86	33332.71	28348.71	27395.29	29379.57	34190.71	32587.14	10000	45406	15295.14	0.793234469	0.100598422	29336.028	27495.798	Down	-0.0934623901629448	Coiled-coil and C2 domain-containing protein 1A OS=Mus musculus GN=Cc2d1a PE=1 SV=2
PA2G4	P50580	12	12	16063.58	20514.5	17603.25	23476.33	19141.67	20683.17	24486.83	12363.83	25371.25	9046.75	0.793343571	0.100538693	19359.866	18390.366	Down	-0.0741187748271134	Proliferation-associated protein 2G4 OS=Mus musculus GN=Pa2g4 PE=1 SV=3
TNKS1BP1	P58871	15	15	13306	14259.54	6169.08	21838.54	16836.92	22249.15	17792.31	13477.38	19785.46	4551.46	0.793589751	0.100403949	14482.016	15571.152	Up	0.104613233373944	182 kDa tankyrase-1-binding protein OS=Mus musculus GN=Tnks1bp1 PE=1 SV=2
DAPK3	O54784	1	1	NA	NA	NA	3456	538	1908	1100	NA	NA	NA	0.793846768	0.100263319	1997	1504	Down	-0.409029765766335	Death-associated protein kinase 3 OS=Mus musculus GN=Dapk3 PE=1 SV=1
GAPDHS	Q64467	3	3	99918.33	109190	151418.33	238691.33	117953.33	144561.67	135185	150109.33	131166.67	120218.33	0.794023318	0.100166744	143434.26	136248.2	Down	-0.0741525301500288	Glyceraldehyde-3-phosphate dehydrogenase, testis-specific OS=Mus musculus GN=Gapdhs PE=2 SV=1
FTL1	P29391	2	2	14810.5	17567.5	2210.5	26265	12497	20796.5	12173	1424	17997.5	NA	0.794191369	0.100074837	14670.1	13097.75	Down	-0.16355970595227	Ferritin light chain 1 OS=Mus musculus GN=Ftl1 PE=1 SV=2
PFN1	P62962	22	22	208971.68	215270.32	249031.77	159266.55	189416.73	156150.23	199624.55	233701.68	205629.18	200767.09	0.794380412	0.099971473	204391.41	199174.55	Down	-0.0373012786749846	Profilin-1 OS=Mus musculus GN=Pfn1 PE=1 SV=2
DNAJA1	P63037	16	16	17448.67	19230.53	15151.33	30193.73	20417	21391.67	17738.53	30407.93	17754.73	9389.4	0.794565055	0.099870539	20488.252	19336.452	Down	-0.0834738005334975	DnaJ homolog subfamily A member 1 OS=Mus musculus GN=Dnaja1 PE=1 SV=1
SLC2A13	Q3UHK1	4	4	7154.5	12533	2857	22364.25	15578.25	22147	24993.75	4539.25	14419.75	2031.5	0.795470465	0.099375941	12097.4	13626.25	Up	0.171691567559747	Proton myo-inositol cotransporter OS=Mus musculus GN=Slc2a13 PE=2 SV=2
CSPG5	Q71M36-3	8	8	14872	29230.83	13341.5	87874.83	33600.83	51459	55592.67	13800.67	32842.17	2260.83	0.795547083	0.099334112	35783.998	31191.068	Down	-0.19818163056084	Isoform 3 of Chondroitin sulfate proteoglycan 5 OS=Mus musculus GN=Cspg5
RPS16	P14131	7	7	64200.86	77016.43	53579.71	99140.29	60870.29	76253	79898.86	53696.71	86684.86	42792.29	0.795841904	0.099173198	70961.516	67865.144	Down	-0.0643660445659421	40S ribosomal protein S16 OS=Mus musculus GN=Rps16 PE=2 SV=4
EIF5B	Q05D44	2	2	NA	NA	NA	7958	7386	6250	9040	NA	8506	NA	0.795996145	0.099089036	7672	7932	Up	0.0480819572107171	Eukaryotic translation initiation factor 5B OS=Mus musculus GN=Eif5b PE=1 SV=2

HSPD1	P63038	152	152	114774.9	110820.36	104922.28	89589.86	105294.36	101551.16	103286.22	102323.15	102252.29	124158.23	0.796216114	0.098969037	105080.35	106714.21	Up	0.02225935900031	60 kDa heat shock protein, mitochondrial OS=Mus musculus GN=Hspd1 PE=1 SV=1
SH2D5	Q8JZW5	1	1	NA	4557	NA	41125	4996	15188	11436	NA	NA	NA	0.796662479	0.098725636	16892.667	13312	Down	-0.343669750536547	SH2 domain-containing protein 5 OS=Mus musculus GN=Sh2d5 PE=2 SV=2
HSPD1	P63038-2	77	77	131395.62	122688	122529.72	83477.68	117501.28	102498.45	115585.78	111738.76	99512.32	168424.85	0.79671887	0.098694897	115518.46	119552.03	Up	0.0495152368271737	Isoform 2 of 60 kDa heat shock protein, mitochondrial OS=Mus musculus GN=Hspd1
TXNDC12	Q9CQU0	5	5	16266.8	9955	9073.8	17275.8	14771.2	19283.8	12385.4	3386.6	14757.2	NA	0.797956259	0.098020915	13468.52	12453.25	Down	-0.113069027428571	Thioredoxin domain-containing protein 12 OS=Mus musculus GN=Txndc12 PE=2 SV=1
VAPA	Q9WV55	7	7	12771.71	28262.29	28758.43	81826.29	22422.43	46424.43	38256.14	29006	32327.57	9946.86	0.798562674	0.097690993	34808.23	31192.2	Down	-0.158243144585988	Vesicle-associated membrane protein-associated protein A OS=Mus musculus GN=Vapa PE=1 SV=2
GNAS	Q6R0H7	7	7	42840.57	62304.43	45680.57	100992	64444.57	89439.86	104343.14	59585.29	57290.57	27957	0.798603967	0.097668537	63252.428	67723.172	Up	0.0985286873983903	Guanine nucleotide-binding protein G(s) subunit alpha isoforms XLas OS=Mus musculus GN=Gnas PE=2 SV=1
ARMC10	Q9D0L7	1	1	5541	5522	5672	15566	6335	9724	6993	NA	4479	NA	0.798651052	0.097642932	7727.2	7065.3333	Down	-0.129188112470146	Armadillo repeat-containing protein 10 OS=Mus musculus GN=Armc10 PE=1 SV=1
DNM1	P39053-5	51	51	43853.48	54165.82	38016.12	72349.3	52289.86	63786.6	62803.18	45610.4	48279.86	28743	0.798736595	0.097596417	52134.916	49844.608	Down	-0.0648124557588378	Isoform 5 of Dynamin-1 OS=Mus musculus GN=Dnm1
GRIN2B	Q01097	24	24	11621.52	21943.61	9753.65	47226.87	28124.57	39037.83	39901.87	15343.65	22225	13774.96	0.799227919	0.097329354	23734.044	26056.662	Up	0.134694339043713	Glutamate receptor ionotropic, NMDA 2B OS=Mus musculus GN=Grin2b PE=1 SV=3
GCDH	Q60759	1	1	2297	9789	NA	9626	6932	7320	7985	NA	NA	NA	0.799293582	0.097293674	7161	7652.5	Up	0.0957700726114212	Glutaryl-CoA dehydrogenase, mitochondrial OS=Mus musculus GN=Gcdh PE=1 SV=2
ABLIM1	Q8K4G5-4	12	12	19862.83	25707.67	17777.67	23102.17	26214.42	24870.58	26774.83	18012.25	30258.33	6781.75	0.799384385	0.09724434	22532.952	21339.548	Down	-0.0785067124537887	Isoform 4 of Actin-binding LIM protein 1 OS=Mus musculus GN=Ablim1
HSPA1A	Q61696	23	23	36955.3	37387.91	35750.57	69396.22	35533.7	47405.78	39422	34443.35	39357.13	45037.74	0.799820087	0.097007693	43004.74	41133.2	Down	-0.0641923708522593	Heat shock 70 kDa protein 1A OS=Mus musculus GN=Hspa1a PE=1 SV=2
DNM1L	Q8K1M6-2	15	15	19123.43	25795.64	21557.29	44020.36	26217.21	31775.93	33543.14	18759.93	24682.57	34928.21	0.801109072	0.09630835	27342.786	28737.956	Up	0.0717972041847198	Isoform 2 of Dynamin-1-like protein OS=Mus musculus GN=Dnm1l
SLC6A11	P31650	9	9	19511.44	36894.22	10026.11	119569.44	35772.33	62674.33	63220	13749.44	33566.11	19486.33	0.801132557	0.096295619	44354.708	38539.242	Down	-0.202759049922533	Sodium- and chloride-dependent GABA transporter 3 OS=Mus musculus GN=Slc6a11 PE=1 SV=2
ARHGEF7	Q9ES28-4	6	6	34768.6	48295.8	38079	70930.6	50201.8	55214	52143.8	27871.6	51126.8	45504.2	0.801585319	0.096050245	48455.16	46372.08	Down	-0.0633938670755672	Isoform D of Rho guanine nucleotide exchange factor 7 OS=Mus musculus GN=Arhgef7
UBE2V1	Q9CZY3-2	16	16	38840.79	37232.29	37603.93	21701.43	32163.29	30629.14	31968.21	36481.86	36730.29	26938.21	0.801814609	0.095926035	33508.346	32549.542	Down	-0.0418832320894103	Isoform 2 of Ubiquitin-conjugating enzyme E2 variant 1 OS=Mus musculus GN=Ube2v1
RMDN3	Q3UJU9	2	2	11231	17975	14646.5	20908	16650	22072	17300	13003.5	9385	NA	0.801929981	0.09586355	16282.1	15440.125	Down	-0.076602352220316	Regulator of microtubule dynamics protein 3 OS=Mus musculus GN=Rmdn3 PE=1 SV=2
CNKSR3	Q8BMA3	2	2	13497	18282	5765	30540.5	17869	26636.5	27371	9988	21673.5	7765	0.802064022	0.095790964	17190.7	18686.8	Up	0.120391245438882	Connector enhancer of kinase suppressor of ras 3 OS=Mus musculus GN=Cnksr3 PE=1 SV=1
RPS10	P63325	11	11	98946.36	97405.91	136986.36	73999.73	79042.18	75650	98368.18	90138.09	131380.36	70874.09	0.80214614	0.095746502	97276.108	93282.144	Down	-0.060484560089108	40S ribosomal protein S10 OS=Mus musculus GN=Rps10 PE=1 SV=1
PPP1R13B	Q62415	1	1	1563	2096	NA	7708	2270	4034	1807	NA	NA	NA	0.802687947	0.095453258	3409.25	2920.5	Down	-0.223239011276909	Apoptosis-stimulating of p53 protein 1 OS=Mus musculus GN=Ppp1r13b PE=2 SV=2
ARHGAP44	Q5SSM3-4	1	1	62767	94452	92265	103765	71959	91505	93868	53060	88935	NA	0.803105306	0.095227505	85041.6	81842	Down	-0.0553273393231317	Isoform 4 of Rho GTPase-activating protein 44 OS=Mus musculus GN=Arhgap44
TPPP	Q7TQD2	37	37	176898.28	131164.97	139489.19	121166.22	158336.53	129791.89	136274.92	146583.72	149532.25	150771	0.803247227	0.095150765	145411.04	142590.76	Down	-0.0282563303881393	Tubulin polymerization-promoting protein OS=Mus musculus GN=Tppp PE=1 SV=1
MYH9	Q8VDD5	33	33	4316.07	10496.93	4227.1	34094.83	14846.5	20287.17	20595.23	3698.27	11837.43	2893.53	0.803277759	0.095134258	13596.286	11862.326	Down	-0.196825689090019	Myosin-9 OS=Mus musculus GN=Myh9 PE=1 SV=4
MRPS23	Q8VE22	11	11	22948.18	21104.27	26083	20976.82	22280.18	22439.64	23857.27	14510.18	26061.73	30110.45	0.803430221	0.095051836	22678.49	23395.854	Up	0.0449283065586715	28S ribosomal protein S23, mitochondrial OS=Mus musculus GN=Mrps23 PE=1 SV=1
NSFL1C	Q9CZ44	9	9	9590.78	8223.67	2845.89	17765.22	10313.44	14212.56	11976.56	2344.67	14312	NA	0.803494941	0.095016853	9747.8	10711.448	Up	0.136004896953185	NSFL1 cofactor p47 OS=Mus musculus GN=Nsfl1c PE=1 SV=1
MRPS26	Q80ZS3	5	5	18277.8	18536	4926	24876.6	16073.8	20472	19528.6	6990.2	24308.6	NA	0.803718551	0.094896008	16538.04	17824.85	Up	0.108101671982503	28S ribosomal protein S26, mitochondrial OS=Mus musculus GN=Mrps26 PE=1 SV=1
AFDN	Q9QZQ1	12	12	10620.58	16548	6764.17	23805.75	19542.08	22932.17	25957.42	7971.33	23323.58	4102.42	0.803735212	0.094887005	15456.116	16857.384	Up	0.125202843218221	Afadin OS=Mus musculus GN=Mllt4 PE=1 SV=3
TUBB3	Q9ERD7	67	67	51904.86	60094.74	57284.15	62617.65	47547.28	56815.71	56545.15	57676.11	52392.62	52124.26	0.803738273	0.09488535	55889.736	55110.77	Down	-0.0202490758767056	Tubulin beta-3 chain OS=Mus musculus GN=Tubb3 PE=1 SV=1
TRIO	Q0KL02-4	8	8	3636.17	6351.67	4049.83	17061.17	9278.83	12422.5	12435.67	5040.33	6381.67	7845.5	0.803845473	0.09482743	8075.534	8825.134	Up	0.128060522175344	Isoform 4 of Triple functional domain protein OS=Mus musculus GN=Trio
ADAM23	Q9R1V7-3	8	8	34575.88	42173.12	27637.75	43179.12	40411.12	46767.62	45065.75	35832.25	38609.5	12958.62	0.803877117	0.094810333	37595.398	35846.748	Down	-0.0687138301790421	Isoform Gamma of Disintegrin and metalloproteinase domain-containing protein 23 OS=Mus musculus GN=Adam23
IGSF8	Q8R366	17	17	21173.59	38624.29	20918.41	89680.18	55782.35	85387.12	68465.71	31224.76	49621.41	14616.41	0.803882972	0.094807171	45235.764	49863.082	Up	0.140508219727976	Immunoglobulin superfamily member 8 OS=Mus musculus GN=Igsf8 PE=1 SV=2
LMNA	P48678-3	7	7	39231.86	18492.43	23466.86	17236.14	24758.14	26236.57	21023.14	23455.57	23567.29	NA	0.804186769	0.094643076	24637.086	23570.643	Down	-0.0638405437133778	Isoform C2 of Prelamin-A/C OS=Mus musculus GN=Lmna
EPDR1	Q99M71	4	4	83924.5	70966.5	80369.25	40358	64012	61408.75	82705.25	70744	58612.5	77742.25	0.804831211	0.09429519	67926.05	70242.55	Up	0.0483802578870797	Mammalian ependymin-related protein 1 OS=Mus musculus GN=Epdr1 PE=2 SV=1
CIT	P49025-4	14	14	20254.71	26266.14	18664	27395.29	31930.14	32071.93	31411.29	28365.64	34025.64	6011.14	0.805382443	0.093997842	24902.056	26377.128	Up	0.0830226282050144	Isoform 4 of Citron Rho-interacting kinase OS=Mus musculus GN=Cit
CLSTN3	Q99JH7	3	3	6115.67	9007	8659	9090.33	8985	12479.33	10243.67	4877.33	11884.67	4690.67	0.805824094	0.093759751	8371.4	8835.134	Up	0.0777831022989584	Calsyntenin-3 OS=Mus musculus GN=Clstn3 PE=1 SV=1
HECW2	Q6I6G8	1	1	13133	33460	3359	60865	29524	38330	36493	20703	27767	NA	0.805886989	0.093725856	28068.2	30823.25	Up	0.135082439988747	E3 ubiquitin-protein ligase HECW2 OS=Mus musculus GN=Hecw2 PE=2 SV=1
SLC25A11	Q9CR62	2	2	7977	6165.5	5570	17776	6830.5	8990	11320.5	6875	4854	9045.5	0.805954406	0.093689526	8863.8	8217	Down	-0.109313562471999	Mitochondrial 2-oxoglutarate/malate carrier protein OS=Mus musculus GN=Slc25a11 PE=1 SV=3
SLTM	Q8CH25	1	1	27592	12774	11832	13238	17505	19665	14749	25510	10832	NA	0.80612957	0.093595148	16588.2	17689	Up	0.0926951447590204	SAFB-like transcription modulator OS=Mus musculus GN=Sltm PE=1 SV=1
ACTG1	P63260	108	108	200380.32	248570.95	240161.78	265468.94	211556.72	246669.51	294660.77	192021.72	218881.22	240364.61	0.806167869	0.093574515	233227.74	238519.57	Up	0.0323682122116842	Actin, cytoplasmic 2 OS=Mus musculus GN=Actg1 PE=1 SV=1
MCU	Q3UMR5	1	1	1593	5556	NA	25581	4613	11038	9200	7834	3229	NA	0.806608967	0.093336954	9335.75	7825.25	Down	-0.254629084146423	Calcium uniporter protein, mitochondrial OS=Mus musculus GN=Mcu PE=1 SV=2
ABI2	P62484	7	7	120432.71	125668.43	103335.71	95170	117509.57	120110.86	116546.57	111085.57	130164.57	68860.29	0.806764194	0.093253385	112423.28	109353.57	Down	-0.0399405169151485	Abl interactor 2 OS=Mus musculus GN=Abi2 PE=2 SV=1
RUFY3	Q9D394-3	2	2	14719	7388.5	NA	44306.5	11181.5	25744	16217	NA	8654	NA	0.807221413	0.093007326	19398.875	16871.667	Down	-0.201370491331627	Isoform 3 of Protein RUFY3 OS=Mus musculus GN=Rufy3
TOM1L2	Q5SRX1-4	12	12	25970	30894.67	23215.56	37258	30780.67	34779.11	34238	18268.67	41329.44	11725.78	0.807260666	0.092986208	29623.78	28068.2	Down	-0.0778191930496738	Isoform 4 of TOM1-like protein 2 OS=Mus musculus GN=Tom1l2
CEP170	Q6A065-3	6	6	19320.33	19878.5	19482.5	18340.5	20231.33	18369.67	20149.83	5357.33	33363.17	14064.17	0.807354411	0.092935778	19450.632	18260.834	Down	-0.0910643758682021	Isoform 3 of Centrosomal protein of 170 kDa OS=Mus musculus GN=Cep170
CROCC	Q8CJ40-2	11	11	31267.18	36756.27	21136.18	33870.09	35299.27	40671.09	33900.64	10129.45	39222.45	26461.45	0.807420193	0.092900393	31665.798	30077.016	Down	-0.0742639952780657	Isoform 2 of Rootletin OS=Mus musculus GN=Crocc
NUDT3	Q9JI46	3	3	70859.67	61377.33	67566.67	49705.33	56319	56652	59504.67	44557	80641.67	73930.33	0.807673906	0.092763948	61165.6	63057.134	Up	0.0439391015024862	Diphosphoinositol polyphosphate phosphohydrolase 1 OS=Mus musculus GN=Nudt3 PE=1 SV=1
MDH2	P08249	133	133	126814.08	120847.51	101399.15	126208.78	125419.82	121884.91	119245.61	114028.25	135710.29	100434.78	0.808048722	0.092562452	120137.87	118260.77	Down	-0.0227194154519883	Malate dehydrogenase, mitochondrial OS=Mus musculus GN=Mdh2 PE=1 SV=3
ARHGEF7	Q9ES28-6	4	4	40733.75	57048	44488	85914.75	60553	66353	61476.25	33450.25	61073.25	53989.75	0.808187306	0.092487975	57747.5	55268.5	Down	-0.0633010328430576	Isoform F of Rho guanine nucleotide exchange factor 7 OS=Mus musculus GN=Arhgef7
KATNAL1	Q8K0T4	1	1	4679	6186	3301	17184	8897	10235	9356	NA	6620	NA	0.808191952	0.092485479	8049.4	8737	Up	0.118256741315124	Katanin p60 ATPase-containing subunit A-like 1 OS=Mus musculus GN=Katnal1 PE=1 SV=1
MRPS7	Q80X85	3	3	17518.33	15785.33	10190	24282.67	18167.33	22233.67	13741.67	4678.67	23110.33	NA	0.808645954	0.092241582	17188.732	15941.085	Down	-0.10871329520144	28S ribosomal protein S7, mitochondrial OS=Mus musculus GN=Mrps7 PE=2 SV=1
MRPS18A	Q99N85	2	2	52813.5	60802.5	30883	26052.5	53802	50790	49263.5	22437.5	55184.5	35213.5	0.808712817	0.092205674	44870.7	42577.8	Down	-0.075672283290308	28S ribosomal protein S18a, mitochondrial OS=Mus musculus GN=Mrps18a PE=2 SV=1
BLVRA	Q9CY64	3	3	53665.67	66460.33	33492.67	62221.67	62267.33	59124.67	70196.33	36058.67	66917.33	NA	0.80889035	0.092110346	55621.534	58074.25	Up	0.0622550837913269	Biliverdin reductase A OS=Mus musculus GN=Blvra PE=2 SV=1
TPPP3	Q9CRB6	6	6	109476.83	105116	63406.17	128213.83	119520.83	114825.17	125426.5	81149.17	129584.67	51431	0.809309484	0.09188537	105146.73	100483.3	Down	-0.0654482324773184	Tubulin polymerization-promoting protein family member 3 OS=Mus musculus GN=Tppp3 PE=1 SV=1
CAMK2D	Q6PHZ2-4	25	25	83841.84	117576.24	76031.8	249950.44	116510.36	153519.36	165110.04	93033.52	105115.96	82333.88	0.809366286	0.09185489	128782.14	119822.55	Down	-0.10403301835137	Isoform 3 of Calcium/calmodulin-dependent protein kinase type II subunit delta OS=Mus musculus GN=Camk2d
REEP2	Q8VCD6	6	6	4548.33	7999.67	4776.33	19180.83	8013.5	11995	11651.67	10416.83	9232	4955	0.809907946	0.09156434	8903.732	9650.1	Up	0.116133723920214	Receptor expression-enhancing protein 2 OS=Mus musculus GN=Reep2 PE=2 SV=2
C2CD4C	Q5HZI2	6	6	22163.17	19466.67	12061.83	23048.83	22652	26492.83	27871	2287.5	32260.83	1629	0.810164847	0.091426605	19878.5	18108.232	Down	-0.134563202120588	C2 calcium-dependent domain-containing protein 4C OS=Mus musculus GN=C2cd4cC2CD4 family PE=2 SV=1
MAP1B	P14873	116	116	38792.31	42904.76	47144.72	44235.59	41924.23	36814.39	47012.19	39305.8	51296.7	44263.97	0.810247269	0.091382424	43000.322	43738.61	Up	0.0245599094841965	Microtubule-associated protein 1B OS=Mus musculus GN=Map1b PE=1 SV=2
ARHGAP1	Q5FWK3	1	1	1006	2076	1271	10730	1612	4616	3080	NA	NA	NA	0.810252537	0.0913796	3339	3848	Up	0.204692705706926	Rho GTPase-activating protein 1 OS=Mus musculus GN=Arhgap1 PE=1 SV=1
PEA15A	Q62048-2	6	6	66014.17	94689.83	89281.33	104589.33	85330.83	96062.33	93621	120937.67	71246.83	38953.67	0.810341303	0.091332025	87981.098	84164.3	Down	-0.0639851915373917	Isoform 2 of Astrocytic phosphoprotein PEA-15 OS=Mus musculus GN=Pea15
ANKIB1	Q6ZPS6	1	1	5978	8616	NA	16677	11006	12544	12917	NA	3246	NA	0.810474879	0.091260442	10569.25	9569	Down	-0.143432935776657	Ankyrin repeat and IBR domain-containing protein 1 OS=Mus musculus GN=Ankib1 PE=1 SV=2
NCKIPSD	Q9ESJ4	2	2	13016.5	15369.5	6029.5	27704.5	21108.5	21238	18320	12518	4094	20988.5	0.810559617	0.091215037	16645.7	15431.7	Down	-0.109252539211893	NCK-interacting protein with SH3 domain OS=Mus musculus GN=Nckipsd PE=2 SV=2
KIF5A	P33175	8	8	28618.43	33722.43	21679.71	49776.43	38375.71	45386.29	41511.86	27709.86	39788.29	25416	0.810857815	0.091055293	34434.542	35962.46	Up	0.0626352228470769	Kinesin heavy chain isoform 5A OS=Mus musculus GN=Kif5a PE=1 SV=3
RAC2	Q05144	2	2	22953	37898.5	24158	106334.5	30043.5	63221	51977	25714	35310	23277	0.811330105	0.090802409	44277.5	39899.8	Down	-0.150192251972552	Ras-related C3 botulinum toxin substrate 2 OS=Mus musculus GN=Rac2 PE=2 SV=1
OCIAD1	Q9CRD0	3	3	14319	21375.33	1092.67	60531.67	19632.33	35177.33	24432.67	8432.33	14022.67	NA	0.811661225	0.090625201	23390.2	20516.25	Down	-0.189137141194591	OCIA domain-containing protein 1 OS=Mus musculus GN=Ociad1 PE=1 SV=1
COX2	P00405	2	2	30229	49523	101051.5	135881.5	58675.5	85463.5	82077	120761	60477.5	54275	0.812248649	0.090311002	75072.1	80610.8	Up	0.102696299552257	Cytochrome c oxidase subunit 2 OS=Mus musculus GN=Mtco2 PE=1 SV=1
WASF3	Q8VHI6	8	8	8732.62	12801.88	8768.75	13068.12	11540.75	12203.12	11801.88	9976.12	13934.38	4717.75	0.812390944	0.090234926	10982.424	10526.65	Down	-0.0611501295177713	Wiskott-Aldrich syndrome protein family member 3 OS=Mus musculus GN=Wasf3 PE=2 SV=1
SYN1	O88935-1	101	101	114952.87	101921.98	96248.54	114838.14	94396.72	115799.32	104506.72	87355.41	105625.73	101265.37	0.812811919	0.090009936	104471.65	102910.51	Down	-0.0217211690856896	Isoform Ib of Synapsin-1 OS=Mus musculus GN=Syn1
MAP7D2	A2AG50	1	1	132995	109304	104920	89966	117737	114538	123917	50253	143107	101831	0.813256314	0.089772556	110984.4	106729.2	Down	-0.0564019686485633	MAP7 domain-containing protein 2 OS=Mus musculus GN=Map7d2 PE=1 SV=1
SRI	Q6P069	4	4	9447.5	8177.5	11264.25	5640.75	8830.5	6829.5	7303.25	11963.25	11517.75	7508.5	0.81361178	0.089582772	8672.1	9024.45	Up	0.0574576160078581	Sorcin OS=Mus musculus GN=Sri PE=1 SV=1
GGCT	Q9D7X8	1	1	2201	3773	NA	8380	3208	5188	2640	NA	NA	NA	0.814319367	0.089205236	4390.5	3914	Down	-0.165741490503103	Gamma-glutamylcyclotransferase OS=Mus musculus GN=Ggct PE=1 SV=1
NCAM4	P13595-4	29	29	20508.68	33058.93	28432.96	83614.71	29004.25	54161.07	51895.21	45353.79	35093.29	23674.54	0.814615886	0.089047125	38923.906	42035.58	Up	0.11095448799353	Isoform 4 of Neural cell adhesion molecule 1 OS=Mus musculus GN=Ncam1
DNM1	P39053-6	50	50	44244.86	54596.1	38142.73	73297.63	52880.18	64493.02	63595.1	46541.22	48583.82	29329.59	0.815245408	0.088711639	52632.3	50508.55	Down	-0.0594208156590548	Isoform 6 of Dynamin-1 OS=Mus musculus GN=Dnm1
ANKRD27	Q3UMR0-3	1	1	189575	653479	NA	1624209	604796	1040296	998285	223022	451871	NA	0.81538197	0.088638896	768014.75	678368.5	Down	-0.179064838690544	Isoform 3 of Ankyrin repeat domain-containing protein 27 OS=Mus musculus GN=Ankrd27
CTNND1	P30999	22	22	15052.53	16139.47	10268.21	23747	18414.89	22909.16	18517.32	9020.84	18596.47	10415.37	0.815417664	0.088619885	16724.42	15891.832	Down	-0.0736707321049655	Catenin delta-1 OS=Mus musculus GN=Ctnnd1 PE=1 SV=2
AKAP5	D3YVF0	23	23	16724.22	17269.96	13098.61	24379.43	18731.43	21695.57	18972.39	13266.22	22876.74	9604.96	0.815505825	0.088572933	18040.73	17283.176	Down	-0.0618893618369831	A-kinase anchor protein 5 OS=Mus musculus GN=Akap5 PE=1 SV=2
STAU1	Q9Z108	1	1	4207	3227	3094	8360	4368	5439	4526	NA	3132	NA	0.815678764	0.088480844	4651.2	4365.6667	Down	-0.0914009966716429	Double-stranded RNA-binding protein Staufen homolog 1 OS=Mus musculus GN=Stau1 PE=2 SV=1
LRRFIP1	Q3UZ39-2	2	2	8444	7885	NA	13441	5811	12090	8105	NA	4448	NA	0.81582974	0.088400467	8895.25	8214.3333	Down	-0.11489166053192	Isoform 2 of Leucine-rich repeat flightless-interacting protein 1 OS=Mus musculus GN=Lrrfip1
TPR	F6ZDS4	2	2	NA	4001	NA	6530	3013	6239	3900	NA	4358	NA	0.816119647	0.088246167	4514.6667	4832.3333	Up	0.098100502382474	Nucleoprotein TPR OS=Mus musculus GN=Tpr PE=1 SV=1
BAG4	Q8CI61	1	1	44075	33956	38002	20569	23335	26380	24904	40444	30777	NA	0.816334885	0.088131644	31987.4	30626.25	Down	-0.0627350036485097	BAG family molecular chaperone regulator 4 OS=Mus musculus GN=Bag4 PE=1 SV=2
PYCRL	Q9DCC4	5	5	111334	104804.2	143864.4	64243.4	106548.8	101258.6	105359.4	102977	119715.6	117701.4	0.816743072	0.087914541	106158.96	109402.4	Up	0.0434182450591307	Pyrroline-5-carboxylate reductase 3 OS=Mus musculus GN=Pycrl PE=2 SV=2
RTN3	Q9ES97	26	26	11351.88	20406.5	7469.33	59680.62	24754.92	39560.62	40406.29	9341.12	19396.88	275.21	0.816748484	0.087911663	24732.65	21796.024	Down	-0.182351842143244	Reticulon-3 OS=Mus musculus GN=Rtn3 PE=1 SV=2
PPP2R2A	Q6P1F6	5	5	37296.2	42020.2	42729.6	85132.8	41080.8	54821.6	36790.4	45735.2	43034	56146.2	0.816760709	0.087905162	49651.92	47305.48	Down	-0.0698421875933874	Serine/threonine-protein phosphatase 2A 55 kDa regulatory subunit B alpha isoform OS=Mus musculus GN=Ppp2r2a PE=1 SV=1
PPT1	O88531	5	5	19195.25	20839.25	13793.5	21067.5	20009	22930.5	20166.5	2805	24799.5	NA	0.816839295	0.087863378	18980.9	17675.375	Down	-0.102807577418355	Palmitoyl-protein thioesterase 1 OS=Mus musculus GN=Ppt1 PE=2 SV=2
TWF2	Q9Z0P5	3	3	22334	19599	6540.33	47009.33	20754	28051	25253.33	13211.67	19360	NA	0.817244118	0.087648197	23247.332	21469	Down	-0.11481015981178	Twinfilin-2 OS=Mus musculus GN=Twf2 PE=1 SV=1
SLC9A3R2	Q9JHL1	2	2	22686.5	20107	10571.5	16300	22514.5	25046	22693.5	5598	25557	NA	0.817957146	0.087269449	18435.9	19723.625	Up	0.0974068812953042	Na(+)/H(+) exchange regulatory cofactor NHE-RF2 OS=Mus musculus GN=Slc9a3r2 PE=1 SV=2
DYNC1I1	O88485-2	8	8	11124.14	15644.71	11964.57	19836.14	16440	23460.57	21886	12332.71	17018.71	4817.57	0.818114477	0.087185922	15001.912	15903.112	Up	0.0841627223420395	Isoform 1B of Cytoplasmic dynein 1 intermediate chain 1 OS=Mus musculus GN=Dync1i1
ACTR3B	Q641P0	2	2	95332	125815.5	119442	196822	150760	172376	187843	37485.5	102983.5	149025	0.818240512	0.087119022	137634.3	129942.6	Down	-0.0829655732826476	Actin-related protein 3B OS=Mus musculus GN=Actr3b PE=2 SV=1
NDUFAF3	Q9JKL4	4	4	12284.25	13233	10634	16090.75	10397.25	15371	11923.75	5857	17565.25	9106	0.818281197	0.087097428	12527.85	11964.6	Down	-0.0663666784274677	NADH dehydrogenase [ubiquinone] 1 alpha subcomplex assembly factor 3 OS=Mus musculus GN=Ndufaf3 PE=2 SV=1
FUS	P56959	6	6	15437	14573	11714	13778.83	12661.67	15327.67	16066.83	7082	15835.33	11617.33	0.818314845	0.08707957	13632.9	13185.832	Down	-0.0481038805975155	RNA-binding protein FUS OS=Mus musculus GN=Fus PE=2 SV=1
ASTN1	Q61137	20	20	9955.28	11756.17	9387.89	14193.44	13240.72	14553.94	14961.22	7731.67	11154.67	12037.17	0.818365167	0.087052864	11706.7	12087.734	Up	0.0462093662889521	Astrotactin-1 OS=Mus musculus GN=Astn1 PE=2 SV=4
FSD1L	Q8BYN5	8	8	36314.57	37945.14	42843.57	29841.71	34918	35082.86	42476.29	43720.86	45106.29	21337.14	0.818756584	0.086845195	36372.598	37544.688	Up	0.045756825435978	FSD1-like protein OS=Mus musculus GN=Fsd1l PE=2 SV=2
VPS8	Q0P5W1-3	1	1	NA	2503	2167	8392	3481	6786	5874	3476	2135	NA	0.818850874	0.086795183	4135.75	4567.75	Up	0.143334712440711	Isoform 3 of Vacuolar protein sorting-associated protein 8 homolog OS=Mus musculus GN=Vps8
PPP3CB	P48453	13	13	14037.15	17488.31	14118.54	40196.69	19478.85	28702.38	26188.54	22214.92	18708.85	1389.69	0.819216175	0.086601482	21063.908	19440.876	Down	-0.11567989749707	Serine/threonine-protein phosphatase 2B catalytic subunit beta isoform OS=Mus musculus GN=Ppp3cb PE=1 SV=2
LASP1	Q61792	23	23	136027.3	118602.83	99264.22	156266.35	140258.78	142105.74	135863.35	79139.78	170529.74	100377.7	0.819220761	0.08659905	130083.9	125603.26	Down	-0.0505684392855038	LIM and SH3 domain protein 1 OS=Mus musculus GN=Lasp1 PE=1 SV=1
CBX5	Q61686	3	3	12253.67	11557.33	3679.33	8946	11827.33	10118.33	10736.67	2102.67	12916	NA	0.819359543	0.086525484	9652.732	8968.4175	Down	-0.106083883844281	Chromobox protein homolog 5 OS=Mus musculus GN=Cbx5 PE=1 SV=1
ADD1	Q9QYC0	33	33	30699.21	34111.64	31278.18	39249.55	40698.21	39061.61	45497.12	25966.09	38190.67	21458.58	0.819459118	0.086472708	35207.358	34034.814	Down	-0.0488657443583248	Alpha-adducin OS=Mus musculus GN=Add1 PE=1 SV=2
ATG14	Q8CDJ3	2	2	1418	NA	NA	1662	1057	1892	1102	NA	NA	NA	0.819518304	0.086441342	1379	1497	Up	0.118451764544436	Beclin 1-associated autophagy-related key regulator OS=Mus musculus GN=Atg14 PE=1 SV=1
PKLR	P53657	3	3	338666	486388.67	254508	550691.33	509267.67	570692.67	528436.67	510657	368462	256834	0.819570214	0.086413834	427904.33	447016.47	Up	0.0630396897189712	Pyruvate kinase PKLR OS=Mus musculus GN=Pklr PE=2 SV=1
HAGH	Q99KB8	9	9	31405	29661.89	33549.89	18484.22	33719.22	31498.33	34023	21553.78	38452.22	26107	0.819920531	0.086228238	29364.044	30326.866	Up	0.0465457474967651	Hydroxyacylglutathione hydrolase, mitochondrial OS=Mus musculus GN=Hagh PE=1 SV=2
BORCS7	Q9CRC6	3	3	37966.67	34482.33	35115	24689.67	40826	29329.33	37691.67	4151	56326.33	NA	0.820209467	0.086075222	34615.934	31874.583	Down	-0.119029829808657	UPF0693 protein C10orf32 homolog OS=Mus musculus PE=1 SV=1
WDR47	Q8CGF6	9	9	50776.71	83308.71	85744.57	103761.57	81143.86	104728.86	139036.29	35394.86	65229.14	33990.14	0.82050559	0.085918456	80947.084	75675.858	Down	-0.0971459833142514	WD repeat-containing protein 47 OS=Mus musculus GN=Wdr47 PE=1 SV=2
PRKCB	P68404-2	9	9	44370.56	63414.78	48890.11	94329.89	66665.33	85244.56	79570.44	51995.56	62209.44	21882.33	0.820648372	0.085842888	63534.134	60180.466	Down	-0.078236616542245	Isoform Beta-II of Protein kinase C beta type OS=Mus musculus GN=Prkcb

ATP6V1E1	P50518	57	57	147469.72	127338.09	107769.61	99903.51	137764.91	116857.89	131009.77	106599.23	157756.96	91092.6	0.82071088	0.085809809	124049.17	120663.29	Down	-0.0399252346078416	V-type proton ATPase subunit E 1 OS=Mus musculus GN=Atp6v1e1 PE=1 SV=2
MRPS5	Q99N87	3	3	58050	72814.33	71384.67	56690	66173	69300	68342	53074	93568.33	27220	0.821308903	0.085493469	65022.4	62300.866	Down	-0.0616845901344241	28S ribosomal protein S5, mitochondrial OS=Mus musculus GN=Mrps5 PE=2 SV=1
DLGAP2	Q8BJ42	21	21	29284.67	34058.67	28101.71	27151.05	38922.05	32156.67	35262.43	25616.76	41360	27312.29	0.821523392	0.085380066	31503.63	32341.63	Up	0.0378743192018229	Disks large-associated protein 2 OS=Mus musculus GN=Dlgap2 PE=1 SV=2
PHACTR1	Q2M3X8-3	10	10	31444	34184	43986.33	27587.67	33016.11	37654.22	39635.33	26589.78	50119.44	22781.22	0.821855692	0.085204433	34043.622	35355.998	Up	0.054570445378854	Isoform 3 of Phosphatase and actin regulator 1 OS=Mus musculus GN=Phactr1
EGLN1	Q91YE3	1	1	NA	NA	NA	1622	1553	1577	1078	NA	1919	NA	0.821885353	0.085188759	1587.5	1524.6667	Down	-0.0582627268563742	Egl nine homolog 1 OS=Mus musculus GN=Egln1 PE=2 SV=2
MRPL51	Q9CPY1	3	3	135949	127434	109076.33	85625	126233.33	106159.33	110638	133380	166283.33	41474	0.822150948	0.085048438	116863.53	111586.93	Down	-0.0666567156999293	39S ribosomal protein L51, mitochondrial OS=Mus musculus GN=Mrpl51 PE=2 SV=1
LRP1	Q91ZX7	27	27	14446.78	19004.07	13933.56	24555.56	22370.96	24832.04	29805.04	13971.81	27073.59	4686.67	0.822182695	0.085031668	18862.186	20073.83	Up	0.089819018624677	Prolow-density lipoprotein receptor-related protein 1 OS=Mus musculus GN=Lrp1 PE=1 SV=1
PCMT1	P23506-2	17	17	118054.18	99846.76	101103.29	109940.53	112261.18	107168.29	112347	123566.94	123738.82	83881.47	0.822352566	0.084941948	108241.19	110140.5	Up	0.0250955365524905	Isoform 2 of Protein-L-isoaspartate(D-aspartate) O-methyltransferase OS=Mus musculus GN=Pcmt1
UBL4A	P21126	2	2	15975.5	22505.5	6547	45408.5	21119.5	30785	23408	9454	18320.5	NA	0.823114614	0.084539688	22311.2	20491.875	Down	-0.122716113988285	Ubiquitin-like protein 4A OS=Mus musculus GN=Ubl4a PE=2 SV=1
CSPG5	Q71M36	11	11	11225.89	21203.44	10590.67	61243	24561.33	37163.11	39615.44	10723.67	23421.78	4478	0.823304422	0.084439552	25764.866	23080.4	Down	-0.158736862078352	Chondroitin sulfate proteoglycan 5 OS=Mus musculus GN=Cspg5 PE=1 SV=2
ZFR	O88532	2	2	42274	47410	58731.5	32820	64747.5	54366	56552	26864.5	68860	NA	0.823889825	0.084130861	49196.6	51660.625	Up	0.0705064838769196	Zinc finger RNA-binding protein OS=Mus musculus GN=Zfr PE=1 SV=2
SLC27A4	Q91VE0	1	1	26263	62777	NA	131654	81769	115125	105923	NA	30360	NA	0.824225109	0.083954159	75615.75	83802.667	Up	0.148309387735193	Long-chain fatty acid transport protein 4 OS=Mus musculus GN=Slc27a4 PE=1 SV=1
ABLIM2	Q8BL65-2	20	20	26456.74	25501.21	14438.63	27395.58	30286	28321.79	31495.21	16741.53	36627	3258.21	0.824389107	0.083867755	24815.632	23288.748	Down	-0.0916161148580212	Isoform 2 of Actin-binding LIM protein 2 OS=Mus musculus GN=Ablim2
EHD4	Q9EQP2	1	1	NA	NA	NA	1860	616	922	1962	NA	NA	NA	0.825475982	0.083295559	1238	1442	Up	0.220059850026558	EH domain-containing protein 4 OS=Mus musculus GN=Ehd4 PE=1 SV=1
PYGO1	Q9D0P5	1	1	67944	105478	80964	97979	75749	105168	86574	47690	89954	NA	0.825638094	0.083210277	85622.8	82346.5	Down	-0.0562876827798819	Pygopus homolog 1 OS=Mus musculus GN=Pygo1 PE=1 SV=1
WASL	Q91YD9	8	8	7286.83	6261.33	7534.67	6530.5	7906	5990.17	5341.5	5679.67	6627.5	13770.33	0.825785203	0.083132904	7103.866	7481.834	Up	0.0747875886293999	Neural Wiskott-Aldrich syndrome protein OS=Mus musculus GN=Wasl PE=1 SV=1
PLXNA4	Q80UG2	7	7	10841.43	28168.57	14320.57	55619.86	22017.71	44178.29	45515	14137.57	18053.57	20938.86	0.825866643	0.083090075	26193.628	28564.658	Up	0.125015360028032	Plexin-A4 OS=Mus musculus GN=Plxna4 PE=1 SV=3
GNB3	Q61011	1	1	169776	210268	110412	293058	124731	248318	160781	93733	181919	NA	0.825973661	0.083033801	181649	171187.75	Down	-0.0855739557401038	Guanine nucleotide-binding protein G(I)/G(S)/G(T) subunit beta-3 OS=Mus musculus GN=Gnb3 PE=1 SV=2
SDF2L1	Q9ESP1	4	4	73511.5	71381	72742.75	46900.75	69785	62633	66910.5	69124.5	78732.25	48988	0.826130705	0.082951236	66864.2	65277.65	Down	-0.0346448579571855	Stromal cell-derived factor 2-like protein 1 OS=Mus musculus GN=Sdf2l1 PE=2 SV=2
TDRKH	Q80VL1	2	2	21026.5	32034.5	15327	28845	25849.5	28364.5	23368	35384.5	15982	NA	0.826208467	0.082910358	24616.5	25774.75	Up	0.0663327818662402	Tudor and KH domain-containing protein OS=Mus musculus GN=Tdrkh PE=1 SV=1
G6PDX	Q00612	1	1	48028	49260	66697	30770	51143	35142	43852	79476	48605	NA	0.826450681	0.082783058	49179.6	51768.75	Up	0.0740214826461168	Glucose-6-phosphate 1-dehydrogenase X OS=Mus musculus GN=G6pdx PE=1 SV=3
VTI1B	O88384	4	4	5313.33	9238.67	1398	32679.33	14877.33	24301.33	22837.33	2709	7925.33	NA	0.827029199	0.082479157	12701.332	14443.248	Up	0.185415360620541	Vesicle transport through interaction with t-SNAREs homolog 1B OS=Mus musculus GN=Vti1b PE=1 SV=1
CISD3	B1AR13	1	1	71614	51918	23010	52981	81029	63341	52285	31622	75669	43435	0.827117539	0.08243277	56110.4	53270.4	Down	-0.0749340835982243	CDGSH iron-sulfur domain-containing protein 3, mitochondrial OS=Mus musculus GN=Cisd3 PE=1 SV=1
PGM2	Q9D0F9	1	1	28467	36863	9136	118508	44640	66705	58668	28994	54418	NA	0.827227536	0.082375018	47522.8	52196.25	Up	0.135326320269183	Phosphoglucomutase-1 OS=Mus musculus GN=Pgm1 PE=1 SV=4
PRKCB	P68404	7	7	53972.14	77596.86	56266.86	118565.14	78510.57	105425.71	97652.43	62887.71	75600.86	22222.86	0.827764449	0.08209323	76982.314	72757.914	Down	-0.0814228566489195	Protein kinase C beta type OS=Mus musculus GN=Prkcb PE=1 SV=4
GSTP2	P46425	3	3	58546	46397.33	93893.67	42490	48408.67	51435	48111.67	56874	70359	75097	0.82856158	0.081675209	57947.134	60375.334	Up	0.0592219561227602	Glutathione S-transferase P 2 OS=Mus musculus GN=Gstp2 PE=2 SV=2
CRK	Q64010-2	5	5	60751	80769.6	62546	55372.6	56682.4	65696.6	73045.2	68627	60106	54896.2	0.828923627	0.081485481	63224.32	64474.2	Up	0.0282423517983682	Isoform Crk-I of Adapter molecule crk OS=Mus musculus GN=Crk
ARPP19	P56212	8	8	283773.88	241862.25	224750.88	64261.25	249210.5	163061.12	210318	213522.62	340782.62	71366.5	0.82904778	0.081420439	212771.75	199810.17	Down	-0.0906765983164689	cAMP-regulated phosphoprotein 19 OS=Mus musculus GN=Arpp19 PE=1 SV=2
SLC1A2	P43006-3	8	8	9296.88	38477.38	15247.62	162623.12	43302	96518.12	72574.25	30934.62	26148.5	6380.25	0.829600107	0.0811312	53789.4	46511.148	Down	-0.209745347042345	Isoform Glt-1B of Excitatory amino acid transporter 2 OS=Mus musculus GN=Slc1a2
CROCC	Q8CJ40	12	12	29036.33	34612.17	19374.83	31952.33	33078.83	38874.42	32263.67	9285.33	36621.92	24256.33	0.82973282	0.081061731	29610.898	28260.334	Down	-0.0673497267870456	Rootletin OS=Mus musculus GN=Crocc PE=1 SV=2
DNAJC11	Q5U458	1	1	4202	7240	NA	19290	4248	11506	8788	NA	2966	NA	0.830114959	0.08086176	8745	7753.3333	Down	-0.173641693169591	DnaJ homolog subfamily C member 11 OS=Mus musculus GN=Dnajc11 PE=2 SV=2
ABLIM2	Q8BL65-5	18	18	27880.65	27456.41	13428.82	33663.88	31241.12	32171.76	34410.71	18574.35	38862.59	985.12	0.830244137	0.080794182	26734.176	25000.906	Down	-0.0967048335086451	Isoform 5 of Actin-binding LIM protein 2 OS=Mus musculus GN=Ablim2
L2HGDH	Q91YP0	2	2	38465	44356	30148.5	53471	61093.5	81652.5	55387	11681.5	47366	NA	0.83140406	0.080187859	45506.8	49021.75	Up	0.107339846318512	L-2-hydroxyglutarate dehydrogenase, mitochondrial OS=Mus musculus GN=L2hgdh PE=1 SV=1
CCDC88A	Q5SNZ0-3	12	12	12459.92	13981.25	12338.75	23722.33	19460.92	23615.25	23824.08	12831	22109.83	4464.75	0.832056621	0.079847119	16392.634	17368.982	Up	0.0834655117593523	Isoform 3 of Girdin OS=Mus musculus GN=Ccdc88a
ERC1	Q99MI1	23	23	28966.22	27465.57	22226.83	30949.17	29856.78	31472.04	33042.91	23811.04	38587.74	17073.57	0.832132813	0.079807352	27892.914	28797.46	Up	0.0460429063674747	ELKS/Rab6-interacting/CAST family member 1 OS=Mus musculus GN=Erc1 PE=1 SV=1
FAM134C	Q9CQV4	4	4	NA	1107.33	NA	4075.33	825.67	2648	1665	NA	892.67	NA	0.832444386	0.079644771	2002.7767	1735.2233	Down	-0.206880194530467	Protein FAM134C OS=Mus musculus GN=Fam134c PE=1 SV=1
CAMK2A	P11798	54	54	64473.04	85728.66	75103.68	160012.11	87417.98	113302.72	128750.36	97524.81	83110.68	71754.4	0.832479334	0.079626539	94547.094	98888.594	Up	0.0647710111948509	Calcium/calmodulin-dependent protein kinase type II subunit alpha OS=Mus musculus GN=Camk2a PE=1 SV=2
RAP2B	P61226	1	1	4712	NA	NA	14738	8853	12809	13541	NA	4702	NA	0.832502413	0.079614499	9434.3333	10350.667	Up	0.133731211848217	Ras-related protein Rap-2b OS=Mus musculus GN=Rap2b PE=1 SV=1
APLP1	Q03157	5	5	10114.4	12096.6	6706	14680.2	11593.4	12463	12218	8260	14189.4	5859.8	0.832740475	0.079490326	11038.12	10598.04	Down	-0.0586969972229578	Amyloid-like protein 1 OS=Mus musculus GN=Aplp1 PE=1 SV=1
SHANK1	D3YZU1	36	36	31628.94	32380.42	34751.44	28503.56	37243.97	30991.28	36690.61	28407.42	41626.78	22834.28	0.832796074	0.079461331	32901.666	32110.074	Down	-0.0351346478689703	SH3 and multiple ankyrin repeat domains protein 1 OS=Mus musculus GN=Shank1 PE=1 SV=1
RAB14	Q91V41	5	5	30978.6	30377.6	47867.4	27671.8	37548.6	34114.6	29027.2	42257.2	33875.4	39905.4	0.833042284	0.079332954	34888.8	35835.96	Up	0.0386440261056512	Ras-related protein Rab-14 OS=Mus musculus GN=Rab14 PE=1 SV=3
FAM171A2	A2A699	12	12	20008.9	22749.1	19132.2	36355.3	31610.6	37737.4	39277.6	18970.9	31646.8	9516.2	0.833539953	0.079073579	25971.22	27429.78	Up	0.0788292648104937	Protein FAM171A2 OS=Mus musculus GN=Fam171a2 PE=1 SV=1
PPP2R1B	Q7TNP2	2	2	16117	13948	15747.5	31401.5	19806	22264	17394.5	4578	36741	22605	0.834823942	0.078405104	19404	20716.5	Up	0.0944261993075013	Serine/threonine-protein phosphatase 2A 65 kDa regulatory subunit A beta isoform OS=Mus musculus GN=Ppp2r1b PE=1 SV=2
CAND1	Q6ZQ38	6	6	11140.83	20564.83	8681.33	61412.5	18247.17	31213	30533.5	4232.5	20099	NA	0.834955229	0.078336811	24009.332	21519.5	Down	-0.15795070708334	Cullin-associated NEDD8-dissociated protein 1 OS=Mus musculus GN=Cand1 PE=2 SV=2
STX16	Q8BVI5	6	6	6644.4	10955.6	4340.2	11807.4	11731.4	13492.4	14656.8	1158.8	10125.2	9092.6	0.835134359	0.078243648	9095.8	9705.16	Up	0.0935514651597205	Syntaxin-16 OS=Mus musculus GN=Stx16 PE=1 SV=3
ATP5O	Q9DB20	25	25	181504.88	181792.36	198851.4	211840.08	189393.4	185068.08	176764.52	171450.32	185676.36	264193.92	0.835326193	0.078143901	192676.42	196630.64	Up	0.029308093951044	ATP synthase subunit O, mitochondrial OS=Mus musculus GN=Atp5o PE=1 SV=1
ACSF2	Q8VCW8	6	6	16960.33	15460.83	17188.33	29119.33	17534.67	21735.67	17661.17	17883.83	22045	19841.33	0.835530156	0.078037871	19252.698	19833.4	Up	0.0428713833060981	Acyl-CoA synthetase family member 2, mitochondrial OS=Mus musculus GN=Acsf2 PE=1 SV=1
ACTG2	P63268	59	59	307024.63	388477.36	366732.1	414800.12	329982.76	384206.9	466670.2	294266.51	338922.92	360159.61	0.835633369	0.077984226	361403.39	368845.23	Up	0.0294055157974199	Actin, gamma-enteric smooth muscle OS=Mus musculus GN=Actg2 PE=1 SV=1
ACTA1	P68134	61	61	297333.75	376279.05	355580.66	401698.52	319603.02	372144.87	451873.95	285356.75	328351.33	348669.3	0.835986767	0.077800597	350099	357279.24	Up	0.029289146441087	Actin, alpha skeletal muscle OS=Mus musculus GN=Acta1 PE=1 SV=1
EFHD1	Q9D4J1	1	1	7585	6625	6573	19047	5879	12071	7175	NA	10025	NA	0.837458762	0.077036569	9141.8	9757	Up	0.093959371592521	EF-hand domain-containing protein D1 OS=Mus musculus GN=Efhd1 PE=2 SV=1
CORO1A	O89053	22	22	82340.86	71319.59	71224.09	87773	77120.68	74283.32	78645.27	60041.27	91408.95	79057.36	0.838202768	0.07665091	77955.644	76687.234	Down	-0.0236670434336924	Coronin-1A OS=Mus musculus GN=Coro1a PE=1 SV=5
CEND1	Q9JKC6	5	5	70689.2	95058.8	82809.8	121112.8	86945.4	89984	110201.6	72694.6	85949.2	109475.6	0.838284776	0.076608421	91323.2	93661	Up	0.036467028452628	Cell cycle exit and neuronal differentiation protein 1 OS=Mus musculus GN=Cend1 PE=1 SV=1
TCEB1	P83940	2	2	119300.5	105746.5	107678	198881	109760.5	133373	139122.5	134956	108000	105336	0.8383887	0.076554584	128273.3	124157.5	Down	-0.0470494924522609	Transcription elongation factor B polypeptide 1 OS=Mus musculus GN=Tceb1 PE=1 SV=1
PHGDH	Q61753	33	33	30345.12	32112.58	34732.97	40554.36	40102.48	43545.79	44141.52	25918.48	42103.45	27055.48	0.8384332	0.076531533	35569.502	36552.944	Up	0.0393468308210433	D-3-phosphoglycerate dehydrogenase OS=Mus musculus GN=Phgdh PE=1 SV=3
GPSM1	Q6IR34-3	7	7	11143.43	14107.14	8492.86	17429.57	14444.71	15749.43	17109.43	3695.29	20515.71	4556.86	0.839613737	0.075920465	13123.542	12325.344	Down	-0.0905292383249283	Isoform 3 of G-protein-signaling modulator 1 OS=Mus musculus GN=Gpsm1
H2AFY	Q9QZQ8	1	1	220155	128096	197058	64882	156715	113347	101765	64615	142245	418265	0.840261737	0.075585412	153381.2	168047.4	Up	0.131746559770875	Core histone macro-H2A.1 OS=Mus musculus GN=H2afy PE=1 SV=3
ACSBG1	Q99PU5	2	2	22475	26348	15039	87993.5	27198	52501	44948.5	28974	29056.5	NA	0.84028794	0.075571869	35810.7	38870	Up	0.118266388050834	Long-chain-fatty-acid--CoA ligase ACSBG1 OS=Mus musculus GN=Acsbg1 PE=1 SV=1
PEA15A	Q62048	7	7	62448	88129.29	81291.29	93068.14	80054.29	90089.29	83495.57	113597	69359.29	33388.86	0.840397018	0.075515497	80998.202	77986.002	Down	-0.0546746909056306	Astrocytic phosphoprotein PEA-15 OS=Mus musculus GN=Pea15 PE=1 SV=1
DTX3	Q80V91	1	1	NA	NA	NA	2499	1494	2423	1208	NA	NA	NA	0.840420649	0.075503285	1996.5	1815.5	Down	-0.137106141832841	Probable E3 ubiquitin-protein ligase DTX3 OS=Mus musculus GN=Dtx3 PE=2 SV=2
CLTC	Q68FD5	23	23	16732.14	29658.45	10749.55	71996.77	26042	45926.27	41038.55	15862.18	21739.5	17587.41	0.840890468	0.075260571	31035.782	28430.782	Down	-0.126478716100494	Clathrin heavy chain 1 OS=Mus musculus GN=Cltc PE=1 SV=3
HPCAL4	Q8BGZ1	2	2	39308.5	63153	50236	71160	50797	62314.5	55849.5	59890.5	34654	NA	0.841058641	0.075173723	54930.9	53177.125	Down	-0.046812148910822	Hippocalcin-like protein 4 OS=Mus musculus GN=Hpcal4 PE=2 SV=3
SPTBN1	Q62261	185	185	17842.2	38781.97	18918.14	116505.83	41414.12	66469.08	67535.92	22599.95	34471.29	20642.55	0.841425276	0.074984446	46692.452	42343.758	Down	-0.141040038105356	Spectrin beta chain, non-erythrocytic 1 OS=Mus musculus GN=Sptbn1 PE=1 SV=2
MAP2K6	P70236	1	1	832	NA	NA	4187	1137	2936	978	NA	1445	NA	0.841506921	0.074942308	2052	1786.3333	Down	-0.200029415616917	Dual specificity mitogen-activated protein kinase kinase 6 OS=Mus musculus GN=Map2k6 PE=1 SV=1
IDH2	P54071	5	5	52835.8	53755.2	42710.4	49943.8	48121.6	54728.4	50261.8	13167.8	62207.8	57457.4	0.842064208	0.074654792	49473.36	47564.64	Down	-0.0567624249923881	Isocitrate dehydrogenase [NADP], mitochondrial OS=Mus musculus GN=Idh2 PE=1 SV=3
TAGLN2	Q9WVA4	3	3	27709.67	28380.33	8496	90852.67	28076	49893.67	35875.33	37568.33	35675.67	NA	0.842264542	0.074551482	36702.934	39753.25	Up	0.115177416332774	Transgelin-2 OS=Mus musculus GN=Tagln2 PE=1 SV=4
HADH	Q61425	1	1	43175	41962	69309	28937	33564	35172	43593	NA	46535	NA	0.842323419	0.074521124	43389.4	41766.667	Down	-0.0549906275013078	Hydroxyacyl-coenzyme A dehydrogenase, mitochondrial OS=Mus musculus GN=Hadh PE=1 SV=2
LRBA	Q9ESE1-3	1	1	NA	NA	NA	6161	1222	3353	2784	NA	NA	NA	0.842844652	0.074252465	3691.5	3068.5	Down	-0.266673575041806	Isoform 3 of Lipopolysaccharide-responsive and beige-like anchor protein OS=Mus musculus GN=Lrba
MBD3	Q9Z2D8	2	2	28900.5	20822	40246	16052.5	30348	27242.5	23301	NA	28490.5	NA	0.842991781	0.07417666	27273.8	26344.667	Down	-0.0500047961396604	Methyl-CpG-binding domain protein 3 OS=Mus musculus GN=Mbd3 PE=1 SV=1
NUCB2	P81117	4	4	25961.33	30010	10914.33	51786.33	27645.33	41374	35236.67	5198	44457.67	NA	0.843107697	0.074116946	29263.464	31566.585	Up	0.10929763702	Nucleobindin-2 OS=Mus musculus GN=Nucb2 PE=1 SV=2
ARHGAP26	Q6ZQ82	1	1	10657	18977	19398	38648	19524	23343	27688	10372	17900	34193	0.843286256	0.074024978	21440.8	22699.2	Up	0.0822827160462543	Rho GTPase-activating protein 26 OS=Mus musculus GN=Arhgap26 PE=1 SV=3
2010107E04RIK	P56379	1	1	522804	211877	303930	193646	311819	228444	257860	222826	248426	505618	0.843598213	0.073864349	308815.2	292634.8	Down	-0.0776424200150644	6.8 kDa mitochondrial proteolipid OS=Mus musculus GN=Mp68 PE=2 SV=1
SLC25A12	Q8BH59	7	7	81761.71	159756.14	82013.57	311208.86	150795.57	229415	227859.43	126264.71	109441.71	35573	0.843853482	0.073732953	157107.17	145710.77	Down	-0.108641507274478	Calcium-binding mitochondrial carrier protein Aralar1 OS=Mus musculus GN=Slc25a12 PE=1 SV=1
ADAM23	Q9R1V7-4	7	7	37827	45991.57	29969.29	46934.43	43688.71	49894.57	49425.86	40951.14	41970.43	14809.86	0.84398262	0.073666497	40882.2	39410.372	Down	-0.0528974669907469	Isoform Delta of Disintegrin and metalloproteinase domain-containing protein 23 OS=Mus musculus GN=Adam23
ERC1	Q99MI1-2	24	24	29549.17	27850	22714.58	30561.79	29927.79	31270	33418.04	24745.83	39105.83	16362.17	0.844146775	0.073582034	28120.666	28980.374	Up	0.0434454503074603	Isoform 2 of ELKS/Rab6-interacting/CAST family member 1 OS=Mus musculus GN=Erc1
SLC12A6	Q924N4-2	1	1	5561	14589	2978	54570	14052	35829	21837	9955	14706	NA	0.844327572	0.073489029	18350	20581.75	Up	0.165585592097517	Isoform 2 of Solute carrier family 12 member 6 OS=Mus musculus GN=Slc12a6
RPL13	P47963	14	14	148041.29	148938.64	143849.93	71028.29	117450	111819.43	143514.64	93538.57	188040.57	66212.36	0.844509304	0.073395561	125861.63	120625.11	Down	-0.061308226258665	60S ribosomal protein L13 OS=Mus musculus GN=Rpl13 PE=2 SV=3
HIP1R	Q9JKY5	14	14	7970.64	20376.93	10649.79	47171.36	23243.57	34786.21	29742.29	10710	15432.57	10048.29	0.845473906	0.072899791	21882.458	20143.872	Down	-0.119433780544846	Huntingtin-interacting protein 1-related protein OS=Mus musculus GN=Hip1r PE=1 SV=2
USP5	P56399	10	10	13850.2	19228.7	16276.8	28150.1	18609.3	23347.1	22290.3	16507.1	15805	21058.2	0.846199058	0.072527462	19223.02	19801.54	Up	0.0427776294772893	Ubiquitin carboxyl-terminal hydrolase 5 OS=Mus musculus GN=Usp5 PE=1 SV=1
PLS3	Q99K51	2	2	55330	51179.5	83688	49015.5	68335.5	56820	67136.5	8846	71258	123180.5	0.846735135	0.072252419	61509.7	65448.2	Up	0.0895395752242403	Plastin-3 OS=Mus musculus GN=Pls3 PE=1 SV=3
UBTF	P25976	1	1	1308	1405	NA	5906	2426	4542	3058	NA	1529	NA	0.846863987	0.072186335	2761.25	3043	Up	0.140172820271959	Nucleolar transcription factor 1 OS=Mus musculus GN=Ubtf PE=1 SV=1
ATAD1	Q9D5T0	5	5	12297.5	15923.5	6960.25	54496.25	21797.25	35624	28223	14372.5	18743.25	NA	0.846939722	0.072147498	22294.95	24240.688	Up	0.120713652071585	ATPase family AAA domain-containing protein 1 OS=Mus musculus GN=Atad1 PE=1 SV=1
SELM	Q8VHC3	1	1	11614	61400	18909	104261	40876	59800	41229	NA	29811	NA	0.847194621	0.07201681	47412	43613.333	Down	-0.120482993449842	Selenoprotein M OS=Mus musculus GN=Selm PE=1 SV=3
SNAP47	Q8R570	4	4	16454	19636.5	10033.5	37005.25	27937.5	33966.25	30359	4110.75	27031.25	NA	0.847425218	0.071898616	22213.35	23866.813	Up	0.103578919655567	Synaptosomal-associated protein 47 OS=Mus musculus GN=Snap47 PE=1 SV=1
QK	Q9QYS9-3	4	4	91861.25	107068.25	82382.25	84556.75	96678.5	95581	77876	97407	114426	68027.5	0.847959282	0.071625002	92509.4	90663.5	Down	-0.0290781106737471	Isoform 3 of Protein quaking OS=Mus musculus GN=Qki
SLC4A3	P16283-3	2	2	14328.5	14016.5	6734.5	37442	24124.5	29376	29133	15468	22227	7131	0.849038036	0.071072853	19329.2	20667	Up	0.0965470555723092	Isoform 14-AE3 of Anion exchange protein 3 OS=Mus musculus GN=Slc4a3
TOMM34	Q9CYG7-2	2	2	15152.5	23518	11725	19654.5	25287.5	21930.5	20263.5	6883	23549	NA	0.849565941	0.070802907	19067.5	18156.5	Down	-0.0706295763854267	Isoform 2 of Mitochondrial import receptor subunit TOM34 OS=Mus musculus GN=Tomm34
TRIM9	Q8C7M3-3	9	9	21855.11	22291.22	15829	21353.33	23186.89	25290.33	30821	16155.11	36837.89	1724.22	0.849615755	0.070777443	20903.11	22165.71	Up	0.0846119699887259	Isoform 3 of E3 ubiquitin-protein ligase TRIM9 OS=Mus musculus GN=Trim9
AAMDC	Q8R0P4	1	1	30664	38600	54090	8976	29856	24526	31058	NA	47701	NA	0.849996191	0.070583021	32437.2	34428.333	Up	0.0859470515418598	Mth938 domain-containing protein OS=Mus musculus GN=Aamdc PE=1 SV=1
RHOJ	Q9ER71-2	1	1	45906	55905	48316	90512	43690	65441	56118	51428	55653	46554	0.850453184	0.070349589	56865.8	55038.8	Down	-0.0471122379187461	Isoform 2 of Rho-related GTP-binding protein RhoJ OS=Mus musculus GN=Rhoj
PRDX4	O08807	2	2	91488.5	100767.5	38389	119535	94003	97169.5	108649	32271	99802	NA	0.850503219	0.070324038	88836.6	84472.875	Down	-0.072666024972597	Peroxiredoxin-4 OS=Mus musculus GN=Prdx4 PE=1 SV=1
CAPRIN1	Q60865	6	6	20637.33	23017.67	7782	35268.83	24055.67	26279.5	24672	3406.83	28535.5	NA	0.851233148	0.069951473	22152.3	20723.458	Down	-0.0961917745770887	Caprin-1 OS=Mus musculus GN=Caprin1 PE=1 SV=2
KIF2A	P28740-2	17	17	28335.35	35629.29	36016.06	37085.59	33246.12	36599.65	39660.29	24559.53	33028.18	39598.29	0.85161299	0.069757723	34062.482	34689.188	Up	0.0263025082032706	Isoform 2 of Kinesin-like protein KIF2A OS=Mus musculus GN=Kif2a
PITPNA	P53810	7	7	88042.86	90739.43	173226.57	74937.86	90846.14	87166.14	103189.43	58610.57	104469.29	191833.14	0.8518223	0.069650995	103558.57	109053.71	Up	0.0745919273841375	Phosphatidylinositol transfer protein alpha isoform OS=Mus musculus GN=Pitpna PE=1 SV=2
BCL11A	Q9QYE3-12	1	1	22835	15025	NA	14094	24913	20478	16283	17863	19937	NA	0.852503736	0.069303709	19216.75	18640.25	Down	-0.0439431540956727	Isoform 4 of B-cell lymphoma/leukemia 11A OS=Mus musculus GN=Bcl11a
C1QBP	O35658	17	17	36691.12	36042.65	35607.65	31702.35	35553.65	33901.76	34058.59	24725.41	41739.35	38249	0.852719939	0.069193582	35119.484	34534.822	Down	-0.0242198602412977	Complement component 1 Q subcomponent-binding protein, mitochondrial OS=Mus musculus GN=C1qbp PE=1 SV=1
CLPP	O88696	3	3	6330	16302	4065	45175	11801.33	25989.67	14704.33	NA	3800	NA	0.852736838	0.069184975	16734.666	14831.333	Down	-0.174191455618785	Putative ATP-dependent Clp protease proteolytic subunit, mitochondrial OS=Mus musculus GN=Clpp PE=1 SV=1
TOM1L2	Q5SRX1	14	14	21979.91	26137	19855.18	31552.73	25841.18	29804.55	29397.73	14947.09	34912.82	11436.73	0.852976039	0.069063168	25073.2	24099.784	Down	-0.0571259177487618	TOM1-like protein 2 OS=Mus musculus GN=Tom1l2 PE=2 SV=1
CALCOCO1	Q8CGU1	1	1	29271	13527	35035	28796	28797	37465	24132	NA	23103	NA	0.853360315	0.068867558	27085.2	28233.333	Up	0.0598947249742646	Calcium-binding and coiled-coil domain-containing protein 1 OS=Mus musculus GN=Calcoco1 PE=1 SV=2
SARM1	Q6PDS3-3	1	1	6327	10000	2152	11382	7395	11794	8700	6259	4750	NA	0.853889215	0.068598472	7451.2	7875.75	Up	0.0799445292143222	Isoform 3 of Sterile alpha and TIR motif-containing protein 1 OS=Mus musculus GN=Sarm1
CNTN1	P12960	30	30	19980.53	38993.6	16973.73	72602.23	33381.23	49940.63	60097.67	16717.53	28846.9	13475.17	0.853948675	0.068568231	36386.264	33815.58	Down	-0.10570582959845	Contactin-1 OS=Mus musculus GN=Cntn1 PE=1 SV=1
GAA	P70699	4	4	19231.75	23451	22610.75	39614.5	19218.25	36373.75	26626.5	12314.25	29245	13882.5	0.854029371	0.068527193	24825.25	23688.4	Down	-0.0676274892906533	Lysosomal alpha-glucosidase OS=Mus musculus GN=Gaa PE=1 SV=2
JAKMIP1	Q8BVL9	1	1	NA	11924	NA	32212	24909	29406	12282	NA	NA	NA	0.854510323	0.068282686	23015	20844	Down	-0.1429422826797	Janus kinase and microtubule-interacting protein 1 OS=Mus musculus GN=Jakmip1 PE=1 SV=2

COX5B	P19536	33	33	176353.73	156932.27	167924.79	203301.33	187854.79	183407.97	162170.64	194228.97	196401.82	143965.79	0.854514274	0.068280678	178473.38	176035.04	Down	-0.0198463115307473	Cytochrome c oxidase subunit 5B, mitochondrial OS=Mus musculus GN=Cox5b PE=1 SV=1
ACTR2	P61161	15	15	56442.87	85087.93	59397.4	163464.2	97376.33	118066.33	152695.47	46667.73	83990.47	32413.67	0.854597071	0.0682386	92353.746	86766.734	Down	-0.0900284546893298	Actin-related protein 2 OS=Mus musculus GN=Actr2 PE=1 SV=1
DCTN4	Q8CBY8	12	12	49573.83	50075	53569.25	42935.08	55006.25	59212.92	58034.83	38609.08	70261.33	32106.25	0.855814407	0.067620407	50231.882	51644.882	Up	0.0400220569253134	Dynactin subunit 4 OS=Mus musculus GN=Dctn4 PE=1 SV=1
ERC1	Q99MI1-4	6	6	29804.33	29450	13507	39081.17	27543.83	33399	32188.17	4510	35017.33	NA	0.856110459	0.067470197	27877.266	26278.625	Down	-0.0851992888690483	Isoform 3 of ELKS/Rab6-interacting/CAST family member 1 OS=Mus musculus GN=Erc1
HMGCL	P38060	7	7	26193.57	27329.29	31469.71	26812.57	29491.14	29627.71	31050.57	25513.57	40735.29	18041	0.856257592	0.067395565	28259.256	28993.628	Up	0.0370123870936312	Hydroxymethylglutaryl-CoA lyase, mitochondrial OS=Mus musculus GN=Hmgcl PE=1 SV=2
TIMM44	O35857	2	2	39095	62239	25160	163239	62164	123776	100059.5	22613	59823	NA	0.856432393	0.067306915	70379.4	76567.875	Up	0.121586003683224	Mitochondrial import inner membrane translocase subunit TIM44 OS=Mus musculus GN=Timm44 PE=1 SV=2
LUC7L	Q9CYI4	2	2	5756.5	5388.5	1339	9589	5013	9047	5975	1701	6453.5	NA	0.856888473	0.067075699	5417.2	5794.125	Up	0.0970434537483608	Putative RNA-binding protein Luc7-like 1 OS=Mus musculus GN=Luc7l PE=2 SV=2
CNPY3	Q9DAU1-2	3	3	7532.33	10472	7537	5746.67	7177	8875.33	8567.33	1735.33	10032.33	NA	0.857087995	0.066974588	7693	7302.58	Down	-0.075140050432906	Isoform 2 of Protein canopy homolog 3 OS=Mus musculus GN=Cnpy3
SRCIN1	Q9QWI6	82	82	59325.18	65182.7	51299.72	67399.91	69904.56	68083.8	75396.65	47804.68	86965.19	43262.57	0.857465595	0.066783297	62622.414	64302.578	Up	0.0381974555415609	SRC kinase signaling inhibitor 1 OS=Mus musculus GN=Srcin1 PE=1 SV=2
NCAM2	O35136	13	13	19436.69	54090.54	12833.23	119296.92	48024.15	83831.15	91568.92	41909.62	48543.54	10079.08	0.857855838	0.066585689	50736.306	55186.462	Up	0.121295913681875	Neural cell adhesion molecule 2 OS=Mus musculus GN=Ncam2 PE=2 SV=1
CAMK2N1	Q6QWF9	3	3	64845.33	49080.67	71866.67	58347	57103	50466.67	60533.67	18557	109879	47314.67	0.858463663	0.066278083	60248.534	57350.202	Down	-0.0711275695929638	Calcium/calmodulin-dependent protein kinase II inhibitor 1 OS=Mus musculus GN=Camk2n1 PE=1 SV=1
RIMS2	Q9EQZ7-3	3	3	3382.67	3570	NA	8512.33	14172.33	9143.33	8572.33	NA	6065.67	NA	0.858703173	0.066156932	7409.3325	7927.11	Up	0.0974514186865284	Isoform 3 of Regulating synaptic membrane exocytosis protein 2 OS=Mus musculus GN=Rims2
GNB1	P62874	55	55	77820.33	88620.67	68263.62	154044.42	82541.92	113845.27	94790.23	106486.88	93613.04	44590.9	0.858825344	0.066095148	94258.192	90665.264	Down	-0.05606808264227	Guanine nucleotide-binding protein G(I)/G(S)/G(T) subunit beta-1 OS=Mus musculus GN=Gnb1 PE=1 SV=3
VPS16	Q920Q4	1	1	NA	2942	NA	11044	2325	5835	9118	NA	3238	NA	0.859837358	0.06558369	5437	6063.6667	Up	0.157379619376165	Vacuolar protein sorting-associated protein 16 homolog OS=Mus musculus GN=Vps16 PE=1 SV=3
ANLN	Q8K298	1	1	3011	5178	1841	5220	3666	5353	3111	NA	2248	NA	0.859989019	0.065507094	3783.2	3570.6667	Down	-0.0834135869672877	Actin-binding protein anillin OS=Mus musculus GN=Anln PE=1 SV=2
RPL10L	P86048	18	18	59553.94	65450.88	57772.25	44747.12	61947.88	51205.06	63342.5	55571.94	68624.69	45798.69	0.860060821	0.065470836	57894.414	56908.576	Down	-0.0247780751116814	60S ribosomal protein L10-like OS=Mus musculus GN=Rpl10l PE=2 SV=1
RAB3C	P62823	5	5	16612	29090.2	13299	72603.8	30982.2	43446.6	36102	NA	24573.4	NA	0.860797326	0.065099091	32517.44	34707.333	Up	0.0940268400613261	Ras-related protein Rab-3C OS=Mus musculus GN=Rab3c PE=1 SV=1
DDX39B	Q9Z1N5	2	2	14275	19586	NA	44473	19837	31295.5	13406	NA	NA	NA	0.861439699	0.064775118	24542.75	22350.75	Down	-0.13497366796598	Spliceosome RNA helicase Ddx39b OS=Mus musculus GN=Ddx39b PE=1 SV=1
RIMS1	Q99NE5-7	24	24	41430.2	47118.15	40671.15	46906.5	55536.8	52996.2	56711.5	45739.65	63936.6	19692.85	0.8615039	0.064742752	46332.56	47815.36	Up	0.0454477403918686	Isoform 7 of Regulating synaptic membrane exocytosis protein 1 OS=Mus musculus GN=Rims1
SPR	Q64105	3	3	21976.33	22978.33	28852.33	26453	26629.33	29482	25959.33	10959.33	25792	39006	0.861891037	0.064547636	25377.864	26239.732	Up	0.048182339274744	Sepiapterin reductase OS=Mus musculus GN=Spr PE=1 SV=1
GOLGA1	Q9CW79	1	1	10663	12832	NA	9406	12770	12127	7493	NA	16153	NA	0.862513811	0.064233942	11417.75	11924.333	Up	0.0626302312385888	Golgin subfamily A member 1 OS=Mus musculus GN=Golga1 PE=1 SV=2
CAPN1	O35350	1	1	NA	3034	NA	15490	3892	9103	7435	NA	NA	NA	0.862656329	0.064162187	7472	8269	Up	0.146218414582043	Calpain-1 catalytic subunit OS=Mus musculus GN=Capn1 PE=1 SV=1
CDH4	P39038	1	1	2858	3706	5832	12294	6864	9567	9777	2350	5304	NA	0.862776371	0.064101757	6310.8	6749.5	Up	0.0969577292535359	Cadherin-4 OS=Mus musculus GN=Cdh4 PE=2 SV=1
AFG3L1	Q920A7	1	1	13867	18378	NA	47114	15450	31080	24713	NA	19999	NA	0.863173722	0.06390179	23702.25	25264	Up	0.0920590589692598	AFG3-like protein 1 OS=Mus musculus GN=Afg3l1 PE=2 SV=2
LNPK1	Q7TQ95	1	1	6031	6916	9875	33868	18332	18685	13842	NA	9322	NA	0.863314998	0.063830714	15004.4	13949.667	Down	-0.105154980567862	Protein lunapark OS=Mus musculus GN=Lnp PE=1 SV=1
SLC4A4	O88343-2	4	4	36894.5	68230.25	14846.5	123514.25	75321.5	79638	71424.25	39952.25	49158.5	NA	0.863536569	0.063719266	63761.4	60043.25	Down	-0.0866812379623636	Isoform 2 of Electrogenic sodium bicarbonate cotransporter 1 OS=Mus musculus GN=Slc4a4
MOG	Q61885	2	2	5040	28835.5	4861.5	116813.5	41509	72161	47667	11029	8485	NA	0.863667431	0.063653458	39411.9	34835.5	Down	-0.178073031040419	Myelin-oligodendrocyte glycoprotein OS=Mus musculus GN=Mog PE=1 SV=1
DCLK2	Q6PGN3-3	8	8	19676.88	29989.62	14440.75	42221.38	35758.38	36738.88	43926.38	12926.12	33124.5	7657.5	0.863897333	0.063537867	28417.402	26874.676	Down	-0.0805273035768093	Isoform 3 of Serine/threonine-protein kinase DCLK2 OS=Mus musculus GN=Dclk2
APOE	P08226	12	12	32875.09	43281.55	13578.36	57847	24178.82	49572.91	35447.55	14263.18	30615.36	NA	0.863947394	0.063512701	34352.164	32474.75	Down	-0.0810825644351651	Apolipoprotein E OS=Mus musculus GN=Apoe PE=1 SV=2
CRELD1	Q91XD7	5	5	14447	18880.6	6561.4	45047.2	30482	38371.8	40576.6	14339.8	28242.2	2586.2	0.864557649	0.063206042	23083.64	24823.32	Up	0.104825345112003	Cysteine-rich with EGF-like domain protein 1 OS=Mus musculus GN=Creld1 PE=2 SV=1
ABLIM2	Q8BL65-4	21	21	26416.45	26376.2	14418.85	30278.55	30539.25	29916.55	33012.1	18689.25	37265.95	3095.3	0.865201269	0.062882852	25605.86	24395.83	Down	-0.0698394465759923	Isoform 4 of Actin-binding LIM protein 2 OS=Mus musculus GN=Ablim2
APBA2	P98084	4	4	2214.67	3160.67	409	5558.33	2649.67	4214.67	2621.33	NA	2111.33	NA	0.866089909	0.062437022	2798.468	2982.4433	Up	0.0918574759990631	Amyloid beta A4 precursor protein-binding family A member 2 OS=Mus musculus GN=Apba2 PE=1 SV=2
SMPD3	Q9JJY3	3	3	3824	24178.33	6856.33	49080.33	25154.67	41801.67	42220	16149.67	14391.67	4238.67	0.866469733	0.062246603	21818.732	23760.336	Up	0.122987976157355	Sphingomyelin phosphodiesterase 3 OS=Mus musculus GN=Smpd3 PE=1 SV=1
PSD3	Q2PFD7-3	14	14	98866.43	99938.29	86039.79	130006.43	87489.29	97548.71	104619	104688.5	121447.57	63278.43	0.867144883	0.061908335	100468.05	98316.442	Down	-0.0312321115274513	Isoform 3 of PH and SEC7 domain-containing protein 3 OS=Mus musculus GN=Psd3
CAMK2G	Q923T9-3	26	26	54521.12	78711.96	45222.77	138641.73	81727.58	98886.19	106099.23	58852.27	67639.92	50272.15	0.86720314	0.061879158	79765.032	76349.952	Down	-0.0631291754158806	Isoform 3 of Calcium/calmodulin-dependent protein kinase type II subunit gamma OS=Mus musculus GN=Camk2g
SLC1A2	P43006	20	20	13454.45	32078	14116.6	106840	36816.2	67709.4	59492.7	21694.4	26726.3	9947.8	0.867831891	0.061564395	40661.05	37114.12	Down	-0.131679308715406	Excitatory amino acid transporter 2 OS=Mus musculus GN=Slc1a2 PE=1 SV=1
GIT1	Q68FF6	42	42	21181.93	24443.34	14775.41	20878.39	27231.15	27310.39	27414.51	14471.93	25946.98	16393.73	0.868054911	0.061452802	21702.044	22307.508	Up	0.039698428456118	ARF GTPase-activating protein GIT1 OS=Mus musculus GN=Git1 PE=1 SV=1
CSNK1D	Q9DC28-2	3	3	19606.67	36138	13380.67	33204.33	37515	36994	38556.33	12687	49129.67	10290.33	0.868189479	0.061385481	27968.934	29531.466	Up	0.0784277080729485	Isoform 2 of Casein kinase I isoform delta OS=Mus musculus GN=Csnk1d
BCR	Q6PAJ1	4	4	15445	17692.25	44738.25	10560.75	14895.5	13800.25	25074.5	32813.75	16139.75	NA	0.868740174	0.061110095	20666.35	21957.063	Up	0.0874014491812761	Breakpoint cluster region protein OS=Mus musculus GN=Bcr PE=1 SV=3
PPME1	Q8BVQ5	1	1	33394	28760	67622	37258	32932	49935	38358	24294	54032	NA	0.868831838	0.061064273	39993.2	41654.75	Up	0.0587262982635376	Protein phosphatase methylesterase 1 OS=Mus musculus GN=Ppme1 PE=1 SV=5
PGD	Q9DCD0	2	2	4912.5	16196	673	41417	9648.5	25122.5	16680.5	NA	6545	NA	0.868856562	0.061051915	14569.4	16116	Up	0.145552245334562	6-phosphogluconate dehydrogenase, decarboxylating OS=Mus musculus GN=Pgd PE=1 SV=3
RPS15	P62843	7	7	42905.71	48407.14	43335.57	73879.86	45767.14	64783.86	49385.71	37278.57	56934	39244.29	0.868967814	0.060996309	50859.084	49525.286	Down	-0.0383401720786818	40S ribosomal protein S15 OS=Mus musculus GN=Rps15 PE=2 SV=2
CBY1	Q9D1C2	1	1	1017	2597	NA	1447	2135	680	NA	NA	2526	NA	0.869153704	0.060903414	1799	1603	Down	-0.166420761096934	Protein chibby homolog 1 OS=Mus musculus GN=Cby1 PE=2 SV=1
TOM1L2	Q5SRX1-2	15	15	20980.75	24821.17	18690.17	30706.08	24869	28624.25	28252.75	15443.08	33178.75	10483.67	0.870645518	0.060158631	24013.434	23196.5	Down	-0.0499345874795558	Isoform 2 of TOM1-like protein 2 OS=Mus musculus GN=Tom1l2
MLIP	Q5FW52	2	2	5683	7739	4229	4274	5199	5502	6143	NA	4195	NA	0.872145713	0.05941095	5424.8	5280	Down	-0.0390320200790105	Muscular LMNA-interacting protein OS=Mus musculus GN=Mlip PE=1 SV=1
CASKIN1	Q6P9K8-4	5	5	22844	27889	23536	47994.4	21572.4	32229.8	35937.2	24865.2	28643.8	26636.6	0.872367209	0.059300667	28767.16	29662.52	Up	0.0442183682203128	Isoform 4 of Caskin-1 OS=Mus musculus GN=Caskin1
HMGA1	P17095	4	4	47913.5	18377.5	25866	10642.5	26210.5	16808	17447.25	29090	23477.25	36173.5	0.872699029	0.059135507	25802	24599.2	Down	-0.0688715002491447	High mobility group protein HMG-I/HMG-Y OS=Mus musculus GN=Hmga1 PE=1 SV=4
KSR2	Q3UVC0	2	2	3299	2315	1374	5666	4588.5	4556.5	3742	NA	1434.5	NA	0.873271868	0.05885053	3448.5	3244.3333	Down	-0.0880469124373564	Kinase suppressor of Ras 2 OS=Mus musculus GN=Ksr2 PE=2 SV=2
RPL23A	P62751	9	9	207336.67	227414.11	192533	241673.33	196356.56	220485	234177.78	197002	275184.89	114797.44	0.873368586	0.058802433	213062.73	208329.42	Down	-0.0324116757664751	60S ribosomal protein L23a OS=Mus musculus GN=Rpl23a PE=1 SV=1
ABLIM1	Q8K4G5-3	10	10	19964.5	25477.3	20355.6	21718.8	25860.3	24175.5	25192.5	20894.2	31565.8	8138.1	0.873718512	0.058628462	22675.3	21993.22	Down	-0.0440627952589182	Isoform 3 of Actin-binding LIM protein 1 OS=Mus musculus GN=Ablim1
UQCR11	Q9CPX8	1	1	25273	24182	16745	25333	15943	23949	16691	27893	19591	NA	0.87387277	0.058551793	21495.2	22031	Up	0.0355204474988473	Cytochrome b-c1 complex subunit 10 OS=Mus musculus GN=Uqcr11 PE=2 SV=1
SS18L1	Q8BW22	2	2	27228	19295.5	19023	15049	22886	19741.5	22913.5	12502	23168	27822	0.874330742	0.058324251	20696.3	21229.4	Up	0.03669072572485	Calcium-responsive transactivator OS=Mus musculus GN=Ss18l1 PE=1 SV=1
DNM1L	Q8K1M6-5	3	3	8851.67	14908	NA	38782	12122.67	18657	23137	NA	17790.33	NA	0.874518719	0.05823089	18666.085	19861.443	Up	0.0895510971469773	Isoform 5 of Dynamin-1-like protein OS=Mus musculus GN=Dnm1l
AK5	Q920P5-2	5	5	18334.6	21639.2	17315.2	15434.8	21648.8	21691.6	22657.2	16866	32548.4	4546.2	0.874608599	0.058186257	18874.52	19661.88	Up	0.0589613181457681	Isoform 2 of Adenylate kinase isoenzyme 5 OS=Mus musculus GN=Ak5
ADGRB3	Q80ZF8	9	9	16179.11	42649.56	17433.78	54570.67	47719.44	49292.56	70847.78	15410.67	51547.89	3535.78	0.87463357	0.058173857	35710.512	38126.936	Up	0.0944617774960499	Brain-specific angiogenesis inhibitor 3 OS=Mus musculus GN=Bai3 PE=1 SV=2
PLCH2	A2AP18-5	2	2	22857	26338	17237	59881	25746	39947	34532	33107	19686	NA	0.876531127	0.057232657	30411.8	31818	Up	0.0652119458286005	Isoform 5 of 1-phosphatidylinositol 4,5-bisphosphate phosphodiesterase eta-2 OS=Mus musculus GN=Plch2
SNW1	Q9CSN1	3	3	8853.33	8097	5758.67	5512	8411	8688.67	9195.33	4518	12333.67	3347	0.876626044	0.057185631	7326.4	7616.534	Up	0.0560301603264617	SNW domain-containing protein 1 OS=Mus musculus GN=Snw1 PE=1 SV=3
LRRC59	Q922Q8	8	8	40966.62	55467.75	34072.12	126372.12	54717.38	85183.5	88002.62	59366.25	58806.75	35658.75	0.876918318	0.057040858	62319.198	65403.574	Up	0.0696928072797211	Leucine-rich repeat-containing protein 59 OS=Mus musculus GN=Lrrc59 PE=1 SV=1
ITPR1	P11881-3	2	2	NA	714.5	NA	6658	2115	3770	3415.5	NA	1307	NA	0.876936159	0.057032022	3162.5	2830.8333	Down	-0.159838667423628	Isoform 3 of Inositol 1,4,5-trisphosphate receptor type 1 OS=Mus musculus GN=Itpr1
ATP6V0A1	Q9Z1G4	11	11	10343.45	11222.73	9891.91	38624.73	16344.82	25283	21429.64	14551.64	12620.55	7461.64	0.877091018	0.056955336	17285.528	16269.294	Down	-0.0874130253733996	V-type proton ATPase 116 kDa subunit a isoform 1 OS=Mus musculus GN=Atp6v0a1 PE=1 SV=3
CNBP	P53996-3	3	3	28523	18856.33	16322.33	21970	24871	17557.33	29577.33	22982.67	39896.33	5523.33	0.877231686	0.05688569	22108.532	23107.398	Up	0.0637515810008241	Isoform 3 of Cellular nucleic acid-binding protein OS=Mus musculus GN=Cnbp
FAM213B	Q9DB60	3	3	74068.33	62025	43754	34368.33	68984	58517	68535.33	26059.33	82667	NA	0.877272999	0.056865237	56639.932	58944.665	Up	0.0575417094128394	Prostamide/prostaglandin F synthase OS=Mus musculus GN=Fam213b PE=1 SV=1
STUM	Q0VBF8	1	1	4260	7453	NA	38158	11469	17320	15752	NA	8892	NA	0.877792641	0.056608065	15335	13988	Down	-0.132638465456666	Uncharacterized membrane protein C1orf95 homolog OS=Mus musculus PE=2 SV=1
1110004F10RIK	Q9R0P4	5	5	25568.4	27851.4	23991.6	19102.6	26961.2	25367.8	26351.2	30251.4	38388.8	7421.8	0.877857376	0.056576038	24695.04	25556.2	Up	0.0494520296256204	Small acidic protein OS=Mus musculus GN=Smap PE=1 SV=1
LINGO1	Q9D1T0	2	2	17627.5	19524.5	13249	30812.5	21931	28711	25370.5	12901.5	12223	NA	0.877873247	0.056568186	20628.9	19801.5	Down	-0.0590571728424941	Leucine-rich repeat and immunoglobulin-like domain-containing nogo receptor-interacting protein 1 OS=Mus musculus GN=Lingo1 PE=1 SV=1
ACBD6	Q9D061	1	1	2603	5736	NA	12529	5622	6364	7338	NA	5079	NA	0.878036538	0.056487411	6622.5	6260.3333	Down	-0.0811364626600156	Acyl-CoA-binding domain-containing protein 6 OS=Mus musculus GN=Acbd6 PE=2 SV=2
ELFN2	Q68FM6	3	3	24058	44010.67	21947.33	71782	43464.67	62692.67	63342.33	14882	44569.33	30059.67	0.878130034	0.056441169	41052.534	43109.2	Up	0.0705245136779794	Protein phosphatase 1 regulatory subunit 29 OS=Mus musculus GN=Elfn2 PE=2 SV=1
EEF2	P58252	28	28	43282.61	58855.93	42769.68	73050.43	55497.21	59100.64	65636.46	47359.93	48315.54	58284.04	0.878318573	0.056347934	54691.172	55739.322	Up	0.0273874754004496	Elongation factor 2 OS=Mus musculus GN=Eef2 PE=1 SV=2
IMMT	Q8CAQ8-4	6	6	44548.83	49270.33	66204.17	56639.5	47238	49967.17	52855.17	32602.83	55179.67	80134	0.8783348	0.05633991	52780.166	54147.768	Up	0.0369059822753458	Isoform 4 of Mitochondrial inner membrane protein OS=Mus musculus GN=Immt
ITSN1	Q9Z0R4	6	6	34534.2	27298.8	47138	55267	41820.2	53986.2	51511.4	18947.2	66176.8	23708	0.87846232	0.056276862	41211.64	42865.92	Up	0.0567792310365868	Intersectin-1 OS=Mus musculus GN=Itsn1 PE=1 SV=2
ITSN1	Q9Z0R4-2	5	5	34534.2	27298.8	47138	55267	41820.2	53986.2	51511.4	18947.2	66176.8	23708	0.87846232	0.056276862	41211.64	42865.92	Up	0.0567792310365868	Isoform 2 of Intersectin-1 OS=Mus musculus GN=Itsn1
HMGB1	P63158	7	7	88378.14	59370.57	95036	70269.57	77944.43	71856.71	70228.57	87854.71	70707.57	96895.43	0.879591228	0.055719111	78199.742	79508.598	Up	0.0239470330519062	High mobility group protein B1 OS=Mus musculus GN=Hmgb1 PE=1 SV=2
GNG7	Q61016	3	3	116096	142231.33	130301	96914.33	114077	105992.33	122400	118154.67	108947	136725.33	0.879595329	0.055717086	119923.93	118443.87	Down	-0.0179161055984027	Guanine nucleotide-binding protein G(I)/G(S)/G(O) subunit gamma-7 OS=Mus musculus GN=Gng7 PE=2 SV=2
IARS2	Q8BIJ6	1	1	NA	2876	NA	10316	NA	7443	7172	NA	NA	NA	0.879705395	0.055662745	6596	7307.5	Up	0.147786525255948	Isoleucine--tRNA ligase, mitochondrial OS=Mus musculus GN=Iars2 PE=1 SV=1
AP2A1	P17426	9	9	58664.33	72688.11	74561.89	110520.11	89381.56	99672.78	102374.89	91218.33	72507	50306.67	0.879723714	0.055653701	81163.2	83215.934	Up	0.0360340518493912	AP-2 complex subunit alpha-1 OS=Mus musculus GN=Ap2a1 PE=1 SV=1
ST13	Q99L47	22	22	66055.91	64034.14	73324.09	40890.64	61530.32	50527.14	57325.55	79026.91	74543.23	50731.55	0.880173704	0.055431611	61167.02	62430.876	Up	0.0295057181039393	Hsc70-interacting protein OS=Mus musculus GN=St13 PE=1 SV=1
ATP6V0A1	Q9Z1G4-3	14	14	8452.5	10374.07	7772.21	36163.29	14307.21	23035.64	19961.43	12873.71	10506.71	5862.71	0.88026027	0.055388899	15413.856	14448.04	Down	-0.0933540260285438	Isoform A1-III of V-type proton ATPase 116 kDa subunit a isoform 1 OS=Mus musculus GN=Atp6v0a1
TST	P52196	2	2	26735	24509	1515	32948.5	27800	35444.5	27892	4904.5	32762	5806.5	0.880371665	0.055333944	22701.5	21361.9	Down	-0.0877476557046868	Thiosulfate sulfurtransferase OS=Mus musculus GN=Tst PE=1 SV=3
DST	Q91ZU6-5	1	1	NA	NA	NA	7109	1132	3713	3396	NA	NA	NA	0.880871028	0.055087674	4120.5	3554.5	Down	-0.213172776255882	Isoform 5 of Dystonin OS=Mus musculus GN=Dst
ROMO1	P60603	1	1	74473	57430	76089	48454	72461	69445	64825	70197	62302	NA	0.880984342	0.05503181	65781.4	66692.25	Up	0.0198394093681922	Reactive oxygen species modulator 1 OS=Mus musculus GN=Romo1 PE=2 SV=1
SRCIN1	Q9QWI6-3	79	79	61206.75	67227.4	52493.01	69893.06	72188.88	70491.52	77854.36	48606.97	89713.17	43726.6	0.881164774	0.054942873	64601.82	66078.524	Up	0.0326066516161108	Isoform 3 of SRC kinase signaling inhibitor 1 OS=Mus musculus GN=Srcin1
CCM2	Q8K2Y9	1	1	1155	842	NA	2483	356	1053	1111	NA	1701	NA	0.881482031	0.054786537	1209	1288.3333	Up	0.09169166884193	Malcavernin OS=Mus musculus GN=Ccm2 PE=1 SV=1
GPD1	P13707	3	3	36489	43020	43582.67	68493	43848.33	62606.67	55847.33	29669	53776	26442.33	0.881549492	0.054753301	47086.6	45668.266	Down	-0.0441245407842759	Glycerol-3-phosphate dehydrogenase [NAD(+)], cytoplasmic OS=Mus musculus GN=Gpd1 PE=1 SV=3
SNRPD2	P62317	1	1	140000	76272	113417	38805	122364	58970	88315	92313	129804	139396	0.881686556	0.054685781	98171.6	101759.6	Up	0.0517872710781852	Small nuclear ribonucleoprotein Sm D2 OS=Mus musculus GN=Snrpd2 PE=2 SV=1
PROSC	Q9Z2Y8	6	6	87900.67	89463	76965.83	83761.5	89071.5	89079.5	83052.17	59759.83	99594.17	101856.17	0.882217434	0.054424364	85432.5	86668.368	Up	0.0207205379560593	Proline synthase co-transcribed bacterial homolog protein OS=Mus musculus GN=Prosc PE=1 SV=1
ARFGAP1	Q9EPJ9	23	23	44548	54607.8	53288.55	48153.2	52841.9	55186.2	60853.2	58884.3	53102.2	29986.2	0.882633623	0.054219533	50687.89	51602.42	Up	0.0257976156628477	ADP-ribosylation factor GTPase-activating protein 1 OS=Mus musculus GN=Arfgap1 PE=1 SV=2
SRCIN1	Q9QWI6-2	80	80	59601.94	65495.67	50591.19	68554.5	70222.83	68958.09	75951.55	48592.06	86858.4	41238.59	0.882675034	0.054199157	62893.226	64319.738	Up	0.0323568918045523	Isoform 2 of SRC kinase signaling inhibitor 1 OS=Mus musculus GN=Srcin1
MBP	P04370-2	23	23	961924.96	730170.48	1226888.7	217244.87	685974.65	437022.65	308968.91	988732.57	494905.52	1392794.8	0.883172657	0.053954385	764440.72	724484.9	Down	-0.0774490202741627	Isoform 2 of Myelin basic protein OS=Mus musculus GN=Mbp
HIST2H2BE	Q64524	30	30	452377.47	205328.83	337134.23	63582.2	257840.53	127465.07	150247.63	408901.67	170503.7	538263.7	0.883976091	0.053559481	263252.65	279076.35	Up	0.0842118257580151	Histone H2B type 2-E OS=Mus musculus GN=Hist2h2be PE=1 SV=3
CIT	P49025-5	15	15	21068.87	27240.2	19052.47	29952.4	33445.73	34742	32908.2	26555.07	35676.93	5610.4	0.884169116	0.053464659	26151.934	27098.52	Up	0.0512964189053152	Isoform 5 of Citron Rho-interacting kinase OS=Mus musculus GN=Cit
RPL27A	P14115	6	6	137382.17	142390.33	113184.17	119446.17	105139.67	93629.5	138681.67	104427.17	129540.17	161929.5	0.884532082	0.053286411	123508.5	125641.6	Up	0.0247038868251177	60S ribosomal protein L27a OS=Mus musculus GN=Rpl27a PE=2 SV=5
MIA3	Q8BI84	7	7	1928.29	4280.71	1564.71	12147	6117.14	8487.43	8325	622.43	5001.29	NA	0.884543359	0.053280874	5207.57	5609.0375	Up	0.107142901330407	Melanoma inhibitory activity protein 3 OS=Mus musculus GN=Mia3 PE=1 SV=2
YARS	Q91WQ3	4	4	15631.75	23386	21106.5	41387.25	28225.25	35659.5	39341.5	940.5	33941	NA	0.884763543	0.053172781	25947.35	27470.625	Up	0.0823025305514708	Tyrosine--tRNA ligase, cytoplasmic OS=Mus musculus GN=Yars PE=2 SV=3
IDH1	O88844	22	22	64455.77	79955.55	82235.14	78027.55	66646.41	79664.32	78299.68	74365.95	76246.64	58857.68	0.885646692	0.052739495	74264.084	73486.854	Down	-0.0151784648525368	Isocitrate dehydrogenase [NADP] cytoplasmic OS=Mus musculus GN=Idh1 PE=1 SV=2
WWC1	Q5SXA9	1	1	24896	25408	NA	31364	23735	33425	27026	NA	16051	NA	0.885937147	0.052597088	26350.75	25500.667	Down	-0.0473090605958068	Protein KIBRA OS=Mus musculus GN=Wwc1 PE=1 SV=1
NRXN1	Q9CS84-3	3	3	10802.33	18731.33	20435.67	55100.67	26573.33	37054.67	25287.33	NA	20694	NA	0.886279227	0.05242943	26328.666	27678.667	Up	0.072140021414234	Isoform 3a of Neurexin-1 OS=Mus musculus GN=Nrxn1
CAMK2N2	Q78WH7	1	1	16331	17513	9674	72522	25729	34649	27142	8487	35428	NA	0.886511261	0.052315743	28353.8	26426.5	Down	-0.101556736279754	Calcium/calmodulin-dependent protein kinase II inhibitor 2 OS=Mus musculus GN=Camk2n2 PE=3 SV=1
NDUFA5	Q9CPP6	17	17	132545.47	131340.76	108797.94	95548.59	115728.18	101246.88	108101.12	112379.71	117441.24	153200.35	0.887156448	0.051999787	116792.19	118473.86	Up	0.0206250004752945	NADH dehydrogenase [ubiquinone] 1 alpha subcomplex subunit 5 OS=Mus musculus GN=Ndufa5 PE=1 SV=3
SRSF3	P84104	7	7	52970.29	46405.57	46815.57	51781.43	53175.71	48290.29	46966	39715.29	62861.57	NA	0.88736856	0.051895962	50229.714	49458.288	Down	-0.0223287734784521	Serine/arginine-rich splicing factor 3 OS=Mus musculus GN=Srsf3 PE=1 SV=1
PTGES3	Q9R0Q7	5	5	42733.5	44213.75	71893.5	28478.5	46886	40752.25	45353.5	55427	53768.25	32906	0.887773509	0.051697818	46841.05	45641.4	Down	-0.0374303705961843	Prostaglandin E synthase 3 OS=Mus musculus GN=Ptges3 PE=1 SV=1
GSK3B	Q9WV60	12	12	23934.5	28684.5	29810	27909.4	30372	32507	38781.4	23196.2	33584.4	8593.1	0.888231635	0.051473763	28142.08	27332.42	Down	-0.0421157619957416	Glycogen synthase kinase-3 beta OS=Mus musculus GN=Gsk3b PE=1 SV=2
MYO5A	Q99104	22	22	10577.95	31214.9	8567.14	90199.76	38050.33	54822.76	64999.24	12868.76	27608.19	4683.67	0.888741798	0.051224394	35722.016	32996.524	Down	-0.114499449793807	Unconventional myosin-Va OS=Mus musculus GN=Myo5a PE=1 SV=2

KCNA2	P63141	1	1	2053	6057	NA	8939	5731	4848	7793	NA	3617	NA	0.88908343	0.051057483	5695	5419.3333	Down	-0.0715804545331822	Potassium voltage-gated channel subfamily A member 2 OS=Mus musculus GN=Kcna2 PE=1 SV=1
CNKSR2	Q80YA9	13	13	16100.08	18682	10737.92	20481.31	23471.38	23023.77	24871.54	16530	18573.31	8997.46	0.88981133	0.050702069	17894.538	18399.216	Up	0.0401249967888765	Connector enhancer of kinase suppressor of ras 2 OS=Mus musculus GN=Cnksr2 PE=1 SV=1
CADM4	Q8R464	8	8	31263.12	36715.38	38158	54105.62	39234.62	52330	45975.75	44515.12	42946.5	18652.62	0.890515424	0.050358554	39895.348	40883.998	Up	0.0353157519999651	Cell adhesion molecule 4 OS=Mus musculus GN=Cadm4 PE=1 SV=1
RABEP2	Q91WG2-2	1	1	5569	2167	NA	7632	3780	7313	2309	NA	3917	NA	0.891181999	0.050033595	4787	4513	Down	-0.0850350275072791	Isoform 3 of Rab GTPase-binding effector protein 2 OS=Mus musculus GN=Rabep2
PCDH8	Q7TSK3-3	2	2	7352.5	7557.5	2629	10535	7592.5	9079.5	8872	4108	8251	4152	0.891192672	0.050028393	7133.3	6892.5	Down	-0.0495422869757995	Isoform 3 of Protocadherin-8 OS=Mus musculus GN=Pcdh8
SLC4A4	O88343	7	7	22661.57	45435.14	8483.71	90549.71	50980.43	56868	52633.29	22829.86	32938.29	NA	0.891266684	0.049992327	43622.112	41317.36	Down	-0.0783115457267889	Electrogenic sodium bicarbonate cotransporter 1 OS=Mus musculus GN=Slc4a4 PE=1 SV=2
SH3BGRL3	Q91VW3	7	7	214913.29	197075.29	72201.29	143769	206097.29	220424.29	251637.29	49811.14	257198	13170.71	0.891829459	0.049718186	166811.23	158448.29	Down	-0.0742043803158534	SH3 domain-binding glutamic acid-rich-like protein 3 OS=Mus musculus GN=Sh3bgrl3 PE=1 SV=1
DAG1	Q62165	2	2	191432.5	160847	198030.5	100094	144632	126047.5	166461.5	144300	226893	112683.5	0.89255014	0.049367378	159007.2	155277.1	Down	-0.0342470143910241	Dystroglycan OS=Mus musculus GN=Dag1 PE=1 SV=4
EHD1	Q9WVK4	4	4	27659.75	35488.25	11862.25	87253	36944.5	55630.5	56796.75	12038.75	25457.5	NA	0.892702932	0.049293039	39841.55	37480.875	Down	-0.0881191418114264	EH domain-containing protein 1 OS=Mus musculus GN=Ehd1 PE=1 SV=1
NUFIP2	Q5F2E7-2	2	2	17662	18978.5	9523	27142.5	31563.5	23909	25959	7328	30261	NA	0.892707388	0.049290871	20973.9	21864.25	Up	0.0599787122483625	Isoform 2 of Nuclear fragile X mental retardation-interacting protein 2 OS=Mus musculus GN=Nufip2
AHSA1	Q8BK64	3	3	35331.67	56125.67	25401.33	53423.33	50868.33	56674.67	57202	21592.67	52756.33	26078.67	0.892902616	0.049195905	44230.066	42860.868	Down	-0.0453663278699635	Activator of 90 kDa heat shock protein ATPase homolog 1 OS=Mus musculus GN=Ahsa1 PE=2 SV=2
SYN1	O88935	107	107	110291.93	97694.32	92780.69	109696.62	91056.55	110870.42	100134.77	85414.44	102129.95	98942.68	0.892923306	0.049185841	100304.02	99498.452	Down	-0.0116334709804674	Synapsin-1 OS=Mus musculus GN=Syn1 PE=1 SV=2
SST	P60041	6	6	38159.2	39945.2	37824.2	25776.2	29333.8	31799.2	36081.8	53115.6	48565.6	7452.2	0.893458478	0.048925626	34207.72	35402.88	Up	0.0495447779873701	Somatostatin OS=Mus musculus GN=Sst PE=2 SV=1
EEF1A1	P10126	52	52	109314.88	109387	132474.27	122905.48	101108.04	106160.31	111656.29	130926.62	106811.19	124805.31	0.893796359	0.048761419	115037.93	116071.94	Up	0.0129096260713288	Elongation factor 1-alpha 1 OS=Mus musculus GN=Eef1a1 PE=1 SV=3
HN1	P97825	4	4	27703	41517.5	16256.5	30331	32864.5	35079	34810.25	18010.25	44147.75	11647.75	0.894881914	0.048234269	29734.5	28739	Down	-0.0491279535385318	Hematological and neurological expressed 1 protein OS=Mus musculus GN=Hn1 PE=1 SV=3
FLOT2	Q60634-3	6	6	9265.17	16132.33	8016.67	50425.17	23265.67	31272	40106.67	5181.83	15172.17	NA	0.894905849	0.048222653	21421.002	22933.168	Up	0.0984096659648691	Isoform 3 of Flotillin-2 OS=Mus musculus GN=Flot2
MRPL19	Q9D338	4	4	30845	34029.5	30974.75	32557.5	28173.25	40091.75	38373.5	38220	39647.75	5071.25	0.895019043	0.048167724	31316	32280.85	Up	0.0437786186752704	39S ribosomal protein L19, mitochondrial OS=Mus musculus GN=Mrpl19 PE=2 SV=1
AIMP2	Q8R010	2	2	NA	NA	NA	2951.5	1541	2457.5	1899	NA	2028.5	NA	0.895215277	0.048072515	2246.25	2128.3333	Down	-0.0777943845283742	Aminoacyl tRNA synthase complex-interacting multifunctional protein 2 OS=Mus musculus GN=Aimp2 PE=1 SV=2
IMMT	Q8CAQ8-5	63	63	56463	70529.38	44041.4	131604.02	76428.43	98405.54	91775.97	62755.95	72001.68	41749.81	0.895462499	0.047952597	75813.246	73337.79	Down	-0.0478931446133173	Isoform 5 of Mitochondrial inner membrane protein OS=Mus musculus GN=Immt
IMMT	Q8CAQ8	64	64	55657.3	69449.31	43353.25	129612.77	75278.11	96919.62	90401.2	61775.39	70999.45	41097.47	0.895753343	0.047811562	74670.148	72238.626	Down	-0.0477611379432879	Mitochondrial inner membrane protein OS=Mus musculus GN=Immt PE=1 SV=1
EMC3	Q99KI3	1	1	16735	61749	13255	270345	64957	129977	136050	55851	48610	NA	0.896274751	0.047558838	85408.2	92622	Up	0.116980320747858	ER membrane protein complex subunit 3 OS=Mus musculus GN=Emc3 PE=2 SV=3
FKBP8	O35465	14	14	22886.85	30582.08	16508.31	39114.77	26576.77	34913.31	33511.54	14223.08	33507.15	15486.69	0.897216281	0.047102854	27133.756	26328.354	Down	-0.0434714374492328	Peptidyl-prolyl cis-trans isomerase FKBP8 OS=Mus musculus GN=Fkbp8 PE=1 SV=2
CAMK2A	P11798-2	23	23	21179.18	25109.95	23309.36	42448.36	25904.73	30261.05	30593.86	27481.32	23341.95	23491.18	0.897393736	0.047016966	27590.316	27033.872	Down	-0.0293938197949534	Isoform Alpha KAP of Calcium/calmodulin-dependent protein kinase type II subunit alpha OS=Mus musculus GN=Camk2a
SYNE1	Q6ZWR6-3	1	1	1809	6709	NA	9164	6685	6534	7062	NA	3937	NA	0.897411463	0.047008387	6091.75	5844.3333	Down	-0.0598182710796689	Isoform 3 of Nesprin-1 OS=Mus musculus GN=Syne1
RPS24	P62849-3	8	8	94835.12	114290.12	108868.75	89572.75	88570.38	91099.5	103626.12	68624.62	131599	109001.88	0.89745347	0.046988059	99227.424	100790.22	Up	0.0225449075568212	Isoform 3 of 40S ribosomal protein S24 OS=Mus musculus GN=Rps24
BDH1	Q80XN0	13	13	36075.69	39591.08	34155.08	59028.38	34459.85	46650.77	45210.46	39679.92	41279.92	33979.38	0.897764965	0.046837347	40662.016	41360.09	Up	0.0245575800074644	D-beta-hydroxybutyrate dehydrogenase, mitochondrial OS=Mus musculus GN=Bdh1 PE=1 SV=2
NRXN3	Q6P9K9	1	1	51008	81549	116785	88878	60363	77999	73394	72515	102167	NA	0.89782412	0.046808731	79716.6	81518.75	Up	0.0322517505672643	Neurexin-3 OS=Mus musculus GN=Nrxn3 PE=1 SV=2
SNAP29	Q9ERB0	12	12	28251.5	30558	20177.67	22897.33	27947	27628.83	32904.75	27207.92	43394.67	3310	0.8984294	0.046516045	25966.3	26889.234	Up	0.0503882017946882	Synaptosomal-associated protein 29 OS=Mus musculus GN=Snap29 PE=2 SV=1
LZTS3	A2AHG0-3	9	9	21372.83	29522	32713.17	11580.33	25803.33	20928.83	26286.67	31818	32806.5	12597.83	0.898682047	0.046393934	24198.332	24887.566	Up	0.0405175364494534	Isoform 3 of Leucine zipper putative tumor suppressor 3 OS=Mus musculus GN=Lzts3
GRIA1	P23818	7	7	21047.14	31980.86	22362.14	57387.14	27626.71	41468.14	40987.29	29949.29	30745.14	11631.29	0.898732	0.046369794	32080.798	30956.23	Down	-0.0514802450623947	Glutamate receptor 1 OS=Mus musculus GN=Gria1 PE=1 SV=1
PGAM5	Q8BX10	5	5	19602.4	32975.4	18438.8	51682.4	25242.4	40395.2	36629.2	19892.2	25509	NA	0.899049391	0.046216449	29588.28	30606.4	Up	0.0488075280233371	Serine/threonine-protein phosphatase PGAM5, mitochondrial OS=Mus musculus GN=Pgam5 PE=2 SV=1
DYNLL1	P63168	1	1	1145	3009	NA	8337	3015	3998	3321	NA	NA	NA	0.899236034	0.046126298	3876.5	3659.5	Down	-0.0831081193624649	Dynein light chain 1, cytoplasmic OS=Mus musculus GN=Dynll1 PE=1 SV=1
GPI1	P06745	17	17	37236.12	50066.53	55674.35	96369.18	55466.59	70889.41	70454.53	56332.59	46412.88	43234.59	0.900407724	0.045560788	58962.554	57464.8	Down	-0.0371205116786419	Glucose-6-phosphate isomerase OS=Mus musculus GN=Gpi PE=1 SV=4
DAAM1	Q8BPM0-3	1	1	NA	6681	936	37981	10219	20183	14651	NA	3220	NA	0.90061277	0.0454619	13954.25	12684.667	Down	-0.137618977746029	Isoform 3 of Disheveled-associated activator of morphogenesis 1 OS=Mus musculus GN=Daam1
KIF2A	P28740	16	16	29448.25	37084.56	37351.94	38720.75	34628.19	37944.19	41104.62	24626.12	33795.38	42073.19	0.901212731	0.045172682	35446.738	35908.7	Up	0.0186805551715806	Kinesin-like protein KIF2A OS=Mus musculus GN=Kif2a PE=1 SV=2
CDC42SE1	Q8BHL7	1	1	15516	15758	22862	9105	16212	14067	14921	NA	19774	NA	0.901455765	0.045055579	15890.6	16254	Up	0.0326212001320803	CDC42 small effector protein 1 OS=Mus musculus GN=Cdc42se1 PE=3 SV=1
ZC3H15	Q3TIV5	1	1	2289	1384	NA	2876	1459	2432	2064	NA	1321	NA	0.901456682	0.045055138	2002	1939	Down	-0.0461291707292011	Zinc finger CCCH domain-containing protein 15 OS=Mus musculus GN=Zc3h15 PE=1 SV=2
GNB2	P62880	26	26	107261.62	121273.58	85086.23	267525.08	111776.35	174384.42	133357.23	154568.04	129481.96	77538.08	0.901661137	0.044956649	138584.57	133865.95	Down	-0.0499776557872674	Guanine nucleotide-binding protein G(I)/G(S)/G(T) subunit beta-2 OS=Mus musculus GN=Gnb2 PE=1 SV=3
NDE1	Q9CZA6-4	2	2	106399.5	90642.5	69527	70588.5	113426.5	85281.5	106599	21360	133749.5	NA	0.901792247	0.044893503	90116.8	86747.5	Down	-0.0549739042776612	Isoform 4 of Nuclear distribution protein nudE homolog 1 OS=Mus musculus GN=Nde1
EIF1B	Q9CXU9	4	4	33672	30890.33	16406	43362.67	32935.33	36988.33	30102.33	19922.33	35828.33	NA	0.90217467	0.044709371	31453.266	30710.33	Down	-0.0344858144437973	Eukaryotic translation initiation factor 1b OS=Mus musculus GN=Eif1b PE=2 SV=2
LRPAP1	P55302	9	9	27264.11	34187.22	18174.11	62666.33	32596.11	50948	37973.67	24276.67	47613.22	7109.44	0.902344791	0.044627485	34977.576	33584.2	Down	-0.0586476484520602	Alpha-2-macroglobulin receptor-associated protein OS=Mus musculus GN=Lrpap1 PE=1 SV=1
SLC30A9	Q5IRJ6	1	1	4206	11353	NA	24945	10474	15031	15753	NA	9331	NA	0.902466147	0.04456908	12744.5	13371.667	Up	0.0693045227583425	Zinc transporter 9 OS=Mus musculus GN=Slc30a9 PE=1 SV=2
DCTPP1	Q9QY93	1	1	1006	NA	NA	1982	1863	1590	1014	NA	2451	NA	0.902519471	0.04454342	1617	1685	Up	0.0594289125955593	dCTP pyrophosphatase 1 OS=Mus musculus GN=Dctpp1 PE=1 SV=1
FLOT1	O08917	20	20	18445.05	30570.45	15601.9	54806.45	31571.9	42386.05	43994.2	14452.2	33450.95	10513.85	0.902838678	0.044389844	30199.15	28959.45	Down	-0.0604737404125869	Flotillin-1 OS=Mus musculus GN=Flot1 PE=1 SV=1
LMAN1	Q9D0F3	1	1	1708	6475	NA	10002	5556	7832	7242	NA	3564	NA	0.9030348	0.044295513	5935.25	6212.6667	Up	0.0659038510187856	Protein ERGIC-53 OS=Mus musculus GN=Lman1 PE=2 SV=1
PSMA5	Q9Z2U1	7	7	33721.71	33769.71	37241.86	29561.71	31649.86	34058	38112.14	41122.29	39545.57	16155.43	0.903039559	0.044293225	33188.97	33798.686	Up	0.0262633026544429	Proteasome subunit alpha type-5 OS=Mus musculus GN=Psma5 PE=1 SV=1
CLCN7	O70496	1	1	1416	1283	NA	7764	579	4203	3253	NA	1540	NA	0.903571969	0.044037251	2760.5	2998.6667	Up	0.119391559264722	H(+)/Cl(-) exchange transporter 7 OS=Mus musculus GN=Clcn7 PE=1 SV=1
SV2A	Q9JIS5	10	10	20127.71	49310.71	13803.43	187926	50012.14	104836	78230.29	17836.57	37244	NA	0.903809953	0.04392288	64235.998	59536.715	Down	-0.109602391991388	Synaptic vesicle glycoprotein 2A OS=Mus musculus GN=Sv2a PE=1 SV=1
DCTN6	Q9WUB4	3	3	38015.33	37402	34180	29690.33	36177	35047.67	33645	45288.33	49384.33	15894	0.904833927	0.043431124	35092.932	35851.866	Up	0.0308677202000502	Dynactin subunit 6 OS=Mus musculus GN=Dctn6 PE=1 SV=1
STXBP5L	Q5DQR4	1	1	678	2830	NA	7004	4025	4555	3841	NA	1926	NA	0.904954514	0.043373249	3634.25	3440.6667	Down	-0.0789695360538766	Syntaxin-binding protein 5-like OS=Mus musculus GN=Stxbp5l PE=2 SV=1
ANK2	Q8C8R3-6	16	16	12368.69	28703.19	12143.81	68868.81	41767.69	54320.88	59769.94	6691.56	31271.06	2011.06	0.904984451	0.043358882	32770.438	30812.9	Down	-0.0888604855782648	Isoform 6 of Ankyrin-2 OS=Mus musculus GN=Ank2
RIMS2	Q9EQZ7-2	1	1	7654	5689	NA	15242	38042	18659	17661	NA	10645	NA	0.904996812	0.043352951	16656.75	15655	Down	-0.0894834266638945	Isoform 2 of Regulating synaptic membrane exocytosis protein 2 OS=Mus musculus GN=Rims2
AP2M1	P84091	19	19	112109.11	162452.63	118001.95	319987.58	185430.16	247310.89	255085	125908.32	145258.11	93630.74	0.905014778	0.043344329	179596.29	173438.61	Down	-0.0503323996601416	AP-2 complex subunit mu OS=Mus musculus GN=Ap2m1 PE=1 SV=1
RAP2A	Q80ZJ1	3	3	9748.67	17943.33	8512.33	23517	15919.67	20711	20604.67	5366.67	16072.33	NA	0.905735304	0.042998704	15128.2	15688.668	Up	0.0524824823166114	Ras-related protein Rap-2a OS=Mus musculus GN=Rap2a PE=1 SV=2
SPECC1	Q5SXY1-3	7	7	19106.33	21711.5	15321.5	29910.33	25650.17	28197.83	28890.5	7262.83	27932.33	NA	0.906113842	0.042817235	22339.966	23070.873	Up	0.0464455751195597	Isoform 4 of Cytospin-B OS=Mus musculus GN=Specc1
4933434E20RIK	Q8R092-2	1	1	NA	2458	NA	9234	3949	9151	5772	NA	1827	NA	0.906341401	0.042708181	5213.6667	5583.3333	Up	0.0988283419455482	Isoform 3 of Uncharacterized protein C1orf43 homolog OS=Mus musculus
RGS7	O54829	3	3	32502.5	30724	23509	22261	25461	33532.5	32116	9515	29405.5	NA	0.906344869	0.042706519	26891.5	26142.25	Down	-0.0407669148251093	Regulator of G-protein signaling 7 OS=Mus musculus GN=Rgs7 PE=1 SV=2
PSMB5	O55234	1	1	11556	14602	12539	17135	9512	17014	9767	11947	12572	NA	0.906887218	0.042446719	13068.8	12825	Down	-0.0271678506086603	Proteasome subunit beta type-5 OS=Mus musculus GN=Psmb5 PE=1 SV=3
RAP1GAP	A2ALS5-3	5	5	17618.2	20387.2	13342.4	18333.8	24975.8	18724.8	18636.2	19128	22409	14377	0.907164433	0.042313985	18931.48	18655	Down	-0.0212248403849379	Isoform 3 of Rap1 GTPase-activating protein 1 OS=Mus musculus GN=Rap1gap
ATP4A	Q64436	7	7	46706.83	144631.33	7731	408018.67	132650.17	241182.17	226483	80395.5	83491.5	NA	0.907867595	0.041977485	147947.6	157888.04	Up	0.093815620824352	Potassium-transporting ATPase alpha chain 1 OS=Mus musculus GN=Atp4a PE=1 SV=3
RPS5	P97461	3	3	62434.33	82214	58049.33	107486.33	73555	78477.33	78586.67	85637.67	73395.67	61839.67	0.908247983	0.041795558	76747.798	75587.402	Down	-0.0219795547633151	40S ribosomal protein S5 OS=Mus musculus GN=Rps5 PE=1 SV=3
UNC13A	Q4KUS2	3	3	6847	29343.33	15393	87252	27691.33	43831	41627.33	25472	23778.33	22833.33	0.90847659	0.041686259	33305.332	31508.398	Down	-0.0800167588793711	Protein unc-13 homolog A OS=Mus musculus GN=Unc13a PE=1 SV=3
APOA1	Q00623	3	3	61029.67	69963.67	31750.67	32756.33	40844.33	63250.33	61337.67	27887	59467	30938.67	0.908580191	0.041636736	47268.934	48576.134	Up	0.0393553459474491	Apolipoprotein A-I OS=Mus musculus GN=Apoa1 PE=1 SV=2
DST	Q91ZU6-3	5	5	1016.2	1545	1426.8	9027.2	1994.8	5268.2	3724.6	799.6	1340.4	NA	0.908801497	0.041530966	3002	2783.2	Down	-0.109179392888161	Isoform 3 of Dystonin OS=Mus musculus GN=Dst
IL6ST	Q00560	1	1	NA	NA	NA	2751	532	2123	1305	NA	1011	NA	0.908828335	0.041518141	1641.5	1479.6667	Down	-0.149742541969043	Interleukin-6 receptor subunit beta OS=Mus musculus GN=Il6st PE=1 SV=2
AP1S1	P61967	1	1	NA	2174	1759	10222	2441	5789	3926	NA	1890	NA	0.908954684	0.041457768	4149	3868.3333	Down	-0.101051540360962	AP-1 complex subunit sigma-1A OS=Mus musculus GN=Ap1s1 PE=1 SV=1
G3BP2	P97379	8	8	85579.38	99324.62	58342.88	122145.12	92686.75	101236.38	104092.88	80877.75	107081.12	56493.88	0.909036632	0.041418615	91615.75	89956.402	Down	-0.0263696800707244	Ras GTPase-activating protein-binding protein 2 OS=Mus musculus GN=G3bp2 PE=1 SV=2
CAR2	P00920	2	2	72637	82898.5	78486	68762	86080	94035	67169.5	104321	94912	36170.5	0.909159042	0.041360138	77772.7	79321.6	Up	0.0284499534830642	Carbonic anhydrase 2 OS=Mus musculus GN=Ca2 PE=1 SV=4
IQSEC2	Q5DU25	16	16	12720.19	18863.56	10768.19	33902.38	27168.44	28542.06	27941.5	16678.56	23111.62	3394.56	0.90923366	0.041324495	20684.552	19933.66	Down	-0.0533470847107394	IQ motif and SEC7 domain-containing protein 2 OS=Mus musculus GN=Iqsec2 PE=1 SV=3
TPD52L2	Q9CYZ2	6	6	47980.83	50584	45781.17	53991.17	49399	59530.17	52796.17	61283.17	74064.33	7074.33	0.909272217	0.041306079	49547.234	50949.634	Up	0.0402672622730465	Tumor protein D54 OS=Mus musculus GN=Tpd52l2 PE=1 SV=1
FAM49A	Q8BHZ0	2	2	10243	27916.5	22494.5	68158.5	29863.5	42878.5	56880.5	12469	21333	NA	0.909479304	0.041207179	31735.2	33390.25	Up	0.0733429604799346	Protein FAM49A OS=Mus musculus GN=Fam49a PE=2 SV=1
FABP5	Q05816	28	28	65032.32	64140.93	61144.25	81395.21	64711.29	68289.04	65440.04	74202.54	79472.18	44945.21	0.909613889	0.041142917	67284.8	66469.802	Down	-0.0175815726277732	Fatty acid-binding protein, epidermal OS=Mus musculus GN=Fabp5 PE=1 SV=3
ETL4	A2AQ25-7	1	1	4035	NA	8845	4931	8733	7401	5488	NA	NA	NA	0.909735248	0.041084978	6636	6444.5	Down	-0.0422454556359094	Isoform 7 of Sickle tail protein OS=Mus musculus GN=Skt
TNIK	P83510	22	22	15978.06	19545.5	23492.25	24407.25	20862.62	22035.62	23054.44	17188.81	21156.81	19784.94	0.909769184	0.041068778	20857.136	20644.124	Down	-0.0148098662411658	Traf2 and NCK-interacting protein kinase OS=Mus musculus GN=Tnik PE=1 SV=2
GRIPAP1	Q8VD04	7	7	32391.57	32827.57	21514.14	39900.29	40387.86	40975.86	34642.57	22913.43	48125.71	16403.71	0.909846946	0.041031658	33404.286	32612.256	Down	-0.034618977231876	GRIP1-associated protein 1 OS=Mus musculus GN=Gripap1 PE=1 SV=1
PTRH1	Q8BW00	1	1	5781	5111	3490	5109	6474	7156	5908	NA	3004	NA	0.910284688	0.040565206	5193	5356	Up	0.0445877352365158	Probable peptidyl-tRNA hydrolase OS=Mus musculus GN=Ptrh1 PE=2 SV=1
AP3D1	O54774	5	5	18554	19974.6	15081.6	81422.6	25622.2	48468.2	44384.2	16831.8	25618.6	NA	0.910854134	0.040551166	32131	33825.7	Up	0.0741539081925193	AP-3 complex subunit delta-1 OS=Mus musculus GN=Ap3d1 PE=1 SV=1
INPP4A	Q9EPW0	4	4	3185	5669.5	1854	14498.5	7539	9451.5	9387	2811	5816.5	NA	0.910901973	0.040528357	6549.2	6866.5	Up	0.0682562254192242	Type I inositol 3,4-bisphosphate 4-phosphatase OS=Mus musculus GN=Inpp4a PE=2 SV=1
ATP6V1G1	Q9CR51	8	8	46375	52697.62	32933.75	95621.25	57128.38	71131.75	50336.75	33135	66901.88	NA	0.911129936	0.040419684	56951.2	55376.345	Down	-0.0404564060322339	V-type proton ATPase subunit G 1 OS=Mus musculus GN=Atp6v1g1 PE=2 SV=3
GRPEL1	Q99LP6	9	9	77575.22	75044.56	92849.89	51727.11	73899.33	65781	62666.56	59405.67	91937	85960.56	0.911453018	0.040265712	74219.222	73150.158	Down	-0.02093189761935	GrpE protein homolog 1, mitochondrial OS=Mus musculus GN=Grpel1 PE=1 SV=1
NKIRAS1	Q8CEC5	1	1	18145	22410	20329	22642	19479	22214	26258	15774	19287	NA	0.911596609	0.040197299	20601	20883.25	Up	0.0196318824024924	NF-kappa-B inhibitor-interacting Ras-like protein 1 OS=Mus musculus GN=Nkiras1 PE=1 SV=1
ALDH7A1	Q9DBF1	5	5	11203	11758.67	9885.67	25248	14700	21099	16776	6746	15505	NA	0.911687821	0.040153847	14559.068	15031.5	Up	0.0460709793666344	Alpha-aminoadipic semialdehyde dehydrogenase OS=Mus musculus GN=Aldh7a1 PE=1 SV=4
CYC1	Q9D0M3	3	3	55393	80734.67	32640.33	113515.67	69596.33	83263	75198.67	44505.33	71242.33	NA	0.911938663	0.040034371	70376	68552.333	Down	-0.0378777624710451	Cytochrome c1, heme protein, mitochondrial OS=Mus musculus GN=Cyc1 PE=1 SV=1
EPHA3	P29319	1	1	NA	3112	NA	8637	3667	4864	4977	NA	NA	NA	0.912533912	0.039750987	5138.6667	4920.5	Down	-0.0625891474477412	Ephrin type-A receptor 3 OS=Mus musculus GN=Epha3 PE=1 SV=1
MT3	P28184	4	4	25228	26025.75	31239.75	21086.5	20684.25	20620.25	22941.5	26817.75	34011.5	17949	0.912810926	0.03961917	24852.85	24468	Down	-0.022515121106464	Metallothionein-3 OS=Mus musculus GN=Mt3 PE=1 SV=1
DDAH1	Q9CWS0	21	21	72497.25	68751.9	56158.85	69277.4	71132.25	79978.05	76815.6	44194.4	88653	42390.55	0.913247464	0.039411525	67563.53	66406.32	Down	-0.0249241549778336	N(G),N(G)-dimethylarginine dimethylaminohydrolase 1 OS=Mus musculus GN=Ddah1 PE=1 SV=3
SLK	O54988	14	14	29546	28160.1	12478	43892.7	31585.6	41504.7	39788.6	19575.5	37939.8	11277.1	0.913859947	0.039120357	29132.48	30017.14	Up	0.0431580041572407	STE20-like serine/threonine-protein kinase OS=Mus musculus GN=Slk PE=1 SV=2
CALML3	Q9D6P8	3	3	47638	68259.67	10740.67	154314.33	41985.67	82547.33	50099	31113.33	82260	NA	0.914034822	0.039037259	64587.668	61504.915	Down	-0.0705570271159292	Calmodulin-like protein 3 OS=Mus musculus GN=Calml3 PE=2 SV=1
RPL27	P61358	5	5	53022	54862.6	18018.2	86936.4	65171.4	69860.6	66232.4	20936.8	72811.6	NA	0.914049396	0.039030334	55602.12	57460.35	Up	0.0474268894434234	60S ribosomal protein L27 OS=Mus musculus GN=Rpl27 PE=1 SV=2
MACF1	Q9QXZ0-3	27	27	5531.23	11185.95	7709.23	30304.09	11808.32	19762.36	19752.64	8997.73	11457.73	9381.23	0.914494078	0.038819103	13307.764	13870.338	Up	0.0597347576793638	Isoform 3 of Microtubule-actin cross-linking factor 1 OS=Mus musculus GN=Macf1
FAM13C	Q9DBR2	1	1	19950	19893	NA	19915	43787	36992	12661	NA	24868	NA	0.91463629	0.038751571	25886.25	24840.333	Down	-0.0595014517389822	Protein FAM13C OS=Mus musculus GN=Fam13c PE=1 SV=2
ADD3	Q9QYB5	14	14	32186.57	38737.21	21845.43	55857.14	37716	45289	46790.93	34910.43	34521.79	28415.29	0.915888791	0.038157256	37268.47	37985.488	Up	0.027492763037052	Gamma-adducin OS=Mus musculus GN=Add3 PE=1 SV=2
ATP6V1B2	P62814	76	76	119420.32	131301.85	112182.94	130006.15	134620.71	139900.65	139727.31	122682.54	122366.28	106928.68	0.916371436	0.037928457	125506.39	126321.09	Up	0.00933468291026337	V-type proton ATPase subunit B, brain isoform OS=Mus musculus GN=Atp6v1b2 PE=1 SV=1
DHX30	Q99PU8-3	1	1	2980	3679	2079	16836	4183	7301	4975	NA	4627	NA	0.916577104	0.037830996	5951.4	5634.3333	Down	-0.0789841684903172	Isoform 3 of Putative ATP-dependent RNA helicase DHX30 OS=Mus musculus GN=Dhx30
PPP5C	Q60676	6	6	41433.17	44563.33	64803.67	53074.83	60054.67	65089.5	75319.17	38764	53095.33	36386.17	0.916796951	0.03772684	52785.934	53730.834	Up	0.0255966892266263	Serine/threonine-protein phosphatase 5 OS=Mus musculus GN=Ppp5c PE=1 SV=3
HNRNPUL2	Q00PI9	6	6	30606.67	28015.83	24114.5	18680.67	26138.17	28629.67	26370.5	13830.83	31291.17	NA	0.916872163	0.037691213	25511.168	25030.543	Down	-0.0274393929677128	Heterogeneous nuclear ribonucleoprotein U-like protein 2 OS=Mus musculus GN=Hnrnpul2 PE=1 SV=2
NUFIP2	Q5F2E7	4	4	13624	13468.33	7831	19413	22564.67	17295.67	19402.33	4885.33	21928.67	NA	0.917150071	0.037559596	15380.2	15878	Up	0.0459549375690615	Nuclear fragile X mental retardation-interacting protein 2 OS=Mus musculus GN=Nufip2 PE=1 SV=1
BSG	P18572-2	10	10	42846.6	55867.4	43393.2	113908.2	59697.8	86954.8	80668	43606.8	62580.9	32958	0.91759494	0.03734899	63142.64	61353.7	Down	-0.0414642313662903	Isoform 2 of Basigin OS=Mus musculus GN=Bsg
PKIA	P63248	5	5	25679.8	31645.4	26544.2	27226	25522.8	28709.8	26417.4	21467	39820.8	22107.8	0.917634067	0.037330472	27323.64	27704.56	Up	0.0199737651965268	cAMP-dependent protein kinase inhibitor alpha OS=Mus musculus GN=Pkia PE=1 SV=2
GFRA2	O08842	4	4	11288.33	17251	2038	18580.67	20408.33	19978	27416	5823.67	17238	2163.67	0.917905626	0.037201968	13913.266	14523.868	Up	0.0619646055314989	GDNF family receptor alpha-2 OS=Mus musculus GN=Gfra2 PE=2 SV=2
GFRA2	O08842-2	3	3	11288.33	17251	2038	18580.67	20408.33	19978	27416	5823.67	17238	2163.67	0.917905626	0.037201968	13913.266	14523.868	Up	0.0619646055314989	Isoform 2 of GDNF family receptor alpha-2 OS=Mus musculus GN=Gfra2
COX4I1	P19783	18	18	106048.11	199205.44	144811.06	497617.22	179901.78	336389.39	284512.39	275188.94	165831.06	109055.28	0.918308601	0.037011348	225516.72	234195.41	Up	0.054478400162102	Cytochrome c oxidase subunit 4 isoform 1, mitochondrial OS=Mus musculus GN=Cox4i1 PE=1 SV=2

GABBR1	Q9WV18	2	2	1844.5	5853	NA	33480.5	11924	28041	19009.5	7756.5	2187.5	NA	0.91856194	0.036891553	13275.5	14248.625	Up	0.102056506342182	Gamma-aminobutyric acid type B receptor subunit 1 OS=Mus musculus GN=Gabbr1 PE=1 SV=1
2310011J03RIK	Q9D7E4	3	3	24949.67	31637.33	32663.67	31951	25818.67	27417.33	27045	26908	35613.67	31285.33	0.918650093	0.036849877	29404.068	29653.866	Up	0.0122044392877626	UPF0449 protein C19orf25 homolog OS=Mus musculus PE=1 SV=1
TRP53BP1	P70399-3	1	1	5232	6246	NA	3577	11912	7711	6200	NA	NA	NA	0.918703223	0.03682476	6741.75	6955.5	Up	0.045031097656333	Isoform 3 of Tumor suppressor p53-binding protein 1 OS=Mus musculus GN=Tp53bp1
TPM3	P21107	30	30	49008.07	50732.32	28959.07	52985.14	44877.39	54151.04	50406.82	41971.32	62812.5	12071.29	0.919196273	0.036591745	45312.398	44282.594	Down	-0.0331661072071729	Tropomyosin alpha-3 chain OS=Mus musculus GN=Tpm3 PE=1 SV=3
KIF5C	P28738	29	29	24152.93	31328.48	26213.62	42927.76	33203.62	38337.93	39586.76	18860.48	35560.48	22654.9	0.919224099	0.036578598	31565.282	31000.11	Down	-0.0260653052717678	Kinesin heavy chain isoform 5C OS=Mus musculus GN=Kif5c PE=1 SV=3
DYNC1I1	O88485	10	10	9720.11	13067.78	10095.56	16322.44	13774	19379.44	18158.33	9592.11	13761.67	3747	0.919244939	0.036568752	12595.978	12927.71	Up	0.0375035976117109	Cytoplasmic dynein 1 intermediate chain 1 OS=Mus musculus GN=Dync1i1 PE=1 SV=2
APC	Q61315-3	8	8	10038.62	11538	5780.88	16667.38	12014.75	15380.88	16207.75	664	16476.25	5358	0.919662299	0.036371617	11207.926	10817.376	Down	-0.0511687523760005	Isoform 3 of Adenomatous polyposis coli protein OS=Mus musculus GN=Apc
CALR	P14211	34	34	77111.07	72628.47	92157.93	96437.9	62931	72485.77	69079.03	95758.77	64070.13	105169.07	0.919885553	0.036266202	80253.274	81312.554	Up	0.0189178605339313	Calreticulin OS=Mus musculus GN=Calr PE=1 SV=1
BEGAIN	Q68EF6	18	18	17076.64	17311.36	8533.5	16523.5	19307.36	16499	18120.93	2108.93	24132.14	NA	0.920366989	0.036038967	15750.472	15215.25	Down	-0.0498770242147293	Brain-enriched guanylate kinase-associated protein OS=Mus musculus GN=Begain PE=1 SV=2
KCNJ4	P52189	1	1	11651	44787	NA	98813	55594	80262	85393	16443	18003	NA	0.921520834	0.035494842	52711.25	50025.25	Down	-0.0754544327083129	Inward rectifier potassium channel 4 OS=Mus musculus GN=Kcnj4 PE=1 SV=1
EWSR1	Q61545	3	3	27367.33	31351	NA	24930.67	31923.67	29694	31922	12989	40482.67	31822	0.922741244	0.034920067	28893.168	29381.934	Up	0.0242009888534352	RNA-binding protein EWS OS=Mus musculus GN=Ewsr1 PE=1 SV=2
UQCR10	Q8R1I1	2	2	9985	14602.5	11489	36697.5	9984.5	21334	17157.5	NA	12887	NA	0.92276227	0.034910171	16551.7	17126.167	Up	0.0492228688571398	Cytochrome b-c1 complex subunit 9 OS=Mus musculus GN=Uqcr10 PE=1 SV=1
MRPS18B	Q99N84-2	6	6	24242	22747.83	29281	25990.17	23949.17	29797.33	29028.5	19071.17	26820	22662	0.922933538	0.034829572	25242.034	25475.8	Up	0.0132992839239805	Isoform 2 of 28S ribosomal protein S18b, mitochondrial OS=Mus musculus GN=Mrps18b
MADD	Q80U28-11	3	3	2965.33	4577.67	1127.33	12363	9085	10201.33	5164.33	1830.67	5831	NA	0.923627086	0.034503339	6023.666	5756.8325	Down	-0.065366541318264	Isoform 11 of MAP kinase-activating death domain protein OS=Mus musculus GN=Madd
APOOL	Q78IK4	1	1	3057	2881	NA	4044	2866	3918	2138	3884	2698	NA	0.924115217	0.034273878	3212	3159.5	Down	-0.0237756270462347	Apolipoprotein O-like OS=Mus musculus GN=Apool PE=2 SV=1
PGAM1	Q9DBJ1	19	19	163704.21	169429.37	145157.74	136477.84	256470.16	141831	166108	136515.53	148346.42	296646.89	0.924153735	0.034255777	174247.86	177889.57	Up	0.0298409378721433	Phosphoglycerate mutase 1 OS=Mus musculus GN=Pgam1 PE=1 SV=3
ZFP330	Q922H9	1	1	14481	15314	11236	9836	13635	12740	14473	5916	23882	9090	0.924743522	0.033978702	12900.4	13220.2	Up	0.0353282029417319	Zinc finger protein 330 OS=Mus musculus GN=Znf330 PE=2 SV=1
ATP1A1	Q8VDN2	102	102	27881.46	63329.18	31540.02	119801.41	59197.02	80305.62	96799.76	38566.04	43125.26	32788.55	0.924758543	0.033971648	60349.818	58317.046	Down	-0.0494317771439846	Sodium/potassium-transporting ATPase subunit alpha-1 OS=Mus musculus GN=Atp1a1 PE=1 SV=1
EMC2	Q9CRD2	3	3	14588	18711	10820	25303.67	15400.33	22355	18802	9270.33	15987.33	NA	0.924963106	0.033875589	16964.6	16603.665	Down	-0.0310256847041132	ER membrane protein complex subunit 2 OS=Mus musculus GN=Emc2 PE=2 SV=1
IMMT	Q8CAQ8-3	55	55	54095.53	68064.75	46591.44	124336.09	73216.73	91254.58	87534.02	63749.76	70078.75	45897.75	0.925026489	0.033845831	73260.908	71702.972	Down	-0.0310106639028938	Isoform 3 of Mitochondrial inner membrane protein OS=Mus musculus GN=Immt
ANP32A	O35381	3	3	37934	42669.33	16115.67	94133	30841.67	53374.33	36916.33	NA	38510.67	NA	0.925125893	0.033799164	44338.734	42933.777	Down	-0.0464544927115178	Acidic leucine-rich nuclear phosphoprotein 32 family member A OS=Mus musculus GN=Anp32a PE=1 SV=1
NRXN1	P0DI97	2	2	9381	16985	9494	66838	23544	30150.5	30418.5	NA	18536.5	NA	0.925696833	0.033531222	25248.4	26368.5	Up	0.0626235376357565	Neurexin-1-beta OS=Mus musculus GN=Nrxn1 PE=1 SV=1
SOD3	O09164	2	2	16788.5	60021.5	38256.5	7834.5	28060.5	14971	5397	77987	34617.5	25451	0.925862827	0.033453352	30192.3	31684.7	Up	0.0696056925532582	Extracellular superoxide dismutase [Cu-Zn] OS=Mus musculus GN=Sod3 PE=1 SV=1
MYEF2	Q8C854-3	4	4	20801.25	12453.25	17620.5	13331.75	13893	13642.5	10312	20364.5	7548.5	24532	0.926238578	0.033277135	15619.95	15279.9	Down	-0.0317547337492835	Isoform 2 of Myelin expression factor 2 OS=Mus musculus GN=Myef2
LPP	Q8BFW7	4	4	13240.33	12590	19981.67	10483	12322	13826.67	13678.67	3218	26120	NA	0.926738705	0.033042698	13723.4	14210.835	Up	0.0503533705235458	Lipoma-preferred partner homolog OS=Mus musculus GN=Lpp PE=1 SV=1
LPP	Q8BFW7-5	3	3	13240.33	12590	19981.67	10483	12322	13826.67	13678.67	3218	26120	NA	0.926738705	0.033042698	13723.4	14210.835	Up	0.0503533705235458	Isoform 5 of Lipoma-preferred partner homolog OS=Mus musculus GN=Lpp
EHD3	Q9QXY6	16	16	35280.67	49017.47	38757	63242	55777.6	60425.87	63100.8	45796.8	52276.67	15721.67	0.926828793	0.033000483	48414.948	47464.362	Down	-0.0286078538750937	EH domain-containing protein 3 OS=Mus musculus GN=Ehd3 PE=1 SV=2
THNSL1	Q8BH55	1	1	3963	2389	NA	9778	5171	7439	5637	NA	2265	NA	0.927105268	0.032870951	5325.25	5113.6667	Down	-0.0584911340517581	Threonine synthase-like 1 OS=Mus musculus GN=Thnsl1 PE=2 SV=1
PRUNE2	Q52KR3-3	1	1	2736	5281	NA	16078	8129	7960	11129	NA	6016	NA	0.927782756	0.032553703	8056	8368.3333	Up	0.0548766358272856	Isoform 3 of Protein prune homolog 2 OS=Mus musculus GN=Prune2
DNM1	P39053-3	58	58	44484.11	54099.63	41605.4	68905.28	51698	62750.56	61382.21	52114.88	47711.14	39818.82	0.928127163	0.032392517	52158.484	52755.522	Up	0.0164201729187854	Isoform 3 of Dynamin-1 OS=Mus musculus GN=Dnm1
ABLIM1	Q8K4G5-5	2	2	19354.5	26859.5	4888	30019	27985	28346	34686.5	3602.5	23721	NA	0.928167097	0.032373831	21821.2	22589	Up	0.0498899638997615	Isoform 5 of Actin-binding LIM protein 1 OS=Mus musculus GN=Ablim1
ACBD5	Q5XG73-3	1	1	16685	14840	7143	35964	17047	24730	22025	NA	10091	NA	0.928715563	0.032117277	18335.8	18948.667	Up	0.0474331230200035	Isoform 3 of Acyl-CoA-binding domain-containing protein 5 OS=Mus musculus GN=Acbd5
RABGAP1	A2AWA9	2	2	27100.5	36576	34620.5	106648	30796	57741.5	51504	36281.5	48922	NA	0.929217704	0.031882524	47148.2	48612.25	Up	0.044117216803683	Rab GTPase-activating protein 1 OS=Mus musculus GN=Rabgap1 PE=1 SV=1
SEPT10	Q8C650	2	2	93545.5	115308	16680	137759.5	121919.5	162904	107653.5	52513.5	84669	64278	0.929509964	0.03174595	97042.5	94403.6	Down	-0.0397748409454348	Septin-10 OS=Mus musculus GN=Sept10 PE=2 SV=1
GNAS	P63094-3	6	6	8904.5	17157.33	14822.67	23743.83	16601	23327	22467	9201	15293.5	9183.17	0.930249208	0.031400691	16245.866	15894.334	Down	-0.0315600834970015	Isoform Gnas-3 of Guanine nucleotide-binding protein G(s) subunit alpha isoforms short OS=Mus musculus GN=Gnas
CS	Q9CZU6	18	18	49047.61	63752.83	43050.28	80281.28	69335.78	84889	89890.61	39485.33	54875.61	30078.11	0.930274684	0.031388798	61093.556	59843.732	Down	-0.0298200681112852	Citrate synthase, mitochondrial OS=Mus musculus GN=Cs PE=1 SV=1
EIF4H	Q9WUK2-2	14	14	48011	46606.69	52821.92	27192.46	39209.31	32346.23	42354.15	37043.15	59584.92	39607.38	0.930396668	0.031331853	42768.276	42187.166	Down	-0.0197368778495739	Isoform Short of Eukaryotic translation initiation factor 4H OS=Mus musculus GN=Eif4h
AKR1A1	Q9JII6	9	9	47382.44	54171	59753.89	58522.44	49969	51789.33	59112.67	45135.33	65510.44	46183.56	0.930418235	0.031321787	53959.754	53546.266	Down	-0.0110977976609056	Alcohol dehydrogenase [NADP(+)] OS=Mus musculus GN=Akr1a1 PE=1 SV=3
NDEL1	Q9ERR1	13	13	30653.69	29221.31	17876.23	26021.38	31594.85	31567.08	32273.77	16230.54	40595.31	12023.62	0.930826223	0.03113139	27073.492	26538.064	Down	-0.0288178542899254	Nuclear distribution protein nudE-like 1 OS=Mus musculus GN=Ndel1 PE=1 SV=2
AKAP9	Q70FJ1-3	1	1	786	918	2201	4015	2425	3193	1096	NA	2146	NA	0.931563057	0.030787743	2069	2145	Up	0.0520440025268984	Isoform 3 of A-kinase anchor protein 9 OS=Mus musculus GN=Akap9
STOML2	Q99JB2	7	7	15904.57	17482.57	4114.29	23645.29	18846.29	23176.43	22391.71	1356.86	23639	6880.57	0.931603952	0.030768678	15998.602	15488.914	Down	-0.0467098505305314	Stomatin-like protein 2, mitochondrial OS=Mus musculus GN=Stoml2 PE=1 SV=1
SNX4	Q91YJ2	3	3	5570.67	5078.33	574.67	10730.33	6514.33	8369	7631.33	1006.33	4943.33	NA	0.931890616	0.030635061	5693.666	5487.4975	Down	-0.0532094872672339	Sorting nexin-4 OS=Mus musculus GN=Snx4 PE=2 SV=1
MAPK8IP3	Q9ESN9-3	4	4	16970.75	24122.75	10245.75	27986.25	24952.5	33259	32255.5	8089	21327	6450	0.932278513	0.030454325	20855.6	20276.1	Down	-0.0406546337737848	Isoform 1b of C-Jun-amino-terminal kinase-interacting protein 3 OS=Mus musculus GN=Mapk8ip3
VPS11	Q91W86	3	3	17510.67	30039.33	30256	29655	34013.33	36936.67	37157	22338	14505.33	NA	0.932644905	0.030283678	28294.866	27734.25	Down	-0.0288715929719621	Vacuolar protein sorting-associated protein 11 homolog OS=Mus musculus GN=Vps11 PE=1 SV=3
SDHB	Q9CQA3	24	24	58301.22	61795.52	52147.48	60705.35	58955.91	60022.48	57309.09	55472.48	70087.96	47228.78	0.932979518	0.03012789	58381.096	58024.158	Down	-0.00884761260776996	Succinate dehydrogenase [ubiquinone] iron-sulfur subunit, mitochondrial OS=Mus musculus GN=Sdhb PE=1 SV=1
HIST1H2BH	Q64478	48	48	328326.46	146376.23	241563.81	50681.96	182966.58	93721.52	105499.15	289581.5	120474.71	372379.25	0.93316828	0.030040032	189983.01	196331.23	Up	0.0474192578703198	Histone H2B type 1-H OS=Mus musculus GN=Hist1h2bh PE=1 SV=3
RBSN	Q80Y56	1	1	1352	1304	NA	5055	394	3153	1160	NA	NA	NA	0.933238151	0.030007516	2026.25	2156.5	Up	0.0898795303295967	Rabenosyn-5 OS=Mus musculus GN=Zfyve20 PE=2 SV=1
CAMK2B	P28652	46	46	63751.87	90785.09	78403.87	153201.07	99093.43	115851.59	126033.41	90272.46	88709.98	72201.48	0.933585278	0.029846005	97047.066	98613.784	Up	0.0231047202217767	Calcium/calmodulin-dependent protein kinase type II subunit beta OS=Mus musculus GN=Camk2b PE=1 SV=2
EIF4H	Q9WUK2	15	15	48296.43	47066.57	54651.71	25981.64	39386.79	33308.43	43787.14	37756.43	60805.79	36778.29	0.934275349	0.02952511	43076.628	42487.216	Down	-0.0198765081206708	Eukaryotic translation initiation factor 4H OS=Mus musculus GN=Eif4h PE=1 SV=3
OXR1	Q4KMM3-3	10	10	28436	32664.14	23206.57	44491.86	37031.57	38781.43	34274.86	20505.14	32846.14	37378.71	0.935248434	0.029073011	33166.028	32757.256	Down	-0.0178917329312676	Isoform 3 of Oxidation resistance protein 1 OS=Mus musculus GN=Oxr1
KCNAB1	P63143	1	1	12387	11841	8638	15195	19736	16915	16280	NA	6477	NA	0.935802136	0.028815968	13559.4	13224	Down	-0.0361347112552923	Voltage-gated potassium channel subunit beta-1 OS=Mus musculus GN=Kcnab1 PE=2 SV=2
CAMK1	Q91YS8	1	1	19558	63501	NA	46577	59008	42188	59273	NA	37005	NA	0.936270507	0.028598657	47161	46155.333	Down	-0.0310969459325469	Calcium/calmodulin-dependent protein kinase type 1 OS=Mus musculus GN=Camk1 PE=1 SV=1
KIF2A	P28740-1	20	20	27005.47	33594.47	32824.84	35911.26	31899.84	34823.63	37605.84	20737.79	31266.42	35430.05	0.937066799	0.028229449	32247.176	31972.746	Down	-0.0123301678893387	Isoform 1 of Kinesin-like protein KIF2A OS=Mus musculus GN=Kif2a
CACNA2D2	Q6PHS9-3	1	1	NA	11524	NA	10604	6348	6908	11572	NA	NA	NA	0.93726326	0.028138407	9492	9240	Down	-0.0388192488905282	Isoform 3 of Voltage-dependent calcium channel subunit alpha-2/delta-2 OS=Mus musculus GN=Cacna2d2
TBC1D10A	P58802	1	1	2267	8096	NA	15556	6784	11447	10541	NA	3465	NA	0.937459723	0.028047382	8175.75	8484.3333	Up	0.0534502208455909	TBC1 domain family member 10A OS=Mus musculus GN=Tbc1d10a PE=2 SV=1
STX1A	O35526	16	16	10663.64	40349.14	11739.21	112961.86	38663.64	83507.43	66670	20751.14	46434.43	6469.43	0.93776776	0.027904702	42875.498	44766.486	Up	0.0622656482540926	Syntaxin-1A OS=Mus musculus GN=Stx1a PE=1 SV=3
EPB41L3	Q9WV92-3	37	37	24911.14	28918.97	25941.78	46903.95	28616.86	38194.27	34027.35	22542.68	30402.19	28200.95	0.93866265	0.027490463	31058.54	30673.488	Down	-0.0179977827823381	Isoform 3 of Band 4.1-like protein 3 OS=Mus musculus GN=Epb41l3
RPL7	P14148	5	5	46180.6	47150.2	75298.6	43680.6	40235.4	42372.6	49757.2	63763.6	62227	37658.8	0.939019112	0.027325568	50509.08	51155.84	Up	0.0183561848566906	60S ribosomal protein L7 OS=Mus musculus GN=Rpl7 PE=1 SV=2
HDLBP	Q8VDJ3	8	8	41921.12	58908.5	32465.38	42809.62	57848.12	48730.88	60977.12	32162.88	63621	24814.25	0.939122168	0.027277908	46790.548	46061.226	Down	-0.0226643138147217	Vigilin OS=Mus musculus GN=Hdlbp PE=1 SV=1
UQCRQ	Q9CQ69	2	2	379776	409136.5	425815	330145.5	363046	305332	349259.5	367971	366307.5	536185	0.939210439	0.027237089	381583.8	385011	Up	0.0128997415212469	Cytochrome b-c1 complex subunit 8 OS=Mus musculus GN=Uqcrq PE=1 SV=3
GMPR	Q9DCZ1	1	1	30895	33916	54198	33763	34912	38525	27012	NA	45423	NA	0.939331912	0.027180923	37536.8	36986.667	Down	-0.0213003791871891	GMP reductase 1 OS=Mus musculus GN=Gmpr PE=2 SV=1
PKP4	Q68FH0	13	13	15100.67	17747.25	4434.33	24086.33	23594.17	27166.42	27081.5	10261.25	21983.75	942.33	0.93936161	0.027167193	16992.55	17487.05	Up	0.0413845646204566	Plakophilin-4 OS=Mus musculus GN=Pkp4 PE=1 SV=1
LARP1	Q6ZQ58	2	2	13248	14946	24400.5	13204.5	14268	14536.5	12187	16211	28393	10243.5	0.939572006	0.027069931	16013.4	16314.2	Up	0.0268485871427233	La-related protein 1 OS=Mus musculus GN=Larp1 PE=1 SV=2
PUM2	Q80U58-3	1	1	14765	24857	8897	54202	26644	33792	36014	15717	20867	NA	0.93977432	0.026976427	25873	26597.5	Up	0.0398433020731894	Isoform 3 of Pumilio homolog 2 OS=Mus musculus GN=Pum2
EIF3I	Q9QZD9	6	6	78563.83	87892.17	108874	68377.83	68385.17	77712	79262.17	90519	109993.17	59001.83	0.939778676	0.026974413	82418.6	83297.634	Up	0.0153055600709505	Eukaryotic translation initiation factor 3 subunit I OS=Mus musculus GN=Eif3i PE=1 SV=1
KRT10	P02535-3	2	2	NA	NA	NA	8142	786	5576	2837.5	NA	3932.5	NA	0.94017059	0.026793339	4464	4115.3333	Down	-0.117327736527354	Isoform 3 of Keratin, type I cytoskeletal 10 OS=Mus musculus GN=Krt10
DYNC1H1	Q9JHU4	19	19	10521	23244.84	12646.74	52918.84	23038.37	37652.84	39227.95	14015	19791.89	15269.11	0.940962339	0.026427759	24473.958	25191.358	Up	0.0416814589288461	Cytoplasmic dynein 1 heavy chain 1 OS=Mus musculus GN=Dync1h1 PE=1 SV=2
FAM162A	Q9D6U8	1	1	98892	119419	35492	259644	96872	189372	155875	26913	100318	NA	0.940995919	0.02641226	122063.8	118119.5	Down	-0.0473882544713101	Protein FAM162A OS=Mus musculus GN=Fam162a PE=2 SV=1
NACA	Q60817	2	2	105157	104811	55971	191808.5	105771.5	153356.5	131680	43055.5	132836	NA	0.941127418	0.026351574	112703.8	115232	Up	0.0320052505101243	Nascent polypeptide-associated complex subunit alpha OS=Mus musculus GN=Naca PE=1 SV=1
ABHD12	Q99LR1	2	2	14713	36048.5	6554.5	67789	31557	44294	44487.5	12832.5	27695	NA	0.941345038	0.026251162	31332.4	32327.25	Up	0.04509550461499	Monoacylglycerol lipase ABHD12 OS=Mus musculus GN=Abhd12 PE=1 SV=2
ADGRL3	Q80TS3-6	9	9	30113.44	33146.22	25045.89	29429.22	30813.78	31801.56	30302.89	26124.56	34507.11	26595.89	0.941403381	0.026224246	29709.71	29866.402	Up	0.00758892398359888	Isoform 6 of Latrophilin-3 OS=Mus musculus GN=Lphn3
ACTN4	P57780	37	37	10971.53	17588.81	14076.75	28025.89	18008.25	21391.47	23983.61	15415.28	16998.36	12226.61	0.941825738	0.026029445	17734.246	18003.066	Up	0.0217046316075387	Alpha-actinin-4 OS=Mus musculus GN=Actn4 PE=1 SV=1
PPM1A	P49443	7	7	32113.29	42847.71	35511.43	45458.71	36792.57	43828.71	47097.29	12498.29	45825.14	40827	0.941865509	0.026011107	38544.742	38015.286	Down	-0.0199544264240452	Protein phosphatase 1A OS=Mus musculus GN=Ppm1a PE=1 SV=1
BCAS2	Q9D287	1	1	4283	2424	3955	3908	2782	4186	3402	NA	2940	NA	0.942468576	0.025733121	3470.4	3509.3333	Up	0.0160950302291737	Pre-mRNA-splicing factor SPF27 OS=Mus musculus GN=Bcas2 PE=2 SV=1
SF3B1	Q99NB9	2	2	1089.5	369.5	NA	2635.5	2387	2014	1580.5	NA	1130.5	NA	0.942699337	0.025626799	1620.375	1575	Down	-0.0409759030362487	Splicing factor 3B subunit 1 OS=Mus musculus GN=Sf3b1 PE=1 SV=1
RHOG	P84096	2	2	24828.5	33500.5	24158	53416.5	27836.5	39134.5	37225	25714	36055	23277	0.943298159	0.025351013	32748	32281.1	Down	-0.0207170601973712	Rho-related GTP-binding protein RhoG OS=Mus musculus GN=Rhog PE=2 SV=1
ITGAV	P43406	2	2	16915	24252.5	21301.5	36423	20659	30101.5	29067.5	30042.5	23156.5	9096.5	0.943405931	0.025301398	23910.2	24292.9	Up	0.0229085263454436	Integrin alpha-V OS=Mus musculus GN=Itgav PE=1 SV=2
NDUFA9	Q9DC69	4	4	15533.75	23096	15744	44124.75	24069.5	36315.5	32440	7768.75	23954	NA	0.943426183	0.025292075	24513.6	25119.563	Up	0.0352289674582223	NADH dehydrogenase [ubiquinone] 1 alpha subcomplex subunit 9, mitochondrial OS=Mus musculus GN=Ndufa9 PE=1 SV=2
APOC3	P33622	1	1	23513	40455	8010	10321	43762	23801	28759	NA	24760	NA	0.944138279	0.024964394	25212.2	25773.333	Up	0.0317571245177577	Apolipoprotein C-III OS=Mus musculus GN=Apoc3 PE=1 SV=2
PCK2	Q8BH04	2	2	19250.5	22316	32785.5	14372.5	20411.5	22671.5	17936	19891.5	27756	22123	0.944995351	0.024570328	21827.2	22075.6	Up	0.0163255770602684	Phosphoenolpyruvate carboxykinase [GTP], mitochondrial OS=Mus musculus GN=Pck2 PE=1 SV=1
PSMD14	O35593	2	2	1848.5	4008	3321.5	4774	5005	5696	2158.5	NA	3780	NA	0.94519203	0.024479949	3791.4	3878.1667	Up	0.0326441338665602	26S proteasome non-ATPase regulatory subunit 14 OS=Mus musculus GN=Psmd14 PE=1 SV=2
CACNA2D3	Q9Z1L5	3	3	5794	9639.67	8329.33	23487	10659.33	15359.33	19331.33	3052	9922.67	NA	0.94526148	0.024448039	11581.866	11916.333	Up	0.0410725742808329	Voltage-dependent calcium channel subunit alpha-2/delta-3 OS=Mus musculus GN=Cacna2d3 PE=1 SV=1
PLBD2	Q3TCN2	2	2	3014	3895	1291	5371	3039	4018	3954	NA	1782	NA	0.945924476	0.024143537	3322	3251.3333	Down	-0.0310206016476646	Putative phospholipase B-like 2 OS=Mus musculus GN=Plbd2 PE=1 SV=2
HIST1H2BB	Q64475	45	45	348743.67	155234.6	254186.8	53366.09	193996.82	99455.49	111970.8	303828.64	126795.73	390278.31	0.946267198	0.023986214	201105.6	206465.79	Up	0.03794955759126	Histone H2B type 1-B OS=Mus musculus GN=Hist1h2bb PE=1 SV=3
DZANK1	Q8C008	2	2	11241	10267	8460	9319	15337	9599.5	15261	NA	7337.5	NA	0.946536406	0.023862677	10924.8	10732.667	Down	-0.0255982921428076	Double zinc ribbon and ankyrin repeat-containing protein 1 OS=Mus musculus GN=Dzank1 PE=1 SV=2
SNAP25	P60879-2	76	76	39934.29	53018.71	22444.01	103832.49	83129.69	89457.36	95485.64	27760.75	85113.89	12266.38	0.947622119	0.023364811	60471.838	62016.804	Up	0.0363957500334428	Isoform 2 of Synaptosomal-associated protein 25 OS=Mus musculus GN=Snap25
MVB12B	Q6KAU4	2	2	23441	18602	10694.5	26200.5	16502	21495	25834	7666.5	22686.5	NA	0.947782055	0.023291518	19088	19420.5	Up	0.0249143967016696	Multivesicular body subunit 12B OS=Mus musculus GN=Mvb12b PE=2 SV=2
SAMM50	Q8BGH2	3	3	57998.33	67913.67	44909.33	78315.33	66802.67	75425	78119.67	38390	63729.33	NA	0.948061653	0.023163419	63187.866	63916	Up	0.0165295806571773	Sorting and assembly machinery component 50 homolog OS=Mus musculus GN=Samm50 PE=1 SV=1
LCP1	Q61233	1	1	742	1293	NA	9331	2333	4910	2434	NA	2484	NA	0.948389672	0.023013184	3424.75	3276	Down	-0.0640633210433993	Plastin-2 OS=Mus musculus GN=Lcp1 PE=1 SV=4
SEC23IP	Q6NZC7	1	1	4290	11001	NA	27128	9970	14844	12059	NA	NA	NA	0.948451783	0.022984743	13097.25	13451.5	Up	0.038503135027647	SEC23-interacting protein OS=Mus musculus GN=Sec23ip PE=2 SV=2
NCAM2	P13595-2	43	43	20257.83	38324.81	26626.19	94058.88	28893.29	56646.31	58464.74	38792.9	37779.45	21549.12	0.948500189	0.022962578	41632.2	42646.504	Up	0.0347276818232647	Isoform 2 of Neural cell adhesion molecule 1 OS=Mus musculus GN=Ncam1
KIF3A	P28741	3	3	7425	13855.33	1496	31791	14312	24967.67	19881.67	6724.33	8967	10400	0.948584973	0.02292376	13775.866	14188.134	Up	0.0425418464048504	Kinesin-like protein KIF3A OS=Mus musculus GN=Kif3a PE=1 SV=2
DNM1L	Q8K1M6	17	17	20692.94	28339.12	19122.75	57672.31	31128.94	40586.5	42885.25	17912.88	27749.19	30562.19	0.949200033	0.022642255	31391.212	31939.202	Up	0.0249675357317755	Dynamin-1-like protein OS=Mus musculus GN=Dnm1l PE=1 SV=2
NCEH1	Q8BLF1	1	1	NA	1388	NA	6775	1557	4245	3911	NA	1963	NA	0.949325338	0.022584928	3240	3373	Up	0.0580385052461395	Neutral cholesterol ester hydrolase 1 OS=Mus musculus GN=Nceh1 PE=1 SV=1
LY6H	Q9WUC3	1	1	6175	13627	NA	52761	12842	24134	26799	NA	15384	NA	0.949632419	0.022444468	21351.25	22105.667	Up	0.0500957097083504	Lymphocyte antigen 6H OS=Mus musculus GN=Ly6h PE=2 SV=2
TXNL1	Q8CDN6	8	8	29402	29911.17	23397	20427.17	23956.17	23433.67	28943.17	25013.5	37332.33	10831.17	0.949900295	0.022321977	25418.702	25110.768	Down	-0.0175842079687314	Thioredoxin-like protein 1 OS=Mus musculus GN=Txnl1 PE=2 SV=3
STXBP1	O08599	73	73	62829.24	85525.35	55224.39	100339.24	90067.35	88834.28	106929.28	66483.92	73140.4	54598.88	0.950404625	0.022091459	78797.114	77997.352	Down	-0.0147176454271216	Syntaxin-binding protein 1 OS=Mus musculus GN=Stxbp1 PE=1 SV=2
GPX4	O70325	3	3	66465.67	76839	105882.33	66616.33	63059	77092	60556.67	128080.67	75533.67	31965.67	0.950774818	0.021922329	75772.466	74645.736	Down	-0.0216138494613656	Phospholipid hydroperoxide glutathione peroxidase, mitochondrial OS=Mus musculus GN=Gpx4 PE=1 SV=4
SLC39A6	Q8C145	2	2	53523.5	63779	58638	63616.5	79396	74509	95382	30459	103137	21071	0.951012992	0.02181355	63790.6	64911.6	Up	0.0251324692247982	Zinc transporter ZIP6 OS=Mus musculus GN=Slc39a6 PE=1 SV=1
STXBP1	O08599-2	74	74	62045.19	84460.1	54512.03	99166.01	88949.37	87765.92	105611.53	65612.41	72239.16	54014.19	0.951171842	0.021741015	77826.54	77048.642	Down	-0.0144926903081719	Isoform 2 of Syntaxin-binding protein 1 OS=Mus musculus GN=Stxbp1
RTN4	Q99P72	27	27	22085.5	35908.04	22670.85	71351.92	35385.04	48930.54	49515.54	23565.62	37355.58	31297.46	0.951376895	0.0216474	37480.27	38132.948	Up	0.0249067220199303	Reticulon-4 OS=Mus musculus GN=Rtn4 PE=1 SV=2
KCTD16	Q5DTY9	2	2	5853	6836.5	488.5	17897	8048.5	8044.5	9415	NA	6582.5	NA	0.951443714	0.021616899	7824.7	8014	Up	0.0344870697757398	BTB/POZ domain-containing protein KCTD16 OS=Mus musculus GN=Kctd16 PE=1 SV=2

BAG5	Q8CI32	1	1	18282	32634	NA	56779	44473	43502	53166	15847	36755	NA	0.951445781	0.021615955	38042	37317.5	Down	-0.0277407562847738	BAG family molecular chaperone regulator 5 OS=Mus musculus GN=Bag5 PE=1 SV=1
KTN1	Q61595-15	15	15	17921.2	28890.8	17834.2	69809.4	35698.87	52767	53875.2	17784.6	36195.2	5301.93	0.951704551	0.021497854	34030.894	33184.786	Down	-0.0363230810582152	Isoform 15 of Kinectin OS=Mus musculus GN=Ktn1
PTPN5	P54830-3	1	1	21874	31700	12455	98878	40022	60430	39473	28154	31626	NA	0.951725571	0.021488262	40985.8	39920.75	Down	-0.0379853329095818	Isoform STEP38 of Tyrosine-protein phosphatase non-receptor type 5 OS=Mus musculus GN=Ptpn5
NDE1	Q9CZA6-3	3	3	76800.67	67839	46351.33	58029.67	81834.67	67115	79172	14240	99200.33	NA	0.951938178	0.021391255	66171.068	64931.833	Down	-0.0272746392761119	Isoform 3 of Nuclear distribution protein nudE homolog 1 OS=Mus musculus GN=Nde1
NTM	Q99PJ0	35	35	16238.27	22103.06	17593.42	58764.36	18330.24	33463.48	35681.64	16761.58	21547.15	28361.03	0.952013811	0.021356751	26605.87	27162.976	Up	0.0298969714318755	Neurotrimin OS=Mus musculus GN=Ntm PE=2 SV=2
MRPS18B	Q99N84	7	7	21945	21248.57	25098	26031.71	21644.71	28208	26348	16346.71	24877.43	19424.57	0.95216898	0.021285971	23193.598	23040.942	Down	-0.00952694044252637	28S ribosomal protein S18b, mitochondrial OS=Mus musculus GN=Mrps18b PE=2 SV=1
RPL35	Q6ZWV7	3	3	148204	192329	144745	103343.33	147464.67	138872.67	187228.33	93904	207094.67	116867	0.952397726	0.02118165	147217.2	148793.33	Up	0.015363657261257	60S ribosomal protein L35 OS=Mus musculus GN=Rpl35 PE=2 SV=1
ATP2A2	O55143	38	38	25937.5	48236.11	43150.37	115189.37	40702.89	69271.18	77052.32	50150.68	42202.63	39899.76	0.952591135	0.021093464	54643.248	55715.314	Up	0.0280306834195742	Sarcoplasmic/endoplasmic reticulum calcium ATPase 2 OS=Mus musculus GN=Atp2a2 PE=1 SV=2
OGFR	Q99PG2	2	2	2970	NA	NA	7387	3832	5023	4252	NA	NA	NA	0.952968512	0.020921449	4729.6667	4637.5	Down	-0.0283912284078189	Opioid growth factor receptor OS=Mus musculus GN=Ogfr PE=2 SV=1
GRM5	Q3UVX5-2	2	2	2378	3171	NA	35956	3478	13333	10218	NA	NA	NA	0.953364858	0.020740861	11245.75	11775.5	Up	0.0664084391314065	Isoform 2 of Metabotropic glutamate receptor 5 OS=Mus musculus GN=Grm5
UBE2K	P61087	1	1	17393	16471	23165	17262	13668	18096	16604	12028	13181	29083	0.953548594	0.02065717	17591.8	17798.4	Up	0.0168444474147456	Ubiquitin-conjugating enzyme E2 K OS=Mus musculus GN=Ube2k PE=1 SV=3
KTN1	Q61595	16	16	17052.88	27598.62	17021.69	67430.62	34132.38	51105.81	51789.25	16673.06	34754.75	4970.56	0.953627667	0.020621158	32647.238	31858.686	Down	-0.0352741783500564	Kinectin OS=Mus musculus GN=Ktn1 PE=2 SV=1
CSNK1D	Q9DC28	4	4	19810.25	32790.5	14093	30938.5	33831.25	35251.5	35004.25	9515.25	46602.25	7717.75	0.953680664	0.020597023	26292.7	26818.2	Up	0.0285501087608283	Casein kinase I isoform delta OS=Mus musculus GN=Csnk1d PE=1 SV=2
PALM2	Q8BR92	4	4	82776.75	97937.25	58187.25	100255	113764.25	123103.75	148764.5	35931	120685.25	32345	0.953741499	0.02056932	90584.1	92165.9	Up	0.0249752340085081	Paralemmin-2 OS=Mus musculus GN=Palm2 PE=1 SV=1
SPCS2	Q9CYN2	2	2	11670	20162	NA	36299	10435	21984	21834	NA	13908	NA	0.9540865	0.020412249	19641.5	19242	Down	-0.0296463512982361	Signal peptidase complex subunit 2 OS=Mus musculus GN=Spcs2 PE=1 SV=1
DNAJB14	Q149L6	1	1	47261	37559	68288	25364	42156	35906	38252	32520	31946	85717	0.954121274	0.020396421	44125.6	44868.2	Up	0.0240774122238694	DnaJ homolog subfamily B member 14 OS=Mus musculus GN=Dnajb14 PE=2 SV=1
HIST3H2BA	Q9D2U9	40	40	440031.03	196276.08	283438.72	70838.98	250856.23	141625.88	148593.8	348844.58	172591.38	455554.88	0.954338699	0.020297465	248288.21	253442.1	Up	0.02964047245301	Histone H2B type 3-A OS=Mus musculus GN=Hist3h2ba PE=1 SV=3
INA	P46660	68	68	122987.72	142448.44	150639.37	88151.22	119950.41	94687.07	105757.5	158155.76	127384.22	142864.13	0.954604971	0.020176308	124835.43	125769.74	Up	0.0107573359589517	Alpha-internexin OS=Mus musculus GN=Ina PE=1 SV=3
SNRNP200	Q6P4T2	3	3	1306	4402	NA	6226	5544	5891	6209	NA	1354	NA	0.954922888	0.020031697	4369.5	4484.6667	Up	0.037532555248694	U5 small nuclear ribonucleoprotein 200 kDa helicase OS=Mus musculus GN=Snrnp200 PE=1 SV=1
TXNRD2	Q9JLT4-4	7	7	39737.29	46513.57	45856.86	54100.57	38370.71	58404.71	47827.14	46929.14	58608.14	15304.14	0.955010989	0.019991631	44915.8	45414.654	Up	0.015934858660037	Isoform 4 of Thioredoxin reductase 2, mitochondrial OS=Mus musculus GN=Txnrd2
HNRNPC	Q9Z204-3	11	11	155003.18	101080.73	111665.64	48456.91	111922.45	82181.82	82681.64	166977.27	99904	103060.45	0.955336748	0.019843516	105625.78	106961.04	Up	0.0181233218034284	Isoform 3 of Heterogeneous nuclear ribonucleoproteins C1/C2 OS=Mus musculus GN=Hnrnpc
LARP4	Q8BWW4	4	4	26005	33600.5	47444.75	15165	24643.75	22834.75	34480	22902.25	34846.75	33565.25	0.95540318	0.019813317	29371.8	29725.8	Up	0.0172839557934238	La-related protein 4 OS=Mus musculus GN=Larp4 PE=2 SV=2
PPFIA2	Q8BSS9	21	21	17023.72	20980.33	10659.89	23608.22	23454.22	26311.56	25046.94	10180.67	27587.78	8013.28	0.955727128	0.019666087	19145.276	19428.046	Up	0.0211523490054758	Liprin-alpha-2 OS=Mus musculus GN=Ppfia2 PE=1 SV=2
WFS1	P56695	8	8	10878.38	26165.38	14135.88	95184.5	26367.88	59587.88	50325.75	32061	26637.75	9243.88	0.955965262	0.019557889	34546.404	35571.252	Up	0.0421762081801054	Wolframin OS=Mus musculus GN=Wfs1 PE=1 SV=1
AKAP2	O54931-3	2	2	16517.5	17508.5	5629.5	29280.5	22643.5	18798	17176.5	NA	19665	NA	0.956240069	0.019433062	18315.9	18546.5	Up	0.0180503611416274	Isoform 3 of A-kinase anchor protein 2 OS=Mus musculus GN=Akap2
PPP1R12C	Q3UMT1	5	5	10626.2	15395	14325	14810.6	13478.4	16360.2	13770.8	3510.6	20399.2	NA	0.956581581	0.019277986	13727.04	13510.2	Down	-0.0229715347122379	Protein phosphatase 1 regulatory subunit 12C OS=Mus musculus GN=Ppp1r12c PE=1 SV=1
ANXA3	O35639	3	3	65093.5	53207	43345.5	37746	54077.5	51586	48415.5	71401	55470.5	24088.5	0.957065197	0.019058476	50693.9	50192.3	Down	-0.0143461007662978	Annexin A3 OS=Mus musculus GN=Anxa3 PE=1 SV=4
WIPF2	Q6PEV3	1	1	34472	28023	60260	34516	31795	42210	29044	NA	40996	NA	0.957334367	0.01893635	37813.2	37416.667	Down	-0.0152089056376628	WAS/WASL-interacting protein family member 2 OS=Mus musculus GN=Wipf2 PE=2 SV=1
ACP6	Q8BP40	1	1	1685	3770	NA	2382	2166	3344	1531	NA	NA	NA	0.957341323	0.018933195	2500.75	2437.5	Down	-0.0369586196290851	Lysophosphatidic acid phosphatase type 6 OS=Mus musculus GN=Acp6 PE=2 SV=1
IQSEC1	Q8R0S2	17	17	34504.2	50511.73	26183.93	71353.47	64860.6	68632.53	78312.53	42448	49938.07	12054.33	0.957371547	0.018919484	49482.786	50277.092	Up	0.0229744776595208	IQ motif and SEC7 domain-containing protein 1 OS=Mus musculus GN=Iqsec1 PE=1 SV=2
ANKS1B	Q8BIZ1	6	6	37818.33	47971	88411.17	38684.83	51530.5	41374.67	60048.33	64332.67	57806.67	43735.17	0.957400252	0.018906463	52883.166	53459.502	Up	0.0156378478297741	Ankyrin repeat and sterile alpha motif domain-containing protein 1B OS=Mus musculus GN=Anks1b PE=1 SV=3
MAP2	P20357	135	135	54608.62	50592.46	49271.41	60506.93	55924.79	57205.62	61829.7	43120.06	62604.56	47348.31	0.958164188	0.018560065	54180.842	54421.65	Up	0.00639788407005248	Microtubule-associated protein 2 OS=Mus musculus GN=Map2 PE=1 SV=2
VPS52	Q8C754	2	2	3707	5640.5	3107.5	12274.5	6443	9023	8387	2611	4431.5	NA	0.958433403	0.018438059	6234.5	6113.125	Down	-0.028363794794637	Vacuolar protein sorting-associated protein 52 homolog OS=Mus musculus GN=Vps52 PE=2 SV=1
ATP1A3	Q6PIC6	187	187	27695.2	68721.05	33236.54	152058.81	67469.95	99293.33	114964.72	43714.27	50751.66	33036.46	0.958470522	0.018421239	69836.31	68352.088	Down	-0.0309919225475263	Sodium/potassium-transporting ATPase subunit alpha-3 OS=Mus musculus GN=Atp1a3 PE=1 SV=1
VCAM1	P29533-2	8	8	35653.38	44023.88	41385.5	39862.75	52039.12	58393.62	57491	23557.62	57453.12	18646.25	0.958611277	0.018357466	42592.926	43108.322	Up	0.0173525645156113	Isoform 2 of Vascular cell adhesion protein 1 OS=Mus musculus GN=Vcam1
GADD45GIP1	Q9CR59	1	1	35698	24456	46304	14697	31597	25294	30260	20132	46182	29045	0.958718777	0.018308766	30550.4	30182.6	Down	-0.017474181190075	Growth arrest and DNA damage-inducible proteins-interacting protein 1 OS=Mus musculus GN=Gadd45gip1 PE=2 SV=1
HIST1H2BK	Q8CGP1	41	41	388541	176643.61	283208.61	59996.12	219786.98	113589.46	130374.95	334849.07	147226.05	424349.54	0.958805883	0.01826931	225635.26	230077.81	Up	0.0281293129743319	Histone H2B type 1-K OS=Mus musculus GN=Hist1h2bk PE=1 SV=3
KIF5B	Q61768	14	14	19366.38	24068.54	13382.31	37200.23	28239.77	32881.85	29762.31	16454.15	28231.31	16345.92	0.958890617	0.018230931	24451.446	24735.108	Up	0.0166404134645807	Kinesin-1 heavy chain OS=Mus musculus GN=Kif5b PE=1 SV=3
BSDC1	Q80Y55	1	1	744	1098	NA	2481	1500	2111	885	NA	NA	NA	0.958968536	0.018195642	1455.75	1498	Up	0.0412750050239313	BSD domain-containing protein 1 OS=Mus musculus GN=Bsdc1 PE=2 SV=1
BAD	Q61337	3	3	20067.33	16484.67	34905.33	10047.67	16318	18390	13417.67	20923.67	26648.33	19693.33	0.959097308	0.018137328	19564.6	19814.6	Up	0.0183182292098286	Bcl2 antagonist of cell death OS=Mus musculus GN=Bad PE=1 SV=1
NCAM1	P13595	48	48	20095.62	36578.6	25544.15	88146.89	28105.43	53937.13	56819.43	35857.09	36332.15	19256.66	0.959628573	0.017896829	39694.138	40440.492	Up	0.0268745822140389	Neural cell adhesion molecule 1 OS=Mus musculus GN=Ncam1 PE=1 SV=3
CORO1B	Q9WUM3	6	6	23308	24405.5	21271.33	20242.83	26055.17	26072.5	25535.83	21237.83	24204.33	18687.5	0.959768971	0.017833295	23056.566	23147.598	Up	0.00568483762172928	Coronin-1B OS=Mus musculus GN=Coro1b PE=1 SV=1
FAM131B	Q3TY60-2	12	12	16763.55	16982.82	13966.36	26259.09	20397.09	21874.27	23151	13720	22995.36	13426	0.959925106	0.01776265	18873.782	19033.326	Up	0.0121441444029064	Isoform 2 of Protein FAM131B OS=Mus musculus GN=Fam131b
SEP15	Q9ERR7	2	2	11158	17264	2681.5	29842	14217.5	19248.5	14386	NA	10665.5	NA	0.960011574	0.017723531	15032.6	14766.667	Down	-0.0257503576159863	15 kDa selenoprotein OS=Mus musculus GN=Sep15 PE=1 SV=3
ADGRL3	Q80TS3-3	20	20	22883	28294	18094.68	29096.26	26008.42	30378.26	29192.74	21207.89	30458	14124.74	0.960216139	0.017630999	24875.272	25072.326	Up	0.0113835425503234	Isoform 3 of Latrophilin-3 OS=Mus musculus GN=Lphn3
TOP1	Q04750	1	1	5397	2843	1617	1619	2811	751	1165	4431	1734	6541	0.96058051	0.017466229	2857.4	2924.4	Up	0.0334376473541736	DNA topoisomerase 1 OS=Mus musculus GN=Top1 PE=1 SV=2
SNX7	Q9CY18	1	1	3318	3535	NA	10714	7483	6802	7812	NA	3842	NA	0.960696178	0.017413937	6262.5	6152	Down	-0.0256831950081066	Sorting nexin-7 OS=Mus musculus GN=Snx7 PE=2 SV=1
FSD1L	Q8BYN5-3	2	2	93213	78382	108280	88259	74781	83469	99469	78382	91482	NA	0.960879949	0.017330869	88583	88200.5	Down	-0.00624302284164966	Isoform 3 of FSD1-like protein OS=Mus musculus GN=Fsd1l
BCL11A	Q9QYE3	3	3	23729.67	15341	13123	14475.67	28186.67	23956.67	19034.67	5954.33	28097.33	NA	0.961019842	0.017267646	18971.202	19260.75	Up	0.0218527921658976	B-cell lymphoma/leukemia 11A OS=Mus musculus GN=Bcl11a PE=1 SV=1
NPTXR	Q99J85	15	15	22621.47	33194.47	10363.13	51326.4	28146.2	46494.07	44069.6	15443	36215.13	6071.93	0.961066336	0.017246635	29130.334	29658.746	Up	0.0259353574651094	Neuronal pentraxin receptor OS=Mus musculus GN=Nptxr PE=1 SV=1
VTA1	Q9CR26	1	1	52996	60347	25483	88686	65383	67282	78819	NA	26784	NA	0.962260648	0.016707274	58579	57628.333	Down	-0.0236052690286078	Vacuolar protein sorting-associated protein VTA1 homolog OS=Mus musculus GN=Vta1 PE=1 SV=1
MTHFD1	Q922D8	2	2	20984	26710.5	2427	43495.5	18226	33955.5	25053.5	14713.5	17305	NA	0.962400097	0.016644342	22368.6	22756.875	Up	0.0248274945109349	C-1-tetrahydrofolate synthase, cytoplasmic OS=Mus musculus GN=Mthfd1 PE=1 SV=4
STX6	Q9JKK1	1	1	NA	1280	NA	3356	433	2091	1188	NA	NA	NA	0.962535413	0.016583283	1689.6667	1639.5	Down	-0.0434827614752596	Syntaxin-6 OS=Mus musculus GN=Stx6 PE=1 SV=1
NDUFB8	Q9D6J5	1	1	579	778	NA	9041	1820	3334	2569	NA	NA	NA	0.962894249	0.016421407	3054.5	2951.5	Down	-0.0494878986326786	NADH dehydrogenase [ubiquinone] 1 beta subcomplex subunit 8, mitochondrial OS=Mus musculus GN=Ndufb8 PE=1 SV=1
SSSCA1	P56873	1	1	92757	71913	69917	45980	93684	76921	86452	39774	94108	80024	0.963595506	0.016105234	74850.2	75455.8	Up	0.0116256283128976	Sjoegren syndrome/scleroderma autoantigen 1 homolog OS=Mus musculus GN=Sssca1 PE=2 SV=1
UQCC2	Q9CQY6	6	6	18376.33	17662.5	13002.83	20560	20173.33	21715	16591.17	21037.83	22505.83	8604.17	0.964356969	0.015762177	17954.998	18090.8	Up	0.0108707150228104	Ubiquinol-cytochrome-c reductase complex assembly factor 2 OS=Mus musculus GN=Uqcc2 PE=1 SV=1
PCCA	Q91ZA3	11	11	100586.09	91492.55	106348.82	81711.18	111173	103911.36	105280.45	88648.55	110633.55	84520.55	0.964464375	0.01571381	98262.328	98598.892	Up	0.00493301511413981	Propionyl-CoA carboxylase alpha chain, mitochondrial OS=Mus musculus GN=Pcca PE=1 SV=2
BOLA2	Q8BGS2	3	3	35276.33	30854.67	35938	29258.33	38444.33	35294	36232	14248.33	48644.67	NA	0.96468291	0.015615415	33954.332	33604.75	Down	-0.0149304787505818	BolA-like protein 2 OS=Mus musculus GN=Bola2 PE=1 SV=1
NDUFB7	Q9CR61	12	12	61976.08	63726.67	82086.25	38974.08	59329.42	54602.83	56223.92	45272.25	66933.58	85226.58	0.965370728	0.015305874	61218.5	61651.832	Up	0.0101760695071178	NADH dehydrogenase [ubiquinone] 1 beta subcomplex subunit 7 OS=Mus musculus GN=Ndufb7 PE=1 SV=3
PMPCA	Q9DC61	5	5	40917.2	54032.6	27949	58990.2	45044	60836.4	58747	35965	53385.6	20112.6	0.965602255	0.015201729	45386.6	45809.32	Up	0.0133747302625354	Mitochondrial-processing peptidase subunit alpha OS=Mus musculus GN=Pmpca PE=1 SV=1
BCAT1	P24288	1	1	4927	5863	3016	25396	7521	13314	7839	6871	10138	NA	0.965624317	0.015191806	9344.6	9540.5	Up	0.0299319671449633	Branched-chain-amino-acid aminotransferase, cytosolic OS=Mus musculus GN=Bcat1 PE=2 SV=2
RRAS2	P62071	2	2	10779.5	16330	8990.5	22836.5	13907.5	19373	17849.5	8042	12379.5	NA	0.965879877	0.015076882	14568.8	14411	Down	-0.015711601011564	Ras-related protein R-Ras2 OS=Mus musculus GN=Rras2 PE=1 SV=1
TPM3	P21107-2	51	51	56273.81	56365.08	35160.42	60227.96	52448.5	59634.33	57089.98	48577.52	70135.65	23030.04	0.966132363	0.01496337	52095.154	51693.504	Down	-0.0111661787910418	Isoform 2 of Tropomyosin alpha-3 chain OS=Mus musculus GN=Tpm3
ANKS1B	Q8BIZ1-4	5	5	44513.2	56330.4	104803.2	44990.4	60234.8	47521.2	70441.8	77199.2	68111.8	50365	0.96621514	0.014926162	62174.4	62727.8	Up	0.0127842849848784	Isoform 4 of Ankyrin repeat and sterile alpha motif domain-containing protein 1B OS=Mus musculus GN=Anks1b
KTN1	Q61595-9	18	18	14967.72	24608.33	14861.83	59670.39	30473.28	45286.5	46108.39	14958.72	31262.72	4418.28	0.96624033	0.01491484	28916.31	28406.922	Down	-0.0256409438337079	Isoform 9 of Kinectin OS=Mus musculus GN=Ktn1
TLN2	Q71LX4	46	46	17019.24	22992.33	17386.22	38844.78	28887.24	33189.67	33883.07	17300.67	26180.31	13311.98	0.966366787	0.014858005	25025.962	24773.14	Down	-0.0146487854081211	Talin-2 OS=Mus musculus GN=Tln2 PE=1 SV=3
KXD1	Q80XH1	1	1	29717	25833	18825	16770	30149	23356	26934	27321	30233	12564	0.966893075	0.01462155	24258.8	24081.6	Down	-0.0105769379774869	KxDL motif-containing protein 1 OS=Mus musculus GN=Kxd1 PE=1 SV=1
PSD3	Q2PFD7-5	15	15	93411.53	94283.4	82602.67	122079.67	82998.47	92056.47	98726.87	99710.73	115080.47	67541.53	0.966955184	0.014593654	95075.148	94623.214	Down	-0.00687411496096556	Isoform 5 of PH and SEC7 domain-containing protein 3 OS=Mus musculus GN=Psd3
PPP1R9B	Q6R891	40	40	49234.43	48117.53	35470.1	51822.55	56070.93	52828.8	59114.57	36543.78	65038.4	25454.75	0.967319732	0.014429953	48143.108	47796.06	Down	-0.0104375850425113	Neurabin-2 OS=Mus musculus GN=Ppp1r9b PE=1 SV=1
HNRNPC	Q9Z204	11	11	155212.27	101642.18	112130.45	48815.36	111848.55	81886	82415.64	166977.27	100176.45	103060.45	0.967435454	0.014378001	105929.76	106903.16	Up	0.0131965404415961	Heterogeneous nuclear ribonucleoproteins C1/C2 OS=Mus musculus GN=Hnrnpc PE=1 SV=1
KTN1	Q61595-6	19	19	14391.95	23745.58	14334.05	58200.84	29429.16	44281.32	44760.58	14171.42	30309.32	4185.74	0.967623394	0.01429364	28020.316	27541.676	Down	-0.0248568710991481	Isoform 6 of Kinectin OS=Mus musculus GN=Ktn1
MESDC2	Q9ERE7	3	3	17889.33	24098.33	17264	42154	22947	31119.67	30479.67	5633.33	30934.67	NA	0.967648907	0.01428219	24870.532	24541.835	Down	-0.0191942444992503	LDLR chaperone MESD OS=Mus musculus GN=Mesdc2 PE=1 SV=1
ARHGAP23	Q69ZH9	7	7	8677.6	9616.8	4757.2	9732.8	9217.6	11299.4	10416	5545.8	13091.6	1146	0.967672338	0.014271673	8400.4	8299.76	Down	-0.0173884066462822	Rho GTPase-activating protein 23 OS=Mus musculus GN=Arhgap23 PE=2 SV=2
BMPR2	O35607	1	1	NA	NA	NA	17327	1417	11808	6112	NA	NA	NA	0.967706726	0.014256241	9372	8960	Down	-0.0648582219181691	Bone morphogenetic protein receptor type-2 OS=Mus musculus GN=Bmpr2 PE=1 SV=1
ERC2	Q6PH08-4	7	7	52986.71	42933.57	38531.43	36710.86	53991	50613	57150.43	30306	75109	9448.86	0.967794277	0.01421695	45030.714	44525.458	Down	-0.0162789016840458	Isoform 4 of ERC protein 2 OS=Mus musculus GN=Erc2
PHACTR1	Q2M3X8	12	12	31636.36	33511.64	41352.91	29492.36	32234.91	38278.82	38241.55	22948.18	48883.36	18639.18	0.968169054	0.014048803	33645.636	33398.218	Down	-0.0106482644051452	Phosphatase and actin regulator 1 OS=Mus musculus GN=Phactr1 PE=1 SV=1
ALDH1L1	Q8R0Y6	12	12	21136.67	24117	18984.75	32661.5	24081.08	33189.83	28154.17	16757.58	20365.83	23260.08	0.968944244	0.013701213	24196.2	24345.498	Up	0.0088745211755542	Cytosolic 10-formyltetrahydrofolate dehydrogenase OS=Mus musculus GN=Aldh1l1 PE=1 SV=1
SORBS1	Q62417-3	4	4	18880	26394.67	3112	33896.33	30926.67	31002	26300.33	2910.33	28973.33	NA	0.968974901	0.013687472	22641.934	22296.498	Down	-0.0221800950733907	Isoform 3 of Sorbin and SH3 domain-containing protein 1 OS=Mus musculus GN=Sorbs1
CD47	Q61735	5	5	11927.6	12620.6	7082	43853.4	15979.2	27205.8	31531.8	3610.2	24508.6	2829.6	0.969328922	0.013528829	18292.56	17937.2	Down	-0.0283022880455453	Leukocyte surface antigen CD47 OS=Mus musculus GN=Cd47 PE=1 SV=2
D630045J12RIK	Q68FD9	3	3	25395.67	38920.67	22945.67	47682.33	48427.67	50656.33	53673.33	12749.33	42396.67	21971	0.969474025	0.013463822	36674.402	36289.332	Down	-0.0152279415197403	UPF0606 protein KIAA1549 OS=Mus musculus GN=Kiaa1549 PE=1 SV=3
AMPD3	O08739	1	1	11373	16386	NA	68228	24996	47710	34567	NA	10477	NA	0.969887023	0.013278851	30245.75	30918	Up	0.0317145628692753	AMP deaminase 3 OS=Mus musculus GN=Ampd3 PE=2 SV=2
SLC4A4	O88343-3	2	2	11798.5	18581	11823.5	129846	31790	60822	41785	42068	14551	NA	0.970122697	0.013173334	40767.8	39806.5	Down	-0.0344260775486463	Isoform 3 of Electrogenic sodium bicarbonate cotransporter 1 OS=Mus musculus GN=Slc4a4
NRXN1	Q9CS84-5	2	2	12783.5	19448.5	30653.5	67060	30735	43852	25173	NA	26087	NA	0.970424134	0.013038411	32136.1	31704	Down	-0.0195299834127959	Isoform 5a of Neurexin-1 OS=Mus musculus GN=Nrxn1
TNIK	P83510-2	13	13	24011.78	27443.44	37859.11	32679	29816.22	29643.22	30637.56	28254.78	30206.67	33547.67	0.970913975	0.012819248	30361.91	30457.98	Up	0.00455771386139613	Isoform 2 of Traf2 and NCK-interacting protein kinase OS=Mus musculus GN=Tnik
OLFM1	O88998-4	4	4	29639.5	31816.25	28541.5	22761.5	24224.25	27821.25	25169.5	39311.25	30311.75	13511.75	0.971021	0.012771378	27396.6	27225.1	Down	-0.009059513437461	Isoform 4 of Noelin OS=Mus musculus GN=Olfm1
GSTO1	O09131	6	6	42614.5	49229.83	66072.33	30684.67	45399.17	39259.83	47076.33	74389.33	46631.83	28415.67	0.971271127	0.012659521	46800.1	47154.598	Up	0.0108868410935241	Glutathione S-transferase omega-1 OS=Mus musculus GN=Gsto1 PE=2 SV=2
UBE2V2	Q9D2M8	24	24	32446.36	32490.64	29106.55	17365.05	29832.05	28061.5	28266.14	25982.86	34089.59	24234.68	0.971469502	0.012570829	28248.13	28126.954	Down	-0.00620204072809089	Ubiquitin-conjugating enzyme E2 variant 2 OS=Mus musculus GN=Ube2v2 PE=2 SV=4
SNAP25	P60879	87	87	36396.98	49148.94	22503.58	96112.25	76279.37	81237.36	87739.48	24809.66	76627.34	13792.82	0.971595723	0.012514406	56088.224	56841.332	Up	0.019242460921468	Synaptosomal-associated protein 25 OS=Mus musculus GN=Snap25 PE=1 SV=1
RTN4	Q99P72-3	23	23	23092.52	38744.09	25314.7	77009.04	38318.04	52655.39	53726.3	24641.83	39342.43	34169.26	0.971714435	0.012461346	40495.678	40907.042	Up	0.0145812782367088	Isoform 2 of Reticulon-4 OS=Mus musculus GN=Rtn4
ATP5F1	Q9CQQ7	12	12	42541.92	62688.08	42905.67	121011.83	62134.25	88575.92	89099.17	35395.75	62976	52002.5	0.971962808	0.012350353	66256.35	65609.868	Down	-0.01414591033243	ATP synthase F(0) complex subunit B1, mitochondrial OS=Mus musculus GN=Atp5f1 PE=1 SV=1
RAB2A	P53994	1	1	1673	5292	4427	6005	5166	5751	4770	NA	2892	NA	0.972005019	0.012331492	4512.6	4471	Down	-0.0133613568462477	Ras-related protein Rab-2A OS=Mus musculus GN=Rab2a PE=1 SV=1
SLC12A5	Q91V14	7	7	14008.17	17734.83	17845.67	45517.17	14078.67	29983.33	22759.17	23751.17	17814.17	13688.17	0.972463242	0.012126806	21836.902	21599.202	Down	-0.015790183877202	Solute carrier family 12 member 5 OS=Mus musculus GN=Slc12a5 PE=1 SV=2
PYGB	Q8CI94	2	2	20856.5	22830.5	NA	37924.5	37091	41304.5	38849	NA	10100.5	NA	0.972802986	0.011975105	29675.625	30084.667	Up	0.0197499571559683	Glycogen phosphorylase, brain form OS=Mus musculus GN=Pygb PE=1 SV=3
CASK	O70589-3	1	1	26903	47347	34887	64083	46021	56120	61411	13277	42797	NA	0.972835423	0.011960624	43848.2	43401.25	Down	-0.0147810258734402	Isoform 3 of Peripheral plasma membrane protein CASK OS=Mus musculus GN=Cask
NPTN	P97300	16	16	69746.8	105852.87	62617.8	196404.27	109912.93	157113.67	173246.8	79181.47	95531.67	45331.2	0.973174481	0.011809288	108906.93	110080.96	Up	0.0154691709639095	Neuroplastin OS=Mus musculus GN=Nptn PE=1 SV=3
NFS1	Q9Z1J3	18	18	44148	45855.67	40445.67	32380.44	44594.61	47475.5	40190.83	42414.22	55397.94	20853.39	0.973296443	0.011754864	41484.878	41266.376	Down	-0.00761879655462884	Cysteine desulfurase, mitochondrial OS=Mus musculus GN=Nfs1 PE=2 SV=3
RRBP1	Q99PL5	26	26	10568.4	18994.6	11626.52	40949.72	19429.2	29482.68	27999.44	12696.84	24060.56	6103.16	0.973426151	0.01169699	20313.688	20068.536	Down	-0.0175168123307735	Ribosome-binding protein 1 OS=Mus musculus GN=Rrbp1 PE=1 SV=2
PIP4K2A	O70172	6	6	31466.83	36938.83	36157.67	35335.83	38599.5	39888.33	44839.83	48524.33	43165.83	3522.33	0.973946103	0.011465076	35699.732	35988.13	Up	0.0116078956022724	Phosphatidylinositol 5-phosphate 4-kinase type-2 alpha OS=Mus musculus GN=Pip4k2a PE=1 SV=1
ATP2B2	Q9R0K7	34	34	18755	40760.55	20969.1	91438.06	49857.97	76052.58	70342.16	22273.84	42597.32	7368.45	0.973990066	0.011445473	44356.136	43726.87	Down	-0.0206136103597794	Plasma membrane calcium-transporting ATPase 2 OS=Mus musculus GN=Atp2b2 PE=1 SV=2
STXBP5	Q8K400	1	1	8653	14652	19043	33460	19750	27147	9924	NA	20921	NA	0.974511505	0.011213029	19111.6	19330.667	Up	0.0164428293221591	Syntaxin-binding protein 5 OS=Mus musculus GN=Stxbp5 PE=1 SV=3
CHCHD1	Q9CQA6	1	1	111264	120226	171170	51669	77577	80652	87862	61106	194376	112949	0.974779399	0.011093658	106381.2	107389	Up	0.0136030076595918	Coiled-coil-helix-coiled-coil-helix domain-containing protein 1 OS=Mus musculus GN=Chchd1 PE=3 SV=1
ERC2	Q6PH08	21	21	36691.81	33708.81	23369.76	46437.48	41194.43	46865.14	45315.67	26332.95	48441.33	13136.33	0.974844516	0.011064647	36280.458	36018.284	Down	-0.0104632190577259	ERC protein 2 OS=Mus musculus GN=Erc2 PE=2 SV=2

TJP2	Q9Z0U1	9	9	14790.5	12718.5	7480.83	12723.33	14735	17407.67	14847.67	8602.5	21415.33	811.33	0.974887545	0.011045478	12489.632	12616.9	Up	0.0146265111531682	Tight junction protein ZO-2 OS=Mus musculus GN=Tjp2 PE=1 SV=2
PHPT1	Q9DAK9	12	12	31116.18	35574.73	22893.73	34076.36	29080.91	35697.27	31372.45	31486.18	33582.36	20033.73	0.974927738	0.011027573	30548.382	30434.398	Down	-0.00539314026162315	14 kDa phosphohistidine phosphatase OS=Mus musculus GN=Phpt1 PE=2 SV=1
FAM49B	Q921M7	4	4	11444.75	21571.25	17783	45870	23496.75	30286.5	41502.75	16649.25	18801.75	11660.5	0.975354418	0.010837545	24033.15	23780.15	Down	-0.015267938083038	Protein FAM49B OS=Mus musculus GN=Fam49b PE=2 SV=1
NPTN	P97300-3	10	10	97457.8	149953.8	77078.1	281917.5	153139.5	224796.4	243235.9	95068	128593.7	59838.4	0.975543068	0.010753553	151909.34	150306.48	Down	-0.0153033673480174	Isoform 3 of Neuroplastin OS=Mus musculus GN=Nptn
STMN1	P54227	16	16	166570.12	161648.94	209123.25	126123.69	146474.62	140170.75	141720	215676	193503	122425	0.975583301	0.010735642	161988.12	162698.95	Up	0.00631689319122211	Stathmin OS=Mus musculus GN=Stmn1 PE=1 SV=2
CADPS	Q80TJ1-4	7	7	17190.17	16912.5	24013.5	39920.33	25419.33	30927.67	35151.5	9248.67	33551.5	13504.33	0.975861708	0.010611723	24691.166	24476.734	Down	-0.0125838990234193	Isoform 4 of Calcium-dependent secretion activator 1 OS=Mus musculus GN=Cadps
MRPL24	Q9CQ06	10	10	30815.1	33089	36560.1	22688	25739.4	25175.8	28936.7	30243.6	38755.4	26307	0.97644395	0.010352681	29778.32	29883.7	Up	0.00509642018521042	39S ribosomal protein L24, mitochondrial OS=Mus musculus GN=Mrpl24 PE=2 SV=1
CHCHD2	Q9D1L0	4	4	15330.75	15818.25	8886	18610.25	14770.5	17886.5	11577.5	22370	16091.25	5984	0.976487257	0.01033342	14683.15	14781.85	Up	0.00966533399816105	Coiled-coil-helix-coiled-coil-helix domain-containing protein 2, mitochondrial OS=Mus musculus GN=Chchd2 PE=2 SV=1
DMD	P11531	3	3	9602.33	11804.33	7034	25369.33	12271.33	18454.33	14994	14833	13475	4919	0.976512523	0.010322183	13216.264	13335.066	Up	0.0129105547681717	Dystrophin OS=Mus musculus GN=Dmd PE=1 SV=3
MRPL33	Q9CQP0	1	1	328563	311916	299526	222987	313152	282183	339124	350140	392983	119605	0.97656354	0.010299494	295228.8	296807	Up	0.00769165211534813	39S ribosomal protein L33, mitochondrial OS=Mus musculus GN=Mrpl33 PE=2 SV=1
AP2A2	P17427	6	6	20636.17	36252.83	19337	79199	42971.33	56476.67	59265.33	19785	36246.33	28633.5	0.976757294	0.010213337	39679.266	40081.366	Up	0.0145463388631368	AP-2 complex subunit alpha-2 OS=Mus musculus GN=Ap2a2 PE=1 SV=2
SPARCL1	P70663	5	5	16992.8	30098.8	14848.2	53252	21724.8	39973.8	28904.4	14654.6	24953.6	NA	0.976912484	0.010144341	27383.32	27121.6	Down	-0.0138550812700766	SPARC-like protein 1 OS=Mus musculus GN=Sparcl1 PE=2 SV=3
RPS8	P62242	25	25	131629.24	130774.16	131764.72	99477.56	128360.44	115705.72	131058.6	113640.44	172766.44	91089.84	0.976968856	0.01011928	124401.22	124852.21	Up	0.00522065501842462	40S ribosomal protein S8 OS=Mus musculus GN=Rps8 PE=1 SV=2
SOD1	P08228	5	5	82823.2	76914.6	72178.2	60046.6	79851.8	88881.8	69450	57889.6	92482.2	64298.4	0.97697473	0.01011667	74362.88	74600.4	Up	0.00460072061398733	Superoxide dismutase [Cu-Zn] OS=Mus musculus GN=Sod1 PE=1 SV=2
PLEC	Q9QXS1-11	86	86	7447.78	13038.59	6812.55	36149.15	17546.94	26064.76	22839.15	10995.39	13175.64	7015.16	0.978451331	0.009460771	16199.002	16018.02	Down	-0.0162091071297254	Isoform PLEC-1E,2A of Plectin OS=Mus musculus GN=Plec
CLINT1	Q99KN9	1	1	3839	5021	NA	37410	6235	17471	13104	NA	8063	NA	0.978479653	0.009448201	13126.25	12879.333	Down	-0.0273968979004903	Clathrin interactor 1 OS=Mus musculus GN=Clint1 PE=1 SV=2
CAMKV	Q3UHL1	39	39	9111.09	17026.94	5333.48	43131.24	15994.64	26392.88	27039.91	6039.18	13918.91	NA	0.978969234	0.009230956	18119.478	18347.72	Up	0.0180594023864546	CaM kinase-like vesicle-associated protein OS=Mus musculus GN=Camkv PE=1 SV=2
RPS19	Q9CZX8	15	15	132199.4	146146.47	128311.47	136509.07	131054.53	124282.6	145821.4	162256.53	174915.27	64194.33	0.979011821	0.009212064	134844.19	134294.03	Down	-0.00589821179855154	40S ribosomal protein S19 OS=Mus musculus GN=Rps19 PE=1 SV=3
SAFB	D3YXK2	2	2	39832.5	22818	78872.5	18530.5	27216	20571	27641.5	35755.5	46313.5	55264	0.979016131	0.009210153	37453.9	37109.1	Down	-0.0133429394175564	Scaffold attachment factor B1 OS=Mus musculus GN=Safb PE=1 SV=2
RAP1B	Q99JI6	3	3	56246	83586.67	90086	68836.33	56726.67	81337.33	77120.67	69022	57399.33	71690.67	0.979125637	0.009161578	71096.334	71314	Up	0.00441015612400791	Ras-related protein Rap-1b OS=Mus musculus GN=Rap1b PE=2 SV=2
DDX17	Q501J6-2	3	3	14660.33	10815	1330	23954	17906.33	18640	12018.67	2211.33	22695.33	NA	0.979262215	0.009101003	13733.132	13891.333	Up	0.0165243074284277	Isoform 2 of Probable ATP-dependent RNA helicase DDX17 OS=Mus musculus GN=Ddx17
IGLON5	Q8HW98	3	3	3885	4532.33	2181.33	9797.33	4956.67	6557	7208.33	1754.33	4951	NA	0.979403659	0.009038278	5070.532	5117.665	Up	0.0133485896417874	IgLON family member 5 OS=Mus musculus GN=Iglon5 PE=2 SV=2
TBC1D10B	Q8BHL3	3	3	32022	53295.67	48137	118065.33	63781.67	69922	81085.67	48563.67	54036.33	63775.33	0.979917742	0.008810379	63060.334	63476.6	Up	0.00949204380547182	TBC1 domain family member 10B OS=Mus musculus GN=Tbc1d10b PE=1 SV=2
POLDIP3	Q8BG81	2	2	14449.5	16591.5	11419.5	12698.5	16310	14391	16433.5	16476	15388	9001.5	0.980112895	0.008723897	14293.8	14338	Up	0.00445429008741471	Polymerase delta-interacting protein 3 OS=Mus musculus GN=Poldip3 PE=1 SV=1
FSCN1	Q61553	50	50	86012.14	93184.22	80362.82	102865.45	95649.22	100996	105302.14	88660.82	101177.65	60777.92	0.98037631	0.008607192	91614.77	91382.906	Down	-0.00365588491915701	Fascin OS=Mus musculus GN=Fscn1 PE=1 SV=4
NFASC	Q810U3	29	29	21452.41	31137.03	24308.9	58936.55	33031.76	46346.59	44651.9	30297.45	33153.59	15511	0.980510281	0.008547848	33773.33	33992.106	Up	0.00931531369003689	Neurofascin OS=Mus musculus GN=Nfasc PE=1 SV=1
RAP1GAP2	Q5SVL6-2	2	2	84425.5	92855.5	26188.5	61805	81153	89267	86133.5	14933.5	89017.5	NA	0.98074582	0.008443534	69285.5	69837.875	Up	0.0114562036861675	Isoform 2 of Rap1 GTPase-activating protein 2 OS=Mus musculus GN=Rap1gap2
PMPCB	Q9CXT8	7	7	14917.43	20603.57	13199	26446.29	15794	25599	23975.14	8725.71	19461.57	12699.86	0.980904357	0.008373336	18192.058	18092.256	Down	-0.00793644402803077	Mitochondrial-processing peptidase subunit beta OS=Mus musculus GN=Pmpcb PE=2 SV=1
MYH10	Q61879	76	76	14927.01	26705.46	16929.81	61605.42	31665.81	42877.25	46004.25	16718.25	35331.76	9546.61	0.981092118	0.008290213	30366.702	30095.624	Down	-0.0129365022020003	Myosin-10 OS=Mus musculus GN=Myh10 PE=1 SV=2
WDR48	Q8BH57	2	2	57751.5	72550	47508	100613.5	72998	97714.5	80367	34580.5	88085.5	48931	0.982097357	0.007845458	70284.2	69935.7	Down	-0.00717131101928753	WD repeat-containing protein 48 OS=Mus musculus GN=Wdr48 PE=1 SV=1
EPB41L2	O70318	15	15	70364.69	84231.54	52414.23	112264.23	94192.38	110739.54	88886.69	80629.31	74644.23	60082.62	0.982260043	0.007773522	82693.414	82996.478	Up	0.00527768365584565	Band 4.1-like protein 2 OS=Mus musculus GN=Epb41l2 PE=1 SV=2
TAOK2	Q6ZQ29-2	4	4	15124.5	17293.75	16132.75	12075.75	14713.25	17114	16337.5	969	25366	NA	0.982638593	0.007606183	15068	14946.625	Down	-0.0116681827672734	Isoform 2 of Serine/threonine-protein kinase TAO2 OS=Mus musculus GN=Taok2
NDUFA3	Q9CQ91	1	1	6082	6313	NA	10082	5365	8827	7752	NA	4420	NA	0.982647265	0.00760235	6960.5	6999.6667	Up	0.0080952764214573	NADH dehydrogenase [ubiquinone] 1 alpha subcomplex subunit 3 OS=Mus musculus GN=Ndufa3 PE=1 SV=1
VPS4A	Q8VEJ9	4	4	29159.5	33239.25	35554.75	28334.75	39547.25	35564.75	36101.25	23439.25	49865.25	20253.25	0.983552665	0.00720238	33167.1	33044.75	Down	-0.00533179299957845	Vacuolar protein sorting-associated protein 4A OS=Mus musculus GN=Vps4a PE=1 SV=1
CD44	P15379-11	1	1	48045	42626	144767	29464	55487	41668	56757	79704	76246	NA	0.983574173	0.007192884	64077.8	63593.75	Down	-0.0109396319014903	Isoform 9 of CD44 antigen OS=Mus musculus GN=Cd44
ERC2	Q6PH08-3	20	20	37634.05	34676.45	24538.25	46037.05	41197.5	46948.4	45289.6	27649.6	49589.6	13793.15	0.984008519	0.007001142	36816.66	36654.07	Down	-0.00638535018278731	Isoform 3 of ERC protein 2 OS=Mus musculus GN=Erc2
NCAM3	P13595-3	34	34	20088.42	34569.3	26158.55	97402.45	28710.88	58669.45	52548.85	42571.94	34610.64	20087.48	0.984866906	0.006622456	41385.92	41697.672	Up	0.0108268110319945	Isoform 3 of Neural cell adhesion molecule 1 OS=Mus musculus GN=Ncam1
RLBP1	Q9Z275	1	1	3721	1881	NA	10956	5406	7567	4906	NA	4133	NA	0.984924153	0.006597213	5491	5535.3333	Up	0.0116012862787634	Retinaldehyde-binding protein 1 OS=Mus musculus GN=Rlbp1 PE=2 SV=3
RAP1GAP2	Q5SVL6	3	3	57117.67	62953.67	17459	42101	54790.33	60421.33	58345.67	9955.67	59927.33	NA	0.985705109	0.006252992	46884.334	47162.5	Up	0.00853425600686952	Rap1 GTPase-activating protein 2 OS=Mus musculus GN=Rap1gap2 PE=2 SV=1
NPTN	P97300-4	5	5	63150	105624.8	58238.4	176318.4	109517.6	145961.6	157521	19353.6	84682.2	NA	0.986243418	0.006015882	102569.84	101879.6	Down	-0.0097413777063426	Isoform 4 of Neuroplastin OS=Mus musculus GN=Nptn
DCLK1	Q9JLM8	72	72	40177.23	54521.66	36077.59	67522.52	55762.03	55448.66	73308.59	38588.19	52132.53	33786.02	0.986303138	0.005989585	50812.206	50652.798	Down	-0.00453313578524775	Serine/threonine-protein kinase DCLK1 OS=Mus musculus GN=Dclk1 PE=1 SV=1
KTN1	Q61595-7	16	16	21136.31	31622.25	23492.5	70082.12	38715.81	54826.31	55684.12	23115.38	39316.69	11017.25	0.986468574	0.005916746	37009.798	36791.95	Down	-0.00851711919026753	Isoform 7 of Kinectin OS=Mus musculus GN=Ktn1
CADM3	Q99N28	14	14	16091.86	36338.71	14585.57	128749.21	38729.43	77542.43	74844.93	25824.07	29275.71	29095.29	0.986739957	0.005797285	46898.956	47316.486	Up	0.0127871268465927	Cell adhesion molecule 3 OS=Mus musculus GN=Cadm3 PE=1 SV=1
DCLK1	Q9JLM8-2	46	46	40373.41	53280.65	37816.91	62744.67	53601.52	50954.15	68788.7	39192.54	50847.74	38646.24	0.986792197	0.005774293	49563.432	49685.874	Up	0.00355965321940748	Isoform 2 of Serine/threonine-protein kinase DCLK1 OS=Mus musculus GN=Dclk1
KTN1	Q61595-11	17	17	20129.94	30245.41	22394.94	67827.24	37064	53141.71	53614.47	21755.65	37777.35	10369.18	0.987219753	0.005586164	35532.306	35331.672	Down	-0.00816929724882756	Isoform 11 of Kinectin OS=Mus musculus GN=Ktn1
EPS8	Q08509	13	13	7905.57	12904.71	2639.14	13083.43	10887.86	13508.14	11314.43	1620.43	11275.57	NA	0.987348616	0.005529478	9484.142	9429.6425	Down	-0.00831418756934283	Epidermal growth factor receptor kinase substrate 8 OS=Mus musculus GN=Eps8 PE=1 SV=2
GAD2	P48320	4	4	11214	13693.5	10851	36320.25	15549.75	26526.5	22915.5	11567.5	8696.25	NA	0.988127597	0.005186971	17525.7	17426.438	Down	-0.00819440021364796	Glutamate decarboxylase 2 OS=Mus musculus GN=Gad2 PE=2 SV=1
SNRPE	P62305	1	1	32630	20608	37769	10025	25907	13421	22060	39350	23928	27689	0.988156523	0.005174258	25387.8	25289.6	Down	-0.00559116412192644	Small nuclear ribonucleoprotein E OS=Mus musculus GN=Snrpe PE=2 SV=1
PTPRS	B0V2N1-4	20	20	28790.22	31391.56	31973.61	22857.89	31962.33	33930.5	35880.61	30676.17	35801.06	11062.33	0.988621699	0.004969862	29395.122	29470.134	Up	0.00367685478117248	Isoform 4 of Receptor-type tyrosine-protein phosphatase S OS=Mus musculus GN=Ptprs
ATP1B3	P97370	1	1	NA	32653	50489	285471	46872	136329	138621	NA	39779	NA	0.988621752	0.004969838	103871.25	104909.67	Up	0.0143512247468037	Sodium/potassium-transporting ATPase subunit beta-3 OS=Mus musculus GN=Atp1b3 PE=1 SV=1
RPS13	P62301	5	5	27561.6	45778.8	33055	76702.8	29624.6	47273.4	37041.2	22209.8	55941	49459.8	0.988683074	0.004942901	42544.56	42385.04	Down	-0.00541952354788106	40S ribosomal protein S13 OS=Mus musculus GN=Rps13 PE=1 SV=2
KCTD12	Q6WVG3	2	2	6723	4353	9883.5	17070.5	6657	8296.5	3462	NA	14874	NA	0.988747992	0.004914385	8937.4	8877.5	Down	-0.00970173939942113	BTB/POZ domain-containing protein KCTD12 OS=Mus musculus GN=Kctd12 PE=1 SV=1
ERC2	Q6PH08-2	14	14	38182.07	37436	26059.79	50298.93	43574.07	45853.71	46612	32342.64	56354.71	14980.07	0.988929261	0.004834773	39110.172	39228.626	Up	0.00436292445066415	Isoform 2 of ERC protein 2 OS=Mus musculus GN=Erc2
SYNE1	Q6ZWR6	2	2	904.5	3354.5	NA	7074	4092.5	4769	4763	NA	1968.5	NA	0.988987095	0.004809376	3856.375	3833.5	Down	-0.00858316849164687	Nesprin-1 OS=Mus musculus GN=Syne1 PE=1 SV=2
MRPL47	Q8K2Y7	2	2	19780.5	23892.5	33810.5	12977.5	24554	21630	24342	18063.5	39169.5	11404	0.989054807	0.004779642	23003	22921.8	Down	-0.0051016862085601	39S ribosomal protein L47, mitochondrial OS=Mus musculus GN=Mrpl47 PE=2 SV=2
OXSR1	Q6P9R2	2	2	42539.5	66378.5	42519.5	64967.5	70640.5	78878.5	73415.5	47645.5	72995.5	13140.5	0.989193435	0.004718774	57409.1	57215.1	Down	-0.00488349066210468	Serine/threonine-protein kinase OSR1 OS=Mus musculus GN=Oxsr1 PE=1 SV=1
MRPL23	O35972	2	2	11802.5	10571	4957	16180.5	7956	9853	10550.5	5292	13631.5	11980.5	0.989517436	0.004576549	10293.4	10261.5	Down	-0.0044779598784448	39S ribosomal protein L23, mitochondrial OS=Mus musculus GN=Mrpl23 PE=2 SV=1
DLG3	P70175	36	36	37222.03	41760.06	32431.12	47551.91	39738.38	42515.62	43630.03	35941.78	47432.09	28909.31	0.989696551	0.004497943	39740.7	39685.766	Down	-0.00199563258960236	Disks large homolog 3 OS=Mus musculus GN=Dlg3 PE=1 SV=1
MIA3	Q8BI84-2	5	5	1889.8	2912.2	1482.2	7730.8	4942.2	6464.4	6570.2	871.4	1362.2	NA	0.989913297	0.004402842	3791.44	3817.05	Up	0.00971219244126762	Isoform 2 of Melanoma inhibitory activity protein 3 OS=Mus musculus GN=Mia3
ATP1A2	Q6PIE5	128	128	27926.49	71716.52	34080.5	133245.4	71693.27	97373.51	112854.15	46031.37	49631.1	34274.51	0.990472049	0.004157776	67732.436	68032.928	Up	0.0063862978503461	Sodium/potassium-transporting ATPase subunit alpha-2 OS=Mus musculus GN=Atp1a2 PE=1 SV=1
TOMM34	Q9CYG7	5	5	7528.4	10368.8	5276.8	9404	12045.2	11097.6	9996.4	2753.2	11968.6	NA	0.990780476	0.00402256	8924.64	8953.95	Up	0.00473028684371995	Mitochondrial import receptor subunit TOM34 OS=Mus musculus GN=Tomm34 PE=2 SV=1
RGS10	Q9CQE5	2	2	5324	5939	1639	11947.5	7117	8193	6269	NA	4651	NA	0.991265626	0.003809954	6393.3	6371	Down	-0.00504095518657249	Regulator of G-protein signaling 10 OS=Mus musculus GN=Rgs10 PE=2 SV=1
NDUFV1	Q91YT0	19	19	62550.89	67568.26	54967.63	68186.74	62966.68	69478.42	69855.68	71580.47	63522.26	42133.32	0.991557191	0.003682232	63248.04	63314.03	Up	0.00150445476082467	NADH dehydrogenase [ubiquinone] flavoprotein 1, mitochondrial OS=Mus musculus GN=Ndufv1 PE=1 SV=1
MINK1	Q9JM52-2	8	8	15932.12	17469.12	18806.25	18417.12	18589.75	22127.38	22723.25	12486.62	22919.75	9123.5	0.99162195	0.003653869	17842.872	17876.1	Up	0.00268416933426517	Isoform 1 of Misshapen-like kinase 1 OS=Mus musculus GN=Mink1
NCDN	Q9Z0E0	7	7	19567.86	61719.43	22486.71	123418.29	49037.71	94617.29	101505.86	29551.71	40672.71	11265.29	0.991789048	0.003580692	55246	55522.572	Up	0.00720438793819909	Neurochondrin OS=Mus musculus GN=Ncdn PE=1 SV=1
DAAM2	Q80U19	1	1	NA	NA	NA	8655	4807	7947	5568	NA	NA	NA	0.991899584	0.003532292	6731	6757.5	Up	0.00566875008669208	Disheveled-associated activator of morphogenesis 2 OS=Mus musculus GN=Daam2 PE=2 SV=4
SRPK2	O54781	2	2	1715.5	3136	NA	11147.5	3581	5787.5	6032	NA	2934.5	NA	0.992621016	0.003216534	4895	4918	Up	0.00676287510502499	SRSF protein kinase 2 OS=Mus musculus GN=Srpk2 PE=1 SV=2
NDUFB4	Q9CQC7	14	14	60037.07	50420.64	65776.43	56705.29	49028.57	49955.64	42469.71	56588.79	59184.14	73487.07	0.992796976	0.003139554	56393.6	56337.07	Down	-0.00144690983381015	NADH dehydrogenase [ubiquinone] 1 beta subcomplex subunit 4 OS=Mus musculus GN=Ndufb4 PE=1 SV=3
RPS11	P62281	10	10	165766.1	167050.8	212509.1	161289.1	143809.9	141416.7	145169.3	139629.8	216452.7	208673.3	0.993196422	0.002964854	170085	170268.36	Up	0.00155445850917745	40S ribosomal protein S11 OS=Mus musculus GN=Rps11 PE=1 SV=3
PTPRS	B0V2N1-6	21	21	27574.11	30213.37	30479.68	22150.11	30718.47	32655.79	34449.53	29287.11	34478.42	10480.11	0.993230337	0.002950024	28227.148	28270.192	Up	0.00219831154370635	Isoform 6 of Receptor-type tyrosine-protein phosphatase S OS=Mus musculus GN=Ptprs
CUL3	Q9JLV5	4	4	20710.75	26968.25	17460.75	33391.25	28965	33385.25	37096	8205.5	23558.75	NA	0.993376837	0.002885971	25499.2	25561.375	Up	0.00351345861265881	Cullin-3 OS=Mus musculus GN=Cul3 PE=1 SV=1
PSMC4	P54775	9	9	26142.89	28519.22	17453.11	18059.56	23972.22	23773.78	32194	20862.67	25603.11	11872.22	0.993855625	0.0026767	22829.4	22861.156	Up	0.00200541359555489	26S protease regulatory subunit 6B OS=Mus musculus GN=Psmc4 PE=1 SV=2
PSMD6	Q99JI4	1	1	8112	19439	NA	44759	26369	26723	31755	NA	15743	NA	0.994077591	0.002579716	24669.75	24740.333	Up	0.00412184264292285	26S proteasome non-ATPase regulatory subunit 6 OS=Mus musculus GN=Psmd6 PE=1 SV=1
RASGRF2	P70392	1	1	144740	122440	104033	62572	120745	103938	117076	53280	170177	NA	0.994192823	0.002529376	110906	111117.75	Up	0.00275187467586275	Ras-specific guanine nucleotide-releasing factor 2 OS=Mus musculus GN=Rasgrf2 PE=1 SV=2
TTC1	Q91Z38	5	5	36988.6	29111.4	40592.2	20923.8	36754.8	29886	32606.4	23890.8	38897.6	38921.8	0.994269152	0.002496035	32874.16	32840.52	Down	-0.00147706008504729	Tetratricopeptide repeat protein 1 OS=Mus musculus GN=Ttc1 PE=2 SV=1
ACSL1	P41216	1	1	11135	24865	20855	47299	23853	36226	43503	5753	17214	NA	0.994720871	0.00229877	25601.4	25674	Up	0.00408537934214357	Long-chain-fatty-acid--CoA ligase 1 OS=Mus musculus GN=Acsl1 PE=1 SV=2
GNB4	P29387	11	11	61431	66423.73	51558.73	64761.27	52748.64	72961.82	59261.09	72538	67617.82	24212.91	0.994768507	0.002277972	59384.674	59318.328	Down	-0.00161271498677101	Guanine nucleotide-binding protein subunit beta-4 OS=Mus musculus GN=Gnb4 PE=2 SV=4
FOXO3	Q9WVH4	1	1	5348	4594	2277	3913	7462	5862	4312	3794	4933	NA	0.994871007	0.002233225	4718.8	4725.25	Up	0.00197063430637473	Forkhead box protein O3 OS=Mus musculus GN=Foxo3 PE=1 SV=1
ARHGEF12	Q8R4H2	2	2	2248	3488	1114	8617.5	3943.5	5244.5	2542	3374	4405.5	NA	0.994976309	0.00218726	3882.2	3891.5	Up	0.00345191357558296	Rho guanine nucleotide exchange factor 12 OS=Mus musculus GN=Arhgef12 PE=1 SV=2
PMCH	P56942	1	1	34105	49939	20096	126972	70202	78492	85575	26685	52332	58902	0.995173267	0.002101299	60262.8	60397.2	Up	0.00321396144018429	Pro-MCH OS=Mus musculus GN=Pmch PE=1 SV=2
CYB5B	Q9CQX2	5	5	11136.8	18310.8	4009.8	37756.2	15169	25228.6	26688.6	8919.4	8452.8	NA	0.995321577	0.002036581	17276.52	17322.35	Up	0.00382201802512107	Cytochrome b5 type B OS=Mus musculus GN=Cyb5b PE=1 SV=1
GEMIN5	Q8BX17	1	1	NA	1366	NA	8889	1600	4921	2949	NA	NA	NA	0.995454924	0.001978401	3951.6667	3935	Down	-0.00609762125336531	Gem-associated protein 5 OS=Mus musculus GN=Gemin5 PE=2 SV=2
ROCK2	P70336	13	13	12312.54	15996.46	7940	37647.54	21812.85	32221.23	28085.46	12066.23	20360.31	3179.23	0.995743358	0.001852582	19141.878	19182.492	Up	0.00305777437830342	Rho-associated protein kinase 2 OS=Mus musculus GN=Rock2 PE=1 SV=1
DPP3	Q99KK7	5	5	5018	7172	505.25	13263	7742.75	10966.5	8853.5	1730	10918.25	1315.25	0.995745389	0.001851696	6740.2	6756.7	Up	0.00352739963004519	Dipeptidyl peptidase 3 OS=Mus musculus GN=Dpp3 PE=2 SV=2
GLOD4	Q9CPV4-2	6	6	81178.83	84376.5	98280.5	57727	73242	66579.17	70469	76937	70248.33	110838.5	0.99607567	0.001707668	78960.966	79014.4	Up	0.000975961907310676	Isoform 2 of Glyoxalase domain-containing protein 4 OS=Mus musculus GN=Glod4
GSTP1	P19157	6	6	37440.4	29412.4	56336.2	39096.6	31883.2	37345.8	32931.6	34124.4	44577.4	45058.2	0.996248636	0.00163226	38833.76	38807.48	Down	-0.000976646615187786	Glutathione S-transferase P 1 OS=Mus musculus GN=Gstp1 PE=1 SV=2
RALB	Q9JIW9	3	3	13541	19514	5476.5	17759.5	13408	16016	14395.5	16533	8758.5	NA	0.996409176	0.001562282	13939.8	13925.75	Down	-0.00145483344432012	Ras-related protein Ral-B OS=Mus musculus GN=Ralb PE=2 SV=1
TKFC	Q8VC30	2	2	15692	24269.5	17488.5	11936	12327.5	16781	20481	7809.5	20367.5	NA	0.996496934	0.001524033	16342.7	16359.75	Up	0.0015043492261851	Bifunctional ATP-dependent dihydroxyacetone kinase/FAD-AMP lyase (cyclizing) OS=Mus musculus GN=Dak PE=2 SV=1
BCAS3	Q8CCN5-2	3	3	10193.33	11026.33	8578.67	13201	16882	14093	14823	1036.33	13825	16033.33	0.996519811	0.001514063	11976.266	11962.132	Down	-0.00170362729180384	Isoform 2 of Breast carcinoma-amplified sequence 3 homolog OS=Mus musculus GN=Bcas3
NACAD	Q5SWP3	5	5	15493.6	19552.6	23636.8	10692	17425.8	14605	16650.4	8942.4	29160.8	NA	0.996756169	0.001411068	17360.16	17339.65	Down	-0.00170546632586632	NAC-alpha domain-containing protein 1 OS=Mus musculus GN=Nacad PE=2 SV=1
PAG1	Q3U1F9	1	1	5495	4512	8028	12167	7167	9854	4747	NA	7844	NA	0.997002542	0.001303734	7473.8	7481.6667	Up	0.00151773291840249	Phosphoprotein associated with glycosphingolipid-enriched microdomains 1 OS=Mus musculus GN=Pag1 PE=1 SV=2
ANK2	Q8C8R3-8	16	16	17986.94	35860.06	22543.62	77645.31	48424.5	62257.81	69644.31	17965.94	36618.12	15715.31	0.997402036	0.00112975	40492.086	40440.298	Down	-0.00184633881475959	Isoform 8 of Ankyrin-2 OS=Mus musculus GN=Ank2
ATP2A1	Q8R429	2	2	18457.5	53010.5	24037.5	195034.5	33010	119649.5	96333	30435	39941.5	36639.5	0.997760867	0.000973534	64710	64599.7	Down	-0.00246121213805128	Sarcoplasmic/endoplasmic reticulum calcium ATPase 1 OS=Mus musculus GN=Atp2a1 PE=2 SV=1
DECR1	Q9CQ62	2	2	69275	77297	69010.5	119778.5	56320.5	80957.5	68793.5	94634.5	66857.5	80341	0.998757842	0.000539798	78336.3	78316.8	Down	-0.000359170082719599	2,4-dienoyl-CoA reductase, mitochondrial OS=Mus musculus GN=Decr1 PE=1 SV=1
RPL24	Q8BP67	15	15	146391.53	133316.27	161943	118968.33	126757.6	114040.47	139651.67	153520.27	155500.8	124586.53	0.998925307	0.000466984	137475.35	137459.95	Down	-0.000161598883428967	60S ribosomal protein L24 OS=Mus musculus GN=Rpl24 PE=1 SV=2
NCDN	Q9Z0E0-2	5	5	27084.4	86017.2	31481.4	171076.4	68129.4	130842.8	140252.4	41372.4	55358.4	15771.4	0.999178303	0.000357005	76757.76	76719.48	Down	-0.000719668496911045	Isoform 2 of Neurochondrin OS=Mus musculus GN=Ncdn
KTN1	Q61595-16	20	20	17140.5	26188	19035.7	58999.45	32156.2	46353.05	46663.45	18616.7	33100.8	8813.8	0.999594542	0.000176124	30703.97	30709.56	Up	0.00026263480704782	Isoform 16 of Kinectin OS=Mus musculus GN=Ktn1
KTN1	Q61595-2	19	19	17830.63	27133.89	19783.16	60433.68	33288.89	47414.37	48040.47	19596.53	34150.95	9277.68	0.999860947	6.03941E-05	31694.05	31696	Up	0.0000887601549650539	Isoform 2 of Kinectin OS=Mus musculus GN=Ktn1
SPATS2	Q8K1N4	3	3	698.67	588	NA	6898.67	3299	3909.67	3791.67	NA	912.67	NA	0.99989263	4.66325E-05	2871.085	2871.3367	Up	0.000126454751415087	Spermatogenesis-associated serine-rich protein 2 OS=Mus musculus GN=Spats2 PE=1 SV=1
PACSIN3	Q99JB8	8	8	8110	9516	6765.2	15672.4	11488.6	11904.4	11135.4	12766	13537.4	2208.4	0.999964163	1.55643E-05	10310.44	10310.32	Down	-0.0000167911759706074	Protein kinase C and casein kinase II substrate protein 3 OS=Mus musculus GN=Pacsin3 PE=1 SV=1
